# Insights into the molecular evolution of peptidase inhibitors in arthropods

**DOI:** 10.1371/journal.pone.0187643

**Published:** 2017-11-06

**Authors:** Joaquin Alonso, Manuel Martinez

**Affiliations:** 1 Centro de Biotecnología y Genómica de Plantas, Universidad Politécnica de Madrid (UPM)—Instituto Nacional de Investigación y Tecnología Agraria y Alimentaria (INIA), Campus Montegancedo UPM, Pozuelo de Alarcón (Madrid), Spain; 2 Departamento de Biotecnología-Biología Vegetal, Escuela Técnica Superior de Ingeniería Agronómica, Alimentaria y de Biosistemas, UPM, Madrid, Spain; Oklahoma State University, UNITED STATES

## Abstract

Peptidase inhibitors are key proteins involved in the control of peptidases. In arthropods, peptidase inhibitors modulate the activity of peptidases involved in endogenous physiological processes and peptidases of the organisms with which they interact. Exploring available arthropod genomic sequences is a powerful way to obtain the repertoire of peptidase inhibitors in every arthropod species and to understand the evolutionary mechanisms involved in the diversification of this kind of proteins. A genomic comparative analysis of peptidase inhibitors in species belonging to different arthropod taxonomic groups was performed. The results point out: i) species or clade-specific presence is shown for several families of peptidase inhibitors; ii) multidomain peptidase inhibitors are commonly found in many peptidase inhibitor families; iii) several families have a wide range of members in different arthropod species; iv) several peptidase inhibitor families show species-specific (or clade-specific) gene family expansions; v) functional divergence may be assumed for particular clades; vi) passive expansions may be used by natural selection to fix adaptations. In conclusion, conservation and divergence of duplicated genes and the potential recruitment as peptidase inhibitors of proteins from other families are the main mechanisms used by arthropods to fix diversity. This diversity would be associated to the control of target peptidases and, as consequence, to adapt to specific environments.

## Introduction

In the genetic context, a peptidase inhibitor is a protein able to attenuate peptidase activity by making a complex with it. At present, the most accepted way to classify peptidase inhibitors is the MEROPS database of peptidases and their inhibitors[1]. The MEROPS database assigns every peptidase or peptidase inhibitor to a family on the basis of statistically significant similarities in amino acid sequence, and families are grouped in a clan when structural information supports their homolog relationships. The MEROPS database is periodically updated and in the most recent version 78 peptidase inhibitor families and 38 clans are included. The peptidase inhibitor usually has an only inhibitory structural domain, but there are many families that include inhibitors with more than one inhibitory domain.

Arthropoda is one of the most abundant and diverse phylum in the animal kingdom and includes the hexapods, arachnids, myriapods and crustaceans. Despite the low information regarding peptidase inhibitors in arthropods comparing with that existing for mammals or plants, a review of the association of peptidase inhibitor with proteolytic signalling cascades in insects reveals their key role in many physiological events[2]. In the last few years several peptidase inhibitor families have been genetically and/or physiologically analysed in some single arthropod species, with special emphasis in *Drosophila melanogaster* and *Bombyx mori* [2, 3]. The most studied inhibitor families are the I1, I2, I4 and I25 (the MEROPS classification). The I1 family is also known as the Kazal family. Kazal inhibitors are widely present in most arthropod clades[4] where diverse roles are performing, such as anticoagulants in hematophagous or defence proteins against predators and pathogens in several species[5]. Similar roles have been assigned to inhibitors belonging to the family I2 or Kunitz[6], which has been extensively analysed in ticks[7]. The I4 family or serpins has been characterized in depth in Drosophilids, where they have multiple functions in immunity and morphogenesis[8, 9]. The set of serpin members has also been reported for several insects and ticks[10–13]. Finally, I25 cystatins have been characterized in ticks and mites, where they perform different roles such as endogenous peptidase regulation, suppression of host immunity, defence against pathogens, embryogenesis and food digestion[14, 15]. Their presence in most arthropod taxonomic groups has been reported[14]. Besides, scattered information on members of many other peptidase inhibitor families in single arthropod species can be found in the databases.

Comparative genomics is a powerful tool to understand the evolutionary features of protein families. The rapid development of sequencing technologies has allowed the generation of genome draft sequences for many organisms, including many arthropod species. The first insect whose genomic sequence was obtained and annotated was *Drosophila melanogaster* in the year 2000[16]. With the appearance of the genomic sequence for the insect *Anopheles gambiae* in 2002[17] comparative genomics was possible in arthropods[18]. From this date, the number of sequenced genomes of insects has grown exponentially, and in the recently developed InsectBase, the sequences of 138 insect species are available[19]. Likewise, efforts have been done to sequence the genome of species from other arthropod groups. The first crustacean was *Daphnia pulex* whose first version of the genome sequence was publicly available in 2007 and was latterly published in 2011[20]. Recently, the preliminary genomic sequences of several other crustacean species have been submitted to databases (http://www.ncbi.nlm.nih.gov/assembly/organism/6657/all/). In 2011, the genome of the acari *Tetranychus urticae* was published[21]. The first scorpion was *Mesobuthus martensii* in 2013[22]. Afterwards, several other arachnid genome sequences, mainly for mites and ticks, have been sent to databases and some of them have also been published (http://www.ncbi.nlm.nih.gov/assembly/organism/6854/all/). Finally, the first genome sequence of a myriapod species, the centipede *Strigamia maritima*, was published in 2014[23]. To date, only the genomic sequence of a second myriapod, the millipede *Trigoniulus corallinus*, is available[24]. Several of these species are in the international initiative named i5k, which started in 2011 with the main goal of sequencing the genomes of 5000 arthropod species to facilitate comparative analyses[25].

Gene duplications are a major source of new material that can give rise to functional innovations[26]. Different evolutionary models have been postulated to explain how new genes evolved to form the extant protein families (reviewed in [27]). The “divergent evolution” model was formerly proposed to explain the functional diversity of phylogenetically related proteins. As data collection increased, the “concerted evolution” model was proposed to deal with the higher intraspecies than interspecies similarities found for the members of a repeated gene family. Lately, genome sequencing revealed a high intraspecific diversity in multigene families, which was associated to interspecies clustering patterns in phylogenies and the presence of pseudogenes. The new model was termed “birth-and-death” model. It is based in the stochastic gain and loss of genes after duplication and speciation events associated to genomic drift, natural selection and concerted evolution [28].

As above stated, peptidase inhibitors are key proteins in many physiological processes in arthropods. Besides, many genome projects covering the main groups of arthropods have been annotated and sequences are available in the databases. Using these repositories, we have extracted the amino acid sequences of the different proteins belonging to peptidase inhibitor families in selected arthropod species. Evolutionary analyses have provided further insights on the mechanisms involved in inhibitory peptidases diversification, which include genomic drift, natural selection and functional divergence. Diversity in the repertoire of peptidase inhibitors may be related to the biological diversity of arthropod species.

## Materials and methods

### Identification of peptidase inhibitor families in arthropods

The MEROPS v11.0 database of peptidases and their inhibitors was used to establish the set of protein peptidase inhibitor families in arthropods[1]. For that, the information available in the MEROPS on the phylogenetic distribution of the 78 peptidase inhibitor families was analysed. Besides, BlastP searches were performed in the Uniprot database using the amino acid sequence of a protein representative of each peptidase inhibitor family. Using this information, the peptidase inhibitor families present in arthropods were identified.

### Arthropod species selection

**Fig 1:**
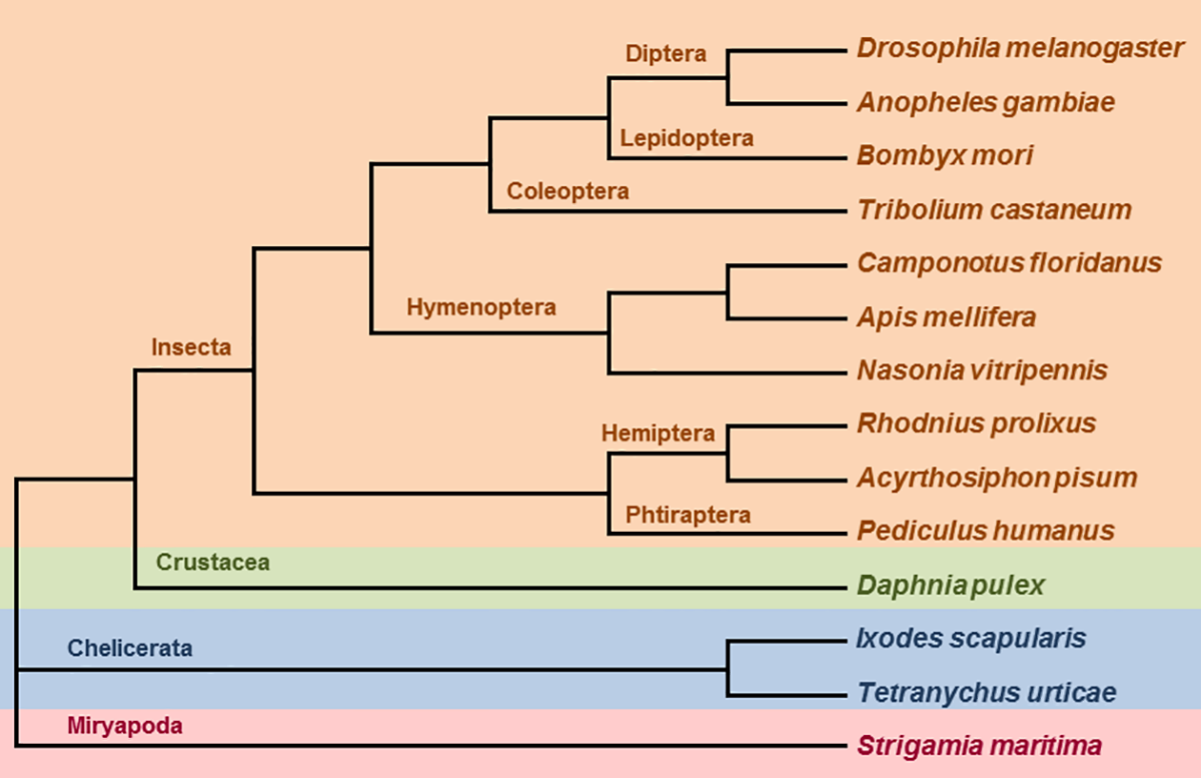
Phylogeny of selected arthropod species. Schematic evolutionary tree of selected fully sequenced arthropods. Insect species are coloured in orange, crustacean in green, acari in blue, and myriapod in pink.

The selection of the arthropod species used in this work was based in two criteria: i) their genomes are fully sequenced and an accurate proteome annotation is available in the genomic database, ii) the selected species cover the highest number of the groups and subgroups included within the phylum Arthropoda. With these criteria, the selected species were: ten species belonging to the main orders included in the Insecta class (two dipteran, *Drosophila melanogaster* and *Anopheles gambiae*; a lepidopteran, *Bombyx mori*; a coleopteran, *Tribolium castaneum*; three hymenopteran, *Camponotus floridanus*, *Apis mellifera* and *Nasonia vitripennis*; two hemipteran, *Rhodnius prolixus* and *Acyrthosiphon pisum*; and a phtirapteran, *Pediculus humanus*); a crustacean species, *Daphnia pulex*; two chelicerates (*Ixodes scapularis* and *Tetranychus urticae*); and a miryapod, *Strigamia maritima*. Their phylogenetic relationships based on the current consensus classification in the systematic literature are shown in Fig 1. All the genomes of these species were accessible at different genomic databases. Gene prediction quality varies among the annotation stage of the different genomes and the gene family distribution and size could slightly be modified if new annotation versions are released.

### Sequence searches in genomic databases

To know the set of members of a family present in a species the most accurate way is to explore its genomic sequence[29]. The improved accuracy of annotation tools, mainly in well-characterized protein families, leads us to explore the proteome annotation of the chose genomes. Thus, Blastp searches for selected peptidase inhibitor families were performed in the selected arthropod publicly available genome databases (S1 Table). NCBI Blastp searches were performed in the species RefSeq database under the Blast genomes option tool. Blastp searches were made in a recurrent way similar to that previously described[30]. First, a search using a complete amino acid arthropod sequence from data banks corresponding to a protein of the selected family was performed. Then, the sequences obtained from each arthropod species with an e-value lesser than 1 were employed to repeat the search in the same species. Finally, after an alignment of the sequences found in all the arthropods selected, the most conserved region was used to a final search in the complete set of annotated proteins of each arthropod species. When putative variants were retrieved for a protein, the longest isoform was selected. To test the accuracy of the results and to know the combination of domains within each protein, all retrieved sequences were subjected to a sequence search in the Pfam database v29.0[31] using the HMMER algorithm with default parameters. The following Pfam domains were used to assign each protein to a family: I1, Kazal_2 (PF07648); I2 Kunitz_BPTI (PF00014.22); I4, Serpin (PF00079.19); I8, TIL (PF01826.16); I17, WAP (PF00095.20); I19, Pacifastin_I (PF05375.12); I21, Secretogranin_V (PF05281.10); I25, Cystatin (PF00031.20); I31, Thyroglobulin_1 (PF00086.17); I32, BIR (PF00653.20); I35, TIMP (PF00965.16); I39, A2M (PF00207.21); I51, PBP (PF01161.19); I63, Lectin_C (PF00059.20); I87, Band_7 (PF01145.24); I93, Fz (PF01392.21).

### Protein alignments and phylogenetic trees

Alignments of the amino acid domains (S1 Fig) were performed using the default parameters of MUSCLE v3.8[32] (S2 Fig). Domains including sequence-specific gaps that cover more than 10% of the amino acid sequence were manually excluded from phylogenetic studies. Phylogenetic and molecular evolutionary analyses were conducted using the programs available on Phylogeny.fr website [33]. The displayed protein peptidase inhibitor trees were constructed by means of the maximum likelihood PhyML v3.0 method [34] using a BIONJ starting tree. The approximate likelihood-ratio test (aLRT) based on a Shimodaira-Hasegawa-like procedure was applied as statistical test for non-parametric branch support[35]. Trees were rooted using sequences from animals other than arthropods retrieved from UniProt database. All families were also analysed with the Maximum parsimony and the Neighbour-Joining algorithms, and with different gap/missing data treatments. Trees were visualized and edited using MEGA v6 program [36]. Visual differences in the tree topologies were not detected and most branches were composed by the same individual domains. Information about gene models for all proteins used to construct the phylogenetic trees is compiled in S3 Fig.

### Functional divergence analyses

To assess functional divergence DIVERGE v.3 software was used [37]. The Gu’s type-I functional divergence was measured as it is an indicator of functional changes between members of a multigene family [38]. The coefficient of divergence (θ_D_) and the LRT (likelihood-ratio test) of the coefficient of divergence were calculated to assess if there has been a significant change in evolution rates after duplication or speciation events.

## Results

### Protein peptidase inhibitors families in arthropods

Firstly, the protein peptidase inhibitor families presented in arthropods were determined by searches in the MEROPS and Uniprot databases. Thirty-three families with members in arthropods were identified. Fourteen peptidase inhibitor families were restricted to a specific clade or even to a specific group of species inside a clade. Some of them were restricted to species included in the order Ixodida (I52, I53, I68, I72, I74), others to dipteran species (I64, I76, I77), one to the subfamily Triatominae of heteropteran (I59), one to the family Scarabaeidae of coleopteran (I88), and four were found in species belonging to various insect groups (I11, I44, I71, I83). The other nineteen families had members in most arthropod clades and were selected for a deeper analysis. The I29 family comprises the inhibitory propeptides of the C1A papain peptidase family, which are almost always contained in the same molecule. Only two proteins from *D*. *melanogaster*, one from *Nasonia vitripennis*, two from *Tribolium castaneum* and one from *T*. *urticae* putatively had one or several I29 domains not linked to a C1A peptidase. Then, this family was excluded for the analysis. Families I15 and I43 were also excluded due to the high variability of the proteins included in these families that made difficult to select which are actual peptidase inhibitors.

To get the complete set of members for the rest of families, several species were selected covering most groups and subgroups within the phylum Arthropoda (Fig 1). Genome extensive searches were done to know their distribution and the number of members in each arthropod species (Table 1). Ten of the sixteen selected families included proteins with more than one inhibitory domain in their sequences. This feature was remarkable for several families, such as the I1 family in which a sequence from *S*. *maritima* putatively had 24 Kazal domains. Total numbers showed a wide range of inhibitory domains and sequences among the different families. There were families with more than one hundred and fifty sequences (I1, I4 and I63) whereas some others had less than fifteen sequences (I21 and I35). Likewise, the number of total inhibitory domains ranked from 11 in I35 family to 449 in I1 family. The content of several families among species also showed a strong variability. For example, the number of sequences in the I2 family ranged between three in *Rhodnius prolixus* and 36 in *Ixodes scapularis*. These differences were also detected in the number of inhibitory domains, which ranked from six in *T*. *urticae* to 55 in *D*. *pulex*. This species diversity was found in several other peptidase inhibitor families, such as I1, I4, I8, I19, I25, I32 and I63. On the contrary, the content of several families was mainly conserved among different species/groups of arthropods. For example, there was only an inhibitor of the I21 family in all the species selected. Small differences were detected in some other families, such as I35, I39, I51 and I93. Strong differences in the content of different peptidase inhibitor families were also found between species belonging to the same taxonomic group. For example, the dipteran *D*. *melanogaster* had 21 I2 and 6 I8 sequences whereas the dipteran *A*. *gambiae* had 5 I2 and 21 I8 sequences. In the acari, this variability was also found. *I*. *scapularis* had considerably more sequences of the I2 and I8 families than *T*. *urticae*, whereas the contrary was observed for the I25 and I32 families.

**Table 1:** Number of sequences and domains (in brackets) for every peptidase inhibitor family in selected arthropod species.

	**I1**	**I2**	**I4**	**I8**	**I17**	**I19**	**I21**	**I25**
	**(Kazal)**	**(Kunitz-A)**	**(Serpin)**	**(Ascaris)**	**(WAP)**	**(Pacifastin)**	**(7B2)**	**(Cystatin)**
***Drosophila melanogaster***	12 (24)	21 (32)	26 (26)	6 (9)	2 (3)	0 (0)	1 (1)	4 (5)
***Anopheles gambiae***	10 (20)	5 (14)	15 (15)	21 (23)	3 (4)	1 (1)	1 (1)	2 (7)
***Bombyx mori***	18 (50)	13 (25)	22 (22)	17 (52)	4 (5)	4 (15)	1 (1)	1 (10)
***Tribolium castaneum***	10 (28)	8 (17)	12 (12)	7 (14)	5 (7)	2 (6)	1 (1)	1 (9)
***Camponotus floridanus***	7 (26)	4 (14)	5 (6)	2 (6)	3 (4)	1 (1)	1 (1)	1 (2)
***Apis mellifera***	6 (22)	4 (14)	6 (6)	9 (13)	4 (7)	1 (1)	1 (1)	1 (2)
***Nasonia vitripennis***	23 (58)	5 (15)	7 (8)	8 (12)	1 (2)	12 (40)	1 (1)	1 (3)
***Rhodnius prolixus***	15 (32)	3 (12)	3 (3)	1 (5)	2 (2)	7 (15)	1 (1)	2 (2)
***Acyrthosiphon pisum***	9 (28)	4 (11)	15 (16)	1 (5)	1 (1)	1 (1)	1 (1)	1 (2)
***Pediculus humanus***	9 (24)	4 (17)	9 (9)	1 (6)	1 (1)	3 (3)	1 (1)	2 (2)
***Daphnia pulex***	7 (26)	22 (55)	5 (5)	1 (4)	1 (5)	0 (0)	1 (1)	10 (39)
***Ixodes scapularis***	7 (15)	36 (50)	20 (20)	32 (43)	0 (0)	0 (0)	1 (1)	11 (11)
***Tetranychus urticae***	8 (40)	5 (6)	16 (16)	9 (18)	0 (0)	0 (0)	1 (1)	25 (25)
***Strigamia marítima***	10 (46)	6 (19)	8 (9)	2 (5)	7 (14)	0 (0)	1 (1)	2 (2)
**TOTAL**	**151 (439)**	**140 (301)**	**173 (177)**	**117 (215)**	**34 (56)**	**32 (83)**	**14 (14)**	**64 (121)**
	**I31**	**I32**	**I35**	**I39**	**I51**	**I63**	**I87**	**I93**
	**(Thyropin)**	**(IAP)**	**(Timp)**	**(Alpha 2M)**	**(IC)**			
***Drosophila melanogaster***	3 (8)	4 (7)	1 (1)	4 (4)	9 (9)	32 (32)	11 (11)	7 (7)
***Anopheles gambiae***	5 (8)	8 (11)	1 (1)	5 (5)	7 (7)	18 (19)	5 (5)	6 (6)
***Bombyx mori***	2 (6)	4 (7)	1 (1)	0 (0)	6 (6)	11 (16)	4 (4)	5 (5)
***Tribolium castaneum***	3 (9)	4 (7)	1 (1)	2 (2)	7 (7)	9 (10)	6 (6)	6 (6)
***Camponotus floridanus***	4 (9)	6 (12)	1 (1)	2 (2)	3 (3)	9 (9)	3 (3)	7 (7)
***Apis mellifera***	3 (9)	0 (0)	1 (1)	2 (2)	3 (3)	9 (9)	3 (3)	7 (7)
***Nasonia vitripennis***	3 (9)	11 (14)	0 (0)	2 (2)	8 (8)	22 (25)	4 (4)	6 (6)
***Rhodnius prolixus***	1 (1)	3 (6)	0 (0)	1 (1)	3 (3)	6 (6)	7 (7)	6 (6)
***Acyrthosiphon pisum***	2 (3)	10 (12)	0 (0)	2 (2)	5 (5)	6 (7)	6 (6)	4 (4)
***Pediculus humanus***	2 (3)	3 (6)	0 (0)	1 (1)	3 (3)	11 (11)	5 (5)	6 (6)
***Daphnia pulex***	5 (11)	5 (7)	0 (0)	2 (2)	4 (4)	29 (29)	4 (4)	6 (6)
***Ixodes scapularis***	2 (3)	5 (7)	1 (1)	1 (1)	6 (6)	3 (3)	9 (9)	5 (5)
***Tetranychus urticae***	5 (12)	16 (17)	1 (1)	2 (2)	6 (6)	4 (4)	14 (14)	7 (7)
***Strigamia marítima***	7 (17)	7 (9)	1 (1)	3 (3)	2 (2)	20 (27)	5 (5)	9 (9)
**TOTAL**	**46 (108)**	**86 (122)**	**9 (9)**	**29 (29)**	**72 (72)**	**187 (205)**	**90 (90)**	**85 (85)**

Insect species are coloured in orange, crustacean in green, acari in blue, and myriapod in pink.

### Total peptidase inhibitors in the selected arthropod species

**Fig 2:**
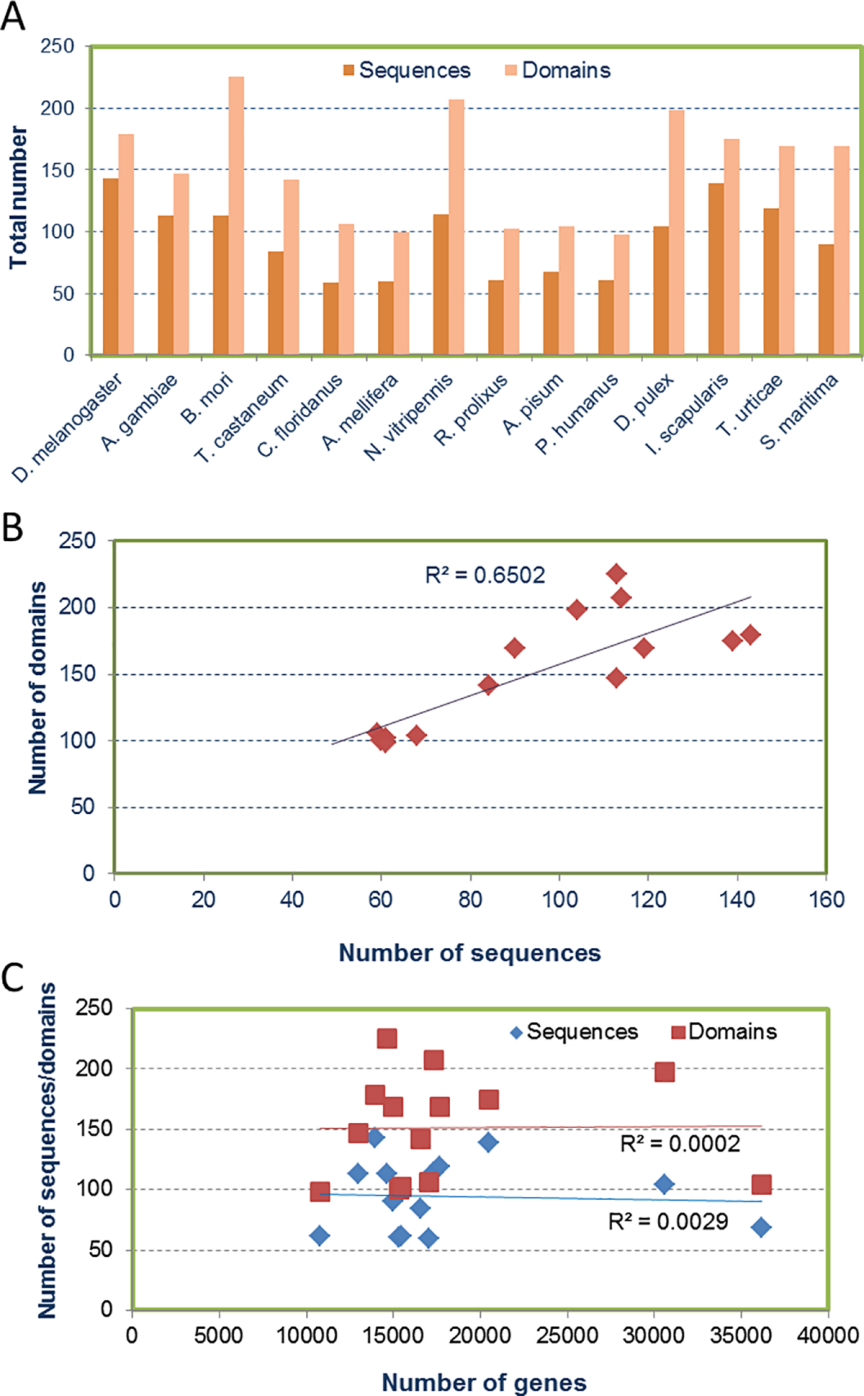
Total peptidase inhibitors in the selected arthropod species. (A) Total numbers of inhibitory protein sequences and inhibitory domains. (B) Dispersion graph showing the linear trend of the two variables (number of domains and number of sequences). The coefficient of determination (R^2^) is included. (C) Dispersion graph showing the linear trends of the two variables (number of sequences/domains and number of genes). The coefficients of determination (R^2^) are included.

The total number of peptidase inhibitors in the individual species may offer clues on the evolutionary features of arthropods. Huge differences were detected in the content of peptidase inhibitors among species (Fig 2A). The number of peptidase inhibitor sequences in *D*. *melanogaster* and *I*. *scapularis* more than duplicated the number of sequences in *Camponotus floridanus*, *Apis mellifera*, *Rhodnius prolixus*, *Acyrtosiphon pisum* and *Pediculus humanus*. Similarly, the number of peptidase inhibitor domains in the species with the lowest number of sequences was half of the number of domains in species such as *B*. *mori*, *N*. *vitripennis* or *D*. *pulex*. Interestingly, whereas the species with a minor number of sequences also had a minor number of domains, the species with a higher number of sequences were not the same that the species with a higher number of domains. This particularity is shown in Fig 2B, where a moderate positive correlation between the number of peptidase inhibitor sequences and the number of peptidase inhibitor domains is observed. On the other hand, there were not remarkable differences among the total numbers of peptidase inhibitors of the species representing Crustacea, Chelicerata and Miryapoda taxonomic groups (Fig 2A), although the content of inhibitors in the different families varied among these species (Table 1). For insects, a great diversity was observed in the number of sequences between the different taxonomic groups. Hymenopteran, hemipteran and phtirapteran species had considerably lower number of sequences than dipteran, lepidopteran and coleopteran species. But also variability was found inside these taxonomic groups, since the hymenopteran *N*. *vitripennis* had a remarkably higher number of sequences and domains than *C*. *floridanus* or *A*. *mellifera* (Fig 2A). There were no correlation between the total number of peptidase inhibitor sequences/domains and the number of genes present in the genomic sequence of every species (Fig 2C).

### Phylogenetic features of peptidase inhibitor families in arthropods

**Fig 3:**
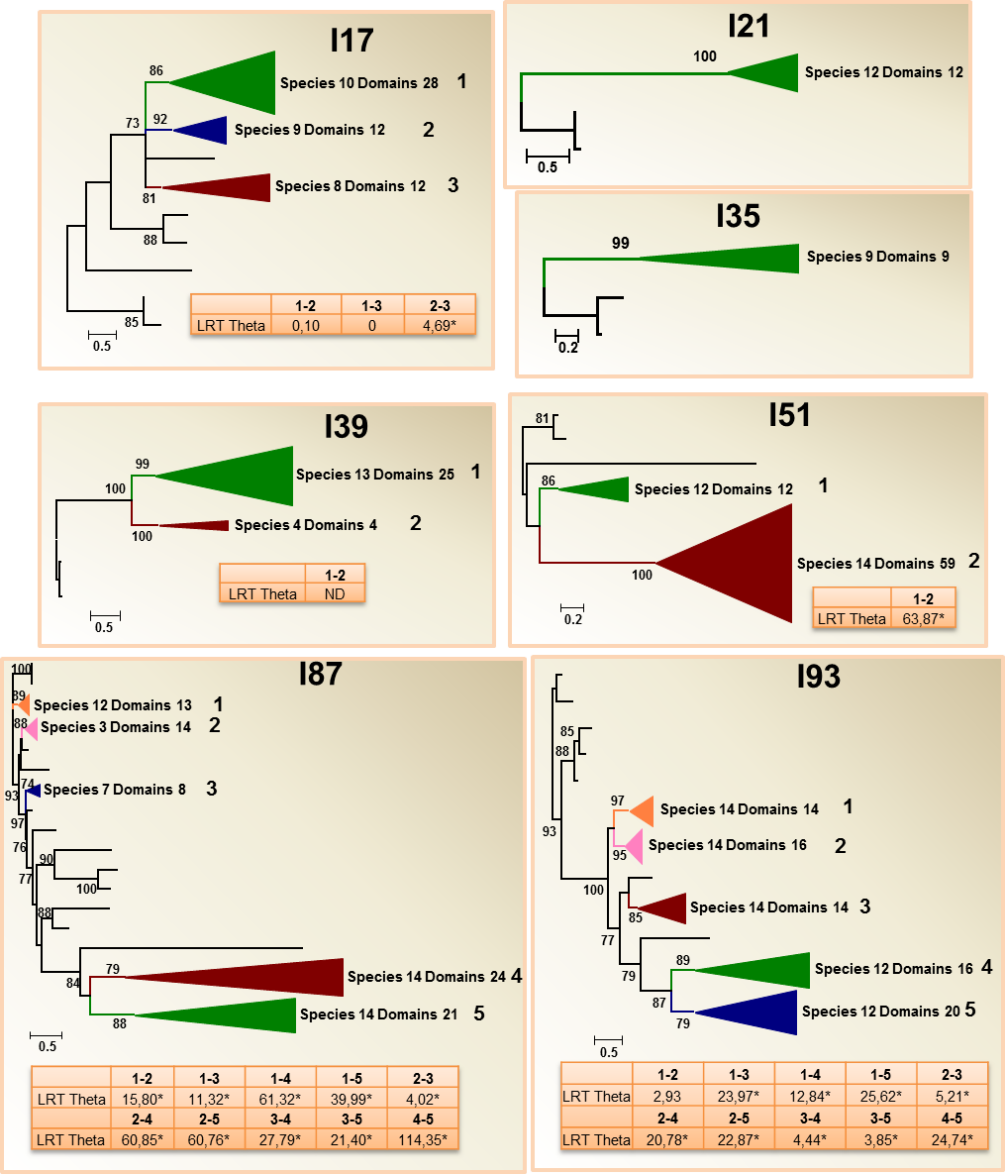
Phylogenetic distribution and divergence rates in peptidase inhibitory families without species-specific clades. Schematic PhyML phylogenetic trees using the inhibitory domains from the selected arthropod species are depicted. Coloured triangles show collapsed branches. Triangle size is proportional to the number of nodes (domains) included. The number of species with any domain in the collapsed branch is indicated. aLRT values over 50 are shown. LRT Theta values for Type I functional divergence between numbered clades are included. Complete phylograms are shown in S3 Fig.

**Fig 4:**
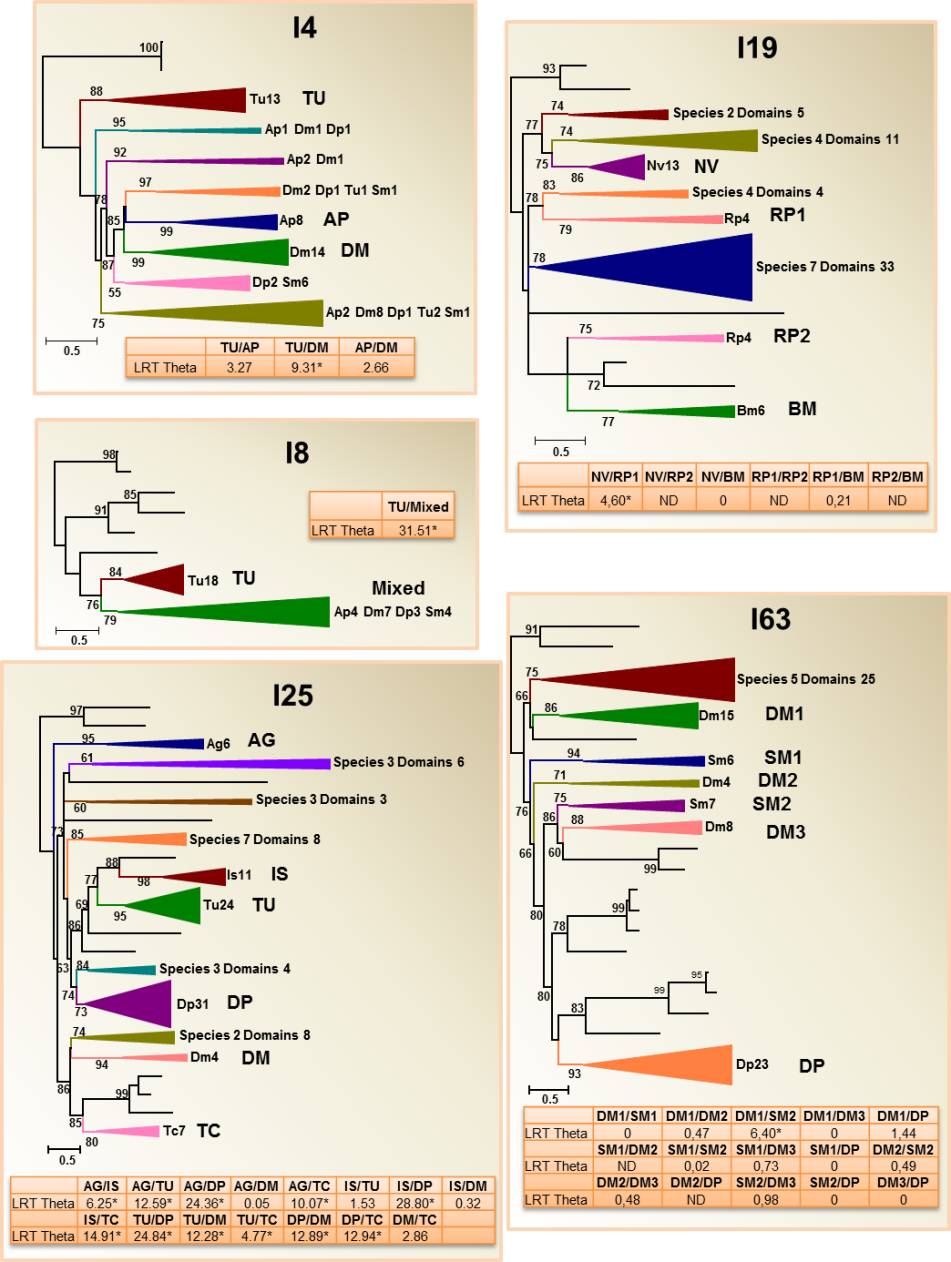
Phylogenetic distribution and divergence rates in peptidase inhibitory families with species-specific clades. Schematic PhyML phylogenetic trees using the inhibitory domains from the selected arthropod species are depicted. Coloured triangles show collapsed branches. Triangle size is proportional to the number of nodes (domains) included. The number of species with any domain in the collapsed branch is indicated. Numbers after species abbreviation indicate the number of inhibitory domains in the collapsed branch. aLRT values over 50 are shown. LRT Theta values for Type I functional divergence between clades formed by species-specific expansions are included. Dm, *Drosophila melanogaster*; Ag, *Anopheles gambiae*; Bm, *Bombyx mori*; Tc, *Tribolium castaneum*; Nv, *Nasonia vitripennis*; Rp, *Rhodnius prolixus*; Ap, *Acyrthosiphon pisum*; Dp, *Daphnia pulex*; Is, *Ixodes scapularis*; Tu, *Tetranychus urticae*; Sm, *Strigamia maritima*. Complete phylograms are shown in S3 Fig.

**Fig 5:**
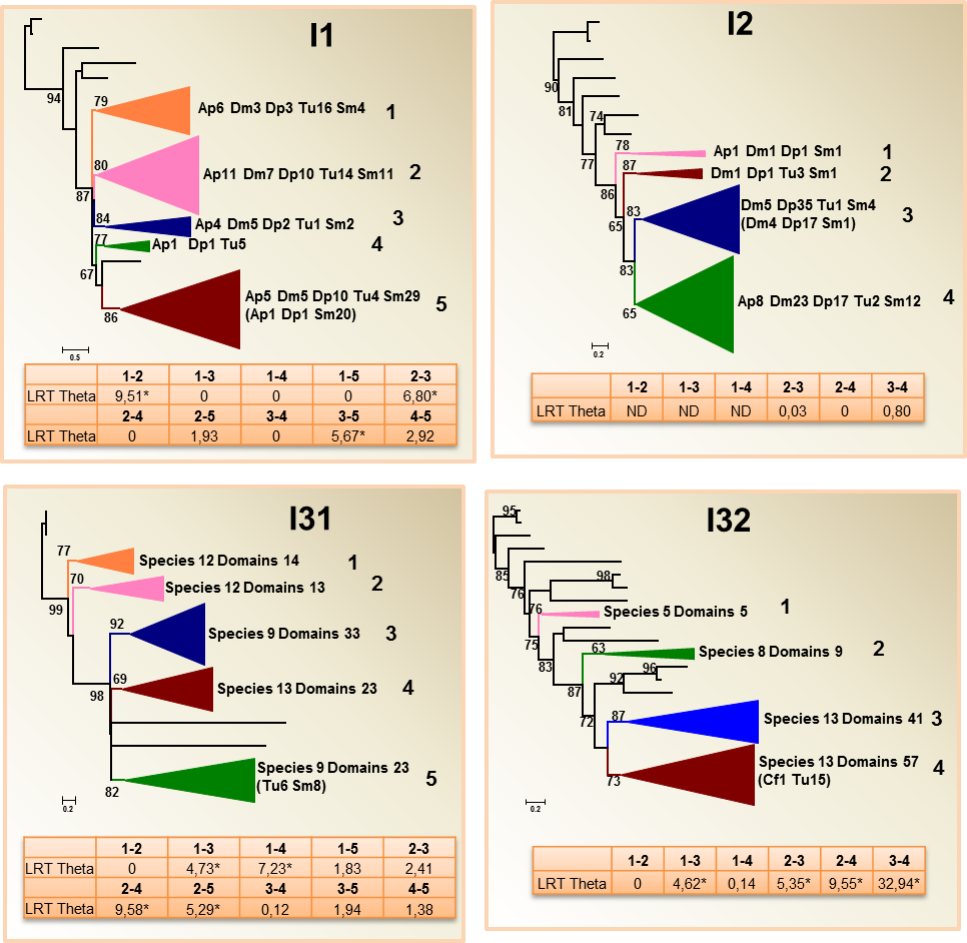
Phylogenetic distribution and divergence rates in peptidase inhibitory families with species-rich clades. Schematic PhyML phylogenetic trees using the inhibitory domains from the selected arthropod species are depicted. Coloured triangles show collapsed branches. Triangle size is proportional to the number of nodes (domains) included. The number of species with any domain in the collapsed branch is indicated. Numbers after species abbreviation indicate the number of inhibitory domains in the branch. In parentheses the number of inhibitory domains in a species-rich clade. aLRT values over 50 are shown. LRT Theta values for Type I functional divergence between numbered clades are included. Dm, *Drosophila melanogaster*; Cf, *Camponotus floridanus*; Ap, *Acyrthosiphon pisum*; Dp, *Daphnia pulex*; Tu, *Tetranychus urticae*; Sm, *Strigamia maritima*. Complete phylograms are shown in S3 Fig.

Phylogenetic trees were constructed to a better understanding of how the different families of peptidase inhibitors have evolved during the evolution and diversification of arthropods. For that, the amino acid sequences of the inhibitory domains were used. The complete set of domains for the fourteen selected species was chosen for most families, with the exception of families I1, I2, I4, I8 and I63. The number of domains for these families was too elevated to display an understandable phylogenetic tree. The domains of two phylogenetically unrelated insects, *D*. *melanogaster* and *A*. *pisum*, the crustacean *D*. *pulex*, the acarian *T*. *urticae* and the myriapod *S*. *maritima* were selected to construct the trees for these families. The complete phylograms for every family are shown in S4 Fig. Schematic phylograms highlighting the major evolutionary clades detected in each family are depicted in Figs 3–5.

The peptidase inhibitor families could be classified in three different groups according to their evolutionary patterns. The first group was formed by families without remarkable species-specific clades, the families I17, I21, I35, I39, I51, I87 and I93 (Fig 3). The complexity of the phylogenetic trees corresponding to these families was variable. Families I21 and I35 were single-copy families, with the whole members from the selected arthropods grouped in a single branch and mostly reflecting evolutionary relationships. The I17, I39 and I51 phylograms showed groups of homologous domains in two or three main branches comprising members of most species, with the exception of an I39 group that was formed only by hymenopteran and coleopteran domains. In these three families, a few species-specific duplications could be observed. The members of the families I87 and I93 were arranged in many groups. These groups included domains from most selected species, with the exceptions of two groups of the family I87, one of them formed by acari and myriapod domains and the other by insect domains.

The second group comprised families with species-specific clades that were clearly detected in the phylogenetic trees as well supported branches. These families were I4, I8, I19, I25 and I63 (Fig 4). The elevated number of domains in the I4, I8 and I63 families forced construction of phylograms using only the selected five species. In the I8 phylogram, a clade comprising 18 domains was detected in *T*. *urticae*. In the I4 family, species-specific clades in *T*. *urticae*, *A*. *pisum* and *D*. *melanogaster* were observed, whereas in the complex I63 family, three clades formed by *D*. *melanogaster* domains, two by *S*. *maritima* domains and one by *D*. *pulex* domains were detected. The I19 and I25 phylograms were constructed using the domains for all species. In the I19 family specific clades for *N*. *vitripennis*, *R*. *prolixus* and *B*. *mori* domains were found, and in the I25 family, specific clades including a high number of domains were detected for *D*. *pulex* and *T*. *urticae* as well as small clades formed by *A*. *gambiae*, *I*. *scapularis*, *D*. *melanogaster* and *T*. *castaneum* domains.

The third group was formed by families in which species-specific clades were detected but these expansions were found in phylogenetic sub-branches and includes any additional domain. It was the case of families I1, I2, I31 and I32 (Fig 5). The phylograms for the I1 and I2 families, with elevated number of members, were constructed using the domains for the five species selected. Although most domains were in branches including sequences of the five species, a subgroup formed by 20 domains of *S*. *maritima* together with single domains of *A*. *pisum* and *D*. *pulex* was detected. Likewise, in the I2 phylogram, a clade with 17 domains of *D*. *pulex* in a branch including *D*. *melanogaster* and *S*. *maritima* domains was found. For the I31 family, most branches were formed by domains of most species, but one subgroup was formed by several *T*. *urticae* and *S*. *maritima* domains. The I32 family included different branches with minor species-specific duplications, and a branch including a subgroup formed by 15 *T*. *urticae* domains and one *C*. *floridanus* domain.

### Evolutionary analysis for testing functional divergence

Functional divergence was inferred to further explore the evolutionary implications of the phylogenetic analyses and genomic features. Type-I functional divergence was measured in pairwise comparisons between the clusters observed in the phylogenetic trees. The likelihood-ratio test of the coefficient of divergence (LRT Theta) determined the prediction of functional divergence between pairwise clusters. In every phylogenetic tree, we focus on the clusters that were significantly divergent to the rest of tested clusters. In the families without remarkable species-specific clades, functional divergence was highly detected (Fig 3). In the I51 family the two main clusters, which includes most of the selected species, functionally diverged before speciation events. In the I87 and I93 families, all clusters were significantly divergent of any other cluster with the exception of clusters 1 and 2 of the I93 family. In the families with species-specific clades, functional divergence was not remarkable (Fig 4). When the species-specific clusters were pairwise compared, robust divergence was only detected between the *T*. *urticae* and the mixed clusters of the I8 family, and between the crustacean and arachnid clusters with the rest of arthropod clusters in the I25 family. Finally, in the families with species-specific clades that included any additional domain from a different species, divergence was only clearly apparent between the cluster 3, which includes domains from all species with the exception of *T*. *urticae*, and any other cluster (Fig 5).

## Discussion

Comparative genomics is a powerful tool to understand the origin and evolution of the genes present in extant species. The vast diversity of arthropod biology makes this group of organisms a model to compare the gene repertoires in phylogenetically related species. A previous review of the gene composition in the insects, which comprise a very diverse set of organisms, state the existence of a core of universally conserved genes together an array of lineage-specific and species-specific genes[39]. Peptidase inhibitors are a compendium of proteins grouped in different families with a common feature in plants and prokaryotes, their intrinsic variability[30, 40]. Based on this characteristic and in the diversity of evolutionary adaptations achieved for arthropods to exploit very different ecological niches, we expected a strong diversity in the repertoire of most peptidase inhibitor families, but also a restriction in copy number for those families performing universally conserved physiological functions.

New gene families typically originate either from a strong divergence in sequence of duplicate copies, from divergence in the sequence of horizontal transferred genes, or from genes originated *de novo* from non-coding sequences [41]. Fourteen of the thirty peptidase inhibitor families identified in arthropods were restricted to a monophyletic group or even to a specific species. Interestingly, most of these families are related to the anticoagulant properties of blood-feeding, hematophagous arthropods[42]. Members of the tick-specific I52, I53, I68, I72 and I74 families, dipteran-specific I64, I76 and I77 families and heteropteran-specific I59 family are implicated in the inhibition of peptidases related to blood coagulation and fibrinolysis. Conservation of these inhibitors could be a consequence of the adaptive value conferred for a successful blood-feeding strategy, crucial for their survival. Some of these families are most probably derived from duplications followed by strong sequence divergence, such as the I52, inhibitors of coagulation factor Xa, with a structure similar to I2 Kunitz[43], the I59 family, inhibitors of the complex factor IX/IXa, with a structure derived from the lipocalins[44], or the I68, inhibitors of carboxypeptidases M14, which present some similarity with the structure of I37 and I46 inhibitors[45]. The evolutionary origin of the other blood-feeding related clade-specific inhibitor families is obscure. A distinctive feature of them is the absence of cysteine residues in their amino acid sequences, placing them in the restricted group of cysteine-less peptidase inhibitors. A strong divergence from inhibitory proteins with disulphide bridges that lack the cysteine residues to favour a structural rearrangement leading to a change in their target peptidases[46], or a *de novo* origin, are the most probable hypothesis to explain the conservation of these families. The rest of the taxonomically restricted families have unique characteristics. The I11 family is widely represented in bacteria and protozoa. The single members found in a dipteran and a lepidopteran species could have been originated by horizontal transfer and their role in insects remains unknown. Members of the I44 and I71 families are scattered in different taxonomic groups and their function has to be determined yet. Members of the I83 family are distributed in several insect species and have been related to insect defence against fungal infections[47]. Finally, the I88 member has been found in a coleopteran species, has structural similarity with I1 Kazal inhibitors and could play a key role in protecting against bacterial infections[48].

Between the peptidase inhibitor families presented in most arthropod clades, a first partition may be done between families under “single-copy control” and under “multi-copy licence”[39]. The I21 and I35 families are examples of families usually under “single-copy control”. Whereas evolutionary constraints are reflected in the high amino acid conservation of I21 members, a more relaxed evolution was apparent for I35 members based in their lower amino acid conservation and their lack in several arthropod species. The members of single-copy families could be involved in conserved and tightly regulated physiological processes. The I21 protein from *D*. *melanogaster* is involved in hatching behaviour[49] and the I35 protein from *T*. *castaneum* in embryogenesis, morphogenesis and innate immunity[50], which supports a key role for the members of these families in developmental processes in arthropods.

The rest of peptidase inhibitor families are multi-copy families that are ubiquitously represented. In eukaryotes, expansions and contractions of multi-copy families are mostly associated to the stochastic birth-and-dead model. In this scenario, genomic drift involving random duplication and deletion of genes plays a key role. Genomic drift leads to the alteration in the composition of genes, which may serve as a driving force to create novelty that can be exploited by a species for adaptation to environmental perturbations[28, 41]. Genomic features, such as transposons or repetitive elements, have also been related to gene amplification[51]. The fact that no correlation has been detected in the selected arthropod species between the number of peptidase inhibitor sequences or domains with the total number of genes suggests that these genomic features are not relevant to the diversification of peptidase inhibitors.

Five multi-copy families have no species-specific clades in the phylogenetic trees suggesting that most of the observed clades come from a series of duplication events in the ancestral arthropod genome. Diverge analyses provide some cues to the evolution of these families. Whereas the extant repertoire of I17 and I39 members could likely be the result of a stochastic birth-and-dead evolution without a relevant functional divergence, ancestral duplications in I51, I87 and I93 families lead to functionally divergent proteins that were conserved in most arthropod clades. However, little information is known about the role of these inhibitors in arthropods and a panoramic vision of the function of these proteins cannot be showed. Members of I17 WAP, I39 alpha-2-macroglobulin and I51 IC carboxypeptidase Y inhibitor families have been related to the immune response and could be related to an ancient immune system[52–56]. On the other hand, members of the I87 family have been related to signalling and development in some insects[57, 58], and nothing is known about the role of I93 members in arthropods.

The rest of multi-copy families have strong variations in the number of protein sequences and/or in the number of inhibitory domains presented in each species. These variations are putatively associated with species/clade expansions and are the responsible of the large differences in the total number of sequences/domains among arthropod species/clades. Whereas four of these families (I1, I2, I31, and I32) did not show a species-specific distinctive branch in the phylogenetic tree, the other five families (I4, I8, I19, I25, and I63) did. From these findings, we could hypothesize that expansions lead either to a higher repertoire of inhibitors that share a common interspecific function or to a higher divergence between species/clades that could be translated in the appearance of new functions. Diverge analyses point to a higher repertoire without remarkable functional divergences. Robust functional divergence was only supported for the *T*. *urticae* clade of family I8 and the *D*. *pulex* and arachnid clades of family I25, which could also be a consequence of different rates of evolution on each side partly independent of functional constraints, or of further functional changes within the clusters. The trigger for expansions has been associated to the genetic bottleneck that occurs upon speciation[59]. Reduced population size allows conservation of duplicated genes by subfunctionalization. As family size increases, duplications are more probable leading to a higher family size. This increase in the gene content would permit the adaptation of a species to a specific environment, leading to preservation of fitness-benefit genes by natural selection[60]. However, the lack of an exhaustive analysis of the functions of most inhibitors in many arthropod species impedes to check the accuracy of this hypothesis. Protease inhibitors have not been associated with any physiological role in *S*. *maritima* or *D*. *pulex*, and limited functions have been associated with members of most families in other arthropods. In any case, members of the most studied peptidase inhibitor families are involved in a wide range of physiological processes. I1 Kazal proteins have been associated with blood feeding, reproduction, prevention of excessive autophagy, and protection from host and microbial peptidases[5]. I4 Serpins have a role in immunity, morphogenesis, and blood feeding[2, 42, 61–63]. I25 Cystatins have been associated with the regulation of morphogenesis and development, in the inhibition of heterologous cysteine peptidases during insect immune response and blood and seed feeding [64–68]. Finally, I2 Kunitz are involved in reproduction, formation of extracellular matrix, defence against pathogens and blood feeding [6]. Interestingly, a specific expansion of Kunitz proteins in the hard tick *Ixodes scapularis* is the only reported example of the appearance of a new function for putative peptidase inhibitory proteins. These lineage-specific expanded genes exhibit significantly higher expression during long-term blood feeding, have lost the ability to inhibit serine proteases, and have taken a new function of modulating ion channels[7]. In contrast, the less studied families seem to carry out more specific functions, I31 Thyropins in blood feeding[69], I32 Inhibitors of Apoptosis (IAP) in modulation of caspase-dependent and–independent functions[70], I8 Ascaris, TIL-type protease inhibitors, in insect resistance to pathogenic microorganisms[69, 71], I19 Pacifastin, in the regulation of crustacean immune response processes[72], and the putative inhibitors of the I63 C-type lectins family, in the inmune system[73]. However, the absence of a more complete analysis of these families together the wide tissue and time expression of their members, as found for I19 Pacifastin proteins[72] suggest that with a more profound investigation in these peptidase inhibitory families a wide involvement in more physiological processes will be found.

## Conclusions

Taking together our findings, the strong diversity in the repertoire of peptidase inhibitors in arthropods seems to be the result of several evolutionary forces. Peptidase inhibitors have to regulate endogenous peptidases, which are involved in most physiological process in any arthropod, and have to modulate the action of exogenous peptidases present in the organisms that interact with it. Genomic drift in distant phylogenetic clades leads to passive expansions that may be used by natural selection to fix particular adaptations. Besides, some functional divergence events contribute to reach diversity. Overall, conservation and divergence of duplicated genes and the potential recruitment as peptidase inhibitors of proteins from other families are used by arthropods to control the broad repertoire of target peptidases. The final peptidase inhibitor repertoire would contribute to the vast diversity of arthropod biology and to the adaptations achieved for arthropods to exploit very different ecological niches.

## Supporting information

S1 Fig Amino acid sequences of the domains belonging to the different peptidase inhibitor families. (DOCX)

S2 Fig Comparison of the amino acid sequences of the domains belonging to the different peptidase inhibitor families. (DOCX)

S3 Fig Information about gene models corresponding to the peptidase inhibitors used in this study. (DOCX)

S4 Fig Phylograms of the peptidase inhibitor families. (PPT)

S1 Table Genome versions and Blast pages. (DOCX)
