# Supplementary material for: Insights into the molecular evolution of peptidase inhibitors in arthropods
Source: PLoS One. 2017 Nov 6;12(11):e0187643. doi: 10.1371/journal.pone.0187643 (PMC5673224; doi:10.1371/journal.pone.0187643)
Supplement: S1 Fig — (DOCX) [file pone.0187643.s001.docx]

**S1 Fig.** Amino acid sequences of the domains belonging to the different peptidase inhibitor families. Dm, *Drosophila melanogaster*; Ag, *Anopheles gambiae*; Bm, *Bombyx mori*; Tc, *Tribolium castaneum*; Cf, *Camponotus floridanus*; Am, *Apis mellifera*; Nv, *Nasonia vitripennis*; Rp, *Rhodnius prolixus*; Ap, *Acyrthosiphon pisum*; Ph, *Pediculus humanus*; Dp, *Daphnia pulex*; Is, *Ixodes scapularis*; Tu, *Tetranychus urticae*; Sm, *Strigamia maritima*.

**A. I1 Kazal.**

>ApKaz-1a

CPRICPQGSSEEPVCGSDGIIYPNECELKKKTCGKGIVVAESSTQC

>ApKaz-1b

CEHRCGKEQDAVCGTDGRTYLNRCMMQVEICRVGTSMSHLGPC

>ApKaz-1c

CPVNCDQAPMDGPICGSDGNVYKNTCQMKLFTCGQGVVRTSKKHC

>ApKaz-1d

CRESCWRVSKPTCGSDGNIYSNACRMKSKNCGKHVFEVPMAFC

>ApKaz-1e

CPVNCDKEKDRLTCGSDGNVYRSECEMKMLNCGQQTKKKVTKVDLEKC

>ApKaz-1f

CNKGQCPDDADPICGNDAQNYKNQCQLDQATCLRGIQMAHLGKCSS

>ApKaz-1g

KCPQSCENEREEPVCASDGNVYRSECDLKMNTCGQKVVAVPPHHCPT

>ApKaz-1h

CHQQCDKTKEFVCGSDNKLYRNECEMKRENCGKHVYVVPMKRCL

>ApKaz-1i

GCQRVCPTLYDPICGTDLKTYSNDCFLEMENCRSRSLVSKQYHGVCGQ

>ApKaz-2a

CQGDDSGDGAGGPVCGNDWRDYPSACHVRKASCASGRNIAVKYRGLCDPCD

>ApKaz-2b

CSEFCGEDFIPVCGSDGRTYTNECFLRRQACRMMPGLRIVFHGPCD

>ApKaz-2c

CPEPTLCETSVRPVCGTDGYTYESKCLLERIGCSKRSGVTVAYDGHCGDA

>ApKaz-2d

CPLCSEHHEPVCGTDGNTYSNECKLKYHSCQQKQIIGVSHNGTCNDCTK

>ApKaz-2e

CPTFKCANASGKVCGTDGITYTDLCHLQNASCATRKKIFAAYAGECGS

>ApKaz-2f

CPETCVDTKSKEPVCGSDLITYNSECELLQRACVKNGTHNLTILFYGDCK

>ApKaz-2g

CIFNCSATDSSPVCASDVRTYNSLCLMKMEGCQRQQELRLRPMELCQGMEV

>ApKaz-2h

CRATCAEDAIRTTLVCGSDGQTYSSECQLKLYACRYQKDIVVKSHTSCKDE

>ApKaz-3a

CSPNCKQHDGLKVKGPVCGTDGVSYKSHCRLKKRSCRTKDQSLLVDYHGLCQS

>ApKaz-3b

CVTVCPPGMSSSGQVCGSDGRTYQSACHLREAACHAGNAIPIAYKGLCKE

>ApKaz-3c

CNMSCPKPVPSEKLKQTGGKVCGSNDKTYHSWCQMFMDACATGVVIETKASGPCPDG

>ApKaz-4a

CNCAPHHVPVCGSNGNTYPNSCLAKCAGLSDVDLKFGTCWN

>ApKaz-4b

CVSEKTSCEDAPKGVVCDTDGGEHSNLCHLLRSGKTLAYSGPCLV

>ApKaz-4c

CLVGCNSTGRVCGVDGNTYPSECAAFAESTSVDYSGPCAS

>ApKaz-5

QYRCACKETDPVCGSDNRTYSNMCQLNEAAAELGHNATQLRIQYRGPCQS

>ApKaz-6

CKSECPNDYNPICGSDGAKVNLSFGNKCVMEKYNCEHNAKLTVKMETECPN

>ApKaz-7

CNPCPVMKPTFLCGSDNRTYSSLCRLDYHNCLHTTNIKVNCKGFCPCK

>ApKaz-8

CVRKCGPAKRRVCGTDGRLYDNRCELHKSGCLSGVDIQIDHSLKCFIP

>ApKaz-9

CKGRVERCERKKAATRRPVCGTDNISYPSRCALLRVRCFNDSLLRVKHRGRCKEKQPCW

>DmKaz-1a

CPRSCPPSITVGAEPVCGSDGLIYANICELRKKTCSRSGVSLIKDVRDGC

>DmKaz-1b

DCKHRCSTEKDPVCGTDGRTYLNRCMLRVQSCRVGLAAVKLSHVGPC

>DmKaz-1c

ESCPVDCNSAPKDGPVCSSDGNVYNSTCEMKLKTCGQGVVKTSRKHC

>DmKaz-1d

MCRESCWRVARPTCGSDGRLYASPCKMRSSNCGKHVFEVPLSYCM

>DmKaz-1e

CPTECPKSDTDSSSQYVCGSDGNIYSSLCELKMLNCGPQRKSIQKVSMDKC

>DmKaz-1f

CKQLPPCKDFNSLFGSIFSSKRNDKLCGTDAKTYNNECELAHATCLRGVNLAHIGPCT

>DmKaz-1g

CGDACTRADLEQQPVCGSDGNTFASMCEFKRRTCDLRVVPVSLKNCAL

>DmKaz-1h

CESDCDAQPPSFVCGSDNNLYKSECHMRKENCGKHVFVVPLKRCLA

>DmKaz-1i

CARICPREFEPVCGSDNKTYLNDCFLEIENCRANQTVNVNYYGACG

>DmKaz-2a

CRIECPWDNLDVDSSGYDERQAVCGVDGKTYRSACDINRMICKIGRSIAVAYPGPC

>DmKaz-2b

CRYKCPRKQQRPVHKICGYNNQTYNSWCEMHKHSCESRYFIGVKSQGSC

>DmKaz-3

CAAKQGECDDNEGPVCGTDGQTYPTRCHLLRAQCGGHQVSLKYSGSCNAC

>DmKaz-4

VFQYSCPCPRNYDPVCGSDSVTYSNQCVLDCLIKEGRSITVEKKGRC

>DmKaz-5a

CEFECSSRYQPVCGISSKSGERKTFRSRCEMLRTACISRSEWMVHRWGVCP

>DmKaz-5c

CDKTCPTVYQPICATRNGINHTIVNECYLERVRCKDPKSIWKLSHKGECA

>DmKaz-6

AQKSRCPCPKISEPVCGSDNITYPHLCFLICKAWHENVNFVKKGRC

>DmKaz-7a

TACPTFCPSIYKPVCGTDGQNFKEFASTCNLLSHNCRRERNSVQAYAATDAAWC

>DmKaz-7b

KECFKPCSMIYQPVCITNGKYRAELANSCLLENFNCALQVSGAQPAELFRLLREEKC

>DmKaz-8

PICPCPRNYEPVCGSNLVTYPNRCEFDCVRRNVERQGRSMGLLRDGTC

>DmKaz-9

CTKECPDIYDPVCAQIFQEEYLTFSNECEMRNYICTNERPYSFISVGECVE

>DmKaz-10

TRPLCPCPRIYFPVCGSDHVTYTNSCELKCAAQIKLIYVVKAGRC

>DmKaz-11

CDYSCPEKDPSVCATNGQCILKFESRCAMSAYNCRNPQKMFKPVEDHRCTQ

>DmKaz-12

LEVCPDNCQDQYNPVCGKYKDTRRNFRSECELQLVKCRTGHPWRKQHDGPCE

>SmKaz-1a

CPRQCREIGSTPVCGSDGVIYASECEMKRRNCGRPTVVALPSLKRC

>SmKaz-1b

CTAFCYGHHDPVCGSDAVTYDNECKWAQQACATGIRLAHPGTCHN

>SmKaz-1c

CPNNCPPVRGFLERPVCASNGNVYRSECQLRLQTCGQKVTTASYIYC

>SmKaz-1d

CRPRLEKCNKGTCGAGFNPICGSDGRTYNNDCAFRVAACQKGINRAHEGIC

>SmKaz-1e

CPKECPIPSPGDGPVCGSDGNVYPSACEMKKRTCGRRVVPVKKSFC

>SmKaz-1f

CDTNCPIEAGLKPVCGSDATLYMSECHMFSKNCGKHIFEVPLAYC

>SmKaz-1g

CKEYCSPIPDPVCGSDGKAYSNECFLGLETCRTRGKVRMDSTWKCG

>SmKaz-2a

CTFGQDICPLIYAPVCGSDGNTYSNKCFFGFAQKTKPTLTIKHSGICTKSK

>SmKaz-2b

CPESSKHGICTLEYAPVCASNDVTYPNKCSMCADMYMNNKKEGKDVILTVKHYGVC

>SmKaz-2c

CPETSKHGICTLEYAPVCASNGETYATKCNMCAHKYKQGDVKLRVVHPGRCG

>SmKaz-2d

CLPRDNTGACTREYMPICGSNGKTYPNRCTFCYAKNQEETSGKSLSMEYTGEC

>SmKaz-2e

CPEMSKKGACPRNYRPVCGSDGKTYGNLCMLCVEKYNQHRQDSGMALRLEHEGACK

>SmKaz-2f

CRPEWKTGICTREYNPICGSDGVTYGNPCTFCYSMFEKQAKGNDIRVKREGQCL

>SmKaz-2g

CPKSLANGICTLEYSPVCGTDGIVYSNKCMFCYAQQKKLKEGVEMYFDHMGMCY

>SmKaz-2h

CTASRPCSFGKTPVCASDGKTYSNICVFFAKMDLSLYMVHSGSCTIYDSCS

>SmKaz-2i

CSENNQHIICTEEYNPVCASNGQTYGNKCEMCADSYKKTKTGEDDVDLKVIYAGKCGTFDYCL

>SmKaz-2j

CLHCSEMSKDGICSREFNPFCGSDGRTYGNLCMLCAEIYRQKQAGTYEKLSFLHTGECP

>SmKaz-2k

CPGKNENGICTTEYSPVCASNDQTYSNKCEMCAEVYKQRKEDEDFSLKVIRPGKCESFDYC

>SmKaz-2l

CASCPVQSHSRDVSHCADQEDNPICGSTGKTYNNTCLFQVATLIDTSLYIAHPGKCTIYDL

>SmKaz-2m

CPEISDYYKCNDAYHPVCASNAITYGNKCKMCSDLYRQRKEIDISIHFQVIHTGICG

>SmKaz-2n

CLESDMKEECSKDYNPVCASNGITYASRCVFCFNKHHPIHQDNEIVLEHVGKCLY

>SmKaz-2o

CPKLSKRGICPRGGNPVCSSDDTTFENLCMLCAEIYRQKKRGTYINLAIQHNGECQI

>SmKaz-2p

CSSSVCPSIWNPLCGSDGKTYANDCEMKIARCKNPNLTENHAGSCEFQ

>SmKaz-2q

CLKEDKLEMCTDEYDPQCGSDGTTYPNHCMLCAAMYNGSDNLKVINRGPCKKVE

>SmKaz-2r

CDEQILKIECTQDEFPLCGTNGITYGNPCKFCKGFYALKKAGTELNVKNLGKCPKHEDC

>SmKaz-2s

CPISPINACPLNYAPVCASDDNTYPNLCAMCAAMYKEHKPLRVVYEGVCQA

>SmKaz-2t

CLPFETMGLCNKQYSPVCGTNGVTYGNKCALCYAMSQRHDLKLDHPGVCI

>SmKaz-2u

CDMNIKDCSKESHPVCATDGTTVPNDCVFCMYKSDREKDGVKVGLAHRGEC

>SmKaz-2v

CTEMSKFGACPYTYFPLCATDGKSYPNLCVMCAEMYKQRKNGHFDFVLRKLHEGIC

>SmKaz-2w

CAGVGKNGVQVCSEEYSPYCGSDHATYKNKCQFCREMYEREGKGGLLSVNMEGECPE

>SmKaz-2x

CSSSDLNGACPDNYMPVCGSDGVTYANNCERCAEMSRRHQAGENIFIYNKYKGKCP

>SmKaz-3a

CPTQCSLDKIDPVCGSDGKLYYTQCHLEKETCGQDVSKVSWSDC

>SmKaz-3b

CKADCEKGFCTMEYSPRCGSNGNKYGNKCTLLWASCGLPEPIK

>SmKaz-3c

CPDQCPFGLIDPVCGSNGKLYYTECYLEKATCGQGVTKVPWSEC

>SmKaz-3d

CKADCDKSICTLEYSPRCGANGSKYGNKCALLWASCGL

>SmKaz-4a

CQTNCPSVFSPVCGTDGRMYSNRCQLNVANCQSNGRIRLDPTRACCRYNNC

>SmKaz-4b

CTVKRCPDVCYQLFDPVCDTNGKQYGNKCFFRIAACRDKNIRYDRKGAC

>SmKaz-4c

CSSICIALYKPVCDTNGNRYENDCRLSIAKCHDKRVVYDSTGACLD ^^

>SmKaz-5a

CSRACPDSKAKKYVCGVDGVSYTSFCHLRQAACQKGRAIPVAYPGKC

>SmKaz-5b

CHFNCRAVKFHLQGPVCATNNVTYASWCHMMQASCSLRTALELRSNAKCEE

>SmKaz-6a

CAEAAATCTGPRPMKPTCGSDGMLYYNECHMKDEVYGIVPYPVYPVAWSEC

>SmKaz-6b

CENINCLQLCTREYDPVCGSDGEIFGNPCSFLWEHACG

>SmKaz-7

CVCQRYCKKHKKMVCGSDGNLYPNHCELHRASCVTGTSITIDRKNSC

>SmKaz-8

CQETCSAVFAPVCGSDDVTYSSECQLRMASCNQQKRILVKYKGSCE

>SmKaz-9

CPDKCPSYGDHRGSLPVCATDGKDYPNVCELRRAACQNMKDVEERYQGKCGE

>SmKaz-10

CDENCSKEINRVCGTDGVTYNNPCLLKKANCESNGKIQEDHTGASCK

>TuKaz-1a

CTHQCNPNVHHYVCGNDNVTYPSSCHLQLSSCKLQQTIYVKHEGNCH

>TuKaz-1b

CLQCESIYKPICGSNRITYDSVCHLLRDSCLKKVNLTVSYSGPCE

>TuKaz-1c

CRQCPDENEPVCGSNGVSYANECELHKASCQDQKAIYISHKGPCR

>TuKaz-1d

CPQVCLRLDAPICGSNGVTYDNECELRVRSCLAQTDITIAHLGPCD

>TuKaz-1e

CPTSCSDPVYEPICANDGNTYPNECEMRKNACLNDMDLRSLFYGQCE

>TuKaz-1f

CKFNCSTMIRQSKGKTETVCGSDGRYYENLCSLKEESCRRQIEIKSVDSDQCN

>TuKaz-2a

CDTFQCDSTPFDPICANDNKTYSNQCQLNFTSCNLQTPLTVQYKGKCE

>TuKaz-2b

CPSCSHHYEPVCASDGLFYTNECVLRRESCKENLNLRIVHSNECG

>TuKaz-2c

CPKNCSQEKDPVCGSDGLNYLNECQLRLTSCQQRQYITVTSKVQCD

>TuKaz-2d

CPKDCPSIYEPVCSTDGYTYANECQMRLTACQRKLELSVTFYGECQ

>TuKaz-2e

CSYSCPVRDESRDEFVCGSDGRLYENECKLQEEACRRQQEVALESRDKCD

>TuKaz-2f

CNIDCSPEEKVSDKVCSSDGTIFPNECQLKIYSCRMQRPLTTLYKGPCK

>TuKaz-3

CLDSCPSTYEPVCGSNGETFVNECKLRMESCKRAINLFVRYPSACD

>TuKaz-4

CPITKKCPVHKHKICGSDGKLYDSHCHLRKISCEKGIQLRPVHPDQCD

>TuKaz-5a

CQEDCPLDGGISVCGTDGLTYQNECLLNLNACKKNVAILVAYHDTCD

>TuKaz-5b

ICPTDCPTSFEPVCSSEGTTYTNECHLRHSMCSTGITLTVAYYGECQ

>TuKaz-5c

CSYSCPSQESDEDPVCGSDGRLYENECKLQEEACRRQQDIKPENRSMC

>TuKaz-5d

CDMKCQQDVTRKRLAGSGSERVCASDGTTYPSECGLHLYACRMQKNLTVLYQGECT

>TuKaz-6a

CKTCSSHINPVLLCATDNTTYYSECDIERVSCLKNEPIAIKHHGPCT

>TuKaz-6b

CPYCEDSGPSVCGSDGITYSSQCSLSTACCTEKRYITVRYDGPCQ

>TuKaz-6c

CPKVCIRDDRPVCGSNGKTYESECELQMASCHLQINLTVAHHGSCA

>TuKaz-6d

CNFNCDSEPEESVCADDGLTYANQCSLRRSSCLKGRKLVSLFFGTCD

>TuKaz-6e

CDINCQNNSTDVSNSSMDYSIVCGSDSNTYQSECQMLRASCLHQTDIVILHKGPC

>TuKaz-7a

CPEKCYTYGDSVGSRPVCGSDGRDYPNECELRRKACVSNKEISVKFQGKCD

>TuKaz-7b

CRCNAVEEEIIIICGSDGKTYTNECILRVEACKARKSLRILYNGECG

>TuKaz-7c

CPPMCPQIMNPVCGSDGKTYDSECELRREACLLHKNVTLVYKGLCG

>TuKaz-7d

CQSCTEEYKPVCGSDGISYSNECKLRRESCEQQKSLKVISQGYCN

>TuKaz-7e

CIKNCSHIKMPICGSDGVSYANECELRLASCTKKEYLTIASKGPCD

>TuKaz-7f

CPSECPQTYEPVCSTDGLTYINECQMRVNACQKNVELGISFYGECQ

>TuKaz-7g

CSYQCPTTSGPDDSVCGSDGRLYENECKLQEEACRRQQEIQPALNRLC

>TuKaz-7h

CAMRCPEVTLGPGHLIRRTSQVICASDGNTYLSECQMKFFACRMQRKINLVHHGQCRNT

>TuKaz-8a

CLSTCTVDVFAPVCGSDNITYSSECQLKLASCNQRTTLFVKHPGECQV

>TuKaz-8b

CPEKCLIYGDTKSSGSVCGSNGMNYPNSCELKRWSCIHSADVTIKYHGKCD

>TuKaz-8c

CSSLCNDNFKPVCASDGKTYANECFLRVESCRSRHNLYIIHQGDCN

>TuKaz-8d

CPSECPPILKPICGNDGVTYESVCDLQRKSCLLNQPVKVKHYGSCS

>TuKaz-8e

CPTSCTEDYNPVCGTDGISYVNPCKLARESCEKRRSIEVAYKGLCG

>TuKaz-8f

CSESCVPVDEPVCGTDGKTYPNECSLRVTSCKQQTYIAVESKGACN

>TuKaz-8g

CREDCPEENESVCASDGVTYRNQCEMEKISCQTNIELSVRFYGKCS

>TuKaz-8h

CLFNCDETNSSEIICANDTYFYPNECSMMEKSCHNRKPLTIVSPEIC

>TuKaz-8i

CQYNCDSSDLLPVCGSDNNTYGSTCQLNFVACRNQKILTVIKIGSCN

>DpKaz-1a

CPRFCPKENKPVCGSDGVIYTNECDLYRRNCGLEVETVDDKMC

>DpKaz-1b

CGNKCSLDRDLVCGSDGRTYLNICVLKVETCKRGIRLSHVGPC

>DpKaz-1c

PCPQQCSSNDKDGPICASNGNVYKSICEMKRHTCGQGVVKTSEKFCQ

>DpKaz-1d

HCKEICWKASKAVCGSDGHIYASSCQMKVKNCGRHVFEIPISNC

>DpKaz-1e

CPAECDSAEPQEVCGSDGNIYGSPCELRMLNCGPNSDKVIAVDWLRCS

>DpKaz-1f

CQDDLENPDYVCGNDGRTYPSPCHLRMASCTRGTELAHVGACMKITPDNS

>DpKaz-1g

CPESCPDQRESDQPVCGSDGNVYRTQCEMKKQTCGQHVTATESCN

>DpKaz-1h

SCNSQCDRKVQSVCGSDGKIYRNLCEMRGKNCGKYVYEVPMARC

>DpKaz-1i

CNRICPSEYDPVCGTDRKTYSNECFLQLENCRSRSLVIKKYHGKCG

>DpKaz-2a

CNVFCTFEFNPICGTDGQTYSNPCSLNAKNQCDGTAISKAYNGRC

>DpKaz-2b

CQIFCTLEYMPVCGTDGKTYPNICHLNAYNKCHGTHIEKAHDGPC

>DpKaz-2c

CLPYEFCVPDPHPPVCGTDGKTYRNACQLARSNACLHPPEVKVCHKGPCKF

>DpKaz-2d

CRMPCTREYNPVCGTDGRTYANPCVLKAKNTCDGTRVHKAYDGVC

>DpKaz-2e

CNGPGYSFNPICGSNGKTYDNECELNGRNKCDGTKIEKAHDGEC

>DpKaz-3a

DPSCNCKKNYAPVCGTDGKTYSNECVLSVETKVLLKAGIGRKAICV

>DpKaz-3b

CVCTLQYEPVCATDGKTYGNACALECSKKQTCGQHVIPVALHHC

>DpKaz-3c

ECGCQFKLAPVCGTDGKTYDNENCMEVEVTQCGTVIDSPIRKAHDGECK

>DpKaz-4a

CKGPCPPIHRPVCGSDQLTYSSNCELERESCLQKRSIKLLYEGVC

>DpKaz-4b

CPLCGGEWDPVCGTDGVTYTNPCRLRYESCRHNKSLSIVYKGLCNLIDDT

>DpKaz-4c

CPKACAKMAITEVCGTDGITYRNECELKQAACRNQQFIVVASKGDCD

>DpKaz-4d

CPMDCPVASSAEQTVCGSDGVSYGSECDLRLAACRKQLNVVMAYEGPCS

>DpKaz-5a

CSSYGDSVGSRPICGVDGKDYANMCELHKSSCLANRMIAVKFQGSCDPCA

>DpKaz-5b

CGDTCPSDFQPVCGSDGRSYSSQCHLQQEACRSQRHLRILYKGLCESGTYGC

>DpKaz-6a

CEIGCLEIYEPLCGTDGKTYPNKCTLFVENNCDDTSPNRIRKAYDGEC

>DpKaz-6b

CIKEALKDCISIQIIICGTDGIVYGNPCQLKAKNKCDGTSVGEAPASHCQQ

>DpKaz-7

CLATCPDHFVPVCGSNNQSYDNFCLMHRDACLTGVHISLKKKGYC

**B. I2 Kunitz-A.**

>ApKun-1a

GCQSKPILPGDRCKEPKDRGSCSDFTVKWFFDTEYGGCSRFWYGGCNGNNNRFKTQEECKDICVEP

>ApKun-1b

DVCYLPKSVGPCEGYYPTWYYDQDRKQCAQFVYGGCLGNNNKFQTREECEHLCVIPDT

>ApKun-1c

PCEQPLTPGPCKGNFSRWYYDKSTRSCSQFNYGGCKGSQNNFLNKESCNHKCINPL

>ApKun-1d

EECLMTVARGDKNCDKKIPRWYFDNNENACKPFYYTGCGANANNYETQESCEKKCPSKR

>ApKun-1e

DTCKLPALVGECHDYVNRWYFNSLDGRCRQFYYGGCGGNENNFETEYNCENKCIDSGRIT

>ApKun-1f

KCFLNQDRGNCSNMSSKYFYDRQDGVCKPFMYGGCGGNDNRFESKQECERQCF

>ApKun-1g

EAQDLCQLPKVEGPCRGDFRQWYYDKNSDRCFQFQYGGCRGNTNRFNDRQTCETRCVQN

>ApKun-1h

SDVCLIPLDPGPCLQTVDMWYFKTSSRRCESFSYSGCEGNANKFQSVEECERICHPYIDPNA

>ApKun-2

SSLAKEICSMEKNEGACNVNAVRYYYDKTFKTCKQFKYGGCRGNENNFKSAKECYKVCHGVV

>ApKun-3

ILICMPVTANLTRDRCFLPPDEGNCGNRLTLRVKYYFDSANDDCSEFIYFGCGGNNNRFDTFEECENVCIFYGF

>ApKun-4

TVFDVKALENFKERCEIPIMRGPCQNWIHKWYYDSILHQCRTYISGICKSENIFDSEAECLYYCVGAK

>DmKun-1

ANTGRNTHPEQFCLMPARKGVCRALIPRWRYDPEQKKCVEFKFGGCDGNENNFASYKDCMSTCEGM

>DmKun-2

GSSSGNGAVVSCKELNNFNCYVGRNEGNFCSRKDQTKVVTRWYFDKGVCKPFNYKGCNGNRNRFCSQESCDARCGD

>DmKun-3

HERCSFIANPGPCKGNFEMFAYDMDNNVCVEFIYGGCGGNPNRFQTKKECILLCNALADEDEYLIVYTDKNEQQITDGTMSEEG

>DmKun-4

CYDELTDSPCRDNDVANFWYYDHVSDQCAIYWSDRCDTNRNKFKSKEECEETCRLPRHK

>DmKun-5a

PQKACGLPKETGTCNNYSVKYYFDTSYGGCARFWYGGCDGNDNRFESEAECKDTCQDYTG

>DmKun-5b

KHVCLLPKSAGPCTGFTKKWYFDVDRNRCEEFQYGGCYGTNNRFDSLEQCQGTCAAS

>DmKun-5c

TCEQPVESGPCAGNFERWYYDNETDICRPFTYGGCKGNKNNYPTEHACNYNCRQP

>DmKun-5d

KDRCALPKQTGDCSEKLAKWHFSESEKRCVPFYYSGCGGNKNNFPTLESCEDHCPRQ

>DmKun-5e

KDICEIPAEVGECANYVTSWYYDTQDQACRQFYYGGCGGNENRFPTEESCLARCDRKP

>DmKun-5f

SRQDVCDEEPAPGECSTWVLKWHFDRKIGACRQFYYGNCGGNGNRFETENDCQQRCLSQEP

>DmKun-5g

VAQCSQPADPGQCDKWALHWNYNETEGRCQSFYYGGCGGNDNRFATEEECSARCSVNIDI

>DmKun-5h

KCFLAFEPGNCYNNVTRWFYNSAEGLCDEFVYTGCGGNANNYATEEECQNECNDAQ

>DmKun-5i

TTCALPPVRGRCSDLSRRWYFDERSGECHEFEFTGCRGNRNNFVSQSDCLNFCIGEPVVE

>DmKun-5j

YSVCAEPPEAGECDNRTTAWFYDSENMACTAFTYTGCGGNGNRFETRDQCERQCGEF

>DmKun-5k

DVCNEPVTTGPCTDWQTKYYFNTASQACEPFTYGGCDGTGNRFSDLFECQTVCLAGRE

>DmKun-5l

GSAKEICLLPVATGRCNGPSVHERRWYYDDEAGNCVSFIYAGCSGNQNNFRSFEACTNQCRPEPNK

>DmKun-6

TKMGSYKVRQEKCLFIPSYGRCKKHIAVYGYNIITNRCSEFTYSGCGGNPNRFMTDSQCRNTCYVVPARKTVSEPDYYADDGVTEPMQVDEEDDY

>DmKun-7

KQPKCWYVANPGPCDDFVKVWGYDYLTNRCIFFYYGGCGGNPNRFYTKEECLKTCRVYRPPNRKKREENLDEEEEEEFEEDIDN

>DmKun-8

KNEICGLPAAANGNCLALFSRWSYDAQYNVCFNFIYGGCQGNENSFESQEECINKCVE

>DmKun-9

KRRCLQPLDVGKGKAYLRNWFYNSTSQRCQRFIFYGGASNGNNFNTQARCHKICLAQVSLPINFNSTAA

>DmKun-10

ADEICQLTPEANGFGKIMSCAHYSNWFSYHSDKNECLEFSYGGCGGNENRFQTKAICEDLCKNKVEQL

>DmKun-11

YEREQYNIRKKICLQSSEYGKCKGRRKLWFYNPKKSKCQVFIYSNCGGNGNLFYTKESCVEFCGKYDWKKVRKTGLRRSADYRRKDGN

>DmKun-12

KAFCYLPYEFGKCGGHRIMWAFSNKEQECVPFVFSNCGGNENRFYTKENCEKACATIQSRFVLAN

>DmKun-13

YEKCAGPGDPGPCKQYIYKWRYEPTTNECTNFIWGGCEGNPQNRFGTEAECLFHCIGGPHT

>DmKun-14

ICVQAPDPGPCRGTYMRYAYDPQNQHCYSFTYGGCRGNRNNFLTENDCLNTCNVLRSPYSSR

>DmKun-15

SLGLPSLENQTHEQIEQIIACRQPKAPGLCRGHQLRYAYNKKTGNCESFIYTGCASTENNFLTFEECRRDCMQRLRY

>DmKun-16

WVHAKPEMCQQPSSMVGMAQDGAACMAFMPAWTYDASKNACTEFIFGGCGGNSNQFSTKSECEKACKD

>DmKun-17

KNAICGLPHSLNGDGRISCEAYIPSWSYDADRNECVKFIYGGCGGNNNRFNSREICEDKCLQ

>DmKun-18

KDPICGLPAGIDGNGLIKCAAFIPSFSYHPETNSCEKFIYGGCGGNENRFGTQELCEQKCKE

>DmKun-19

SELTVPEDCHQPKETGRCFALFYRYAYNVDTQSCEEFVYGGCAGNKNNFESKEQCEQACLVKSAVSSTDSTTEQNSEVATETSTSS

>DmKun-20

IKPQRLVPDPKCLQPLDVGPCRMSLERFYYNKDSKACETFKYGGCRGNDNRWGFRQTCEEACIPKK

>DmKun-21

KRVKLCLQPMISGRCFGYVESYAYNPIKRHCEPFIYGGCGGNDNRFSTKAECEFNCRDI

>TuKun-1

TDICLAPLRSEPCLTGMLAEPIRRWYYDNQSQLCLPFNFTGCEDISNNFKTKQDCEILCRQML

>TuKun-2

REHCKQAHYYGYCEQNVTSYHFDTATNQCLPFTYSGCGGSINTFDTLKQCTDYCT

>TuKun-3

VCMTPKDVGPCRGYYPRWYYDGDKAMCLQFIYGGCRGNRNNFERYTDCSKMCEVLLRDFVKA

>TuKun-4a

AVCKLAPQPGPCYSYNPRYFFNDETKTCELFIYGGCQGNGNNFETQKDCMSLCSLTS

>TuKun-4b

KREDICFQPFAEGTCKENLIRYYYDPEVNKCIKFIFKGCFQYDNNFEKLEDCESLCVI

>TuKun-5

NHCRQTHYYGYCQQNLTYYHFDTKTNQCLPFTFSGCGGTVNTFATLKACTDYCTA

>DpKun-1a

EICALDKDRGSCRNFTVGWFFDMEYGGCSRFWYGGCDGNDNRFPTQDDCKAHCVEPIG

>DpKun-1b

IEACSLPRVAGPCEGNYPSWYHDTTTGSCKQFRYGGCLGNTNRFSTREDCNQQCIAPK

>DpKun-1c

DKCEKPQDAGGCQGTFQRWSYDKTSMTCQEFNWGGCQGNENNFLSERECHLRCKDTSRSRV

>DpKun-1d

AEERCNMTADYGRCQGNQLRWHFDSKSRHCHSFLYSGCGGNANRFESYQACASICKEAP

>DpKun-1e

DSCLLPRAEGPCSEKKSRWYYDQAERRCMPFYFGGCQGNANNFESQNACEESCRALSLV

>DpKun-1f

DTCRMPRVIGDCKEFTERWYYDEADEECRAFLFGGCNGNANNFDSMDSCNQRCKSTVSPIVPE

>DpKun-1g

TEFCFLPKQEGSCDESVLQWFYDRPEGVCKQFIYKGCDGNQNRFADRQECESRCSQSQ

>DpKun-1h

DVCILPRVVGPCSGSFRQWYYDAGSDNCYEFDYGGCQGNPNRFNNAQECQNRCQRVRPITTT

>DpKun-1i

TGGDICGLEVEPGPCRASVPAWYFNRQTSRCEAFSYGGCDGNANRFHSEEQCERQCGSF

>DpKun-1j

QDVCRLPPDRGPCRGSFRKYYFDRSSLQCLELVYGGCRGNGNRFSSLEECQSLCLQRAEVAPPGNVTS

>DpKun-1k

VVCRLPMDVGPCRERYDRWYFDSERSTCQPFVYGGCAGNMNRFKSFESCTTFCSPSDRTRPAEPA

>DpKun-2a

KVDDEDDCSLPAIQPGIKKSCSGFLTRWTFNNGACQQITYGGCGGTKNLFETEYACNAKCNR

>DpKun-2b

PSPCHLPSAAGYCRAHIPSFYFDSVSGECKSFVYTGCKGNANNFPSMEDCRKTCKVRQI

>DpKun-2c

ICSLPPDNSKTTGRACMAFVPSWTFNSTSNKCESYVYGGCGKTANLFRTEEACQSTCGST

>DpKun-2d

NQESCLLPVAKGPCFGFMKRYGFNKEKNRCELFTYGGCQGNFNNYVTADQCFDACGGALPSLASECEQ

>DpKun-2e

LCLLPRDIGRCRASVPSFYYDADQLKCVLFNFGGCHGNENRFSSEAECLSTCQHSDVAETG

>DpKun-2f

LQRCLMPMHIGPCRMSLEKFYYDAEKKDCLLFFYGGCKGNSNQFDTVEECRQTCRVKSED

>DpKun-2g

KPSVCEMPQEVGPCKGQVPAYFYNKDSGACESFWFGGCRGNANRFETEAECQTKCIPSSLTPV

>DpKun-2h

VAEHCKLPADIGPCRAAKPRYHYNLTAGECQPFNFGGCRGNNNNFQTIEQCQSECAAGGAVN

>DpKun-2i

INRCKLPADVGFCRSFQERFYYDSIESQCKTFSWGGCRGNSNNFPTSEECMVTCDRQGKLAA

>DpKun-3a

VDVCSLPPVNPSPIACSGLIPRWTYNAKAGLCEKYTYGGCFGTENLFKNEFACLAKCNK

>DpKun-3b

ATAPCMQPKATGNCRAFIPSFFFDTQTGLCTSFTYTGCGGNDNNFSSEDECDLKCNGLQG

>DpKun-3c

EKCSLPPVNPSPFSCLAFIPSWTFNSTTGECQSYVYGGCGKTANLYNSHDECNTACGPD

>DpKun-3d

DSKCTLPPVTPSPFSCLAAIPSWTFNSTAGKCESYLYGGCGKTANLFNSQDDCNAACGPK

>DpKun-3e

QEICQSPVVKGPCFALWKRFAFIKEKNRCELFYFGGCQGNRNNFRTADDCYKTCGGDEPN

>DpKun-3f

DLCFLPKVIGPCKMSRPSFHFDATKGDCRPFLYGGCKGNENRFETLEACLDTCSSVA

>DpKun-3g

RPSVCLQPKVTGPCRGLETNYFFDSTKEKCLAFNYGGCEGNDNRFETLEKCRQVCGEI

>DpKun-3h

FARCKLPADVGMCRGFAQRFFYDNADKECKPFTYGGCLGNANNFPSQEECHSACAPPVIDAKAR

>DpKun-4a

NNEEVDICALPPVNPGSKACRGFFRRWTYDIKTETCATYIYGGCGGTENLFQTEFACLAKCNKPGL

>DpKun-4b

APCMQPKAEGLCRAVIPSFYFDVQTGKCTMFDYSGCHGNSNRFATEEECEQECYDFPN

>DpKun-4c

DVCSLRVVNPGPKRCKINTQRRWTYSAKTKACEIFEYIPCTSKDPPNLFLNEYACLANCNQQ

>DpKun-4d

SPCMQPKAAGNCRASFHSFYFDKKTGMCTAFTYTGCGGNENQFSSEEECYLKCNNV

>DpKun-5a

ASGCFLPLSRGICRAHWVRYYFDPSSRSCQSFVYSGCGENGNNFHSLNECRLASAVRV

>DpKun-5b

DPVCLLPPVADPTQNCNNFSIKYYFDPVDGDCEDFLYSGCGATENVFNSELACELRCENVHDQLN

>DpKun-5c

CENVHDQLNSHESIERCNQFSIKWYYDSATRDCEKIFYTGCGGSENLFASEDLCEMRCDQD

>DpKun-6

SDDICMLPPLKGTLSCSGVFYRWTYNSTTEVCEKFVYGGCQATENVFRNQHACLAKC

>DpKun-7a

PSVCFLPPIEGSIQCKGFFIRWTYNAQTEKCEKFIYGGCFGTANLFRNQHACL

>DpKun-7b

GSSICLQKKDEGSRSCSASIPSYFFEATSGLCKPFRFSGCDGNGNRFPTEQECEQACYYGPSVAAVCDPALSTV

>DpKun-8a

MSLMLVTLQAAQFDDVCSLKRESVKCRTLLPTWYFDKATGTCYSFNIGECSRNFNSFGTKKICEKRC

>DpKun-8b

PANVCSLKKDSGMCRAAVTAWYFDPPTDSRNKGECKNFLYGGCGGNANRFASKKKCDDMCV

>DpKun-9a

GIEICDLPPIENKGFECYALKHSWTFKSGKCVNYVYGGCLGTENLFDTEEASC

>DpKun-9b

SCDLPPVAKQSTATCLAYFPSWTFDSKSGKCKQYVYGGCHKTENLYETEADCLSKCGPAVSK

>DpKun-10

TTTHACQTPADPGPCTGELIRFFYDSTALRCRQFIYGGCEGNKNNFGTEADCMKMCAHTLVD

>DpKun-11

IVNVCELPKEPGMCRANFPRWAYNPETQLCEKFSFGGCGGNANNFHSHQQCASRC

>DpKun-12

SACLKPKVTGPCRAAIPSFFFDATTGVCTPFNYGGCGGNDNRFATEKACQLACSAPTNSEENN

>DpKun-13

CFLPLSKGFCRAHWVRYYYDPSSRSCKSFVYGGCDGNGNNFHSLNECRLACLN

>DpKun-14

AICMEEVDVGPCRSSSFPRWYFDAHKGMCISFNYGGCRGNRNNFEKREDCVNTCE

>DpKun-15

CLQPKVIGPCRASIPRFFFDATTGVCTPFNYGGCRGNDNRFISEKACQLAC

>DpKun-16

CLQPKLIGNCRSSIPSFYYDATTGVCRPFNFSGCDGNSNNFGSVKSCERACMGPDFLSDLPPSMSN

>DpKun-17

PDYCSLPPVMAGEKICKGYIRKWTFNETESACASYVYGGCNGTKNLFDTEEECQAACP

>DpKun-18

CMQPKAVGSCRGSIPSYFFDMQTGQCTPFNYGGCGGNDNRFKSYHECDFKC

>DpKun-19

DVCFQEKLLGSCKASVLRYSFNEKRNRCEAFRYTGCSGNMNNFDSERECKAVCP

>DpKun-20

TDVCSLKRESVKCQTLLPNWYFDKATGTCYSFNIQKCSRNVNSFGTKKICEKRCA

>DpKun-21

CSQPAETGHCWALFPRYFYNVSSRSCEEFISGGCEGNENNYESVQECSQHC

>DpKun-22

MCSLPPVDPSNISCFAFIPSWTFNSALGRCQSFVYGGCGRTANLFDTQNDCDAACGSESNSGETIRF

>SmKun-1a

GVVCGLVEDRGSCSNYTVKWRFDMEYGGCARFWYGGCEGNDNRFDSQEECEEVCVIP

>SmKun-1b

NACQLPKATGQCTTYTVAWYYDVPSGQCGQFYYGGCLGNNNRFATQEECERTCGHT

>SmKun-1c

EDPCQLPKLEGPCNNNYISWYFDKDEVMCKEFRYGGCKGNRNNFPTERDCIQQCASK

>SmKun-1d

KEACLLPKEVGPCSGQYTRWFYNIVESQCKPFNYGGCQGNTNRFESLQQCQVSCND

>SmKun-1e

VGDVCSLPKVEGPCQERAVKWYHDSNEDRCLQFYYGGCDGNANRFETELECQHVCSA

>SmKun-1f

ADVCALPSDHGPCSSAAEERWYFNQNSGRCDQFIYGGCGGNGNNYVSLAECEHRCGVKI

>SmKun-1g

LEDCHLDKAYGRCDDSTIRWYYKKDTGVCEQFHYSGCNGNNNRFETRPECEEKCFHS

>SmKun-1h

QEICELPKVVGPCSGQFSQWYFNPQADQCEEFQFGGCLGNGNRFNTFEDCQARCKKTV

>SmKun-1i

SADPCTLPRDVGPCQSSVHSWYFDGQERACKAFVFGGCGGNTNRFVSEESCKRACGEF

>SmKun-1j

HDVCSLPNEHGPCVGEFPKWYHDPTDNECKTFVYGGCSGNGNRFESKKECEQVCIAR

>SmKun-1k

SEICRLPPDAGPCTNFERRWHYDSVRGTCIPFDFGGCRGNKNRFKSFDVCLGFCS

>SmKun-2a

VICSLPRRAGSCEALLERWFYNSDTSRCESFVFGGCGGNLNNFITRDDCEKSCVVN

>SmKun-2b

EVCSQPQDPGLCMAAIPRWSFHAPTNTCQEFTYGGCGGNLNNFQTQADCRFVCPVI

>SmKun-2c

EKCSQPRVVGVCRASYRRYNYNRTTEQCEQFVYGGCGGNDNNFNSMEECAAECDA

>SmKun-2d

NVCEQPKRVGMCRASMRRWYFDQFSHRCEEFIFGGCDGNDNNFETREECEARCPDL

>SmKun-3

YDVCSLPKVEGPCKELSVKWRYDSNKDRCLQFYYGGCDGNANRFETELECQHVCS

>SmKun-4

DCYASSEPGPCDALIPKYYYSHQSDTCQAFTYGGCGGNLNKFDTVQECEDKY

>SmKun-5

EICMQESHVGPCRGYFPRWYFDLGKAMCLQFIYGGCRGNKNNFERYEECNEMCAF

>SmKun-6

YCDNPAKKGNCDGEIQRFYYDEKSHSCKKFIYTGCDGNENNFHTEAQCMEFCSEN

**C. I4 Serpin.**

>ApSRP-1

SLANHDFSFSLYKELAKTENGNIFFSPFSIHVIMFMASMGAASKTFDEMINTIHLNETTHSMEGYRTLLEDLLSNNENLKMATGMFVDETFNVKKSFVENSMKYLKSSMEKKNFKDDPEKQRKYLNDWVLSKTNNKIKDLFPKDSITKDTALVLANAVHFQSSWVYKFKDAEDDSFYITPSNKVPVKMMTLVHDLQYYHDSDLKFAALELPYEHYAFKMIILLPDAKDGLKELENNLSKINLNDISNKMSQYHVTVKLPRFKLEQSLQLEDTLSNLGCPTMFTQAANFSNIVEHGDLHVSKVLHKAYVDVNEKGTEAAAATVIMVMYSARYPIEPLKKVEFHADRPFVVAVASRNNDVLFMGRLSNP

>ApSRP-2

CANHDFSFSLYREVAKTETGNIFYSPFSIHVIMFMASTGAASKTFDEMVATIHLNETTHSLEAYEKLLEDLTSGNDNLKLATGMFVDTAFNVKDSFVENSKKYLKSSIEKLNFKNDPEQQRQYLNNWVLNETNNKIKDIFPKDSINNDTALVLANAVHFKSAWAHQFKHVYDGSFYVTPSDKVPVKMMYLERDFQYYHDSVLNFTAIEIPYKNYAFKMIILLPDAKDGLNNLENDISKFKLHDISKKMTQHYLSVRLPRFKIEQSLQLDETLSNLGCPTMFSQAANFSNIVEDGKLNVSKVLHKAYINVDEFGTEAAAVTTIQFQLLCARSSIDVTVDHPFMFFISTKCNSIIFVGRMTKID

>ApSRP-3a

FHAFNWRLCKALHDVEKNNAVISSISIKLVLLMLYEGALGNTAKQIEQVVGISGHKQNIRERYSQKLQSLQSHGKDDYELDIGTKLFMDVSVQPKPDFIETISRWYNSSLEVVDFSKPVNAVNSINQWAEILTHGRIQQLISEAETKESTVLLLLNAIYFKGYWTTPFNKELTKRGAFYINSKTAIDVQLMTAYSNFKSSTIESLNAKLLSLPYQGNKFVMYIILPDENGLDDLINKINPILLGESIKNMKTFSTKVVLPRFSFEYTSILGPLLQKLGITDMFGQNANLTNLGNDGQFGSLIVSNILQKAGLEVNEQGSTAHAATEVELDHRFGDPIEVYFEVNRPFLFMIEDITMDTIVFVGQVMNPLS

>ApSRP-3b

YFDRELFQELSLNQPDSNVVVSPASIKTMLTLLSEGARGDTLDQLNRVLRLPTDQSTFHNVLHANQLSMESSLIDLVVVNNIFVKNKNSISNNFKETAQDKYSANITEINLLNIEASVKMINKQISDATQGLINSVISKDDFDGNTELLLTNVLYFKGDWLLKFNENSTKNQCFYTKPSMCVEANMMNLQNQLGYGYIPDIKAQVIELLYKDNNFSMVILLPDENTSTVQVLKDSQHNSFSKILNSIDERAVNLYLPRFKIDFSTKLPSVLKKMGLTSIFSSNANLTSIFNPAKQVLVKDITHKVTMEVNEKGSKAGAVTVVSVIPLSNIPQPSPVTVLIDRPFIFYIFNRATKNILFSGQVYDV

>ApSRP-5

STIHNFSFSMYKEVSKTETGNIFFSPFGIHLIMFMASTGAASNTFDEMVATIHLNETSWKTDQTLEAYRQLLEDLTSANDNLKLATGMFVDTDFDVKDSFVENSKKYLKSSMEKLDFRNDPERQRQYLNNWVLIQTNNKIKGFFSCKDSITQDTSLVLVNAVHFKSDWAHKCIYVYDGSFYVTPRNKVTVKMMSLIRDFQYLHDTVLKFKALELPYKHHGFKMTILLPDDKNGLKNLENNFSKFKIHEISEKMTQNYVKVKLPRFKIEQSLELDKTLSNLGCSTMFTPGAANFSNIVENDELYVTKILHKAYIDVDEDGTEAAAVTSLIFKKGSGLSKDFIVDHPFIFFISTRCNFILFVGRMTKID

>ApSRP-6

LTSNYSFSLFKELSSSVEGNVFVSTYSIQFLLLLLAFGSKSKTNDQLKSVLHLSKDKPPNFENIKSVIAKLEVPGHLTVANGIFSDKAFSLNPEYTKNTQKYLNSEVRSVDFSGNPTSGESELNKWVSTKTNGKISGIFKPGEIKKETVLVLASAVHFQNLWKKQFAETKNASFCLTATNHIDIKMMHQTGHFKYYKDNHLKFAAVEIPYKVGGYEMLIILPDKMDAVKDLENVFLKKSKNYAHLLSNMTIHNVELDVPKFKFESEMDLIKTMQKLGLTEIFLPTADFSELSSSGAGKLKVSSMKHKTYVDVNEKGTEAAAVTGAGIENYNLEYVPKDIKNVKFHACHPFLFIIKKDKDILFMGRLSNPTA

>ApSRP-8

SHGSNHDHNIVISPICIAAAMSLVLLGAHGETKTEVGKLFGYDEITLSHLADNNNQNYKDLGRLLNYFQTNSGEKLGTEVNLAKAVFVQNGYNLTKNFIKAAEDYLNTKLVTVDFKSDGEHTKHIINQWVSNQTRGKINDILPDIPSADTKTIIASALYFTGEWENPFFINYTRIKPFCYGAITGDRKSSKQNKDCIYVQMMVGSSEVLFHKNEGLEFKAIGLPYKGNQFITYFVLPDTNISLSSLTAKMNGKTIKNITRTAKITELTYFVPKMTLKSLTNLRPVLQNLGINKMFDPSKADLSNMASDPGSYISDILHQVEIDVNEIGTVASAATVVTITRGGQTIFNINKPFIFFIHHVESDTVVFWSTVYKPMPYS

>ApSRP-10

LLKATSDDNFNYVVSPFASSVILALAAEGADSETKSQLVATLGGELPDKNSYKEVLSTIKGYSLDGFAQNKVVLKNFLYVYKNYSVHESYAQLARDYYLTDVRSVARPDLEMKRAATNNIAADDDESTADFKEHALLIFNGLSLEMTWPKSTWHKTSMSWNGKVVKAFGAAGNFAIAHIPSLECTALKLPYKNTDYALLVLLPKNKDVSLNEVLKKLKPENGIEKLTKAMTIKPSFVTMPCFQSSNITHLKTVLQQGTQTNSVFTESADLSKLSSDKLYLDDVVQQANLRVCVDGTSSSALTSSAFTSQRVIKESVVMDRPFAYALYNVANGIVYAAGKLEQPVWEDT

>ApSRP-11

CNELSFKMWTAVTGKGQIASRSLVLSPFELTAMLAMVFLGARGSTSGQMNDVLRLDDMVTFNPHQVLRNITHSITNINNPGVATASFVREIYSHKGNGKILEFYKERVQQYYDGHVEEVDFNTIGDVLRRRTNLLVKRQTLGRVVEYLRGSGLSLTPPFAAFSANVFQTSCESASTEGRDGEMYFVVRPSTRQRRLVPVPAAVWRGGFLAGYEPGLDATAVCLGPDSAVSTILVLPGQQGQVAPGDGLARLEQRLIETSYRRGGWSRVLRSLLPRPGLELQVPRFSHRSVLNATAALQKMGLRDVFSDQKADLRGVNGLYDLYLSDMLQVNTFSTCGEDTIGARHHVETYPASPQRMGRAGHESGPGSDHGDGHGSDDESVRDGGRRKRNAEDLPLHSPYSHLPLNLRPRQARLPDVPRLRFDRPFLYLVRHNPTGMIIYLGRFNP

>ApSRP-12

TNFRYTSLTSNYSFTLFKELSHSVEGNVFVSTYSIQFLLVLLALGSKSKTGDQLKTLLRLPQKNAEPNYDNIKSVMTNIEDPDYFTTANAVFTDMAFVLRNDYVDKVRVYLNAEVKSLDFAGNPEKEVSEINKWAEHKTDGKISNIFEPGTIDRDTVLVLASVAYFRNAWKNQFTDTKNASFCLTPSKHIDVEMMHQRGLFRYHHDDRYKFSAVELPYKAGGFDMLVILPDRADGLNDLENAFLKDSKNFAYLQGNLTVHDVTVDLPKFKFESDVSLIKTMEKLGCTEMFTSSADFSYISTSGAGKLKVSDIKHKAFVNVDETGTEATGVTGYSSKYKKSEYSPSDVKAVKFHACHSFMFIIKKNTNIIFMGRLSNPNP

>ApSRP-13

YESLSLANHDFSFSLYKELAKTDEGNIFFSPFSIHVIMFMASIGAASKTFDEIINTIHLNKTTHSMEGYRELLEDLLSDNDSLKLATGMFVDEIFKVKKSFVENSMKYLKSSIEKLNFKNEPKEQRRYLNNWVLSKTNKKIKDSITNDTALILANAVHFQSTWIYSFNDAEDDSFYVTPSDKVPVKMMTLVDNLQYYHDNDLQFAALKLPYKFYAFNMIILLPDVKDGLKDLENNFSKINLNDISNKMSKYDVTVKLPRFKLEQSLQLDGTLSNLGCTTMFTESANFSNIVEHGELYVSKVSHKAYVHVNEKGTEGAAVNGLNAIIRTMPDRLPKVYFVADHPFLVSIVSRTKTILFMGRLSNPLK

>ApSRP-14

ELDDKVFKSINEINYLSDNKMKPLTTNLEILRSANHNFSFSVYKEVAKTETGNIFYSPFGIHLIMFMASTGAASKTFDEMVATLHLNETSYSMEAYRQLLEDLTSANYNLKLATGMFVDTDFDVKDSFVENSKKYLKSSMEKLDFRNDPERQRRYLNNWVLIQTNNKIKDFFSCKDSITKDTALVLVNAVHFKSDWAHTFKHVYDDFFYVTPSNKVTVKMMTLTRDFQYLHDTVLKFKALELPYKHHGFKMTILLPDDKNGLKNLENNFSKFKIHEISEKMTQNYVKVKLPRFKIIQSLELDKTLSNLGCPTMFTPGAANFSNIVEDGKLYVTKISHKAYVDVNEDGTEAAAVTSIHMNAGSGRSKDFIVDHPFIFFISTMCNFILFVGRMTKID

>ApSRP-15

LEALRSANHDFSFSLYKEVAKTETGNIFYSPFSIHVIMFIASIGAVAKTFDEMVATIHLNETTYSLEAYRQLLEELTNENDKLKLATGTFVDTAYNVKDSFVENSRKYLKSSSKKLNFKNDPERQRQYLNDWVLNETNNKIKDVFPTDSINHDTALVLANAVYFKSAWAHQFTRCIDGSFYVTPSNEVAVKMMIREHGFQYYHDDLLQFTALELPYENHSFKMIILLPDAKDGLNTLENNFSKINLHEISKNMTQHYIRVKLPSFKLEQSLQLKETLSNLGSPTMFTRAANFSNIVEDGNIYASKVVHKAYIDVNQYGTEAAAITKMEFIPRRARSYTDFIVDHPFMFFISTRYNTIIFVGRMTAIIN

>DmSRP-1

PPPNRPPPVFSYMDRFSSELFKEIIKSQSQQNVVFSPFSVHALLALIYGASDGKTFRELQKAGEFSKNAMAVAQDFESVIKYKKHLEGADLTLATKVYYNRELGGVNHSYDEYAKFYFSAGTEAVDMQNAKDTAAKINAWVMDTTRNKIRDLVTPTDVDPQTQALLVNAVYFQGRWEHEFATMDTSPYDFQHTNGRISKVAMMFNDDVYGLAELPELGATALELAYKDSATSMLILLPNETTGLGKMLQQLSRPEFDLNRVAHRLRRQSVAVRLPKFQFEFEQDMTEPLKNLGVHQMFTPNSQVTKLMDQPVRVSKILQKAYINVGEAGTEASAASYAKFVPLSLPPKPTEFVANRPFVFAVRTPASVLFIGHVEYPTPMSV

>DmSRP-2

ISGTSVKPSNLPAAYSNGYVDLATSDRIANSVLNFANILGQHLANGKTQIYSPLSIVHSLALLLLGAKGRSYEELSTVFDIPDTSRLHEQFGLMLQDLQQPTREAISAGRPLTDWRASSAMRSNRRAQRPGAHEVHLANGLFTQTGYTLNPDYRRVIVEVYASDLQIQDFEGSPATARYNINAYVAQHTKNHIENIIASDIPQTTRMILANALYFKAFWETDFIESATRPDNFYPNGEGTEPVMRVQMMATGGAYPYHEDHELGCKIIGLPYRGNLSTMYIIQPFKSSVRELMALQKRLTADKIESMISRMYRRAALVAFPKMHLTESVNLKTVMQRMGLGGIFSAVQNDLSLIATNEATRTNALGGNSLQNLEAQRRAGTGGARSDLVVDDIVHKVDFTVNEQGTEAAASSVTYLKKSGPDVLFRGDTPFMVLVRHDPTKLVLFYGLINEPPAAA

>DmSRP-3

MSEAANPTPYDCHIGAGIYHSIATSFAEQNVVVSPLLLEATLSLLFLGSDGATAEELQKQLRLKQRFASNAKMANFYAAELGNITTDADTFLQLQNRLMLSSESGVADDFQKIAQTYFHATAECVDLEQTEKLRRHISEQILASVGGGSWKDIHVAGGSSANTLLLLLAANLQSKWFLPFSAYRTGLYEFHSGSQVKSVPMLFDDDMFVKFAELRDLDARAIELPYEHAEELSMLLILPNQRGGLQELEKQLHDLDLGALQQRMQMEGVQVLLPKFSIDFECSLRQPLKQLGFEEIFAASANFKHLHASANLPIADVLQKLRINLNESGSGSGPELPKNATEYKPIVISNSSRQKFFRADHPFFFAIRSENVTYLMGHVVEF

>DmSRP-4

MKDEEFAQGLEQFALCLHDHLCRASAGLNIIYSPLSIHISAAMLRMGTSEGSATAKEMDEGLRFGGLEAQQVAESFGVVLKSYEQCQVLKMANGLYVMKGLQVDEQFGHILEQKFRSKPMEIDFGSEQAASIINKWVESQTNNLIKDIIGPRVLTKDSRLCLVNGIHFKGEWSISFNEKETREEDFFGSDRPTRVRMMHVCENFFFAVLPMFEATALRMNYSACNLAMIILLPDEKSNLTSLEKKLSDISLEVVSSAMNLEKVDVKIPSFTAEFQQELSQVLMLMGMNRIFSGQAELGGMLQSEESLFVSQIVHKAFIEINEVGTEAAAATAAVATFRSMPARQGPPKVFHANRPFFYAIKDNTHGLLFAGHFITTKVEQSEKCN

>DmSRP-5

MSEPQEGRNQFARNLIDVITKDALQQSKDPHINTVFSPASVQSALTLAFMGASGSTAEELRNGLQLGPGDRHHIALNFGEFWRTSCNYGDRGPVLKSVNRLYVNDSLELLTEFNEIAVDFFQSKAEATRFADSEGATQLINDWVEQETEHKITNLLQSDAVNNETSALLINVLYFKGKWQKPFMPETTSIDHFHVDRDTHVQVNMMYQEDKFRFAELPQLKARAVQLPYDYSNIHMLILLPNEVNGLQELEQQLNTVDLADIDAALTLQDVEIFLPRMCIEYDVDLKQVLNQLGITEVFSDKAKLDGLFTSQSGQKISAARHRGYIDVNEAGSEAAAVSFMKIVPMMLNMNKKLFKADHPFVFYIRNPQAVFFAGRFSNPKSG

>DmSRP-6

MANTLNYSKSPAGEAQFASQLFGQLAKSQSGRNIVFSPSSIRTGLALAYLGAEGSTADELKLGLGLEGAGKTEVAEKLDQLLAKGQWEKASGDEDVPKLKYANRIFVTQRFKLTQTYQDLVSKNFAAAAENVNFTQKADTAKHINSWVEEQTHQQIKDLIAPESLDADTSAILVNAIYFKADWQSSFPDYATYASDFVNHGGRKVSVDTMSQEDYFRFGELTELKAKVVELPYTGTDIVFLIILPQEEQGLAIVEEKLMGIDLNEISSQLRRRKVRVQLPKFKFEFDVPLQAALEELGIKKLFSPGANLSSLYQGSEPLRISEVKHKAIIEVNEKGTTASGATFIKVSVESLTIGEEVFEFIADHPFFFAIKDAQNTLFLGHVSQL

>DmSRP-7

MASGGTTAPSLSASPIVFARNLFRALNDEVPPVNMMVSPAGARSAMTLVFMGAGGKSADELRSKLILGVSNKSEVAKQHAESWTDECSCAKKGVALRLVTRLYVNEEEKIRTDFNDMALEFFNAEAYSLNYLNPEDSVKKVNKWLEKHTFYTVRNLFTPEVFNSDSSVILVNSLFFRAKWNKIFPQQLTQIDDFWINPRQRMEVSMMRQIGQFRYGESKKLKSQILQLPFERSNLTMMIILPTAIDGLPELEEKLGQLDMNEVAAKSLMKEVDVTIPKFRIECTVDLKVPLQKMGINSVFDAGQADLSDLFEMKTPQKISEARHKVFLNVTEFGCEVAPEAEVQPEVLKKNPDRKFFKADRPFVFAIRDRKNVYFVGHFVKP

>DmSRP-8

MYVLLLILLGISRYRAQKNSQLPDELYAAIVNSFSNRNIMFSTEMIRSSMLFIYVGVEEDESEQIRKAMHYRGTHLSEYKPKTQKIFAMSVKKAPVAKSLTRFYVRQNMKMSTEYRVFMRHTEGRARNIAFAREQLDEVNTFYSHEMGEQIGQVVKESWWKPNSQGLLVNAIFFNLSWERTFNPEATYPREFRVNATKSVMIPMMHEDSKFAFGILGNLKATAVLVPFSHGDLRMLLIKPDQPDGLAALQMKLQAMNILSVARNLTMMDVFVGIPKFKIHSDLELSPAFEKMGIKDIFKPSKSFSTLLHRNTNFRIDGVIHVVTFEFQEQGIGTPSTDVGNGSLTHTFNGVKYFLATHPFAFYIIDNTSIYFAGHVTSF

>DmSRP-9

MKLGFLGLFGMVLMIMFYEGAEGYTVNELRDVLGIYVDYPTLRRWYEDVRAYHYLNSENTKLFSLRYAYYDDVGDLELVKGYNSVVLEGVGEGNVVLREGRPRGVDFDQGASIIINDDIDKASHAKIFSSYSRRSFNSTVTVLGITVSYFKAKWKYPFDKSQTKVEQFYNDGGSPAGKVEMMVQTGKYAYVNNVKGLQADVLELPFGEHELVMIVLLPKSSQGVNLVLYQLKNLGLHRLLEKLEASKNETDVEVKLPKFDTRSVLSLEDTVYDAGLTDLRNDFADLDKLLIAIGHRGACLTLYHQFARIVVDEEGLPNAVPQKSSGKNNIKFHVNRPFAYLVLQKKHKLLIHSGVFREGEIQ

>DmSRP-10

MKLGFLGLFGMVLSLRAHICANTVDTKSLLQKMTDARLQFALNLLQMESTHLNLENFAMTPFSTWSLMIMFYEGAEGTTLKQIRDVLAIYVDYPTLRRWYEDVRAYHYLNSENTKLFSLRYAYYDDVGDLELVKGYNSVVLEGVGEGNVVLREGRPRGVDFDQGASIIINDDIDKASHAKIFSSYSRRSFNSTVTVLGITVSYFKAKWKYPFDKSQTKVEQFYNAGGSPAGKVEMMVQTGKYAYVNNVKGLQADVLELPFGEHELVMIVILPKPSQRVSLVLKQLKNLGLHRLLEELEASKNESDVEVKLPKFDTRSVLSLEDTVYEAGLTDLRNEFADLGRMLIPTGDRGAYLSLYHQFARIVVDEEGLPNAVPQKSSGTNNIKFHINRPFAYLVLQRTHKLLIHSGVFREGEIQ

>DmSRP-11

MHTFSLVLLALLPVVTIAALDKPELSFLNEFSQIFKGERDFSLALMKQIREIYPSGNLFFSPFSTYNALLLAYFSSSEQTERELAQALNLGWALNKQQVLVSYTLAQRQDEFRWRQSPMELSSANRIFVDRTINVSNKFNTLLYGATKELDFKNDPETGLKEINDWIADKTHNQIRDMLSSEEITPHTMLVLANAAYMKGQWLSQFKVEETALKPFFINEREQEMVYMMHKTGAFKMTIDEGLQSQIIKLPYRTIYKSKETHISTPESKSDISMIIILPNSNKISLNRVISRLNADSVKKWFERALPQKIELSLPKFQFEQRLELTPILSLMGVNTMFTRNATFGDLTADPISLVIDDAQHLAKIKVDEVGSTAAAATILLVSRSSRQPDPTKFNCNHPFVFLIYDEKVDTILFAGVYSDPRQMQH

>DmSRP-12

MIHWRLLSALLVGLAIALTLPVDGELLARSPASVSSNRFGLRLTTKLGLTQPDANVVVSPLLIQAALSLLYAESSSEYGSQLRQALELTHASHPKLAVQDFETLLTDLKQSAAIGCRLRLLSDLYAQQRFTFNFRNEFETLAARMGVGCHRLSWESASNAAQDINYAFLSRSNFSLGELVSAPQLESLAEHNTPFLHVSGVTFRAPWAWAFDPTETQSINFFAGGNRPRLVDAMFGQHRYRYAEVPALDAQLIEVPFATADLRMLIVFPNRPDGLAQLERKLAQSDLHQLRSQLEERKVALTLPKLRVLVHSDLKHVLEELGLAKLFTSEVHLSEVFSSILSSSAPPLGAVVQSGLLELQEDGGNADDSFSFGDLFRRALPLVINHPFFYAIGNGKTLLLSGHIVDI

>DmSRP-13

MKYLCWILVTTSVLGQFTKQLYRSFLQDNKQYNIIASPLCVEIGMSMILMGADGNTANELRTALNLPEDKKNVATIYDKLLTKLERGKKVAILHLANRLFVNETIGVNKRYNKLVNKHFRAEAEAIKLADRLKAAWAINDWVLDQTLDNVKDIIIPSDLTPDESAVMINAAFFKGYWKTRFDKMNTKPKVFYVSKSYQVNVNMMSQVGRFKMRTSTIDQIIELPFAYSNLSMVIVLPKDNGSLTQAEATIESYPQIVLTEMDVHVQLPKFKIDFRMELVETLKSMGIQDLFNSSSDISVLLNQSGTRISQVVHKAFIEIDEEGGSAGSASASPIRGLSDYATSVVTFTVNSPFVFMIRDDDNIYFRGRVVDPLKKSNPIDRITI

>DmSRP-14

MFKRVLICFLIFLLKHSYAYNFEINLTKQLSKGRLARNFVYSPIAIRQALGLLYLSKDNVTDQQLESALQLTGLNQEEIISLFKEAREKVAQEQFTMGNRIYLSPDYNASPNITQLSENLGVEVKNMTFSGDQSAASEIKKWLNKWIGKAGGNLFGKNDISQTTQIVAVQGMSYSCVWKNRETALTNRTFTLLRQNKKPFVYTTQMMYTEAPMDFFNNDQVRGVMVPFKNSDMGMLVLLPRPRYSTQQILYSLDTILKIKLRRSKKTHLFLPKFKVSESVDLNMALKALGIQNLFTNTNAANFKQYNSFDADQNRVLMTIDVGDDFDDRVVYVNRGFVFVVKDKSTIYMIGRMDAV

>DmSRP-15

MDKAESNFIASPLCIEIGISMILMGAKGTTAEELRSVLDLPVDVTEMAKKYERIMSNFQKHNGLRFTNWLYVNETYEVRQDYNTLMKSTFMAEGKDPLSQRKASNSISFSIHRKSHKGMRTISNDHNLQINESAVLVNTVYYSGAWKTRFSKKDTKLKVFHGDHNKKVYVRMMSHVGRFRIADHSYGQIIEMPFDNSDLSMIIGLPLHNTYLSSIEKILRTLSESLVENNVHVELPKFKIKYQTELVESLKKLGIHLIFSNTSDLSGLLTNGTGAKINHVVHKSFIEINERGASTGEASDHAESIQKKTRASTSFKVNRPFVFLIRDKHTVYFRGRVVRLPNELHL

>DmSRP-16

MKYLYLLLLATSVESGFWEDFYRILASQNAKRNLIYSPISAEIIMSMVYMASGGKTFEELRNVLKFSENKTLVANNYRSLLSDLKRRETFIILHMANRIYVNKKYCLVPEFNQLARKAFKAKAKSIRLDDPVSASAIVNSWILNRTRGMIRNIVLPKDFNSDTSAFLVNAIYFKGQWLYNFKADQTHIADFYVSANEIIPVKMMTLSASLLSGYIDDIDAKIIELPYWNSTLSMRIILPNSVDGLRKLKEKVGFIDYHLEKKSVNVKLPKFKIESKAQLKGIFENLGILDVFKPSADLNGLVLESGAKIDKIVQKAFLKIDEKGGEASAATGVLTRRKKSIDNLIQPPMEFIADHPFFYVIHDNKVIYFQGHIVEPRW

>DmSRP-17

TAPTAFQSGVSHIQSMRSNFDTDVLVSISQGVQDFALDLLQRISVEVEKANKDFMISPFSVWSLLVLLYEGSEGETRNQLKKSLRINVEDEKLRGAYKVWSSFLNITTSTIEVATLQAIYTGKGYPIKNNYRDAIQNYNVQPMEVDFYSPDSVIQINEDTNRTTRGLIPYTILPQDVYGAKMFLLSSLYFKGQWKFPFNKTLTREEPFFSESGEVIGKIPMMVQEANFAYVSNVEGLDGYVLELPYGTQDRLAMIVVLPKRGFKLNDVANNLKALGLRPILQRLAAFRNRASEDNEVEVMMPKFVTATDFTLKGVLIQMGIRDLFDENTANLDRMSSGLFAKLVVHSTKIIVDEQGTTAGAVTEAALANKATPPKFLLNRPFQYMIVEKATGLLLFAGQVRNPKAA

>DmSRP-18

PVHTADVTMADAAHQEFARRLALFSINVYGKLSGQKPGENIVFSPFSIQTCAAMARLGAENETATQLDQGLGLASSDPEQIAHSFHQVLAAYQDSQILRIANKIFVMDGYQLRQEFDQLLSKQFLSAAQSVDFSKNVQAAATINNWVEQRTNHLIKDLVPADVLNSESRLVLVNAIHFKGTWQHQFAKHLTRPDTFHLDGERTVQVPMMSLKERFRYADLPALDAMALELPYKDSDLSMLIVLPNTKTGLPALEEKLRLTTLSQITQSLYETKVALKLPRFKAEFQVELSEVFQKLGMSRMFSDQAEFGKMLQSPEPLKVSAIIHKAFIEVNEEGTEAAAATGMAVRRKRAIMSPEEPIEFFADHPFTYVLVHQKDLPLFWGSVVRLEENTFASSEHDEL

>DmSRP-19

MAVIISCLLLLLATVSQSKTVGYDAAADRNLLAADLYNAVAADHLNENVVISPATIQSSMALAFVGAKGQTASELQQGLRLGPGDADAVSQRSGSYQQALTRDNNFRLANNIYINENLEFKGSFRDVAQRQFDSNIDKLDFHPPYNKRTADGINRAVATKTNGKITDILRAELLNDRTEGVIVNGVSYSAAWQKAFRLDKTEKRSFRTGSGQSVKVDTMWTLQNFNYAEVNSLDAKVVELPYQNPDFSMLLLLPNRKDGLRSLQQSLSGKNLLAEIGAMSQQKVEVLLPKFSVTFGLGLEGPFKKLGVHTMFSRDGDFGNMYRMFVSHFINAVEHKANVEVTEAGVDQPLETGLLKGLFSRSKKFEADHPFVFAIKYKDSIAFIGHIANYAYV

>DmSRP-20

MNHWLSIILLGVWISAPEGLGNTIKDRNLFATELFQTLATDRQDENVIISPVSIQLALGLAYYGAEGRTAAELQKTLHASAKESKDGLAESYHNLLHSYIKSKTVLEIANKVYTRQNLTVSSHFREVAQKYFDSEVEPLDFSRETEAVEQINRWVKQQTENKIERVVESLEPDTNVALVNAIYFKARWARPFNDEDTRDREFWLSESRSIQVPTMFADNWYYYADYPELDAKAIELFFENINLTMWFILPNQRSGLQALEQKLKGVDFNLLEDRWQWQSVSVYLPKFKFEFDTDLRPTLHKMGISAMFSDAADFSNIFQDSPIGTRITKVQHKTFIDVNEIGCEAAGASYAAGVPMSLPLDPKTFVADHPFAFIIRDKHAVYFTGHIVKF

>DmSRP-21

FNLEFARGGARFTSELFQLLSAGGLKENVVFSPFSIQTCIALAFAGSQGETADEIAKALHFVSNFPPEVAQTFQFVLEKYRNSNLLRVANKLYVQEGKQLKPAYQSAIKEQYHSEAESINFALNDAAAQAINAWVNAKTQGKITELVSADSFSDNTRLVLLNALHFKGSWAHKFSEERTEEDIFWVGEEEQVKINYMNQKAKFNYGFFEDLGCTALEMPYQDSDLSMFVLLPQERTGIYALAEKLKTVNLVDLADKLTVEEVHVKFPKFKVDYSLELAEKLKQLGITKMFTDQAEFSNLLESPEGVFVSKVLHKATIEVNEEGTEAAAATGMIMMTRMMTFPLQFQADRPFLYVIWNKKNILFAGAFVKAA

>DmSRP-22

DAGLLDQRLNLYKGQQNFAVSMLNVIRQSTPNENVFFSPYSTYHALLLAYFGSSGDTEKELAKVLHLDWADSKEVVRSAYILEKMNRKERQSKMPLEFSSADRIFFANDLHVTECARNRLAEEVQQIDFKSQTEESRKQINDWIAKQTHDQIRNMLSADEITPRTRLVLANAAYLKGQWLSQFKTEKTVPMPFYTSPSNYSLVSMMQQKGTFLLNVDEQLRAHVLQLPYRTVFESQEKEDSSPDENSDISMVLILPPFNSNSLEDVLSRLNADSLDDSLKQAMPREIEVSLPKFEFEQRLELNPILAKMGVSKMFDESVATFDDLTSETISIGDSKHVAKIKVDEEGSTAAAATVLFTYRSARPVEPAKFECNHPFLFVIYDRTSRSILFTGIYRDPKTIKQ

>DmSRP-23

LLATSVSCRFTDDLYQLLAKENADKNLITSPLSVEIALSLAYMGARGKTAQEMRDVLKLPDDKKEVAAKFKDLLSKLEGRESVAILSLANRIYVNNKFKLVPEYNQMVKDSFKAEAEAISANNPKITASIVNKWVDTQTSGKIRDLVMPSDVANLVLVILNAIYFKGQWQKKFNTEQTKSDFHISDQKSVPVQMMSLVRPFGVSYDRELGANVIELPYRNSNLSMVIFLPDKVDGLPELEKKMVGFTPKLININVHLRLPKFKIEFSARLEQVLIAMGIQDAFKTSADFNDLVANSGAHVGGVVHKAFLEVNEEGSEAAAATAVVFRYKSIRSPPMDFNVNHPFAYVIRDAENIYFQGHFVNPEL

>DmSRP-24

LWVTSVACQTSKEIYQLLSKSHTNQNLVVSPVSIETILSMVFMGAEGSTAKELQSALGLPSEDKEAVAARYGALLNDLQGQEEGPILKLANRIYVNDQYSLNQNYNLAVREPFKSEAESISLTNGPVAAERINQWVLDQTSGKIKGMIDPGSMTSDVKALLVNAIYFKGQWESKFDPAKTRASTFQVTANKSVPVQMMAQMGTFRANYFRDLDAQVIELPYLNSNLSMTIFLPREVEGLSALEEKIVGFARPLVAKEVYLKLPKFKIEFRDELKETLEKLGIRELFTDKSDLSGLFADKSGGKVSQVSHKAFLEVNEEGAEAAGATSVAVTNRAGFSTFLMADHPFAFVIRDANTIYFQGRVVSP

>DmSRP-25

VPFRSDSHDPFSWHLLKTVLQNETADKNVIISPFSVKLVLALLAEAAGAGTQTQVELANTQTDIRSQNNVREFYRKTLNSFKKENQLHETLSVRTKLFTDSFIETQQKFTATLKHFYDSEVEALDFTNPEAAADAINAWAANITQGRLQQLVAPDNVRSSVMLLTNLIYFNGLWRRQFATTFQGSFFRSKDDQSRAEFMEQTDYFYYTTSEKLKAQILRLPYKGKNSLFVLLPYALNGIHDLVKNLENDELKSAQWAMEEVKVKVTLPKFHFDYQQNLKETLRSLGVREIFEDSASLPGLTRGADVAGKVKVSNILQKAGINVNEKGTEAYAATVVEIENKFGGSTAIEEFNVNRPFVFFIEEESTGNILFAGKVHSPTTQN

>DmSRP-26

LCNELAFSYWRAITSEKISSARSLVISPFALTSMLSMVFLGARGSTSGEMNEILKLDDMVTFNPHLIFKNITNSVEQASDSDIATAAFVREIFSDRANGKILPFFKEKTQQLYAGHVEEVNFHVVNDIVRRRTNLLVKRHTMGKVLEYLRTNSVWVNGPLATISANLFQTDCSHGSTTDRDGEMFFQVHPTVRQRRLVPIPAVLYRSGFLAGYEPSLDATVVSFGRVQNTVSTVYVMPGHQSSISPMDNLDRLERSLVETAFSDKQAWRRLLTSLMDRPGMEVQLPRFSHRSFVNASLGLQKMGLRGLFKSDFADLRGLTGAGNRDIFLSDMIQINTFSTCGEEKISEHHHVEMYPAPPLRKRNKDVDATDDDAFDSSERVVDFGSLVQESALGRGFYDDLLDPKYLELPLPLRPRQARVPDAPRLRFDKPFLYFVRHNPTGMILFMGRFNPRLL

>SmSRP-1

GLLNGSTDFGFNVFTRLAPNARHSESNMAFSPFSIWSALLMTYLGARGRTEDELNYLLGLKNASKADSGRAYKAVKYWYKLRSEHQINYTLNLANSLFLERTFPLRDCIETYFGSEISRVDFQRAPEAARAAINSWVEKETKNKIRDLFPPGMMGSSTLIAIVNAAYFKGKWQSQFKKENTRKEVFHVTPGRDVMIDMMHQTNLFYHGISADLDAQVLEMPFISEEVSMIFFLPLKDFLVDSVVRELSTERIRWLRHELRKSEVEVAIPKFRVENNFEMTQLLTQMGLRDLFIPSQSNLSGFSDDRRLAVSTVRHKAFLEVNEEGSEAAAATGILAWRSARPLSPTSFIANKPFIFMLHDNLSNVVLFLGVVNRPEIT

>SmSRP-2

DESGFSFIYDLYDRVSVESNDGNVLLSPFSVASVLALTLEGARGDTASQMLRVLHWNTLDTPGVRESIRDALSSMNRATRSYVLAVANRVFLQNGYDVLPNFRHNLRRYHLSDVQSLDFSRADSATKAINSWAARATRNRISELMSSGSVDRQTRLLLMNAVYFKGDWTTQFDAERTQLRPFFPTPTNEISTPMMYTQAEFGYAQLDDLQSSLLELPYRGRHLAMYLLLPDAPDGLDKMRRDLLRYPQIFYSPESFIVPQNVTVLLPKFRLEQNVRLKPVLAHMGIRDLFFQGKCDLSAISNTKDLFVSDVVHKSYLEVNEKGTKAAAVSAITIRGRMMQSETIFRADHPFMFFIQDKVTKTVLFIGHVKNLVDSAE

>SmSRP-3

KGLEFTLDLYKQTAVGNTENIFVSPLSISVALAMTLAGARENTAKEMKDVLKLGASFSTDLEIHEAFQDIINSLLGSQSSAIKLHIANRMYVHHAANILTDYKGTLEKHYNTSSNVVNFEADSEKIRLEINKWVEDQTQTKIKDLIAPGILNDLTRLVLVNAIYFKGNWHKQFDPEHTNADYFFLDANKAIMTDLMHLKSEFRFAEDDNLNCKIIELPYVDEAFSMFVILPTRVDGLTNLEETLNVPDLNNLMSRMRKTKVILTLPKFKIEASLSLKDILSAMGMKDLFSARDADLSGITGQKDLFVSAVLHKAFLEVNEEGSEAAAATAVVMMLRCAMTFDPEFRADHPFLVFIRDNRSGSIMFWGRVVNPNPDATVQE

>SmSRP-4b

AEIEFTLNLYKQCAAVSTSTENIFISPFSISLALCMTLAGARENTAKQMKDVLKLGASFNTDLEIHEAFQEIINSLLELQSSAIKLHIANRMYVHNKTNILHHFKSVLERYYSASSSEVNFEIEAEKATLDINQWVGEQTRRKIKNLIPPGALTACTRLVLVNAIYFKGNWCKPFDSEKTIAEHFFLDENRAIITNLMHETSDFNYIKDEDTLECQVLELPYADEALSMFVILPTEVDGLDRLEKSLTVSKLNELINGMRKQKVDVTLPKFKIESALSLEEFLSVLGMDDLFCPGIADLSGINDQKLYVSAIFHSAVLEVNEEGSEAAAATAILFVEYCMPFTVEFRANHPFLVCIRDNRSGCILFLGRMVNPDLNAAQQ

>SmSRP-5

ISNFKFSLDVIRNSVSKEENVLFSPFGASVALGVTSLAAQGKTLDEIQAALNLKTGNGNVSLEEEIKESIESMKNSEGGSIDFATCIFTQSSCGIMPFFGRKAIDIYQSDIQQLNFSIKKSAAFTINNWVAKHTHDKIMTLVDENSLNPDVQVMITNAIYFRGDWKLPFNSTFTKEKQFKNIKNQTTPVQMMRRTTDFQQAYDSDTNTRVLSLPYADNKTSMIILLPRNVDGLPKLLQSLTPETLQRMTAGLTYSHVTVELPKFKLEVEYSLKSTLSKLGIKDLFTTKLVHIFNTRANLTGIVCNNDVYVNDVIQKSFIEVEETGTTAAAATSIELIPLSKPFEFTVDHPFAFLIMDRVSEIVLFIGSVFDLP

>SmSRP-6

KGNIHFVLKFYKQCVNDSTENALLSPLSVSMALAMVSIGARGDTAARLREVLCLGVEDVNIHAALNDVLESFRSSFGLNLHTLNRVYVHEELKLQPEFIRQLGKYYCSNLIRFNFGHDAERARLEVNHDIVDTTQWKVRDLIGRGQVNAYTRLLAVSSLYFKGEWGSKFKPMQTKVSNFHLGISGRTIAVDMMQQRAEFRIAVCDEVDSTIVELPFSSQRISMYVFLPKDASGLALLESKLTPDVLTAIFAGMRRARVTVTLPRFRLSSCYCLQDSLAALGLANLFHPGEADLGAMAASSSTGDNSHGMHIGGLLLKTYVDVNEEGSEASSAAALCVTTATIRAEPEFTQYFVADHPFLFMIRDNLSGLILFVGRLVSPTK

>SmSRP-7

CNNKFALQLFKRICTHCSNILVSPLSVSSVLAMLLVGARGQTETQLKQILHLQEFSLTEDEDENANDDNANGNSQIHLAYRHLLNSITETGNTGLNAANRLYIQEDVAFDGSVSDYLAENYGADVGCADFARAGDEARAQINDWVSEKIGTDSWQQGLVGEGVVDLFTTLVAITATTFLGEWQFPFNEGATCLYPFNAGGGRKIVIDLMHTPSCCEFRTAFNDDLNCQLVELPYKEDRFAMLLIIPSEHEDLHVFEDKIDVDILDALVGQLEPSSIKIALPRFRVACTFSLSDIIGDMGAGLVLNPDSADLTGFTASNEEGVCLSALIHGTELRVIEKGTEYSPSTPQDSDESEDEGNEIVVDRPFIFILRDTKSGLILFTGCIHEPSTE

>SmSRP-8

QAAHANTQFGLELLKKVSVGNKNVFFSPYSISAAIAMTSLGAAGNTLTEIDHTFHFDTVNTNSDPKAIHKVYKGLIEDYNKPNSNYSLSTANRLFGSKNFEIKPTFQNETKYYYDAELQQVNFDGTAEGIINHWVENQTHDKIKDLFKAGSLTPDTALVLVNAIYFKGNWNSQFKKENTKDEDFHSADNTVSSVKMMNQKSRFNYYHDGTDLKCKVLEMPYVGKSLSMVILLPDTIDGLPNLVQALTAEKFEALLGKLFETQVDVKLPKFKLETEYSLKETLQQLGIHDLFSNSDLTGLSESSVQVSGVKHKAFVNTDEEGTEAAAATGVHLPGLPSLVPPTPQFFVDHSFLFAIVNNVNVLSETATSVSEVVHKAYID

>TuSRP-1

LNIVASNNQFGFKLFKALNKFHGNENILISPLSLFSTLVTVYAGSSASTEDEMISLLQLRTMTEAQIQTAFRDVLHSLLNDVGKKNSLKLLNAIFIDKDYNVSTSYVDKVRTYYNAYLEKVGFSTEPTYVVKWANELVSWWTQGLIPNLLDSLDPLTRLLLINVIYFKGVWSAPFNQALTNEQAVFRNFDQTKSIIPMMRSISSINYHCDYVNVQACVVEKLYSGGSISFLVITPTNGTDLKPLEAALGSQLMYELISQLQENTVELGLPRFSLSGSYDLLKPLEYLGMKAAFSPKKADLSKMGNSKELFVKEAKHKTVLQVTEEGTLAAGATVAEIGTRKRSPRIIIDRPFIFLIRDLKTDAILFLGRVNSL

>TuSRP-2

LALPINQFSLSFYRSVHNLSENVFFSPLSISMMYSMLLRGASGLTAQQIINTFQYPLSFKTSDEIHAAFKELVFDYKMIEIRSWQRNFTLKLGNLVLVDKAFPILADYADHLLNEYHASVAEEDFTAEGYQIMNKVNKWVASKTNNKMVKLFNQPFDYLTKLLLINVIYFEGNWKLPFDRSLTAARTFYNYDGSLGDVQMMSTTGSYRYAEFWHVNMKMLELPFQGDISLILILPVELTHQPGLTKFLNSLTTDQLNSMISSLTQQTVELSLPKFRLEGRYDLNKILPDMGLFLPFITNTDFFTISPSEGLKVTDSMHTSLIQISEEGSQHQTSGSVGSYKLPILARSRGPRFIVNHPFGFIIRDNAYGINLFLGVINKL

>TuSRP-3

PSNDFGLRFLEQINSNGSPKNVLFSPLSAIIAYGMLLEGATGETEQQIKTVLQLSSIGNQTSDISKASKKLLEAYKLVEDGTSDRNFSLAMGNLAMVNKNAKLKEAFAQSLMTNYFAQATNEDFSNGTAVMDKLNSWISEKTKNKINKILTNPPDEATILILVNTLYFKGRWEEPFPKGLTIDDTFTNGDGTLSKVKMMTLRDKRFNFVHNSDKKVKVVELPYIGNISMIFIMPTESNTLANLVPTLNATELDNLLSSMSQTKLRALSIPKFKLEDTHKLHEILPRMGMDLPFGDLAQLPNIAEKSELKVSQSIQKALLELDEEGSVAAAATLIVTVLRMSYFPAEDFVADRPFLFMIRDRLTGVNLFMGQMNKMAN

>TuSRP-4

GLHKFSIDILRSLHNFESKDSSPGLILSPFSIWSALLVSYMGARHETDRELRSVLGLNNVPKHAVGMAYQGLRFWYQLKRNVSLVTKETSKKQAYSIANKIFINDALTLNDCIKQHFATEAESMDFTSNPGGALKAINSWIEEETHGKIKDLIPPGSVTQWTTIIIANAIYFHAKWYNQFDASKTEIGTFHVTPVESIQIPFMKLTANLMYGVSEALRCTVLELPYANQDFSMLILLPDASKGVDSLVRQLKPSHLEDVVANMFDDEISVVLPKFKAEQELELSGPLYSMGIMKLFDPRFADLSGFFQPASANKNGKNKNTTHELQTKGITVNSVVHKVYVSVNEEGTEAAASTAILIARSGRPAFPTRFVVNRPFLFLIRDTATNVILFIGIVRRPYE

>TuSRP-5

PSIDFGLNFLKRISSQCDSKNILLSPLSVITAYCMVLDGAAGKTEQQIKKVLNLDKINDQSEDISGLIKQVIESYRIPATAGSRQKFILNFGNLLMANKDEKFQKAYVQNLKTNYFADAFNEDYKEGQQIVEKINSWVSNNTKNKITSIINEPPSPLDVLYLVNTIYFEGKWLMPFSKDCTKDDIFHNSDGTTVKTKMMTLSGESFNYVNRHDKQLKIVELPYVGNISMILILPTEDNNLKKVIDDLDSTELSSLMESMKKTYLDTLIIPKFKLEDIHQLHNILPKMGMTAPFQKDAEFPRITEEPLPLYISKSIQKAVIEVFEEGTIASAATVVEGLFMLACSPFSTPLITFIADRPFMFFIRDNKSGVNLFMGQLNNMS

>TuSRP-6

SIDFGLNFLKQISSQCDSKNILLSPLSVITGYCMVLDGAAGETEQQIKKVLNLDKINDQSEDISGLIKQVIESYLKPATAGSRQESILKFGNLLMANKDDKFQKAYVQSLKTNYFAEAFNEDFTFGRKIVEKVNSWVSNNTKNKITSILNEPPSPLDVLFLVNTIYFEGEWLKSFSKDRTKDDIFNNSDGTTIKTKMMTMSGEFFNYIVELPYVGNISMILILPTQDNNLKKMIDSLDSTELSSLMESMKKTRLDTLTIPKFKLEDIHQLHKILPNMGMTRPFKKADAEFPRITEESLPLYISQSIQKAVIEVFEKGTIATAATVVNIKLKGKTCSSFRMPLITFIADRPFMFFIRDNKSGVNLFMGQLSKMTRQQFL

>TuSRP-7

SIDFGLKFLKKVNSASDSTNLLLSPLSVIIAYCMVLEGAAGETEKQIKEVLDLDKIDNQHGGISEIIKQLLESYKTNKNFILEFSNLLVTNRKFKLKDTFVRSLQTNYSADAFNEDFTKNGKNVLEKVNSWVSENTKNKIATILNEPPNPDAVCLLLNAIYFEGSWLNPFSKRSTHDKIFNNSDGSTTKVKMMLHAGRYNFLESPEKKFKIPYGGNMSMILVLPIGDNNLKKMVDNLDSTELSRLIDSMTRTRLDSLMVPKFKLEDKHQLHEILPNMGMILPFDQGNAEFPKITERSVYISQSIQKALIEVTETGTVATAVTQLEAVLACAPPKVINFIADRPFLFFIRDNLTKVNLFMGQLNNMS

>TuSRP-8

QSHMQFTFDLMSSIVMTEAYNPNSKLLQSIVFSPLSIQSILMMVHLGVKGRTRAEIASALHLDTFLTSNGINNVNGSFIRTHQIFGESVNSLLDDDDIIKYFSMANQIFVNKDLTVNNNFKIALQRYHGASLRSVDFNSNGVVDKINDWVTKTTKGAIKKFLSSPISPATALVALNALSYKGDWLYKFDEQETQKNSLFQLTNGQNARVSMMVGKLPIAFGEINDGRLKASIIELPYKTQRLGLFMVLPLEDSPNGLFNLMRSLNSTTFTQLIASMKKSKKGDEVNVRIPKFDISSKPDLTTILRYSLGLRSVFSGGEADFTSMFETTPVSQSSSLPPISLSQFTHQAVMSINENGSIAGAASATLVERVGLFSGPYFEADRPFLFFLTDKQSGLILFAGIFAQPN

>TuSRP-9

GFGLKFLKKINSASDSTNLLLSPLSVIIAYCMVLEGAAGETEKQIKEVLNLDEIDNQHGGISEIIKQLLESYKTNKNFILEFDNLLVGNTKFKLKDTFVQSLQTNYSADAFNEDFTNNGKNVLEKVNSWVSENTKNKIATILNEPPKPDAVCLLLNAIYFEGSWLNPFPKRCTHDKIFNNSDGSTTKVKMMLHAGRYNFLECLEKKLKIPYGGNMSMILILPIGDNNLKKMVDNLDSTELSRLIDSMTRTRLDSLMIPKFKLEDKHQLHKILPSMGMILPFDQGNAEFPKITERLVYISQSIQKALIEVTETGTVATAVTQLEAVPCSAPSKVINFIADRPFLFFIRDNLTRVNLFMGQLNNMS

>TuSRP-10

NPSIEFGLKFLKKINSASDSTNLLLSPLSVIIAYCMVLEGAAGETEKQIKEVLDLDEIDNQHGGISEIIKQVIMHILKFGNLLVGNTIFKLKDTFVQSLQTNYSADAFNEDFNNNGKNVMEKVNSWVSENTKNKIATILNEPPNPDAVCLLLNAIYFEGSWLNPFPKRCTHDKIFNNSDGSTTKVKMMLHAGRYHFLESPEKKLKIVEIPYGENMSMILVLPIGDNNLKKMVDNLDSAELSRLIDSMTRTRLDSLMIPKFKLEDKHQLHEILPSMGMILPFDRMSAEFPKITERLVYISQSIQKALIEVTETGTVAAAVTKSACTSARAPSKVINFIADRPFLFFIRDNLTNVNLFMGQLNNMS

>TuSRP-11

LAYSTNQFGFDLLRAMDKSESSTAFCPICISSSLTMMLMGSQGHTSTALRHALYLWGMQTSEINLAYHDMMTHLGVNVPNSVHYRNLGPYGPSPASDYRVSIADNEANTGNDIAFLSHVYVQRDFGINYSYHMLLQRFYKTAIRPLDFIDNGEETRQHINAIVEKETSGKIKDILPDRQSPTTQLLLLSALYFKGSLDLNITSSRKRNYIAPSSSSSAYKQLSGASSNLLGVFSEDSIILEARNVRIRYGFNRFLNCTTIEMPFKGGLITLVAMMPHDPYGLDTLLTRLSAQVLSDVINSLEVRRVDVKIPRLHFETSDRNLSLSLANLGVAYIFKPGYSQLYDISDYKWLHVSDIIHKTYLEIWENPKTFTNTNINSINTNGNTINSNNVNNNNGKSQYQSSNNIEVVFDKPFLFFIMDNISGLILAMGKHGR

>TuSRP-12

SSIDFGLNFLKRISSQCDSKNVLLSPLSVITAYCMVLDGAAGKTEQQIKKVLNLDKINDQSEDISQLIKQVIESYRIPATAGSRQKFILNFGNLLMANKDEKFQEAYVQSLKTNYFADAFNEDFTDGRKIVEKVNSWVSNNTEKKITSILNEAPDSLDGLFLVNTIYFEGEWLKSFSKKRTRNKIFNNSDGTTVKTKMMTLIDERLNYVDRLDKQLKIVELPYVGNISMILILPTEDNNLKNLIDNLHSTELSSLMDSMKKTRLNNLTIPKFKLEDDHELHKILPSMGMTRPFQMNAEFPKITEGSLPSYISQSIQKAVIEVFEEGTIASVSTALRRSTGGVSPKRITFIADRPFMFFIRDNKSGVNLFMGQLSNIT

>TuSRP-13

SIDLGLNFLKKVGSDSTNLLLSPLSVIMAYGMALEGAAGETAKQIRTVLNLDKVDSQQGDVSKIIKQHMEKYLKNKNSTSEEKKCLLEFGNLLMANKDFRLQKTFVQNLQTNYSANAFNEDFTDGQNILEKVNSWVSAATRNKISTILDKPPEPDAVCLLVNTIYFQANWLKPFYEDSTEEKIFTNSDGSTTKIKMMTLSDDTFNFAECLEKKLKIVEIPYFGNISMVLILPTGDNNLKKMIDNLDSTELFSLIDSMSRTRLGTLTIPKFKLEDNHQLHKILPRMGMTRPFQMNAEFPRITEGSLPLYISKSIQKQQKVLNFIADPPFLFFISDNLPKVNLFMGQLNNIT

>TuSRP-14

SSIDFGLNFLKQNSSSQCDSTNVILSPLSVIIAYCMALDGAAGETEQQIKKVFNLDKINGQSEDISGLIKQVMNNRFFRLPTQESIRDSRSVFSLTDFGSYRIPATSRSSQEPILKFGNLLMANKDYKFQEAYVQSLKTNYFADAFNEDYKEGQKIVEKVNLWVSKNTKNKITSILDEPPSPVDVLFLVNTIYFEGKWLEPFPQHRTRDDIFNNSDGTKIKTKMMTLKFQDFNYVDRLDKQLKIVELPYVGNISMILILPTEDNNLKKVIDNLDSTELSSLMESMSMTNLDTLTIPKFKLEDKHELHNILPRMGMTRPFEMNAEFPRIIEEPRPLYITKSIQKAVIEVFEKGTIASAATVVGPMRTMASAPQRPLIEFIADRPFMFFIRDNKSGVNLFMGQLNNMTR

>TuSRP-15

SSIDFGLNFLKQSSSSQCDSKNILLSPLSVITAYCMVLDGAAGETEQQIKKVLNLDKINDQSEDISGLIKQVIESYLKPATAGSRQESILKFGNLLMANKDDKFQKAYVQSLKTNYFAEAFNEDFTFGRKIVEKVNSWVSNNTKNKITSILNEPPSPFDVLFLVNTIYFEGEWLKSFSQDRTKDDIFNNSDGTTIKTKMMTMSGEIFNYIVELPYVGNISMILILPTQDNNLKKMIDSLDSTELSSLMESMKKTRLNTLTIPKFKLEDIHQLHKILPNMGMTRPFKKADAEFPRITEESLPLYISQSIQKAVIEVFEKGTIATAATVVNIKLKGKTCSSFRMPLITFIADRPFMFFIRDNKSGVNLFMGQLSKM

>TuSRP-16

SIDLGLDFLKKVGSDSTNLLLSPLSVIIAYGMALEGAAGETEKQIRTGLNLDKVDSQQEDISKIIKQLMEKYLKNKNSASEKKCLLEFGNLLMANKDFRLRKTFVQNLQTNYSANAFNEDFTDGQNILEKVNSWVSEATRNKISTILDEPPEPDAVCLLVNTIYFEANWLKPFYEDSTEEKIFTNSDGSTTKIKMMTLSDDTFNFAECPEKKLKIVEIPYFGNISMVLILPTGDNNLKKMIDNLDSTELSSLMDSMSRTRLDTLTIPKFKLEDNHQLHKILPSMGMTRPFQMNAEFPRITEGSLPLYISKSIQKALIEVTEAGTVATAATQVEFMLGCSLYSPQPQKVFNFIADRPFLFSIRDNLTKVNLFMGQLNSI

>DpSRP-1

MQFSLNFFKKVFAATQSDVQSKSASENLFFSPMSIYSALLLAYFGANNRTEDQLTEILGLQNMDKVGAVQAYKLVKFTRQLMRVAGLVKYDFDIANRFYFNEDENIRPCIKDIFNEDIEMLNFAFQPAESRTRINQWVEDITRNKIKDLVTSDTINANTRIALVNAAYFKGQWASQFKVANTRLTSFAINNKEEGVANMMFQKGRFRHAAVEELQANLLEMPFLGGDVSFFALLPKGNNGLEETVSRLTLDTLRNAMASTFPLTVDVGIPKFRLEQTLSLRNVLVKMGLTDMFDSLAADFSGFNGVPGLKFDEATHKAFIEVNEEGSEAAAATALVAFRMARPLDGVRFICDHPFMFFIYDNLSESILFMGVYRNPK

>DpSRP-2

KAAAALQNFSVSLFQAVGKHHSPTENVFISPFSVAAVLSMVGVGARGNTAVQLKKSMGLTNYVAENGNSDSVIGSLIQSIKGDENFTLEAANQLYVAEKYQLTDDFKQNLNDNYGAAGQTVDFAVDASRTKINEWVEEFTQHKIKDLLPEGSVNSLTKLVLVNAVYFKGNWMRKFDSSLTAVEPFYLGSKDKQKNVNMMHIDAEFRTGYIESLDARLLELPYVGRKLSMFIVLPNKIDGLPELELKMHEASLDDSNVEMRSAKLHVAIPKFKLDADIKLKDILIKMGIADLFDANAADFSGISGEKDLFVSNIFHKSFIDVNEEGSEAAAATGSTCRKKRSLNFEEDQIVDPFIADHPFMWLLRDNDSGMWIFLGRYVDPI

>DpSRP-3

NDCATNTVFSPLSIASTLTMLLMGSSGNSYIQLRSALGYHNDANDVDINGAYKFLMERVKRMDVEAGSSILLSIANGLFSQKQSRFTDDYINKAKEYYQSEVNELDIIRNPYGSANVINRWVSDKTKGKITNILSSLPPDTQLVVANAVYFNANWADPFTPDVTRREDFHVSSSEILTPLTMHTHSMVAYIENEELGCKMIGMPYKGEELGMFILLPTEKQGLASLSRLEDKLTVEKLEHMFSRMEAKTVAISLPKFRIQQKLQLKNVLRGLGVTDLFSPSSADLSRMTSKTGVALDNIIHQTFIEVTESGTEAAAATVLNLSRDGPSKTFAANQPFLFIIRDIPSKAVLFFGRVVRPGDA

>DpSRP-4

KYDNLVLSPSSISLVLAMALIGAQGNTAKQIKEAFHVTNQNDETIACNIGALNRPTQGSGVTLSTVNRALVSDDFRLTEFFRSTLQNQFSATVENVNFSLPSTLEAINKQIEKLTNDKIRNLIPKESLGASTKLILLNAIYFKGNWLKAFDSTKTRVRPFYVSPNHKPIPTKMMASRNYFRTAFIKEANLRALELPYSGSQFSMVILLPNQLNGLTHLESSLSPKLLSLIDSKLNKIQMDVVIPKFKLEFSPDLKSVLRSVGIVDLFNTDADLSKISGSKELFVSDAFHRTIIEVNEKGTEAAAATAFRFIARSFRRTPFIPKFVADHPFIFLIRDNRKNTIRFVGRFCKPEKN

>DpSRP-5

FSRSHFAFSLDLYSALAGQSPANSEGEAGNLLFSPYSVSTALSMIFLGAGAGSTTSLQLRSALHLNNFSFSDVHDSYKTVINKLSDPYYAEILVTMNGIFQQEGIFVSEKYKRALEEFYNVQIQPMDFVRHPQLAVDNINSWARNFTKQKISHSLRKTAAPASVHQELGITLANGLAFRSHWLFRFDPASTFDKGLFYTTSKKRFEIPMMVGRFKIPVGYSSDLECRIAELPFSSRRVSFFIILPDDVDRGITKLEANMTSDNIKALFSTLKDETVNIRLPRFRLEQQEIELTKTLAALGIHDVFDNEEADLSGISSEKLHLNHVIHKTFLEVEEDGMAESTTSGLNRLGAFGEKYFEVDHPFIFFLWDYHSGILLFIGRITTPEP

**D. I8 Ascaris.**

>ApTLI-1a

QCSGGKVYSQCLPVVQETCTIAAEVKSKNINSYCEEGCVCPFGTVLNDGVCVVKEKCPCKLR

>ApTLI-1b

VQCPAGQNYDTCGNSCQRTCQDISTINNCKSNCVEGCYCPKGLTLSEFGECIPVAECP

>ApTLI-1c

CSRRKHLTFTECEPSEPITCRNMHDPPQSTPAICYSGCVCKKPYVLDSFTKECVLPNECP

>ApTLI-1d

VNCPTPLVYKECFNKVCEPTCDSLLQSDPCPKLPGFCFPGCYCPEGFIKELGSCIKPSKCRN

>ApTLI-1e

SCPKGLFYKACSSGCEETCDNYKALRSGESLCKNLPTEMCTCPTGQVFNNSICVNENRCEPCD

>DmTLI-1

RCPANETFLACGPDCQTECATLGKPCLVRHIRCPDGCYCNKGFARNAAGTCIPLRRCNEGGYGN

>DmTLI-2

DCSVNGTQTDCPTACPETCDTKGKPNCTLICGGPCVCKPGYVVNRMIPACVLRSDCPK

>DmTLI-3a

SCGFGRVYQACGPNVEPTCDSDLALPASKGACNEGCFCPEGTVQYKEACITRELCP

>DmTLI-3b

KCPLGQVFDECGDGCALSCDDLPSKGSCKRECVEGCRCPHGEYVNEDGECVPKKMCH

>DmTLI-3c

CAKQPYAEFTKCAPKEPKTCKNMDKYVADSSDCLPGCVCMEGYVYDTSRLACVLPANC

>DmTLI-3d

CPSPLVHTDCYKRRCEPSCDNVHGDDCPVLPDACFPGCYCPEGTVRKGPNCVPISECK

>DmTLI-4

CTANGTQTECPVACPETCEYSGNGPCVKMCGAPCVCKPGYVINERIPACVLRSDCP

>DmTLI-5

FCGENATMVRCAGVCPETCAFKSLKCPKYCGVNCVCKPDYVFNENLQLCILKTDCP

>SmTLI-1a

SCSENKVYQDCGSDCQASCSNPIRKCKSEWCNDGCFCTKGMLLGPKGKCYQPSDCP

>SmTLI-1b

PCPSGQVFSQCSSSCSRSCVDIASNSKCEEQCVEGCNCPLGMTLDSDGECIPVSECS

>SmTLI-1c

CLDADNMEPTDCLVECPLTCSNFHKPPICQTVRCKPGCKCKDGYIFDSKAKTCIKPSDCPC

>SmTLI-1d

QCGEGMYYDQCGPACMKTCKNIDEHEKTENCEADPVEGCFCIGDRVLKDGVCVDPMECKK

>SmTLI-2

TCPRGAVYTNCAPSCAQTCFDAPQNTELCSKPCAPGCLCPPKTVLHRGRCIKPHRYK

>TuTLI-1

WTDDIDDPCPTDRVYNPCGSACPLTCAQPKKGKCNRMCVPGCFCKPGLYENAAGNCVTLDECY

>TuTLI-2

KLERCSSERQEFNECGTACPPTCYLPNPEFCTRECVARCFCKEGYFEALDGQCYTEQECVAIINAP

>TuTLI-3a

TEQCEKPKVFLECGSACPPTCAKPKPTVCTLQCVKGCFCPKGTFLNDSGKCVEKCSSDGN

>TuTLI-3b

PWCPNSKVYTECGSNCPPTCANPKPGPCGSACVKGCFCPIGTFENAAGECVKQCSSNGS

>TuTLI-3c

DGFIPKVNNDCGSACPPTCANPNPVCTKQCVKGAFCPEGTFENASGDCVKTC

>TuTLI-4a

CVEKCTKPKVFSTCGSACPPTCDNPNPICTLQCVKGCFCPKGTLLNRSGECVKNCPGSG

>TuTLI-4b

STPSCPSPKIYNKGGSARTPTCANPKPEVCTAVCVEGCFCPEGTLLNGSGECVKNCS

>TuTLI-4c

SCPNPKIYNKCGSACAPTCAAPKPEACAQVCVEGCFCPEGTLLNSSGECVKNCSSNGDNSS

>TuTLI-5a

FTNTSKVFTDCGSLCPPTCAKPKPGICATVCVEGCFCPKGTIENAAGKCVKQCSKSKV

>TuTLI-5b

CSKSKVFNSCGSPCPPTCANPNPGFCGTACVRGCFCPKGTLENAYGECVEQCSSNG

>TuTLI-5c

DYIPKVYSTCGSACPPTCANPNPICSQQCVEGCFCPKGTIEDASGKCVKQC

>TuTLI-5d

CSKSKVYNECGSLCPPTCANPKPGPCAAVCVKGCFCPKGTFENAAGECVEQCLTNGSS

>TuTLI-6

PPTAAKVYNSCGSACPPTCANPEPKFCILVCKADWFCPKDYYLNSAGNCVLKEYCDNV

>TuTLI-7a

PRKVQKRIIGCGTACPRTCSNPKIDSCIQVCTGNPECPEGYFENNIGKCVLWKDF

>TuTLI-7b

KPRKVEKVMIGCGSACPLTCKYPEPRMCIQVCTGLPECPRGYYENHLGECVLREDC

>TuTLI-8

RTATKVWNPCGTNCPLTGANPKPRACIKSCKADYFCPSGYLENSIGQCVIEEDCDLQ

>TuTLI-9a

EQCEKPKVFLECGSLCPPTCAKPKPEACAAVCVKGCFCPKGTFLTDSGKCVEKCSSDG

>TuTLI-9b

CPNSKVYTDCGSACPPTCANPNPGPCAAVCVKGCFCPKGTLENASGECVKQCSSNGS

>DpTLI-1a

CGVHCRGGQTYQVCANSCARTCYDIALYPKCRRKCVEGCNCPEGQTLDPFGLCIPIHECPCI

>DpTLI-1b

CRSSHNEEYTPCEPEHQLTCKTMGQQVSMKKPVECRPGCQCKKGYIFDPTSKTCIKPSECPCH

>DpTLI-1c

CAIDCGTDGSVYKECNKKKCERSCQNNKNPHPCPPMPDLCYPGCVCPDGLVRHSDGKCVKPSECRDC

>DpTLI-1d

CPIECPSGMEHQQCGSGCQSICGQDKDLTFTCPMSVNDGCYCPTGHAFNQELGRCVPQDHCEPCD

**E. I17 WAP.**

>ApWAP-1

NKPGECPVGDLDSVASLDRPCLEQCNSDDFCHAEYKCCRHSCGITCQQPV

>AgWAP-1

GDECPLASKVGSCSPTCLTDRDCADIGGKCCSNACNRKSCVER

>AgWAP-2a

EKPGSCPRANEIESTDNAGFLCGTPCSHDLECPQMQKCCQSDGCGRNCQQPHN

>AgWAP-2b

PKKPGQCPFLVPPGSENSESDSCEYECRTDAHCDGSKRCCSNGCGTQCVEPQ

>AgWAP-3

YKKYGDCPPKPPSKLDSLCLNTCDGLDYKCPGVEKCCEHSCGHSCQSPY

>BmWAP-1

EHKPGKCPVSDTPKWEAACVQACNSDSQCDGTQRCCHHGCGSTCSEPL

>BmWAP-2a

LQKAGTCPEPATKDLDCSTPCSHDLECPSMQKCCDGGECGRHCVLPHN

>BmWAP-2b

KKSGQCPYLVPQSGACEWSCRSDAECSGGERCCATGCGTACTQPV

>BmWAP-3

VKKGNCPEFPRGPWICSHTCTGDSDCPRALKCCHNRCGVLTCQKPEI

>BmWAP-4

AAGTCPLPSKVYGCSPKCKEDYECTHGKVCCSNSCNAKSCSEPAAY

>CfWAP-1a

AKAGSCPPALPVQFCGRSCYVDAHCAGIGKCCPTRCGGSICSMP

>CfWAP-1b

EKPGSCPSVPTGRWVCSSTCNSDNDCRGSLKCCKNRCGALACQKPM

>CfWAP-2

KPGYCPERTSMTPFEAACLDACVDDSRCPDLAKCCPHDCGITCMHPVG

>CfWAP-3

SGNCPLRNTVTNCTPRCMNDEQCPFNQKCCPNKCSSTSCAQSS

>DmWAP-1a

FQKPGICPAPDHSQYTERTGYMCGSPCSHDLECRNMEKCCFTKGCQFNCQQPGNVT

>DmWAP-1b

PRKPGQCPYLVPPGPDNLDANTCAYECRTDAHCDGARRCCSNGCGTQCVDPQL

>DmWAP-2

GPRAGSCPKIGRQSRARLSCLDNCQYDHECPEVQKCCPSSCGPMCVEPLG

>NvWAP-1a

KRAGSCPADEPEHCGSPCSHDLDCPGPQKCCASDKCAASVCVAPK

>NvWAP-1b

IKPGQCPYLVPSSSSCEVLCSTDQECTAGDKCCSTGCGTQCVSPV

>PhWAP-1

DKFGYCPDSFDNIPVFVQACVFFCTDDRDCPSTEKCCLHSCGQTCKPAL

>RpWAP-1

ALEGDCPALNGEISKNACLAETCTSDLDCGTKAMCCSNGCVLTCIEVD

>RpWAP-2a

LTCPPPTLIPVCRITCKTDTECADLENEMCCPTACGGSLCQRA

>RpWAP-2b

GLKPGTCPKSPSGPWICSNMCSTDSDCHRKDKCCPNRCGAMVCQKP

>SmWAP-1a

CPTLIDAHPELCPPTSSMNKMKMCVSDSTCNGTDKCCFDGCTNICTQPI

>SmWAP-1b

EKPGKCPVDAISSECGDKCSDDSSCMGLLKCCSSNECGSHCVPS

>SmWAP-1c

HKPGICPQSDVCVNDSTTCVRDDDCREDEKCCPHTCGSSCAKPLR

>SmWAP-1d

PKSGACPVIADDEIGACAEECKNDVDCPGPQKCCLNGCGAHVCRDPA

>SmWAP-1e

GKRGQCPYWVLSTIEKCDSECKSDYDCAGESKCCSNGCGLSCVDRVNKT

>SmWAP-2a

FDCPDQDKIQNGTSCLLDSECPGEEKCCPKSPCSYTCTAPV

>SmWAP-2b

TNREGKCPPSTSIIVDGGSRCMLDTNCPGSQRCCATPLGCLICVQPNIT

>SmWAP-2c

CSDPFSTVTASCPTPGNMQLTQPCLIDQDCSSGSKCCAFTNCSYFCAQA

>SmWAP-3a

CNSPKYGSCPSEGLINPSFYPCVTDRECSGGRLCCGSNYANT

>SmWAP-3b

NKPDGLCPRVELYTNPTIYCSSNDQCPGNWVCCYGKAGKTCSRPQL

>SmWAP-4

SSKPGSCPAMGLFGPFCASCTADTQCPGMQKCCPAGNSGRNCCS

>SmWAP-5

RKPGDCPKIFVMNPWCPVCSEDSQCPGIQKCCPDSGKKCCFA

>SmWAP-6

GKRGFCPYPIPEIQTTGIEAILCSSDRNCPGNLKCCPIIQRGSGKT

>SmWAP-7

PKPGTCPAMGLYSPFCASCTVDTDCLGIQKCCPAGFSGRNCC

>TcWAP-1a

TKSGDCPPYPNVGICEVACFEDNHCAGHFKCCRTACGGTFCTAPV

>TcWAP-1b

GEKQGSCPVAPSGPWVCSSRCALDSDCRGAKKCCRNRCGAMACTKPE

>TcWAP-2a

IQKTGTCPEKIDAGDECGQMCSHDLECPSVQKCCQTQQCGASCTHPKNV

>TcWAP-2b

PKKQGQCPYLVPATSTSCDFECNSDMACNGTMRCCSNGCGTQCVEPLL

>TcWAP-3

SEKPGDCPPSLPPPACIISSLKLCETDEGCFGPMKCCKNDCGGAICLPV

>TcWAP-4

FNKPGRCPQNEKTLSPFDAVCLKTCTQDNQCSNLKKCCRHSCGVTCQHPE

>TcWAP-5

SSNCPTASRIDSCSPKCKDNSNCHGAQVCCTNICGTKSCTDI

>AmWAP-1a

EKPGSCPPPLPVDICSQSCFSDSHCLGIGKCCPTNCGGFVCTKPV

>AmWAP-1b

EKPGSCPAIPKGRWICSSTCSVDSDCRSTMKCCKNRCGAMACQKPD

>AmWAP-2

YKEGHCPLRNSVSKCIPRCVSDYQCSFNEKCCPNKCGSESCVQAS

>AmWAP-3

TKPGYCPDKASMTPFEAVCLIACVDDSRCPDLTKCCRHDCGVTCMHPIG

>AmWAP-4a

VGRCPAFEEQNVCPARAPTCENDFQCQSAGERCCKTACGTKCVNGEL

>AmWAP-4b

KRPGTCPLEEAAICGNTCQHDLECPGPEKCCKSEKCGGSVCSVPQG

>AmWAP-4c

ATKPGQCPYLVPSSSSCELQCSNDQECSATEKCCSTGCGTQCVAPVM

>DpWAP-1a

KGTGRCPSKIRDPDRCPPVSSATDEANKRTDECSSDDDCHGTSKCCTDGCRRLCVMPLLTS

>DpWAP-1b

ITKPGSCPALDGQTNSTDCGVPCSNDMECQGADKCCPSLGGCPAGAGQHCVPPFNF

>DpWAP-1c

VIKAGQCPYLVPISVDSCDSECSADEDCDGQLKCCSNGCGTQCVEPLIK

>DpWAP-1d

IKPGFCPPVRSRTFLRVLAQFAGGAACADQCVSDADCAGPTRCCPGECGSTCTHPVLL

>DpWAP-1e

LPKPGACPAAAHPFGCPADTKAVIECSSDSDCSGRAKCCSNGCSSTCTSPEEN

### **F. I19 Pacifastin.**

>ApPac-1

DHGSTFEMDCNTCNCFAGEITCTKKHCEPATV

>AgPac-1a

CEEKCEPGTTFMEDCNKCRCGPDGQKACTRKMCPPNELSDDSQVR

>AgPac-1b

GQVCSPNEIKMKDCNRCRCANNGIGWFCTRRACPQRAKRSEPAPE

>AgPac-1c

KKCTPGTTFRSDDGCNTCFCTETGHAACTLKACLPPG

>AgPac-1d

PGFSCTPRSSFKYQCNTCLCSDDGKMAGCTFKFCVPGEW

>AmPac-1

MCVPGKSFFDGCNTCTCTDDGNFICTMTACEDYDPETDTSV

>BmPac-1a

ITCKPNQEFKSDCNLCKCSQSGHSYTCTHNECLEGDTGTDADV

>BmPac-1b

HSVCKPRNSFYVSCNICRCNDFGTDYACTNKLCPLPA

>BmPac-2a

PKECKPNETFQIGCNRCRCNSEGTLYSCTRIGCLESEEKNHT

>BmPac-2b

VKTCQPGQEFRLDCNKCLCDKEGKDFSCTRMDCNALNS

>BmPac-2c

ATCVPGSVYNQGCNVCRCTDEGRHATCTLMRCPQEKEETH

>BmPac-2d

FRCNPGEQFTRDCNDCTCSADGKSVFCTLRLCDQDITPH

>BmPac-3a

ACLPNSYAIIDCNICYCNSNGEIDEERCTRNICDPREDSRRSS

>BmPac-3b

GYCEPQHVYKKDCNVCKCQSNGQIMTCTTRVCNSLSV

>BmPac-4a

RCQPGTSFQRDCNTCVCLDNGLGLCSLDACRRSSTPKKF

>BmPac-4b

GRECAPGSSWSNQCNSCRCNADGYGICSDEACTEHII

>BmPac-4c

KECAPKTMWKNECNTCWCTSDGKPMCTRMECITNNTPEKSE

>BmPac-4d

RECAPGSTWSNQCNSCRCNADGYAICSDEACAEHINEPKKD

>BmPac-4e

CVPNTTWKNECYTCWCTSDGKPMCTRVECITNNTPKKSEL

>BmPac-4f

RDCAPGSTWSNQCNSCRCNADGYAICSDEACAEHIDEP

>BmPac-4g

DCAPKTMWKNECNTCWCTSDGKPMCTKMGCISYNNFG

>CfPac-1

RKCVQGKYYFDGCNKCFCGYNGIGACTRRFCDPSVTIPP

>NvPac-1a

FHCTPGSTFQMDCNSCTCSNDGKTAMCTGIACIQENKSDVT

>NvPac-1b

EFHCTPGSNFHQDCNSCICLKDGQSAMCTGIACPTKVKRDLE

>NvPac-1c

QVCVPKSKFNDYCNTCGCSDDGSSFICTRRLCDPEVWNKDGTM

>NvPac-1d

HKCKPRHLFKKDCNHCVCNAGGETAQCTVLDCSKLDL

>NvPac-2a

IFSCLPGSVFLQDCNACTCSNDGLSAACTDMACPGDLN

>NvPac-2b

KVCEPSTVFKVYCNTCGCSSDGSSFSCTRMACNQDIWNVDGSL

>NvPac-2c

EKVCEPRTQFKEYCNTCGCADDGLSYICTRRMCDENIWNKDGS

>NvPac-2d

ICKPHSNFKDYCNTCFCNNDGSEFACTRMSCPPEVWNKDGSL

>NvPac-2e

VCEPRSHFKDYCNTCACSEDGTTYGCTMMMCDESVWNKDGTR

>NvPac-3a

FCTPGSYFKKDCNMCSCSMDGKTAACTDMLCPNEMKNY

>NvPac-3b

YCTPGKMFSPDNCNICKCSADGLKAMCTLKLCSDD

>NvPac-3c

KQVCEPLTQFKDYCNTCFCSNDGLSFACTRMMCDHAIWNKDGSM

>NvPac-3d

KVCQAGSRFNDYCNTCFCNNDGTDFACTRMHCDENIWNKDGSM

>NvPac-3e

KVCQAGTKFSDYCNTCFCNEDGTSFACTRMMCDKNLWNKDGS

>NvPac-3f

PRVCEPNTHFMDYCNICACSEDGTTYGCTMMNCDQNVWNKDGS

>NvPac-3g

TRVCEPNTHFNEYCNTCACSADGMNKACTMMNCDLSIWNKDGSR

>NvPac-3h

RVCQPNTQFKEYCNTCACSADGTNKACTMMDCDLDMWNKDGS

>NvPac-3i

KVCQPGKAFSPDGCNTCVCNEYGTQLACTSKLCMTTLKQAY

>NvPac-3j

ACTPGKSFYSECNRCVCLETGNHAFCTLMDCAAL

>NvPac-4a

PLCEPGERFKLDDCSSCICNAAGTTAECTLGFCDNFKTRMA

>NvPac-4b

YCEPGRMFSPDNCNLCKCSNDGTKAVCTMKLCEARKARSAN

>NvPac-4c

YCEAGRMFSPDNCNLCKCSNDGTKAMCTQKLCQETEVT

>NvPac-4d

QVCQPLSQFKDYCNTCTCSEDGSSYACTRMYCDKDIWNRDGS

>NvPac-4e

ERVCQPGKAYSPDGCNTCVCNRYGTGQACTSKLCLSNLKA

>NvPac-4f

ACIPGRAFYSECNECVCTRSGRSAFCTLMSCPTSP

>NvPac-5a

ENCFPGAVFQDDCNGCICGSDGKATCTNMDCNML

>NvPac-5b

LQCVPGSELIHRCNQCFCTDSGTAMMCFKMGCGA

>NvPac-5c

EKCNPGMIFASDCNVCICSKNGKGVCTTFSCDTTYRF

>NvPac-6a

QVCTPGTYFKTECNTCVCAKDGSASICTQKQCPPGLF

>NvPac-6b

MQCKPKTRFKFYCNTCWCSEEGTTRICTKKYCPDNIFNKDGSL

>NvPac-7a

TGKCTPGQVFFMSCNLCKCSSDGNYAACTFMQCFDFNF

>NvPac-7b

QCPSKSFYNDCNMCVCGPDDASAACTMMMCMPGETQQPS

>NvPac-7c

CPAGEFFHDKCNVCHCSANGFSAACTLMGCPSEDTTQPR

>NvPac-8a

CPAPVFSNGCNTCVCSKVGVNAACTLKACLDVD

>NvPac-8b

KCPAQQFYDDCNRCVCSADGHSAACTRLACPPHRV

>NvPac-9a

GCPAKEFYLDCNMCNCGDSNEPACTYKACPQPP

>NvPac-9b

KSCPAGQHFYWKCNDCSCEENGREASCTRNFCPDFG

>NvPac-10

NECPPNESFMDKCNYCRCGPEGKDAACTKMNCP

>NvPac-11

KQCVPGKSYFDGCNTCFCSEAHSVQCTRRLCPDPWKR

>NvPac-12

TNKCPANQPFKWNCNYCTCGPEGKDASCTRMACPQH

>PhPac-1

ERCEPGQSFAKECNTCTCPDSGLKSLAGCTLKLCL

>PhPac-2

CIPGSIFKKGCEMCSCSPDGQILSCVPISCKNLK

>PhPac-3

DSCTPGETFKKLCNDCTCPPTGHKSAATCTLLTCGEE

>RpPac-1a

KQCTPGTTWKEDCNTCFCSSTGQIGCTLMACHHYQLPTK

>RpPac-1b

GKNCEIGTTVKLDCNICHCTAMGLACTRRLCHGQEL

>RpPac-2a

TKSCEVGTTWKEECHSCFCTKEGKVSCSKEACPPQLVPK

>RpPac-2b

KDCKEGESWQEDCNKCYCSHDGKPVCTRMLCPSVL

>RpPac-2c

AGCVEGEVWKEDCHTCHCTMGRKACTRELCLSES

>RpPac-2d

NCPPNQQTWEEDCNQCYCERGKKVCTKALCPDQG

>RpPac-3a

EQCGNVGEQFPSEDGCNTCSCDEGGAVVCTEKSCLLQ

>RpPac-3b

ECIPGTKVPSGDGCNDCTCTDEGNIGPCTLKACPGI

>RpPac-3c

CPSCEPGTSVTAPDGCNSCFCQDDGTIGGCTKMACPPKSC

>RpPac-4

RCAPGVTLDAGDGCNKCICSEKGVVADCTRMACPGVVQSEV

>RpPac-5a

CTPGKRFLSEDGCNWCVCNRDGSNAACTLMLCPAKRDRLSK

>RpPac-5b

NNCPPGKKFLAEDGCSWCICGPEGTSPVCTLTLCPPEKVF

>RpPac-5c

QECIPGQITPANDGCNFCICNKDGQIGGCTKKLCLNGIEKAPE

>RpPac-6

CRICLPGKHFMPDGDCNICKCSDDGMSALGCTNEKCTEVRSG

>RpPac-7

EHGSTFFMECNACSCYAGEIICSKRQCPKSSLSIT

>TcPac-1a

TEQCKVGDTKFKDCNFCKCTNGAFECTEKKCPDRGKRGVP

>TcPac-1b

PCAPNDYFKIDCNTCYCNIEKTGYLCTENLCPLTEP

>TcPac-1c

DFSCTPGQTFKKDCNTCTCTPDGKNAVCTLKKCAEAVANA

>TcPac-1d

EAECNNGDTKKVDCNSCRCTNGLWSCTKKVCLERKTRNT

>TcPac-1e

VCQPGTTFKKDCNTCVCNKDGTNAACTLKACL

>TcPac-2

ICKPLSKFKIDCNTCRCSGDGRQYSCTEMKCPPLG

**G. I21 7B2.**

### >Ap7B2-1

### EREPTGADYDDAASDGPNAVPSIRDNEYVQHGTLWGAQYMSGGAGEGVQTLNPDSPTRNKNVKTDAALPAYCNPPNPCPVGYTDEDGCIMDFENTAAFSRDYQESQECMCDSEHMFDCARSNSGTKHSVDVDELVRTFQVDDEHKSLVAKKFHVKKAFNPYLQGEFLPIAAKKGINI

### >Ag7B2-1

### LRELVDRMGKDLAEAADSYIDPSAMDELPASRLALMARVTKDLESEQLDYDALLDGSNPNPSPRDQEYLQHSSLWGHQYVSGGAGEGPNRPKPQVKTDASLPAYCNPPNPCPVGYTEDQGCTMDFENTAAFSREYQAAQDCMCDAEHMFNCPAAAQSESNPQMDSDLENFIARQFHTQEHKNLVAKKFHVKKSYNPFLQGEKLPVAAKKGFNVN

### >Bm7B2-1

### REVVERMGKDFNDAASSYLEFPASDRHLALMAHASKDLENEQLDYDSLIDGNPSPSLRDQEYLQHSSLWGHQYVTGGAGEGEQRLRPSGVVPNRQMVKTDAVLPAYCNPPNPCPVGYTEDQGCISEFENTAAFSREYQLSQRCMCDGEHMFSCPSDSTSDIDLRFPEHHKNLVAKKYKPDMENPYLMGERLPIAAKKGFDVS

### >Cf7B2-1

### YKVIDQMEKELVDTADTYLEYPEKVKELPIELPADYDGMDTLNPNPSIRDQEYLQHSTLWSHQRNNNNNNNNNNNKNKSNDRHRIQPTGLKGIKDEKAENPLPAYCTPPNPCPVGYTSENNCLTNFENVATFSRDFQNAQDCMCDTEHMLECSGDTGNSNSLSNVQISDSDFDEIVEQFQEENPFFRGEKLPIAAKKGIHVI

### >Dm7B2-1

### VLMTDLLNRMDKDMQVGYYDVGNEAAAGSKDNVDLVSRSEYARLCDGGSDCILQSGSASGAASHPSLRDDEFLQHSSLWGHQFISGGMGEGPNRYPTIVKNDAGLPAYCNPPNPCPEGYDMETQGGSCIVDFENTAIFSREFQAAQDCTCDNEHMFDCSEQDSADVGGDKGDLNSAVEQYIMQMGQENSLNNVNSLAKKAGYPVMPDPRLDDAVINPFLQGDRLPIAAKKGNLLFH

### >Is7B2-1

### ILGGPSLRDQEYLQHSSLWGHQYVAGGAGEGYQRLKPDGSGKNIQVVKTDAVLPAYCNPPNPCPKGYTG

### >Nv7B2-1

### RDLIERMGSELADAAGDNYLDERESASSGMRGLPDKEIPLEMPIDYEAIDAINPKASIRDQEYLQHSTLWSHQQLNNYKTNDRHRIKPGAQAASKNSEKGEKSPENQLPAYCTPPNPCPVGYTSENHCIENFENTAAFSRDYQSAQDCMCDSEHMLDCPSSADTDNNVVPGMPITNADFDQIVERFQEENPFFRGEKLPIAAKKGINVG

### >Ph7B2-1

### FRDVVDRMGKDLAEAADSYLDMPDNERLQTNGFLSNRVIKDLENEETSDPIDYDRLSNNPNPSLRDREYLQHSSLWGHQFVAGGAGEGKQRLKPDGSVQNQQQVKTDETLPAYCNPPNPCPIGYTGEDGCLEEFENTAAFSRDYQTSQDCMCDSEHMFECPVRDFQSHGNDLNEEDLDLDRIMEELTEGEQHKNLVAKKFHTKKDVNPYLNGEKLPVAAKKGINLSN

### >Rp7B2-1

### DKATSSLQNINGNSESQQTGSKQDNLPAYCNPPNPCPIGYSVEDGCLEDFENTAAFSREYQARQDCMCDSEHMFDCTNPRNSDGITQSDLDRLVQQFHVEDEHKTLVAKKYHEKKSTNPYLLGEKLPVAAKKGINIG

### >Sm7B2-1

### LREVVAKMGEATNDYLELPSDNVVTDDESDRSRVPKEFDNDRQFQPDYDTLDTMIRDQEYLQHSSLWGHQYMAGKPTFVLPAYCNPPNPCPKGYTHEDGCLENFVNSAAFSRNYQAAQECMCDTEHMFDCPTSTRDSEISALAQSIQNEGVMSHALDKIMEEFDVAHNEHKNMVAKKHYQPEPRENFFHEFLKFRNEPTKVNPYLQGEKLPIVAKKAPKF

### >Tu7B2-1

### EGTLSKIPRAKFQTRMDDILLGREPSLRDAEYLEHSSLFGHKYVQGGAGEGRQLLKPDGSVENYQVIKSDTILPAYCDPPNPCPIGYTTDDGCLESFENSASFSRDYQSSQKCMCDREHMFDCSGNTEENQLDALARSIQNEVISDSDLDDLVDRMQEGHKVVAKKFHASKKRNPDYLLGEKLPIVAKKAPHLA

### >Tc7B2-1

### EMEEEPLFPLDYEALGEPNIHPSIRDQEFLEHSSLYGSQFMSGGAGEGKQRLRPQGTIQNIQEIKSDSTLPAYCNPPNPCPVGYTAEQGCIEKFENTASYSRRYQAAQDCMCDTEHMFECPNPNPGDSDDDSFNDLEFNQFLQHTMQMNPGLQHKNLVAKKFHQYARTSGRLNPFLSGERLPVAAKKGNNVVF

### >Am7B2-1

### LRELINQMGNELIDTADSYLEYQDKPKEIPLELPTDYDSMDTLNPNPSIRDQEYLQHSSLWSHQHITNNDKVNDRQRIKPGSVKNIKNEKENALPAYCTPPNPCPVGYTSKNNCIVNFENTAAFSRDYQSAQDCMCDTEHMLDCSVDSTNSNNLPNMHISNSNFNQIVDQFQVEENPFFRGEKLPIAAKKGLNV

### >Dp7B2-1

### YEEAPESLNEPRERLGLGLSLRDQEYLKHSSLFSRQQEDDQPPSGTPTGSVKVSTPSSSSGKTENVLPAYCNPPNPCPIGYTAEDGCLEEFENTAAFSREYQAAQDCMCDTEHMFDCPASSLNHKNDKNRATANMVDTAIRKIMSDFQGEHKSLVSKKFFADKSENPYLQGDKLPIAAKKGNRVVA

### **H. I25 Cystatin.**

>SmCPI-1

MSAIGGTGAMKEATDEIQMICKSVKDAVESKLNKTFSEFQATSFKSQVVAGVNYFIKVHVGAGEHLHLRVYKPLPGQGELSLHSVQEGKT

>SmCPI-2

VGGWIDIQEDSEEILNVADRALARIEAQSNALFKQRISHVTNAKRKVVAGLMYEFKMKTEYTSCRKDLFVDYSQCSVNTEHEAQICFVNTWEKKWE

>RpCPI-1

GEPKEASVESEEIKAAANFAAERIDQMSNSIYKQILVRILEATSQVAAGIKMDLKLELGNTECMKNMDKKANCEVVSENAEKMICRVSVWSQPWKQSSGKSHLKLSKFYC

>RpCPI-2

GGESSLDTNDKRVTDLTDYVEDELTNRSNSQYTKTIVKVLNATVQVVSGKLTRLTVEVTDTNCLKSENKLKSLCSASNQ

>IsCPI-1

GGYSERANHQANPEFLNLAHYATSTWSAQQPGKTHFDTVAEVLKVETQVVAGTNYRLTLKVAESTCELTSTYNKDTCLPKADAAHRTCTTVVFESLQGDKSVSSFEC

>IsCPI-2

GGYRERSNQDDPEYLELAHYATSTWSAQQPGKTHFDTVVEVLKVETQTVAGTNYRLTLKVAESTCELTSTYNKDTCQANANAAQRTCTTVIYRNLQGEKSISSFEC

>IsCPI-3

GGYSLKTDHHTNPKYLDLAHFATSSWSAQQPGKTHFDTVEEVERVETQVVAGTNYRMTLKVVESVCELTSTYSKEACTAKANAAHRNCITVIYENLQGEKSVSSFDC

>IsCPI-4

GGYTRKTDHQTNPKYLELAHFATSSWSAGQANKAYYDTVEEVLEAQTQVVAGINYKLTLKVAESVCEITSQYTKEACTPKPDAVRKTCTTVIYEKVWENMKSVSSFSC

>IsCPI-5

GGWRSRDVYSDPAYAELAHYAVSSQAGDSEFYDTVLELLEVETQVVAGMNYRLKFSTAETACKVGVDEYSRERCLPKVNLPKATCTAVVYERPWQNHREVTSYEC

>IsCPI-6

GVWRKHHPDVDPRYKEWAHFAISSQVEDRTNFDTLMTLISVESQVIAGVDYKLKMKVAESNCVIGVDSYSRERCHLKVDAPYMICTALVNYMPWEHKTSLKSYNC

>IsCPI-7

GVWIKHQPDMDPRYKEWAHFAISSQVEDRTNFDTLMNLMSVESQVIVGVDYKLKMKVAESDCVIGVDSYSRERCYLKVDVPYMLCTAVVNYRPWEHKASLKSYNC

>IsCPI-8

GVWRRHHPDVDHRYKEWAHFAISSQVEDRTNFDTLMTLISVESQVIAGVDYKLKMKVAESDCVIGVDLYSRERCHLKVDVPYMICTAVVNYRPWEHKASLKSYNC

>IsCPI-9

GGWEKRDPHENPHFQELAHYAVSKGSKPKRYYDTVVTLIEVYTQLVAGVNYRLNYTYATTDCRTDQEYKPSKCRPKGKVRGWCESIVYEMPCEHIVQISQHHC

>IsCPI-10

GGWKTQDLTNPKFENLAHYAVSTQVEGREYYDTVLGILEVKTQIVDGVMFMLKFTTTQSTCKIEAGVEYSKLNCHPRTSKVVLALHGVVASRS

>IsCPI-11

GVWRRHHPDMDPRYKEWAHFAISSQVEDRTNFDTLMTLISVESQVTRPQDVTSLETSSKEQHAAPKRTSIVLVTFLVSFCFLQPYMLCTAVVNYRPWEHKTSLKSYNC

>TuCPI-1

GGWQTSDANSETIKDLAQVATEHRNSQINSLYYRTLVEIKSAKQQVVNGMKYELTLVLADTNCAKQDAGAKLCPVGQGAAKEECVYTIWVESTKEEPAVTSSSC

>TuCPI-2

SGWQTSDANSGTIKDLAQVATEHRNSQINSLYYRTLVEIKSAKQQVVNGMKYELTLVLADTNCAKQDAGAKLCPVGQGAAKEECVYTIWIESTKEAPEVTSSSC

>TuCPI-3

GGWGSVDANSETIKDLAQVATEHRNSQINSLYYRTLVEIKSAKQQVVNGMKYELTLVLADTNCAKQDAGAKLCPVGQGAAKEECVYTIWIESTKETPEVTSSSC

>TuCPI-4

GGWRDVDVDNQTVHLLSQMAINHRNSDEDTLYYRKLVNVESARMQVVSGLKYEVTLVIGETHCAKEDEAAMLCQVEPNSLREKCVYTFWLEAKTQNTNIVTSSC

>TuCPI-5

GGWSSISVDHPTVIQLAAKGVEHHNKIANNLYYKKLITIKEAKSQVVAGMNYEVKFLIGKTECVKSDANAASCEVSANAIPELCTYVFWVRPGSDNAQITQASC

>TuCPI-6

GGWNSLSTDDSTVNQLAIKSVNHHNSVNNSAYYKKLVKIQEARYQVVAGFKYEIKFLIGKTECAKTGNYTDSCQVAVNSPTELCTYVFWMPPVDKDRITSFDC

>TuCPI-7

GGWMPKDIDYEPAKDNAKYAAKLINDQSNDMYFQNLIHIHDVKSQVVGGVKYNITFDMSKTICRKNEIDSDKPEQCVPDRNATIKRCYAVVYERPWESKRQLLDHKC

>TuCPI-8

GPWVTVPVDDPIIQKYTEQALERQNKEFGDRYKRLVSINQAKRQTLSGYRFEIEMIIRETDCNKNDPKKHQCQFNSARAPEPVVFDVWVKGD

>TuCPI-9

GEWGRVSMDNPTVLELAELAVDDHNKLSTNDYYFKLVKITSVSFQALNGIKYSITFIIGQTKCFKTDPNHKKCDLLHKNINIMNLCSYLFWIKPGTPKTVEIIHHAC

>TuCPI-10

GPWTPLPVDDPTVIKLADQAVVDINAQDNSLYYNKLILIRDKRRVGDKIEYELKLVIRQTDCPKSKPYTDTCQINQDTLPQICTYELFSRAGSKNKITTLQC

>TuCPI-11

PEWKPLPVDDPTVIKLADLAVVDINAKENSSYYNKLVQIRAAKSRLMSGIEYELRLDIQKTDCPKSKPYTDACQINVTTLPPICTVHLHEDPGSKEIKITAFQC

>TuCPI-12

GPWVPVPVDDPIIQKYAEEAVETRNKKYDGHYKRLMSVDVAKKQFISGYRFEIEMTIRETECHQNDPKKHQCQFNSARAPEPVVFDVWVNTRNQ

>TuCPI-13

TEWTPLPVDDPTVIKLAKQAVADVNSQEKFTYNKLIVITEAKSVVDDGVTYMLKMIIQKTYCPLSKPYHDDCEINQGLPGSTCIVDAHKPVGSEEIKIGRLQC

>TuCPI-14

TEWTPLPSDDPTVVKFANLAVADINGKEKLFYNKLIQIKEAKSSKAKFYVFKVIVGKTDCPIRGPYTDACQIKDDAPRKECSVYYYYNTVEWKHGTYTC

>TuCPI-15

VKYKPLPVDDSTVIKLANQAVAKINAEGNGKFYNKLIEIKEARSKLRHSKITYTIKVILRKTYSHKSKPYTDACGINDTAPPKLCTIDAYVRVGSDESKIVILRC

>TuCPI-16

VKYKPLPVDDSTVIKLANQAVAKINAEGNGKFYNKLIEIKEARSKLRHSKITYTIKVILRKTYSHKSKPYTDACGINDTAPPKLCTIDAYVRVGSDESKIVILRC

>TuCPI-17

TEWTPLPVDDPTVIKLAKQAVANVNSQEKFNYNKLIEIQEPKSVVDDGVTYMLKFIIRSTYCPISKPYHDDCEIRELPGRICSVDAHKPVGSEEIKIGRLQC

>TuCPI-18

TEWKPLPVDDPTVIKLANQAVAHINEKWEKLFYNKLIEIKEAKSVADDGITYMLKMIIRLTYCPKSKPYHDDCEIRQASSAQICTVEGHKPSGSEEIKISNLSC

>TuCPI-19

GGWSSLSVDHPTVIQLAAKGVEHHNKIANNLYYKRLISIEEAKSQVVAGINYEVKFLIGKTECVKSDANAASCEVSANAIPELCTYVFWVQPGSDNAQITQASC

>TuCPI-20

GAWSSNSVDDPLVAELAAKGLDYENRYGNSFYYKKLITIKEARIQAAKPYGVNHEVKLLIGQTDCAKWNVNATSCEVSPNAIPELCTYVTWVSPDLHWRELTQASC

>TuCPI-21

SVWKSIPVDDPTVNLLTEKGIEHRNKNDNSIYYEKLIAIKKAQTQALAPAKYKIEFLIGPTECLKTDPNSASCQISTNKASETCIFVFLIRRGSNDIHITRDFC

>TuCPI-22

GGWKILPVDDPTVVQLAAKGVECYNKNSNNIYYNKLIKIKKAASEIVAGMLYEIKFLIGATDCVKSEPDASSCKVSPNAIPKLCTYHFWIKSWSGFEQITQVSC

>TuCPI-23

GAWSSNSVDDPLVVELAAKGLDYENRYGNSFNYKKLITIKEARAQADKVSGINHEVKLLIGQTDCAKWNANATSCEVSPNATPELCTYVIWVSPDLHWRSLMQASC

>TuCPI-24

GPWTPLPVDDPSVIKLAKQAVVDINAHDKSLYYNKLIEIREAKSRVADKIEYELKLVIRITDCPKSKPYTDACQINQDLPPKLCTYELFVRAGSKNKFTTLQC

>TuCPI-25

IEWTPLPVDDPTVITLADQAVAYINAQDNSLYYNKLIQIKEAKSRVADKVEYELKLVIRITDCPKSKPYTDACQINQDEPPKLCTIDAYVRAGSEENKIYKFLC

>DpCPI-1a

INASEDVELKDPTLVAISTFAANSILKDAETFGIQNTSSLHKIMDAHVVPASKPSPTQYMLTIELGNNCTNTNDNCTGGRHICEVSVLDAPWNEKRVLDEDKTKC

>DpCPI-1b

GITRVPSEELTTSLIRHAVLASLAIIDAESLSAFQFKVIGYSLVDSRVSVDSGTRDLDLKMGLVPTLCLKRPEEENEDESKSCTIDTERDPSMCTITLTQWPWVLDGYLFSDFDC

>DpCPI-1c

GCPANMDVDSIQVKELANFALSALEDAANCTKVQSILRITKATSQVVSGTLYVLTIELVDTNCIRSENTDRSQCPANELTEGNHRQCTVGIWDQPWLNSKQIREPQC

>DpCPI-1d

GVKTARSLSPLSPDDEEVKDIAAFALNRLDSFDDSNSKKRILVTVVEGTASTEGRSKTFKMKIHVALADCPEGGSANNEACLASLSGNPQHYLCDIQVLVPLRDSRFVQRRLVNSRC

>DpCPI-1e

GAQQSADKSSPYIQSVADFATKAISQRSNGNILNLIRVIRADTQLVAGKKVTLDVEVGFTNCSKAEGAGTFCQLDSSQANVICHVAVWDRAWLNDRKVTNVTC

>DpCPI-1f

GGHGQVDTMSTEIEAYSDFALAVIEEQSNADEKLKVTKILASSVQIVQGKNIRLSLEVASTSCKKDQPIGDNCAIDESKGFQVCNIQIWDRAWLQEKQVTDLNC

>DpCPI-1g

GGYSAADPTDPAILEIANFATQAASSSQSTDASNSAPFTLAKIHSAKKQVVAGINFKLDLEFTRLNESLFCRVIVLEQSWLSVREVTNMTC

>DpCPI-1h

GGYSQSDINDASVKEMADFATQAISRSTNAGALSVAKIISAETQVVSGRNYKITLQVQGDAGVQTCTVVVYDQSWTKTRKLTSFKC

>DpCPI-1i

GGYCPIDPNEKAVKEMANFAAISLSRSMNSVPLKLAKIRFAERQVVAGFNYRLDLEFTEPRGTVHCKVVVFDQAWTSTRELSQMQC

>DpCPI-1j

LGTYISIDVSDEQVGELATFATTILSHVRNAGDLTLVKISSASKQVLDGPNYRLGLQVSSVDGTNLMCDVVVYSQGNARQLTYSSC

>DpCPI-1k

GGVTSMDLHSQKIKELSDFAVSAISLRSNEPNAPSKVRVLNASKQVVSGMMYTLQLELNFVDCQQDSEACIRRQICNVSIWEQPWLKKREMTKLTC

>DpCPI-1l

GGISPADPSSEEIKAHAAFALQAIQAQSNSRNLLNIVRIKNAGTQTVAGKKIYLTIEIGQTKCPANETSQSCSFDDQTDRQLCKIEIWTRPWLNERTVTSLKC

>DpCPI-2a

GGFSPIDVDDPNVKEIADFATSTISESSNSGPLSLIKIVKAESQVVAGRNYKLILELSSVVDGASETEETLCEVIVFHQPWTQTRKLSKSNC

>DpCPI-2b

GGFSPIDTDDASVKEMADFATTRISESSNSGPLALIKVVKAESQVVAGMNYKLTLELGSAADGAVGSNLICNVLVFHQSWTHTLELKESNC

>DpCPI-2c

GRKLGYTLFAPTSMAFMMQMPQDAGDPLVMDADFRRSVLIRHFVREDVSSDDIGKLDKLVMADSNEVIFTRKSANELCNLPPIDSDRSECEAYIPSWTFQPNSGRCENYVYGGC

>DpCPI-2d

GGFSTIDVDDAYIKEIADFATTTISANNNSGPVRLIRIIKAESQIVAGKNFKLTLKLNSAIDEADSLLCDVVVFDQSWSQTRQLKQSNC

>DpCPI-3

GGYSPANVHDIDVNEMADFSRRAISSRSNSGPSTLIRIVKAEKQVVAGMNYKLTLEMENANDGVILCDVIVFDQPWTNTRRLRESSC

>DpCPI-4a

GEFSPIDVNDSEVREIADFATTAISASSNAGPFRLIKILKAESQVGIGAVNFKLTLEVDGADEKNLRCEVVVFDQSLVKSWTSVC

>DpCPI-4b

GGYSPIDVNDAKVKEIARFAAAAVSINLNSGPIALVNIVKAESEAVAGRNYKLILELEGSDGEARICEIVVFDQPWTNTRILSNSNC

>DpCPI-4c

GGFMPMNVNNEQVREIAEFATSAISSKINSGPVTLVNIVMAESQTVAGKNYKLTLELEGSQRDKHLCKILAFDQPWTKTRILSEFNC

>DpCPI-5a

GTKLSYTFFAPTSFAFTMQTPQDTVDPLFVDASLRNKVLIRHFARQSISSDQLAKLDKLVMADSQEVVLSGKSGIEICDLPPIENKGFECYALKHSWTFKSGKCVNYVYGGC

>DpCPI-5b

GGYSAASASEEDVQEIAKFATHALSQNANQASPFVLVQVVKAEKQIVSGINYRLHVELKENADSANVISCTVVVYDQSWTSTRQITSSEC

>DpCPI-6a

GGYKPAMVDAADVKRMAAFATSAISASDSGPAVQLIWIRRAWKQVVSGTNYKLILELLNTNTGQVLLCEVIVFDQPWTNTLELRSFRF

>DpCPI-6b

GGYVIRNVNDSDVKEMAAFAFSILTANSHPHHLALIKILKAESQVVAGTNYKMALLFANRPQHHSRYLLLCDVIVFDQPWTHTRKLTEYKC

>DpCPI-7a

GGFSPLDVENVTVKEMAAFATTAISANTNSGPVTLVKVVKAQSQVVAGLNYKLTLELNGAEGAILCEVTVFDQSWTNTRKLTESKC

>DpCPI-7b

GGYKTIDVNDATVKEMAEFATSAIPEKMNSGPVTLIKIIKAKSQFVAGINFKLTLELEGVQGAIQCDVIVFYQRWSKTRKLTQSKC

>DpCPI-8a

VGFYRIDVDDVEVKEIALFAAKALSKSQNSILKLNKIILAEAEDVAGKNFKLVLRLENLDEEEVSFSKSFINCEVVVFDQSWTSTRILRESDC

>DpCPI-8b

GGFSQIDVDNDVKEIAIFAGTAIASKRNSGPARVTKIAKAESQIVAGTNYKLTLELNQPLATEKFLICDALIFDQSWTKTRILSEHRC

>DpCPI-8c

NKFSSIDVDDPDVKAVGEFAMKVANTAAASSGHSAPVKLVKILKAEWQVVDAVGRNFKLTLELDDGAEESLLCVVSVFEQSTWKMIQLSFVTREV

>DpCPI-8d

GGFSSIDVNDPAVKEIASFATSTIASKKSKSGPTKLIKIVKAESENVAGINYKLTLELNQPLAIDRSIICDVIVFDQTWTSTRILSESHC

>DpCPI-8e

GGFYPTDVENPEVKEMAIFASKVLESKRNSGPTKVTKIVTAESQAVAGTNYKLTLEISQPIAVVETERFLLCKVVVFNQPWTKTRILAEWNC

>DpCPI-8f

DGYSPVDVDNSYVKEIAAYATTAISSSRNSITLSLQRILNAEARVFGGTNYKLTLELDHFIAGAKAENLLCKVIVFDQKEDYRKMTDSLC

>DpCPI-8g

SGFSPADVQADDVREMAYFATHVISNTRNPVALAEIVKAESQAHAAGRNYKLTLKLDSMVKETTAGIPTFQSGDLLCEIIVFFQTWSNTRILSESNC

>DpCPI-8h

DGFASLDVEDVKVKEIAAFASNSISANSGPVTLLRILKAEAQTVAGKNYKLIIELIGTERDIQICDVVVFDQERSQTRILIDSKC

>DpCPI-8i

NGFSPVDVNNPEVKEMANFATSSISASDNPFILKLIKVVDAEAKFLFGKNFKLILRVKNMVKGADGDREMLCEVVVFDQSWTSTRKVTESTC

>DpCPI-9a

GGFTPAKLNDAEIVKMVDFATTAVSASMNSGPVKLLKIVKAEIQAVSGTKYKLNLELAGAYSKVIPCEVVVFHQPKTNTQKMLRSSC

>DpCPI-9b

GGFSSLDVDDAEVKEIAAFASSAISANTNSGPLTLVKVLKAQSQVVAGLNFELRLELKGAKGAILCEVLFLRQCWSQISAAPFRSDC

>DpCPI-10a

GTKLSYTFFAPTSFAFTMQTPQDTVDPLFVDASLRNKVLIRHFARQSISSDQLAKLDKLVMADSREAVITRTADGKISIDNAEIQPGAIALSQNLGNVYLVERVFMTGDEVSNAISAHFQNNPNTALC

>DpCPI-10b

GGFTSVSINDADVLEMARFATNALSVNKASPLVLVAVVQAEKQIVAGVNYRLQLKFNGQQLESEENHFIDCQVTVFDQVWTATRQITSFQC

>AgCPI-1

GGVSDDPELNKEEHAERIGAALATTDGHAGKAYKLHRVTKQVVSGVQYVYFISFENEESGQQYKITVWERPWLKEKDPAEARKITFEV

>AgCPI-2a

GGIVAIHVDAVGGKEQLRPTLRGLSYLATSFLPRDYKFVEIVSATREVVAGVRYELLASAEDETTGQRHLCQLVILEKPWITNEYGEKYRTLEYTNC

>AgCPI-2c

GGATPVDVKEPTHIERVRLGLVGYESGKHSNFEILFGTVQVVAGTIHRYKIALKDDDQKVYSTCDVKVFTPLPSAANGSKPDYDFDC

>AgCPI-2d

GAAQELTPEEYAKEEHQTRIRTGLQQQSALVDGSGNERKVKVVGATVQLVAGKSYTYRLSFPDDELKRVCKLTVWEKPWLKEKAPQEAFKASFEC

>AgCPI-2e

GCATPLAANEYGNSEHQERIDKILSFHGLTRGNSLKVINATSQVVAGMKYVYFIQHNNAVCKLTSWERVWLAQSHPEDAYKYTYDC

>AgCPI-2f

GGSRSLSQDELAAAEHLERVDKILVSSGGSKESSNARIVSGTVQIVSGKLYKYAVEFDVDGSSKLCKLSSWERPWLEKKDPTEAYKY

>AgCPI-2g

GSSNELTAEELKDKSHVERIRAGMVSYNSERSKAYNEFEILAGSTQQVAGSLYKYTFRVTSESDIVCKISIWERVWLESQDQRKYNVKC

>AmCPI-1a

PNLPGFISFGEQVAKSMDELIQNDFKHKVIDIVKVTRAIPPSSNIIQYQILLHIGESDCLKNAIEQSECSVQLNSSFKICLVTFEEKPWQQSSRKIVKNNC

>AmCPI-1b

DINNPTVQELANKGLKKFSENSEGSNEPMIVEIVDASRQVVSGYLYKIRVKLGTSNCPKGTKEKCQLKEGTEIKECLFSIWSQPWIDKGSPKITINC

>ApCPI-1a

GGYNDVEADSEKIRELALFSLDSITQQTMSKRSLGLIRVVSAKSQVVAGINYKIKLLVCEKDSTLGENIVMDPKNCRSCDITIWEQSWLNKKNVTKVAC

>ApCPI-1b

GAKISLNSNDKKVQDIVAYALLSIDRQEGSNKPHVLSKIINVSKQIVSGIIYNIELEICDNSTSEVDEKKCRICNIKVWEQAWENNKNTSEFNC

>BmCPI-1a

GCSTRVDVDAAGVQELASLAVHHLDRHDDTAKYSLISVVDVERQVQVVNGVRYILTLLVNNNTCTENQSEDCQVVTPCRISILEKPWLRLPSGVKYRSILSNNC

>BmCPI-1b

NTIRTVPPNDPILQMMVRVSLKKIEKESNEKNAMKVSKIIDANVQKTSGILTKFLVVLDRLNCSQNTPISLRQNCTTVEDLGSKVCDVVVFEKLWLKDKDVSFTC

>BmCPI-1c

NIRKTFEIDDYKVSEMLQESLMYLDVKSNRNNKQKIVDVNSVSTQINAGLLTEIIFTVAYTSCRNDVKVDINTCNVLEDEPLRNCKAQIWDRTWIEDGTQIKVSC

>BmCPI-1d

GGLQLQDAHDQKYKLLAEESLRQFLQKNGTTKPHTVVRLNKVTTQVVSGTLIRLDFVAAPTGEESRYQCHSEIWERPWLKKTDIEVNC

>BmCPI-1e

SGMTESDVTEPHYKKLAQQSLNQFLKESGNTKPHIVVRLNKVTTQVVSGTVTQLDFVAAPTGEESHYQCHSKIWEQPWLKKTSIEVDC

>BmCPI-1f

GGKHEEDPSDKEFKVLAQESLHEYARLEKNDFIHKVIDVNRVSTQTVSGKIYNIHFSAVPTSCSTAVQDPSFCEQKDGSSILQCHARIWSRPWLGKKTTTITC

>BmCPI-1g

QTLDDGDYIDEELRNYYAERANQYLNQVSDTNNLYKLITVHAIKYGKQMGRNIVQMYIEVAPTFCLRHADENELGGCEEIEALDHKLCYGRLWPSPDDELVVQSVSVIC

>BmCPI-1h

ETVSGISIRRLIKSSIKELEKNPDQKYKLIHLGTPYLVPSLDSDVPIKLSFLIGSTNCTKEVDIENSPLQCFLDGSKSSKPCTSFVWFVPNTKDIYQINVQC

>BmCPI-1i

LNSANVSANDLEIRELVKQSLDKLEMASVHRYKQRVIQINSFSTKITTGKVTTIDFDVGYTSCLKYEWVDDVMTCQFLEHLPRRHCVSKVFERLWAANGKNIDVSC

>BmCPI-1j

PLEAHVEFESAEMALQLANEALKHIEARYPNPRKQKILRIFTLEKQVVAGIHYRMKVEVGLTNCTALTNRSDCKHISDESLNKFCRVNVWMRPWTNHPPNFRVTC

>DmCPI-1a

GDAESTESSETTTDQAVSEPPITLVHVLNPGEREYLSPNLIGVQNIAMTFLPLSMNFVNIIDAFREITAGVRYEILLNALDTKAIQPAEADIVCRLVILEKPWLRTQWGDKHRELVTSNC

>DmCPI-1b

GRHKPYDEEAAKAQLQKSLDKLTAGEGPHYKIVKVYSASRQVDSGILTRIDADLIDGSEEQHRCIVDIWTKVWVRKDEHEITFKC

>DmCPI-2

GAPKPLDGDDLSKAKELLDTTLAKLATGDGPNYQVVNVISASSQLVAGSLYKFEVKLSNGAETKECNVKIWDRPWLHEQGEATNVKVQC

>DmCPI-3

GGVSQLEGNSRKEALELLDATLAQLATGDGPSYKAINVTSVTGQVVAGSLNTYEVELDNGSDKKQCTVKIWTQPWLKENGTNIKIKC

>DmCPI-4

GGISQLEGNERKEALELLDATLAQLANGDGPSYKALNVTSVTGQVVAGRLNTYEVQLDNGSEIKQGTVQIWSRAWLKENGTNIKIKF

>NvCPI-1a

PSLPGLSAFGNQVMQSMDEAGVSDFKHKLISIVRVTRAVPPGANVVQYQLLVEIGESNCLRTSLIEIAECPLQSNLPIKLCLVTFEERPWQSGSRKITRNNC

>NvCPI-1b

GKALDSKLTSRKLAVKAVELLDDLDEDDKRKHVIDVIDSRRERQDKDNSNVVIYMTVEVAATDCSEKDQGTECLDEVLPGPTQICKLDISTNEKKPLQSPKLLHWSC

>NvCPI-1c

GAPTSHSKDDPEVQRYVQLGLEKYTENYQGTNQPMISNIKDVSVQVVSGLLYKIQTDIGVSTCSKGTVTGDCQLSKDHGVEECVIEAWSQPWLDKGNPKITVKC

>PhCPI-1

GGEKETDVNNPTVLSAIKSTMVKLNENLSSGENEKKFVETLKATVQVVSGTLTRVLLRINQGEETHYCYSKVWEQLWLNKTEVLAHHC

>PhCPI-2

GDRNLLNISDPEIIRLSKIALLKLNEEKNIFNQEKILVKILKASKQIISGSLTELTLQILEKNVPKYYVAKIWERPWLNKTEVTFFDY

>TcCPI-1a

GCPFDLNTNAEGVDELIDVALEHIQTERAKKHALVKVLRLQQQVVTGVKYILTAEFAPTLCEKSANLDASSCPRDTNAETTICEITYLHKPWISKAKHVIKNNC

>TcCPI-1b

RAIRQLEKISPDEKGLVKNLADFAANALDSIDDDNNKRIILQILGAKKMVGDDGVYYHIIMRMGVSRCLEDSPINPYENCKDKLFENYTKICKVQVYVNDDFGSKKVVKSQC

>TcCPI-1c

GAPNRIDKNSEKIRQFVKEGINGFNANYNSKNNKVKPVEVVSATTQVVAGTLYKITTKISESDCSKNDNKDLDDCNILEGASPKTCELEVWEKLWENFRQFTIKC

>TcCPI-1d

GGESPVTKDNEYVVKYLEAALNQLDSESPHENKFKVHEFISATSQTVSGHIYRIKTKVVLSDCKKTASTERGQCGTLKDAKPKTCKFEVFEQTWVPNSRRIKTDC

>TcCPI-1e

GGITEVDKDDDEVKTFVREGLLNLNTHLTTSNKVKPVEVVSASVQVVAGSLHRIKVKISESDCSRNDQKDFEQCNVLEGASPKLCEMEVWDKPWEDFRRYTIKC

>TcCPI-1f

RGESPVAKDNEYVIKYLEAALNQLDAESEHENKFKVHEFISATSQIVSGHIYRINAKVILSDCKKTVSTERGQCGTLKDAKPKTCKFEVFEQLWVPNSRQIKTDC

>TcCPI-1g

GGIKEVDKNNDEVKTFVHEGLLNLNTHLTTSNKVKPVEVVSASVQVVAGSLHRIKVKISESDCSRNDQKDFEQCNVLEGASPKLCEMEVWDKPWEDFRRYTIKC

>TcCPI-1h

GGESPVAKDNEYVIKYLEAALNQLDAESEHENKFKVHEFISATSQIVSGHIYRIKAKVILSDCKKSISTERGQCGTLKDAKPKTCKFEVFEQLWVPNSRQIKTEC

>TcCPI-1i

GGATEIDKKSDKVKQYVRESLTHLNTQLTSSNKVKPVEVLSATSQVVAGTIHRIKVKISESDCSKDDEKDFDECNIREGASPKICEVKVWDKPWQNFRQYNITC

>CfCPI-1a

GCPYELNPNLPSLLVFAEQALKSIDEQTANDYKHKLMSIVKVTRSVPVSSNMIQYQLLLLIGESECLKNALEQEQECPLRATNSIKLCSVTFEQRPWLPTSLKIIRNNC

>CfCPI-1b

GGTITTSVNDPEVQLYANKALRKVSEESDGPNEPFIVEIIEASVQVVAGKLYKIKAKLGTSDCPKGTKTNCQLQAGSEVKECLITVWSRPWIDHGSPEITITC

**I. I31 Thyropin.**

>SmTyr-1a

EKRARALETHVPIAKCRSDGSFESIQCDEAECWCIDDGGFELAGTRTNETNVNCSKPRP

>SmTyr-1b

CEQQRLLAELLSRNEKSDRGYVPECSVKGHFKSKQCSRNGLVCWCVDLRGNKLPRTMGPAENVTCA

>SmTyr-1c

PLSRNLAYPAPQCTREGLFEPIQCADGVCWCVDEFGVELAGTRDSNRRIVDCT

>SmTyr-1d

KTGCEHARAVARHFAPSCRAEDGQFETVQCANGTCWCVDDSGVEFPGTRTLGRPNCSSP

>SmTyr-1e

LVIARCNAFSGAWESIQCLPSLGLCWCVNRDGDQMAGSLVRGVPACSS

>SmTyr-1f

VTLPQCTDDGAYSPTQTTGILSWCVTTDGQPIHESIGRGDIRCSPEG

>SmTyr-1g

KMLTRCQKEEIVAQTWSQRVPSCDNEGGFVPTQCDDRVCWCVDQAGIKVPDFEN

>SmTyr-2

EREKAQQNQNILGLFVPECKASGTYKAKQCHEAYCYCVDPQGTQIKGTIF

>SmTyr-3

SYIPSCDEHGYYQATQCSHATVELCWCVDKHGVEYANTRMKGKPDCDSIVGK

>SmTyr-4a

KTCREERQEALDRQENNPEMVGIHVPKCDANGDYQPKECKQAYCSCLDYDGYPIRGYL

>SmTyr-4b

CHEERQDALDRQQNNIGMVGIHVPKCDQDGTYSPKQCIEAYCHCVDKDGNVIVK

>SmTyr-4c

HGMIGRTIACDKAGNYERSQCTGSKCYCVDSKTGEKIGDVVPISQK

>SmTyr-5a

QKPCRVDRAKTTGKYPMCDGNGNYEPKQCEYSPRQTGNTLKKRCYCAHKDGHLLGNYYFD

>SmTyr-5b

VGKTVSCDEIGNYKSAQCLGSGCYCVDRKSGNRIGDVVHINQAQTLN

>SmTyr-6a

VSYEEADRNSEWFLPIQCEKDGTYKPIQCKQDICFCVDKDGNRIFGDFPLLE

>SmTyr-6b

GQFDHVICQKNGNFHKLQCLRDTCFCVNETTGNLTSSIVPK

>SmTyr-7

QLAADQQHTEGKVFVPECTIEGKYSKLQCFKPTGYCWCVDPESGKTIPGTSIQDDKPDCD

>IsTyr-1

CDVNRDSFLSTIEWCACFNKSRAYIPQCDEEGYFMPAQCHSSAGMCWCVDRHGAEFANTRRRDRPDC

>IsTyr-2a

CPDARRCWSQRSQALEQVRSGASGVFVPDCSADGAFVQVQCHRLTGYCWCVDAQGKVLSGSSVQNRRPNC

>IsTyr-2b

EVQDCSSARRKALEAHRQAPKGRIYVPECGSDGTYAEAQCHTGYCWCVNQRTGRPIRGIATLGVKPDC

>TuTyr-1a

SSDTTSCTYRRQMETNSNTRLPGRLIPECRPDGKFASLQCHGEAVGGGRFCQCWDPEGNIIRAPSKKIKACDC

>TuTyr-1b

CDCILQRHQITSEKPKRVGHFTPQCEENGHFKKYQCHASTGHCWCVHPTNGTQIGEKSRTLTAEGC

>TuTyr-2a

CLLERSANLAVKLESDHQSGKEILIPECDLTKGLYKAQQCHKETGYCWCVDVNNGKPIWKTSAKGDKASTDC

>TuTyr-2b

PNCSESRQIALKNSANFSHSKTLIPSCNNQDSFLYNEVQCHELPGFCFCVRPSSGQLIPGISSKLPAKPDC

>TuTyr-3

CFDRKSKPCLIEKSQRKKVYDEFVPKCDKDGFYQPIQCHLHKCWCVDRYGIEIENTRQTEIPDCD

>TuTyr-4a

QCAYGNTKCLQHRTEALKTSLNESEPNYIPGCNTFGDYSNYQCFGKRCFCVDENGDRIFGDFLDSQ

>TuTyr-4b

CWSELTRRNEELKFLKKKFDFVVGYELPECNLDGSYKAKQCDETSCYCVNQKGERYGARVTIPRDSSE

>TuTyr-4c

SEIEQMRCNCIRDKDLLRQANDKTNWSDYDCDHLGNYNPIQCFGDSCFCVDQNGLPIDYERYNVTLKD

>TuTyr-5a

IQQREKAINLNGKSFCSNRAGILVKTGFLFETSEYQPECDYETGQYKPKQCKTSKCYCVDPDSGEKTFGTVESVNGSNMTC

>TuTyr-5b

CSVALSRYTSDPDNYLDYQSSRHLHCSENGNFASLQCHDQLCFCADPSTGQQVSPLVLIDAVSSLSC

>TuTyr-5c

CEQLLQPLANLTSFLGRKGIDAIGLNSMQCDLDGNFVHRQCTDSECSCVNSVGENVANYRIDRYGNDDRTMKC

>TuTyr-5d

IQEYEITTLGKPYDKLLCDLYGNFKANQCFESGSRCFCVDSNGNRISNVISSSVI

>CfTyr-1a

CEQLALAAVRRSRALGAEGLSQFVPRCDNETGEFERIQCNPQGRGCWCVDEIGAEIPGTRALNKNDIDC

>CfTyr-1b

CHRDRMLAEILSVSERQGRGYVPQCREDGGFETRQCSRNGLVCWCVDDEGRKISGSMGPSKKIDC

>CfTyr-1c

CEYLRDFNDRMEGTREGMSLAIPAPQCEQDGSFKSLQCHNSTDCNCVNHRGVILKTGVGPTASADC

>CfTyr-1d

CQHARAVAEHAARESGEPARRIYIPRCDTNGVFEPVQCHNGVCWCVDEEGKEAAGTRVLEGIVPRC

>CfTyr-1e

LSQCERLREKNLKRSQRLKQPTFLPKCNSDSGTWEPVQCLEHVGVCWCVNRKGQPIKGSLIRGTEPKC

>CfTyr-1f

LEMDERRAGKILGTRCQAMKNKGHVPTICDPQGRFEPTQCAGDTCWCVDEAGNQLIGSEPFLKGTSIC

>CfTyr-2

CFYKAERPCAAVRRRSSPDSAPSCDSRGYYRSTQCHRGLGLCWCVDPHGVEFAGTRTRGTRPDC

>CfTyr-3

MSDRRSVLEDQKQNSQEKFYIPQCTSDGRYHRVQCYSGYCWCVYQDTGKPIPGTSSKNRTPNC

>CfTyr-4

ETPACFSARLTARLGARPICQHDGTYAPIQCHIETGYCWCVTPQGRPLPDTSVKHKKPRC

>DpTyr-1

CFSNADRPCVAMRRRSKPGLLGAYIPTCDSDGFFLPTQCHTAVGTCWCVDKHGVEQNGSRARGKPDC

>DpTyr-2a

SAGGLGKKSAPTGETFIPECNEDGRFAEIQCHQGTGYCWCVTPDGKPIPGSSIRHNKPNC

>DpTyr-2b

ANDCYSDREAAQEEVDHGAKGMYVPECTPDNKYQRVQCHKSAGYCWCANDETGKPIPGTSVQNSKPTNC

>DpTyr-3a

CERQRVETVKRARALQMTDNDVSLPSCDPIGDYEPVQCDPLTGNCFCVDESGFELAGTRARSLQLVNC

>DpTyr-3b

CLQQQQIAQLLSLTEREGKGYVPQCDEDGQKFAARQCSRNGLVCWCVDPELGTKVKGSMGSAQDVVC

>DpTyr-3c

CEMMKEIADGRKPAEPGYNLILKNPRCTPQGEFEEEQCDKDGQCWCVDEFGVELAGTRGVASVQQRS

>DpTyr-3d

CQHMQMIMKYKARENGLPANRLFIPRCRPEDGAFEAVQCDPVTRACWCVSADGRELAGTRVPPGLQPQC

>DpTyr-3e

STCEELREKNLKMAEKFKKVVFTPKCNKANGDWEPVQCLEEVGICWCVDKDGEHIKGSLTRGSPTC

>DpTyr-3f

SSTCTSPEENSSGPLMMSISPPVCTLKGDYAREQSQGEFSWCVDTTGQPIDDSFTRGSVRC

>DpTyr-4

KKGGGGGGGGSGELIPECDHLGRFQPIQCLPAKSSSGQVSCWCVDEAGNQVANTTQFLRGEQTC

>DpTyr-5

TRCQKMRQTQLKKGGGGGSGELIPECDHLGRFQPIQCLPAKYSSGQVSCWCVDEAGNQVANTTQFLRGEQTC

>AmTyr-1

CFSKAERPCAAVRKRSSPDVAPACDSRGYYRSTQCHRGLGLCWCVDPHGVEFAGTRTRGSKPD

>AmTyr-2a

CEQLAQAAVRRSRALGPRGPAQFIPKCNNETGEFERIQCDPREKQCWCVDEIGVEIPGTRENSIDAIDC

>AmTyr-2b

ACHRDRVLVEMLSISERQGRGYVPQCSEDGGYESRQCSRNGLVCWCVDNNGRKISGTMGPADKVDC

>AmTyr-2c

CEYLREFNERMEGTREDMSLAIPPPQCEKDGSYKPLQCHNGTCSCVNDRGVVLKSSVNRSSDCK

>AmTyr-2d

CQHARAVAEHAARESGEPARRIYIPRCDTNGAFEPVQCHNGMCWCVDKKGREAAGTRVLEGIVPKC

>AmTyr-2e

LSQCERLREKNLKTSQRLKQPTFLPRCDSENGMWEPVQCLEHVGVCWCVNGKGQPMKGSLTRAPEPKC

>AmTyr-2f

LETDEKQIGKVLGTRCQAMKDKGHVPAICDRQGRFEPMQCAGDTCWCVDEAGNQLIGSEPFLKGTNI

>AmTyr-3a

TPACFSARLTARPSARPICRSDGTYAPVQCHEETGYCWCVTPQGRPLPDTSVRNERPRC

>AmTyr-3b

CLTDRRSVLEDERQHSQEKFYVPACTPDGRYHRVQCYSGYCWCVYQDTGKPIPGTSSKNHTPNC

>PhTyr-1

CHCFDKTDRPCTAAKRRLSPDMLGVFVPDCDNQGYFRSTQCHTSTGMCWCTDKHGVEYANTRIRGMPNC

>PhTyr-2a

CRTRQPCFRELKKAERNRENQEFVPTCLKDGTFSPMQCHNETGFCWCVTPKGKLIPNSAKRHDKPNC

>PhTyr-2b

DQEPEANDCFSDRQAVLAEQRSNNLYVPECTPDGRYNRIQCYKSTGYCWCVNEDDGKPIPGTSVKDQLPKC

>BmTyr-1

CLRRAARPCTALARAHPQPHAGAYVPSCDARGFYRPRQCHAALGVCWCVDAHGVELPGSRTKGAPAC

>BmTyr-2a

NVTMCTQQKMLAELLVVSEREGKGYVPQCAANGSFESRQCSRNGLVCWCVDTDGNKLRGSMGPSATVHC

>BmTyr-2b

LRDFDEKMEGTVDGMKLALPAPTCQQDGSFTSQQCANGRCWCVDSFGTEIPETSTHNASAVDC

>BmTyr-2c

CQQRRALALHTAAESGNPPAWAWVPQCTEDGAYQEVQCRRSDKTCWCVDTAGNEIPGTRTSNSTPTC

>BmTyr-2d

PCERLREKNEAAALKYGKGTFIPVCDASGAWEPVQCMSHIDVCWCVSARGEPLKGSLVRGSKPSC

>BmTyr-2e

EEKISFKTKCQMLQAEIDNGSEGYRPRCLSDGSFSPRQCVRGRCWCVDAAGERRHHAGPVP

>RpTyr-1

CSDKQPCWADRNGRDPEGGLVFIPKCLSDGRYAPVQCHEATGYCWCVTPQGKPLPNTSVRHARPKC

>NvTyr-1

FSKAERPCAAVRKRSSPEVAPNCDSRGYYQSTQCHRGLGLCWCVDQHGIEFAGTRVRGTKPDC

>NvTyr-2a

CEQLALAARRRSRALGPGSPAQLVPRCDNVTGEFERVQCDPSGGCYCVDEYGGEVAGTRAPQRRLVDC

>NvTyr-2b

ACHRNRMLAELLSISERQGRGYVPQCSEDGEYERRQCSRNGLVCWCVDALGQKVSGSMGPAEKVDC

>NvTyr-2c

CEYLRDFGERMEGTREGMALAIPAPQCEEDGSYRALQCQEKNCSCVDEYGASLKSPVEPASTDC

>NvTyr-2d

CQHARAVAEHGARESGEPARRSYIPRCDADGQFEPVQCHAGMCWCVDEEGREAAGTRVVEGLLPKC

>NvTyr-2e

LSQCERLRERNLKTSQKYKQPSFLPRCNPDTGAWEAVQCLEHVGVCWCVNKKGEPIKGSLTRDAEPKC

>NvTyr-2f

SAEGKEINKVLGSRCQAMRERGFVPAICDKYGRFEPTQCAGETCWCVDEAGNQLVGSEPFVKGTNIC

>NvTyr-3a

GARIVCRPDGTYAPVQCHPQTSYCWCVTPQGRPIPNSTVREGRPRC

>NvTyr-3b

CISDRRSVMEDQRQNSDRKFYIPECTPDGRYHKVQCYSGYCWCVYQDTGKPIPGTSSKDLTPNC

>TcTyr-1

CFQRTERPCAAVKRKITPELLGVYVPDCDNQGYYRPTQCHSAIGMCWCVDKHGVEFANTRTHAKPNC

>TcTyr-2a

CQTLRMAASRRAKALGVEARSVRMPRCNKSGGFEPIQCDNEIVSSCWCVDEAGFELPGTRAPAAALVNC

>TcTyr-2b

CTHPKNVTECLHQRALSEILAVSERAGRGYVPQCSEDGQFEPKQCSRNSLVCWCVDRMGRKIRGSMGPAGNTNC

>TcTyr-2c

CEYLHDFSESMEGTREGMTLALPSPSCDSDGNYISTQCHKGECWCVDNFGTEIPRTRGTTQNC

>TcTyr-2d

CQHQNALSQHQAHESGVPAGRVYIPQCTPEGAYEPKQCNPGTNECWCVDWRGFEISKTRTNSQLSC

>TcTyr-2e

LSQCERLREKNQRAAERYHKPTFMPRCEANTGNWETVQCLEHVGVCWCVTPQGEPLKGTLTRGAQPLC

>TcTyr-2f

SNTRCEALGGQCDTTGKFLPTQCEEETCWCVDEAGNQLLHTNTFKKGEITC

>TcTyr-3a

CHFEKARCVNKNLTLAKRGPCRQQKLCRDWEIYRHSNPDYKFHATCRPDGSYAAAQCHPDTGFCWCVTPQGIPLPYTSVRWRPDAKPHCGRKKKSTRRR

>TcTyr-3b

DEDPSDCLSDRQTALNDGGQFYVPECTPDGRYKKIQCYKAAGYCFCVHEDTGKNIPGTSVKNGKPKC

>ApTyr-1

RCFDKTDRPCTAIKRRISPNALGVYVPACDAEGYYEHTQCHSSVGMCWCVDKHGVEVPNSRVRGKPNC

>ApTyr-2a

NDSLLRVKHRGRCKGDPTRTPDSYMPRCKADGTYFRIQCHKKEGYCWCVTPAGKVVANTIVRGQKPKC

>ApTyr-2b

QEEEAEVNDCLTDRQEALDDPSTSSHKYIPECTVDGRYKHVQCYKSVGYCWCAQEDTGKPIPGTSVKDSNPKC

>DmTyr-1

CFEKTDRPCAAVRRRIAGDFAGAYAPDCDIQGFYKPTQCHNSVGVCWCVDKHGVEFANTRTRGKPNC

>DmTyr-2a

CNACLEAVKFARRQQERDPGYFVPRCRKDGNFAAMQCYGNNGCWCSDSQGRPIADDNKQFRRKGKLRC

>DmTyr-2b

DSNCWMDQSVTLEEQGHGGKSVLFVPQCLPDGRYQRIQCYSSTSTSYCWCVNEDTGKSIPGTSVKNKRPQC

>DmTyr-3a

ACQHLRRSESRRAKALEGSSVRVPRCQKNGDFDAIQCQDEKHGRDCWCVDDYGVELPGSRNETRTGVVC

>DmTyr-3b

CQQPGNVTSCHQAKALADILSINEREGRGYVPECNGPGGQFSPRQCSRNGLVCWCVDPRTGHKIKETMGAANNVNC

>DmTyr-3c

ACQHLQAIQLHQSSELGIPARQMAVAQCDPNNGKWNQVQCSPDGHCWCVDDQGKILPGTRVKSPATPKC

>DmTyr-3d

QCERLKLKNNLAAQRTGHSSVWFQPRCDPVTGHWSPVQCLGKQPQPMDRHTEIVSRAFASEPAASAGEEAPGVCWCADKKGAPLKGTLTRESEPIC

>DmTyr-3e

VEQATLKPMELKTTRCRALSKTAPFPVSCDEAGAFRPLQCNGRSCWCVDAAGNQLQSTHVFGAGDRRC

>AgTyr-1

CFEKTDRPCAAVRRRLGNDLSGSYAPDCDSQGFYKPTQCHQAVGVCWCVDEHGVEFANTRTRGKPNC

>AgTyr-2a

RGSCKDVCIASRTYALQQRASSPYTVKYVPRCREDGTYAPVQCIDGGGCWCVNGQGKQLPNTMVQHGKPIC

>AgTyr-2b

SDCLSDRKYALDEQKYGTNALYVPECTPDGRYQRVQCYRSTGYCWCVNEDTGKNIPGTSTKDEKPVC

>AgTyr-3a

CQHLRRAEARRAKSLGDSLLQTVRIPRCTALGDFEPVQCSNELNGTECWCVDEYGVEITGSRRSHADDVNC

>AgTyr-3b

QPHNVTVCHQARMLSELLSVNEREGRGYVPQCDGPGGSFSTRQCSRNGLVCWCVDPKTGNKLKGTMGAAATVSCEVVENMIGGR

>AgTyr-3c

ACQHLQTIQLHQASELGVPPKQKYIAQCDIDGSFRTIQCGPGNVCWCVDEFGNEKSGTRTNNGQPNC

>AgTyr-3d

QCERLRLKNAMAAKRAGQPNTWFQPRCDPETGFWSPVQCLGSMEDTTNASNGTNGTAPAIAEPPAPVGVCWCADKKGAPVKGSLTKGSEPKC

>AgTyr-3e

EPIAKRLSASATRCQALQMAASFPVACDTAGSFEPMQCNGDTCWCVDAAGNQLPLSSTFKRGQRSC

**J. I32 IAP.**

>ApBIR-1

WQMKRLKSLQNWTMGKPSAKDMAEAGFYCPNPDIPDTVRCFSCFIELDGWESTDKPWEEHKKRALSLNPPCRFIEI

>ApBIR-2a

TTYENRLRTFYGVWKLNFITPDQMAKAGLYYLGIQDRVRCLYCSTEFDYWQQGDDPVVEHKRQSPQCQFFN

>ApBIR-2b

LEARLKSFEKCLIPLKQNIQTLCEVGFFYIGNGTNDQMLCYYCSQGLKDWEENDEPWTEHAKWAQSCSFVQL

>ApBIR-3a

KNRLKTFAGVWKLQFITPTQMAKAGLYYVGPQDRVRCTFCSSEYDYWQPGEDPSAEHKRQSPHCAFFND

>ApBIR-3b

RLQSFEKCLIPLKQNIQTLCEAGFYYQGTGTNDSMRCYYCDQGLIDWDDYDEPWTEHARWSNTCIHVLL

>ApBIR-4

YENCLKSFKKWPSECITPDKLARAGFYYTGIQDKVRCLYCPIGFECWGKDDDPYIEHKLASPECPYFKEKLDH

>ApBIR-5

ESRKKTFETFTKKLTHDVKTFCKAGLFYIGENDRMLCFCCNQGLMDWEVDDDPWVEHARWSPLCSYVLLS

>ApBIR-6a

SNRLSTFAGWPVSFIISPKCLAAAGFYYTKQTDKVKCAFCNICICHWEFGDNAVDEHKRHNPDCSFIL

>ApBIR-6b

FSARLKSFRGWNNESQKPEDLATAGFFFTGSNDEVRCYYCDGGLQNWEVADNSWVEHAKWFPNCGFLNLV

>ApBIR-7

LSRLKSYNSFPPTLCQNKYSLSEAGFKYSGTADIVECFCCGLVLQKWTKDDIPFVEHAKWNPKCIFVLL

>ApBIR-8

FTSRLKTYNLFPPTIPQNKYVLSECGFIYTGVQDIVECFSCGLVLHNWKKDDIPWIEHSRHNSKCIYVLLSKGNHFVEH

>ApBIR-9

SRFKTFKLFPSNTSQNKYTLSECGLKYSGLDDVVECFCCGLILHNWERLDDPWIEHCRFNPRCLYVLLM

>ApBIR-10

MNRLNSLQNWTMNKPSAKEMAEAGFYCPNQDTPDTVRCFSCFIELDGWEPTDQPWEEHRNRNLSSKPPCKFVEI

>AgBIR-1a

NRLRSFTSRWPVTFISPNVLARYGFYYVGTDDTVKCYFCRVEIGLWEPQDDVIQEHLRWSPYCPLLK

>AgBIR-1b

ADRLKSYEDWPTSLKQKPQQLSDAGFFYTGMSDRVKCFSCGGGLKDWEQEDDPWQQHAIWYSNCHYLQL

>AgBIR-2a

EINRLRTYFPLWTVPYIYPEELARWGFFYTGYRDCVRCYFCHIELGGWDEHDVVIEEHLKWSPDCRLMT

>AgBIR-2b

DNRLASFQEWPKCMKQTPEQMADAGFFYTGKSDVVICFCCGGQLRDWLPEYNPWVEHAKNFSGCPYLKLV

>AgBIR-3

DARIRSFESWRFGHMQNPTRLAVAGFYYTGTDDEVRCFQCDAGLRDWLVTDDPWQEHARCFAECTFLRLV

>AgBIR-4a

NRLRTFPLWTVPYIYPEELARWGFFYTGYRDCVRCYFCRIELGGWDEHDVVIEEHLKWSPHCRLMTKRPT

>AgBIR-4b

GGDRLATFKEWPKSIPQTPTQMADAGFFYTGKSDVVACYYCGGNLRDWLAEDDPWVEHVRNFSECPYVKLV

>AgBIR-5

IEGDRLATFKEWPKSMPQTPERMADAGFFYTGKSDVVACFYCGGNLRDWLAEDDPWVEHVRNFSECPYVKLV

>AgBIR-6

SARVRSFRNWPYSGIIHPLRLAYAGFCWRGVDDKVHCFDCGLTLGGWLRTDDPWEKHARSSPNCPFIENE

>AgBIR-7

EAARRQTFEAWPHMDYKWVLPDQMAQAGFYHQPGENGNKDRAMCFTCTVCLVCWEKTDEPWSEHERHSPECPFVKGEF

>AgBIR-8

QEDREKSFKHWPFSDDKQCSIQKMAEAGFYWHGTETEIDIAACFVCGKELDGWEESDDPWSEHRKHAPQCPFVKF

>BmBIR-1

VEERIKTFKNGPFNDKNKCNVRNMAEAGFYSVATGVEDADAAKCFLCGKELDGWESTDDPWIEHKSHAAQCAFVQL

>BmBIR-2a

TNRLNTFTNWPALAPVDPIRIAKAGFFYTGQGMEVQCFSCGGKISEWNYGDQVMWRHRRMEPNCTFVVN

>BmBIR-2b

ALRLLSFSHWEDDSVSREALVSAGFYHIGGGRLRCAWCGGELAPFRRFGSLGRPLEVHRMYFPRCAHAAA

>BmBIR-2c

ASRLATFDSWPTDKQQTPKDLSEAGFFHTGTDDQVRCFYCDGGLGKWEAGDAPWTEHARWFPHCGYVLLLKG

>BmBIR-3

EAERKETFKRWPHMDYKWALPARMAQAGFYHQPSPSGDDRAMCFACNVCLVCWEKSDEPWVEHERHSPNCSFVKGEYT

>BmBIR-4a

EEERLKTFDQWPVTFLTPEQLARNGFYYLGRGDEVCCAFCKVEIMRWVEGDDPAADHRRWAPQCPFVRKQMY

>BmBIR-4b

AARLATFKDWPRCMRQKPEELAEAGFFYTGQGDKTKCFYCDGGLKDWESDDVPWEQHARWFDRCAYVQLVK

>CfBIR-1a

EKRLATFREWPSNAAIGASCLAKAGFYYTGNYLEVQCFLCGTMISDWNYGDQAMARHRRKAPNCPFVVDPA

>CfBIR-1b

VSQRLQTFDSWPLTSIIRPEQLALAGFYYLQYKDLVECAFCKGILMNWKVGDDPEHAHKLNFPNCDFYMRE

>CfBIR-1c

TTYEKRLQTFHNWPKNLKQTPEMLATAGFYYQGYDDQVRCFHCDGGLHGWQPMDDVWIEHAYWFPKCGFVLLM

>CfBIR-2a

ELARLESFKNWPCAWMKPEKLAAAGFYYTGESDKVKCFECHVEICQWQPDDSPMVDHQRWSGRCRFVRNIP

>CfBIR-2b

YEARLSTFETWPKAMSQTKEELAEAGFFYTGNGDQTLCYHCGGGLRDWEPEDDPWEQHAKWFDYCSYLLMTKG

>CfBIR-3a

EIVRFNTFKNWTVLYVKSEKLSAAGFYFTGRNETIKCFDCYLEISKWPNGVNVVENHILFSPKCRFARKISC

>CfBIR-3b

YQTRFNSFISWPLVKIQTGQQLAEAGFFYTGQKDKVVCFYCGLILKEWTDYEDPWEAHYKWAAICFYILTIKG

>CfBIR-4a

YRFENRRLMSFTNCVSTVNSRHYLQYYNFASAGFYYIQNDDKIKCFDCNIIISDWKDIDPMAKHQQQFPRCRVVRRIPCGNV

>CfBIR-4b

YYERRLESYVSWPVTIPQKKEDLAAAGLICANDGDIVTCFYCGQALQKWEATDDPKNEHIKWYPDCAFINRLLAE

>CfBIR-5a

RFEIVRLHSFMKSVLKRECAQKYAKNGLYYIDKGNKMKCFECGIIIFGMENKDPQEEHKRNYKCRFIREIPF

>CfBIR-5b

YESRLNSYQLWPVNGLKKEDMAAAGFVCANYEDKVYCFHCSVQMNDWKPHDDPIQIHNIRCPDCKFIKRLVEKR

>CfBIR-6

FSEAARRNTFPKWPHMNYKWALPDQMAQAGFYHEPNATGDDRAMCFTCNVCLVCWEPTDEPWSEHERHSPACPFVKGEYTQN

>DmBIR-1

HRVESYKSWPFPETASCSISKMAEAGFYWTGTKRENDTATCFVCGKTLDGWEPEDDPWKEHVKHAPQCEFAKLS

>DmBIR-2

VRRQTFEKWPHMDYKWALPDQMAQAGFYHQPSSSGEDRAMCFTCSVCLVCWEKTDEPWSEHERHSPLCPFVKGEYT

>DmBIR-3a

VRLATFGEWPLNAPVSAEDLVANGFFATGNWLEAECHFCHVRIDRWEYGDQVAAGHRRSSPICSMVLAPN

>DmBIR-3b

NRLVTFKDWPNPNITPQALAKAGFYYLNRLDHVKCVWCNGVIAKWEKNDNAFEEHKRFFPQCPRVQMGP

>DmBIR-3c

ARLRTFTDWPISNIQPASALAQAGLYYQKIGDQVRCFHCNIGLRSWQKEDEPWFEHAKWSPKCQFVLL

>DmBIR-4a

TRLKTFTDWPLDWLDKRQLAQTGMYFTHAGDKVKCFFCGVEIGCWEQEDQPVPEHQRWSPNCPLLRRRTTNNVP

>DmBIR-4b

TARLRTFEAWPRNLKQKPHQLAEAGFFYTGVGDRVRCFSCGGGLMDWNDNDEPWEQHALWLSQCRFVKLMK

>IsBIR-1

RESNRLTSFARWPFQENCACTPAKMAQAGFYHCPIDNEPDLARCYVCFKELTGWEPDDDPVKEHARSMDCAFVQLRK

>IsBIR-2

REKRRATFVNWPRHAFSNVEALVDAGLFYEGEDDMAICYYCGGALRSWQKDDIPFVEHARWYPECTFVKLSMEPALYN

>IsBIR-3

LVSRVQSFGDEYVQKFKGDPETLAKAGLFYNGFMECDRAVCFQCGGGLYQWDDGDSPFEEHARWYPDCPFVRLSLGDA

>IsBIR-4a

EEHRRRTFDSWPQVSPTMALKLARAGFYHVGRGRTRCFSCGTECGDWRETQGAVERHRTLSPDCAFLRSML

>IsBIR-4b

HRLRTFARWPLDFLDPTDLAGAGFYYLQQDDRVRCAFCRGTIHNWERGDDPLVEHGRHFPCCPFLLDP

>IsBIR-4c

ARLRSFAKWPPASPLRPPDLVKAGFFYIGILDYTKCFHCDGGLCNWERGDDPWEEHARWFPKCQFVLL

>IsBIR-5

DARRATMTNFPREKFQDVESLVAAGFFYDGYMDRVICFSCGGALFHWDEHDDPLIEHVRWYPDCAYVLLCLGPQE

>NvBIR-1

KKGRLETFKHWPFKSENHQCNPDNMARAGFYAIGGKDEPDLAECFMCCKQLDGWEPDDDPWLEHKKHQPNCQFIKLDK

>NvBIR-2a

LRLQSFENWPSEHVRPADLAAAGFYFTKQIDRVRCFECSTEVCRWEQGDDPMVEHQRWGGRCRFIRKLPCGN

>NvBIR-2b

YEARLLTFNDWPSTRVSQTKEQLADAGFFYTGTGDQTTCYHCGGGLKNWEPKDDPWVQHAKWFSTCFYVRLVK

>NvBIR-3

INNPNDSNHQMAQAGFYHQPYSTGEDIAMCFTCSVCLVCWEPTDESWSEHERLHSPACPSVKG

>NvBIR-4

AARRDTFSKWPHMNYKWALPDQMAQAGFYHQPNSTGEDRAMCFTCSVCLVCWEPTDEPWSEHERHSPACPFVKGEYTQNVP

>NvBIR-5a

EEVNRLRTFLDWPANCPVSTARIAKAGFYYTGTAQIAQCFLCGTRVSEWNFGDQAMALHRIANPECPFVLDPI

>NvBIR-5b

YSHRLNTFRNWPIPAIVSPERLARSGFYYLQQADMVECAYCQGVILKWEPGDDPDREHRIHFPNCDFYMRDGA

>NvBIR-5c

YEGRLRTFQGWPSNLRQTPEMLADAGFYYVGAQDQVRCFHCDGGLRNWEETDDAWIEHARWFPKCGYVALVR

>NvBIR-6

YESRLLTFDEWPSRVTQTKEELADAGFFYGGSGDQKTCYQCGGGLKNWEPNEDPWVQHAKWFSTYFFV

>NvBIR-7

STQTALLKLTDDIRMAVDKKKSKEEIADAGFFYGGSGDQTTCYQCGGNLKNWEPNQDPWIQHGYKWFSTYF

>NvBIR-8

PDYMIYEDRLHTYKLWLKESSYRPEILAEMGFCYNCKEDEVYCFCCNGELSQLQPGEDLWGRHAIFAPDCTYLRNRKGEKFINEARTK

>NvBIR-9

TYEDRLHTYKIWLKESSPSPETLAEMCFCYNCKEDKVYRFCCNCELSQLQLGEDLWGRHAILAPDCTYLRNQKGDKFLNEARIK

>NvBIR-10

TYEDRLHTYKLWPKKSSHRSETLAETGFCYNCKEDEVYCFCCDCELSPLQPGEDLWGRHHPPQSRSKMILDLEKVSEPW

>NvBIR-11

YIAYKDRLYTFILWLEGSSQKPEILAEMGLCYNYYENQVYCFYCDCKMSQHGEDLWIRHAILAPDCNYLRTR

>PhBIR-1

DRLETFNEHWKDLKGFKFCTPKNFAEAGFYNSSSVKFPDNVKCFACFKELSDWEKNDDPWQEHVKRGSKCPFVI

>PhBIR-2a

ESKRLKTFTHWPVSFINPKDLAKNGFYFTNVDDVVKCAFCKTQIGFWEEGDDPNKDHLKLSPMCPFLR

>PhBIR-2b

YASRIKTFDKWEAHNIQKPEKLAEAGFYYIGHEDNVICFHCGGGLKDWEKDEDPWVEHARWFSKCRFVFL

>PhBIR-3a

EQRLNTFRDWPGNAAVEPSRIAQAGFYFTGPGLNVTCFSCGCNISDWNYGDQVMTRHRNLSPNCAFVRDP

>PhBIR-3b

SARLDSFSNWPIPFIVTPEALAETGFYFLHKGDAVQCAFCNGIACRWEVGDIPEAEHRRHFPDCPFLL

>PhBIR-3c

DSRLRTFENWPSNLTQQPNVLAQAGFFYVGRQDPDMVRCFHCDGGLRHWAPEDEPWSEHARWFPNCPFLL

>RpBIR-1a

ESERLKTFDQWPVEFMPRHKMAEAGFYYLKKDDIVRCVFCGVEIGKWVPGDDPMVDHMKWSPQCRFVRKLPVG

>RpBIR-1b

DSRLRSYATWPVSLKLKPHILSDAGFFYTGKGDQTICYHCGGGLKDWEETDEPWVEHARWFCKCPYVLL

>RpBIR-2

QAIDKFFFKRDVVCIPTRNCNNNQEMAKAGFFYLKGDCVQCYYCEKELDCWEEEDDAFVEHKNHAAYCPFVKM

>RpBIR-3a

RLRTFANWPEDAAVDPRRIAKAGFYYMGQGLEVQCFSCGGRIAEWNYGDKVMAKHISLDPRCPFVLN

>RpBIR-3b

ESRMTTFATWPIPNVINPVKLAQSGFYYTQVDDKVQCAFCDGLVGNWEYGDEPDVEHQRHFPACTFVSN

>RpBIR-3c

FESRVRTFANWPSTVSQKPEQLAEAGFYFTGNRDKVRCFHCDGGLQLWEKDDVPWLEHAKWFSDCGFVLLT

>SmBIR-1

DRLKSFNKWPYQRESKCTPVEMARAGFYCPDEKFADLAKCFVCDKELDGWEIGDVPWHEHESHSPNCP

>SmBIR-2

ARRETFGKWPHMNYKWALPDQMAQAGFYHQPNSTCDDRAMCFTCNVCLVCWEPTDEPWSEHERHSPSCPFV

>SmBIR-3a

RHRLESFSRWPANAPIEAKKLAKAGFYYKGKDFSVKCFSCSRTIEEWNFGDQAIQKHANLNPNCDFVCN

>SmBIR-3b

ERLKTFRGWPSKVVKPEDLARNGFFYLRDEDKVQCFFCRGVVGQWEDGDNPAIEHRKHFRNCPFMSGY

>SmBIR-3c

ENRLKTYETWPTAIPMLPTALAEAGFYYAGVSDHVRCFCCDGGLRNWEVNDDPWVEHARWFGKCCFLT

>SmBIR-4

RGRALTYDGWNCKFLSPAVMARAGFVYLGYSDAVRCVYCGNCLETWVRNDKPLLEHLRHFPDCEFMKAV

>SmBIR-5

DRLGTFKNWPIDFVSPRSLARAGFIYLDESDTVECVFCEGRIARWVKGDKPMIEHYRFSPYCPLMRIVF

>SmBIR-6

RLCTFQNWANPFVQPRALARAGFVFLDEKDIVECIFCHIRLDEWKMKDIPLFEHYRFSPFCPLTLGE

>SmBIR-7

QRKKTFHNWPAYKCNVDSQAPARAGFIYLGEEDSVECVYCFGRLKQWAYNERPILEHYLWFPYRPL

>TuBIR-1

IEARIETFEKHDGHIVAQITPSKYAEAGFFSYGEHDAVTCYFCAGTIANWLPNDDPWQIHANLFPDCMYVYLKRGR

>TuBIR-2

KDARIATFASHDGHVIKEITPEKYAEAGFFSYGEHDAVTCYYCAGTIANWLPNDDPWNIHAHLFPDCMFVYLKRG

>TuBIR-3

SRFNTFSRWPEQNNQSPSKLAEAGFVYQGRDDIVVCYHCGLTAFNWTCDDDPWIEHVNALPLCGFIYIMRGN

>TuBIR-4

ARIATFASHDGHVIKEITPEKYAEAGFFSYGQHDAVTCYYCAGTIANWLPNDDPWNIHAHLFPDCMFVYLKRGKN

>TuBIR-5

LTSRLSTFTNWTSESISAPELAKAGFLFAGQTDTVVCFHCGIHIRNWIPEDDAWIAHAICAPWCTYLY

>TuBIR-6a

ESVRLQTFYSMLGQSWTACYRDFEELAAFGFYHTSAPRTVACIFCSYRNYISSVPRILLTLHNVQSPSCPAIRGEA

>TuBIR-6b

ETRRFSFISWPHTEMIPHKELAKAGFYYTGDSDTTRCFHCGGILSSWKKNTNVWSVHAYYFPHCHYLYLKR

>TuBIR-7

ELSTFISRLLDSLIKNQNLAEAGFYYTGFADGTVCYSCGVLWDMWTANDDPWVRHAHLSPKCYHIYLHRGS

>TuBIR-8

ERTDQTKFTYQINSMSKDARIATFAKAGFFSYGEHDAVTCYYCAGTIANWLPNDDPWNIHAHLFPDCMFVYLKRG

>TuBIR-9

QSRIDTFSNWNSENISVNNLAEAGFVYSGKADIAVCFHCGVHVGNWLPGEDAWVSHAKFAPWCTFLYIRKGF

>TuBIR-10

FEKNRVKTFKNWPHKTGKISKENMAKAGWFKCLPESADDAVECFCCLKQMEGWSSNDDPWKEHLDHSPDCEFAKTGV

>TuBIR-11

DQRKNTFSDWPLKDIISPQKLAEAGFFYSGLFDIVHCYHCDGALQNWQIGDDPWAVHANAFQNCAFIYI

>TuBIR-12

ESRLSTYETWTNENKSPSELAQAGFLFIGETNTVVCFHCGIHIQNWIPDDDVWVAHAICSHWCTFLYIKCG

>TuBIR-13

MKRLESFESWKRDDICRTELADAGFVYYDVDNKVICYQCGCIVNDWFENADPWLIHAQSSPFCFHLY

>TuBIR-14

ESRLATFKDWPNPAITPLELAEAGFFYSGQSDLVTCFHCANSLLEWIKNDNAWVNHALFSPCCTFIYIMC

>TuBIR-15

CRVASFAKHDGHVVKEITPDSYAEAGFFSYGEHDAVTCFFCAGTIANWLPKDDPWELHALHFPDCMYVYLKR

>TuBIR-16

ASRLSTYGDWINTNISSTALAEAGFYYTNKLDIVACFHCGVHICDWLSAENPWVSHAKFSPWCTFIYIR

>TcBIR-1

ANRKSTFKKWVFSDKVMCNAAKLAEAGFIFVGNSLEPDSVKCFLCNKSLDCWAEDDDPWTEHIKHSPKCSFAKKNK

>TcBIR-2

EAKRRETFTHWPHMDYKWALPDQMAQAGFYHQPNASGDDRAMCFTCTVCLVCWERTDEPWSEHERHSPSCPFVMGE

>TcBIR-3a

ADRLSTFIDWKSSAVTPEALAKAGFYFLNNPSKPDLVKCAFCKAEICSWEQDDEPLSEHVRWSPNCPFAKEKSQNL

>TcBIR-3b

ARLESFATWPSSAKQSPETLADAGFYYRGVEDHTICFSCGGALRDWKDEDEPWEEHAKWYPRCEFLVASKGHD

>TcBIR-4a

EQNRLDTFEEWPQDAAVSPPRIAKAGFFYTKHDVTVECFSCHLTISEWNYGDQVMAKHKTLNPSCPFVLNPTT

>TcBIR-4b

AVRLKTFAKWPKPHIVAPERLARAGFYYLNTGDNTKCAFCKGVVRAWEPGDDPDQEHKRHFEDCPFVLTE

>TcBIR-4c

ESRLRSFATWPPDLIQTPDILSQAGFYYEGMGDQVRCFHCDGGLRHWDPQDDPWTEHARWFPRCSFIKLV

>DpBIR-1a

DIRLRTFNEHFSATFLSPLLLAKAGFFYVGVDDQVQCAFCRGVVRDWEINDDPRREHQRLFPSCAFILG

>DpBIR-1b

TLDARLKSYNNWPSHLKQTPRAMALAGFFHLGTNDHVNCFHCGSGLRNWEPEDDPWLEHARWFPQCRFVML

>DpBIR-2a

ALRLATFQGWPLEYLSPRDLSRAGFFYRGLADQTQCAFCCITISQWEAHDDPMAEHRRHAPNCPFVLQL

>DpBIR-2b

ARLKTFDDWPPGLEQRPPQLAEAGFYYMKTGDHVKCFCCDGALRNWEPKDDPWVEHARWFSRCNFLVSV

>DpBIR-3

ARLSTFQRWEQSAPNSPTPQALSSAGFIYRGVGDHTQCFTCLVVLSQWHIDHDPDLEHRRHSPSCEFVLNR

>DpBIR-4

ENRLSTFFKNGQGLSGRWPFLEDCNCTPEKMATAGFFWCGSESQPDLVRCFVCLKDFEGWEPNDIPKDEHKRLSPQCPYV

>DpBIR-5

ASRRETFASWPHMNYKWALPSQMAEAGFYHQPNTPESDRAVCFLCNVCLICWEPSDEPWSEHERHAATCPLVKGDY

**K. I35 Timp.**

>AgTIMP-1

AETCSCLPQHPQTAFCDSQYVIVAQVLRKTASKNEAMDAYKIAIKKEYKMSDEARQLLNHGKLYTSTMDSACGIKLKPSTLYAIAANSEQVGLCDFIRPYDELSLVEKRGLAGVYRKGCKCKINHCWDDKCHQRLGSCNWTPFAPKGICETSYGSCVPAGVTKKNGAPIKCHWRR

>BmTIMP-1

ASACTCALEHPQTHYCKSDFVIVGRVQKTFRGREDYDIYKVKIRNVFKATDKAVAALRSGRLFTPPHESLCGVSLQPRETYVITGQVLHLEAHIYLCGYIAKWREVTPRQRKGFRLLYKQGCTCKVHETRRRTKSPNTCVTNYNECYERHGICLHDRERRCHWTRAP

>CfTIMP-1

AACSCMQAHPQTKFCESDFVAVIKVKKVLPVNEYEIAYKVKINRVFKSNSKADIALMQNLLRTPSSSSMCGVTLQVGETYVLNGRIVSGQALISSCGLSIRWADTTSRQRKGLRQLYQPGCVCDILYTHWRRKGAVLESSGGKRCLWESTPGPQDCQEKHGVCIAASGSCSWMPSV

>DmTIMP-1

RPADACSCMPSHPQTHFAQADYVVQLRVLRKSDTIEPGRTTYKVHIKRTYKATPEARRMLRDGRLSTPQDDAMCGINLDLGKVYIVAGRMPTLNICSYYKEYTRMTITERHGFSGGYAKATNCTVTPCFGERCFKGRNYADTCKWSPFGKCETNYSACMPHKVQTVNGVISRCRWRR

>IsTIMP-1

APRSHGIELLRLFRGGPKAMWALAEGLLWTPGNDGLCGVSLHENVRYLVTGSLHGAKPWVSACGFVRPWNSLTRKQRKGFQRLYQQGCRCSVRLQPGPNTQCEWETAFRGVEDCQEQYAMCVPQANSGCTWLGG

>SmTIMP-1

CAVGDTCTCFPVHPQAHYCNSDYVLLVKVNNSTIGNQTQATHRMIDVKIKKSFKANEKVNFAIKNGQIWTPMNDGVCGINLKPNAKYLITGKVEGGKAFISTCDYYQEWSNLTPKQRKGFKLLYKLGCECKVAYCPLAKRGHKCNVNANTCSWTTAFDKDGDCQGHYSICMRQITGYCQWN

>TuTIMP-1

TSDACSCVYSHPQEHFCSSDFVVTLSIQGDPKQLHQYNYLRYPVKVYKIYKGMDKTSVRKGFIYTGSALSSCSPTLAKNTTYLMTGRIVNGKPFVSICNFISEWSSLTYRQKKGFRRNFGRSCGCKVTDAGSYAFYSGY

>TcTIMP-1

QYSNACSCMGYHPQTQYCRADFVILARVKRSTVLNSLKVYKVRIRKTYKGSDKATVALKSGRLLTASDEAMCGANLEAGRVYAISGQVNSLKAHINLCGMAIPWRNLTRRQRKGLKSVYKKGCDCRIEYCAGRKCHKTPDTCLLTNRFCHPKQAICLRQKSRKCMWGR

>AmTIMP-1

VACSCMNSHPQTLFCNSDFVILVRVKKMTNVNEFETAYNVKVNKFFKANKTTYPALRKNILWTASSDSMCGAQLKLGETYVVSGRVIYGDKAHISSCGIAMPWRFVTSRQRKGFRHLYHSSCMCKVRYTPWWIKGITLENTDGTECLWESRPGPEECQKDFGICMYRESGCYWTPS

**L. I39 Alpha 2M.**

>ApA2M-1

HKSYSVFIQTDKAIYKPGHKVQFRAIVLNYHLKPTVTGALDIYITDGQGNRVKHWSRALTTRGVFSSELQLSESPVLGDWNIVVTVLDQVFHKSFLVAEYVLPKFEVTIEVPEHTTFKQSVVSATIHAKYTYGKPVKGEATVSVYPEYYSDLIQPIYQNPLRKVVPINGKTVVQFDVVKDLSLNDEFRRIITFDVTVEEALTGRSQNTSANVMFHNHKYKMDLIRTSEYFKPGLKYTAFIKMSHHDGTPVYDDRNPVKVWHGFSHETEKLDESKHMLPRNGLIPLVYYPQINASVIVIEAEYLNQREALSTILPAHSTSNTFMQATVLTERPTVNKDVEIQVNSTESLQYITYQVLGRGDVIVASTVQIPNAGQHTAVIRFLATYAMAPTAHVIVQFVKDDGEVVADAIDVELDGVLQNYINVDVSRDEVEPDTSVDINFEAKPNSYIGVAGIDQSVLLLKTGNDISHDNVLDELRTYDNGEHSNYMPYLRESLDRRSMFWWPGSYTAHQAFDKSGATILTNAFVNDYNPWVYYRSNVMDDQEMMPIPSTVSETSSSIKVRKNFPETWLWESTESGQDGRASMKSTVPDTITSWVITAFSVDSLYGLGLLDSPKKLKVFRPFFISVDLPYSVRRGEYVSIPVVVFNYLSKDVTADVTLENIGQFDFADTSNDVRDSKLELYKRKSLTIKSNSGSPTSFLIQTKDLGYISIKLTATSKLAGDAIEKKLLVKPEGETIYKNKAIFVDLRKESLFEKNITLEIPSNIVPDSEFIEIGAVGDILGPSTMNLASLIQMPFGCGEQNMLNFVPNIVILDYLKNTKQLTTAVETKSLKYMETGYQQELTYRRSDGSFSAFGSADASGSTWLTAYVVKSFRQAMPYIPIEEKIIIEGLQWLSNNQANNGSFPEVGYVSHSDIQGGSSKGLALTAYTLIAFLENQKATPVYRNTINRAVEYLVRNLPGVEDPYAIAICSYALHLADHPEKNVAFNLLELKANTVDGKKWWKRMDRANDKKNPWVHEPNSVDVEMTAYALLTYLQRELVEDGLPILHWLVSQQNDQGGFASSQDTVITLYALSQMAERITPGTLKLSATFSYMKNGQSELKVTKDNAMVLQLIELPKRTRILNVTATGTGLAIIKVSYRYNVNVTGAWPLFSLDPQVDKNSNANHLQLSVCSGFRGGNDSNMAVMEVTLPSGFTVDNDALPSLRLSNNIKRVETKDGDTVVMLYFDKMMAEEYCPTISAFQTHKVANQKPVPVTVYDYYDQSRRARVFYAPRKAT

>ApA2M-2

LVEGVEHTLTANEEAEVVVLSSCPCNRYVNYVVTTEGHVAIWQKYKPVVHMTKIMDQVDICRFNLTFNVDPVMAPTSHLLVYYTTEKGETINDVISFNVKQTDPKVKISLKDNKNNWYPEELMELNLMAEENSLVCLIGGRGTENISPLRNMDDDTDLLESGLLFLEKRLDGKVTTSRQSDLYPRHHSFLDTFSMDQLWTWKCVNFTSDVIKKGMNIHAPSKPGHWKLRMLTVGSTGLKITDTLDIKVTSTLEVDVRTPVEINVGETVQTDIYVANNVNSCMDVNALLSLSEGAVFSSSNQPFVAEKMRLGAYGATSLIVRITALKEGLKNLTVDISGYVSEKCHLINDNKKSEDLELSNSTASIVVKSVPIYVHPEGIQHQTTDNAYFCANEQLIVSTTSDFRYQHINAPKNRDGIVFEIQAKKSAHILLSQERKPTTLMYQIVLGDLDNTISWIGRGKHGNGVHLTSRNTPGILSEEEPRTFWISWEKGVLAFGYGQEIHQYPLLKWNMDKKIKINHIGFATILGTTGQFRVWNYNDEAGFSQVLHLETPNTMISGSESGTLVVTGGLNFPFFVQNEPKFSTSLVSFLSTFTPLLMSEHNNNGTEEKSLVDLLSKSIPVLLSYQNSDGSFGDHPNVPCYWCDIRVLEILWRSQSHVGVDSDLIKGLKTWIQKQVFEDFSTVVGQSDMEINKIICAADTLATLMELGIESEIDSKIANHTKSYLEQHLDNVVKPYPLAITSYALMVSNSYFTKKALTKLQSLSTNQESEFGWPKVHPTSDWYDDVVPQKKGENISIDEFKASLYCLMIYSARRELKSSEPIVRYLYYRTKILDTYPELAYLAVKAFAMYDKIGSDPHRKLTISLATSGMELTDTLELDPSTKSQYLHLPSLPTKVFVYATGAGCSTIQGRVLYSTYTTAENDKKPFDLWSGVTDVIQPSKGFTDEIYGHAITLRLKTCFRMNNETEDAIRLEVKLFSGYYFDKISSASVSDVHHDSHSNHIWFVFAKVKSSCIVCVSYTAKSIQKVTGLRPAVAKVYVVSRPDQSSYKLFH

>AgA2M-1

FCSVLIQTDKSVYKPGDTVRYRVLVLDRSMKLLPAGDSGMMVYIRDGKGNRIKQWSNASLGECGVFQAELTLSTEPVLGEWTINVEVVGLKESKTFDVDEYVLPTYEVTVESPGYTFLDDELLKVVVNSKYTYGKPVAGELTVSVKLASSMCFRREPTETSICQKVLPIDGKTDVEFNLKEILSSKTYIRELTIEAEVCETLTGRTQKGSTTVQLHDERYQVRMIEESSYFPGLPYNAWIQVTNLDGSPVQDGAKEVEIVLRNYNIDLHKQSSTLDDKGMAQLNVKLDELDFDYVSVEVKYRGKDYYVQGITKPRDYEEALMRVRLSEKEPTAGKDLTFDVACTKPLQCVSYSLLARGELLAGGAVKGSEASTTISITIPSTFAMVPRAKLLVHYISSAGYIVSSYDTVEFKRVFENQIQLTLSKDELKPVETLDIDIRTEKDSFVGLLAVDQSVLLLKSGNDISRDEVVQQLEMYESAQNYHWDAYSTSDCQSVGAVLLSNRFIPRDIFPQARLFACSTSAGGFGAAPMMAACKMKGVIMESEMATAPVNEPTVRSKFPETWIWESISKCKEMESIRKIVPDTITSWIITGFSLSKSHGLGLVDNPSKVNVFMPFFLSIDLPYSVKLGETIRIPVVVFNYMDEDQLADVIFYNNDDEFEFVSDTKDQKEKHRQEQITVPRGTGKTLTFVLKPTKVGHVTLKITAKCALAGDGIERQLLVEPEGLPQYINKALLVDLRLVKEIKQPFEVEIPVDAVPDSTNVEVSVIGDVLGSSIENLDSLIRMPFGCGEQNMLNFVPCIVVLDYLKACKRLTVEIESKAKRCMEIGYQRELTYKHQDGSFSAFGESDKSGSTWLTAFVAKSFQQAAKHMTIEEDVIDSALGWLSKVQTADGAFPEVGTICHKDMQGGAGSGMALTAYTVIAFLENPKLGEKYKASVDKALTYVKEHISELDDVYAHALAAYALQIADHPLKNEVYASLLSKSNKQGDIQWWSKEIPEKNDSNCCWWYRPCSVNVEMSAYGLLATLEASSAGLEGLPIMKWLVSQRNDKGGFESTQDTVVGLQALSKMAAQLSSSEADMSLKVIITGEQEKCLQVNGGNILVLQKHELAANTRKLEMIATGTGCALFQLSYKYNIKDVDNSPRFTLKPEAKQGSIKSCIDLSITTSFIPKEDQAVSNMAVMEVDMPSGFIVESDTLKQLKQHEMVKKVETKRSDTTVVLYFDNIGEEAVHLQMSAFQKHEVENAKPANVIIYDYYDNTRCARSFY

>AgA2M-2

FSVFVQTDKSIYKPGDTVRFRVLVLDPNTKPLQKADNISVHINDAKANRIKQWKEGKLVKGVFESELTLSTAPVLGAWTINVEVLGSKHNKVFEVDEYVLPKFEVTVESPGITTFKDGKVKAIIRSKYTYGKPVKGEATVSVSPEFQFHYVQPFAKDVITRKVIPIDGKGSVEFDLREDIHLEGDYSRNIVIEAVVEEELTGRKQNASAKVMIYDRRYKMELVKSDDNFKPGLPYTAWLKVSYQDGAPVQDQTNPVEVKQSSFESTTSVQNYTLDQNGMAKLEINTEVNSSYINVVGVYLGQEFYLHGISKAESDVDSYIRAQVLTEMPLVGKDVLVEVTSTSPMKYFTYQLLGRGDVLLSNTIAVPESKTQSFKFPATFAMVPRAKLVVYYIAPNGDMVSDSKVITFDSELQNFMKVSLSKEQSKPGQDVEISISTNPDSYVGLLGVDQSVLLLKSGNDITKQQVFSELEKYEERSYGFYRRKKRFAWNPHAEHRDFSTVGAFVMSNANDPPQIHPVFFSLPALAAPPGVIITSARPFVAATALSASSPVASDPIVVRRTFPESWIWESDEGFSGEKTLQKKVPDTITSWIITGFSVNPIYGLGLTQQPRKLNVFLPFFVSTNLPYSVKRGEVVAIPIVVFNYMEDDQTAEVVLHNDEQEFEFADVENEVVESNKVELFRQKRLDIASNTGKSVSFMVKPKKLGHITIKVTAKTKIAGDAVERQLLVEPEGLPQFINKAAFIDLRAAPELTKTFEVEIPKNAVPDSTRIEVAVIGDVMGSTIQNLDSLIRMPYGCGEQNMLNFVPNIVVLDYLKATNKLTANIEAKAKKFMEAGYQRELGYKHRDGSFSAFGENDKSGSTWLTAFVARSFKQAANHITIDEGVIDKSLEWLSDHQAPNGSFPEVGVVSHKDMQGGSGSGVALTAYTLIAFLENINLVDKYKNTINKAIDYVYRNTESLDDTYALALAAYALQLADHSSKGLILSKLDTKATTDSDSKWWHKPIPETEQKNPWYSRPNSVNVEMSAYGMLAFLEAGLDTDALPIMKWLIGQRNDKGGFQSTQDTVVGLQALAKLAAKITSPNNDVTLTAKINENQEKRMTVNAENGMILQKFELPSAARNIEIQATGSGFAVVQLSYKYNMNVTGEWPRFVLDPQVNANTNPDYLHLSVCASFVPSAGQNVSNMAVMEVGFPSGFTADSDTLPSLENMPFIKKVETKDGDTTVVLYFDSLDQRELCPTISAFRTHKVAKQKPAPVVIYDYYDNSRIARQFYDGPKASLCDICENEDCGEACSIRSQKQRSSDSPSRQPTVEGTMQSGSQTVSVSFFTFLLATLLVRM

>AgA2M-3

SKSISGLIQVDKPVFKPGDTVNFRVIVLDTELKPPARVKSVYVTIRDPQRNVIRKWSTAKLYAGVFESDLQIAPTPMLGVWNISVEVEGEELVSKTFEVKEYVLSTFDVQVMPSVIPLEEHQAVNLTIEANYHFGKPVQGVAKVELYLDDDKLKLKKELTVYGKGQVELRFDNFAMDADQQDVPVKVSFVEQYTNRTVVKQSQITVYRYAYRVELIKESPQFRPGLPFKCALQFTHHDGTPAKGISGKVEVSDVRFETTTTSDNDGLIKLELQPSEGTEQLSIHFNAVDGFFFYEDVNKVETVTDAYIKLELKSPIKRNKLMRFMVTCTERMTFFVYYVMSKGNIIDAGFMRPNKQPKYLLQLNATEKMIPRAKILIATVAGRTVVYDFADLDFQELRNNFDLSIDEQEIKPGRQIELSMSGRPGAYVGLAAYDKALLLFNKNHDLFWEDIGQVFDGFHAINENEFDIFHSLGLFARTLDDILFDSANEKTGRNALQSGKPIGKLVSYRTNFQESWLWKNVSIGRSGSRKLIEVVPDTTTSWYLTGFSIDPVYGLGIIKKPIQFTTVQPFYIVENLPYSIKRGEAVVLQFTLFNNLGAEYIADVTLYNVANQTEFVGRPNTDLSYTKSVSVPPKVGVPISFLIKARKLGEMAVRVKASIMLGHETDALEKVIRVMPESLVQPRMDTRFFCFDDHKNQTFPINLDINKKADSGSTKIEFRLNPNLLTTVIKNLDHLLGVPTGCGEQNMVKFVPNILVLDYLHAIGSKEQHLIDKATNLLRQGYQNQMRYRQTDGSFGLWETTNGSVFLTAFVGTSMQTAVKYISDIDAAMVEKALDWLASKQHFSGRFDKAGAEYHKEMQGGLRNGVALTSYVLMALLENDIAKAKHAEVIQKGMTYLSNQFGSINNAYDLSIATYAMMLNGHTMKEEALNKLIDMSFIDADKNERFWNTTNPIETTAYALLSFVMAEKYTDGIPVMNWLVNQRYVTGSFPSTQDTFVGLKALTKMAEKISPSRNDYTVQLKYKKSAKYFKINSEQIDVENFVDIPEDTKKLEINVGGIGFGLLEVVYQFNLNLVNFENRFQLDLEKQNTGSDYELRLKVCASYIPQLTDRRSNMALIEVTLPSGYVVDRNPISEQTKVNPIQKTEIRYGGTSVVLYYDNMGSERNCFTLTAYRRFKVALKRPAYVVVYDYYNTNLNAIKVYEVDKQNLCEICDEEDCPAECKK

>AgA2M-4

TTTALVQLSKPIYKPGDVLQFRVIVLGGDLKPPAPSVTATVIVHDPQRNVIRRWTAVSLQLGVFEEQLQIGTVPLLGRYTITVTVTGANEIVSKTFDVREYVLPAFEVAVKARAVPLEKHQRLNLTLSARYYTGQPVRGVATVELYLEDDKLDQRRVVGVYGAIQLDLPFNEHLSVYDDSQDVRVHVAFTQDETNRTIYKEQRITVYKLPYRVELVKEQPEFRAKVPFDCQLRVRYQDGTPAKGAAFEVKVEGAYTTDRRVAYTSDAAGVIKLTLQPEASSESIDITVRVFAREVFLEKSTLKRTGGMNFVSRTGKRWQRGAALRAHKQAVKIGKPFKLKVTCNEELSFFMYYVVSRGVIVDSAFLQPKKVTEHFIEIEASDQMVPRSKVIVVTVAKNVVLCDFVDIDFEDLRNNFDLQIDRTEIRPGDQLQLNMRGPPGAFVALAAYDKSLLQYSNNHDIFWEDVWGVFDKFYSVERNEFDLFHSLGLFARTMEHITFDKANDQTARDGSSSSKNGPSNSQASFRTNFLESWLWKTDKIGSSGSATTKESVPDTITAWHLTGFSIDPVYGLGIIKQPLQLTTVQPFYIVPNMPYSIKRGELVELQFIVFNNFPKKYKASVTLFSVDNQTEFVGRPATETSYTKSIEASPDTGVPVAFLIKARKLGEMTVRVDASIEPAKDSIESVIRVIPESLVKREMISRFFCHNTYQNQSFVLGLDFDRKADAGTRKIDFILTPNILTSVMDNLESLLSVPTGCGEQNMMRLVPIVLVLDYLTSIGSADKQLTAKAIGLLRAGYQNQMRYRQPDGSFGLWEKSGGAVFLTAFVGKTLATAAKYISEIEPSMVEQAFDWLAARQHSTGRFDEVGPVFHRDMQGGLRQGIALTSFVLIALLEQPKVATKHRAAIEKGIDYVTQTLGSIEDSYDLAIATYALLLQKHSSGERFLEKLIGLSTVQQNGTERFWARDAHGIETTAYGLLSFVLAEKYVDGTSIMRWLVKQRYTPGSFPRTQDTFVGLKALTKLAEKISPSRNDYSVQLRHAGRKKEFRVTSQDIGTLQNAQQGVDETAQLELHVAGIGFGLLQVVYEYGVDLRNFTAQFVLELQKSVTNANHQLQLEVCSSFTPQLSDGRSNMVLVEVNFPSGYTVEQRGQPITGATKHNPIQKTEVRFGATSVVVYYNSMGPERNCFTITAYRRQKVTLKRPAYVLV

>AgA2M-5

ISGLIQIDKPVFRPGDLVKFRAIVLDTELKPPARIKSVNVTIQDPHQNKIRGWPAAKLYAGVFENDLQLAPAPLLGVWNITVQVGEEQLVFKTFEVKEYVLTSYDVQVMPSVMPLVEHQTLNLTIVANYHFGKPVQGVAKVELYLVDDTLDQKKELTMYGMGQVELRFNELLELYEDQQDVRVKLTFTEQHTNRTVVKEQAITVYKHPYRAQLTKESPQFRPGTPFKCTLTLIYHDGRPAGHVPFFVNVEGEDVDHQQTYTTGRDGTIKLLMRPTELTETIDITVSEDNSEFTYTERIEKVHADTNVFLKLELKSPIKLGKLIRLMVTCNERMTFFIYYVISKGNIVDAGFVRPNRQTKFMFQLTASEKMIPKAYIFVATVSQDVVVWDSLEIDLKQFSNHLDIIIDEKELKPGQEIELLLKGRPSAYVGLAAYDKGLLAYSKQHDLFWEDVMQVFDTFHATDQNEFDVFNSMGLFARLSGGNRIGASPTTTERFGSAASRPISRLVAYRTNFLESWLWQNVSIGRTGSRTVHEVLPDTTTSWYLTGFSIDPVYGLGIIKKPIEFITVKPFYIVDSLPYSIKRGEAAVLQFTLFNNLEAEYIADVTLYNVANQTEFIERPDKDLSYTKSVSVPPKVGVPISFGVKARKLGEMVVRIKASIMTGKETDAMEKVIRVIPENIMFEKTETRFFSMDEYGKQEFNMQLDIPKNISTVQIKCRISSNLLSPVIHNLDSLLDVPSASGAPSMINFIPPLVVLDYLKAVSSTTTHLIEKATGLLRNGYQLELKYRQRDGSFGNWRDSKGSVFVTALVGTSLEAASKHITEVDLTLVDRLFEWLAAKQHSSGRFDEEQPITYYSLQGGSRNGIALTSFVLIAFLQNTKASAQHRSIIEKGIQYVANQLESIADVYDLSLATYALMLADHRQKSSALNKLIELGIATNETRYWPRDTASIETTAYALLSLVHAKRYADGLMVMHWLVNQQSATGSFPRTQDTFVGIRALAALSEAIAPQKNDYTAIVLHGKARKVYKVAASEADQEYHDVLPGDSKLVRFSANGRGFGMFTVAFQYGIDVRNIEHGFSLRLVDQFSNEAYTLQLQVCTSFSPQLMHTRSNLALVEVNFPSGYVVSRKSLVDETRRNPFKDVEVRYGQTSLVIYYETLGPEENCFSVTANRLFRVAFHRQAYVMVHDTYDEKFRAIKFYQVPH

>AmA2M-1

HDSLLTFVETDKPTYKPGQDVKIRILMLMHDLKPWQKSIPEVWIENPSFVKVKQWTNVSTENGMAQLTFPLSPEPSLGSWHIKVMKKKPYPNLIHSTTFKVEKYVLPKFQMTINSPQYILANVENVTWNICVKYSYGKPVKGNLLLKLTPQTPSWTRLPNLPAIRYETKLDKGDGCTDFVLSGSVLGLAHWKMDPNNIVLIAEFTEAGTGIVETTISRTVVLHEALKLEYEHYTPKYFKFGLPYHGKLRVLRYDDTPAPNEKIQICLKVRGKIEWEKDVVDCRDFRSSTDGFVDFVVPPQHKNIVLLSFVATAVDYPTTYYSPEQRWRVFMNQPSTSITVNPWYSPSDSYLTVARGNQPIVCGEKYSFNVMYTTSSNMNETISFHYSINSKGSILIYGHVKHKPNRDTILNYFEFHNLLGTIESSANKTNKEAIVHRFPLSVKVTPSMAPVSELLLYYVRSDGEIVATTYTIEVGHCFENKVKSTWHTDAQIPGSPTQYHVEAAPRSLCAISAVDKSTLFLSKSESNLMSSTQTFDALKRFHPTPKFYFPWENSRCKSAIGPEEMKEEINHLPQFLRSKRQTITYSKRVNYVDAVQAFVDFGVIVMSDLVLETRPCPWLFMEYTALSRQYISTNEYMSMKDNSEFAVAAAAMDSGIGYVDQNQAQMATLRSYFPETWLWELVPIGEEGKITIERTLPHTITDWVGYTTCISPTHGLGIAPPTTITAFQSFFLDYNLPYSIKRGEIMRFKVSLFNYMHHSLPVKIKLEEMEKIDLHLSEPTASFCVKPRDNIVHEYILKPRVIGEVNITVTAFVDIDYPEPCGSETVIFTQDVIVKPILILPEGFPVEETKSALICPKDSSDDSSFMWELTLPKDAVPDSGRAYLNLIGDILGPALENLDKLIKLPKGCGEQNMILFVPNNHVIKYLDAMRINKPDLRAKAIRNMEKGYQRELKYRFMDGSYSAFEEGESSIWLTAFVLKSFAQAASLIHIDKYVLESSVSWITMNQLEDGCFPVIGTVFHKSMKGGLQEHGSSSALTAYILISLLESGVPLSPSVVNDAQKCLEKGMNNDDLYTTVLTTYVLALLEHPKANSSMKSLMNRATRYKNLIWWEDKSKPSIGLSIEMTAYVILTLLKLGEENLSEALKAVRWISKQRNSEGGFTSTQDTILGLEALTKYAMIVHHNNITDLSVLVTASKEVDDVYKLQDENRVILKQIRLPILPTIVEIFAQGEGCVLIQSNLKYNVASSTGSDAFDLSAEVRSVGYGNECSLQEITICSRYKMADEESNMALLEVGIISGYVPDRASLHSLLDPSSKVKLFEEDQDIVTIYFNKLTGQKTCISFRIIQEYFIDHLKPANIKLYDYYQQELTVSTNYKIPS

>AmA2M-2

LKSYSVFIQTDRAIYKPGNKVMFRCILLNSRLRPTLERLVDIYITDGKGNRIKQWIRPPVTHAIFNGEIELSEFPVLGTWKITANVGDQTFEKDFEVAEYVLPNFEVTIDASKHFTFKESKITATIYAKYTYGKPVKGEATITAYPDIFSGVIQPIYQQPVRKVIPINGKVIVDFDIYNELKLTDEYERPVMLDVTVEESLTGRRQNTSTHITLHKNKYTMDLIKTSEYYKPGLKYTAFIKITYHDGTPVRDNKNPVIIKYGYSYDNQSIYTNITGMLDENGMVKLDFYPPKTNHNISYPLNIEAQYLNLYEWFPSTNQAMSQNNEYIQAILKTEKPMVNQDIEIEVNSTIPLKYINYEIFGRGDILDAGSIYVQNKHTTNFKFLATYVMAPTAHVIVYYVGNDGEVIADALDVELEGVLQNFVDIKMAPKEVAPGENVNLIITSKPNSYIGLLGVDQRSLLLKSGNDISYEQVYKELKSYDNAHESPYTNSIFDRYLWSPGSATAKDVFRESGAIIITNGYVHENLPIKQPGILEGRITGSPHGASTLRPDLGPPVMHKLATRPPLAGPYAFSRIPPPVWNKPRVFLMHDILNTWLFTNFSAGHEGKNELKRNVPDSITSWVLTAFSVNDVHGLGLIKEPQKLKVFRPFFIAMDLPYSVIRGEIVAIQIVVFNYMNKNVVAEVLLTNEGQFDFAEISNEIQDVPKLELYRKKKVEVKANSGSSISFMIIPRELGYITIKATANSILAGNSVNRKLLVKAEGETQYVNRATFLDLRNTKSTSINVTIDIPKNAVPGSEHIEISAVGDILGPSILNLANLIKMPSGCGEQNMLNFVPNIMILNYLKNTNQLTQAVQNKALRYMEIGYQRELTYRHNDGSFSAFGMSDSSGSTWLTAFVAKAFKQAAAYIPIEDRIIDEALQWLSNNQAPNGSFPEVGKVSHRDMQGGAAKGLTLTAFTLIAFLENANTNGRYRNTINKGIDYIVRNINDLDDAYALSICTYALNLAKHPYENTAFNLLESKAMTKEDIKWWNKPIPVNDKNPWYYSLPRSIDVEMTSYSLLTYLERNLIADSIPVMKWLVKQRNAEGGFASTQDTVIGIQALAKLGEKLITKNNDIQNNNISVTFAYEEGQNQMNINSDNSMILQKQMLSRKTRLVNITATGNGFVLVQVTYQYNLNVTGAWPLFTLDPQVDKNSNANHLQLSICSGFVPTKEANESNMVVMEVNLPSGFTVDKESLPSLEGDTIVILYFDEMSRQEYCPTVSAFRTHKVAKQKPVPVTIYDYYDSSRRARVFYEPRKATLC

>CfA2M-1

IVGPTDVYIIDGDGNRIKQWNRPPVTHGIYSSELELSQSPVLGNWKIIANVGDQTFEKEFEVAEYVLPNFEVTIDSPKHVQFKESKITVFVHARYTYGKPVKGEATITAYPDIFSGVIQPIFQQPVRKVVPIDGKAIVDFDILSELRLTDEYERPVMIDVVVEEALTTRRQNTSIQITLHKHKYTMELIKTSEYFKPGLKYTAFLKLTNHDGSPVQDTKNDVLIAYGYSYDRSDYFNITKMLDQNGMIQLDLYPPKQKDNISFPLNIEAQYLNLHEWFPTTNSATSLSNEYIQAVLKTEKPMVNNYVEIEVNSTAPLKYISYEVLGRGDILDAGSIYVQDKYTTSFKFLATYVMAPTAHIIVYYVKTDGEVIADSLDVELAGVLQNFIDLKVTPGEVMPGESVNLVISAKPNSFIGLLGVDQRSLLLKSGNDISYEQVYKELQSYDRVKASPYTDSFFGRPLWSPGSGTADDVFRKTGVVILTNGLVHESFPMLYYRANLDEALYASAAGFSGPAALPPEKVKVRKNFPETWLWQTLDAGYQGKAELRRNVPDSITSWVLTAFSVSDAHGLGLIEEPRKLKVFRPFFISMDLPYSVIRGEIVGIQIVVFNYMNKDLTAEVLLTNEGQFDFAEVSNEVHDVPKLELYRRKKVDVKANSGSSVSFMIIPRELGYITIKATANSVLAGDSVEHKLLVNAEGETQYKNEAVFLDLRNVENTGANITINIPNNAVLGSESIQISAVGDILGPSIPNLANLIKMPFGCGEQNMLNFVPNIVILNYLKNTNQLTQAVQSKALKYLDTGYQQELTYRHTDGSFSAFGMSDPSGSTWLTAFVVKSFKQAAEYIAVEDRIINEALEWLSNNQASNGSFPEVGKVSHRDMQGGAAKGLALTAYTLIAFLENEDSVGKHRNTINKAVDYIVRNMEELNDTYALSLCAYALNLAKHPYETSAFNFLESMAMKKQGIKWWSKPIPKDDKNPHYSLPRSVDVEMTSYALLSYLRRNLVADAIPVMKWLVKQRNTEGGFASTQDTVIGLQALAKLAEKLSKDTSSVRIAFKYGRDGQGYMNINSGNSMILQKQILPSKTRFVNITASGKGFVLVQVSYQYNLNVTGAWPLFTLDPQVDKNSNPNHLQLSICSGFVPTKEANESNMAVMEVSLPSGFTVDRDSL

>CfA2M-2

ITFVETDKPVYKPGQDVNIRILMLKHDLKPWKKTIPKVWIENPSEVRVAQWTNVSTENGMTQLKFALSPEPSPGAWKIKVEKTRSQPQLIHTTVFEVKKYVLPRFQVTVTSPGYILADAENVTWNICAKYSYGKPVKGRLLLKSTPQIPIWRRKPNLPEIHYETELDSSDGCTEFVLSGAVLGLAQWKVAPNNIVLIANFTEAGTGIVETTISRTVVVHQALKLEFLPYTPKYFKLGLPYHGKLRVSRHDDTPAPHEKIQLCVRVRGEDDWLRVVVECRNFTSSSDGFVDFVVPPPHRNIVLLNFIATGVDYPTKYYSPDTRWRVFMDQPSAHIVVNPWYSPSDSYLAVARGYQPIICGEKYSFNVMYTVPAKSKTNESISFHYSINSKGDLLIYGHVKHKPTRDMVLNYSEFRNLFGAVETSGNKTNQDTIVHRFPLSVKITPSMAPVSELLLYYVRPDGEIVATTYSIKVGHCFENKVKSAWHTDAQTPGTVTQYHVEAAPWSLCGISAVDKSTRFLGSKANLIDADQTFEQLKRFYIEPELRPIWTQCKVTTQQETNTEEIDHLPIPLWGRRKKRTVISPGPYSGLTNYVDAIQAFDDFGAVVMSDLILETRPCPQLHRMDRFRSSTIQTFFLRSTSVLESEAGMFKMAAKTLPMAYPLLNEGPEQAYVDQVPDQPATLRSYFPETWLWELVPTGKEGKVAIERTLPHTITDWVGYTTCISSTHGLGIAPPTTITGFQPFFLDYSLPYSVKRGEMLHMKVSLFNYMQHSLPVKIKLEDATGLDLHLSHAVASFCVKPRDSVVHEYILRPRVLGDVNITVSASVDSEYGEPCGPEVLLYTRDVIVKSILVLPEGFPVEATKSAFICPKDFSDDSTITWHLDLPDDLVPESARGYVSMIGDILGPALENLDNLVRLPMGCGEQNMILFVPNIHVIGYLDTTGVENPELRAKAVRNMEKGYQRELIYRHPDGSYSAFGPNVTEDGSSIWLTAFVIKSFAQAKNIIHIDERDLKISVKWMLKKQLENGCFPMIGRVFHKDMKGGLQDDDSSSSALTAYILISLLESGVPLTATLINNALHCLEKGMENGGGTTYTAAISTYALSLLEHPKANNSMKLLMERATRNNDLLWWEDKPSLGLSIEMTAYAVLSLVKLGGEANMVEALKAVRWMSKQRNAEGGFTSTQDTVLGLEALTKYAAAMSNDNTDLSVLVTGNEVDQLYRMHNDNRMVLTQIRLPVIPTIIEIFAEGEGCVLVQSNIKYNVAHATGSEAFDLSVNAASSTWVDECSMQKITICTRYKMADGESNMAVLEVGMISGYIPDRTSLHSLLEDPATKVKRFEEDRDVVTIYFDKLINQKTCISFMVTRENVVDRLEPANVKLYDYYQQELTISSSYSFAPTCSS

>DpA2M-1

HSVFIQTDKAIYKPGHLVQFRVIVVNPQLKPSVVGSLDVYMTDGKGNRIKQWNRVFTKQGVFASELQLSDQPVLGDWNITAVVSGQSFSKHFQVAEYILPKFQVTIDLPTYLTFNESKMVATVKAKYTYGKPVKGNVTIAAYPQYRVSYIQPFFTEPVRKTVQIDGTVDVDFNLFKELKLVDDFERDIRFDVTVIEGLTERKQNMSSLLTLYKYKYKMELIKTSDSFKPGLKYTAFLKLAYQDNTPIQDANGVVIVKHGFSHNQDEYNRTEYPVPRNGILELNFYPPVDENVYTLGIETQYQDLVEWFSTINRAQSPSNSFIQVILKTENPKVNEEIAIEVNSTAPLDSYIYEVMGRGNLVVARTVQAGNQRSHTFRFQATAAMAPVARVVVYYVRADGEVVADALNFDVDGTFQNFVDIQVTPDSVEPGKAVDIVVKAKPNSYVGVLGVDQSVLLLKTGNDISRQDVLDEVKSYDSTRRPDFESWLPEVGGPKMCEELRLAAPTLSVDVSFSKKVEEPIALRQHFPETFLWLDITNLGTDGTARFVKEAPDTITSWVITAFSLDTFHGLGVIEQPAKMQVFRPFFIQLNLPYSVIRGEVVAIQAVVFNYMNKEITAELTFENIGDFQFIDNGLEDNEISSEAIFRKKSVRIPAQDGTPVSFLIRPTTLGNIDLRLTAKAATAGDAIVKKLLVKAEGETIYRNKAYLLDLRSIRNYNKNVSVTIPFNAVPGSAAVELSAIVDIMGPSINNLNTLLRMPFGCGEQNMLLFVPNIVVTEYLKNIGQLTDAISSKALGFMETGYQKELTYKRDDGSFSAFGKSDAAGSTWLTAFVARSFRQAQPYITIEDHVIEDSLKWLSANQAPNGSFPEVGKVSHTDMQGGSGKGVPLTAYVLLAFLENKAGLRYGPSMQKAAEFLVKELPSITDPYALSLVTYALHLAEVEERDAAFDMLQAKANTTDEEFRFWSKPKSEKDKSNPWSSLTTSVDVEMTAYALLTFLQRGLVIEALPIMKWMVANRNSNGGFSSTQDTVIGLYALAKLAEKITVPNTNINVKIKHDTGAETFSLNRENAMVLQKFKLPPKTSHVEISAVGSGFSIIQVSTSYNLNVTGEWPLFTLDPQLFKNANQNRMQLTICSSFVGEESNMAVMEISLPSGYVMDEDSLPSLRAIKDVKKVETKEGGTGISLYFDKMTRNTVCPTVQAYRVFKVAEQRKVPVVMYDYYDSSRRARVFYEPV

>DpA2M-2

SMTIFIQTEKPVYHQSQIVRFRTIPINTELRAFDDAVDVYMIDPRGFVVRRWLSRQSNFGAVSLDYALSDQPTFGEWTIRVTAQGQTEEEHFHVEEYYQTRFEVNVTMPAFYFDSDDFIHGTVMANYTSGAPVRGNLTLKATFKPIRATPLIPDRPGSIRDSVIERNLTFKEFRGFYTFKYPMAELQRMVPKLDGIELRITAIVGDRYREDYVEGFSTARIYNSSLKLNFLGGSPQVIKPAMPFTCFLAVSFHDGSILPAERFRYDRLEIRPTVQFRSSASRTLEVREAKALPEQPGIWEFTIDLRSELAQTTGSNVGTGDKRVKDLVNEISALKLIANYKDGYGERATAEMLAITHYSIGDKHIKVLTSTRSPKVGEYLILHVRTNFYAETFNYIVVSKGIVLVTGSQSSSPSVTTLSIPLSAEMAPVATVVIYHVAKYGEVVADSLTIPVNGISRNNFTLTLNPMKDKTGDTVEVVVLGDPGSYVGISAIDKGFFNMQAGNELSYAEVITKMTSFDESLNGTLRHYWTSREGDAETVVNYPASTFGIDANRTFEYASLVVFTDAFVVRRPDACNITLGFLACMSGSCYRSERRCDGQYDCDDRSDEAGCPSNIRRELAQYRLKRINRLLRMYENSWLWKDINIGPHGHSIFSVQIPEIPTHWVVSAFSVSPKNGFGLVRSSREFAGYRPFYMNVEMPTNCRQGEQVGIRITLFNYATIEADVVVTLADSPDYKFVHVEEFGEVKSYEARTSRGEKQHLTWIPAQGSQVVYIPIVPTKLGEIEVTIQAKSLIRKDQVVRRIRVEADGVPQFRHTSVMLDLSNRAWFLQYVYVNVTETPIIPYEKDRYYVFGSNRARVSVVGDVVGPAFPNMPVNATSLLTLPMDCAEQNMFSFAANLYTVKYMRLTTQRKREIDRQAFYHLNIGYQRQLSFQHRDGAFSYFRADWDYSSKSVWLTAFCARILAEANFNEWENYLYIDPGVIAKAVDWMIQFQSPEGAFYEVAPRFADRKMNSTTSWGFNDPIRYRNISLTAHVVIALTTIRDLPGELGPRVAVSRSRAISWLDRNLNLLEKFGDPYEVAIVAYAMMLAKSTSAEAAFGLLQQKAREDGGYKYWGREPVPLPAQRLENQRPFLLPRLPNAFDAANVETTAYALLTYVGRQELFVEPIVKWINTQRLTDGGWASTQDTIIATQALIEYTVRHRIREVTSLTLHVEATSNPQLQRTMYITENNLATPQFMDIPNAWGTVKIQARGAGYAIAQLSLQYNVDVNRFVTPPAVRAFSVVPRLSFSGRNNSHINYDICSSWTNQRESNQSGMAVLDVAVPTGYYMQQQVLDEYILSRKVRNLRRAKFLDHKVVFYFDALDGDDTCVKFTFERWHPVANMTRYLPVRVYDYYAPERFNETVVQTYEL

>DmA2M-1

KKPSVFVQTDKATYKPADLVQFRILFLDENTRPAKIEKPISVIIIDGAQNRIKQLSDVKLTKGVFSGELQLSEQPVLGTWKISVSVDGDNRETKSFEVDKYVLPKFEVIVDTPKAVVIADKVIKATIRAKYTYGKPVKGKATVSMERSYGYFGDLNANGNKQEKTIDVDGKGHVEFDIIHWAQRGQYLPPIKLFAVVTEELTGNKQNATATVVLHQQRYSIEPYERPEHFEANKSFIYQVVVKNVDGSPVTNSAKNVKIGFDKSYSYFHEPSPKTRINFEAPVNENGIATFNVRLPDSDSRYYRIFASFDGSENTIGSISKFEPTPMSREPLKIQVNTKKPRLGEQVSFDVVSIEDLPYFVYTIVARGNVILSDYVDVPDGQKTYTVKFTPTFSMVPKATIYVYYVVNNDLQFEEKTIDFEKEFSNSIDVSAPTNAKPSEEVKLRIKTDADSFVGLLGVDQSVLLLKSGNDLSQDDIFNSLNIYQTSTPWMNGYGRYPGQTSGLVTLTNANYPYNTEFPDYVEDDPEIYAFENNLDALPPMPAIANFPPDTGNTVQPVEIRKNFADVWIWQSIGRSVGEEEFTLTKKIPDTITSWVVTGFSLNPTSGIALTKNPSKIRVFQPFFVSTNLPYSVKRGEVIAIPVVIFNYLDKTLDADVVMDNSDQEYEFTEATNEVLEKAIDEVRRVKRVTIPANSGKSVSFMIRPKNVGFTTLKITATSALAGDAIHQKLKVEPEGVTLFENRAVFINLKDQPEMSQSLDADIPNEVVPQSEFIEFSVVGDLLGPTLQNLDNLVRMPYGCGEQNMVNFVPNILVLKYLEVTGRKLPSVESKARKFLEIGYQRELTYKHDDGSYSAFGKSDASGSTWLTAYVMRSFHQAGTYTDIDPKVITAGLDFLVSKQKESGEFPEVGKLFDNANQNPLALTSFVLLAFFENHELIPKYQSAIKKAVRYVAEEADKTDDQYSLAIAAVALQLAKHPQSEKVIAKLESVARKENDRMWWSKATESTGEDGRVFHWKPRSNDVEITSYVLLALLEKDPAEKALPIIKWLISQRNSNGGFSSTQDTVIGLQALTKFAYKTGSGSGTMDIEFSSAGESKNTIKVNPENSLVLQTHDLPKSTRKVDFTAKGTGSAMVQLSYRYNLAEKEKKPSFKVTPTVKDTPNQLLIVDVCAEYVPLEDADKDKDSNMAVMEIALPSGFVGDSTSLGKIQAVDRVKRVETKNSDSTVVVYFDSLTPGDVRCLPLEASKAHAVAKQKPASVSLYDYYDTERKATEYYQVKSSLCDICEGADCGEGCKKD

>DmA2M-2

NWLYIQSDKATYKPGDKIQFRVLFLDKNTRPAVIDKPIKIEIRDGDQNLIKSWKDIKPAKGVYSGELQLSDRPVLGNWTVTATVQDEGKVTNVLVVDKYVVPKFEVVVLTAKNVAASAGYIRATIKARYTFKKPVKGHVVATIEGSSTEQSLPIDGEVNVEFPISATAKRLLKITAIVTEELTDIKHNGTAYVTVHQHRHKLEDLFWPTHYRPGVSSEFKTVVRNLDGSPVMDSSKMVNFNVLCCQVSKNFSASLQNSIATEHIMLPETCQSCLVTSTFDTAENIERYIYKLNKPLMIAINTKKPQLRKLLKINIISDTYLPYFILTVVARGNIVLSLFQEMKEKKKSQEIEFEPTFALVPQATIFVHYIIDGVLMSDEKTVDIERDFENTIEILTTNEALPRDEVSLKVKTNPHSFVGLLGVDQSVLLLRSGNDLNRDLILNNLATYSTDLVILTNANINIYRSSGGCYTNPGYTNCTGSLIGRTMFKNEPTKNSGPVPIVGSTRAQASLPPVRKLFPETWLFSNITDVGANGEYIIKETVPDTLTSWVITGFSLSPQSGLAVTRNPSRIRVFQPFFITTNLPYSVKRGEVIAIPVIVFNYLGMDVKAKVLMDNSDGQYEFIETTNKNVSQYLRGVRRKKTLWIPANTGRGISFMIRPKKVGLTTLKITAISKYAGDRLHQILKVEADGVQKYVNKAVLINVQRLNRRSLAPPEKTIIIEKADNVIEGSETVEFEVCGTSQAPQLEHLDDLVHLPCGCGEQNMFNFVPSILALSYLKAKNRQDQEIENKAKRYVETGYQIELNYKRNDGSFSAWGQHDALGSTWLTAYVIRSFHQAAKYIDIDKNVLVAGLDFLVSRQSTDGKFKELGMVIHNSHGSPLALTSFVLLTFFENEEYMPKYKHVIDRAVEFVVTEVHQSNEPYDLAIAALALSLARNRNAYKVLDKLDKLATRRGDHKWWTGSDKCKSSEVETTSYVLLALLEHNISDEPKPIVDWLISKRNSNGGFVSSQDTVVGIMALTKYELQSHASTEAIDIEFWHLNEDKKHVRVTKENEFKVQTHQLPENTNEVKLLAKGQGRAQVQLTYRYNVATKEARPSFKLTTTVKKSHKGRLILGICGTYTPIAASERNKTTNMALMQVQLPSGYVCDIEPFADIEAISDVKRVETKNEDTEVHIYFEKLSPGDRKCLTLEAIYTHAVANLKPSWVRLYDYYATERSATEFYHVDTSLCDICHGNECGNMC

>DmA2M-3

FKPYIKIQTDKGKYKPGDTINYRVIFLDENLRPDTAKDEVVVWFEDSKRNRIKQEKHIKTTGGVYTGKFELSEFATLGSWSLHVQNGDQHHDGGIYFGGRKQFGGFGHRWHRSDELVNFEVEKYVLPKYSVKMDATQQVSVRDGEFNVVLKANYTYGKPVNGKVLVNVHLDSTSSWENVDGKTVQTDYPGHSVVGTADMVGGKAKLTMDLKDFASYLPHKTSSSYAQITATVEEDFTGVKLNETGGVQLYPYRYEMSCTDYSSCFSFKPDKEHELNFKITYVDGSLITDTKSVVKAKFTEGIRRNYAFYAFGTDHQEPELPTIEKKTFVFESHLNASGVAPFKVVLPDLPDIANFTRYYSIELEFVDEKRDLYTTYPYREPKQIENPSSEEEKEWFRAEVQRPKDVWNLKIGQEYQVILNSSRPLKYFVYNIVGRGNILETKRVDLAEPQTTVNVTIKPTFLTTPYGRVYFYYVDETGEFRYTEETFSVEVELQNQIEIKAPAEVKPGADVALEIKTSPKSFVGLLAVDQSVLLLGSNNDLNKESFNWRLNGYDTSTPWQGGYSYYPGERTGVVTMTNAYFFYNRTAPDYNIQGFGGSSFAMRKTTVAHDSHVFHSGAGGPTQAVGFSAESASASAAPVVRKNFAETWIFADIESTEEEVFKWVKTIPDTITNWVVTGFSLHPQKGLGVTNDQTNIKTFQPFFVSVRLPYSVKRGEVINVPALVFNYLPKTLDVELTLDNEDQEYDFVDASNEVIGDQKRTQNIRVGANEAAGASFLIRPKVIGNILLKFKAISPLAGDAIHKPLKVVPEGITQYQNRAFFINLKDTGEFKNTFELEVPEDVVPDSERVEFGLVGDLLGPVVKNLENLLRLPSGCGEQTMSKLVPNYLVRDYLKSIKKLTPALDTRIKRNLQDGYQHMLHYRHDDGSFSSFGPTKWRQEDPVRNGSTWLTAYVLRSFSKIKDIIDLDEQILAKGYEFLLTRQAENGSFTEHGEYFYSSQRSLLTLTANSLLALLEEEKPNQAAIDKAVAYLSANTAESIELLPKSIAIYALQKAKAPEAAKQVASLKSLAKHEDDRTWWTEDLDKLRASKNCGRWWCWIWSQDVEITSYALLSLLDSDQETADSVLNTVRWLIAQRNGFGGFASSQDTVVGLTALIKFAEKSGYEAAKWEVTVSNKGKREKTEKLNTSEENDLLLQTVEFPQGTKSLEFEAKGTGAAMVQISYQYNLVEKEPKPSFKIQTTVLPESSPANLELSVCVDYVEEGESKESNMAILEVSLPSGYTADEDSFADIRNIERVRLVETKNGDSVVVIYFENLAKNEEKCIRIEAYRTHAVANQKPSSVVLYDYYDTNKKATEYYSIKSKLCDICEGDDCKSKC

>DmA2M-4

QHTVLVQTDKSIYKPGDLVHYRVLILDANLKPARGYGRVHVDIKDSGDNIIRSYKDIRLTNSIYSNELRLSDSPRFGTWSIVVDVSDQEHTQTFEILDHILPKFVVDIDTPKHAIYKDGKIAATVRAHYAFGQPIVGEATLSIYPTFFGSLQPFVNDLITRKVVPIDGNAYFEFDIENELHLKQDYERQYLLDALVEEKSTGSVQNYSTVLTLHLNHYRVEAVKVPSYYIPGVPFEATARIARNDGGQLRDFNPQITAYLTNVYGSSEMYNRTAYSLDASGEIKMKFTVPIGDRDEFHSIIVDYQGVISEVGKIPSKHLHSKNYITAKVLNDRPTVNQEISVVVRSFAPIKYFMYQVVGRGDIILSRNVDVAPGTFHTIKFLASFAMMPRANLLVYTVIDGEFVYDEQVIQLEENLLNAVQVDAPIRAPPGQDIDIGISTKPYSYVGLMLVDQNANDLRSGHDLTHKRLMDALRSYELSDVNTPMGSPGKESGVITMSNTDYFIEKEAESNPALDREVSTGPEEDKLTTVRKTDIGPAHKIEVNTLPPGKGRYAFSYTPKPFWHNPRVHVMRDPADTWLFLNISASSDGRNSIHRRIPSEMTSWVVSAFALDPVNGLGLSPPNGKLEAYKEFYISTELPYSIKRDELIAIPFVVHNNRDSDLNVEVTFYNSALDFDFPQLDPKATNQPKVELYRRRSLQVPGRSARSVSFIVTPKRVGPLLVKAMAASSQAGDTVEQNLLVEHPGAMERINRGFLFELNSNAQNRRNVTIAVPRNAIPESTRIEVSAVGDLIGSLVGNLDSLILLPTGCGEQTMVNFVPNLIVLRYLGRLRQLTPEVELRATNNLAIGYQRILYYRHENGAFSAFGLDIKRSSTWLTAYVARSLRQAAPFTQVDSNVLQKALTYLGSVQSANGGFEERGDVFERFGDDGISLTAFVTLALMENVDLYPEYRNNINKALDFITRGLDGSSNLHAMAIGTYVLSRANHNAKAAFLQRLDSMATNKDGLKWWNKTAPAGEQQSPWYNATRSVNIEISAYAALALLENNLVGDALPVLNWLMDQRNPKGGFVASQDTVVGLQALLMFAERFSSQGNNLQIGFHYGEGAETIINVNAENSLALQTVELPNNLKNLSVSATGRGMALAQVSYTYNTNVTSAWPRFVLDPTVNRNSHADYLHLSACASFVSVVGENEQRSNMAVMEVHLPSGFVVDRDTLPTLESSERIKKVETQNRNTKVVIYFDYLDRREVCPTLHAYKTVKVTKHRPVAVVMYDYYDSARRARQFYRAPKSNICDICEHANCGDLCEKAEKRESKRPDDYTAIAGHSSGSRHTAIPLASVVMVLSMLLKTLSC

>IsA2M-1

SYSVFVQTDKAVYKPGQKVLFRVIVMDPYLLPTVTGAMNVHVTDAKGNRIHQWDRVLTQKGIYSSELQLSDQPVLGDWAIHVDILGQKYSKNFTVAEYVLPTFEVRVKLPAYATYNKSEVVATVSATYTYGKPVKGTVTLTVAPRTRYHQLRPRPYEQYQTKAEIDGSVDIPVAVVRDLSLKTDFFRRDIEFFALVEERLTGRKYNSTSYLTLHDKEVKVELVKTSETFKPGLKYTCFLKVAYQDDTPVHDAVNQLTLYQGFNFNEDLWKTSRHWVPANGVVRLELFPPNDNATVVLGLRAEFRGQTHYLEGIYPARSPTRSFLQAWVTTEDPMIGDLVEVEVNSTQPLDHLVYEVMGRGDIVFAQTLPASGVRTYRFSFSTSFRMAPRARVLVYYVRKDGELVADAVNFDLGGILRTPVQVQSNLAETKPGGQVDILVSTRPNAYVGLLGVDQSVLLLKKGNDLSQEQVIEELESFDSGKQARVWPPWYRRRRRSLWWPGSTTAHDLFKDSGMVVLTNGLVYESDDGLFARKQVIRLDTDVLTNPVLPPSDLPEAPPPVPGRIRLRQQYPETWLWSNVTASHDGRVVISSTVPDTITSWVISAFALDSLTGLGIAPSQAKVTVFRPFFVTASLPYSILRGESVAIQCVVFNYNNKPVQARVTLENAKSEFVFTSLSNDVGGEQSKDRRSKEVTVPAQDGVAVSFLITPTKLGYIDIHVSATSSLAGDSILKKLLVKPEGSKQHFNRAVLVDRRNPSAPPTSTNISIPIPKNAVPGSERISVSAVGDLLGPHVNNLDQLLVMPHGCGEQNMLDFVPNVVVLDYLRRANRLSPAVRGKALRNLEDGYQRQLTYKRDDNSFSAFGNTDRSGSTWLTAFVLKSFVQAVPYTSVDPAVLENATRWLVERQKPDGSFEEPGEVIYKPMQSGAGSGAALTAYVLIALLENKVGFQHALRFAASAAEEFLLKELRTQSDPYVVAVVTYALHLSGHRARDGAFQKLLSLATREDDMVFWKDPGVAPVNTTDKQSDFFFKAHFKDVEMTAYALLTLMERGDVSAAIPVMRWLVSKQNSNGGYSSTQDTVIGIQALARLAASVVSQTIAVDASVKYGDGRKRTLKIHSGNALVLQRIELPSDLKYVEIESSGFGVAIIQVSWSFNLAVSSEAPAFFLNPLLDKTSTESYLQLSVCTHYRGEGEASNMAVMEVGLPSGYLFDFDTLSSIHRTKEVRRVESQDSDTNVVIYFDRIGREELCVTVPAHREHKVANQKPVPVKVYDYYDLARSARMFYSPYKT

>NvA2M-1

SYSVFVQTDRSVYKPGSKIQFRCIVLDSRLRPTANRQLEIYITDGQGNRIKQWERPRLHQGIFNGELELSQSPVLGDWEIVAEIGGQTFKKAIQVAEYVLPKFEVTIDSPPHATFKEGKITVLVHAKYTYGKPVKGEATITAFPDIYSGVLQPIYSPPIRKTVNIDGKTTVDFDIANDLKYDDDYKRPVVIEVAVEEAVTGRRQNNSMQITLHKHKYTMELLRTAEYYKPGLKYTAFLKVTYHDGSPVVDNTNPVHISYGYTYNSEDLHNITRMLDKNGMVELDFYPPLSMPDKIFRPLRIEAQYLNLHEWFPSTNPATSRSESYIQAMLRTDKPKVNEYVEIEVNSTHPLKYLSYQILGRGDVLNAASIQISDRYTATFKFLATYVMAPIAHVVVYYVREDSELVADSLDVELEGTLQNFVDIKPVSDEVGPGDNIDLTITAKPNSYVGLLGVDQRSLLLKSGNDITYDQVRKELMSYDVNDAAFYDQEDYEHSWIRPGSANADEVFRKTGTVVLTNGYVHKNPQLTYPEEEGDRLKNPAQGISISTLRPDIGPPVKHRFATRPAWAGRYAFSYVPLLPWRPRVFLMHDISDTWLFSNMSSGYEGKTVIRRTVPDTITSWVLTGFSVDPAFGLGLIEAPRKLRVFKPFFLSMNLPYSVIRGEIVAIPIVVFNYMSKDLNVEVVLENNGDFEFAEVSNEVHDNTKRLELYRTKKIFVKANSAESVAFMIVPTKLNHITIKAKATSVMAGDSVEYPLLVKAEGETQYRNKVVFVDLRDTDSMKTNVTVDIPKHFVSDSEYVEVSAVGDILGPSIPNLSKLIKMPFGCGEQNMLNFVPNIVILDYLKNTNQLSPAIESKSIRYLETGYQQELTYRHTDGSFSAFGKTDPSGSTWLTAFVAKSFKQAEKYITVEEKIIADALKWLAEKQAPNGSFPEVGTVSHRDMQGGAAKGLALTAYVLSAFLEVENIEGRYRNVIYKGVDYVVRNMQGIDDNYALSICTYVLSLARNAYEDEAFRLLDSKATTKDEQKWWSKPIPEDDKKNPWFSLSRTVDVEMTSYALLAYLRRNQLSDAAAIMKWLVKQRNAEGGFASTQDTVVGLYALAKLGEKLRTNVYDVQVRITTDVGESKEININSRNFMIVQKHLLLSRTRAINITATGTGFALVQVASRYNLNVTGAFPLFTLDPQVDKISTNDHLQLSICSGFIPTKEANESNMAVMEVSFPSGFTVDQDALPSLELSQNVKRVETKNGDTMVVLYFDKMVHDKSYCPTVSAYRTHKVAKQKPVPVSIYDYYDSSRRARVFYEPKMTTLCD

>NvA2M-2

LVTFVETDKAIYKPGQDVNIRILTLRHDLKPWIKAIPKVWIENPSEVRVAQWTNVTTENGMAQLTFPLSTEPSSGIWRIKVEKNRPQLVHTSTFEVRKYVLPRFQVTIGAPSYILADAPNATWKVCVRYSYGEPVKGKLLLSLRPQTPIWKRKQTVADINYEETLDAKSDGCMNYTVSAQVLGLPHWKVAPNNVVLLANFTEAKSGVVETATSRSPVMHQPLKLEFSPHTLKYFKPGLPYHGKLRVLRADASSPAPNEKIQVCLRIRRKDEWQRSVVECRNFTSSNADGFLDFIVPPQNKNIVLLSFVATAVNYPTKYYSPDKRWRVFVDQPSAYIDVEPWYSPSSSYLSVTRGSQPLVCGEKYSFNVMYTSGTSSTVNATNLSPDDTEPISFHYSINSKGDLLVFGHVKYKPRKDTLLDYSEFRHVLGAGAGSKPNPSVHRFPLSVKITASMAPVSELLLYYVRPDGEVVTASHSIEVGHCFENKVKTAWQQEKQSPGSLAKFHVEATPLSLCGISAVDKSTRFLTQSQPVSSEGGSAASTGSNLLEPEATFARLKPFHLPPETMPMQSTWAHCDKSQGAEDSNSDEGPMEEIDHLPKPAARNKRHSVTYNVAANYVDAIQAFDDFGTIVMSDLILESRPCPPWRSSFGRVPLGPSSDEIDEPDRMLKAVKAMPLAFQFGAPGIPGPEASPMGQDVNYVNPTSMESQTSTIVRSYFPETWIWELVPTGKDGRATIERQLPDSITDWIGNTVCISSKSGLGIGNPVQITSFQPFFLDYSLPYSVKRGEQLRLKVSLFNYMQHSLPVLIKLLDHEGLDLGFNSSSEASYCLGPRDSIVHEFPLLPRELGEINVTVAAEVDKERAEACGAPTPLPDNRDEIIKPVLVKTEGFPVEISRSSFLCPRDFSDDTSLVWELELPRPEDEQVVEGSVSAYVSLIGDVLGPALENLEQLVRLPMGCGEQNMILFVPNIHAIAYLDAINRQTGSEMRARAIKNMQKGYQRELNYRHPDGSYSAFGAAADEAGSGSMWLTAFVVKSFAQARSIIQIDERDLKLSVKWIVRRQLENGCFPVVGQVFHKDMKGGLREEDGSSSALTAYVLIALLESGVPLSAALVNNALYCLEKASVSDHFADNPYTGALTTYALALLEHPRANESLRSLMGRASRQKDLLWWEDKSRPGSLALSIEMTSYGLLSLIKLGGENNTLEALRVVRWLSKKRNAEGGFSSTQDTVLGLEALTKYALKMANASATELSVLLTANDMEKLFKINDENRMLLNRVELPTLPTTLEIFAEGEGCLLVQSSLRYHKAKASGSEAFDLSTSTASVSTADQTPNDGCSIQRLTVCTRYKLPDEESNMAVLEIAMVSGFRPDRASLHDLLDEHATGVKRFEENDDTVAIYFDKLTAQKTCISFQAIRENVVDHAEPANIKLYDYYQQELTVSTSYAFMDVC

>PhA2M-1

SYSVFIQTDKAIYKPGHKVLFRAIILNAHMKPATHDFLDVFITDGKGHRVKQWNRTSTTKGVFSGELLLSEYPVLGDWKIVVNVVDQVFSKTFQVAEYVLPKFEVIIDSPKHATFKDSKVVSKIKAKYTYGKSVKGEATVTAYPLYYSGFIQPIFENPIRKVVPIDGKAIVEFDIVKELKITDDYERTIQVDVTIKLVYHDGKPVIDDVNPIIVKHGFSYDTEKYTAKEYKLPLNGLLELNFHINDPNVTTLGIEASYLDLTEWFSTVSKSMSPSETYLQTTLMTENPKTNQDIVIFVNSTSPLKYYNYLVLGRGDVLISNTVQVPEMSKTHKFLFVATPAMAPTAHVLVYFMTESGEVVADGLNVDFDNILQNFVKIDAKPLTSEPGKSVELSIQAKPNSYVGVLGIDQSVLLLKSGNDISHDDVLNELRSYDSGNYSPYSNFHNNFFRRFKRSFFYWPGSATAQKVFDNSGAVILTNGWVYDFVPLIHFRSKLPSVEVEEFPMMPEISMKTVDVSSSVINSGPRVRHRFPEAWLWDSIYSGAMPATAFGGGAIIPEAAFDAPLSSVGAPASEGAPVKIRKNFPETWLWESLDSGYDGRAILKPTVPDTITSWVLTGFSVDPVYGLGLIEAPTKVKVFRPFFVSLDLPYSIIRGEIVSIPVVVFNYFSEEVTAEVTLKNEGEFEFSDAANEVEDTPSTHRTKTVKIKPNSGEALSFMITPKTLGYISISVTATSKLAGDGVDRKLLVKPEGETQYRNKAIFVDLRSGGEFKTNVTLDFPKNIVPDSEFVEISAVGDILGPSIPNLQNLIRMPFGCGEQNMLNFVPNIVVMNYLQNTRQLTPAIQSKAMKFLETGYQQELTYRRDDGSFSAFGKSDPSGSTWLTAFVAKSFQQAGAYITVEERIIEEALDWLQKNQGSNGSFPEVGHVSHQDMQGGAAKGLALTAFTLITFLETQKVNPKFKNTIDKAVDYIVKNLDGLEDPYAIAISSYALHLAEHPSKDQAFHLLEEKAKTEDDMKFWKKPIPAGDEKNPWHERLPNGVSVEMTAYAMLTYLERNLIEDAFPIMKWLVSQRNDEGGFASTQDTVIGIFALAKLAEKITSPNFNVQAVFHYKAGSQAQTTINVNTQKAMILQKHELPKKVREINITATGSGFAIVQVSYRFNLNVTGAWPLFTLDPQVDKNSDNNHLQVSICSKFVGTKETNESNMAVMEVSLPSGFVVDSDSLPSLRVSQNVKRVETKDSDSVVILYFDKLIKQEYCPTISAYRTHMVANQKPVPVIVYDYYDSSRRARTFYQPLKATIC

>RpA2M-1

YSVFIQTDKAVYKPGHMVLFRVLVLSAHLKPVETRTIDVHITDGKNNRIKQWINAEPNRGVFSGELELSKNPILGDWKIVATVGGQIYAKVFSVAEYVLPKFEVTVNIPKHITFKDSKFGATVRAKYTYGRPVKGEATISVAPSYVSDILQPIFMQPVIKTIPIDGKAVVEFDIVKEIKLNQDFERLIVFDVTVKEELTGRKQNTTAEMWVHKHKYKMDLIKTSQYFKPGLKFTAYIKLAHYDGTPVSDQTNPLKVKSGYSYESSEYIESHYKIPPTGVVELTFYPPKNISVLGIEAEYLDIKEWFSTVNAAVSPSDTFIQAIIKTKKPKVNNDVVLVVNSTEPLRYVTYLVLGRGDVVTANSVRVPEGAKTVEWRFLATYNMAPIAHIIIQYVKADGEVIADSLDIQLEGTLQNYVRLDSTLEESEPGGNIQLNIETKPNSYVGVLGIDQSVQLLKTGNDIDEEEVVRELSSYETSDFGIFRPFKKVLDDLGQRRSVYWSPGSFTADEVFSGSGAVILTNGYVHKHTPWLYFRGGMPQDDLMFSANVSPEMAFDGGASSGLAAVKVRSDFPETWLWEALDTGVDGKARLNKQVPDSITSWIISAFSLDPVYGLGLMDVPKKVKVFRQFFISLDLPYSVIRGETMTIPVVVFNYMDKSVYADVTLENTGQFEFADYSNDVNEAPKLELYRRKKLTIQPNSGSSTSFMITPKELGFIDIKVVAKSTLAGDIVERKLLVKAEGKTVYKNAALFIDLRNSNHFKTNFTLDIPKYIVAGSEQIEIATVGDILGPSISNLAHLIKMPFGCGEQNMLNFVPNIVILDYLKNSYKLTKAVEERCLNYLEKGYQQELTYKHDNGSFSAFGNSDTSGSTWLTAFVAKSFYYAGRHSDIVDPQVISDALSWLASKQANNGSWSEVGQVSHKEMQGGAAEGLALTAYTLTAFLETRTLIGRYNNVINKAVDYLDRNVRLINDTYPLAVTAYALTLARHPTSSIAFDKLESLANTSSDMKWWTRSLSVSERKNPHMFSPNSIDVEMTAYAMLGYLERSLVNECLPIVRWLISQQNEDGGFASTQDTVVALGALAKFAAKIIVPNTDIAVSFTYGKDVTKEFKINSANSIILQKQEIPKQIREVNITARGSGFAVAMVAYSYNVNVTGAWPLFTLDPQVDKNSDHNHLQLSICSAYVGGNESNMAVMEVSLPSGYVVDQDSLPSLEISQDVKRVETKDRDTVVVLYFDKMTAKEYCPTISAFRTHKVAMQRPVPVTVYDYYDQSRQARVFYTPSVTS

>SmA2M-1

YMVFIQTDKAIYKPGQPVLIRVIVVSPSLRPTGTERLDIFVTDGDGNRVKQWNRVFTQRGVFTTEMPLSDEPVLGDWNITVNILVINKLKFYAKIEEIDQKYQKSFTVAEYVLPSFDVRIDLPNYATFTESDIVATITAKYTYGKPVKGKAVIMVTPLVRSPQIRTYYEDPLRKTVEIDGKVDVPFNLQQELNIKDDYHRMVRFEVIVTESVTERRENATGVIAMFKYKEKIELIKHSETFKPGLKFSAFVKVADNDDIPVNDSTNPLIIRYGYGHDDSLYQTKQFKIPTNGSIELEIYPPLADNIQRLIIIAKYKSIEQYFPPIRRAESPSNTYIQAVLMTEKAKVVFKWFAGGEEVLIHVNSTAPMPSFNVVAGLGALLKKIAKCCDSVALQIYQIVLGRGDIVFADKVEANGQKTIAVKFVATNNMSPRTRFIIYYTTSSGEVVADGLSFEVEGVFRNFVIKLFQKIVDLKANKHGSQPGDTINFQVTTQPNSFVGLLAVDQSVLLLKSGNDVTQEEIIHQLELFDTGKQPKSHLDLIYSSIWFPGSATASEVFKDAGVMVMSNALVYEEYNFIMPRGGGELRPGVEKGKPVGDYYIPKEAEVGFRDPLDGSIIPIMRQHFPETWIWSNATAGQDGRAVFTREAPDTITSWVISAFSLDMFTGLAVSPSPLRVTIFRPFFISLNLPYAVIRNEAIAIQAVIFNYMKETIEVCATVTLENTNQFDFVTVEDVVNEVEVVNSKTKKVESGTPATVYFMIVPKELGYIDVKVTARSSTRSQVVSDSLKRKLLVKPEGVPQFVNKAYLIDLRSSSLFNASVNVSIPKTAVSGSERVEISTIADIMGPTVDNFDNLLQLPFGCGEQNMIRFVPNIVVIDYLSSIQHLTPIVKSVALTNMETGYQRQLTYKREDGSFSAFGNSDQSGSTWLTAFVMRAFSQAKYFIAIDEQVVNGSLYWLIAQQLENGSFPEVGVVSNKAIQGGSGKGLALTAYVLLAFVENKAERIFSSQMTKALRLLEDQIESIEDSYSLAIVSYTLHVINSGKKDAAFRQLQSKSISAGEFRYWKKNASAETAETIKLATPIDIEMTAYALMSYVLRNDLSGSILIMKWLITQRNVNGGFQSTQDTVVGIQALTMLAKRIVDSQIYIDVMFQYDNEQKNVHLDKDNSMILMKEEIPSTVKMVNITATGRGFAIVQVSYSYNIMVSKENPSFQVNPFVDRSSTKDRLQLNVCAAYAENGATSNMAVMEVTLPSGFVIDRDSLPALHRVDEVKRVDIKDRDTTVVVYFDKLDNKLVCPTIKAYRTYRVAKQKATAVYVYDYYDQAKAARYFFQ

>SmA2M-2

FSAFIQTDKAVYKPGQLVHFRIITTDIILKPVDLKESSIYIVDSQGNRVKQWSNVTFEKGVFEESLQLYTYGKPVKGKVLLNVTDYYCKWPCSPYNMKPFSTTTSIDGTADIQVNLVRDLALPDWMRHDRRFDVFAVVTEELTGRKQNGSGDITMYDSQYKLSFSESGNFKPGLKYTITLQVSLQDGSPILDDGEISINYFSSWNSPGKTLNYTIPKNGEIDVEIIPPESTENLRFETSYKGARTTSYVSKAQSLSERYLQITLLSQKPKIGDEVEVLVDSTKELNDNLIVQVIGRGKILHHESIPPSKAKSQTLKFKITSEMAPKIRVIVYYATSCGEVVADALDFSVEGIFKTKVNIHANPNSTKPGSPVDISVQTEPQSYVALLAVDQSVLLLKSGNDITQREILDDLQSYEIGRQSYYSYRDGNYMIRPWRPPSPSTMQLFSNVGLVFFTNGLFAHIPSYGGYGGYGGYGGGGMYDGELYEMDAMPRPAVHFAPQAFASGNRGPPAAPPPRPPPPPQSRPISDTLMEPTRVRRHFPETFLWTNATAGADGMISITANAPDTITSFFITAFAMNENTGLGLSKSPEKLQVFRPFFVALNLPYSIVRGEAVALQALVFNYLKEDVDAEITLENGQNALDFVELENTVDEDKSQKKLIKTIRAKAGEGTSVSFLVAAKKLEYIDINVVAKSKVAADAIVRKLLVKAEGKKMYSNKAFLVDLRNQSSFEAKVSIDIPAGAVSESEKIEVSAISDVMGSTINNIDQLLRMPYGCGEQNMLNFVPNIVITEYLTKTKQLNDEIKNKALKFMESGYQRELTYKRTDNSFSAFGNSDKNGSVWLTAFVVKSFVQAKNYITIDDTVVSSSLTWLAQQQAKNGSFSEVGEVFHKAMQGGSGKGLSLTAYVLSAFLESKGIELLPGTSSVEEVVKSSLEYLEKELHNLKSDYDLVITTYVLHLADSKKKDEAFEKMNKVAKTEKDVKFWSVPLPVENSSVPYYNRPASVDVEMTAYAMLTYVQRGLIPEAIPIMRWLISKRNSNGGFESTQDTVMGIQALAKFAASLTPPAGSKLDISVSYDSNKTDFAITKETALILHREQLPRTTRDLTISASGNGVGVVQVSWSYNVLTTEDRPAFSIHVNATGENEELIINSCAKYIYKVNGESNMAVMEIEFPSGYVADLDHLPSINEEKKIKRVETKNGDTSIVVYFDKIGKEVCTSARGHRAFKVAKIKPALVSVYDYYDPIKRGEQFYNAP

>SmA2M-3

SGYAFLQTDKPLYTPKDIVKIRILNLNEKLIPNDKPVLLEIKNPNASRVLYEKFNPDDKGIIEYVFKFPIFPVHGLWSASIKYGYQLTGIKTVHFEVKEYELPTFSVKIEPPKVILKNQKLIETRITANYVYGKPVVGLLNIKFGIKTVEGIVVEIGSLYLQPLKEGERWISINVERDIKQKNIVWFPEVDGSNLLIEAEVIEKATGNRETAIDYSTIFSYSPFVVSFPRSSLDFKPGVPYQVQADVKFVNGKEAPKVSVSIISAVSKKGGQVFRHLPRPLASLTNELGRVMFELGTGIEDQELEITVTTEEEGLSVENQATNVLNVIKFKSPDTTSYVWIAAPPEEMIFTVGRTFQTQVTVFPAAGDIKLHYMVVSRGMLLLQGESASERNAVVHPIQFSVTADMSPSARVIVYIIKNNQIIADSMKIDVHQTCKYNNGDDLKLSATATGFYKPGQNVKIEITGERDSFVGLLAVDEAVYLLNKEGLLTRKTMFDEMERHDLGCGPGGGQDTEAVFRKTGFLILSNAAINEHKRIDGECDANHKRNKRSLSTLPKPVKVEHFYTGLAAVETEVTYKASYMAVARSAMSNKANDANLEDSELIDEFEVYVLQNLRHLFPETWIFHTEKITNKECSKNNKNSCKLTYSTNFPHSITTWRVQALAVSQTNGSLCIAEPIRLTVFKSVFIQMQLPYSAVRMEQILVPVTIFNYGDEELPVTVYMYGVEGICMGAGAGERSESQKITIPKNSATTVTFPIMPLEVASYPLRVVALSWSESDAVEKILRVVPEGIPVQKPLSIMLDPSGTIRQRKKRNAIDGIEETFDEEHNRQSLVIDMPLPKEYIPGTEKCLVSTMGDFLGQAVVTSIQGLEARFFHLPTGCGEQTMIKLAPLVYSVLYLKRTGRLTAEGEKNGYSLMQQGYSFMLNYRKADGSFAVYQHVASSTWLTALVAKVLCQASLFIDIPKEVTCNALDWTLRKQRDTGAFFADFGVYHTEMIGGVQSEATLTAYVLISIMECSKCDSADKRIAALRAISYLEHHVGFLNHPYSLAVVTYALTLAKSPKASSANAKLKALAIFNEAGDTRFWDVNHAEFQGNKPWIYVNRPNALAVETTSYALLTQLLFDDIAYSHPIINWLNQQRNDQGAFASTQDTIMALQAMAEYSYRAKLPALNMVCNVSSQTSRRQRSLIMTNENSLVLQKLELPAGGKIHVDVEGKGIATMSLSLHYNIESSKKDQCKFDLKIQTTEIEDIIMPQMPKPEFDGFDLLPEMVVRTITSSEDQKRKFGYKVVVQKEQEDDEYEYEYDNENDLAHESRHIVKLEICVRHLGDKPAGMSILDIGIFTGYKPVKDDLVKLLKDATISQYEPSDTSVVLYIDTVPHDEDLCIKLRTMQEISVGKVQPTTVKIYDYYEPDKSCQKFYTPDG

>TuA2M-1

DTGFGFIQTDKPIYTPKEKVRIRLMRLDDDLKPMADKVKLSIKNPHKIIMDEVVLESSKDNYFINHEFNIPPHFLQDPVKNKWTIIMAYGPEFQVTSNATFEVREYILPLFQVTLKSLQFITPSTSIVNGSVKATYLTGKPVTGTVRFKFKIRDSTNRSIGIGQTDELELVKGETSYKFSTNEFSESGVEVSMIIGSVLVVEATVIEAATRQKVKHIDSSTYFVSSPYKVSFESSFRVFRPEHPIRLIAEIYDVHNQPVVGIPVRLSVFSSRMVTKDVISDDLGRVTVDYDTTTQDKKIIFEVRTRDAKLKEDEQSFSRLLVEPSNQTSAALTLVEKNDRFKVGDKYTNSIIFEGSTFIFSQAYYITVVRGRIDKLERIPDNDNFGFTIEPHMAPSFRLIAISYRYDRVVSDSLLINVDPPECSLNLTYTNSKGATNDIEPGENGTLIVSANQSENRRQISIVGVDEAVYLLRSANTLTRHGLRKMFNSKDKGCGPGGGIDPTDVLLNSGLVIAGIPSNSIGSSCVHMNDKSSKRRPIRASPRIYITSSSSSMRKFASSPTYSSHSASPVSSSKQYSTNAFINQCCRLGKIKPNDKAKAMSCEERRDILLRSVKNVNCASAFLDCCLNSIIPPLLRTSVFDVSSGESKGQDNTEAGVTLGEEDAIEESTLIRQDFRETWLFDLVTLDESQTSVKYPVTVPHTITSWRLNAMSLSAKDGLCLMDKPLRLISNKELHIRVDLPYSIVVNEQVEMLVTIFNNGPTRKKVNLFMYGVDGVCSEADAGQKTERRLVTVEPGMLHTEGFALSPIRTGEFKIQVDALAHGTSDVVIKTLHVVPQGITIIDSYAVQLDPRNLQHRQKRSIKRENLFDSIDPDKGEQKTRIDLIPRTQTSAIVPDSEECVVSAIADSLGPSVITTLSNINHLIIKPTGCGEQNVIRMAPTLFTLDYLNATGRLTVTQRETGLKYLKAGYENQMMFRKLDGSFSTFEKRPSSLWLTAFVTRILCKAAPFLGDSLDPEVILTAVDYLVDHQESSPSGSWKEYHPVIHKSALGGLTGVIPITAFTYTTLRSCENFTYPRILAKRRDKSLKLAESYLCGKLTSELAKGDPYHLALLAYSLSTTTCLQSAERRKSIISRLREIGVYSSGENKLFWNTTTPIETTGYVLLALLNLRETKPDEIKAIINYLESQRSYTGAFDATQDTIVALEALSTYAKSAYNLTDINLICNISSGRFRKSIEFHEDNAQVMRTFEINNECDYVDMITRGTGLGSVRVKYKYNVLEAPEKLCGFELDVNVTQAIDSPEQIILSDSDIQLDDLFNITMLAEIGVLNDRLVQVESVVDSTLTDNNDSSSSKTGPSYTANEKAESNLKLSGASKVTNKLTVCAKRFDSVDSGMVILEVGILSGFVPDENDLVALKKNNPFISSYEKTARSVIFYLEDISSKQQYCLNFKIYQENKVANLQSAMVKIYDYYKKGQGCSQLYHSTRRT

>TuA2M-2

RLARFGVGPWGIGNYKLTTIGTGGLTFTNETKLVYDGKSFSVFIQTDKAIYKPGQKVLFRAIIVRPNLALADTGANYVYIEDAKSNRIKQWDRVFTTHGVLSFDFQLSENPVLGDWTINVEIDRKIFKKTFTVAEYVLPTFSVDVTLPNYVTYNKSDLIANVKATYTYGKPVTGEVTLTVQPRIRYGSLTTRPLEQYQVKAKLDGSVDIPVDVVRDVKLRKDFYDREIEFFALVEESLTGRKYNKTAILKVYDKDINIELIKTAPYYKPGLKVICYLKVAYRDDTPVEDNGPPVKIMYGYGYDEDAYNSIIEEVPNKGIIKFEFTAVNNSHSVIGMKAEYKGQVYNLNSFEMSLSPSSNYIQVAIPDGYQAKIDETLRLSVTTTEPIESFVAIVVSKGKIVSTETFYFKSGKQHVASLSITHSMAPKARIIVYYVRTANSEIVADSVAIDVGGIFRTNVEVSASTEKAKPGEKVDIKVKTNSNAYVAVLGLDESVLLLKSGNDITRDDVTNELATYDSNYYNPNSDDSFSYFNRNYFNSLSAAANIFHNAGLIILSNGIVFDHSIQLHYRSSFPVSLSESADSMSAFDEAPNPVGSRFYTKSLSSSTAPVKVRKYFPETWLWANQTSGEQILRYGFSIPGLAPSPAGAVSGKSIVLDDKLLDYRPRLAKIVLPDATESRIVLRKNFPETWLWDHTYSRSDGLATFSSNIPDTITSWSVTAFAVDKRTGLGVVEKPTKVTVFRPFFVKLNLPYSIIRGETVAIQVLVFNYFTKPREAQVVLHNDNKEFNFTVASNEVDTYLNDATRSQFVQVAAESAASVTFLISTNRVGSINLKVTALTDNAGDTVIKQLLVKPEGKTQYLNRAVLLDFSKAGGSSSNHLVPLWIPNNAVPGSKGLSVSVIGDVLGTSLNNLDDLLKIPYGCGEQNMLNLVPNIVVLQYLKTAGRLTLDIQSKALHHMELGYQRELTYKRSDGSFSAFGESDKNGSTWLTAFVLKSFYQAKEYITIDDKVLAEATDFLLKQQKSDGSFPEHGEIHHKPMQGGAAGSQGALSAYVTIALLQNNYFKQQYPKHFDRSEKYLYEQLREAKTSYETNIIAYALHLMKSATIEPALAKSLIKMKQDGDVSYWSDEDETEKTNFTNKQSSHFFLPKSTDIEATAYGLLSLVNHNQTDQAVTVMKWLISRQNAQGGFSSTQDTVIALQALSSIASHLTSSTQNIDVTFKFGNVTEKQTSRTFQIRDNNAQVLQKFEMTDARGETIPDHVEIVTQGTGVAVVQVSWRYNLAVSAEEPAFFLNPILGKASTDNFLQLNLCTYYKAGLATNMAVMEVELPSGYSADVDALQSIKSQGNGIKRIESHNGDTNVVIYFDRLTREELCLTVPAHRTQKVANNKPVPVTLYDYYNRHESARIMYEPKM

>TcA2M-1

PNKKITFIETDRMTYKSKDTVRLRVLTLGNNLLPILTHKIPFVRIRNPLGVGVIVWENVTTELGLAQLEYQLPQDPIEGKWKVEIGEDFRAFEVSKYVLPRFKVQILHPKIIYIGSRVAIKVCGRYSYGEMVKGSAFIRLSSIFPNFKTFQSLKKMDEGCAEFVLTPTDFSYFSIKKLFPLSDPKISVLITATVTEHGTDKIELDATKSVISLKPYGLKFAKKAMFMPGLPYQGFLQLNNVNMDLRGQVIEICYNIAIKKSWNYLNNEQCSNFTLQGNEKLIPFHILPLKNNVIHLQLNARSLNFTNIVDNFLVVRLFSPSLTYITIDQIHHSNNCKSIQQFAVQYTTEKLKEHENVTFFYMIKSRGQIFKLRKITHNVRKSSPNYSTEFKDILGASHKHTKASSIAKFTLKFKLDEKIFSNYQLLIYYVTPEGELASANKEVEVEPCLNNKVEANWSHKQIAPGATASLLIESKSESLCSVVTTDKAVTFMDDLRFLNVKSLLKPFLQQKEAPESGRKSCLPPVKKNRRRRFVYSFSEDFDAYDIFEKFGIVTITNFKVVTKPCYTGPIPPTEDPVSSLTDQYDTQNEDNITPIRSFFPETWLWEIVPVRSVAVIHRTLPHTITTWMTNVMCVSATEGVGFSKTGEITTFRPFFVDILTPYSIKRGETLYLHAIIFNYLTYNIPIRITLGTSEGLKLVDTKNRKSFSYCISSNNTATHIFELKGTDVGNVNITVVAELDPNFPGHCGPEIIINKRDVVFKTLIVEPEGHPITVTKSALLCATDNITWELPVPNDVVAKTANSKLILNGDILGQTIQNLDDLIAMPTGCGEQIMANLAPNIYILKYLNETKQLTSSVRHKIARNLKIGYQRILNYIHKDGSFSAFGYHDSSGSMFLTAFVVRTLQEMKKLVYVDQKIIERAVLWILSHQLENGCFSTMSHVFQDMGGTNSENSTAALTAYVIISLLDGNIDVPEAVKTNAKYCIRGYYDLDRYTLAISSYALLKINWFSEAERMLKKLFQVSSHKDNMMWWTNREINGSEASDIEVTSYVLLALIQQKNEENLAKAHSIVQWLSTKLGHRGSFKTTQDTVVALDALTKYSKFLSHKTDININVVALESAHNFVMTDKDRLKSKKIVLKNPANKVRVEVQGQGCVLIQAITSYNVKQLRNGDAFKLDMEVLPVSNIDKCSITTLSPCFKYNGPDHIANMAILEVGLPSGYQADRASLYKLIDESSVKMFEELEEKIVLYLTKLGNRQMCVNFNINENAIVKSRSNSTVKLYDYYKPEYQVSQFYKIKENC

>TcA2M-2

SYSVFIQTDRAVYKPGSKVLFRAVVLNSQLKPAAEVRNELLHIFVTDGQGNRIKEWKGIQALRGVFTGEVKLSESPVLGNWNISVKIHGQTFSKSIEVAEYILPKFIVNIQAPKHMTFKENVLVANIQTQYNYGKKVKGEATVTVYPTIFSGVIQPIFQNPIRKVIPIEGSATVTFDIAKELKLTDEYERVVMVDVTVEEASTGRRQNNSVEVHLHKYNYKMDLIKTADYFKPGLKYTAYVKVSSHEGTPLRSENREVTVRYGYSRADEVYVTEKHRLDKNGVAKLEYYTPVNVTNTTALRIEAQYQDLKERISPIPAAVSYSNTFLQVSLETERPIVNLDVEILVNCTERLRYISYVLMGRGDVLNANTFQVDNMHEYRFHFTATHAMVPVAHLIVSYVRDDGELVGDALDIEVDGLLQNYMEIQVNPVETEPELDIDIAIRAQPNSYVAIMAVDQNAVKMRPGFDLTHSEVAEELKKYDPAQQSPYSMIMHDSKYHFFWKPGAANPHSAIYNSGADLLTNSHVDRHQPTLEDIYLRPVFYGTSTVKPDRGFGLPLHTVTRPPLAGPYAFSRIPKPVWNKPKVYLTEEIADTWLFTNFSSGYEGKTSIRRKIPSSLNTWVVTGFSLDPIHGLGLTTTSKKVKVSKSFVVTLDLPFSVQRREILAVPVVVYNYMDKDVNAEVTLHNPEQKFEFAEVSNNVNSTRKVELYRRKKINIKRNSGTSVSFMIRPLKQDTIEIKVTANSPKNQDVAIKHLQVTTEGETEYYTKTVLIDLRNNPNYKKSINFTIPQNMVTGSEKIEVSAVGDLLGPTMVHLENLIRLPTGCGEQNLIHLMPNLIILQYLRYTRQVTPTIQNEALDLLEKGYQQQLSYKRKDGSFSAFGMRDEKSSVWVTAYVALTLRQAKGHIYVDEKIIEGCLEWLANIQGRNGSFVEVGSVIYKEIQSREGNSLALTAFTLLAFIENQKYASTYSNTINKGLDYIARYISEQESIHTIALCSYTLQLARHPSKQSAFNLLDLRSKSRGNLKWWSKDVPSNEIKNPWNKLPRSIDIETSAYGLLTFLEANYFEDAIPVLNWLLDQQNSLGGFTSSQDTFVGLWAIYKLVLKLATNVNMQVEFTYGKDQRHNFNVNKNNAMIVQKLQLPKDIREVNVTAQGKGLAVFRVSYEYNMNVTGPWPMFTLDPQVDKNSNKDHLQVSICTGFVSRNLSETPESNMAVMEVNLPSGFTADIDSLPSLEVSQNVQKVETSNGLTRVTLYFNNVSSVNEYCPTVSAFRTHKVANQKPVSVIIFDYYDSSRRARQFYRSRTSTLC

**M. I51 IC.**

>ApSCPYI-1

PSDKIQVSYPSGVIVDMGNELTPTQVKDEPSVTWPADPNALYTLCMTDPDAPSRKEHTYREWHHWLVGNIPGNDIAKGETLSEYVGSGPPPETGLHRYVFLAYKQPSKLNFDEPRLTNRSAEKREKFSIAKFALKYNLGNPVAGNFYQAQY

>ApSCPYI-2

KEIIQVNYSNGAKALLGNELTPTKVKDQPLVSWNADANSFYTLCLIDPDAPSRAEPTNREWHHWLVGNIPGGNVSLGETLSGYVGSGPPPKTGLHRYVFLVFKQPSKLSFDEPRISNKSAEHRDKFSINKFALKYNLGTPVAGNFYQAQY

>ApSCPYI-3

EVVQVNYMSGAKALLGNELTPTKVKDQPSVSWNADPNSFYTLCLTEPDAPSRAEPIQREWHHWLVGNIPGGNVSLGETLSGYIGSGPPPNIGLNRYVFLVYQQPSKLSFDEPRLSNRSVEHRNKFSVNEFALKYNLGTPVAGNFYLAQ

>ApSCPYI-4

QVYYPSGLKAELGYELTPTQVKDQPSVRWNAELYSFYTLCLTDPDAGQLKEFNHWLVGNIPGADVSVGETLTAYVGSATPPKTGLHRYVFLVYKQPSKLVFDEQHISNRTAENRFKFSIHNFSKKYKLGTPVAGNFYLAQY

>ApSCPYI-5

ISYDYNSKKVPVYRGNIIKPNEALYSPKVNFEAPEKTLWTLMLTNPDGHLHKENSEYIHWLVGNIPGGDVNRGETVFNYLQPFPAKGTGYQRMIFVLYKQSSEIDFSSIKSVSEKIDLANRTFSTFDFYCSHEDIMTPAGLAFYQ

>AgSCPYI-1

PPESLLHVTYPGGLRVNLGNILTPTEVKHVPEVAWPEAEPDAYYALVLTDPDAPSRTAPKFREWHHWLVVNIPGMDLAKGDTLSDYIGAAPPRKTGLHRYVFLLYRQNERIYYKESRLSNRSTQGRGKFSTHKFSEKYELGLPVAGNFFQAQF

>AgSCPYI-2

VAPEQTIKITYPQSDVEVSLGNQLTPTQVKARPKLCWEVEPSALYTLLMADPDAPSRSNPEMRSWKHWLVGNIPGADVDAGDVLADYVGSGPPQGTGLHRYVFLVYKQPSRIVFNETVLSSRNPNRGKWNPAEFVKEYELGVPVAGNFYQAQYDDY

>AgSCPYI-3

KEFAKITYPSGVTVSGGNELRPTQVKDQPRVEWTAKPDAYYTLFMVDPDAPNRQEPKFREIGHWLVGNIPGTKVEDGDHMYAFVGSGPPNGSGLHRYVFLVYEQPGGLIDFSKAPRVSNRSRNHRVNYRHREFVKQYGLGELVAGNFYQAQY

>AgSCPYI-4

AEVAKVTYPSGAVVSEGNVLTPTQVKDVPKVEWNADSGALYTLCMTDPDAPSRKEPTYREWHHWLVGNIPGADVAQGETLSAYVGSGPPQGTGLHRYVFLVYKQNGKLTFDEPRLTNTSADNRGGFAIRKFAEKYQLGNPVAGNFYQAEWDD

>AgSCPYI-5

PDCWARVSFKSGRQAEGGNRLTPTQIRNPPVVSWNANERALYTLILTDPDVPSRDDPRYREFIHWAVGNIPGNDIDRGETLVEYLGAVTPRGTGLHRFVLLVFEHLQKLDFSAEPRITAQCGTVRRYFSTRNFTRKYDLSGVYAGNFFQTQYD

>AgSCPYI-6

PDAFAKVVYRGKKLVDAGKELSPAEVREEPKVEWYADPTALYTLIMTDPDSPSRMEPWNREFAHWLVGNVPGRHVQNGDTLFEYIPVFPRSGVGFHRYIFLVFRQQSWNDYSQAPRASSKNRTPRIRFCTRDFARHYSLGSPVAGNFFIAQYD

>AgSCPYI-7

FVPRVALDIQYQAGELLHPVKYGNVLKPSETQAAPQVQFDGNFNFTGQPASEEQQSWWSLLLTNPDGHFEDSEKEYCHWFVGNIPNGDVTSGEELVPYLQPFPAKGTGYQRHIFVLYKQTSRLDFSQYRITDAFDLPARTFRTLDFYRQHQDSITPAGLAFFQS

>AmSCPYI-1

PENVLKVTYPNQISVDIGKVLTPTQVKDKPNVTWNGDANTYYTLCMTDPDAPSRKNPKFREWHHWLIGNIPGSEIAKGDVLSDYIGSGPPKDTGLHRYVFLLYKQPGKLTFDERRLTNRSGQNRGNFSIRKFATKYKLGDPIAANMYQAEFDD

>AmSCPYI-2

APTEKIEVKYGNKSVDLGNELTPTETQQIPEIHYKHEGGVLYTLVMTDPDVPTRKGYNREFRHWLVGNIPEENIAKGEILAEYVGPAPPKNSGKHRYVFLVYKQNQGSITFDERRLSNRDGPQRKRFNVKKFAEKYNLEGPLAGNFMRVEY

>AmSCPYI-3

YKIDDDTSVKVYTGNVIKPAEASEMPYVEYKVEDDTLWTLVMCTPDGNLENSNNEYCHWFLGNIPGNKLEMGEQIIDYMKPFPARGVGYYRYIFILYKQNQRLDYVEYKKDQPCLTLKERNWNTLEFYRKYQDYITPAGLAFFQSDWDPT

>BmSCPYI-1

ALLQVKYPSGVEVKEGNELTPTQVKDEPSVKWDAEPGQYYTLAMTDPDAPSRKEPTFREWHHWLVGNIQGNEVNSGETLSQYVGSGPPEKTGLHRYVFLLYKQPSKLTFDEPRLTNTSSDKRANFKIAEFAKKYNLGDPIAGNFYEAQ

>BmSCPYI-2

PTKNIELKYPSGAIASQGNELTPTQVKDQPSVTFEAEADAFYTLVFTDPDNYDGPELVYREWHHWLVGNIPGGDVSAGETLSGYIGSGPPQGTGIHRYVYILYKQPGKLDFDEKRLTNTSIDGRASFSTKKFAEKYNLGAPVAGNFYRA

>BmSCPYI-3

SELLNIQYSNGINVDLGKELTPTQVKDAPTVKWASKENEYYTLAMVDPDAPSRENPKFREWHHWLVGNISGGNIGKSEILSEYIGSGPPKGTGLHRYIFLIYKQPEKCDFSKVPKLPNNSGEKRGKFSISQFAQQYKLGIPVAGNFFVAQ

>BmSCPYI-4

AALLQVKYPSGVEVEEGNELTPTQVKDEPSVKWDAEPGQYYTLAMVDPDVPSRKLPINRELQHWLVGNIQGNEVCSGETLSQYLGAAPPIATGLHRYVFLLYKQPSKLTFDEPRLPNISTVKRINFKIAEFAMKYNLGVPIAGNFFVAQ

>BmSCPYI-5

NIDVLYNLKDGTFLPVCAGNVIKPTEALEAPIITYESDDNALWTLAFTSLDGHLYENEKEYVHWLVANIPGNAIEKGETLVDYLQPFPLKGTGYHRYVFVLYKQDKTIDYALPKVTSSSALQDRTFVTREWYKKHQDNITPIGLSFYQ

>BmSCPYI-6

TSVGGTIVNDHNCDVLLPAQVFLDEPLFQYFMADSKKFYTIILVDPDSPPQVDGEFYLHMLKSNIPGLALKTKESSKTIGIDYRGYKPPTPSRGIDTHRYITLLYEQADGNNFLPTVPSSRNRFSLAKWLLGKNLCGPVAGTQFRLQF

>CfSCPYI-1

VPANVLNVTYPNNLSIEIGKVLTPTQVKDQPTVQWDGETNAFYTLCMTDPDAPSRQNPKFREWHHWLVGNIPGSDVSKGDVLSEYIGSGPPQGTGLHRYVFLLYKQPGKLTFNEKRLTNRSGDNRGKFSIKNFAAKYKLGDPIAGNMYQAEFDDY

>CfSCPYI-2

APIEKIEVKYGGKVVDLGTELTPTETHEIPEIHYKHEGGVLYTLVMTDPDAPRRGGYNREFRHWLVGNIPEENIAKGEILAEYVGPAPPKNTGKHRYVFLIYKQNQGAITFDERRLSTWDGSQRKRFSIKKFAEKYNLEGPIAGNFMLAEYD

>CfSCPYI-3

VPLDISYELENDQLTRVYNGNVIKPSEASNVPNVKYNAKAGSLWTLIMTTPDGNLNTCNEYCHWFIGNIPGNRVEEGEELIDYLRPIAPYGIGYCRYIFVLYKQDCHIDFSGYKKTKPCLNLKERDWKTLEFYRKHQDQMTPAGLAFFQSDWDSS

>DpSCPYI-1

AATITIKYDSGVAVDGGNELTPTQVQNQPIHIEWPVEEGAHYTLCMTDPDAPSRNTPTFREWHHWLVVNIPGNDIKNGEVLSQYVGSGPPEGTGLHRYVFLAYKQPGPLTCDEPRLTNRSGKHRGKFSIRKFAEKYNLGQPIAGNVYQAKWD

>DpSCPYI-2

EAVHVSYDSGVHVDQGKELTPTQVKNEPTKVNWLAEEGSNYTLCMTDPDAPSRAEPSKREVLHWLVVNIPGNEINKGEVLAEYIGSGAPKGTGLHRYVFLVYKQPGVLSCDEPRISNRSREGRINFSIRKFAVKYNLGQPIAGNLFQAQY

>DpSCPYI-3

APPETLIVEYEGGLIVNGGNQLTPTQVQNKPVKIQWTFQDGDLFTLCLIDPDAPSRDLPLLREFQHWIVVNVPGNDFMKGEALAVYLGSQPPPLSGFHRYTFLVYKQPNYLTCDENRLLEQNIKGRGKFSIRKFAAKYNLGQPVAGNVFLSKA

>DpSCPYI-4

YIVNVDYADHACVHMGNQLVPRQTQLQPQQVNFPTNGSGGLFTLMAIDPDVPSRNNSIYSEFLQWLVVNIPDEDIERGDVLAEYLGPLPSHKGGQHRFIFLAHKQPDGSIINTRGLPHAEPCDWASRARFSARKFAQLHRLGQPTAINYFTTEF

>DmSCPYI-1

PNQLLKVTYSNNLVAKDGVELTPTQVKDQPVVEWDAQPGEFYTLIMTDPDAPSRAEPKFREFKHWILANIAGNDLASGEPIAEYIGSGPPQGTGLHRYVFLLYKQSGKLEFDEERVSKRSRKDRPKFSAAKFAINHELGNPIAGTFYQAQYD

>DmSCPYI-2

AGTIKVIYGDDLEVKQGNELTPTQVKDQPIVSWSGLEGKSNLLTLLMVDPDAPTRQDPKYREILHWSVVNIPGSNENPSGGHSLADYVGSGPPKDTGLHRYIFLLYRQENKIEETPTISNTTRTGRLNFNARDFAAKHGLGEPIAANYYQAQYDDY

>DmSCPYI-3

KATITYPSGVQVELGKELTPTQVKDQPTVVFDAEPNSLYTILLVDPDAPSREDPKFRELLHWLVINIPGNKVSEGQTIAEYIGAGPREGTGLHRYVFLVFKQNDKITTEKFVSKTSRTGRINVKARDYIQKYSFGGPVAGNFFQAQYDDY

>DmSCPYI-4

ATLLTVTYGGGQVVDVGGELTPTQVQSQPKVKWDADPNAFYTLLLTDPDAPSRKEPKFREWHHWLVVNIPGNQVENGVVLTEYVGAGPPQGTGLHRYVFLVFKQPQKLTCNEPKIPKTSGDKRANFSTSKFMSKYKLGDPIAGNFFQAQWD

>DmSCPYI-5

TAVVEYPGDIVVKPGQVLTPTQVKDEPCVKWEADANKLYTLCMTDPDAPSRKDPKFREWHHWLVGNIPGGDVAKGEVLSAYVGSGPPPDTGLHRYVFLIYEQRCKLTFDEKRLPNNSGDGRGGFKIAEFAKKYALGNPIAGNLYQAE

>DmSCPYI-6

GPQEFLNVTYHGHLAAHCGKVLEPMQVRDEPSVKWPSAPENYYALLMVDPDVPNAITPTHREFLHWMVLNIPGNLLALGDVRVGYMGATPLKGTGTHRFVFLLYKQRDYTKFDFPKLPKHSVKGRSGFETKRFAKKYRFGHPVAGNFFTSQWS

>DmSCPYI-7

ELLRIKYDNTIDIEEGKTYTPTELKFQPRLDWNADPESFYTVLMICPDAPNRENPMYRSWLHWLVVNVPGLDIMKGQPISEYFGPLPPKDSGIQRYLILVYQQSDKLDFDEKKMELSNADGHSNFDVMKFTQKYEMGSPVAGNIFQSRWD

>DmSCPYI-8

KVISVLYPCDIDIKPGIMVVINETLKQPIIRFKADPEHYHTLMMVDLDVPPDNNTEWLIWMVGNIPGCDVAMGQTLVAYDNRRTIHGSNIHRIVFLAFKQYLELDFDETFVPEGEEKGRGTFNCHNFARKYALGNPMAANFYLVEWL

>DmSCPYI-9

EHLFGSAYFVPRVPLNISYQLDGDSLAPVYNGNVIKPTEAAKAPQIDFDGLVDPITGQAAGQDTYWTLVASNPDAHYTNGTAECLHWFIANIPNGKVSEGQVLAEYLPPFPPRGVGYQRMVFVLYKQQARLDLGSYQLAAADYGNLEKRTFSTLDFYRQHQEQLTPAGLAFYQTNWDESLTQFYH

>IsSCPYI-1

VPQGVANVNYGDGTAVCMGNTISPQIASNKPTVSFEAQDALPPYTLVMVDPDAPSASEPIYRSYLHWVMVNAPSSDGFGEGEEAVQYIGPAPPQGSGPHRYVFLVVAQNGRNISKSDVSYSDRKSFNFEMFLQNNSLPQPLAANFFFSE

>IsSCPYI-2

EGVKVHYPSSNAVVKMGNVIRKEDAAQAPTIEFKERRNNLYTIMMLDPDAPSRRNPKHRSWVHWLIVNAEGPGTGRVDPDNVIQSYKGPGPPAGSGAHRYVFLIFCQGKRRINAKAVKQWVPQRPGFDLAKFRRRANLHLPFAGNYFFA

>IsSCPYI-3

PQGVVNVNYGNGTAVCMGNTISPQDTSNKPTVSFEAQDASPPYTLVMVDPDAPSASKPIYRSWLHWVVVNVPSSDRFGEGEEAVQYNGPAPPKGSGPHRYVFLVVAQDGKNISKSEVSYSDRRSFDFERFLKNNSLPQPLAANFFFS

>IsSCPYI-4

KQNVVVRYQNCDVSLGNTLRPEEAASSPDSVVFQTHSNSLYTLVMVDPDAPSRQNPKMRFWRHWLLVNVPSNCDLSGADCVTEYAGPSPPKGSGPHRYAFLVYTQGSTRISERDVHVPEARGKFNLAKFLSSLGLADALAANFFYS

>IsSCPYI-5

SLSCRRFLRPSCVRFQSCAAKSKMEAHQVVPDVIDTVPPGVVQVNPDAPSRQSPKYREWHHWLVVNIPGVNVPQGEVLSEYVGSGPPKGTGLHRYVFVVYKQPGRLTCDEKRLTNRSGDHRGEFKIREFAKKYQLGEPVAANFYQA

>IsSCPYI-6

EHDAEHVMPVYHGNHILPSEASQAPSVSFDSEPDALWSLVLTSLDGHLLDNDKEYLHWFVVKYIPGACNVANGEVVCDYMQPFLPRGTGYHRYVFVLYKQEGLIDYSKFKLPPKCTSLEQRTFKTHNFYEEHEKVLTPAGLAFFQ

>NvSCPYI-1

VKVSYPSGVSVDIGKELTPTQVKDQPSVEWDADSSSYYTLCMTDPDAPSRKDPKFREWHHWLVTNIPGKDVSKGDVLSDYIGSGPPPDTGLHRYVFLVYKQPSKITFDEKRLTNRSGDGRNNFSIKKFAQKYNLGNPIAGSMYQAAF

>NvSCPYI-2

ELLSVTYNDRPVEFSMELTPTQVKDAPAVTWSPEASTFYTLCMTDPDATSRKNPILREVLHWLVTNIPGNDVSQGENLAEYRGSGPPEGSGLHRYVFLLYKQPGKLSFDGEKRISNRSRDGRLKFSIRKFADKYGLGEPIAGNMYQAQ

>NvSCPYI-3

DLLSVSFEDKVLRFIGEELTPTQVKDVPSVAWKSEASGFYTICMTDPDAPSRSEPKFREFLHWLVVNVPGEDIAKGDTLAAYVGSGPPKDTGLHRYVLLAYKQPAGKIDVSEEKRIPNNSRDGRPKFSIQKFADKYKLGAPIAGNMYQAEY

>NvSCPYI-4

NELLTVTFKDSNDKDKDVQFGDELTPTLVKDPPAMSWFSEDSAYYTVAMVDPDAPSRDDPNLREMLHWLVCNIPGGDLSKGDVIVEYVGSAPGKDTDLHRYVLLAYKQPEKLTIEEAHISNHEHTGRPAFSIKNFADKYKMGDPLAGNMYRA

>NvSCPYI-5

YYKVSYNNKEFGFASELTPTEVKDAPTHIGWGLDSSSFYTLIMNDPDAPSRQDPKMREFLHWAVVNIPGDDFSKGETLAEYMGAGPPQGTGLHRYIITLYRQPSKLTFDEKPMNNLSIEGRVNFNLRKFIEKYKLDEHVAGNMFKAQY

>NvSCPYI-6

TKPFSIAYEGKSVQLGEEWTPTGTIPIPTVKWDFESSTFYTIIMIDIDPPSRAKANFREFVHWFVVNIPGNDISQGQTIAEYTPTAPPIDGGMHRVVFLVYKQPEKLTFDEPYAGNRSLDGRFYFSQRKFSAKYNMGAPIAGNVFFS

>NvSCPYI-7

NAPNETIEIKYGDKEVKLGNEFTPSETKEIPEVHYKHEGGVLYTLVMTDPDVPVRGYNREWQHWVVGNIPEDKVAKGEVLTEYVAPAPSKTTGLHRFVFLLYKQNQGSITFDERRIGNRDKRRNRFSTKKFAEKYNLEGPIAGNYMKAK

>NvSCPYI-8

FGSEDKLARVHRGNLIKSYEAKNAPSVTYKAEPDSLYTLLLTTPDGNFSDPSYEYCHWFIGNIPGNDVAKGEQLVDYLRPIPPKGIGFCRYIFVLYKQDKKIDFSEYKKEIPCLKLTDRDWKTYDFIRKHQDYMTPAGLAFFQCDY

>PhSCPYI-1

AEVAEIKYGNLALSLGNELTPTQVKNPPSVLKWKAEEDSFYTLCMTDPDAPSRKDPKFREWHHWLVVNIPGTDVNKGETLSEYVGSGPPKGTGLHRYVYLIYKQNGKIETSKLRKLTNKSGDHRGKFSIQKFSEEHNLGNPIAGNFYQAQWDD

>PhSCPYI-2

HLKVDKENCGSEYVKSEWQAEPKVNFVDAKHDKSYTVMCVDPDPPGYEKGQYWLHWLVSNVKGDDLAKGDLTKAKHSLPYYGPAPPEGSGLHRYIFLAFEQENDNVELDVPKVRSKFHLNEWLAKHTKLCGAHARTQVHTEKFFT

>PhSCPYI-3

LVAPVYWGNIVKPDEAFIKPQVKFKSDPNELWTLSLVCPDGHLTIQNGEYIHWLVGNIPGGEVGKGEEIWDYLPPFPPRGVGYLRYIFVLYKQEKKIDFSSLKNKLPRLELSDRNFNTLNFYKERQDELTPAGLSFFQSD

>RpSCPYI-1

PAQHLKVTFENNAVNDGNVLTPTQVKNQPSVEWDADPSALYTLCMTDPDAPSRKDPKFREWHHWLVVNIPGSDVSKGEVLSEFISSAPPKGTGLHRYVLLVYKQKEKLTPDEPRLKNNSGSNRAKFSVSKFAKKYNLGEPVFGNFYQAEWDD

>RpSCPYI-2

RAPAQKLKVTFGGKEVDDGNILTPTQVKNQPSVEWNADPNSYYTLCMTDPDAPSRENPKFREWHHWLVVNIPGNDITKGEVLSEFISSAPPKGAGLHRYVLLVYKQNDKLSCNEPRLKNNSGKNRGKFSISKFAKKYKIGDPMFGNFYQAEWD

>RpSCPYI-3

NIDYINGNSVVPAYWGNVLKPEEAQSVPNVTFDSKKDDLWTLVMTTPDDYFSPNMEYCHWFVGNIRDGDLKTGEVIFDYLQPLPAQGLGYLRYIFVLYKQEEILNYNGLKLKNKNMQEERIFSTYEFYKERQKSLTPAGLSFFTAD

>SmSCPYI-1

QGILEIKFGNHELKMGNILTPTQVQNKPTHIFYVAESGAFYTLYMTDLDVPSRKEPKCRECNHWLIINIPGNEVSKGDVLSDYIGSGPLQGTGLHRYVYLVYKQQNKITSDEPKLENNSLEKRSNFKIKNFAKKYNLGEPIAGNFYQ

>SmSCPYI-2

DVATPVFNGNHLKPSEMKSMPEIEFPSDPQSLWTLVATNLDCHFEQDSFEYLLWFVYVLQLYNLHVRGNIRGNDISTGETICDYLQPFPAKGSGYQRVVFVLYKQDEKINYNSLKRQAPCLNLKARTFLTNDFYK

>TuSCPYI-1

SGISICGTELDSLQTFDEPLVKFPAETNTNYTFMMLDIDAPSPSAPIMRSFIHWMVVNALGGQLEPQSTVHPYISPMPTPGLGAHRYVFMVFEQPKGFTIDPNATVLDRNKFNVAEWVKQNTLFGPVAGNYFLEGN

>TuSCPYI-2

LDLIEVTYPNAICRESVSCGNELKSFQTLEEPIIKYPSESNTLYTFMMLDPDALSPAAPTLRSYIHWMVINVERDDLKSGSTIHSYIAPTPTPLLGAHRYVFMVFEQPEKFAVGSDAIVLERNNFNVAEWAERNRVFGPIAGNYFLE

>TuSCPYI-3

LNLIEVKYSSASVSCGTELKSLQTAEKPMIKYPTKSNTLYTFMMLDLDEPSPSLPTFRSVIHWLVINVKRDDLQSGFNIYSYLIPVPTPNMGAHRYVFMVFEQPKEFAIGSNAMIAVGSKFNVSEWASQNKVFGPVAGNYFLGK

>TuSCPYI-4

HTVEVKYPSGAEVKFGNELTPTVVKDVPTHISWPSEEGALYTLCMTDPDAPSRQNPKYREWHHWLVVNIPDNDVGKGKTISEYVGSGPPKGTGLHRYVFLVYKQPGQLSPDEKFRSNRCGEGRECFKIREFAKKYKLGVPIAGNFYVAQWDDYVP

>TuSCPYI-5

SEHYLALVDIEYAGDLQVSCGNELTPNQTAKEPVVFNYHSGWYEHYSVVMIDPDAPKDKGTVLHWMVVNIPGSNMTQGEVACPYFGPKPPMDHGLHRYISLIYLQSDEGPVAVKDWSQNRYNFNLTSWVQDNNLHGPLFGNFFKAQN

>TuSCPYI-6

TKVKPDVVIDTSLLNGKTLSFEDFQKVLPNGINLLGDSNNFYTLALLNLDSQFGKSDPVCHWMVSNIHNQSDGSTVHKEVISFIPPYAFNGFGYHRYVFMLLQSKEPKELPEINDVELKKRVINLNSLLDKGLTPVGLSWFQSSWDE

>TcSCPYI-1

ETTAEVTYPSGVKVEMGNELTPTQVKDVPTVKWNADNNALYTLCMTDPDAPSRKEPKFREWHHWLVGNIPGGNVGQGETLSAYVGSGPPEGTGLHRYVFLIYKQSGKINFDEKRLPNTSGDNRGCFSIRKFAEKYKLGQPVAGNFYQAQWD

>TcSCPYI-2

VAEVHYPKGVKVQLGNTLTPTQVKDPPTVKWEAESDAFYTLCMTDPDAPSRKDPKFREWHHWLVVNIPGDSIEKGEVLSGYIGSGPPKGSGLHRYVFVNYKQKGKISCNEKRLPSNSGDGRGKFSIKKFAEKYQLGEPLAGNFFQAEWD

>TcSCPYI-3

PSKLLKVEYKKTNKEVHLGNELAPKDVRDAPSVTYSGDPHAFYTLVMTDPDAPSRKNPKAKEWNHWLVGNIPGSDLSKAQVLTEYVGAGPPKDTGLHRYVFLLYKQPGKITFQEEHKSNTNGNRAKFSTENFAKKYGLGNPVAGNFYQAK

>TcSCPYI-4

AHLFVTYPNGKKVHLGEELTPSEVKDEPQVKWDAASTKYYTLVMFDPDAPSRSDPSFADVKHWLVGNIQGGDVSTGDVIAEYFGSGPPKDTGLHRYIFLVYEQKERLTFDEPRSLKLSRAHRLKWSLKEFVKKYNLGAAVAGDYFKAKWE

>TcSCPYI-5

SAKITITYPGGRTVEFGKELKPEEVKDEPQVCWDAAPDKYYTLLMFDPDAPSRMEPKIADVKHWLVVNIQGCEVKTGEVIAEYMGSGAPQGTGLHRYIFLVFEQKGKMQFKEPKSGKLDKEHRISWSMRKFRRENELGEAYAGNYFVA

>TcSCPYI-6

SQITIIYPKKTVDLGQEFAPQDVREQPQVHWEADPEKYYTLVMTDPDAPSRRCPFVAEVIHWLVGNIKGCDMSTGEVIAEYRGAGPPRGTGLHRYLFMVFEHEQAVTFDEVRMPKEGSRRHRLRFSTENFRKKYNFERIFAWNF

>TcSCPYI-7

KDNVQHPVYYGNVIKPADASNKPEVHYESDDKTLWTLIMTNPDGHFTQQDKEYVHWFVGNIPGNKIEKGETIVDYLQPIPPKGTGYHRHIFILYKQEKKLDFSDFKKPGKCLNLEDRTFSTLDFYRERQDDLTPGGLAFFQADWDRS

**N. I63.**

>ApLec-1

IFFKANWYKAAQYCRYHGMHLASISNQEENDKLEKYIKDNGLGHEHFWTSGTDQAEEGSFFWLANGRPIGYTNWNAGEPNNFKYENGEEEHCLELWNRDGKGLKWNDSPCSFETFFVCEM

>ApLec-2a

IVNELLNWEDAEEYCSQNGGGGHLASIDSHRTQMLIDTILINSPSYSDNAPYWIGATDMNNEGFFKWTDSSPFTYSNWYQGHIHQPYSGPNSKQPNDDGLSRQDCVELRQVYRPQNRLLKYFNRNSSYTWNDRDCSVKNRFLCQTQ

>ApLec-2b

PEVSWHTARSICNGIKAELTSVHNAEEEQFVESFIRESTDSRSAIYWLGGAWNDKSWYWVDNSTETFSAWLATDAANALPYKNICLAISWLSSPPVNLPRGLYWTANDCDTVGGYICKKNQTKL

>ApLec-3

KTSKVDWLEARNICREYCMDLVSIETQEENNLIFRLIQQNDAPYIWTSGRLCDFKGCENRPDLEPKTVNGWFWSATRGKISATNQTSAGWTYIPWSKSGHKKTPQPDNAEFDINGTVESCLSVLNNVYGDGIAWHDIGCYHEKPFVCEDSD

>ApLec-4

EVDWLDARNICRRHCMDAVSLETPQENEFVKQRISRGNIRYIWTSGRKCNFNGCDRPDLVPANVNGWFWSGSGAKIGPTTQRNSGDWSHTGGFGQAQPDNREAPQGNDESCLAVLNNFYQDGVKWHDVACHHLKPFVCEDSDELL

>ApLec-5

ELNYFLAYEYCRSIGLQLASFETLEKTNSISDFLRNAGYNKFDYWTSGNRLGTDMLIWMSTGLPFNTTFNQMKRPNSPDNNGLDNEDLKMNARPELPIARKKRGESGSRDGCVAIKAPNMDWVTADCTDLKDFICEQTRC

>ApLec-6

DRVTWFEADAVCQFHHAQLATVESNSQFEAIRSYLKELDVIENVWIGLKRNSEASEFTWTNYQPLARSGYFREEVPRSSDPVCVVTDPTANFKWHSLHCGGPEVASFVCELPVPY

>DpLec-1

NEGNTFAKARDTCIKQRGDLWNNVARSTLELITRELERKKHAMKSPMVWVGAQKEASFTSRTWRWVTGNVVERPPWGREQPNNYNGEQNCVVLDGGRDWLWNDVGCNLDQIHWICKFPPLTCGHPD

>DpLec-2

NKSYSWYEANQFCKEGNMALLSLENENEDRLVYKHIKSILALNGELYWTSGQHSLRWEWADGESINYTNWKTGKSEPNEEIKNEAYLYLDTSYDFESGFWFASNNSRLLQSSDNSSFSRPMSYICES

>DpLec-3

KMIREDARQHCRDNNMHLLSIETQEENEFVINLVKKNDDLIPNQFWTLGYNANLDPNNWGWQQPNLNVSKPLTYKNWCRGSEPDNGFENEHFIAVINRCWHDVPGHFDWASVCEREANIFDPNLDEKFF

>DpLec-4

VGKNWISADTFCKDEGMGLLSLETVEEDKLIYDHIKITPELNSVSYWTSGMYSLDGDKIWEWASTEPFQPLTYVNWSPGQPDNNGPGYCLHLDLIKTFSAGYWADIFCTNSFRFICESIN

>DpLec-5

NRVTWLDADKFCRIGGMTLLSLESKEEDQMINNHIKSTSEFSDDYYWISGKFSNNRWEWANNEPLTYTHWYTGEPSYNQSGSFVYINYLFPNGIWFDQIGPAYILFICESIDE

>DpLec-6

RLEWVSADTFCRDENMRLLSIETYEEDQLIYNQVKSISQLHSTDYYWTSGKYSNNRWEWASTEPFQAMNYTNWFTGEPSHSQTGSFAYINFNFNNTGLWFDEIGPTPTTTDATTASTTSVTTKATTETTK

>DpLec-7

QLKRDWAGANSFCRGGSMALLKIESSQENDLIYNHYLATPGITQDIYWTSGRYSRDGNKEWEWATAPPYAKFNYTNWSPTSTSGPQPDFCLPNGSDCNPAYAEQFSVDIFFLFDGRWYDVVNTQAMNFICESIA

>DpLec-8

LPTWMRCDEYCKGGNMTLLSLETEAEDMLINSHVQANPELNFSEYWTSGRYSQEGNNRWEWASTQPFQPFNYTNWHPIYNQPDDNEPGSCALLYFLDYSGYWADNVCIYSTRFVCESIE

>DpLec-9

SQLDWTSADQICRDGSMTLLTIETLEEDQSIFDYVISSSKLVPNGLYWTAGKYSQDNDEWEWASNEPFEPFSYENWGVGEPSNATDEYCAYANFSPPQNFSAGYWYDDRCATPGFKFICEQND

>DpLec-10

ECWSNANYMCTQGGMSLVAIETGEEDSVISRHLMNIPELKGYAYWTAGRYNDTEWQWFSNKKPITFDDLKNTDKVKDKEAVSNRCILLNYVEEDYPSGGYASRICASFGYRFVCEATA

>DpLec-11

PTALDWAGADSFCRGGNMALIKIESSQENDLIYNHYLATPGITKDIYWTSGRYSRDGNKEWEWATTPPYAKFNYTNWSPNSTSGPQPDFCLNGSDCNPAYAEQFSVDISFFFDGRWYDVVNTQAMNFICESIA

>DpLec-12

FLMAWNDAKYYCRLNNYTLISLETKAEDDLIHQHIETTELLSELPWFWTSGTYSEDQWKWSTSPLPGEPFSYSNWASTRPIYNMNGYCTEINYSTFQGAWVDFPCDFTNYFICEGNS

>DpLec-13

AQQSWAQANDFCKANSMKLLSIETQNQQKDVSDILMPLIGIWTSGSYNSGQKSFTWLSTQKPFTYVNWFYGEPNTGLSNQCVRIRIVPESLTDGKWATLDCNQWLPSICEDN

>DpLec-14

NWTAAQEFCRKNDMFLLSLETQNETELINNHIKNSGLPKDFYWTSGSDEANEGQWIWTSTQENITVTNWRNNQPDGGKKENCLYLHSRDEFKWGDWMCNLSQYFICEW

>DpLec-15

GNWEYAYERCLEQNMTLSTIETQQEDKMIDDFFQLNHEFNDRILWTSGKYSELGSQWGWNHYGNETVAPGTPMGYTNWYPGRPKNNKDGRCLGLVFNDYGVTYENGHWDEFSCQNGYYGVFCEETQQ

>DpLec-16

GNWEYAYERCLEQNMTLSTIETQQEDKMIDDFFQLNHEFNDRILWTSGKYSELGSQWGWNHYGNETVAPGTPMGYTNWYPGRPKNNKDGRCLGLVFNDYGVTYENGHWDEFSCQNGYYGVFCEETQQ

>DpLec-17

KVDSWYIGDSYCRSNGATLLSLETQTEISLIDNYIASASSGLDKYAYWTSGQWGGTGYVWTGSNQPFTATNWYPGQPDDMATGFCVRLFYGTDYAGRWSDIICSGEGYTPFYFICEL

>DpLec-18

TQKPWVDAFDFCKANGRSLISFKTAEKQAEFETYLPAIIAKSDNLFTTIGFWTSGTFCLETLAANVYCPSKNTWAWAATRENFGYVNWETGQPDVTMLPRTACARAVPTNKYKWDDIDCGQFLPFICE

>DpLec-19

FKANWYKSAKFCNYHGMQLASIESQIENDQLEKHIKEFGFGNEHFWTAGTDQGEEGSFFWMSTGRPVTFTNWNAGEPNNFRYENGEEEHCLELWNRDGKGLKWNDSPCSFETYFVCEAPL

>DpLec-20

VKTQLSWAKANDYCNTYDMKLLSIKTQSVQNDVSDILMPLVLGYPISKQIGVWTSGAYNTGQKGFAWLEPFTFVNWFYGEPNTGISNPCVRIVPESMSDAKWATLPCDQWLPFICEKN

>DpLec-21

TMFCVDHNMKLVSFENRTEEELVQAAWGTEVSYWTSLTDTRRDGTWLWESSMTVPADYTNWYPKRPNTAVNNVDDCMAYGGATYLNFWGDIACTTMAHAICEAQP

>DpLec-22

KKATWYKAEAFCRQFRMTLADVPTDKVEWIKHKVNNPSAVDGKGEWYWTSATDRFARGQMIWMNTGQLYQNPNSNSDATSLCVIVGPITPVSAESTSFLQEIESLEDEYFALIETISKEDDINDILIHGTCPKHFT

>DpLec-23

STQKPWIDAYNYCAANGRKLISIPNVARQLEISTYLPSVIGKDKVDIFSYIGFWTSGIYALTAYTWASIPQYFTYTNWIVGNPDITNLPRATCIRAAPAENYQWDDIDCGQFLPFICE

>DpLec-24

VPQQLPWLKAYEFCNSYRKNLISIQSSTDQQVVTQFLMPLILNDPVAKNIGVWTSGASVPGLKTYFWVSKLSLFFYSNWFYGEPRPTSLSECVRIAPVPEGPWASTPCEQWLPFVCQD

>DpLec-25

RNQADQTMYCVDNKMKLISLENATEEERVQAAWGTLSPFWTSLTDTRREGTWLWESSMTVPEYTNWYPGRPNSVASNTDDCMAYGGATYLTFWGDVNCATTMAHAVCEAQP

>DpLec-26

ANKNHADHSMLCIDNNMTLVSFENRTEEEFVQRTWSTQIPFWTSLTDTRRDGTWVWEGTMTILDTGDYTHWFPGRPSSASDNEEDCMLYGGNTFLAYWGDVNCTTTLANAICEAHP

>DpLec-27

VFNTKSFPDMTMYCLDYKMKLIIFENRTEEEIVQKTWGTQNPYWTSLTDSRRDGTWVWESTMTVLKAGNYTNWFPGRPSYVANNADDCMLYGGTIYLNFWGDISCATLARGICEVQP

>DpLec-28

HTTYCSDNNMKLVSIEDNNEENTIYLAWVVNFWTSGTYNKTTGIWTWSSTMANVSPGYTNWAPFEPNNTLVDGNCLTIKNGWYDDPCANLYDAVCESHPED

>DpLec-29

NVRRNWQDANASCKEIQAKLAEPKSTDELRKLTDYLRYNRTDQIGGGYWTGGLNPGLMWLWPSLGTSLTNIDPSMWFNTPDISTENNNGKCLRLSYDRNLQRYALQGSDCQRYLYFVCEYDVNSTAP

>DmLec-1

FFKANWFKATQYCRYHGMHLASISSQEENDRLEKHIRDFGLGHEHFWISGTDLADEGNFFWMATGRPITFTNWNAGEPNNFRYENGEEENCLELWNRDGKGLKWNDSPCSFETYFVCEVQPN

>DmLec-2

PMNKVNWFQAAGACRMMNAHLASIEDKPEMEALIKYMKAKGFKNNDYFWISGNDLGTEGAFYWMSNGRPMTYAPWNGPKQMPDNYGGNENCVHMFATREMINDANCKIQMLYVCEATEPKTF

>DmLec-3

AKVNWFQAQATCAAYGYTLVSITSEQDQRSLRNFLFNYARNQQDLLTDPLWTSGTDLASDNNWVWFSKGRAVNYRNFQNGLPGYSSDNRHCLGINGINGLWVNENCSELRYFVCEKRCQ

>DmLec-4

LQKNWFGAYEICRQQQAELISLETFDELRLVSEYLLANNIFERYWTSGTDLGTKGKHVWFSNGQPLSTDLWYGGEPNNKNNEEHCDELGSDFRPTKSPGMNDRNCNFESSFICEEVQPK

>DmLec-5

KLDRNWYDAFEACRQMNADLVAFEDRKEQKLIYHYLVDNEMDTTYWTAGTDLAEQDSFVWFSNGQPVASDLWCNNEPNNAKNEEHCVEYKPLHPEAKMGLNDRVCTFKTGYICRAPQP

>DmLec-6

KDAYDWQSAVDFCRDMGGYIAAIKDQEELDAISARLDDKSYWLGINDLQSSNTYVSVASGREVEFLNWNAGEPNHGNEDENCVELIRSKMNDDPCHRKKHVICQTDKEV

>DmLec-7

PELNYFLAYQYCRSLGLQLASFETKEKAESMTTYLKNAGYGNYDFWTSGNRLGTGMFLWMSTGLPFNATFDFFENSADAIQAGLLDPVDHNSNTSPQRTARDSSGAEKGCVILKQPTLKWMPEDCSAVKDFICEQTRCYY

>DmLec-8

VSEKNWSTASKTCRNMGGHLADIKDEADLAAIKANLKEDTHYWLGINDLDHEGKFLSMPTGKQTTFLKWASGRPSQLDTLNCVFLYNGEMYDYPCHYTFRFICQTEE

>DmLec-9

AKTNWFEASNHCRQNGGFLLNLESREELELLSPHLHPAYSYWLSINDLGERGVYVSEATGLEAPFLNWSAGEPDNSSGYDRCVELWLSTTSFQMNDLPCYSSVAFICQLN

>DmLec-10

EVNWLEANHVCNRVGAVLATVRNEEQHQLMLHYVNRKERIFGNRTFWLGATNLVDRSYFWTWMSTGIPVTYAQWSRREPKSDRTGQDACLVLGTDNLWHSEPCQRKHNFICENVCQ

>DmLec-11

KHKVDWFKATSMCHKMGAHLLTIQSEDELDAIRTELKDINDGSHDFWLDINDIAKWGEFISLATGMNPPFLKWHKHRPQVQIHQRCVHLRGGEMMDGKCSEQFLFICQLAVN

>DmLec-12

DETRRNWTSAGSACRQMGTQLATIRSAEELAALRAKLNKERHYWLDITDLEKEGDFRISASGKRPNFLKWRAGQPNNFSGNQHCVDLLDGLMYDNKCESLSYFICQSDD

>DmLec-13

EEKLNWHDALDKCHKMGGHLASLQSQEELDRFNNQLNGLNRYWIDVTNQFNESEFVSVTKGSKANFLSWADGEPTKDGECVDIRTFNGKTTMNDNSCFANLYFICEKSIE

>DmLec-14

QTKVNWYVAYENCRRLQSELVTFETAEEFDAIAAFLNARGDRSEHWTSGNDLGKTGTHYWFSNAQLVTIKRWAPKQPDNAGGREHCIHLGYIYGYSTEFQLNDRPCHNHASSLFKYICEAPKQ

>DmLec-15

GTESLNWYEAYEKCRELNSELVTFETDQEFDAVTAFLTANGSRLTYWTSGNDLAKTGSHRWFTNAQRISSLRWARNQPDNAGQKEHCIHLGYIYKDSRKFELNDRPCSQDPNSLFKYICEAPE

>DmLec-16

IKKINWFGAQNNCLRKGLNLADVSTMEDFKAVVHYVTSQVGFDDFWFGGNDLQSEGRFKYISSGKLVRYMGDSNIVEPTQRSNLDDCLEIRIRPNVTVVLDVNCQEKKYFICEQNQM

>DmLec-17

VRQNWFDAADKCRRMGGHLATPQDEDELYLIRKQLEARWFWLDISNLVDKDQYISLATGKEVSYLKWRHGEPKKSSTANCAYLYAGDYYTYQCSDRNFFICQAV

>DmLec-18

VKQNWFDAMTKCREMGGHLASPQNEEELHLISQKLDTESYWLDLSDLTDHGQYISLVSGSKAPFLKWNKGQPNRENAQCVRVKGGLYQTFQCDHRVLFICQANQ

>DmLec-19

KKMNWFGALNNCLRKGLTLADLSNQRDFDGAIGFLSGLGNTEDFWFGGNDLYHEGRFQYISNGRLVRYYSNYSNVLPLEHSECDDCLEVRIRSEINMVSADNCHERQYFICSERYC

>DmLec-20

ESLNWYEAYEKCRELNSELVTFETDQEFDAVTVSNGSRLTYWTSGNDLAKTGSHRWFTNGQRISSLRWARNQPDNAGQKEHCIHLGYIYKDSRKFELNDRPCSQDPNSLFKYICEAPEME

>DmLec-21

VELNWLDAQAKCRRMGGHLASIKTKQEFDAIVEKLDDSKSYFLGVNENTKTGEFVSAASGKSGLYHEWGPGEPHHNNDQERCVSILRKLMYVGNCTYEKSFICQYGI

>DmLec-22

GSWKNFYESDRHCRSLNAGLLSISNPTEFNVINEWLPIIAPYQPEFWTSGNKLGGTSDYYWQSTGQKAVYLPWSAGQPTTTAGDCLTLMANVTMTPEEAILSVHRLTVKPCTQWAPHICQAPLE

>DmLec-23

SLEVDWLDARNICRRHCMDAVSLETPQENDFVKQRIARGNVRYIWTSGRKCNFAGCDRPDLQPPNENGWFWSGSGAKIGPTSQRNTGDWSSTGGYQQPQPDNREAAQGNDESCLSILNNFYNDGIKWHDVACHHIKPFVCEDSD

>DmLec-24

HNLQVNWRTAEQRCIEMGGHLAAFQNAEEYNAIVGQLNKANYWLGVNDLAKQGEFISLASGKRATYFKWRKNEPKYNNPTQHCAYVFGHENIMIVLSCTTDVMHFICQSD

>DmLec-25

DIVQQNWTSALSACQKMGGNLASIINEADFNAIVSQLSKDNTYMIGISDLAEKGVFISVSSGKRAPFLKWNPGEPLYEHVDQRCVSIHNGGMWVASCTSDFKYICEAN

>DmLec-26

QEANWHVADRSCRKLGAELMVLDNQEDKLLTTTFLKSMGLSFTQSWHHSVWAGINCLGNRRTFLLARNGETVPYLNWVPLEPKLCRT

>DmLec-27

ENKQNWFGASNTCRQLGGHIATIRDEQEFNEIFSRAPAGVFWIDMNAMFKNGLFASSLTGRSPPFFKWKKEERGNKFDCVNVYNKEMYNENCFNTHLFICQAEQWD

>DmLec-28

NFSDRHCQSLNAGLLSFSNKMEFTAINEWLTTVVPQSPELWTSGNKLGGSEDYYWQSTGKKAFYLPWQAGQPTPITGDCLTLLANVTMTAEGTTMSEHRLSVRGCTKWAPHVCQAP

>DmLec-29

QKKMPWDSAYDTCRQMGGHLANILDEKELNEIFSEETKKKYWVDINSRANDGASWISTLSGRDVPFLKWKPDLATNIHRHCVYINSNEMYFENCANDNYFACQAEQWA

>DmLec-30

MQTWFEAYVTCRKMNGHLANIQDEKELDGILALAPNNSYWIDISKLVENGGTFVSTLTGREPFFVKWKSNQDTKKKNQCVYIYAKEMSYDECFEKKSFVCQADQWA

>DmLec-31

NIKHSWDKSAELCRRYGAELVAIDSYAENNETLAIARASDPNQRASDKYWLGLASLDDLRTNTLESASGALISQYSGYWSLHQPNAESGECVAAAFAGKSQSWDLGTCESLLPFMCRAQA

>DmLec-32

RQPLNFLDALSFCRSRGGTLISESNPALQGFISWELWRRHRSDVSSQYWMGAVRDGSDRSSWKWVNGDELTVSFWSHPGGDEDCARFDGSKGWLWSDTNCNTLLNFICQHQPKT

>SmLec-1a

NLVTWLDAYTHCSRLNSQLLSIETIEESFAINLHMSRKHGRDVTYYWTSGSDAYQQGRYIWMSNGKPLSYTNWCANEPNDENGFERCIHLNYLPNGMFAICWLWNVTNACP

>SmLec-1b

AVIWVDAFKHCATLDSQLVSIETVEESFAINLHLYYKHGQTQVSYWTSGSDAYQEDDYIWMSKGKPFTYSNWCANEPNNDGNEDCIHLNYYPSGTTCWNDAKCWSYNRLFICEKEQ

>SmLec-2

NQPANFMGASRTCESRGGSLVDETSPTLQGFLSWELWQRHRNEPSGQYWMGATRDSNNLNNWKWISGNDVSVSFWNLPGGNENCSRFDGTKGWLWSDTNCNLNLNFICQHRPLT

>SmLec-3

ENPVTWIDALTHCGKLNSQLLSIETVEESFAINLHIYRKHGHNKVAYWTSGSDSYQQHRYIWMNIGIPFSYTNWCHPGPDNYDGNEDCMHLIHGSNGNTCWNDAKCWATHVFDDNLTK

>SmLec-4a

MTWEGARNQCLSLGGDLVTFKSRQDETRVLNLVTNSNDDSSGDDFWLGLRLLNDSGINEWRWNDQSRLSYTNWNILLVHENVTANVEMFCGIFEIITEQWTIEPCSLTAISVCEADVG

>SmLec-4b

EYANWTTASFSCRSKGDDYDLVSIHSPLEHEFLLNELKSLGMERAWIGLNDKAIEGQFVWSDGSPFDYNDWAWHEKLIGKPNKFSTTTKGFGKITPDCVKIEMTSIAGKWTSYDCNVELGYICQKTFR

>SmLec-5a

YLTSWNNAQDFCNYMESDLVIISNEKIQKDINMYRYRQLNSMNVKDFWIGLNDFKSPGDFEWVDETKPMYSNWLNDTVNASGNCVMLDVETQYWKIQNCTSEFGFICSKDWSFNQ

>SmLec-5b

GENNWQTAENWCETENGNLTTISSSLENIFVYSFVRQKVGSYWIGLYVDDSGLKWEAENQDTFFNWDGQPEISSGLCAVMERNGKWLMQLCDGNQIKTAICEKPVS

>SmLec-6

NGTFLQGIAYCRNLMSDLVSIETKIESHCINYHLTGTNDLGLYWTSGVADFNRGNTRSFHWLSTGEAFTFTNWCPGQPDRWKENDFCILLITKCWNDVPCQGNYQFLCEYNLLFAN

>SmLec-7

IEASRNCTAMHSFLLSIQTEREGVIVFNQAVKENVKKEPFGYWTSGTRVTSKRGFEFVWSSTWEPLTYTAWCPSEPNNWMNDEYCIHLTNLQSHNTKACWNDRGCMESTTHSRHPMHYICETEA

>SmLec-8

DNKKKSWYDVRDKCRSMGKSLLSLDSNDKNDFFNRIFHTRNMSNCLGRIRNLRSNNTWQHRYIYWRWAATERDISYKYWCPHSEVVKEAMCGYIALDPMCWMAERCTNKDVNSFVCEAV

>SmLec-9

PEVTWYTARKICREMQGQLSSVGSQAEQNFIIDRIKKMETYTSGTLFWLGGFRKLNHDRGWQWVDSSPFNYSAYKLEARLLFDWYPDWMKEKNEPIPGLKHQNKFAQESCLAFQWKSVKDSRGRLKYSGLYWESQTCEQSGGYICKKNQIAL

>SmLec-10

LALSWTAADAKCKELTKGGLVADVDITVQRVLSNILNDASIKQPIQSHFWIGGKTTVTKGNKNVHWIWKNGAELEKFYGKLQPDMSATELACLSLSRELDWQWDDKTCESSFVFVCQHNIL

>SmLec-11a

SGNFDDSELACEMGNGRLAMVKSPEIHLFLHKLRIRRFLLNYWIGLNDKVAEGVWVYSDGSRVQGFKRWAPGEPNNAKGQHCGKIIMSKNLLWDDDQCNNQWLYICQFQG

>SmLec-11b

IATYDQAAQQCEFKGGYLANIKTPSTYEFIKGLVYNNRKWLEMDEKKMADILRVNNNRIPAIWIGVRYYKGKWVFSDDTVTNFTIWAAGEPNNAKTEEDCVLMSQQWHDVKCNTIRNFACQYNV

>SmLec-12a

LNYTDSIKYCDQMGGEIVSIGNDAEQDAIRNLLVDEKAFDSTCIHKAYLGIHEDISKQWRSGDGEFVSGISEMFFMVDEGSEKCAAIQRPGYFKLNNVQCTGTSYCQICEYDN

>SmLec-12b

YAFSLKECQSDGGRLAQVSSIKQLEEIQDLLGKDAHYSEKCNSGFYVDLTSEKRYYKTPANETVNYTKFVDDSRRSMDRHCAVVHELQRYMWSDQDCSLASCFLCRMTG

>SmLec-12c

LVNNAEARAICRNNFGSLASLVSPFERNFIVDFLMNRSNVTESTSTWFVESTGGLPSGSDDDIGYPTNLGGSATQCVSVHVSPRVGWSVIDCEAKAGFICHD

>SmLec-13

TWLDAVRFCGDLGADSISLETQAETEAIYGEIKRIHGVGGEGAAYWTSGNNFEQTGGEFNWWHGLKPFNYTNWCHLQPDNMGLIENCVLIWYWPRGYSCWNDRFCKTNLICVSCNK

>SmLec-14

TGTYLEGYEYCKARDSELISIETKVESDIINRFIVDSHNPSAPGNFWTSGFDFNRNRQFYWMGTGTNFSDTNWCSGEPNGPTYEYCVHIINNNVLLGTGTKFSHINWCSGEPNGPTYEYCVHIIN

>SmLec-15a

GIPFFNAYRICASNNQHLATIDNEELHIQMNQQIESRDVGTNYWIGLKSFKGNYGLPISNWHWNGTGKSLKFKAWCPPETATREHHKYEWCGYLDSEKKCWRTKPCDRDTEQNIHGFICE

>SmLec-15b

SATWYEAFDVCSQHNKKLLTLDNKKLSDHFKKEMAKRSTDLVWIGLHSYPEIIDIETYLYWQWNGTGQDLDYKDWCVNEPLIAVSMCGYMATNGKVQCWKSVKCSNAGITSFICEDL

>SmLec-16

QGVNFFEAYRFCSDNKQNLANIDNEKLSTDMMNEVKNRKINYWIGLKSFKQYGIQVNKWHWNSTSNEVTYKNWCPPQTNKDNEYYKNEWCGYINVNEQCWKTDVCDRDSKIVSGFIC

>SmLec-17

KGGNFNTARQYCKNRGGDLTHHITNVTQNFLATEMERLRSRMSQVLLWLGMTKVPGSKVRTWRWVTGQQVVSPLWGDDQPNNYNGEQNCAVYDGGRNWLWNDVGCNLDYLLWICQYGPP

>SmLec-18

HIAAYRICNDNNQQLANAEIEDLATALINETNNRKASYWIGLKNFDIYGIPIKKWHWNSTRTAVTYQNWCAPQTHKLNPQYKNEWCGYIHKDEQCWKTEACDRDSRNVNGFICESSI

>SmLec-19

CRYNSYWAGIRQVEDSKWVFMDYSVLGNFSYWAPKEPDGNENEDCVLLAAHVQTKWVTTQCHWGYRFICQLVDAC

>SmLec-20

VTWKEADQRCNSNSQKLVCIENNEEFDFIKTQVKKTGINYWIGLTATEDTTTKLIEWSWIPKRHRNYFSNWCAPDGLQLGAFHHCAFIRADEQCWQAWECDTNYGAANGFICKNM

>TuLec-1

GGSFNDAENYCKARGGLVVNSVGDVTQNFLQYELQRLKAKLKSRLVWLGAKREIPANQPVTHRSRSNVWRWVNGGLITQFLWADDQPNNYNGQQNCIVLDGGRKWQWNDVTCDLDYLPWICQYTP

>TuLec-2

NDKPMNFENARRFCDLRGGSLVDETSPALQGFLSWELYRRHRSDQYGQYWLGAVRDTASPNNWKWINGKDVSISFWSQPRQNFNCSRFDGTRGWLWADTNCNLPIHFVCQHRPL

>TuLec-3

SGSYDENLKYCNGLNASMITIESSEENDEIFKQFASLNIFRFWIAGKTEGILLNEIHQTKIDLMKSTYFSNWGQGSPNCPIGEECCIMVDSQKQWHNYVCSNDLYFACEKRRIFESSE

>TuLec-4

DNLKYCNGLNASMITIESSEENDEIFKQFASLHIFRFWISGRTEGFKLNKKHQTKVDLMKSTDFSNWGLNSPHCTPGEQCCIMVNSFKQWQDYDCLEDFNFACEKRRI

**O. I87.**

>ApBand7-1

KVVQEYERAVIFRLGRLVSGGAKGPGIFFILPCIDNYARVDLRTRTYDVPPQEVLTKDSVTVSVDAVVYYRVCNATISVANVANAHQSTRLLAQTTLRNVLGTRPLHEILSDRDAISKTMQVSLDEATESWGIKVERVEIKDVRLPVQLQRAMAAEAEAAREARAKVIAAEGEQ

>ApBand7-2

NTGILFVPQQEAWIVERMGKFNRILEPGLNFLIPFLDRIGYVQSLKELAIDIPKQTAVTLDNVTLNIDGVLYLRVNDPYLASYGVEDPEFAITQLAQTTMRSELGKISLDKVFRERENLNFAIVESLNKASASWGLVCFRYEIRDIKLPNRVQEAMQMQVEAERKKRAAILDSEGIREAD

>ApBand7-3

FCLHRVDEGHVAVYYRGGALLSQISYPGYHIMMPFLTTFRSVQVTLQTDEVKNVPCGTSGGVMIYFDRIEVVNILNASSVFDIVKNYTADYDKTLIFNKVHHELNQFCSVHNLHEVYIDLFDQIDENLKVALQKDLTEMAPGLKVHAVRVTKPKIPETIRKNYEIMEAEKTKLLIAEQRQKVVEKEAETER

>ApBand7-4

SVANTALYNVDGGHRAVIFDRFTGIKNTVVGEGTHFLIPWVQKPIIFDVRSRPRNVPVITGSKDLQNVNITLRILFRPLPEQLPKIYTILGVDYDERVLPSITTEVLKAVVAQFDAGELITQRENVSRKVSETLIERAGQFGVVLDDISITHLTFGKEFTQAVELKQVAQQDAERARFLVEKAEQQKQ

>ApBand7-5

WGFVTCGPNEALVISGFCYGKPNLVPGGRAFVWPVIQYCQRICLNTMTIQVDSPKVYTIQGVPLSVTGIAQVKIQGQNEEMLLTACEQFLGKPKQEIHEIALHTLEGHQRAIMGSMTVEEIYKDRKKFSKQVFEVASSDLVNMGITVVSYTIKDIRDEEGYLRALGLARTAEVKRDARIGEAEAKRETT

>ApBand7-6

NSMFTVEGGHRAIMFNRIGGIQREVYPEGLHFRLPWFQYPVIFDIRSRPRKISSPTGSKDLQMVNISLRVLSRPDAIKLPDMYQHLGIDYDEKVLPSICNEVLKSVVAKYNASQLITQRQQVSLLIRKQLVDRARDFNIILDDVSITELSFGKEYTAAVEAKQVAHQEAQRAVFFVERAKQERQQKIL

>AgBand7-1

CFKVVQEYERAVIFRLGRLMQGGAKGPGIFFILPCIDAYARVDLRTRTYDVPPQEVLTKDSVTVSVDAVVYYRVSNATVSIANVENAHHSTRLLAQTTLRNTMGTRHLHEILSERMTISGSMQLSLDEATEAWGIKVERVEIKDVRLPVQLQRAMAAEAEAAREARAKVIAAEGEQKASR

>AgBand7-2

CFKVVQEYERAVIFRLGRLRSGGARGPGVFFVLPCIDNYCKVDLRTVSFDVPPQEVLTRDSVTVSVDAVVYYRIRDPLNAVVQVANYSHSTRLLAATTLRNVLGTRNLSELLTEREAISHSMQVTLDEATDPWGVQVERVEIKDVSLPDSLQRSMAAEAEAAREARAKVIAAEGEMKS

>AgBand7-3

IMFVPQQEAWIVERMGKFHRILEPGLNVLLPVVDRVKYVQSLKEIAIDVPKQSAITSDNVTLSIDGVLYLRILDPYLASYGVEDPEFAITQLAQTTMRSELGKMSLDKVFRERESLNISIVESINKASEAWGISCLRYEIRDIKLPSRVHEAMQMQVEAERRKRAAILESEGVR

>AgBand7-4

LYNVDGGHRAVIFDRFSGVKQQVTGEGTHFFVPWVQRPIIFDIRSQPRNVPVITGSKDLQNVNITLRILFRPVPDQLPKIYTILGQDYDERVLPSITTEVLKAVVAQFDAGELITQREMVSQKVSDDLTERAAQFGVILDDISITHLTFGKEFTQAVEMKQVAQQEAEKARFMVEKAEQMKQAAI

>AgBand7-5

GFVTCGPNEALVVSGCCHMKPLLVPGGRAFVWPSIQQVQRISLNTMTLQVESPTVYTSQGVPISVTGIAQVKIQGQNEDMLLTACEQFLGKSEAEIQHIALVTLEGHQRAIMGSMTVEEIYKDRKKFSKQVFEVASSDLVNMGITVVSYTLKDIRDEEFNGSNRGYLKSLGMARTAEVKRDARIGEAEARCD

>AmBand7-1

MVVQEYERAVIFRLGRLLSGGAKGPGIFFILPCVDNYARVDLRTRTYDVPPQEVLTKDSVTVSVDAVVYYRVNNATISITNVENAHHSTKLLAQTTLRNTMGTRPLHEILSERETISGNMQVSLDEATDTWGIKVERVEIKDVRLPVQLQRAMAAEAEAAREARAKVIAAEGEQK

>AmBand7-2

CFTFKVVQEYERAVVFRMGRLKGAAYGPGTFFVMPCVDNCVRVDLRTVSFDVPPQEVLTKDSVTVSVDAVVYYRIKEPLNAVIKIANYSHSTRLLAASTLRTVLGTRNLAEILSERETISHTMQTSLDEATEPWGVKVERVEIKDVRLPVQLQRAMATEAEAAREARAKVIAAEGEMLAS

>AmBand7-3

LFVPQQEAWIVERMGKFHRILNPGLNILTPIIDKIKYVQCLKEIAIEIPQQSAVTSDNVTLNIDGILYLRVVNPFLASYGVDDPEFAVVQLAQTTMRSELGKISLDKVFREREGLNVCIVDSINKASEAWGITCLRYEIRDIRLPQRVQEAMQMQVEAERKKRAAVLESEGAREAEIN

>AmBand7-4

LYNVDGGHRAVIFDRFTGIKNQVVGEGTHFIIPWVQRPIIFDVRSRPRNIPVITGSKDLQNVNITLRILFRPIPDSLPKIYTVLGIDYAERVLPSITNEVLKAVVAQFDAGELITQREIVSQKVREDLTERATQFGLILDDISITHLTFGKEFTQAVEMKQVAQQEAEKARFLVEKAEQH

>AmBand7-5

GFVTCGPNEALVVSGCCYSKPLLVPGGRVFVWPIVQQVQKISLNTMTLQVESPTVYTCQGVPISVTGIAQVKIQGQNEEMLSTACEQFLGKTEEEIHNIALVTLEGHQRAIMGSMTVEEIYKDRKKFSKEVFEVASSDLVNMGITVVSYTLKDIRDEEGAKGYLKALGMARTAEVKRDARIGEAEARRD

>AmBand7-6

SMYTVEAGHRAIIFSRLGGIQQDILTEGLHFRIPWFHWPIIYDIRSRPRKLSSPTGSKDLQMVNISLRVLSRPDAQSLPTMYRQLGLDYDEKVLPSICNEVLKSVVAKFNASQLITQRQQVSNLVRKELTERARDFNIVLDDVSITELSFGKEYTAAVESKQVAQQEAQRAAFFVEKAKQEKQQKIVQA

>AmBand7-7

NVHTCGPNEALVVSGGCCGSMKKRTIVGGYAFTWWFVTDVQRLSLEVMTLNPVCESVETAQGVPLTVTGVAQCKIMKADELLHTASEQFLGKSVYEIKSTILSTLEGHLRAILGTLSVEEVYKDRDQFATLVREVAAPDVGRMGIEILSFTIKDVYDDVQYLASLGKAQTAAVKRDADVGVAEANRD

>BmBand7-1

CFKVVQEFERAVIFRLGRLRKGGARGPGLFFVLPCIDTYRKVDLRTVSFDVPPQEVLTRDSVTVAVDAVVYYRIKEPLNAVVRVADYSASTRLLAATTLRNVLGMRDLAQLLSDREAISHMMQASLDEATEPWGVEVERVEIKDVRLPVQLQKAMAAEAEADREARAKIIAAEGEIKA

>BmBand7-2

CFKVVQEYERAVIFRLGRLLSGGAKGPGIFFILPCIDTYARVDLRTRTYDVPPQEVLTKDSVTVSVDAVVYYRVHNATISIANVENAHHSTRLLAQTTLRNTMGTRPLHEILSERETISGNMQLSLDEATEAWGIKVERVEIKDVRLPVQLQRAMAAEAEAAREARAKVIAAEGEQKAS

>BmBand7-3

LYNVDGGHRAVIFDRFAGVKQLVVGEGTHFFIPWVQRPIIFDIRSRPRNVPTITGSKDLQNVNITLRILFRPVPDQLPRIYTILGIDYDERVLPSITSEVLKAVVAQFDAGELITQREIVSQKVNDSLTERAAQFGLILDDISITHLTFGKEFTQAVELKQVAQQEAEKARFLVEKAEQQK

>BmBand7-4

VTCGPNEALVISGCCYSKPLLVPGGRAFVWPAIQSVQRISLNTMTLQVESPTVYTSQGVPISVTGIAQVKIQGQNSEMLLSACEQFLGKTEQEIQHIALVTLEGHQRAIMGSMTVEEIYKDRKIFSKKVFEVASSDLINMGITVVSYTLKDIRDEEGYLKALGMARTAEVKRDARIGEAEAQAE

>CfBand7-1

CFKVVQEYERAVIFRLGRLLSGGAKGPGIFFILPCVDNYARVDLRTRTYDVPPQEVLTKDSVTVSVDAVVYYRVNNATISIANVENAHHSTRLLAQTTLRNTMGTRPLHEILSERETISGNMQVALDDATDTWGIKVERVEIKDVRLPVQLQRAMAAEAEAAREARAKVIAAEGEQK

>CfBand7-2

FVPQQQAWIVERMGKFHKILEPGLNILFPVVDKVKYVQILKEMAIDVPQQSAVTSDNVTLSIDAVLYLKVTDPYLTSYGVEDAEFAIIQVAQTTMRSELGKIPLDKVFREREELNVSIVESINKASNAWGITCLRYEIRDIRFPPRVQEAMQMQVEAERKKRAAILESEGV

>CfBand7-3

NSALYNVDGGHRAVIFDRFAGIKNAVIGEGTHFFIPWVQKPIIFDIRSRPRNVPVITGSKDLQNVNITLRILFRPVPDSLPKIYTILGVDYDERVLPSITTEVLKAVVAQFDAGELITQRELVSQKVSDDLTDRASQFGLILDDISITHLTFGKEFTQAVELKQVAQQDAEKARFLVEKAEQQ

>DpBand7-1

CFSVKVVQEYERAVIFRLGRLLKGGARGPGIFFIVPCIDTYRKIDLRTVSFDVPPQEILSRDSVTVAVDAVVYYRVHNPTIAVSNVENFSHSTRLLAATTLRNVLGTKNLAEVLSERETISHTMQSSLDEATDPWGVKVERVEIKDVRLPVQLQRAMAAEAEAAREARAKVIAAEGEQKASHAL

>DpBand7-2

ICFKVVQEYERAVIFRLGRLLSGGAKGPGIFFILPCIETYTKVDLRTGVFDIPPQEVLTKDSVTVSVDAVVYFRVSNATVSVANVENAHHSTRLLAQTTLRNILGTKDLHEILGDRETISGSMQAALDEATESWGIKVERVEIKDVRLPVQLQRAMAAEAEASREARAKVIAAEGEFK

>DpBand7-3

TIMLFVPQQEAWVVERMGKFHKILKPGLNFLIPVLDNIKYVQSLKEIAIDVPQQSAITLDNVTLSIDGVLYLRIVDPYKASYGVEDAEFAITQLAQTTMRSELGKIHLDSVFRERENLNLGIVEAINKASEAWGIACLRYEIRDIKLPARVQEAMQMQVEAERKKRAAILESEGIRE

>DpBand7-4

YNVEGGHRAVIFDRFSGVKNEVVGEGTHFFVPWVQKPIIYDIRSRPRNVPVITGSKDLQNVNITLRVLFRPVPTSLPNIYSTLGIDYDERVLPSITNEILKAVVAQFDAGELITQREVVSQKVSEALTERAGQFGLILDDISITHLTFGKEFTQAVELKQVAQQEAERARFLVEKAEQL

>DmBand7-1

CFKVVQEYERAVIFRLGRLMQGGAKGPGIFFILPCIDSYARVDLRTRTYDVPPQEVLTKDSVTVSVDAVVYYRVSNATVSIANVENAHHSTRLLAQTTLRNTMGTRHLHEILSERMTISGTMQVQLDEATDAWGIKVERVEIKDVRLPVQLQRAMAAEAEAAREARAKVIAAEGEQ

>DmBand7-2

CFKVVSEYERAVIFRMGRLRSGGARGPGVFFVLPCVDDYYPVDLRTVSFDVPPQEVLSKDSVTVTVDAVVYYRISDPLKAVIQVYNYSHSTSLLAATTLRNVLGTRNLSELLTERETISHTMQMSLDEATDPWGVKVERVEIKDVSLPTALQRAMAAEAEAAREARAKVIAAEGEMKS

>DmBand7-3

CFKVVAEYERAIIFRLGRLSGGARGPGMFFILPCIDEYRKVDLRTVTFNVPQQEMLTKDSVTVTVDAVVYYRISDPLYAVIQVEDYSMSTRLLAATTLRNIVGTRNLSELLTERETLAHNMQATLDEATEPWGVMVERVEIKDVSLPVSMQRAMAAEAEAARDARAKVIAAEGEKKS

>DmBand7-4

TIVPEYSRMIILRLGRLRKGLRGPGLVFILPCIDETHRVDMRTDVTNVRPQDVLTKDSVTITVNAVVYYCIYSPIDSIIQVDDAKQATQLISQVTLRNIVGSKTLNVLLTSRQQLSREIQQAVAGITYRWGVRVERVDVMDITLPTSLERSLASEAEAVREARAKIILAEGELKASK

>DmBand7-5

YEFHRLVIFRLGRIRSCLGPGLVFLLPCIDSFNTVDIRTDVVNVDPQEMLTKDSVSITVNAVVFYCIYDPINSIIKVDDARDATERISQVTLRNIVGSKGLHELLASRQQLSLEIQQAVAKITERWGVRVERVDLMEISLPSSLERSLASEAEATREARAKIILAEGEAKA

>DmBand7-6

SEYERAVILRLGRLRPKPPRGPGVIFLVPCIDDLAVVDIRTRSFDLHRQEILTRDMVTISIDGVVYYSIKSPFDAMLQVYDPEEATEKLAMTTLRNVAGTHKLMDLLSSKEYLSNQIEGILYNSTEPWGIRVERVEIKEIFMPDQLKRALAVEQEAMREAKAKVAAAQGERDAV

>DmBand7-7

CVMFVPQQEAWVVERMGRFHRILDPGLNILVPVADKIKYVQSLKEIAIDVPKQSAITSDNVTLSIDGVLYLRIIDPYKASYGVEDPEFAITQLAQTTMRSELGKMSMDKVFRERESLNVSIVDSINKASEAWGIACLRYEIRDIRLPTRVHEAMQMQVEAERRKRAAILESEG

>DmBand7-8

LYNVEGGHRAVIFDRFTGIKENVVGEGTHFFIPWVQRPIIFDIRSQPRNVPVITGSKDLQNVNITLRILYRPIPDQLPKIYTILGQDYDERVLPSIAPEVLKAVVAQFDAGELITQREMVSQRVSQELTVRAKQFGFILDDISLTHLTFGREFTLAVEMKQVAQQEAEKARFVVEKAEQQKLASI

>DmBand7-9

GFVTCGPNEALVVSGCCYMKPLLVPGGRAFVWPVGQQVQRISLNTMTLQVESPCVYTSQGVPISVTGIAQVKVQGQNEDMLLTACEQFLGKSEAEINHIALVTLEGHQRAIMGSMTVEEIYKDRKKFSKQVFEVASSDLANMGITVVSYTIKDLRDEEGYLRSLGMARTAEVKRDARIGEAEARAE

>DmBand7-10

QSLYTVEGGHRAIIFSRLGGIQSDIYSEGLHVRIPWFQYPIIYDIRSRPRKISSPTGSKDLQMINISLRVLSRPDSLNLPYLHKQLGVDYDEKVLPSICNEVLKSVIAKFNASQLITQRQQVSLLIRKELVERARDFNIILDDVSLTELSFGKEYTAAIEAKQVAQQEAQRAVFFVERAKQEKQ

>IsBand7-1

CVKIVQEYERAVIFRLGRLVKGGARGPGLFFIIPCIDNYTKVDLRTVSFDVPPQEILTKDSVTVAVDAVVYYRIQNATVAVTNVEDYGRSTRLLAATTLRNVLGTKNLSEILSEREPISHTMQTNLDEATDAWGVKVERVEIKDVRLPVQMQRAMAAEAEASREARAKVIAAEGEQRAA

>IsBand7-2

CIVIVKEYERAVIFRMGRLLPGGAKGPGLFFIVPCTDNYSVVELRTWAFDVPPQEVLSKDSVTLAVDAVVYYRVFNPVIAITNVQDFARSTKLLASSILRNVLGTKSLSEMLSERDSISQLMQSTLDAATDPWGVKVERVEMKDFRIPVQMQRAMAAEAEAMREGRAKVIAAEGEQRAS

>IsBand7-3

CLVVVQEFERAVIFRLGRLQPGGAAGPGLFFIIPCIDEYRVVDLRTVVFNVCPQEILSKDSVTVAVDAVVYYRVFNPVAATVNIKDHARSTILLAATILRNVLGTKMLSDVLSQRKSISRTMQTLLDVATDPWGVKVERVELTDVQLPAQMQRAMAAEAEAVREGRAKVVAAEGEQRA

>IsBand7-4

CIVIANEYQRVVIFRLGRLVSGGARGPGLFFIIPCVDRYCEIDLRTISIDVPAQEILSRDSVTVTVDAVIYYRIVNPIASVMNVEDYFVATNLLAAAMLRNVLGTKNLSDILSDRESISQMMQSALDVATDPWGVKVERVEIKDVRLPHQMQRAMAAEAEAVREGRAKVVAAEGEER

>IsBand7-5

VKVVPQQQAWVVEKLGKFDKVLQPGLNLLIPVIQRVAYKHTLKEEAIDVTAQTAISNDNVTLSIDGVLYVKIIDPMAASYGVNNPYYAITQLAQTTMRSEIGKLPLDRTFEERETLNVAIVAAINQAAINWGIQCMRYEIKDIQPPQTILKAMELQVAAERQKRAQILESEGNRQAKINHAEGEKA

>IsBand7-6

AHGQVSRILEPGLNLLLPIVDRVRYVQSLKELAIDVPQQSAITLDNVTLNIDGVLYLKVVDPYRASYGVEDPEFAITQLAQTTMRSELGKIALDSVFKERESLNIAIVDAINKASGAWGIVCLRYEIRDIRLPQRVHEAMQMQVEAERKKRAAVLESEGI

>IsBand7-7

LFSVDQRQSAVVFQFGEAVRTIENPGLNIKIPFIQNVEFFDKRLLDVEVEAKELTAADGKRVIVDAYAKFQINNPVMFYKTVHDYQGVKIRLTRNLESSMRKVIGKISLSSLLSQERINVMLNILNQVDGEAKSFGIDVVDVRILRADLPKENSAAIYRRMQTAREKEATQIRAEGQEE

>IsBand7-8

KGFVDGGHRAVIFDRFTGVKNYVVGEGTHFLIPWVQRPIIYDVRSRPRNVPVVTGSKDLQNVNITLRILFRPVQEQLPRMYTTLGVDYDERVLPSITNEVLKAVVAQFDASEMITQREVVSQKVCDELTERASQFGVILDDISITHLTFGKEFTQAVEMKQVAQQEAERARFLVEKAEQQK

>IsBand7-9

SVFTVDGGHRAIIFNRIGGIQKDVFAEGLHFRIPWIQYPIIYDIRSRPRKISSPTGSKDLQMVNISLRVLARPDAIMLPTVYRMLGTDYDERVLPSICNEVLKSVVAKFNASQLITQRQQVSLLVRRELTERARDFNIILDDVSITELSFGKEYAAAVEAKQVAQQEAQRAMFTVEQAVQERQQKI

>NvBand7-1

CVIFKVVQEYERAVVFRMGRLKAGPQGPGTFFVIPCIDNCVRVDLRTVSFDVPPQEVLTKDSVTVSVDAVVYYRIKEPLNAVVKIANYSHSTRLLAASTLRTVLGTRSLAEILAERETISHTMQAALDEATEPWGVKVERVEIKDVRLPVQLQRAMAAEAEAAREARAKVIAAEGEM

>NvBand7-2

CFKVVQEYERAVIFRLGRLLSGGAKGPGIFFILPCVDSYARVDLRTRTYDVPPQEVLTKDSVTVSVDAVVYYRVNNATISIANVENAHHSTRLLAQTTLRNTMGTRPLHEILSERETISGNMQISLDEATDSWGIKVERVEIKDVRLPVQLQRAMAAEAEAAREARAKVIAAEGEQK

>NvBand7-3

VMFVPQQEAWIVERMGKFHRILEPGLNLLIPVIDSVRYVQSLKEIAIDVPKQSAITSDNVTLSIDGVLYLKINNPYLASYGVQDPEFAIIQLAQTTMRSELGKIALDKVFQEREGLNISIVESINKASEAWGISCLRYEIRDIKLPERVHVAMQMQVEAERKKRAAILESEGI

>NvBand7-4

YNVDGGHRAVIFDRFVGVKNNVTGEGTHFFIPWIQKPIIFDIRSRPRNVPVITGSKDLQNVNITLRILFRPVPESLPKIYTILGVDYDERVLPSITTEVLKAVVAQFDAGELITQRELVSQKVSEDLTERASQFGVILDDISITHLTFGKEFTQAVELKQVAQQEAEKARFLVEKAEQQ

>PhBand7-1

CFKVVQEYERAVIFRLGRLLSGGAKGPGIFFILPCVDNYAKVDLRSSVFDIRPQEVLTKDSVTVSVDAVVYYRVCNATISVANVENAHHSTRLLAQTTLRNTMGTRLLSEILSERENISQVMQSALDDATVAWGIKVERVEIKDVRLPIQLQRAMAAEAEASREARAKVIAAEGEQK

>PhBand7-2

FRVVQEYERAVIFRLGRLRKGGPRGPGIFFVLPCIDSYSKVDLRTVSFDVPPQEVLTKDSVTVTVDAVVYYNIKDPLSAVVQVSNYSHSTQLLAATTLRNVLGTKNLSEILSERETIAHTMQTSLDEATDPWGVKVERVEIKDVRLPVLLQKAMAAEAEAAREACAKVIAAEGEMKASKA

>PhBand7-3

GFVTCGPNEALVVSGCCYNKPLLVPGGRAFVWPGIQEVQRISLNTMTLQVESPTVYTSQGVPISVTGIAQVKIQGQNEEMLTAACEQFLGKSENEIQNIALVTLEGHQRAIMGSMTVEEIYKDRKKFSKHVFEVASSDLVNMGITVVSYTLKDIRDEEGYLKSLGKARTAEVKRDARIGEAEARRDAQ

>PhBand7-4

SLYTVEGGHRAIIFSRIGGIQKEVYSEGLHFKIPWLEYPIIYDIRSRPRKISSPTGSKDLQMVMISLRVLSRPDAINLPTMYRTLGLDYDEKVLPSICNEVLKSVVAKFNASQLITQRQQVSLLVRRELTERARDFNIILDDVSITELSFGKEYTAAVEAKQVAQQEAQRAAFVVERAKQER

>PhBand7-5

LYNVDGGHRAVIFDRFAGVKNQVIGEGTHFFIPWVQRPIIFDTRSRPRNVPVITGSKGNIVIIPLPEQLPRIYTILGVDYDERVLPSITTEVLKAVVAQFDAGELITQREVVSQKVSEELTDRASQFGVILDDISITHLTFGKEFTQAVELKQVAQQEAEKARFLVEKAEQN

>RpBand7-1

VFKVVQEYERAVIFRLGRLRAGGARGPGIFFVLPCIDVYAKVDLRTVSFDVPPQEVLTKDSCTVCVDAVVYYRIEDPLRSVVAISNYSHSTRLLAATTLRNVLGTRNLAEILAEREVISHTMQTALDLATEPWGVKVERVEIKDVRLPVQMQRAMAAEAEATREARAKVIAAEGEMKA

>RpBand7-2

VNQFERAVILRFGKLRKGRARGPGLIFVLVCIDNVMKVDLRTATYAIPPQEVLTKDSCTVSVDAVVYYSVSDPIRAVVQVVSFRYSTCTLAATILRNIMGQKNLTEILSERESIAFVIKEALDSATHPWGITVERVEIKDVRLPVQMQRAMAAEAEATRDARAKVIAAEGEL

>RpBand7-3

CKIAYQYERVVILRLGRVREGGPRGPGIYLYLPCVDERMKLDLRTLVFQLDPQQVLTNDSCTLQIDVIIFCRVEDPVKTVVAVSDLRVATLNLASTILRNIIGQRDLTEILSQKDAITLALKKILDVGTFPWGVKVLRVELMGIRLPLNMQRAMASEAEATREAKAKMIFAEGELL

>RpBand7-4

FIPQQEAWIVERMGKFHRILEPGLNILFPILDSVKYVQSLKEMAIDIPKQSAITSDNVTLNIDGVLYLRVLDPYLASYGVEDPEFAITQLAQTTMRSEIGKISLDNVFRERESLNVGIVASINKASEAWGITCLRYEIRDIKLPVRVQEAMQMQVEAERKKRAAILESEGVR

>RpBand7-5

LYNVDGGHRAVIFDRFTGVKNQVVGEGTHFFIPWIQRPIIFDIRSRPRNIPTVTGSKDLQNVNITLRILFRPVPDQLPKIYTILGVDYDERVLPSITTEVLKAVVAQFDAGELITQRELVSQKVNEELTERAAQFGVILDDIALTHLTFGKEFTQAVELKQVAQQEAERARFLVEKAEQQKQ

>RpBand7-6

FVTCGPNEALVVSGCCHTKPLLVPGGRAFVWPTLQCVQRISLNTMTLQVESPTVYTSQGVPISVTGIAQVKIQGQNEEMLLAACEQFLGKSDAEIQHIALVTLEGHQRAIMGSMTVEEIYKDRKKFSKHVFEVASSDLVNMGITVVSYTLKDIRDEEGYLKSLGMARTAEVKRDARIGEAEARKD

>RpBand7-7

SMYTVEGGHRAIIFSRLNGIQKDVFSEGLHFRVPWFQYPIIYDIRSRPRKISSPTGSKDLQMVNISLRVLSRPDSLQLPFMYRQLGLDYDEKVLPSICNEVLKSVVAKFNASQLITQRQQVSLLVRRELIERARDFNIILDDVSITELSFGKEYTAAVEAKQVAQQEAQRAVFVVERAKQERQQ

>SmBand7-1

CVKVVQEYERAVIFRLGRLVKGGARGPGIFFIIPCIDTYRKVDLRTVSFDVAPQEILSKDSVTVAVDAVVYYRISNATIAVANVEDYGHATRLLAATTLRNVLGTKNLSEILAERETISHTMQAVLDEATDPWGVKVERVEMKDVRLPVQLQRAMAAEAEATREARAKVIAAEGEQR

>SmBand7-2

CLKVVQEYERAVIFRLGRLLSGGAKGPGLLFILPCIEDFVNIDMRTLTFDVPPQEVLTKDSVTVSVDAVVYYRVSNATISVANVENAHHSTRLLAQTTLRNMLGTKNLHEILSDRENISATMQSNLDDGTEGWGIKVERVEIKDVRLPVQLQRAMAAEAEAAREARAKVIAAEGEQKA

>SmBand7-3

CWVVIKEYERAITFRFGRLRKIEPRGPGLIFILPCVDTYNVVDLRTLYFDVPPQEILTKDSVTIAVDAVVYYRTFDATMAITNVQDYKKASHLLAASILRNTLGTKNMVDILTQRESLSYAMQKQLDEATDPWGVKIERVEMKDVRLPHNMQRAMAAEAEATREAKA

>SmBand7-4

LYNVDGGHRAVIFDRFTGVKNFVVGEGTHFLIPWVQKPIIYDIRSRPRNVPVVTGSKDLQNVNITLRILFRPIPDTLPKLYMSLGSDYDERVLPSITNEVLKAVVAQFDASELITQREIVSQRVSEALIERAGQFGLFLDDISITHLTFGKEFTHAVELKQVAQQDAERARYLVEKAEQVK

>SmBand7-5

FVTCGPNEALVVSGCCHSKPLLVPGGRAFVWPFVQQVQRISLNTITLVVDSPKVYTNQGVAISVTGIAQVKIQGQNEDMLMTACQQFLGKTEDQVKGIALVTLEGHQRAIMGTMTVEEIYKDRKKFSKQVFEVASSDLVNMGITVVSYTLKDIRDDEGYLKALGMARTAEVKRDARIGEADARRDG

>TuBand7-1

KSYVVQDYERSVTLRLGNLVGDGAKGSGIIFIIPCIDTYCKVDLRTVSFNVPPQEVLTKDLVCIKVDAVVYYRIVDSTASILNVSDVGRSTRRLAAITLRNVVATKTLTEIISERVIISSKIENNLQEATRPWGVDVERVEMKEVRLPAQLQKLMATEAETEREASAKIIIASGEQK

>TuBand7-2

CVKVVQEYERAVIFRLGRLLDGGAKGPGIFIILPCIENYTKVDLRTLTFDVPPQEVLTKDSVTVSVDAVVYYRVFNATVSISNVENAHHSTRLLAQTTLRNILGTFNLQEVLISRESISTSMQTVLDEATERWGIKVERTEITDVRLPVQLQRAMAAEAEAAREARAKVIAAEGEQK

>TuBand7-3

VEIIKEYERAVVFRLGRLKKGGVEGPGVHVLIPCIDTLSKVDLRTITFDVPPQEILTSDSVTVSVDAVVYFRICNPIFAVTNVQDYRRSTQLLAATTLRNVLGGKSLSQLLSELDTISQILKSNLDTTTESWGVKVERVEIKDIRLPTQLQRAMAAEAEAMREARAKVIASEGEQKASR

>TuBand7-4

CIKVVQEYERAVIFRLGRLVTGGARGPGIFFIIPCIDTYSKVDLRTVSFDVPPQEILSKDSVTVAVDAVVYFRISNAIASVSNVEDYARSTRLLAATTLRNVLGTKNLSEILSERESISHMIQSSLDEATDSWGVKVERVEIKDVRLPVQLQRAMAAEAEAAREARAKVIAAEGEQRAS

>TuBand7-5

NIVVQEYERAVIFRLGRLVKGGAKGPGIFFIIPCIDTYSKVDLRTVSFDVPPQEILSKDSVTVAVDAVVYYRISNATIAVSNVEDYGRSTRLLAATTLRNVLGTKNLSEILSERESISHIMQSSLDEATDPWGVKVERVEIKDARLPVQLQRAMATEAEAAREARAKVIAAEGEQR

>TuBand7-6

VKVVQEYERAVIFRLGRLVKGGARGPGIFFIIPCIDSYCKIDLRTVTFDVPPQEILSKDSVTVAVDAVVYYRISNATVAVTNVADYGMSTRLLAATTLRNVLGTKNLSELLSERESISHMIQSSLDVATDPWGVKVERVEVKDVRLPQQLQRAMAAEAEASREARAKVIAAEGEQK

>TuBand7-7

CIKVVQEYERAVIFRLGRLLKGGARGPGIFFVIPCIDSYTKVDLRTVTFDVPPQEVLSKDSVTTTVDAVVYYRISNATVAVTNVEDYGRSTRLLAATTLRNVLGTKNLSELLSERESISHMIQSSLDEATDPWGVKVERVEVKDVRLPQQLQRAMAAEAEASREARAKVIAAEGEQKA

>TuBand7-8

CVKVVQEYERAVIFRLGRLLSGGAKGPGIFIILPCIENYTKVDLRTLTFDVPPQEILTKDSVTVSVDAVVYYRVNNATISIANVENAHHSTRLLAQTTLRNMLGTHNLHEILSDRELISTSMQTVLDEATERWGIKVERTEIKDVRLPVQLQRAMAAEAEAAREARAKVIAAEGEQKA

>TuBand7-9

FCIKVVQEYERAVIFRLGRLLKGGARGPGIFFIIPCIDSYCKIDLRTVSFDVPPQEILSKDSVTVAVDAVVYYRISNATVAVTNVEDYGRSTRLLAATTLRNVLGTKNLSELLSERESISHMIQSSLDEATDPWGVKVERVEVKDVRLPVQLQRAMAAEAEAAREARAKVIAAEGEQKASR

>TuBand7-10

VIFVPQQEAWVVERMGKFHQICEPGLNFLIPIVDRVKYVQSLKEIAIDIPKQSAVTSDNVTLAIDGVLYLRVVDPYKASYGVEDPEFAITQLAQTTMRSEIGKITLDTVFKERETLNIAIVEAINKAGLAWGITCLRYEIRDIKLPERVQEALSMQVEAERRKRAVVLESEGRREAEINVAEGEKQA

>TuBand7-11

CIKVVQEYERAVIFRLGRLVSGGARGPGIFFIIPCIDTYSKVDLRTVSFDVPPQEILSKDSVTVAVDAVVYFRISNAIASVSNVEDYARSTRLLAATTLRNVLGTKNLSEILSERESISHMIQSSLDEATDSWGVKVERVEVKDARLPFQLQRAMAAEAEATREARAKVIAAEGEQRA

>TuBand7-12

LYNVDGGHRAVIFDRFSGVKPNVVGEGTHFLIPWVQKPIIFDVRSRPRNVPVVTGSKDLQNVNITLRILFRPVPENLPKMYSSLGVDYDERVLPSITNEVLKAVVAQFDAGELITQREIVSQRVSEDLTERAAQFGLLLDDISLTHLTFGREFTAAVEMKQVAQQDAEKARFLVEKAEQLKK

>TuBand7-13

CIKVVQEYERAVIFRLGRLVTGGARGPGIFFIIPCIDTYSKVDLRTVSFDVPPQESKVVNYKMDAQKAPAMIEAELSLRHGAMKILSKDSVTVAVDAVVYFRINNAIASVSNVEDYARSTRLLAATTLRNVLGTKNLSEILSERESISHMIQSSLDEATESWGVKVERVEIKDVRLPVLLQRAMAAEAEATREARAKVIAAEGEQR

>TuBand7-14

VKKIKPLERCIIYRLGKRLPIKGPGLVIVIPFVDVIDFIDLNPHRLCVVSKEQMLTSDGSLIEFIDFTVEMTVFNAIRTSTQLKDSRQNVDQFVKLSFLNTMGGIHVEDLERKMEFIIKQYAETCNQYINKWGWSMVVIEIPRIKVL

>TcBand7-1

CFKVVQEYERAVIFRLGRLRTGGARGPGIFFILPCVDSYCKVDLRTVSFDVPPQEALTKDSVTVTVDAVVYYRIQDPLNAVTKVTNYSNSTRLLAMTTLRNILGTRNLAEILSDREAISHAMQTNLDVATDPWGVKVERVEIKDVSLPQQLQRAMAAEAEASREARAKVIAAEGEMKASR

>TcBand7-2

CLKIVQEYERAVIFRLGRLRSGGPRGPGIFFILPCIDDYIKIDLRTVTFDIPPQEVLSKDSVTIWVDAVVYFRVEDPLAAILKVENFRTSTHLLAMTTLRNILGTKTLMEILSDRENIVHLMQTQLDVATDPWGIKVERVEITDIRLPQSLQRAMATEAEASREARAKIIAAEGEMNAAK

>TcBand7-3

CFKVVQEYERAVIFRLGRLLSGGAKGPGIFFILPCIDAYARVDLRTRTYDIPPQEVLTKDSVTVSVDAVVYYRVSNATVSIANVENAHHSTRLLAQTTLRNIMGQRPLHEILSERESISQHMKALLDEATDSWGINVERVEIKDVRLPIQLQRAMAAEAEAAREARAKVIAAEGEQKAS

>TcBand7-4

IMFVPQQEAWVVERMGKFHRILEPGLNVLIPVVDRVKYVQSLKEIAVDIPKQSAITSDNVTLNIDGVLYLRIVDAYLASYGVEDPEFAITQLAQTTMRSELGKISLDKVFRERENLNVSIVDSINKASEAWGMTCLRYEIRDIKLPPRVQEAMQMQVEAERKKRAAILESE

>TcBand7-5

YNVDGGHRAVIFDRFSGIKKQVIGEGTHFFIPWVQRPIIFDVRSRPRNVPVITGSKDLQNVNITLRILFRPVPDQLPRIYTVLGQDYEERVLPSITTEVLKAVVAQFDAGELITQRDLVSQKVSEDLTERASQFGVILDDISITHLTFGREFTLAVELKQVAQQEAEKARFLVEKAEQN

>TcBand7-6

AMYTVEGGHRAIMFNRIGGVQKDIYTEGLHFRVPWFQYPIIYDIRSRPRKISSPTGSKDLQMVNISLRVLSRPNASQLPIVYRQLGLDYDEKVLPSICNEVLKSVVAKFNAAQLITQRQQVSLLVRRELTERARDFNIILDDVSITELSFGKEYTAAVEAKQVAQQEAQRAAFIVEKAKQERQ

**P. I93.**

>ApFz-1

GHHGRCEPITIPFCMGIAYNETIMPNILGQMRQDEAGFEVQQYYPLVKIQCSHDLQFFLCSVFAPVCTIIEKPIPPCRSLCVSARNGCEAIMNRFQIEWPDNLECNQFPENGQLCVGEN

>ApFz-2

XGRCEEITIPMCRGIGYNMTSMPNQLNHETQEEAGMEVHQFWPLVEINCSADLKFFLCSVYTPICIEEYQRPLQACRSVCERARDGCLPVMQRYGFVWPEKMQCDKLPVHGGPELCMAQD

>ApFz-3

PRRCTPVRLNLCKSVLEYNLTSYPNHFGHKNLDEIHDDLIAFRDLVDAECYQRTLDFVCQLLQPACKPDVKLDRDEITLPCKHSCRLFTTGCGHRIPEKLLAGFDCAKLPDYSNIGSTCTSK

>ApFz-4

NHSLSCLGVKLPYTSTSTDLVPDAETPEEAQERLHYWQGLKKVPKCWAAIQPLLCALYMPNCENSSLHLPPQEMCKLIENHCQILKIENSWPAEFNCENDTIYPAKLCKND

>AgFz-1

QRTCEPIRIELCRGIGYNETSLPNIVGHELQSDANFTLQTFFPLIQFGCSKQLKFFLCATYVPMCTPKVSMPIGPCRSLCNTVKNRCHPILQGFGFPWPSALDCNRFPEENNHEHMCMQG

>AgFz-2

SRCEEITIPMCRGIGYNLTSFPNEMNHETQEEAGLEVHQFWPLVEIKCSPDLKFFLCSMYTPICIEDYHKPLPVCRSVCERARAGCAPIMESYSFNWPERMACENLPVSGDSDNLCMEMP

>AgFz-3

NRCEPITIPFCIGIPYNRTIMPNRFGHTKQDEAALEVHQYVPLVKIDCSPDLKFFLCLLYAPVCTILPFPIPPCRSLCESARACESIMKTFNFPWPENLECSQFPEYGGEELCVSKH

>AgFz-4

GVCLPVIVKFCQQHRVPYNYTVFPNYIGHFGQPEAQIEIDLFEALVDVQCYELVPLFLCSLFVPKCGNSGATVPPCKSLCTETMRRCSFFFDVFGLELPEYLRCSIFNDAVSDQEECVGMA

>AgFz-5

KCEPLRLGYCRSVGYNVTTYPNFFGHGSLEEVEADLISFRELVDAECFRQAFDFICRLLQPPCEYRSIEEPTAGTVCRQYCQSFWAGCGERLPERLRRYLDCERFPESTGVQSCHS

>AgFz-6

GKCEPIKHRQCLHSTLPYSSISLDLTDSYSQEEMHTKLHQYNALKSVPKCWAVIQPFLCAVFTPKCEKINGQDMVYLPTLEMCKLTLEPCRILHNTSFFPEFLKCNETLYPSKCNNDVREEMKFNASGQCQK

>AmFz-1

NGRCEEITIPMCRGIGYNLTAMPNELNHDNQEEAGLEVHQFWPLVEIKCSPDLKFFLCSMYTPICLPEYTKPLPACRSVCERARAGCAPLMQQYGFSWPERMACERLPAHGDPENLCMEQDNH

>AmFz-2

HHGRCEPITINLCMNIPYNETIMPNLMNHQKQEDAGQEVHQFAPLVKMKCSPDLRFFLCTVYAPVCTIIDRAIPPCRSLCESARAGCERLMNSFGFAWPDNLDCSKLPENGGPELCVGHNET

>AmFz-3

PGVCLPIIVAFCKYHKIPYNFTIFPNYMGNFGQRDAQHELELYSAVIDVKCYELAALFLCSVFVPKCGSRGHVVRPCRSLCYHTKRRCGFFLDVFGLTLPEYLECDLFPENSNSDECVG

>AmFz-4

AKCVEIQKNTCMGTRLPYTTTTLELIPEHITQDIIEEKLHVLQTLRHVPKCWAVVQPLLCSIFMPKCINDTVDLPSQEMCKMVSGPCRIVFNHTIWPSFVKCENTDLFP

>AmFz-5

RRCTSLNLSYCKHLPYNVTSYPNILGHRSLADVQEDVIAFRELVDAECYRLAYDFVCQILQPTCISSQPEDLLQLPCRSFCREFWNGCGNRLPDKFKPLLDCSNFPEYVDQGGCRAKPGCVQ

>BmFz-1

QPRCQDITIPMCRGIGYNLTSFPNALDHDTQEEAGLEVHQYWPLVEIKCSSDLKFFLCSVYTPICIEDYAKPLPACRSVCERARAGCAPLMQQYGFPWPERMACDAAECECACRPPLLAA

>BmFz-2

ASVRTCEPIKVAMCKNIGYNQTGMPNLARHTLQADADITLQTFSPLVQYGCSSQLHLFLCSVYVPMCTDKVALPIGPCRGLCDSVHARCFPVLHGFGFSWPPELDCTLFPAENNHEHMCMEGP

>BmFz-3

QHGRCEPITIQFCQKLRYNQTIFPNILNQARQEDAAANMLLFTTLIKLNCSPDLRFFLCSVYAPVCTILDSAIPPCRHLCEAAKQSCDVVIRKFDFPWPPELECSEFPEVSDNNICV

>BmFz-4

GVCLPVIVSFCHQHRISYNFTVFPNYIGHFGQRDAQQDLEIYDAVVDVRCYELTALFLCSLFVPKCGPLGHMVRPCRSLCQETMRRCGFFLEVFGLSMPDYLQCEIFPESTDTDVCLGNREV

>BmFz-5

GKCERITLPLCQELGYNWTSMPNLMGHKDQKEAEQAMSPFSDILGSGCSLQARFLLCSAFAPLCSEEVSGSVSACRALCETVADDCKEQIKILSPTVMLDCSAFPLRANRKLCMRAPN

>CfFz-1

GRCEPITINLCMNIAYNETIMPNLMNHQKQEDAGQEVHQYVPLVKMKCSPDLRFFLCTVYAPVCTIIEKAIPPCRSLCESARSGCEGLMNSFGFAWPEALDCSRMPENDGTELCVAT

>CfFz-2

GRCEEITIPMCRNIGYNLTAMPNELNHDTQEEAGLEVHQFWPLVEIKCSPDLKFFLCSMYTPICLPEYTKPLPACRSVCERARAGCAPLMQQYGFSWPERMACERLPNHGEDPENLCMEQD

>CfFz-3

GACEPIRIEMCRGLGYNVTVMPNLVGHEIQGDADFTLQTFSPLIQYGCSAQLHLFLCSVYAPMCTEKVPAPIGPCRGLCEQVRARCFPVLQGFGFPWPAALNCSKFPPENNHQHMCMEGP

>CfFz-4

GPKCEKLSVSFCRGLRYNLTAMPNFMGHEDQRQAERGLTTFMPLVHYNCSRHLRLFLCAVFAPVCSEHVAMQIPACKSLCLSVRRDCEPALTSLTLPWPHMLDCDRFLDRGRNTLCVQP

>CfFz-5

GVCLPIIVNFCQQHNVPYNYTVFPNYMGNFGQREAQHELELYDAVVDVRCYELAALFLCSVFVPKCGYGGRVVFPCRSLCHQTKRRCGFFLKVFGLSLPDYLECELFPESSNPDECIGY

>CfFz-6

KCNKLDLPYCKHLSYNISSYPNVLGHRSLADVEEDVIAFRELVDAECYPLAYDFICQVLQPACQLSHPEDLLQLPCRSFCREFWNGCGNRLSEKIKRALDCSNFPEYVGPGSCR

>CfFz-7

SAKCVPLHKGTCMGTKLPYSFTSLDLIPERVTQDIIEEKLYSLQALKHVPKCWAVVQPFLCSIFMPKCVNNTVELPSQEMCRMVSGPCRMLINHTIWPSFAKCDNTKLFPRLC

>DpFz-1

QPTCLDIPRNLSLCHGIRYSKMRLPNLLDHDSMAEVIQQAASWVPLLNVRCHADTQLFLCSLFSPVCLDRPIYPCRGLCERVRQGCEGRMKTYGYPWPDMLRCDKFPLDNDMCIGPL

>DpFz-2

HGRCEAITIPLCKDILYNETIFPNLLNHQKQEDAGLEVHQFYPLVKVKCSPDLQIFLCSVYAPVCTVLDKPIPPCRSLCLSARSGCEGLMNKFGFQWPESLECSRYPEGGAAGELCVGENN

>DpFz-3

DARCEEITIPMCRGIGYNWTSMPNSLHHETQEEAGLEVHQFWPLVEIQCSPDMRFFLCSIYAPICIQDYPTSIPACKSVCLRAESGCAPLMRKYGFAWPDRMQCDKFPNFGDPQNLCMDARN

>DpFz-4

AKCQPIKNNICLTTKLPYSQTSLELVTDSHTQDDVQDKLMFWQNLKSVPKCWAVIQPFLCALYMPRCENGTVELPSQEMCKVIRNPCRIVELEHRSGWPDFMRCEDADKFPSGCKN

>DpFz-5

VEPECIRRQLPFCRGVLPYSETILPNWVGDNTEAERNFSVPYFEIIAESECHPRVQQYACAVLEPPCRGSGISLPPCRQFCRAIAEDCSSYVLSALTLASVLDCDQFPQSNDPDVCLNL

>DpFz-6

SQCVPRNLAFCNNTLNYTETVYPNLSGDLSEDDFVRSWAFLQTVIDSYCHPLIEQFVCQAAQPECRPNDKMPIGPCRQLCLEVAKACDPHIWSILEKEKMMFNCEQYAMVDDLNLCFSS

>DmFz-1

NRCEPITISICKNIPYNMTIMPNLIGHTKQEEAGLEVHQFAPLVKIGCSDDLQLFLCSLYVPVCTILERPIPPCRSLCESARVCEKLMKTYNFNWPENLECSKFPVHGGEDLCVAE

>DmFz-2

FRQCETIRIEMCRKIGYNETSMPNLVGNEMQTDVEYTLQTFAPLIEYDCSSQLKLFLCAAYVPMCTPKAPVHAIGPCRSLCESVRIRCHPVLQGFGFPWPPALDCDKFPRENNHETMCMEG (2fred)

>DmFz-3

NLRCEEITIPMCRGIGYNMTSFPNEMNHETQDEAGLEVHQFWPLVEIKCSPDLKFFLCSMYTPICLEDYHKPLPVCRSVCERARSGCAPIMQQYSFEWPERMACEHLPLHGDPDNLCMEQPSYTEA

>DmFz-4

GLQCQPIAVSACQGLGYNMIALPNLAGHTNQLEAELQIAKLVPLIESGCSRRARFLLCSSLFPLCTPDVPRPVAACKLLCETVRGECMENAPPELMELWPSFLNCDGLPQPEKHELCMQIPQEV

>DmFz-5

RRCSPLELSYCRQVGYNITTYPNLLGHASYEQLAEDVIVFRELVDGECHREAYDFVCRLLQPPCDTHGSDLQPTPGQICREYCESFMAGCGGRLPQRFRQFFDCERFPESTGTQSCHQKPHCVSD

>DmFz-6

PGTCLPIIVRFCQGPQIPYNYTVFPNYIGHFGQLETQTDLDSYEALVDVRCYELVSLFLCTLFVPKCGQSGATVPPCKTLCTETMRRCGFFFDVFGLSLPEYLNCKLFKDFPSSEDCVGL

>DmFz-7

NSGCQSTMLPMCQGVLDYDLTFNREGAAPRDAVSMAAYDSLIRANCSVRAAEFICGALEPECRPLHIGQLPPCRRICKAILEACSIPIYNSDVLGELFDCNLYPDAHESHKCEDPTRRRDYCY

>IsFz-1

PTCLEIPANLTLCRDIGYSKMRLPNLLEHDSMAEVQQQARSWVQLANRRCHPDTQLFLCSLFSPVCLERPIFPCRSLCEAVRSGCEGTMLRYGYPWPDMVRCDKFPVDNDMCISVQ

>IsFz-2

RMCEPIRIEMCKDIGYNVTGMPNLVGHELQQDAQLQLQTFKPLVQYGCASQLKFFLCSVYVPMCTEKVAQPIGPCRPLCESVRTRCQPVLQEFGFPWPAALNCSKFPPQNNHRHMCMDGPA

>IsFz-3

GRCEPISIPLCKDIQYNETIMPNLLNHQKQEDAGMEVHQFFPLVKVKCSPDLQFFLCSMYAPVCTILEYPIPPCRSLCMSARSGCESLMNKFGFKWPESLECDKFPEVGSEKLCVGEN

>IsFz-4

RCEDITIPMCKGIGYNQTSMPNQFNQDTQDEAGMEVHQFWPLVEIQCSDDLKFFLCSLYTPICMEEYAGSVPACRSVCERARAGCAPIMRQYGFAWPERMNCDALPQYGDQEQLCMDAKE

>IsFz-5

CEPIINATCFGVSLPYGHTTVELVNDSTSQLEIQERLDLWQGLQQIPRCWAVVQPLLCAVYRPKCADGRVTLPSQEMCRLVRGFCKIVALGSEWPSFLRCQHDIFASGCKN

>NvFz-1

HGKCETITISLCKDMPYNETIMPNLLNHQKQEDAGPEVHQFSPLVKLKCSPDLKFFLCAMYAPVCTILEKALPPCRSLCESARNGCERLMNNFGFYWPESMECSKFPENRDGVLCVGKN

>NvFz-2

SRCEEITIPMCKGIGYNLTTMPNELNHDTQDEAGLEVHQFWPLVEIKCSLDLKFFLCSMYTPICLPEYSKPLPACRSVCERARMGCAPLMHQYGFSWPERMACERLPNQGDPENLCMEQD

>NvFz-3

AKCERLNVSFCRGLRYNLTAMPNFMGHEDQLQAERELAKLMPLVHYNCSRHLRFFLCSVFAPVCSEHVAMQIPACKPLCLSVRRDCENTLKDLTLPWPHMLDCDRFPDSGNTLCVQP

>NvFz-4

ICLPVIVNFCQYHKIPYNYTVFPNYMMHFSQREAQHDLELYDAVIDVRCYELAGLFLCSVFVPKCGHKMGRAVGPCRSLCFETVRRCGFFLDVFGLTFPDYLQCDIFTESTDPNVCIGHQE

>NvFz-5

RRCSPIEFSYCKHLPYNVTSYPNFFGHNDAREVNDDIIAFRELVDAECYKQAYDFICQVLQPACLEGDEEDVLSPPCRGFCKEFWSGCGSRLPERLKAALDCAKFPEYADEGSCRS

>NvFz-6

KCVKMINTTCLGATLPYDTTSLDLMPKYTTQEMIMEKLHILRGLEHIPKCWAVVQPFLCSLFLPKCVNDTVDLPSQEMCKVVSGPCKILLNHTIWPNFIKCDNEELFSRSC

>PhFz-1

RTCEPIRVELCRGLGYNMTGMPNLVGHDLQGDADFTLQTFSPLIQYGCSGQLHFFLCSVYVPMCTEKVATPIGPCRGLCESVRSRCFPVLQGFGFPWPAALDCSKFPQENNHEHMCMEGP

>PhFz-2

MCRGIGYNLTSMPNELNHESQEEAGLEVHQFWPLVEIGCSPDLKFFLCSMYTPICIEDYHKPLPACRSVCERAREGCTPLMLQYGFPWPERMACERFPIHDSDPENLCMEQ

>PhFz-3

GKCEQIKIPFCMGLQYNETIMPNLLGHTKQEDAGLDVHQYFPLLKVKCSPTVQLFLCSLYFPVCTILPNPLPPCRSLCISARKGCEDLMNQFGFFWPDKFECDNFPEVNQSQS

>PhFz-4

MCQNLGYNLTTLPNFMDHKDQSQAERALGTFMPLVHYNCSKHLRQFLCAVLTPMCSEQIKGAIPPCRGFCERIQGDCQPVIDQFEFPWPSLLNCSRFPVYNGYCISG

>PhFz-5

QCSPLSLEYCSKMSYNTTSYPNIVGHSNYFQVLDDVISFREIVDAECYRLAYEFVCLILQPPCEKRGGAKEEEEEDFLVMPCRSYCNEFIKNCGSRISSRFMNFLDCTKFSEFSDTGICV

>PhFz-6

GFCFKLIVEFCANHKVPYNFTTFPNHVVNMNQDQAEQELDLYDALVDVRCYELSALFLCSVFVPKCGRQGELLYPCKSLCEETKRRCGFFLEVFGLPALPEYLMQCNLLPDSDDPDACVGH

>RpFz-1

GKCEPITVPICKKIQYNVTIMPNLLNHNTQEDAALEVHQFYPLVKIDCNSVLLFFLCSVYVPVCTILDRPIPPCRSLCISARSGCEEVLHKFGFLWPENLECDRFPDPPDICV

>RpFz-2

GPRCEEITIPMCRGIGYNLTSMPNELNHDSQEEAGLEVHQFWPLVEIRCSPDLKFFLCSMYAPICIEDYHRPLPACRSVCERARSGCAPLMQQYGFQWPDRMACEKLPVHGDPDNLCMEM

>RpFz-3

LCLPVIVPFCIQHKVPYNFTMFPNYIGHFNQREASQELEVYDAVVDVRCYELAALFLCSVFVPKCGPEGQLVRPCKSLCTETKRRCGFFLEVFGLTLPNYLDCDLFPESEDQNVCIGH

>RpFz-4

RTCAPMELNYCNRYVANYTSYPNIVGHYNMEQVMDNVIIFRELVDSECYRLAQEFVCQLLQPPCSQNLILPCRSFCNEFWEGCGSRLPANLQDYFNCSQFPEYSAEGPPCLP

>RpFz-5

NCHSPQLPMCRGVIPWDLTSIPSLPGISTMESLREAMPYFELILDSGCSQRARQFLCTLLEPECQPLGSSVTPPCRNTCKVVAEECSDFIINILDLSQIFKCDNYPDSEDECIN

>RpFz-6

AKCEPLNYTTCLGVKLPYTWTSLDLVDGLVSQEQGQEQLKEWRRLVHIPKCWAVIQPFLCALYMPKCDDHGVYLPSQEMCKIIMGPCRILVSYEPWPAVFRCDNQTRYPPMCKNDVRELK

>SmFz-1

VQPTCVDIPRNMTLCHDIGYTKMRLPNLLDHDTMAEVSQQAGSWVPLLNIECHPDTQLFLCSLFSPVCLDRPIYPCRSLCDKVRAGCEGRMRVYGYPWPDFLRCEKFPLDNDMCITA

>SmFz-2

DPSRCEEITIPMCRGIGYNATFMPNQFNHDTQDEAGLEVHQFWPLVEIQCSPDLKFFLCSMYAPICIEDYLQPLPACRSVCDRAKAGCAPLMRLYGFAWPERMNCDRLPEYGDPSQLCMDSK

>SmFz-3

GRCEPITIPLCKDIQYNETIMPNLLNHQKQEDAGLEVHQFFPLVKVQCSPDLQFFLCSMYAPVCTILDQAIPPCRSLCMSARVGCEGLMNKFGFQWPESLDCSKFPEAGSHDICVGE

>SmFz-4

RCERITIPMCQDMPYNLTRMPNYMGHAEQSEAAIEVHEFIPLVEIGCSKHLKFFLCSLYAPMCSEQVDLAIPSCQSICEEVKTHCLPILQQFNFNWPRMLNCSRLPVPEMNELCMEFP

>SmFz-5

SCDPIRIEMCRGLGYNVTSMPNLVGHELQQDAEMQLQTFSPLVQYGCSSQLRLFLCSVYVPMCTDKVPMPIGPCRSLCEVVRSRCQPVLQEFGFPWPSALNCSQFPPENNQHHMCMVGPG

>SmFz-6

CTKCVYNTVPMCQNMGYNLTLFPNTFKHGTQEDVSEALLFYSALMKSNCSQSLAFFLCSLYVPVCRPDYSYSILPCNSLCRKILNHCDAAMKELQLDWPSRIDCYYFPVVDEYNICSEE

>SmFz-7

ERKQCVPVGARFCNDVFYNHTSFPNIFGHKTRYEVDDFVDQHFSQLITSGCYNHLKHFLCSLLQPSCSQHQVIFPCQEFCHAFLTQCHSVFPFILSRFLHCNNYPSIKNTAEKCLAEP

>SmFz-8

GSQCRHRTLSFCDDVSSYNSTYLPNLAGATTEFEKWQLVSYYNSIVDWECNLWLKEYLCYILEPMCVDEVAVPPCNSLCKAAKKGCEKFITGSPTLEAVFRCDVFPSATAQILCAGLG

>SmFz-9

QAQCENLHSNLCFGIALPYKQTSLSLLGSSMNQSEVQRQMQLWAGLSSVPRCWSVIRPLLCAVYMPRCENGSIDLPSYDLCRITRKRCRIVEVIENWPEFLKCDKPHFKHQCKNE

>TuFz-1

PKCEEITVPMCRGIGYNMTSMPNQFHHEKQDEAGMEAHQFRLWKFLTTCSVCERAHAGCAVIMLTYGFPWPESMDCNNFPVYPGAPEQLCMDQ

>TuFz-2

RSKCEEITVPMCRGIGYNMTSMPNQFHHEKQDEAGMEAHQFWPLVEISCSDDLRFFLCSMYTPICMEDYTERLPVCRSVCERAQAGCAAIMLQYGFPWPESMDCNNFPVFGSQEQLCMDH

>TuFz-3

VRTCEQIKFESCKKIGYNVTGFPNSAGHETQDEAGQTFQTFDPLIKFRCSSQLKFFLCSVYFPMCTEKIVQTIGPCRPFCETVRDRCLPILNDFGFAWPSYMNCSLFPAANDNQTMCMVG

>TuFz-4

AECIRRSLPMCNGQVPYNSTVYPNYIGDANEIEASRSLPYYNYIAKSKCNRRIKQLLCTFLEPPCVEGRPIPPCKKFCRIALEGCAEYVPATLELSAAFDCRRYPDSTDPSVCVN

>TuFz-5

RPKCEEITVPMCRGIGYNMTSMPNQFHHEKQDEAGMEAHQFWPLVEISCSDDLKLFLCSMYTPICMEDYPGRLPACRSVCERAHAGCAAIMLTYGFPWPESMDCNNFPVYPGTPEQLCMDQK

>TuFz-6

KKRCETVSVAFCREIGYNETIFPNLLNHNSQQNAGLALHRITPLIKVNCSPDLKLLLCAVFFPPCTILEAPIPPCRSICLSSKNGCEDVIHRFGQEWPSYLDCDKFPDVEPCVS

>TuFz-7

RHDCVPMKVAYCIKHNVRYSHITLPNKNDDDTSQESINIQLAEYDPILSVKCYSLLPLFLCSLSTSFCNSTSQPIKPCRSFCKEALRRCDFFLSVFSLEWPSEINCDQYPDDPDPDVCVGYR

>TcFz-1

HDKCKPITVPFCIDVPYNSTIFPNLVGHNTQEDAGYEVHQYFPLIKINCSADLHLFLCSVFVPVCTILEKPVPPCRSLCLSAKSGCEGIMRKFGYNWPENLDCNQYPENSNLCVEKHNI

>TcFz-2

VRTCEPIRVDMCTNLGYNMTEMPNLGGNDIQQEADYTLKSFSPLIQYGCSSQLKLFLCSVYVPMCTEKVANPIGPCRGLCESVRAKCYPVLKGFGFSWPDALNCSRFPVENNHEHLCMEGPKD

>TcFz-3

PRKCFPLQLKYCTKLPYNVTTYPNLMGHRNVIEVKDNVITFRELVDAECYRHAYDFICQILQPSCVSGEGQDEMILPCRSFCREFMAGCGARLSEKMKESLDCSQFPEYSCAAKPGCVE

>TcFz-4

PGICLPVIVNFCLQHKVPYNYTVFPNYMGQFGQRDAQQELELYDAVVDVRCYELSALFLCSLFVPKCGPHGEVVRPCRNLCNETKRRCGFFLDVFGLTLPEYLDCSLFPEKPDRGHCIGY

>TcFz-5

PAKCQLLNYTTCMGMKLPYFSTTLELTDLTTQEKVQEKLHHYKYLRFIPKCWAVIQPFLCALYMPKCENGKVDLPSREMCQLTLKPCKMFYNSSIFPKFLNCDDERIFSSNCKND

>TcFz-6

ERCEDITIPMCMGIGYNQTRMPNELNHETQEEAGLEVHQFWPLVEIKCSPDLKFFLCSMYAPICLPGYKKPLPPCRGLCKRAREGCEPIMTQYGFKWPERMDCEQFPVYGASPDQLCMD
