# Supplementary material for: Insights into the molecular evolution of peptidase inhibitors in arthropods
Source: PLoS One. 2017 Nov 6;12(11):e0187643. doi: 10.1371/journal.pone.0187643 (PMC5673224; doi:10.1371/journal.pone.0187643)
Supplement: S2 Fig — (DOCX) [file pone.0187643.s002.docx]

**S2 Fig.** Comparison of the amino acid sequences of the domains belonging to the different peptidase inhibitor families. The alignments were generated using the MUSCLE program. Dm, *Drosophila melanogaster*; Ag, *Anopheles gambiae*; Bm, *Bombyx mori*; Tc, *Tribolium castaneum*; Cf, *Camponotus floridanus*; Am, *Apis mellifera*; Nv, *Nasonia vitripennis*; Rp, *Rhodnius prolixus*; Ap, *Acyrthosiphon pisum*; Ph, *Pediculus humanus*; Dp, *Daphnia pulex*; Is, *Ixodes scapularis*; Tu, *Tetranychus urticae*; Sm, *Strigamia maritima*.

**A. I1 Kazal.**

DmKaz-11 -------C-D---YS-------CP---EK-------DPS------VCAT-NGQ-CILKFE

DmKaz-7b -----KEC-F---KP-------CS--MIY-------QP-------VCIT-NGK-YRAELA

SmKaz-5b -------C-H---FN-------CR--AVKFHLQ---GP-------VCAT-NNV----TYA

DmKaz-7a -----TAC-P---TF-------CP--SIY-------KP-------VCGT-DGQ-NFKEFA

DmKaz-5a -------C-E---FE-------CS--SRY-------QP-------VCGISSKSGERKTFR

ApKaz-3c -------C-N---MS-------CPKPVPSEKLKQTGGK-------VCGS-NDK----TYH

DmKaz-9 -------C-T---KE-------CP--DIY-------DP-------VCAQ-IFQEEYLTFS

DpKaz-6b -------C-I---KEALKD---CI--SIQ-------II-------ICGT-DGI----VYG

SmKaz-6a -------C-A---EAAAT----CT--GPR----PM-KP-------TCGS-DGM----LYY

TuKaz-4 -------C-P---ITKK-----CP--VHK-------HK-------ICGS-DGK----LYD

SmKaz-3a -------C-P---TQ-------CS--LDK----I--DP-------VCGS-DGK----LYY

SmKaz-3c -------C-P---DQ-------CP--FGL----I--DP-------VCGS-NGK----LYY

DmKaz-6 --AQKSRC-P------------CP--KIS-------EP-------VCGS-DNI----TYP

SmKaz-1g -------C-K---EY-------CS--PIP-------DP-------VCGS-DGK----AYS

DmKaz-1i -------C-A---RI-------CP--REF-------EP-------VCGS-DNK----TYL

ApKaz-1i ------GC-Q---RV-------CP--TLY-------DP-------ICGT-DLK----TYS

DpKaz-1i -------C-N---RI-------CP--SEY-------DP-------VCGT-DRK----TYS

DmKaz-1g -------C-G---DA-------CTRADLE------QQP-------VCGS-DGN----TFA

ApKaz-1h -------C-H---QQ-------CD---KT----K--EF-------VCGS-DNK----LYR

DmKaz-1h -------C-E---SD-------CD---AQ-------PPSF-----VCGS-DNN----LYK

DpKaz-1h ------SC-N---SQ-------CD--RKV-------QS-------VCGS-DGK----IYR

DpKaz-1d ------HC-K---EI-------CW--KAS-------KA-------VCGS-DGH----IYA

SmKaz-1f -------C-D---TN-------CP---IE----AGLKP-------VCGS-DAT----LYM

ApKaz-1d -------C-R---ES-------CW--RVS-------KP-------TCGS-DGN----IYS

DmKaz-1d ------MC-R---ES-------CW--RVA-------RP-------TCGS-DGR----LYA

SmKaz-1a -------C-P---RQ-------CR--EIG------STP-------VCGS-DGV----IYA

DpKaz-1a -------C-P---RF-------CP--KEN-------KP-------VCGS-DGV----IYT

DmKaz-1e -------C-P---TE-------CP--KSDTDSSS--QY-------VCGS-DGN----IYS

DpKaz-1e -------C-P---AE-------CD---SA-------EPQE-----VCGS-DGN----IYG

ApKaz-1e -------C-P---VN-------CD--KEK----D--RL-------TCGS-DGN----VYR

DpKaz-1g -------C-P---ES-------CPDQRES------DQP-------VCGS-DGN----VYR

DmKaz-1a -------C-P---RS-------CP--PSITVG-A--EP-------VCGS-DGL----IYA

SmKaz-1e -------C-P---KE-------CP------IPSPGDGP-------VCGS-DGN----VYP

DpKaz-1c ------PC-P---QQ-------CSSNDKD-------GP-------ICAS-NGN----VYK

ApKaz-1c -------C-P---VN-------CDQAPMD-------GP-------ICGS-DGN----VYK

DmKaz-1c -----ESC-P---VD-------CNSAPKD-------GP-------VCSS-DGN----VYN

ApKaz-1a -------C-P---RI-------CPQGSSE-------EP-------VCGS-DGI----IYP

ApKaz-1g ------KC-P---QS-------CE---NERE-----EP-------VCAS-DGN----VYR

SmKaz-1c -------C-P---NN-------CPPVRGFLE-----RP-------VCAS-NGN----VYR

TuKaz-6d -------C-N---FN-------CD--SEP----E--ES-------VCAD-DGL----TYA

TuKaz-1e -------C-P---TS-------CS-DPVY-------EP-------ICAN-DGN----TYP

TuKaz-5b ------IC-P---TD-------CP--TSF-------EP-------VCSS-EGT----TYT

TuKaz-8g -------C-R---ED-------CP--EEN-------ES-------VCAS-DGV----TYR

TuKaz-7f -------C-P---SE-------CP--QTY-------EP-------VCST-DGL----TYI

TuKaz-2d -------C-P---KD-------CP--SIY-------EP-------VCST-DGY----TYA

SmKaz-2w -------C-A---GVGKNGVQVCS--EEY-------SP-------YCGS-DHA----TYK

SmKaz-2r -------C-D---EQILKIE--CT--QDE-------FP-------LCGT-NGI----TYG

SmKaz-2g -------C-P---KSLANGI--CT--LEY-------SP-------VCGT-DGI----VYS

SmKaz-3b -------C-KADCEKGF-----CT--MEY-------SP-------RCGS-NGN----KYG

SmKaz-3d -------C-K---AD-------CD--KSICTLEY--SP-------RCGA-NGS----KYG

SmKaz-10 -------C-D---EN-------CS--KEI-------NR-------VCGT-DGV----TYN

SmKaz-4a -------C-Q---TN-------CP--SVF-------SP-------VCGT-DGR----MYS

SmKaz-2m -------C-PEISDYYK-----CN--DAY-------HP-------VCAS-NAI----TYG

SmKaz-2s -------C-PISPINA------CP--LNY-------AP-------VCAS-DDN----TYP

SmKaz-2i -------C-S---ENNQHII--CT--EEY-------NP-------VCAS-NGQ----TYG

SmKaz-2k -------C-PGKNENGI-----CT--TEY-------SP-------VCAS-NDQ----TYS

SmKaz-2b -------C-P---ESSKHGI--CT--LEY-------AP-------VCAS-NDV----TYP

SmKaz-2c -------C-P---ETSKHGI--CT--LEY-------AP-------VCAS-NGE----TYA

DpKaz-7 -------C-L---AT-------CP--DHF-------VP-------VCGS-NNQ----SYD

ApKaz-4a -------C-N------------CA--PHH-------VP-------VCGS-NGN----TYP

SmKaz-2d -------CLP---RDNTGA---CT--REY-------MP-------ICGS-NGK----TYP

ApKaz-6 -------C-K---SE-------CP--NDY-------NP-------ICGS-DGAKVNLSFG

SmKaz-2v -------C-T---EMSKFGA--CP--YTY-------FP-------LCAT-DGK----SYP

SmKaz-2e -------C-P---EMSKKGA--CP--RNY-------RP-------VCGS-DGK----TYG

SmKaz-2o -------C-P---KLSKRGI--CP--RGG-------NP-------VCSS-DDT----TFE

SmKaz-2j -------CLH---CSEMSKDGICS--REF-------NP-------FCGS-DGR----TYG

SmKaz-2f -------CRPEWKTGI------CT--REY-------NP-------ICGS-DGV----TYG

TuKaz-3 -------C-L---DS-------CP--STY-------EP-------VCGS-NGE----TFV

SmKaz-2q -------CLK---EDKLEM---CT--DEY-------DP-------QCGS-DGT----TYP

SmKaz-6b --CENINC-L---QL-------CT------REY---DP-------VCGS-DGE----IFG

DmKaz-2a -------C-R---IE-------CP--WDNLD---VDSSGYDERQAVCGV-DGK----TYR

ApKaz-4c -------C-L---VG-------CN---ST-------GR-------VCGV-DGN----TYP

TuKaz-8h -------C-L---FN-------CD--ETNSS-----EI-------ICAN-DTY----FYP

DmKaz-12 ----LEVC-P---DN-------CQ--DQY-------NP-------VCGKYKDT--RRNFR

DmKaz-5c -------C-D---KT-------CP--TVY-------QP-------ICATRNGI--NHTIV

ApKaz-9 -------C-KGRVER-------CE--R---KKAATRRP-------VCGT-DNI----SYP

ApKaz-4b -------C-VSEKTS-------CE--DA------PKGV-------VCDT-DGG----EHS

SmKaz-2n -------CLESDMKEE------CS--KDY-------NP-------VCAS-NGI----TYA

ApKaz-2g -------C-I---FN-------CS-ATDS-------SP-------VCAS-DVR----TYN

SmKaz-4b --CTVKRC-P---DV-------CY--QLF-------DP-------VCDT-NGK----QYG

SmKaz-4c -------C-S---SI-------CI--ALY-------KP-------VCDT-NGN----RYE

TuKaz-7h -------C-A---MR-------CP--EVTLGPGHLIRRTSQV---ICAS-DGN----TYL

TuKaz-6a -------C-----KT-------CS--SHI-------NPVL-----LCAT-DNT----TYY

SmKaz-2t -------CLP---FETMGL---CN--KQY-------SP-------VCGT-NGV----TYG

ApKaz-1f -------CNK---GQ-------CP--DDA-------DP-------ICGN-DAQ----NYK

SmKaz-2h -------CTA---SRP------CS--FGK-------TP-------VCAS-DGK----TYS

SmKaz-2l -------CASCPVQSHSRDVSHCA-DQED-------NP-------ICGS-TGK----TYN

TuKaz-1b -------CLQ------------CE--SIY-------KP-------ICGS-NRI----TYD

SmKaz-2a -------C-TFGQDI-------CP--LIY-------AP-------VCGS-DGN----TYS

DmKaz-1f -------C-K---QLPP-----CKDFNSLFGSIFSSKRNDK----LCGT-DAK----TYN

ApKaz-2c -------C-P---EPTL-----CE--TSV-------RP-------VCGT-DGY----TYE

SmKaz-2p -------C-S---SS----V--CP--SIW-------NP-------LCGS-DGK----TYA

SmKaz-2x -------C-S---SSDLNGA--CP--DNY-------MP-------VCGS-DGV----TYA

ApKaz-2a -------C-Q---GD----D--SG--DG------AGGP-------VCGN-DWR----DYP

TuKaz-1f -------C-K---FN-------CS--TMIRQSKGKTET-------VCGS-DGR----YYE

TuKaz-7g -------C-S---YQ-------CP--TTS-------GPDDS----VCGS-DGR----LYE

TuKaz-2e -------C-S---YS-------CPVRDES----RD-EF-------VCGS-DGR----LYE

TuKaz-5c -------C-S---YS-------CPSQESD------EDP-------VCGS-DGR----LYE

DmKaz-10 ---TRPLC-P------------CP--RIY-------FP-------VCGS-DHV----TYT

DmKaz-2b -------C-R---YK-------CP---RK-Q----QRPVHK----ICGY-NNQ----TYN

ApKaz-5 ----QYRC-A------------CK---ET-------DP-------VCGS-DNR----TYS

DpKaz-2c -------CLP---YEF------CV-PDPH-------PP-------VCGT-DGK----TYR

ApKaz-2f -------C-P---ET-------CV---DT-K---SKEP-------VCGS-DLI----TYN

DmKaz-3 -------C-AAKQGE-------CD--DNE-------GP-------VCGT-DGQ----TYP

SmKaz-2u -------C-DMNIKD-------CS--KES-------HP-------VCAT-DGT----TVP

DpKaz-1f -------C-Q---DD-------LE------------NPDY-----VCGN-DGR----TYP

DpKaz-1b -------C-G---NK-------CS---LD-R-----DL-------VCGS-DGR----TYL

ApKaz-1b -------C-E---HR-------CG--KEQ-------DA-------VCGT-DGR----TYL

DmKaz-1b ------DC-K---HR-------CS--TEK-------DP-------VCGT-DGR----TYL

SmKaz-1b -------C-T---AF-------CY--GHH-------DP-------VCGS-DAV----TYD

DpKaz-5b -------C-G---DT-------CP--SDF-------QP-------VCGS-DGR----SYS

TuKaz-8b -------C-P---EK-------CL---IY-GDTKSSGS-------VCGS-NGM----NYP

ApKaz-2b -------C-S---EF-------CG--EDF-------IP-------VCGS-DGR----TYT

SmKaz-9 -------C-P---DK-------CP---SY-GDHRGSLP-------VCAT-DGK----DYP

TuKaz-7a -------C-P---EK-------CY---TY-GDSVGSRP-------VCGS-DGR----DYP

DpKaz-5a -------C-S------------SY------GDSVGSRP-------ICGV-DGK----DYA

ApKaz-7 -------CNP------------CP---VM-------KPTF-----LCGS-DNR----TYS

DpKaz-3a ----DPSC-N------------CK--KNY-------AP-------VCGT-DGK----TYS

DmKaz-8 -----PIC-P------------CP--RNY-------EP-------VCGS-NLV----TYP

DmKaz-4 --VFQYSC-P------------CP--RNY-------DP-------VCGS-DSV----TYS

TuKaz-6b -------C-P---Y--------CE---DS-------GPS------VCGS-DGI----TYS

TuKaz-6e -------C-D---IN-------CQ---NN-S--TDVSNSSMDYSIVCGS-DSN----TYQ

TuKaz-8i -------C-Q---YN-------CD---SS-D----LLP-------VCGS-DNN----TYG

SmKaz-1d CRPRLEKC-N---KGT------CG---AG-F-----NP-------ICGS-DGR----TYN

TuKaz-5a -------C-Q---ED-------CPLDGGI--------S-------VCGT-DGL----TYQ

TuKaz-2f -------C-N---ID-------CS---PE-E--KVSDK-------VCSS-DGT----IFP

DpKaz-3b -------C-V------------CT--LQY-------EP-------VCAT-DGK----TYG

TuKaz-1a -------C-T---HQ-------CN--PNV-------HHY------VCGN-DNV----TYP

TuKaz-8d -------C-P---SE-------CP--PIL-------KP-------ICGN-DGV----TYE

DpKaz-4d -------C-P---MD-------CP---VA-S--SAEQT-------VCGS-DGV----SYG

SmKaz-5a -------C-S---RA-------CPDSKAK-------KY-------VCGV-DGV----SYT

ApKaz-3b -------C-V---TV-------CP---PG-M--SSSGQ-------VCGS-DGR----TYQ

TuKaz-2a -------CDT---FQ-------CD-STPF-------DP-------ICAN-DNK----TYS

DpKaz-4c -------C-P---KA-------CAKMAI--------TE-------VCGT-DGI----TYR

TuKaz-5d -------C-D---MK-------CQQDVTRKRLAGSGSER------VCAS-DGT----TYP

ApKaz-2h -------C-R---AT-------CA------EDAIRTTL-------VCGS-DGQ----TYS

SmKaz-8 -------C-Q---ET-------CS--AVF-------AP-------VCGS-DDV----TYS

TuKaz-8a -------C-L---ST-------CT-VDVF-------AP-------VCGS-DNI----TYS

ApKaz-3a -------C-S---PN-------CK---QH-DGLKVKGP-------VCGT-DGV----SYK

ApKaz-8 -------C-V---RK-------CG--PAK-------RR-------VCGT-DGR----LYD

SmKaz-7 -----CVC-Q---RY-------CK---KH-K-----KM-------VCGS-DGN----LYP

TuKaz-7c -------C-P---PM-------CP--QIM-------NP-------VCGS-DGK----TYD

DpKaz-4a -------C-K---GP-------CP--PIH-------RP-------VCGS-DQL----TYS

TuKaz-7b -------C-R------------CNAVEEEIII-------------ICGS-DGK----TYT

DpKaz-3c ------EC-G------------CQ--FKL-------AP-------VCGT-DGK----TYD

DpKaz-2e -------C-N------------GP---GY----SF-NP-------ICGS-NGK----TYD

DpKaz-6a -------C-E---IG-------CL--EIY-------EP-------LCGT-DGK----TYP

DpKaz-2b -------C-Q---IF-------CT--LEY-------MP-------VCGT-DGK----TYP

DpKaz-2a -------C-N---VF-------CT--FEF-------NP-------ICGT-DGQ----TYS

DpKaz-2d -------C-R---MP-------CT--REY-------NP-------VCGT-DGR----TYA

TuKaz-6c -------C-P---KV-------CI--RDD-------RP-------VCGS-NGK----TYE

TuKaz-1c -------C-----RQ-------CP--DEN-------EP-------VCGS-NGV----SYA

TuKaz-1d -------C-P---QV-------CL--RLD-------AP-------ICGS-NGV----TYD

TuKaz-2c -------C-P---KN-------CS--QEK-------DP-------VCGS-DGL----NYL

TuKaz-7e -------C-I---KN-------CS--HIK-------MP-------ICGS-DGV----SYA

TuKaz-2b -------CPS------------CS--HHY-------EP-------VCAS-DGL----FYT

ApKaz-2e -------C-P---TFK------CA---NA-----S-GK-------VCGT-DGI----TYT

ApKaz-2d -------C-P----L-------CS---EH-----H-EP-------VCGT-DGN----TYS

DpKaz-4b -------C-P----L-------CG---GE------WDP-------VCGT-DGV----TYT

TuKaz-8c -------C-S---SL-------CN--DNF-------KP-------VCAS-DGK----TYA

TuKaz-8f -------C-S---ES-------CV--PVD-------EP-------VCGT-DGK----TYP

TuKaz-8e -------C-P---TS-------CT--EDY-------NP-------VCGT-DGI----SYV

TuKaz-7d -------C-----QS-------CT--EEY-------KP-------VCGS-DGI----SYS

* *

DmKaz-11 SRCAM-SAY--NC-----RNPQKMF---KPV-EDH--RCTQ------

DmKaz-7b NSCLL-ENF--NCALQVSGAQPAEL--FRLL-REE--KC--------

SmKaz-5b SWCHM-MQA--SC----SLR--TAL---ELR-SNA--KCEE------

DmKaz-7a STCNL-LSH--NC-----RRERNSVQAYAAT-DAA--WC--------

DmKaz-5a SRCEM-LRT--AC----ISR--SEW---MVH-RWG--VCP-------

ApKaz-3c SWCQM-FMD--AC----ATG--VVI---ETK-ASG--PCPDG-----

DmKaz-9 NECEM-RNY--IC----TNE--RPY---SFI-SVG--ECVE------

DpKaz-6b NPCQL-KAK-NKC----DGTSVGEA-------PAS--HCQQ------

SmKaz-6a NECHM-KDE--VY-----GIVPYPV---YPV-AWS--EC--------

TuKaz-4 SHCHL-RKI--SC----EKG--IQL---RPV-HPD--QCD-------

SmKaz-3a TQCHL-EKE--TC--------GQDV---SKV-SWS--DC--------

SmKaz-3c TECYL-EKA--TC--------GQGV---TKV-PWS--EC--------

DmKaz-6 HLCFL------IC-----KAWHENV---NFV-KKG--RC--------

SmKaz-1g NECFL-GLE--TC----RTR--GKV---RMD-STW--KCG-------

DmKaz-1i NDCFL-EIE--NC----RAN--QTV---NVN-YYG--ACG-------

ApKaz-1i NDCFL-EME--NC----RSR--SLV---SKQ-YHG--VCGQ------

DpKaz-1i NECFL-QLE--NC----RSR---SL---VIKKYHG--KCG-------

DmKaz-1g SMCEF-KRR--TC----------DL---RVV-PVSLKNCAL------

ApKaz-1h NECEM-KRE--NC--------GKHV---YVV-PMK--RCL-------

DmKaz-1h SECHM-RKE--NC--------GKHV---FVV-PLK--RCLA------

DpKaz-1h NLCEM-RGK--NC-----GKYVYEV---PMA------RC--------

DpKaz-1d SSCQM-KVK--NC-----GRHVFEI---PIS------NC--------

SmKaz-1f SECHM-FSK--NC-----GKHIFEV---PLA------YC--------

ApKaz-1d NACRM-KSK--NC-----GKHVFEV---PMA------FC--------

DmKaz-1d SPCKM-RSS--NC------G--KHV---FEV-PLS--YCM-------

SmKaz-1a SECEM-KRR--NC----GRP--TVV---ALP-SLK--RC--------

DpKaz-1a NECDL-YRR--NC------G--LEV---ETV-DDK--MC--------

DmKaz-1e SLCEL-KML--NC-----GPQRKSI---QKV-SMD--KC--------

DpKaz-1e SPCEL-RML--NC---GPNS-DKVI---AV--DWL--RCS-------

ApKaz-1e SECEM-KML--NC----GQQTKKKV---TKV-DLE--KC--------

DpKaz-1g TQCEM-KKQ--TC-----GQ--------HVT-ATE--SCN-------

DmKaz-1a NICEL-RKK--TC----SRSGVSLI---KDV-RDG---C--------

SmKaz-1e SACEM-KKR--TC-----GRRVVPV-------KKS--FC--------

DpKaz-1c SICEM-KRH--TC----GQG--------VVKTSEK--FCQ-------

ApKaz-1c NTCQM-KLF--TC-----GQ--GVV---RT--SKK--HC--------

DmKaz-1c STCEM-KLK--TC------G--QGV---VKT-SRK--HC--------

ApKaz-1a NECEL-KKK--TC--------GKGI---VVAESST--QC--------

ApKaz-1g SECDL-KMN--TC-----GQ--KVV---AV--PPH--HCPT------

SmKaz-1c SECQL-RLQ--TC------G--QKV---TTA-SYI--YC--------

TuKaz-6d NQCSL-RRS--SC----LKG--RKL---VSL-FFG--TCD-------

TuKaz-1e NECEM-RKN--AC----LND--MDL---RSL-FYG--QCE-------

TuKaz-5b NECHL-RHS--MC----STG--ITL---TVA-YYG--ECQ-------

TuKaz-8g NQCEM-EKI--SC----QTN--IEL---SVR-FYG--KCS-------

TuKaz-7f NECQM-RVN--AC----QKN--VEL---GIS-FYG--ECQ-------

TuKaz-2d NECQM-RLT--AC----QRK--LEL---SVT-FYG--ECQ-------

SmKaz-2w NKCQF-CRE--MY---EREGKGGLL---SVN-MEG--ECPE------

SmKaz-2r NPCKF-CKG--FY---ALKKAGTEL---NVK-NLG--KCPKHEDC--

SmKaz-2g NKCMF-CYA-----QQKKLKEGVEM---YFD-HMG--MCY-------

SmKaz-3b NKCTL-LWA--SC---------------GLPEPIK------------

SmKaz-3d NKCAL-LWA--SC----------GL----------------------

SmKaz-10 NPCLL-KKA--NC----ESN--GKI---QED-HTG-ASCK-------

SmKaz-4a NRCQL-NVA--NCQSNGRIRLDPTR---ACC-RYN--NC--------

SmKaz-2m NKCKM-CSD--LYRQRKEIDISIHF---QVI-HTG--ICG-------

SmKaz-2s NLCAM-CAA--MY----KEH--KPL---RVV-YEG--VCQA------

SmKaz-2i NKCEM-CAD--SYKKTKTGEDDVDL---KVI-YAG--KCGTFDYCL-

SmKaz-2k NKCEM-CAE--VY-KQRKEDEDFSL---KVI-RPG--KCESFDYC--

SmKaz-2b NKCSM-CAD--MYMNNKKEGKDVIL---TVK-HYG--VC--------

SmKaz-2c TKCNM-CAH--KY----KQG-DVKL---RVV-HPG--RCG-------

DpKaz-7 NFCLM-HRD--AC----LTG--VHI---SLK-KKG--YC--------

ApKaz-4a NSCLA------KC----AGLSDVDL-------KFG--TCWN------

SmKaz-2d NRCTF-CYA-KNQ----EETSGKSL---SME-YTG--EC--------

ApKaz-6 NKCVM-EKY--NC----EHN--AKL---TVK-MET--ECPN------

SmKaz-2v NLCVM-CAE--MYKQRKNGHFDFVL---RKL-HEG--IC--------

SmKaz-2e NLCML-CVE--KY-NQHRQDSGMAL---RLE-HEG--ACK-------

SmKaz-2o NLCML-CAE--IY-RQKKRGTYINL---AIQ-HNG--ECQI------

SmKaz-2j NLCML-CAE--IYRQKQAGT-YEKL---SFL-HTG--ECP-------

SmKaz-2f NPCTF-CYS--MF---EKQAKGNDI---RVK-REG--QCL-------

TuKaz-3 NECKL-RME--SC----KRA--INL---FVR-YPS--ACD-------

SmKaz-2q NHCML-CAA--MY----NGS--DNL---KVI-NRG--PCKKVE----

SmKaz-6b NPCSF-LWE-HAC---------------------G------------

DmKaz-2a SACDI-NRM--IC----KIG--RSI---AVA-YPG--PC--------

ApKaz-4c SECAA-FAE--ST---------------SVD-YSG--PCAS------

TuKaz-8h NECSM-MEK--SC----HNR--KPL---TIV-SPE--IC--------

DmKaz-12 SECEL-QLV--KC----RTG--HPW---RKQ-HDG--PCE-------

DmKaz-5c NECYL-ERV--RC----KDP--KSI--WKLS-HKG--ECA-------

ApKaz-9 SRCAL-LRV--RC----FND--SLL---RVK-HRG--RCKEKQPCW-

ApKaz-4b NLCHL-LRS--GK---------------TLA-YSG--PCLV------

SmKaz-2n SRCVF-CFN--KH---HPIHQDNEI---VLE-HVG--KCLY------

ApKaz-2g SLCLM-KME--GC----QRQ--QEL---RLR-PME--LCQGMEV---

SmKaz-4b NKCFF-RIA--AC-------RDKNI---RYD-RKG--AC--------

SmKaz-4c NDCRL-SIA--KC---------HDK---RVV-YDSTGACLD------

TuKaz-7h SECQM-KFF--AC----RMQ--RKI---NLV-HHG--QCRNT-----

TuKaz-6a SECDI-ERV--SC----LKN--EPI---AIK-HHG--PCT-------

SmKaz-2t NKCAL-CYA--MS-----QR--HDL---KLD-HPG--VCI-------

ApKaz-1f NQCQL-DQA--TC----LRG----I---QMA-HLG--KCSS------

SmKaz-2h NICVF-FAK--MD---------LSL---YMV-HSG--SCTIYDSCS-

SmKaz-2l NTCLF-QVA---------TLIDTSL---YIA-HPG--KCTIYDL---

TuKaz-1b SVCHL-LRD--SC----LKK--VNL---TVS-YSG--PCE-------

SmKaz-2a NKCFF-GFA---------QKTKPTL---TIK-HSG--ICTKSK----

DmKaz-1f NECEL-AHA--TC----LRG----V---NLA-HIG--PCT-------

ApKaz-2c SKCLL-ERI--GC----SKR--SGV---TVA-YDG--HCGDA-----

SmKaz-2p NDCEM-KIA--RC-----KN--PNL---TEN-HAG--SCEFQ-----

SmKaz-2x NNCER-CAE--MS----RRHQAGENIFIYNK-YKG--KCP-------

ApKaz-2a SACHV-RKA--SC----ASG--RNI---AVK-YRG--LCDPCD----

TuKaz-1f NLCSL-KEE--SC----RRQ--IEI---KSV-DSD--QCN-------

TuKaz-7g NECKL-QEE--AC----RRQ--QEI---QPA-LNR--LC--------

TuKaz-2e NECKL-QEE--AC----RRQ--QEV---ALE-SRD--KCD-------

TuKaz-5c NECKL-QEE--AC----RRQ--QDI---KPE-NRS--MC--------

DmKaz-10 NSCEL------KC-----AAQIKLI---YVV-KAG--RC--------

DmKaz-2b SWCEM-HKH--SC----ESR--YFI---GVK-SQG--SC--------

ApKaz-5 NMCQL-NEA--AA---ELGHNATQL---RIQ-YRG--PCQS------

DpKaz-2c NACQL-ARS-NAC----LHP--PEV---KVC-HKG--PCKF------

ApKaz-2f SECEL-LQR--AC----VKNGTHNL---TIL-FYG--DCK-------

DmKaz-3 TRCHL-LRA--QC-----GG--HQV---SLK-YSG--SCNAC-----

SmKaz-2u NDCVF-CMY--KS---DREKDGVKV---GLA-HRG--EC--------

DpKaz-1f SPCHL-RMA--SC--------TRGT---ELA-HVG--ACMKITPDNS

DpKaz-1b NICVL-KVE--TC--------KRGI---RLS-HVG--PC--------

ApKaz-1b NRCMM-QVE--IC---RVGT--S------MS-HLG--PC--------

DmKaz-1b NRCML-RVQ--SC-----RVGLAAV---KLS-HVG--PC--------

SmKaz-1b NECKW-AQQ--AC--------ATGI---RLA-HPG--TCHN------

DpKaz-5b SQCHL-QQE--AC----RSQ--RHL---RIL-YKG--LCESGTYGC-

TuKaz-8b NSCEL-KRW--SC----IHS--ADV---TIK-YHG--KCD-------

ApKaz-2b NECFL-RRQ--AC----RMM--PGL---RIV-FHG--PCD-------

SmKaz-9 NVCEL-RRA--AC----QNM--KDV---EER-YQG--KCGE------

TuKaz-7a NECEL-RRK--AC----VSN--KEI---SVK-FQG--KCD-------

DpKaz-5a NMCEL-HKS--SC----LAN--RMI---AVK-FQG--SCDPCA----

ApKaz-7 SLCRL-DYH--NC----LHT--TNI---KVN-CKGFCPCK-------

DpKaz-3a NECVL------SV----ETKVLLKA---GIG-RKA--ICV-------

DmKaz-8 NRCEF------DCVRRNVERQGRSM---GLL-RDG--TC--------

DmKaz-4 NQCVL------DC----LIKEGRSI---TVE-KKG--RC--------

TuKaz-6b SQCSL-STA--CC----TEK--RYI---TVR-YDG--PCQ-------

TuKaz-6e SECQM-LRA--SC----LHQ--TDI---VIL-HKG--PC--------

TuKaz-8i STCQL-NFV--AC----RNQ--KIL---TVI-KIG--SCN-------

SmKaz-1d NDCAF-RVA--AC----QKG----I---NRA-HEG--IC--------

TuKaz-5a NECLL-NLN--AC----KKN--VAI---LVA-YHD--TCD-------

TuKaz-2f NECQL-KIY--SC----RMQ--RPL---TTL-YKG--PCK-------

DpKaz-3b NACALECSKKQTC-----GQHVIPV---ALH------HC--------

TuKaz-1a SSCHL-QLS--SC----KLQ--QTI---YVK-HEG--NCH-------

TuKaz-8d SVCDL-QRK--SC----LLN--QPV---KVK-HYG--SCS-------

DpKaz-4d SECDL-RLA--AC----RKQ--LNV---VMA-YEG--PCS-------

SmKaz-5a SFCHL-RQA--AC----QKG--RAI---PVA-YPG--KC--------

ApKaz-3b SACHL-REA--AC----HAG--NAI---PIA-YKG--LCKE------

TuKaz-2a NQCQL-NFT--SC----NLQ--TPL---TVQ-YKG--KCE-------

DpKaz-4c NECEL-KQA--AC----RNQ--QFI---VVA-SKG--DCD-------

TuKaz-5d SECGL-HLY--AC----RMQ--KNL---TVL-YQG--ECT-------

ApKaz-2h SECQL-KLY--AC----RYQ--KDI---VVK-SHT--SCKDE-----

SmKaz-8 SECQL-RMA--SC----NQQ--KRI---LVK-YKG--SCE-------

TuKaz-8a SECQL-KLA--SC----NQR--TTL---FVK-HPG--ECQV------

ApKaz-3a SHCRL-KKR--SC----RTK-DQSL---LVD-YHG--LCQS------

ApKaz-8 NRCEL-HKS--GC----LSG--VDI---QID-HSL--KCFIP-----

SmKaz-7 NHCEL-HRA--SC----VTG--TSI---TID-RKN--SC--------

TuKaz-7c SECEL-RRE--AC----LLH--KNV---TLV-YKG--LCG-------

DpKaz-4a SNCEL-ERE--SC----LQK--RSI---KLL-YEG--VC--------

TuKaz-7b NECIL-RVE--AC----KAR--KSL---RIL-YNG--ECG-------

DpKaz-3c NENCM-EVEVTQC----GTVIDSPI---RKA-HDG--ECK-------

DpKaz-2e NECEL-NGR-NKC----DGT---KI---EKA-HDG--EC--------

DpKaz-6a NKCTL-FVE-NNC----DDTSPNRI---RKA-YDG--EC--------

DpKaz-2b NICHL-NAY-NKC----HGT---HI---EKA-HDG--PC--------

DpKaz-2a NPCSL-NAK-NQC----DGT---AI---SKA-YNG--RC--------

DpKaz-2d NPCVL-KAK-NTC----DGT---RV---HKA-YDG--VC--------

TuKaz-6c SECEL-QMA--SC----HLQ--INL---TVA-HHG--SCA-------

TuKaz-1c NECEL-HKA--SC----QDQ--KAI---YIS-HKG--PCR-------

TuKaz-1d NECEL-RVR--SC----LAQ--TDI---TIA-HLG--PCD-------

TuKaz-2c NECQL-RLT--SC----QQR--QYI---TVT-SKV--QCD-------

TuKaz-7e NECEL-RLA--SC----TKK--EYL---TIA-SKG--PCD-------

TuKaz-2b NECVL-RRE--SC----KEN--LNL---RIV-HSN--ECG-------

ApKaz-2e DLCHL-QNA--SC----ATR--KKI---FAA-YAG--ECGS------

ApKaz-2d NECKL-KYH--SC----QQK--QII---GVS-HNG--TCNDCTK---

DpKaz-4b NPCRL-RYE--SC----RHN--KSL---SIV-YKG--LCNLIDDT--

TuKaz-8c NECFL-RVE--SC----RSR--HNL---YII-HQG--DCN-------

TuKaz-8f NECSL-RVT--SC----KQQ--TYI---AVE-SKG--ACN-------

TuKaz-8e NPCKL-ARE--SC----EKR--RSI---EVA-YKG--LCG-------

TuKaz-7d NECKL-RRE--SC----EQQ--KSL---KVI-SQG--YCN-------

1. **I2 Kunitz-A.**

DmKun-4 --------------------CYDELTDS------P------CR-DNDVA--N---FWY-Y

DmKun-15 SLGLPSLENQTHEQIEQIIACRQP-KAP-G----L------CR--GHQ---L---RYA-Y

DmKun-9 -----------------KRRCLQP-LDV-G----K------GK--AYL---R---NWF-Y

DpKun-5c --------------------CENV-HDQLN----SHESIERCN--QFS---I---KWY-Y

DpKun-1d ----------------AEERCNMT-ADY-G----R------CQ--GNQ---L---RWH-F

ApKun-4 ------TVFDVKALENFKERCEIP-IMR-G----P------CQ--NWI---H---KWY-Y

DmKun-6 --------TKMGSYKVRQEKCLFI-PSY-G----R------CK--KHI-------AVYGY

TuKun-2 -----------------REHCKQA-HYY-G----Y------CE--QNV---T---SYH-F

TuKun-5 ------------------NHCRQT-HYY-G----Y------CQ--QNL-------TYYHF

SmKun-1f -----------------ADVCALP-SDH-G----P------CS--SAAE--E---RWY-F

DpKun-1c ------------------DKCEKP-QDAGG-----------CQ--GTF---Q---RWS-Y

DmKun-5h -------------------KCFLA-FEP-G----N------CY--NNV---T---RWF-Y

DmKun-5l --------------GSAKEICLLP-VAT-G----R------CN--GPSVHER---RWY-Y

TuKun-4a ------------------AVCKLA-PQP-G----P------CY--SYN---P---RYF-F

SmKun-2b ------------------EVCSQP-QDP-G----L------CM--AAI---P---RWS-F

DpKun-2d ----------------NQESCLLP-VAK-G----P------CF--GFM---K---RYG-F

DpKun-3e -----------------QEICQSP-VVK-G----P------CF--ALW---K---RFA-F

DpKun-19 ------------------DVCFQE-KLL-G----S------CK--ASV---L---RYS-F

DmKun-5i ------------------TTCALP-PVR-G----R------CS--DLS---R---RWY-F

DmKun-5f ---------------SRQDVCDEE-PAP-G----E------CS--TWV---L---KWH-F

SmKun-2a ------------------VICSLP-RRA-G----S------CE--ALL---E---RWF-Y

DmKun-16 ------------WVHAKPEMCQQP-SSMVG----MAQDGAACM--AFM---P---AWT-Y

DpKun-3c ------------------EKCSLP-PVNPS----P----FSCL--AFI---P---SWT-F

DpKun-3d -----------------DSKCTLP-PVTPS----P----FSCL--AAI---P---SWT-F

DpKun-22 -------------------MCSLP-PVD------P--SNISCF--AFI---P---SWT-F

DpKun-2c -------------------ICSLP-PDN-S----K-TTGRACM--AFV---P---SWT-F

DpKun-9b -------------------SCDLP-PVA-K----Q-STA-TCL--AYF---P---SWT-F

DpKun-9a ----------------GIEICDLP-PIE-N----K-GFE--CY--ALK---H---SWT-F

ApKun-1e ------------------DTCKLP-ALV-G----E------CH--DYV---N---RWY-F

DmKun-5e -----------------KDICEIP-AEV-G----E------CA--NYV---T---SWY-Y

DpKun-17 -----------------PDYCSLP-PVMAG----E----KICK--GYI---R---KWT-F

DpKun-4c ------------------DVCSLRVVNP-G----P----KRCKINTQR-------RWT-Y

DpKun-2a -------------KVDDEDDCSLPAIQP-GIKK-S------CS--GFL---T---RWT-F

DpKun-3a -----------------VDVCSLP-PVN------P--SPIACS--GLI---P---RWT-Y

DpKun-4a -------------NNEEVDICALP-PVNPG----S----KACR--GFF---R---RWT-Y

DpKun-6 ----------------SDDICMLP-PLK-G----T----LSCS--GVF---Y---RWT-Y

DpKun-7a -----------------PSVCFLP-PIE-G----S----IQCK--GFF---I---RWT-Y

SmKun-1k -----------------SEICRLP-PDA-G----P------CT--NFE---R---RWH-Y

DpKun-1k ------------------VVCRLP-MDV-G----P------CR--ERY---D---RWY-F

ApKun-1h -----------------SDVCLIP-LDP-G----P------CL--QTV---D---MWY-F

DpKun-1i ---------------TGGDICGLE-VEP-G----P------CR--ASV---P---AWY-F

SmKun-1i ----------------SADPCTLP-RDV-G----P------CQ--SSV---H---SWY-F

DpKun-21 --------------------CSQP-AET-G----H------CW--ALF---P---RYF-Y

DmKun-19 ------------SELTVPEDCHQP-KET-G----R------CF--ALF---Y---RYA-Y

DpKun-11 ----------------IVNVCELP-KEP-G----M------CR--ANF---P---RWA-Y

SmKun-2d ------------------NVCEQP-KRV-G----M------CR--ASM---R---RWY-F

TuKun-1 -----------------TDICLAP-LRS-E----P------CL-TGMLA--EPIRRWY-Y

TuKun-4b ---------------KREDICFQP-FAE-G----T------CK--ENL---I---RYY-Y

DmKun-11 --------YEREQYNIRKKICLQS-SEY-G----K------CK--GRR---K---LWF-Y

DpKun-8a ----MSLMLVTLQAAQFDDVCSLK-RES-V----K------CR--TLL---P---TWY-F

DpKun-20 -----------------TDVCSLK-RES-V----K------CQ--TLL---P---NWY-F

ApKun-1d ------------------EECLMT-VAR-G----D----KNCD--KKI---P---RWY-F

DmKun-12 -----------------KAFCYLP-YEF-G----K------CG--GHR---I---MWA-F

DmKun-5d -----------------KDRCALP-KQT-G----D------CS--EKL---A---KWH-F

SmKun-4 -------------------DCYAS-SEP-G----P------CD--ALI---P---KYY-Y

DmKun-7 ----------------KQPKCWYV-ANP-G----P------CD--DFV---K---VWG-Y

ApKun-2 -------------SSLAKEICSME-KNE-G----A------CN--VNA---V---RYY-Y

DmKun-13 -----------------YEKCAGP-GDP-G----P------CK--QYI---Y---KWR-Y

DpKun-2g ----------------KPSVCEMP-QEV-G----P------CK--GQV---P---AYF-Y

DmKun-3 -----------------HERCSFI-ANP-G----P------CK--GNF---E---MFA-Y

DpKun-1f ------------------DTCRMP-RVI-G----D------CK--EFT---E---RWY-Y

SmKun-6 -------------------YCDNP-AKK-G----N------CD--GEI---Q---RFY-Y

DmKun-14 -------------------ICVQA-PDP-G----P------CR--GTY---M---RYA-Y

DpKun-10 ---------------TTTHACQTP-ADP-G----P------CT--GEL---I---RFF-Y

DpKun-14 ------------------AICMEE-VDV-G----P------CR-SSSF---P---RWY-F

TuKun-3 -------------------VCMTP-KDV-G----P------CR--GYY---P---RWY-Y

SmKun-5 ------------------EICMQE-SHV-G----P------CR--GYF---P---RWY-F

DpKun-5b -----------------DPVCLLP-PVA-D----P---TQNCN--NFS---I---KYY-F

DpKun-2f -----------------LQRCLMP-MHI-G----P------CR--MSL---E---KFY-Y

DpKun-2i -----------------INRCKLP-ADV-G----F------CR--SFQ---E---RFY-Y

DpKun-3h -----------------FARCKLP-ADV-G----M------CR--GFA---Q---RFF-Y

DpKun-8b ----------------PANVCSLK-KDS-G----M------CR--AAV---T---AWY-F

ApKun-1c -------------------PCEQP-LTP-G----P------CK--GNF---S---RWY-Y

DmKun-5c -------------------TCEQP-VES-G----P------CA--GNF---E---RWY-Y

DmKun-5b -----------------KHVCLLP-KSA-G----P------CT--GFT---K---KWY-F

SmKun-1g -----------------LEDCHLD-KAY-G----R------CD--DST---I---RWY-Y

SmKun-1c -----------------EDPCQLP-KLE-G----P------CN--NNY---I---SWY-F

DpKun-1e ------------------DSCLLP-RAE-G----P------CS--EKK---S---RWY-Y

SmKun-1h -----------------QEICELP-KVV-G----P------CS--GQF---S---QWY-F

ApKun-1g ---------------EAQDLCQLP-KVE-G----P------CR--GDF---R---QWY-Y

DpKun-1h ------------------DVCILP-RVV-G----P------CS--GSF---R---QWY-Y

SmKun-1b ------------------NACQLP-KAT-G----Q------CT--TYT---V---AWY-Y

ApKun-1b ------------------DVCYLP-KSV-G----P------CE--GYY---P---TWY-Y

DpKun-1b -----------------IEACSLP-RVA-G----P------CE--GNY---P---SWY-H

SmKun-1j -----------------HDVCSLP-NEH-G----P------CV--GEF---P---KWY-H

SmKun-1e ----------------VGDVCSLP-KVE-G----P------CQ--ERA---V---KWY-H

SmKun-3 -----------------YDVCSLP-KVE-G----P------CK--ELS---V---KWR-Y

DmKun-10 ----------------ADEICQLT-PEANGFGKIM-----SCA--HYS-------NWFSY

DmKun-5k ------------------DVCNEP-VTT-G----P------CT--DWQ---T---KYY-F

DpKun-1j -----------------QDVCRLP-PDR-G----P------CR--GSF---R---KYY-F

DpKun-5a -----------------ASGCFLP-LSR-G----I------CR--AHW---V---RYY-F

DpKun-13 --------------------CFLP-LSK-G----F------CR--AHW---V---RYY-Y

DmKun-2 -GSSSGNGAVVSCKELNNFNCYVG-RNE-G----N-----FCSRKDQTKVVT---RWY-F

DmKun-5g -----------------VAQCSQP-ADP-G----Q------CD--KWA---L---HWN-Y

DmKun-5j -----------------YSVCAEP-PEA-G----E------CD--NRT---T---AWF-Y

DmKun-21 ---------------KRVKLCLQP-MIS-G----R------CF--GYV---E---SYA-Y

DmKun-20 ---------IKPQRLVPDPKCLQP-LDV-G----P------CR--MSL---E---RFY-Y

ApKun-3 -----ILICMPVTANLTRDRCFLP-PDE-G----N------CG--NRLTLRV---KYY-F

SmKun-1d -----------------KEACLLP-KEV-G----P------CS--GQY---T---RWF-Y

DmKun-17 ----------------KNAICGLP-HSLNGDGRIS------CE--AYI---P---SWS-Y

DmKun-18 ----------------KDPICGLP-AGIDGNGLIK------CA--AFI---P---SFS-Y

DmKun-1 --------ANTGRNTHPEQFCLMP-ARK-G----V------CR--ALI---P---RWR-Y

SmKun-2c ------------------EKCSQP-RVV-G----V------CR--ASY---R---RYN-Y

DpKun-2h ----------------VAEHCKLP-ADI-G----P------CR--AAK---P---RYH-Y

DmKun-8 ----------------KNEICGLP-AAANG----N------CL--ALF---S---RWS-Y

DpKun-2b -----------------PSPCHLP-SAA-G----Y------CR--AHI---P---SFY-F

DpKun-2e -------------------LCLLP-RDI-G----R------CR--ASV---P---SFY-Y

DmKun-5a ----------------PQKACGLP-KET-G----T------CN--NYS---V---KYY-F

SmKun-1a -----------------GVVCGLV-EDR-G----S------CS--NYT---V---KWR-F

DpKun-1a ------------------EICALD-KDR-G----S------CR--NFT---V---GWF-F

ApKun-1a --------GCQSKPILPGDRCKEP-KDR-G----S------CS--DFT---V---KWF-F

DpKun-1g -----------------TEFCFLP-KQE-G----S------CD--ESV---L---QWF-Y

ApKun-1f -------------------KCFLN-QDR-G----N------CS--NMS---S---KYF-Y

DpKun-3g ----------------RPSVCLQP-KVT-G----P------CR--GLE---T---NYF-F

DpKun-7b ----------------GSSICLQK-KDE-G----S----RSCS--ASI---P---SYF-F

DpKun-4b ------------------APCMQP-KAE-G----L------CR--AVI---P---SFY-F

DpKun-3f ------------------DLCFLP-KVI-G----P------CK--MSR---P---SFH-F

DpKun-18 --------------------CMQP-KAV-G----S------CR--GSI---P---SYF-F

DpKun-3b ----------------ATAPCMQP-KAT-G----N------CR--AFI---P---SFF-F

DpKun-4d ------------------SPCMQP-KAA-G----N------CR--ASF---H---SFY-F

DpKun-16 --------------------CLQP-KLI-G----N------CR--SSI---P---SFY-Y

DpKun-12 ------------------SACLKP-KVT-G----P------CR--AAI---P---SFF-F

DpKun-15 --------------------CLQP-KVI-G----P------CR--ASI---P---RFF-F

* .

DmKun-4 DHVS-----DQCAIYWSDRCDTN-R--NKFKSKEECEETCRLPRHK--------------

DmKun-15 NKKT-----GNCESFIYTGCAST-E--NNFLTFEECRRDCMQRLRY--------------

DmKun-9 NSTS-----QRCQRFIFYGGASN-G--NNFNTQARCHKICLAQVSLPINFNSTAA-----

DpKun-5c DSAT-----RDCEKIFYTGCGGS-E--NLFASEDLCEMRCDQD-----------------

DpKun-1d DSKS-----RHCHSFLYSGCGGN-A--NRFESYQACASICKEAP----------------

ApKun-4 DSIL-----HQCRTYISGICKSE----NIFDSEAECLYYCVGAK----------------

DmKun-6 NIIT-----NRCSEFTYSGCGGN-P--NRFMTDSQCRNTCYVVPARKTVSEPDYYADDGV

TuKun-2 DTAT-----NQCLPFTYSGCGGS-I--NTFDTLKQCTDYCT-------------------

TuKun-5 DTKT-----NQCLPFTFSGCGGT-V--NTFATLKACTDYCTA------------------

SmKun-1f NQNS-----GRCDQFIYGGCGGN-G--NNYVSLAECEHRCGVKI----------------

DpKun-1c DKTS-----MTCQEFNWGGCQGN-E--NNFLSERECHLRCKDTSRSRV------------

DmKun-5h NSAE-----GLCDEFVYTGCGGN-A--NNYATEEECQNECNDAQ----------------

DmKun-5l DDEA-----GNCVSFIYAGCSGN-Q--NNFRSFEACTNQCRPEPNK--------------

TuKun-4a NDET-----KTCELFIYGGCQGN-G--NNFETQKDCMSLCSLTS----------------

SmKun-2b HAPT-----NTCQEFTYGGCGGN-L--NNFQTQADCRFVCPVI-----------------

DpKun-2d NKEK-----NRCELFTYGGCQGN-F--NNYVTADQCFDACGGALPSLASECEQ-------

DpKun-3e IKEK-----NRCELFYFGGCQGN-R--NNFRTADDCYKTCGGDEPN--------------

DpKun-19 NEKR-----NRCEAFRYTGCSGN-M--NNFDSERECKAVCP-------------------

DmKun-5i DERS-----GECHEFEFTGCRGN-R--NNFVSQSDCLNFCIGEPVVE-------------

DmKun-5f DRKI-----GACRQFYYGNCGGN-G--NRFETENDCQQRCLSQEP---------------

SmKun-2a NSDT-----SRCESFVFGGCGGN-L--NNFITRDDCEKSCVVN-----------------

DmKun-16 DASK-----NACTEFIFGGCGGN-S--NQFSTKSECEKACKD------------------

DpKun-3c NSTT-----GECQSYVYGGCGKT-A--NLYNSHDECNTACGPD-----------------

DpKun-3d NSTA-----GKCESYLYGGCGKT-A--NLFNSQDDCNAACGPK-----------------

DpKun-22 NSAL-----GRCQSFVYGGCGRT-A--NLFDTQNDCDAACGSESNSGETIRF--------

DpKun-2c NSTS-----NKCESYVYGGCGKT-A--NLFRTEEACQSTCGST-----------------

DpKun-9b DSKS-----GKCKQYVYGGCHKT-E--NLYETEADCLSKCGPAVSK--------------

DpKun-9a KS-------GKCVNYVYGGCLGT-E--NLFDTEEAS---C--------------------

ApKun-1e NSLD-----GRCRQFYYGGCGGN-E--NNFETEYNCENKCIDSGRIT-------------

DmKun-5e DTQD-----QACRQFYYGGCGGN-E--NRFPTEESCLARCDRKP----------------

DpKun-17 NETE-----SACASYVYGGCNGT-K--NLFDTEEECQAACP-------------------

DpKun-4c SAKT-----KACEIFEYIPCTSK-DPPNLFLNEYACLANCNQQ-----------------

DpKun-2a N--N-----GACQQITYGGCGGT-K--NLFETEYACNAKCNR------------------

DpKun-3a NAKA-----GLCEKYTYGGCFGT-E--NLFKNEFACLAKCNK------------------

DpKun-4a DIKT-----ETCATYIYGGCGGT-E--NLFQTEFACLAKCNKPGL---------------

DpKun-6 NSTT-----EVCEKFVYGGCQAT-E--NVFRNQHACLAKC--------------------

DpKun-7a NAQT-----EKCEKFIYGGCFGT-A--NLFRNQHACLAKCN-------------------

SmKun-1k DSVR-----GTCIPFDFGGCRGN-K--NRFKSFDVCLGFCS-------------------

DpKun-1k DSER-----STCQPFVYGGCAGN-M--NRFKSFESCTTFCSPSDRTRPAEPA--------

ApKun-1h KTSS-----RRCESFSYSGCEGN-A--NKFQSVEECERICHPYIDPNA------------

DpKun-1i NRQT-----SRCEAFSYGGCDGN-A--NRFHSEEQCERQCGSF-----------------

SmKun-1i DGQE-----RACKAFVFGGCGGN-T--NRFVSEESCKRACGEF-----------------

DpKun-21 NVSS-----RSCEEFISGGCEGN-E--NNYESVQECSQHC--------------------

DmKun-19 NVDT-----QSCEEFVYGGCAGN-K--NNFESKEQCEQACLVKSAVSSTDSTTEQNSEVA

DpKun-11 NPET-----QLCEKFSFGGCGGN-A--NNFHSHQQCASRC--------------------

SmKun-2d DQFS-----HRCEEFIFGGCDGN-D--NNFETREECEARCPDL-----------------

TuKun-1 DNQS-----QLCLPFNFTGCEDI-S--NNFKTKQDCEILCRQML----------------

TuKun-4b DPEV-----NKCIKFIFKGCFQY-D--NNFEKLEDCESLCVI------------------

DmKun-11 NPKK-----SKCQVFIYSNCGGN-G--NLFYTKESCVEFCGKYDWKKVRKTGLRRSADYR

DpKun-8a DKAT-----GTCYSFNIGECSRN-F--NSFGTKKICEKRC--------------------

DpKun-20 DKAT-----GTCYSFNIQKCSRN-V--NSFGTKKICEKRCA-------------------

ApKun-1d DNNE-----NACKPFYYTGCGAN-A--NNYETQESCEKKCPSKR----------------

DmKun-12 SNKE-----QECVPFVFSNCGGN-E--NRFYTKENCEKACATIQSRFVLAN---------

DmKun-5d SESE-----KRCVPFYYSGCGGN-K--NNFPTLESCEDHCPRQ-----------------

SmKun-4 SHQS-----DTCQAFTYGGCGGN-L--NKFDTVQECEDKY--------------------

DmKun-7 DYLT-----NRCIFFYYGGCGGN-P--NRFYTKEECLKTCRVYRPPNRKKREENLDEEEE

ApKun-2 DKTF-----KTCKQFKYGGCRGN-E--NNFKSAKECYKVCHGVV----------------

DmKun-13 EPTT-----NECTNFIWGGCEGNPQ--NRFGTEAECLFHCIGGPHT--------------

DpKun-2g NKDS-----GACESFWFGGCRGN-A--NRFETEAECQTKCIPSSLTPV------------

DmKun-3 DMDN-----NVCVEFIYGGCGGN-P--NRFQTKKECILLCNALADEDEYLIVYTDKNEQQ

DpKun-1f DEAD-----EECRAFLFGGCNGN-A--NNFDSMDSCNQRCKSTVSPIVPE----------

SmKun-6 DEKS-----HSCKKFIYTGCDGN-E--NNFHTEAQCMEFCSEN-----------------

DmKun-14 DPQN-----QHCYSFTYGGCRGN-R--NNFLTENDCLNTCNVLRSPYSSR----------

DpKun-10 DSTA-----LRCRQFIYGGCEGN-K--NNFGTEADCMKMCAHTLVD--------------

DpKun-14 DAHK-----GMCISFNYGGCRGN-R--NNFEKREDCVNTCE-------------------

TuKun-3 DGDK-----AMCLQFIYGGCRGN-R--NNFERYTDCSKMCEVLLRDFVKA----------

SmKun-5 DLGK-----AMCLQFIYGGCRGN-K--NNFERYEECNEMCAF------------------

DpKun-5b DPVD-----GDCEDFLYSGCGAT-E--NVFNSELACELRCENVHDQLN------------

DpKun-2f DAEK-----KDCLLFFYGGCKGN-S--NQFDTVEECRQTCRVKSED--------------

DpKun-2i DSIE-----SQCKTFSWGGCRGN-S--NNFPTSEECMVTCDRQGKLAA------------

DpKun-3h DNAD-----KECKPFTYGGCLGN-A--NNFPSQEECHSACAPPVIDAKAR----------

DpKun-8b DPPTDSRNKGECKNFLYGGCGGN-A--NRFASKKKCDDMCV-------------------

ApKun-1c DKST-----RSCSQFNYGGCKGS-Q--NNFLNKESCNHKCINPL----------------

DmKun-5c DNET-----DICRPFTYGGCKGN-K--NNYPTEHACNYNCRQP-----------------

DmKun-5b DVDR-----NRCEEFQYGGCYGT-N--NRFDSLEQCQGTCAAS-----------------

SmKun-1g KKDT-----GVCEQFHYSGCNGN-N--NRFETRPECEEKCFHS-----------------

SmKun-1c DKDE-----VMCKEFRYGGCKGN-R--NNFPTERDCIQQCASK-----------------

DpKun-1e DQAE-----RRCMPFYFGGCQGN-A--NNFESQNACEESCRALSLV--------------

SmKun-1h NPQA-----DQCEEFQFGGCLGN-G--NRFNTFEDCQARCKKTV----------------

ApKun-1g DKNS-----DRCFQFQYGGCRGN-T--NRFNDRQTCETRCVQN-----------------

DpKun-1h DAGS-----DNCYEFDYGGCQGN-P--NRFNNAQECQNRCQRVRPITTT-----------

SmKun-1b DVPS-----GQCGQFYYGGCLGN-N--NRFATQEECERTCGHT-----------------

ApKun-1b DQDR-----KQCAQFVYGGCLGN-N--NKFQTREECEHLCVIPDT---------------

DpKun-1b DTTT-----GSCKQFRYGGCLGN-T--NRFSTREDCNQQCIAPK----------------

SmKun-1j DPTD-----NECKTFVYGGCSGN-G--NRFESKKECEQVCIAR-----------------

SmKun-1e DSNE-----DRCLQFYYGGCDGN-A--NRFETELECQHVCSA------------------

SmKun-3 DSNK-----DRCLQFYYGGCDGN-A--NRFETELECQHVCS-------------------

DmKun-10 HSDK-----NECLEFSYGGCGGN-E--NRFQTKAICEDLCKNKVEQL-------------

DmKun-5k NTAS-----QACEPFTYGGCDGT-G--NRFSDLFECQTVCLAGRE---------------

DpKun-1j DRSS-----LQCLELVYGGCRGN-G--NRFSSLEECQSLCLQRAEVAPPGNVTS------

DpKun-5a DPSS-----RSCQSFVYSGCGEN-G--NNFHSLNECRLASAVRV----------------

DpKun-13 DPSS-----RSCKSFVYGGCDGN-G--NNFHSLNECRLACLN------------------

DmKun-2 D--K-----GVCKPFNYKGCNGN-R--NRFCSQESCDARCGD------------------

DmKun-5g NETE-----GRCQSFYYGGCGGN-D--NRFATEEECSARCSVNIDI--------------

DmKun-5j DSEN-----MACTAFTYTGCGGN-G--NRFETRDQCERQCGEF-----------------

DmKun-21 NPIK-----RHCEPFIYGGCGGN-D--NRFSTKAECEFNCRDI-----------------

DmKun-20 NKDS-----KACETFKYGGCRGN-D--NRWGFRQTCEEACIPKK----------------

ApKun-3 DSAN-----DDCSEFIYFGCGGN-N--NRFDTFEECENVCIFYGF---------------

SmKun-1d NIVE-----SQCKPFNYGGCQGN-T--NRFESLQQCQVSCND------------------

DmKun-17 DADR-----NECVKFIYGGCGGN-N--NRFNSREICEDKCLQ------------------

DmKun-18 HPET-----NSCEKFIYGGCGGN-E--NRFGTQELCEQKCKE------------------

DmKun-1 DPEQ-----KKCVEFKFGGCDGN-E--NNFASYKDCMSTCEGM-----------------

SmKun-2c NRTT-----EQCEQFVYGGCGGN-D--NNFNSMEECAAECDA------------------

DpKun-2h NLTA-----GECQPFNFGGCRGN-N--NNFQTIEQCQSECAAGGAVN-------------

DmKun-8 DAQY-----NVCFNFIYGGCQGN-E--NSFESQEECINKCVE------------------

DpKun-2b DSVS-----GECKSFVYTGCKGN-A--NNFPSMEDCRKTCKVRQI---------------

DpKun-2e DADQ-----LKCVLFNFGGCHGN-E--NRFSSEAECLSTCQHSDVAETG-----------

DmKun-5a DTSY-----GGCARFWYGGCDGN-D--NRFESEAECKDTCQDYTG---------------

SmKun-1a DMEY-----GGCARFWYGGCEGN-D--NRFDSQEECEEVCVIP-----------------

DpKun-1a DMEY-----GGCSRFWYGGCDGN-D--NRFPTQDDCKAHCVEPIG---------------

ApKun-1a DTEY-----GGCSRFWYGGCNGN-N--NRFKTQEECKDICVEP-----------------

DpKun-1g DRPE-----GVCKQFIYKGCDGN-Q--NRFADRQECESRCSQSQ----------------

ApKun-1f DRQD-----GVCKPFMYGGCGGN-D--NRFESKQECERQCF-------------------

DpKun-3g DSTK-----EKCLAFNYGGCEGN-D--NRFETLEKCRQVCGEI-----------------

DpKun-7b EATS-----GLCKPFRFSGCDGN-G--NRFPTEQECEQACYYGPSVAAVCDPALSTV---

DpKun-4b DVQT-----GKCTMFDYSGCHGN-S--NRFATEEECEQECYDFPN---------------

DpKun-3f DATK-----GDCRPFLYGGCKGN-E--NRFETLEACLDTCSSVA----------------

DpKun-18 DMQT-----GQCTPFNYGGCGGN-D--NRFKSYHECDFKC--------------------

DpKun-3b DTQT-----GLCTSFTYTGCGGN-D--NNFSSEDECDLKCNGLQG---------------

DpKun-4d DKKT-----GMCTAFTYTGCGGN-E--NQFSSEEECYLKCNNV-----------------

DpKun-16 DATT-----GVCRPFNFSGCDGN-S--NNFGSVKSCERACMGPDFLSDLPPSMSN-----

DpKun-12 DATT-----GVCTPFNYGGCGGN-D--NRFATEKACQLACSAPTNSEENN----------

DpKun-15 DATT-----GVCTPFNYGGCRGN-D--NRFISEKACQLAC--------------------

* * : .

1. **I4 Serpin.**

DmSRP-14 -------------------MFKRVLICFLIFLLKHSYAYN------FEINLTKQL-----

ApSRP-11 -------------------------------------CNE------LSFKMWTAVT----

DmSRP-26 ------------------------------------LCNE------LAFSYWRAIT----

ApSRP-10 --------------------------------------------------LLKAT-----

DmSRP-12 --MIHWRLLSALLVGLAIALTLPVDGELLARSPASVSSNR------FGLRLTTKL-----

TuSRP-11 ---------------------------------LAYSTNQ------FGFDLLRAM-----

DmSRP-8 -------------------------MYVLLLILLGISRYRAQKNSQLPDELYAAI-----

DmSRP-17 ------TAPTAFQSGVSHIQSMRSNFDTDVLVSISQGVQD------FALDLLQRIS----

DmSRP-9 ----------------------------------------------MKLGFLGLF-----

DmSRP-10 -MKLGFLGLFGMVLSLRAHICANTVDTKSLLQKMTDARLQ------FALNLLQME-----

DmSRP-3 ---------------------------MSEAANPTPYDCH------IGAGIYHSI-----

ApSRP-8 ------------------------------------------------------------

DpSRP-5 ---------------------------------FSRSHFA------FSLDLYSALAGQSP

ApSRP-3b ---------------------------------------------YFDRELFQEL-----

TuSRP-8 -----------------------------------QSHMQ------FTFDLMSSIVMTEA

DmSRP-19 ---------------MAVIISCLLLLLATVSQSKTVGYDAAADRNLLAADLYNAV-----

SmSRP-7 ------------------------------------CNNK------FALQLFKRI-----

TuSRP-1 -------------------------------LNIVASNNQ------FGFKLFKAL-----

ApSRP-3a -------------------------------------FHA------FNWRLCKAL-----

DmSRP-25 ------------------------------VPFRSDSHDP------FSWHLLKTV-----

DmSRP-2 ----------ISGTSVKPSNLPAAYSNGYVDLATSDRIAN------SVLNFANIL-----

TuSRP-2 ---------------------------------LALPINQ------FSLSFYRSV-----

TuSRP-3 ------------------------------------PSND------FGLRFLEQI-----

TuSRP-14 ------------------------------------SSID------FGLNFLKQN-----

TuSRP-12 ------------------------------------SSID------FGLNFLKRI-----

TuSRP-5 ------------------------------------PSID------FGLNFLKRI-----

TuSRP-6 -------------------------------------SID------FGLNFLKQI-----

TuSRP-15 ------------------------------------SSID------FGLNFLKQS-----

TuSRP-13 -------------------------------------SID------LGLNFLKKV-----

TuSRP-16 -------------------------------------SID------LGLDFLKKV-----

TuSRP-10 -----------------------------------NPSIE------FGLKFLKKI-----

TuSRP-7 -------------------------------------SID------FGLKFLKKV-----

TuSRP-9 ---------------------------------------------GFGLKFLKKI-----

DpSRP-3 ------------------------------------------------------------

DmSRP-11 MHTFSLVLLALLPVVTIAALDKPELSFLNEFSQIFKGERD------FSLALMKQI-----

DmSRP-22 -----------------------DAGLLDQRLNLYKGQQN------FAVSMLNVI-----

DpSRP-1 --------------------------------------MQ------FSLNFFKKVFAATQ

TuSRP-4 ------------------------------------GLHK------FSIDILRSL-----

SmSRP-1 --------------------------------GLLNGSTD------FGFNVFTRL--AP-

ApSRP-1 ----------------------------------SLANHD------FSFSLYKEL-----

ApSRP-13 ------------------------------YESLSLANHD------FSFSLYKEL-----

ApSRP-5 -----------------------------------STIHN------FSFSMYKEV-----

ApSRP-14 ---ELDDKVFKSINEINYLSDNKMKPLTTNLEILRSANHN------FSFSVYKEV-----

ApSRP-15 ------------------------------LEALRSANHD------FSFSLYKEV-----

ApSRP-2 -----------------------------------CANHD------FSFSLYREV-----

ApSRP-6 ------------------------------------LTSN------YSFSLFKEL-----

ApSRP-12 -----------------------------TNFRYTSLTSN------YSFTLFKEL-----

DmSRP-1 -------------------------PPPNRPPPVFSYMDR------FSSELFKEI-----

DmSRP-20 -----------------MNHWLSIILLGVWISAPEGLGNTIKDRNLFATELFQTL-----

DmSRP-5 ------------------------------MSEPQEGRNQ------FARNLIDVITKDAL

DmSRP-7 -----------------------MASGGTTAPSLSASPIV------FARNLFRAL-----

SmSRP-2 ----------------------------------DESGFS------FIYDLYDRV-----

SmSRP-5 -----------------------------------ISNFK------FSLDVIRNS-----

SmSRP-8 --------------------------------QAAHANTQ------FGLELLKKV-----

SmSRP-6 -----------------------------------KGNIH------FVLKFYKQCV----

DpSRP-4 ------------------------------------------------------------

DpSRP-2 --------------------------------KAAAALQN------FSVSLFQAV-----

SmSRP-3 ------------------------------------KGLE------FTLDLYKQT-----

SmSRP-4b ------------------------------------AEIE------FTLNLYKQC-----

DmSRP-4 ----------------------------MKDEEFAQGLEQ------FALCLHDHL-----

DmSRP-18 -----------------PVHTADVTMADAAHQEFARRLAL------FSINVYGKL-----

DmSRP-21 -----------------------------FNLEFARGGAR------FTSELFQLL-----

DmSRP-6 ------------------------MANTLNYSKSPAGEAQ------FASQLFGQL-----

DmSRP-16 ------------------------MKYLYLLLLATSVESG------FWEDFYRIL-----

DmSRP-23 -------------------------------LLATSVSCR------FTDDLYQLL-----

DmSRP-24 -------------------------------LWVTSVACQ------TSKEIYQLL-----

DmSRP-13 ------------------------MKYLCWILVTTSVLGQ------FTKQLYRSF-----

DmSRP-15 -------------------------------------MDK--------------------

DmSRP-14 SKGR---LARNFVYSPIAIRQALGLLYLSKD--NVTDQQLESALQ--LT----GLNQEE-

ApSRP-11 GKGQI--ASRSLVLSPFELTAMLAMVFLGAR--GSTSGQMNDVLR--LD-DMVTFNPHQ-

DmSRP-26 SEKIS--SARSLVISPFALTSMLSMVFLGAR--GSTSGEMNEILK--LD-DMVTFNPHL-

ApSRP-10 SD-----DNFNYVVSPFASSVILALAAEGAD--SETKSQLVATLGGELP-----------

DmSRP-12 GLTQ---PDANVVVSPLLIQAALSLLYAESS--SEYGSQLRQALE--LT---HASHPKL-

TuSRP-11 DK-----SESSTAFCPICISSSLTMMLMGSQ--GHTSTALRHALY--LW----GMQTSE-

DmSRP-8 VN-SF--SNRNIMFSTEMIRSSMLFIYVGVE--EDESEQIRKAMH--YRGTHLSEYKPK-

DmSRP-17 VEVEK--ANKDFMISPFSVWSLLVLLYEGSE--GETRNQLKKSLR--IN-----VEDEK-

DmSRP-9 GM------------------VLMIMFYEGAE--GYTVNELRDVLG--IY-----VDYPT-

DmSRP-10 STHL---NLENFAMTPFSTWSLMIMFYEGAE--GTTLKQIRDVLA--IY-----VDYPT-

DmSRP-3 AT-SF--AEQNVVVSPLLLEATLSLLFLGSD--GATAEELQKQLR--LK--QRFASNAK-

ApSRP-8 SHGSN--HDHNIVISPICIAAAMSLVLLGAH--GETKTEVGKLFG--YDEITLSHLADN-

DpSRP-5 ANSEG--EAGNLLFSPYSVSTALSMIFLGAGAGSTTSLQLRSALH--LN----NFSFSD-

ApSRP-3b SL-NQ--PDSNVVVSPASIKTMLTLLSEGAR--GDTLDQLNRVLR--LP---------T-

TuSRP-8 YNPNSK-LLQSIVFSPLSIQSILMMVHLGVK--GRTRAEIASALH--LDTFLTSNGINNV

DmSRP-19 AA-DH--LNENVVISPATIQSSMALAFVGAK--GQTASELQQGLR--LG----PGDADA-

SmSRP-7 CT-----HCSNILVSPLSVSSVLAMLLVGAR--GQTETQLKQILH--LQEFSLTEDEDEN

TuSRP-1 NK-FH--GNENILISPLSLFSTLVTVYAGSS--ASTEDEMISLLQ--LR----TMTEAQ-

ApSRP-3a HDV----EKNNAVISSISIKLVLLMLYEGAL--GNTAKQIEQVVG--IS-----GHKQN-

DmSRP-25 LQNET--ADKNVIISPFSVKLVLALLAEAAG--AGTQTQVELANT--QT---DIRSQNN-

DmSRP-2 GQHLA--NGKTQIYSPLSIVHSLALLLLGAK--GRSYEELSTVFD--IP------DTSR-

TuSRP-2 HN-----LSENVFFSPLSISMMYSMLLRGAS--GLTAQQIINTFQYPLS----FKTSDE-

TuSRP-3 NS-NG--SPKNVLFSPLSAIIAYGMLLEGAT--GETEQQIKTVLQ--LS--SIGNQTSD-

TuSRP-14 SSSQC--DSTNVILSPLSVIIAYCMALDGAA--GETEQQIKKVFN--LD--KINGQSED-

TuSRP-12 SS-QC--DSKNVLLSPLSVITAYCMVLDGAA--GKTEQQIKKVLN--LD--KINDQSED-

TuSRP-5 SS-QC--DSKNILLSPLSVITAYCMVLDGAA--GKTEQQIKKVLN--LD--KINDQSED-

TuSRP-6 SS-QC--DSKNILLSPLSVITGYCMVLDGAA--GETEQQIKKVLN--LD--KINDQSED-

TuSRP-15 SSSQC--DSKNILLSPLSVITAYCMVLDGAA--GETEQQIKKVLN--LD--KINDQSED-

TuSRP-13 GS-----DSTNLLLSPLSVIMAYGMALEGAA--GETAKQIRTVLN--LD--KVDSQQGD-

TuSRP-16 GS-----DSTNLLLSPLSVIIAYGMALEGAA--GETEKQIRTGLN--LD--KVDSQQED-

TuSRP-10 NS-AS--DSTNLLLSPLSVIIAYCMVLEGAA--GETEKQIKEVLD--LD--EIDNQHGG-

TuSRP-7 NS-AS--DSTNLLLSPLSVIIAYCMVLEGAA--GETEKQIKEVLD--LD--KIDNQHGG-

TuSRP-9 NS-AS--DSTNLLLSPLSVIIAYCMVLEGAA--GETEKQIKEVLN--LD--EIDNQHGG-

DpSRP-3 ND-----CATNTVFSPLSIASTLTMLLMGSS--GNSYIQLRSALG--YH---NDANDVD-

DmSRP-11 RE-IY--PSGNLFFSPFSTYNALLLAYFSSS--EQTERELAQALN--LG---WALNKQQ-

DmSRP-22 RQ-ST--PNENVFFSPYSTYHALLLAYFGSS--GDTEKELAKVLH--LD---WADSKEV-

DpSRP-1 SDVQSKSASENLFFSPMSIYSALLLAYFGAN--NRTEDQLTEILG--LQ----NMDKVG-

TuSRP-4 HNFESKDSSPGLILSPFSIWSALLVSYMGAR--HETDRELRSVLG--LN----NVPKHA-

SmSRP-1 NA-RH--SESNMAFSPFSIWSALLMTYLGAR--GRTEDELNYLLG--LK----NASKAD-

ApSRP-1 AK--T--ENGNIFFSPFSIHVIMFMASMGAA--SKTFDEMINTIH--LN------ETTH-

ApSRP-13 AK--T--DEGNIFFSPFSIHVIMFMASIGAA--SKTFDEIINTIH--LN------KTTH-

ApSRP-5 SK--T--ETGNIFFSPFGIHLIMFMASTGAA--SNTFDEMVATIH--LN--ETSWKTDQ-

ApSRP-14 AK--T--ETGNIFYSPFGIHLIMFMASTGAA--SKTFDEMVATLH--LN------ETSY-

ApSRP-15 AK--T--ETGNIFYSPFSIHVIMFIASIGAV--AKTFDEMVATIH--LN------ETTY-

ApSRP-2 AK--T--ETGNIFYSPFSIHVIMFMASTGAA--SKTFDEMVATIH--LN------ETTH-

ApSRP-6 SS--S--VEGNVFVSTYSIQFLLLLLAFGSK--SKTNDQLKSVLH--LS-----KDKPP-

ApSRP-12 SH--S--VEGNVFVSTYSIQFLLVLLALGSK--SKTGDQLKTLLR--LP----QKNAEP-

DmSRP-1 IK-SQ--SQQNVVFSPFSVHALLALIYGASD--GKTFRELQKAGE--FS-----KNAMA-

DmSRP-20 AT-DR--QDENVIISPVSIQLALGLAYYGAE--GRTAAELQKTLH--AS---AKESKDG-

DmSRP-5 QQSKD--PHINTVFSPASVQSALTLAFMGAS--GSTAEELRNGLQ--LG----PGDRHH-

DmSRP-7 ND-EV--PPVNMMVSPAGARSAMTLVFMGAG--GKSADELRSKLI--LG----VSNKSE-

SmSRP-2 SV-ES--NDGNVLLSPFSVASVLALTLEGAR--GDTASQMLRVLH--WN----TLDTPG-

SmSRP-5 VS-----KEENVLFSPFGASVALGVTSLAAQ--GKTLDEIQAALN--LK---TGNGNVS-

SmSRP-8 SV-----GNKNVFFSPYSISAAIAMTSLGAA--GNTLTEIDHTFH--FDTVNTNSDPKA-

SmSRP-6 ND-----STENALLSPLSVSMALAMVSIGAR--GDTAARLREVLC--LG-----VEDVN-

DpSRP-4 -------KYDNLVLSPSSISLVLAMALIGAQ--GNTAKQIKEAFH--VT----NQNDET-

DpSRP-2 GKHHS--PTENVFISPFSVAAVLSMVGVGAR--GNTAVQLKKSMG--LT--NYVAENGN-

SmSRP-3 AVG----NTENIFVSPLSISVALAMTLAGAR--ENTAKEMKDVLK--LG--ASFSTDLE-

SmSRP-4b AAVST--STENIFISPFSISLALCMTLAGAR--ENTAKQMKDVLK--LG--ASFNTDLE-

DmSRP-4 CR-AS--AGLNIIYSPLSIHISAAMLRMGTSEGSATAKEMDEGLR--FG----GLEAQQ-

DmSRP-18 SG-QK--PGENIVFSPFSIQTCAAMARLGAE--NETATQLDQGLG--LA----SSDPEQ-

DmSRP-21 SAGG---LKENVVFSPFSIQTCIALAFAGSQ--GETADEIAKALH--FV----SNFPPE-

DmSRP-6 AK-SQ--SGRNIVFSPSSIRTGLALAYLGAE--GSTADELKLGLG--LE----GAGKTE-

DmSRP-16 AS-QN--AKRNLIYSPISAEIIMSMVYMASG--GKTFEELRNVLK--FS-----ENKTL-

DmSRP-23 AK-EN--ADKNLITSPLSVEIALSLAYMGAR--GKTAQEMRDVLK--LP-----DDKKE-

DmSRP-24 SK-SH--TNQNLVVSPVSIETILSMVFMGAE--GSTAKELQSALG--LP----SEDKEA-

DmSRP-13 LQ-DN--KQYNIIASPLCVEIGMSMILMGAD--GNTANELRTALN--LP-----EDKKN-

DmSRP-15 -------AESNFIASPLCIEIGISMILMGAK--GTTAEELRSVLD--LP-----VDVTE-

:

DmSRP-14 -----------IISLFKEA----------------------------------------R

ApSRP-11 -----------VLRNITHS-----IT--------------------------------NI

DmSRP-26 -----------IFKNITNS-----VE--------------------------------QA

ApSRP-10 -----------DKNSYKEV-----LS---------------------------TIKGYSL

DmSRP-12 -----------AVQDFETL-----LT------------------------------DLKQ

TuSRP-11 -----------INLAYHDM-----MTHLGVNVPNSVHYRNLGPYGPSPASDYRVSIADNE

DmSRP-8 -----------TQKIFAMS-----VK----------------------------------

DmSRP-17 -----------LRGAYKV------WS------------------------------SFLN

DmSRP-9 -----------LRRWYEDVRAYHYLN----------------------------------

DmSRP-10 -----------LRRWYEDVRAYHYLN----------------------------------

DmSRP-3 -----------MANFYAAE-----LG--------------------------------NI

ApSRP-8 -----------NNQNYKDLGRL--LN----------------------------YFQTNS

DpSRP-5 -----------VHDSYKTV-----IN------------------------------KLSD

ApSRP-3b -----------DQSTFHNV-----LH----------------------------ANQLSM

TuSRP-8 NGSFIR-----THQIFGES-----VN------------------------------SLLD

DmSRP-19 -----------VSQRSGSY---------------------------------------QQ

SmSRP-7 ANDDNANGNSQIHLAYRHL-----LN--------------------------------SI

TuSRP-1 -----------IQTAFRDV-----LH-----------------------------SLLND

ApSRP-3a -----------IRERYSQK-----LQ------------------------------SLQS

DmSRP-25 -----------VREFYRKT-----LN------------------------------SFKK

DmSRP-2 -----------LHEQFGLM-----LQ---DLQQPTREAISAGRPLTDWRASSAMRSNRRA

TuSRP-2 -----------IHAAFKELVFDYKMI------------------------------EIRS

TuSRP-3 -----------ISKASKKL-----LE--------------------------AYKLVEDG

TuSRP-14 -----------ISGLIKQV-----MNNRFFRLPTQESIRDSRSVFSLTDFGSYRIPATSR

TuSRP-12 -----------ISQLIKQV-----IE--------------------------SYRIPATA

TuSRP-5 -----------ISGLIKQV-----IE--------------------------SYRIPATA

TuSRP-6 -----------ISGLIKQV-----IE--------------------------SYLKPATA

TuSRP-15 -----------ISGLIKQV-----IE--------------------------SYLKPATA

TuSRP-13 -----------VSKIIKQH-----ME-------------------------KYLKNKNST

TuSRP-16 -----------ISKIIKQL-----ME--------------------------KYLKNKNS

TuSRP-10 -----------ISEIIKQVI----MH----------------------------------

TuSRP-7 -----------ISEIIKQL-----LE--------------------------------SY

TuSRP-9 -----------ISEIIKQL-----LE--------------------------------SY

DpSRP-3 -----------INGAYKFL-----ME---------------------------RVKRMDV

DmSRP-11 -----------VLVSYTLAQRQDEFR----------------------------------

DmSRP-22 -----------VRSAYI-------LE---------------------------KMNRKER

DpSRP-1 -----------AVQAYKLVKFTRQLM--------------------------------RV

TuSRP-4 -----------VGMAYQGLRFWYQLK--------------------------RNVSLVTK

SmSRP-1 -----------SGRAYKAVKYWYKLR--------------------------------SE

ApSRP-1 -----------SMEGYRTL-----LE--------------------------------DL

ApSRP-13 -----------SMEGYREL-----LE--------------------------------DL

ApSRP-5 -----------TLEAYRQL-----LE--------------------------------DL

ApSRP-14 -----------SMEAYRQL-----LE--------------------------------DL

ApSRP-15 -----------SLEAYRQL-----LE--------------------------------EL

ApSRP-2 -----------SLEAYEKL-----LE--------------------------------DL

ApSRP-6 -----------NFENIKSV-----IA----------------------------------

ApSRP-12 -----------NYDNIKSV-----MT--------------------------------NI

DmSRP-1 -----------VAQDFESV-----IK--------------------------------YK

DmSRP-20 -----------LAESYHNL-----LH--------------------------------SY

DmSRP-5 -----------IALNFGEF-----WR-----------------------------TSCNY

DmSRP-7 -----------VAKQHAES-----WT-----------------------------DECSC

SmSRP-2 -----------VRESIRDA-----LS------------------------------SMNR

SmSRP-5 -----------LEEEIKES-----IE--------------------------------SM

SmSRP-8 -----------IHKVYKGL-----IE------------------------------DYNK

SmSRP-6 -----------IHAALNDV-----LE--------------------------------SF

DpSRP-4 -----------IACNIGA------LN--------------------------------RP

DpSRP-2 -----------SDSVIGSL-----IQ--------------------------------SI

SmSRP-3 -----------IHEAFQDI-----IN------------------------------SLLG

SmSRP-4b -----------IHEAFQEI-----IN------------------------------SLLE

DmSRP-4 -----------VAESFGVV-----LK----------------------------------

DmSRP-18 -----------IAHSFHQV-----LA---------------------------------A

DmSRP-21 -----------VAQTFQFV-----LE----------------------------------

DmSRP-6 -----------VAEKLDQL-----LA-------------------------KGQWEKASG

DmSRP-16 -----------VANNYRSL-----LS-------------------------------DLK

DmSRP-23 -----------VAAKFKDL-----LS-------------------------------KLE

DmSRP-24 -----------VAARYGAL-----LN------------------------------DLQG

DmSRP-13 -----------VATIYDKL-----LT------------------------------KLER

DmSRP-15 -----------MAKKYERI-----MS--------------------------------NF

DmSRP-14 EKVAQEQFTMGNRIYLSPDY-NASPNITQ-LSENLG--------VEVKNMTFSG--DQSA

ApSRP-11 NNPGVATASFVREIYSHKGNGKILEFYKERVQQYYD--------GHVEEVDFNTI-GDVL

DmSRP-26 SDSDIATAAFVREIFSDRANGKILPFFKEKTQQLYA--------GHVEEVNFHVV-NDIV

ApSRP-10 DGFAQNKVVLKNFLYVYKNY-SVHESYAQLARDYYL--------TDVRSVARPD--LEMK

DmSRP-12 SAAIGCRLRLLSDLYAQQRFTFNFRNEFETLAARMG--------VGCHRLSWES--ASNA

TuSRP-11 ANTGN-DIAFLSHVYVQRDF-GINYSYHMLLQRFYK--------TAIRPLDFIDN-GEET

DmSRP-8 --KAP-VAKSLTRFYVRQNM-KMSTEYRVFMRHTEG---------RARNIAFARE----Q

DmSRP-17 ITTSTIEVATLQAIYTGKGY-PIKNNYRDAIQNYN---------VQPMEVDFYS--PDSV

DmSRP-9 -SENTKLFSLRYAYYDDVGDLELVKGYNSVVLEGVGEGNVVLREGRPRGVDF----DQGA

DmSRP-10 -SENTKLFSLRYAYYDDVGDLELVKGYNSVVLEGVGEGNVVLREGRPRGVDF----DQGA

DmSRP-3 TTDADTFLQLQNRLMLSSES-GVADDFQKIAQTYFH--------ATAECVDLEQ--TEKL

ApSRP-8 GEKLGTEVNLAKAVFVQNGY-NLTKNFIKAAEDYLN--------TKLVTVDFKSD-GEHT

DpSRP-5 PYYAE-ILVTMNGIFQQEGI-FVSEKYKRALEEFYN--------VQIQPMDFVRH-PQLA

ApSRP-3b ESSLI-DLVVVNNIFVKNKN-SISNNFKETAQDKYS--------ANITEINLLN--IEAS

TuSRP-8 DDDIIKYFSMANQIFVNKDL-TVNNNFKIALQRYHG--------ASLRSVDFN---SNGV

DmSRP-19 ALTRDNNFRLANNIYINENL-EFKGSFRDVAQRQFD--------SNIDKLDFHPPYNKRT

SmSRP-7 TETGNTGLNAANRLYIQEDV-AFDGSVSDYLAENYG--------ADVGCADFARA-GDEA

TuSRP-1 VGKKN-SLKLLNAIFIDKDY-NVSTSYVDKVRTYYN--------AYLEKVGFSTE-PTYV

ApSRP-3a HGKDDYELDIGTKLFMDVSV-QPKPDFIETISRWYN--------SSLEVVDFSK--PVNA

DmSRP-25 ENQLHETLSVRTKLFTDSFI-ETQQKFTATLKHFYD--------SEVEALDFT-N-PEAA

DmSRP-2 QRPGAHEVHLANGLFTQTGY-TLNPDYRRVIVEVYA--------SDLQIQDFEGS-PATA

TuSRP-2 WQRNF-TLKLGNLVLVDKAF-PILADYADHLLNEYH--------ASVAEEDFTAE-GYQI

TuSRP-3 TSDRNFSLAMGNLAMVNKNA-KLKEAFAQSLMTNYF--------AQATNEDFSN--GTAV

TuSRP-14 SSQEP-ILKFGNLLMANKDY-KFQEAYVQSLKTNYF--------ADAFNEDYKE--GQKI

TuSRP-12 GSRQKFILNFGNLLMANKDE-KFQEAYVQSLKTNYF--------ADAFNEDFTD--GRKI

TuSRP-5 GSRQKFILNFGNLLMANKDE-KFQKAYVQNLKTNYF--------ADAFNEDYKE--GQQI

TuSRP-6 GSRQESILKFGNLLMANKDD-KFQKAYVQSLKTNYF--------AEAFNEDFTF--GRKI

TuSRP-15 GSRQESILKFGNLLMANKDD-KFQKAYVQSLKTNYF--------AEAFNEDFTF--GRKI

TuSRP-13 SEEKKCLLEFGNLLMANKDF-RLQKTFVQNLQTNYS--------ANAFNEDFTD--GQNI

TuSRP-16 ASEKKCLLEFGNLLMANKDF-RLRKTFVQNLQTNYS--------ANAFNEDFTD--GQNI

TuSRP-10 ------ILKFGNLLVGNTIF-KLKDTFVQSLQTNYS--------ADAFNEDFNNN-GKNV

TuSRP-7 KTNKNFILEFSNLLVTNRKF-KLKDTFVRSLQTNYS--------ADAFNEDFTKN-GKNV

TuSRP-9 KTNKNFILEFDNLLVGNTKF-KLKDTFVQSLQTNYS--------ADAFNEDFTNN-GKNV

DpSRP-3 EAGSSILLSIANGLFSQKQS-RFTDDYINKAKEYYQ--------SEVNELDIIRN-PYGS

DmSRP-11 WRQSPMELSSANRIFVDRTI-NVSNKFNTLLY------------GATKELDFKND-PETG

DmSRP-22 QSKMPLEFSSADRIFFANDL-----HVTECARNRLA--------EEVQQIDFKSQ-TEES

DpSRP-1 AGLVKYDFDIANRFYFNEDE-----NIRPCIKDIFN--------EDIEMLNFAFQ-PAES

TuSRP-4 ETSKKQAYSIANKIFINDAL-----TLNDCIKQHFA--------TEAESMDFTSN-PGGA

SmSRP-1 HQINY-TLNLANSLFLERTF-----PLRDCIETYFG--------SEISRVDFQRA-PEAA

ApSRP-1 LSNNE-NLKMATGMFVDETF-NVKKSFVENSMKYLK--------SSMEKKNFKDD-PEKQ

ApSRP-13 LSDND-SLKLATGMFVDEIF-KVKKSFVENSMKYLK--------SSIEKLNFKNE-PKEQ

ApSRP-5 TSAND-NLKLATGMFVDTDF-DVKDSFVENSKKYLK--------SSMEKLDFRND-PERQ

ApSRP-14 TSANY-NLKLATGMFVDTDF-DVKDSFVENSKKYLK--------SSMEKLDFRND-PERQ

ApSRP-15 TNEND-KLKLATGTFVDTAY-NVKDSFVENSRKYLK--------SSSKKLNFKND-PERQ

ApSRP-2 TSGND-NLKLATGMFVDTAF-NVKDSFVENSKKYLK--------SSIEKLNFKND-PEQQ

ApSRP-6 KLEVPGHLTVANGIFSDKAF-SLNPEYTKNTQKYLN--------SEVRSVDFSGN-PTSG

ApSRP-12 EDPDY--FTTANAVFTDMAF-VLRNDYVDKVRVYLN--------AEVKSLDFAGN-PEKE

DmSRP-1 KHLEGADLTLATKVYYNRELGGVNHSYDEYAKFYFS--------AGTEAVDMQN--AKDT

DmSRP-20 IKSKT-VLEIANKVYTRQNL-TVSSHFREVAQKYFD--------SEVEPLDFSRE-TEAV

DmSRP-5 GDRGP-VLKSVNRLYVNDSL-ELLTEFNEIAVDFFQ--------SKAEATRFAD--SEGA

DmSRP-7 AKKGV-ALRLVTRLYVNEEE-KIRTDFNDMALEFFN--------AEAYSLNYLN--PEDS

SmSRP-2 ATRSY-VLAVANRVFLQNGY-DVLPNFRHNLRRYHL--------SDVQSLDFSR--ADSA

SmSRP-5 KNSEGGSIDFATCIFTQSSC-GIMPFFGRKAIDIYQ--------SDIQQLNFSI--KKSA

SmSRP-8 PNSNY-SLSTANRLFGSKNF-EIKPTFQNETKYYYD--------AELQQVNF----DGTA

SmSRP-6 RSSFGLNLHTLNRVYVHEEL-KLQPEFIRQLGKYYC--------SNLIRFNFGHD-AERA

DpSRP-4 TQGSGVTLSTVNRALVSDDF-RLTEFFRSTLQNQFS--------ATVENVNFS---LPST

DpSRP-2 KGDENFTLEAANQLYVAEKY-QLTDDFKQNLNDNYG--------AAGQTVDFA---VDAS

SmSRP-3 SQSSAIKLHIANRMYVHHAA-NILTDYKGTLEKHYN--------TSSNVVNFEAD-SEKI

SmSRP-4b LQSSAIKLHIANRMYVHNKT-NILHHFKSVLERYYS--------ASSSEVNFEIE-AEKA

DmSRP-4 SYEQCQVLKMANGLYVMKGL-QVDEQFGHILEQKFR--------SKPMEIDFG---SEQA

DmSRP-18 YQDSQ-ILRIANKIFVMDGY-QLRQEFDQLLSKQFL--------SAAQSVDFSKN-VQAA

DmSRP-21 KYRNSNLLRVANKLYVQEGK-QLKPAYQSAIKEQYH--------SEAESINFAL--NDAA

DmSRP-6 DEDVP-KLKYANRIFVTQRF-KLTQTYQDLVSKNFA--------AAAENVNFTQK-ADTA

DmSRP-16 RRETFIILHMANRIYVNKKY-CLVPEFNQLARKAFK--------AKAKSIRLDD--PVSA

DmSRP-23 GRESVAILSLANRIYVNNKF-KLVPEYNQMVKDSFK--------AEAEAISANN--PKIT

DmSRP-24 QEEGP-ILKLANRIYVNDQY-SLNQNYNLAVREPFK--------SEAESISLTNG-PVAA

DmSRP-13 GKKVA-ILHLANRLFVNETI-GVNKRYNKLVNKHFR--------AEAEAIKLADR-LKAA

DmSRP-15 QKHNG--LRFTNWLYVNETY-EVRQDYNTLMKSTFM--------AEGKD-------PLSQ

DmSRP-14 ASEIKK---WLNKW---IGKAGGNLF---G-KNDISQTTQIVAVQGMSYSCVWKNRETAL

ApSRP-11 RRRTNL---LVKRQ--TLGRV-VEYL---R-GSGLSLTPPFAAFSANVFQTSCESASTEG

DmSRP-26 RRRTNL---LVKRH--TMGKV-LEYL---R-TNSVWVNGPLATISANLFQTDCSHGSTTD

ApSRP-10 RAATNN---IAADDDESTADF---------------KEHALLIFNGLSLEMTW-----PK

DmSRP-12 AQDINY---AFLSR--SNFSL-GELVSAPQLESLAEHNTPFLHVSGVTFRAPWAWAFDPT

TuSRP-11 RQHINA---IVEKE--TSGKI-KDIL-----PDRQSPTTQLLLLSALYFKGSLDLNITSS

DmSRP-8 LDEVNT---FYSHE--MGEQI-GQVV---K-ESWWKPNSQGLLVNAIFFNLSWERTFNPE

DmSRP-17 IQ-INE---DTNRT--TRGLIPYTIL-----PQDVYG-AKMFLLSSLYFKGQWKFPFNKT

DmSRP-9 SIIIND---DIDKA--SHAKI-FSSY---S-RRSFNSTVTVLGITVSYFKAKWKYPFDKS

DmSRP-10 SIIIND---DIDKA--SHAKI-FSSY---S-RRSFNSTVTVLGITVSYFKAKWKYPFDKS

DmSRP-3 RRHISEQ--ILASV--GGGSW-KDIH-----VAGGSSANTLLLLLAANLQSKWFLPFSAY

ApSRP-8 KHIINQ---WVSNQ--TRGKI-NDIL-----PDIPSADTKTIIASALYFTGEWENPFFIN

DpSRP-5 VDNINS---WARNF--TKQKI-SHSLRKTAAPASVHQELGITLANGLAFRSHWLFRFDPA

ApSRP-3b VKMINK---QISDA--TQGLI-NSVI---S-KDDFDGNTELLLTNVLYFKGDWLLKFNEN

TuSRP-8 VDKIND---WVTKT--TKGAI-KKFL-----SSPISPATALVALNALSYKGDWLYKFDEQ

DmSRP-19 ADGINR---AVATK--TNGKI-TDIL---R-AELLNDRTEGVIVNGVSYSAAWQKAFRLD

SmSRP-7 RAQIND---WVSEKIGTDSWQ-QGLV---G-EGVVDLFTTLVAITATTFLGEWQFPFNEG

TuSRP-1 VKWANE---LVSWW--TQGLI-PNLL------DSLDPLTRLLLINVIYFKGVWSAPFNQA

ApSRP-3a VNSINQ---WAEIL--THGRI-QQLI---S-EAETKESTVLLLLNAIYFKGYWTTPFNKE

DmSRP-25 ADAINA---WAANI--TQGRL-QQLV---A-PDNVR-SSVMLLTNLIYFNGLWRRQFA--

DmSRP-2 RYNINA---YVAQH--TKNHI-ENII-----ASDIPQTTRMILANALYFKAFWETDFIES

TuSRP-2 MNKVNK---WVASK--TNNKM-VKLF-----NQPFDYLTKLLLINVIYFEGNWKLPFDRS

TuSRP-3 MDKLNS---WISEK--TKNKI-NKIL-----TNPPDEATILILVNTLYFKGRWEEPFPKG

TuSRP-14 VEKVNL---WVSKN--TKNKI-TSIL-----DEPPSPVDVLFLVNTIYFEGKWLEPFPQH

TuSRP-12 VEKVNS---WVSNN--TEKKI-TSIL-----NEAPDSLDGLFLVNTIYFEGEWLKSFSKK

TuSRP-5 VEKINS---WVSNN--TKNKI-TSII-----NEPPSPLDVLYLVNTIYFEGKWLMPFSKD

TuSRP-6 VEKVNS---WVSNN--TKNKI-TSIL-----NEPPSPLDVLFLVNTIYFEGEWLKSFSKD

TuSRP-15 VEKVNS---WVSNN--TKNKI-TSIL-----NEPPSPFDVLFLVNTIYFEGEWLKSFSQD

TuSRP-13 LEKVNS---WVSAA--TRNKI-STIL-----DKPPEPDAVCLLVNTIYFQANWLKPFYED

TuSRP-16 LEKVNS---WVSEA--TRNKI-STIL-----DEPPEPDAVCLLVNTIYFEANWLKPFYED

TuSRP-10 MEKVNS---WVSEN--TKNKI-ATIL-----NEPPNPDAVCLLLNAIYFEGSWLNPFPKR

TuSRP-7 LEKVNS---WVSEN--TKNKI-ATIL-----NEPPNPDAVCLLLNAIYFEGSWLNPFSKR

TuSRP-9 LEKVNS---WVSEN--TKNKI-ATIL-----NEPPKPDAVCLLLNAIYFEGSWLNPFPKR

DpSRP-3 ANVINR---WVSDK--TKGKI-TNIL------SSLPPDTQLVVANAVYFNANWADPFTPD

DmSRP-11 LKEIND---WIADK--THNQI-RDML---S-SEEITPHTMLVLANAAYMKGQWLSQFKVE

DmSRP-22 RKQIND---WIAKQ--THDQI-RNML---S-ADEITPRTRLVLANAAYLKGQWLSQFKTE

DpSRP-1 RTRINQ---WVEDI--TRNKI-KDLV---T-SDTINANTRIALVNAAYFKGQWASQFKVA

TuSRP-4 LKAINS---WIEEE--THGKI-KDLI---P-PGSVTQWTTIIIANAIYFHAKWYNQFDAS

SmSRP-1 RAAINS---WVEKE--TKNKI-RDLF---P-PGMMGSSTLIAIVNAAYFKGKWQSQFKKE

ApSRP-1 RKYLND---WVLSK--TNNKI-KDLF---P-KDSITKDTALVLANAVHFQSSWVYKFK--

ApSRP-13 RRYLNN---WVLSK--TNKKI----------KDSITNDTALILANAVHFQSTWIYSFN--

ApSRP-5 RQYLNN---WVLIQ--TNNKI-KGFF---SCKDSITQDTSLVLVNAVHFKSDWAHKCI--

ApSRP-14 RRYLNN---WVLIQ--TNNKI-KDFF---SCKDSITKDTALVLVNAVHFKSDWAHTFK--

ApSRP-15 RQYLND---WVLNE--TNNKI-KDVF---P-TDSINHDTALVLANAVYFKSAWAHQFT--

ApSRP-2 RQYLNN---WVLNE--TNNKI-KDIF---P-KDSINNDTALVLANAVHFKSAWAHQFK--

ApSRP-6 ESELNK---WVSTK--TNGKI-SGIF---K-PGEIKKETVLVLASAVHFQNLWKKQFA--

ApSRP-12 VSEINK---WAEHK--TDGKI-SNIF---E-PGTIDRDTVLVLASVAYFRNAWKNQFT--

DmSRP-1 AAKINA---WVMDT--TRNKI-RDLV---T-PTDVDPQTQALLVNAVYFQGRWEHEFATM

DmSRP-20 EQ-INR---WVKQQ--TENKI-ERVV------ESLEPDTNVALVNAIYFKARWARPFNDE

DmSRP-5 TQLIND---WVEQE--TEHKI-TNLL---Q-SDAVNNETSALLINVLYFKGKWQKPFMPE

DmSRP-7 VKKVNK---WLEKH--TFYTV-RNLF---T-PEVFNSDSSVILVNSLFFRAKWNKIFPQQ

SmSRP-2 TKAINS---WAARA--TRNRI-SELM---S-SGSVDRQTRLLLMNAVYFKGDWTTQFDAE

SmSRP-5 AFTINN---WVAKH--THDKI-MTLV---D-ENSLNPDVQVMITNAIYFRGDWKLPFNST

SmSRP-8 EGIINH---WVENQ--THDKI-KDLF---K-AGSLTPDTALVLVNAIYFKGNWNSQFKKE

SmSRP-6 RLEVNH---DIVDT--TQWKV-RDLI---G-RGQVNAYTRLLAVSSLYFKGEWGSKFKPM

DpSRP-4 LEAINK---QIEKL--TNDKI-RNLI---P-KESLGASTKLILLNAIYFKGNWLKAFDST

DpSRP-2 RTKINE---WVEEF--TQHKI-KDLL---P-EGSVNSLTKLVLVNAVYFKGNWMRKFDSS

SmSRP-3 RLEINK---WVEDQ--TQTKI-KDLI---A-PGILNDLTRLVLVNAIYFKGNWHKQFDPE

SmSRP-4b TLDINQ---WVGEQ--TRRKI-KNLI---P-PGALTACTRLVLVNAIYFKGNWCKPFDSE

DmSRP-4 ASIINK---WVESQ--TNNLI-KDII---G-PRVLTKDSRLCLVNGIHFKGEWSISFNEK

DmSRP-18 AT-INN---WVEQR--TNHLI-KDLV---P-ADVLNSESRLVLVNAIHFKGTWQHQFAKH

DmSRP-21 AQAINA---WVNAK--TQGKI-TELV---S-ADSFSDNTRLVLLNALHFKGSWAHKFSEE

DmSRP-6 KH-INS---WVEEQ--THQQI-KDLI---A-PESLDADTSAILVNAIYFKADWQSSFPDY

DmSRP-16 SAIVNS---WILNR--TRGMI-RNIV---L-PKDFNSDTSAFLVNAIYFKGQWLYNFKAD

DmSRP-23 ASIVNK---WVDTQ--TSGKI-RDLV---M-PSDV-ANLVLVILNAIYFKGQWQKKFNTE

DmSRP-24 ER-INQ---WVLDQ--TSGKI-KGMI---D-PGSMTSDVKALLVNAIYFKGQWESKFDPA

DmSRP-13 WA-IND---WVLDQ--TLDNV-KDII---I-PSDLTPDESAVMINAAFFKGYWKTRFDKM

DmSRP-15 RKASNSISFSIHRK--SHKGM-RTIS---N-DHNLQINESAVLVNTVYYSGAWKTRFSKK

.

DmSRP-14 TNRTF-TLLR---------QNKKPFVYTT----QMMY--TEAPMDFFNND----QVRGVM

ApSRP-11 RDGEM-YFVVRP-------STRQRRLVPV----PAAV--WRGGF-LAGYE-PGLDATAVC

DmSRP-26 RDGEM-FFQVHP-------TVRQRRLVPI----PAVL--YRSGF-LAGYE-PSLDATVVS

ApSRP-10 STWHK-TSMS---------WNGKV----V----KAFG--AAGNF-AIAHI-PSLECTALK

DmSRP-12 ETQSI-NFFA---------GGNRP--RLV----DAMF--GQHRY-RYAEV-PALDAQLIE

TuSRP-11 RKRNY-IAPSSSSSAYKQLSGASSNLLGVFSEDSIILEARNVRI-RYGFN-RFLNCTTIE

DmSRP-8 ATYPR-EFRV---------NATKS--VMI----PMMH--EDSKF-AFGIL-GNLKATAVL

DmSRP-17 LTREE-PFFS---------ESGEV-IGKI----PMMV--QEANF-AYVSNVEGLDGYVLE

DmSRP-9 QTKVE-QFYN---------DGGSP-AGKV----EMMV--QTGKY-AYVNNVKGLQADVLE

DmSRP-10 QTKVE-QFYN---------AGGSP-AGKV----EMMV--QTGKY-AYVNNVKGLQADVLE

DmSRP-3 RTGLY-EFH----------SGSQV--KSV----PMLF--DDDMFVKFAEL-RDLDARAIE

ApSRP-8 YTRIK-PFCYGAITGDRKSSKQNKDCIYV----QMMV--GSSEV-LFHKN-EGLEFKAIG

DpSRP-5 STFDKGLFYT---------TSKKR--FEI----PMMV--GRFKI-PVGYS-SDLECRIAE

ApSRP-3b STKNQ-CFYT---------KPSMC--VEA----NMMN--LQNQL-GYGYI-PDIKAQVIE

TuSRP-8 ETQKNSLFQL---------TNGQN--ARV----SMMVGKLPIAF-GEIND-GRLKASIIE

DmSRP-19 KTEKR-SFRT---------GSGQS--VKV----DTMW--TLQNF-NYAEV-NSLDAKVVE

SmSRP-7 ATCLY-PFNA---------GGGRK--IVI----DLMHTPSCCEF-RTAFN-DDLNCQLVE

TuSRP-1 LTNEQAVFRN---------FDQTK--SII----PMMR--SISSI-NYHCDYVNVQACVVE

ApSRP-3a LTKRG-AFYI---------NSKTA--IDV----QLMT--AYSNF-KSSTI-ESLNAKLLS

DmSRP-25 TTFQG-SFFR---------SKDDQ--SRA----EFME--QTDYF-YYTTS-EKLKAQILR

DmSRP-2 ATRPD-NFYPN--------GEGTEPVMRV----QMMA--TGGAY-PYHED-HELGCKIIG

TuSRP-2 LTAAR-TFYN---------YDGSL--GDV----QMMS--TTGSY-RYAEF-WHVNMKMLE

TuSRP-3 LTIDD-TFTN---------GDGTL--SKV----KMMT-LRDKRF-NFVHN-SDKKVKVVE

TuSRP-14 RTRDD-IFNN---------SDGTK--IKT----KMMT-LKFQDF-NYVDR-LDKQLKIVE

TuSRP-12 RTRNK-IFNN---------SDGTT--VKT----KMMT-LIDERL-NYVDR-LDKQLKIVE

TuSRP-5 CTKDD-IFHN---------SDGTT--VKT----KMMT-LSGESF-NYVNR-HDKQLKIVE

TuSRP-6 RTKDD-IFNN---------SDGTT--IKT----KMMT--MSGEFFNY----------IVE

TuSRP-15 RTKDD-IFNN---------SDGTT--IKT----KMMT-MSGEIF-NY----------IVE

TuSRP-13 STEEK-IFTN---------SDGST--TKI----KMMT-LSDDTF-NFAEC-LEKKLKIVE

TuSRP-16 STEEK-IFTN---------SDGST--TKI----KMMT-LSDDTF-NFAEC-PEKKLKIVE

TuSRP-10 CTHDK-IFNN---------SDGST--TKV----KMML--HAGRY-HFLES-PEKKLKIVE

TuSRP-7 STHDK-IFNN---------SDGST--TKV----KMML--HAGRY-NFLES-PEKKFK---

TuSRP-9 CTHDK-IFNN---------SDGST--TKV----KMML--HAGRY-NFLEC----LEKKLK

DpSRP-3 VTRRE-DFHV---------SSSEI--LTP----LTMH--THSMV-AYIEN-EELGCKMIG

DmSRP-11 ETALK-PFFI---------NEREQ--EMV----YMMH--KTGAF-KMTID-EGLQSQIIK

DmSRP-22 KTVPM-PFYT---------SPSNY--SLV----SMMQ--QKGTF-LLNVD-EQLRAHVLQ

DpSRP-1 NTRLT-SFAI---------NNKEE--GVA----NMMF--QKGRF-RHAAV-EELQANLLE

TuSRP-4 KTEIG-TFHV---------TPVES--IQI----PFMK--LTANL-MYGVS-EALRCTVLE

SmSRP-1 NTRKE-VFHV---------TPGRD--VMI----DMMH--QTNLF-YHGIS-ADLDAQVLE

ApSRP-1 DAEDD-SFYI---------TPSNK--VPV----KMMT--LVHDL-QYYHD-SDLKFAALE

ApSRP-13 DAEDD-SFYV---------TPSDK--VPV----KMMT--LVDNL-QYYHD-NDLQFAALK

ApSRP-5 YVYDG-SFYV---------TPRNK--VTV----KMMS--LIRDF-QYLHD-TVLKFKALE

ApSRP-14 HVYDD-FFYV---------TPSNK--VTV----KMMT--LTRDF-QYLHD-TVLKFKALE

ApSRP-15 RCIDG-SFYV---------TPSNE--VAV----KMMI--REHGF-QYYHD-DLLQFTALE

ApSRP-2 HVYDG-SFYV---------TPSDK--VPV----KMMY--LERDF-QYYHD-SVLNFTAIE

ApSRP-6 ETKNA-SFCL---------TATNH--IDI----KMMH--QTGHF-KYYKD-NHLKFAAVE

ApSRP-12 DTKNA-SFCL---------TPSKH--IDV----EMMH--QRGLF-RYHHD-DRYKFSAVE

DmSRP-1 DTSPY-DFQH---------TNGRI--SKV----AMMF--NDDVY-GLAEL-PELGATALE

DmSRP-20 DTRDR-EFWL---------SESRS--IQV----PTMF--ADNWY-YYADY-PELDAKAIE

DmSRP-5 TTSID-HFHV---------DRDTH--VQV----NMMY--QEDKF-RFAEL-PQLKARAVQ

DmSRP-7 LTQID-DFWI---------NPRQR--MEV----SMMR--QIGQF-RYGES-KKLKSQILQ

SmSRP-2 RTQLR-PFFP---------TPTNE--IST----PMMY--TQAEF-GYAQL-DDLQSSLLE

SmSRP-5 FTKEK-QFKN---------IKNQT--TPV----QMMR--RTTDF-QQAYD-SDTNTRVLS

SmSRP-8 NTKDE-DFHS---------ADNTV--SSV----KMMN--QKSRF-NYYHDGTDLKCKVLE

SmSRP-6 QTKVS-NFHLG--------ISGRT--IAV----DMMQ--QRAEF-RIAVC-DEVDSTIVE

DpSRP-4 KTRVR-PFYV---------SPNHK-PIPT----KMMA--SRNYF-RTAFI-KEANLRALE

DpSRP-2 LTAVE-PFYLG--------SKDKQ--KNV----NMMH--IDAEF-RTGYI-ESLDARLLE

SmSRP-3 HTNAD-YFFL---------DANKA--IMT----DLMH--LKSEF-RFAED-DNLNCKIIE

SmSRP-4b KTIAE-HFFL---------DENRA--IIT----NLMH--ETSDF-NYIKDEDTLECQVLE

DmSRP-4 ETREE-DFF----------GSDRP--TRV----RMMH--VCENF-FFAVL-PMFEATALR

DmSRP-18 LTRPD-TFHL---------DGERT--VQV----PMMS--LKERF-RYADL-PALDAMALE

DmSRP-21 RTEED-IFWV---------GEEEQ--VKI----NYMN--QKAKF-NYGFF-EDLGCTALE

DmSRP-6 ATYAS-DFVN---------HGGRK--VSV----DTMS--QEDYF-RFGEL-TELKAKVVE

DmSRP-16 QTHIA-DFYV---------SANEI--IPV----KMMT--LSASL-LSGYI-DDIDAKIIE

DmSRP-23 QTKSD--FHI---------SDQKS--VPV----QMMS--LVRPF-GVSYD-RELGANVIE

DmSRP-24 KTRAS-TFQV---------TANKS--VPV----QMMA--QMGTF-RANYF-RDLDAQVIE

DmSRP-13 NTKPK-VFYV---------SKSYQ--VNV----NMMS--QVGRF-KMR---TSTIDQIIE

DmSRP-15 DTKLK-VFHG---------DHNKK--VYV----RMMS--HVGRF-RIA---DHSYGQIIE

DmSRP-14 VPF---------------KNSDMGMLVLLPRP---RY-S---TQQILYSLD--TI-----

ApSRP-11 LGP----------------DSAVSTILVLPGQQGQVAPG-DGLARLEQRLIETSYR-RGG

DmSRP-26 FGR---------------VQNTVSTVYVMPGHQSSIS-PMDNLDRLERSLVETAFSDKQA

ApSRP-10 LPY---------------KNTDYALLVLLPKN---KDVS---LNEVLKKLK--PEN---G

DmSRP-12 VPF---------------ATADLRMLIVFPNR---PD-G---LAQLERKLA--QSD----

TuSRP-11 MPF---------------KGGLITLVAMMPHD---PY-G---LDTLLTRLS--AQV----

DmSRP-8 VPF---------------SHGDLRMLLIKPDQ---PD-G---LAALQMKLQ--AMN----

DmSRP-17 LPY--------------GTQDRLAMIVVLPKR---GF-K---LNDVANNLK--ALG----

DmSRP-9 LPF---------------GEHELVMIVLLPKS---SQ-G---VNLVLYQLK--NLG----

DmSRP-10 LPF---------------GEHELVMIVILPKP---SQ-R---VSLVLKQLK--NLG----

DmSRP-3 LPY--------------EHAEELSMLLILPNQ---RG-G---LQELEKQLH--DLD----

ApSRP-8 LPY---------------KGNQFITYFVLPDT---NI-S---LSSLTAKMN--GKT----

DpSRP-5 LPF---------------SSRRVSFFIILPDD---VDRG---ITKLEANMT--SDN----

ApSRP-3b LLY---------------KDNNFSMVILLPDE---NT-S---TVQVLKDSQ--HNS----

TuSRP-8 LPY---------------KTQRLGLFMVLPLE-DSPN-G---LFNLMRSLN--STT----

DmSRP-19 LPY---------------QNPDFSMLLLLPNR---KD-G---LRSLQQSLS--GKN----

SmSRP-7 LPY---------------KEDRFAMLLIIPSE---HE-D---LHVFEDKID--VDI----

TuSRP-1 KLY---------------SGGSISFLVITPTN---GT-D---LKPLEAALG--SQL----

ApSRP-3a LPY---------------QGNKFVMYIILPDE----N-G---LDDLINKIN--PIL----

DmSRP-25 LPY---------------KGKN-SLFVLLPYA---LN-G---IHDLVKNLE--NDE----

DmSRP-2 LPY---------------RGNLSTMYIIQPFKSSVRE-----LMALQKRLT--ADK----

TuSRP-2 LPF----------------QGDISLILILPVELTHQP-G---LTKFLNSLT--TDQ----

TuSRP-3 LPY----------------IGNISMIFIMPTE---SN-T---LANLVPTLN--ATE----

TuSRP-14 LPY----------------VGNISMILILPTE---DN-N---LKKVIDNLD--STE----

TuSRP-12 LPY----------------VGNISMILILPTE---DN-N---LKNLIDNLH--STE----

TuSRP-5 LPY----------------VGNISMILILPTE---DN-N---LKKVIDDLD--STE----

TuSRP-6 LPY----------------VGNISMILILPTQ---DN-N---LKKMIDSLD--STE----

TuSRP-15 LPY----------------VGNISMILILPTQ---DN-N---LKKMIDSLD--STE----

TuSRP-13 IPY----------------FGNISMVLILPTG---DN-N---LKKMIDNLD--STE----

TuSRP-16 IPY----------------FGNISMVLILPTG---DN-N---LKKMIDNLD--STE----

TuSRP-10 IPY----------------GENMSMILVLPIG---DN-N---LKKMVDNLD--SAE----

TuSRP-7 IPY----------------GGNMSMILVLPIG---DN-N---LKKMVDNLD--STE----

TuSRP-9 IPY----------------GGNMSMILILPIG---DN-N---LKKMVDNLD--STE----

DpSRP-3 MPY---------------KGEELGMFILLPTE---KQ-GLASLSRLEDKLT--VEK----

DmSRP-11 LPYRTIYKSKETHISTPESKSDISMIIILPNS---NKIS---LNRVISRLN--ADS----

DmSRP-22 LPYRTVFESQEKEDSSPDENSDISMVLILPPF---NSNS---LEDVLSRLN--ADS----

DpSRP-1 MPF---------------LGGDVSFFALLPKG---NN-G---LEETVSRLT--LDT----

TuSRP-4 LPY---------------ANQDFSMLILLPDA---SK-G---VDSLVRQLK--PSH----

SmSRP-1 MPF---------------ISEEVSMIFFLPLK---DF-L---VDSVVRELS--TER----

ApSRP-1 LPY---------------EHYAFKMIILLPDA---KD-G---LKELENNLS--KIN----

ApSRP-13 LPY---------------KFYAFNMIILLPDV---KD-G---LKDLENNFS--KIN----

ApSRP-5 LPY---------------KHHGFKMTILLPDD---KN-G---LKNLENNFS--KFK----

ApSRP-14 LPY---------------KHHGFKMTILLPDD---KN-G---LKNLENNFS--KFK----

ApSRP-15 LPY---------------ENHSFKMIILLPDA---KD-G---LNTLENNFS--KIN----

ApSRP-2 IPY---------------KNYAFKMIILLPDA---KD-G---LNNLENDIS--KFK----

ApSRP-6 IPY---------------KVGGYEMLIILPDK---MD-A---VKDLENVFLKKSKN----

ApSRP-12 LPY---------------KAGGFDMLVILPDR---AD-G---LNDLENAFLKDSKN----

DmSRP-1 LAY---------------KDSATSMLILLPNE---TT-G---LGKMLQQLSRPEFD----

DmSRP-20 LFF---------------ENINLTMWFILPNQ---RS-G---LQALEQKLK--GVD----

DmSRP-5 LPY---------------DYSNIHMLILLPNE---VN-G---LQELEQQLN--TVD----

DmSRP-7 LPF---------------ERSNLTMMIILPTA---ID-G---LPELEEKLG--QLD----

SmSRP-2 LPY---------------RGRHLAMYLLLPDA---PD-G---LDKMRRDLLRYPQI----

SmSRP-5 LPY---------------ADNKTSMIILLPRN---VD-G---LPKLLQSLT--PET----

SmSRP-8 MPY---------------VGKSLSMVILLPDT---ID-G---LPNLVQALT--AEK----

SmSRP-6 LPF---------------SSQRISMYVFLPKD---AS-G---LALLESKLT--PDV----

DpSRP-4 LPY---------------SGSQFSMVILLPNQ---LN-G---LTHLESSLS--PKL----

DpSRP-2 LPY---------------VGRKLSMFIVLPNK---ID-G---LPELELKMH--EAS----

SmSRP-3 LPY---------------VDEAFSMFVILPTR---VD-G---LTNLEETLN--VPD----

SmSRP-4b LPY---------------ADEALSMFVILPTE---VD-G---LDRLEKSLT--VSK----

DmSRP-4 MNY---------------SACNLAMIILLPDE---KS-N---LTSLEKKLS--DIS----

DmSRP-18 LPY---------------KDSDLSMLIVLPNT---KT-G---LPALEEKLR--LTT----

DmSRP-21 MPY---------------QDSDLSMFVLLPQE---RT-G---IYALAEKLK--TVN----

DmSRP-6 LPY---------------TGTDIVFLIILPQE---EQ-G---LAIVEEKLM--GID----

DmSRP-16 LPY---------------WNSTLSMRIILPNS---VD-G---LRKLKEK-----------

DmSRP-23 LPY---------------RNSNLSMVIFLPDK---VD-G---LPELEKKMV--GFT----

DmSRP-24 LPY---------------LNSNLSMTIFLPRE---VE-G---LSALEEKIV--GFA----

DmSRP-13 LPF---------------AYSNLSMVIVLPKD---NG-S---LTQAEATIE--SYP----

DmSRP-15 MPF---------------DNSDLSMIIGLPLH---NT-Y---LSSIEKI-----------

*

DmSRP-14 LKIKLRRS--------KKTH--------LFLPKFKVS-ESVDLNMALK-ALGIQNLFTN-

ApSRP-11 WSRVLRSL--------LPRPG----LE-LQVPRFSHR-SVLNATAALQ-KMGLRDVFSD-

DmSRP-26 WRRLLTSL--------MDRPG----ME-VQLPRFSHR-SFVNASLGLQ-KMGLRGLFKS-

ApSRP-10 IEKLTKAM--------TIKP-----SF-VTMPCFQSS-NITHLKTVLQQGTQTNSVFTE-

DmSRP-12 LHQLRSQL--------EERK-----VA-LTLPKLRVL-VHSDLKHVLE-ELGLAKLFTS-

TuSRP-11 LSDVINSL--------EVRR-----VD-VKIPRLHFETSDRNLSLSLA-NLGVAYIFKP-

DmSRP-8 ILSVARNL--------TMMD-----VF-VGIPKFKIH-SDLELSPAFE-KMGIKDIFKP-

DmSRP-17 LRPILQRLAAFRNRASEDNE-----VE-VMMPKFVTA-TDFTLKGVLI-QMGIRDLFDE-

DmSRP-9 LHRLLEKLEASK----NETD-----VE-VKLPKFDTR-SVLSLEDTVY-DAGLTDLRND-

DmSRP-10 LHRLLEELEASK----NESD-----VE-VKLPKFDTR-SVLSLEDTVY-EAGLTDLRNE-

DmSRP-3 LGALQQRM--------QMEG-----VQ-VLLPKFSID-FECSLRQPLK-QLGFEEIFAA-

ApSRP-8 IKNITRTA--------KITE-----LT-YFVPKMTLK-SLTNLRPVLQ-NLGINKMFDP-

DpSRP-5 IKALFSTL--------KDET-----VN-IRLPRFRLEQQEIELTKTLA-ALGIHDVFDN-

ApSRP-3b FSKILNSI--------DERA-----VN-LYLPRFKID-FSTKLPSVLK-KMGLTSIFSS-

TuSRP-8 FTQLIASM--------KKSK-KGDEVN-VRIPKFDIS-SKPDLTTILRYSLGLRSVFSG-

DmSRP-19 LLAEIGAM--------SQQK-----VE-VLLPKFSVT-FGLGLEGPFK-KLGVHTMFSR-

SmSRP-7 LDALVGQL--------EPSS-----IK-IALPRFRVA-CTFSLSDIIG-DMGAGLVLNP-

TuSRP-1 MYELISQL--------QENT-----VE-LGLPRFSLS-GSYDLLKPLE-YLGMKAAFSP-

ApSRP-3a LGESIKNM--------KTFS-----TK-VVLPRFSFE-YTSILGPLLQ-KLGITDMFGQ-

DmSRP-25 LKSAQWAM--------EEVK-----VK-VTLPKFHFD-YQQNLKETLR-SLGVREIFED-

DmSRP-2 IESMISRM--------YRRA-----AL-VAFPKMHLT-ESVNLKTVMQ-RMGLGGIFSA-

TuSRP-2 LNSMISSL--------TQQT-----VE-LSLPKFRLE-GRYDLNKILP-DMGLFLPFIT-

TuSRP-3 LDNLLSSM--------SQTK-----LRALSIPKFKLE-DTHKLHEILP-RMGMDLPFGD-

TuSRP-14 LSSLMESM--------SMTN-----LDTLTIPKFKLE-DKHELHNILP-RMGMTRPFEM-

TuSRP-12 LSSLMDSM--------KKTR-----LNNLTIPKFKLE-DDHELHKILP-SMGMTRPFQM-

TuSRP-5 LSSLMESM--------KKTY-----LDTLIIPKFKLE-DIHQLHNILP-KMGMTAPFQK-

TuSRP-6 LSSLMESM--------KKTR-----LDTLTIPKFKLE-DIHQLHKILP-NMGMTRPFKK-

TuSRP-15 LSSLMESM--------KKTR-----LNTLTIPKFKLE-DIHQLHKILP-NMGMTRPFKK-

TuSRP-13 LFSLIDSM--------SRTR-----LGTLTIPKFKLE-DNHQLHKILP-RMGMTRPFQM-

TuSRP-16 LSSLMDSM--------SRTR-----LDTLTIPKFKLE-DNHQLHKILP-SMGMTRPFQM-

TuSRP-10 LSRLIDSM--------TRTR-----LDSLMIPKFKLE-DKHQLHEILP-SMGMILPFDR-

TuSRP-7 LSRLIDSM--------TRTR-----LDSLMVPKFKLE-DKHQLHEILP-NMGMILPFDQ-

TuSRP-9 LSRLIDSM--------TRTR-----LDSLMIPKFKLE-DKHQLHKILP-SMGMILPFDQ-

DpSRP-3 LEHMFSRM--------EAKT-----VA-ISLPKFRIQ-QKLQLKNVLR-GLGVTDLFSP-

DmSRP-11 VKKWFERA--------LPQK-----IE-LSLPKFQFE-QRLELTPILS-LMGVNTMFTR-

DmSRP-22 LDDSLKQA--------MPRE-----IE-VSLPKFEFE-QRLELNPILA-KMGVSKMFDE-

DpSRP-1 LRNAMAST--------FPLT-----VD-VGIPKFRLE-QTLSLRNVLV-KMGLTDMFDS-

TuSRP-4 LEDVVANM--------FDDE-----IS-VVLPKFKAE-QELELSGPLY-SMGIMKLFDP-

SmSRP-1 IRWLRHEL--------RKSE-----VE-VAIPKFRVE-NNFEMTQLLT-QMGLRDLFIP-

ApSRP-1 LNDISNKM--------SQYH-----VT-VKLPRFKLE-QSLQLEDTLS-NLGCPTMFTQ-

ApSRP-13 LNDISNKM--------SKYD-----VT-VKLPRFKLE-QSLQLDGTLS-NLGCTTMFTE-

ApSRP-5 IHEISEKM--------TQNY-----VK-VKLPRFKIE-QSLELDKTLS-NLGCSTMFTP-

ApSRP-14 IHEISEKM--------TQNY-----VK-VKLPRFKII-QSLELDKTLS-NLGCPTMFTP-

ApSRP-15 LHEISKNM--------TQHY-----IR-VKLPSFKLE-QSLQLKETLS-NLGSPTMFTR-

ApSRP-2 LHDISKKM--------TQHY-----LS-VRLPRFKIE-QSLQLDETLS-NLGCPTMFSQ-

ApSRP-6 YAHLLSNM--------TIHN-----VE-LDVPKFKFE-SEMDLIKTMQ-KLGLTEIFLP-

ApSRP-12 FAYLQGNL--------TVHD-----VT-VDLPKFKFE-SDVSLIKTME-KLGCTEMFTS-

DmSRP-1 LNRVAHRL--------RRQS-----VA-VRLPKFQFE-FEQDMTEPLK-NLGVHQMFTP-

DmSRP-20 FNLLEDRW--------QWQS-----VS-VYLPKFKFE-FDTDLRPTLH-KMGISAMFSD-

DmSRP-5 LADIDAAL--------TLQD-----VE-IFLPRMCIE-YDVDLKQVLN-QLGITEVFSD-

DmSRP-7 MNEVAAKS--------LMKE-----VD-VTIPKFRIE-CTVDLKVPLQ-KMGINSVFDA-

SmSRP-2 FYSPESFI--------VPQN-----VT-VLLPKFRLE-QNVRLKPVLA-HMGIRDLFFQ-

SmSRP-5 LQRMTAGL--------TYSH-----VT-VELPKFKLE-VEYSLKSTLS-KLGIKDLFTTK

SmSRP-8 FEALLGKL--------FETQ-----VD-VKLPKFKLE-TEYSLKETLQ-QLGIHDLFS--

SmSRP-6 LTAIFAGM--------RRAR-----VT-VTLPRFRLS-SCYCLQDSLA-ALGLANLFHP-

DpSRP-4 LSLIDSKL--------NKIQ-----MD-VVIPKFKLE-FSPDLKSVLR-SVGIVDLFNT-

DpSRP-2 LDDSNVEM--------RSAK-----LH-VAIPKFKLD-ADIKLKDILI-KMGIADLFDA-

SmSRP-3 LNNLMSRM--------RKTK-----VI-LTLPKFKIE-ASLSLKDILS-AMGMKDLFSA-

SmSRP-4b LNELINGM--------RKQK-----VD-VTLPKFKIE-SALSLEEFLS-VLGMDDLFCP-

DmSRP-4 LEVVSSAM--------NLEK-----VD-VKIPSFTAE-FQQELSQVLM-LMGMNRIFSG-

DmSRP-18 LSQITQSL--------YETK-----VA-LKLPRFKAE-FQVELSEVFQ-KLGMSRMFSD-

DmSRP-21 LVDLADKL--------TVEE-----VH-VKFPKFKVD-YSLELAEKLK-QLGITKMFTD-

DmSRP-6 LNEISSQL--------RRRK-----VR-VQLPKFKFE-FDVPLQAALE-ELGIKKLFSP-

DmSRP-16 VGFIDYHL--------EKKS-----VN-VKLPKFKIE-SKAQLKGIFE-NLGILDVFKP-

DmSRP-23 -----PKL--------ININ-----VH-LRLPKFKIE-FSARLEQVLI-AMGIQDAFKT-

DmSRP-24 -----RPL--------VAKE-----VY-LKLPKFKIE-FRDELKETLE-KLGIRELFTD-

DmSRP-13 ----QIVL--------TEMD-----VH-VQLPKFKID-FRMELVETLK-SMGIQDLFNS-

DmSRP-15 LRTLSESL--------VENN-----VH-VELPKFKIK-YQTELVESLK-KLGIHLIFSN-

.* : .

DmSRP-14 -----TNAANFKQY-------------------------------NSFDADQNRVLMTID

ApSRP-11 -----Q-KADLRGVN------------------GL----------YDLYLSDMLQVNTFS

DmSRP-26 -----D-FADLRGLTGA----------------GN----------RDIFLSDMIQINTFS

ApSRP-10 -------SADLSKLSS-----------------------------DKLYLDDVVQQANLR

DmSRP-12 -------EVHLSEVFSSIL--------------SS----------SAPPLGAVVQSGLLE

TuSRP-11 -----G-YSQLYDIS------------------DY----------KWLHVSDIIHKTYLE

DmSRP-8 -------SKSFSTLLH-----------------RN----------TNFRIDGVIHVVTFE

DmSRP-17 -----N-TANLDRM--------------------S----------SGLFAKLVVHSTKII

DmSRP-9 -------FADLDKLLIAI---------------GH----------RGACLTLYHQFARIV

DmSRP-10 -------FADLGRMLIPT---------------GD----------RGAYLSLYHQFARIV

DmSRP-3 -------SANFKHLH------------------AS----------ANLPIADVLQKLRIN

ApSRP-8 -----S-KADLSNMA------------------SD----------PGSYISDILHQVEID

DpSRP-5 -----E-EADLSGISS-----------------------------EKLHLNHVIHKTFLE

ApSRP-3b -------NANLTSIFN-----------------PA----------KQVLVKDITHKVTME

TuSRP-8 -----G-EADFTSMFETTPVSQSS---------SL----------PPISLSQFTHQAVMS

DmSRP-19 -------DGDFGNMYRMF---------------------------VSHFINAVEHKANVE

SmSRP-7 -----D-SADLTGFTAS----------------NE----------EGVCLSALIHGTELR

TuSRP-1 -----K-KADLSKMG------------------NS----------KELFVKEAKHKTVLQ

ApSRP-3a -------NANLTNLGND----------------GQF---------GSLIVSNILQKAGLE

DmSRP-25 -------SASLPGLTR-----------------GADVA-------GKVKVSNILQKAGIN

DmSRP-2 -----V-QNDLSLIATNEATRTNALGGNSLQNLEAQRRAGTGGARSDLVVDDIVHKVDFT

TuSRP-2 -------NTDFFTIS------------------PS----------EGLKVTDSMHTSLIQ

TuSRP-3 -------LAQLPNIA------------------EK----------SELKVSQSIQKALLE

TuSRP-14 -------NAEFPRIIE-----------------EP----------RPLYITKSIQKAVIE

TuSRP-12 -------NAEFPKITE-----------------GS----------LPSYISQSIQKAVIE

TuSRP-5 -------DAEFPRITE-----------------EP----------LPLYISKSIQKAVIE

TuSRP-6 -----A-DAEFPRITE-----------------ES----------LPLYISQSIQKAVIE

TuSRP-15 -----A-DAEFPRITE-----------------ES----------LPLYISQSIQKAVIE

TuSRP-13 -------NAEFPRITE-----------------GS----------LPLYISKSIQKQQKV

TuSRP-16 -------NAEFPRITE-----------------GS----------LPLYISKSIQKALIE

TuSRP-10 ------MSAEFPKITE-----------------------------RLVYISQSIQKALIE

TuSRP-7 -----G-NAEFPKITE-----------------------------RSVYISQSIQKALIE

TuSRP-9 -----G-NAEFPKITE-----------------------------RLVYISQSIQKALIE

DpSRP-3 -----S-SADLSRMT------------------SK----------TGVALDNIIHQTFIE

DmSRP-11 -------NATFGDLTA-----------------DP----------ISLVIDDAQHLAKIK

DmSRP-22 ------SVATFDDLTS-----------------------------ETISIGDSKHVAKIK

DpSRP-1 -----L-AADFSGFN------------------GV----------PGLKFDEATHKAFIE

TuSRP-4 ------RFADLSGFFQPASANKNGKNKNTTHELQT----------KGITVNSVVHKVYVS

SmSRP-1 -----S-QSNLSGFS------------------DD----------RRLAVSTVRHKAFLE

ApSRP-1 -------AANFSNIV------------------EH----------GDLHVSKVLHKAYVD

ApSRP-13 -------SANFSNIV------------------EH----------GELYVSKVSHKAYVH

ApSRP-5 -----G-AANFSNIV------------------EN----------DELYVTKILHKAYID

ApSRP-14 ------GAANFSNIV------------------ED----------GKLYVTKISHKAYVD

ApSRP-15 -------AANFSNIV------------------ED----------GNIYASKVVHKAYID

ApSRP-2 -------AANFSNIV------------------ED----------GKLNVSKVLHKAYIN

ApSRP-6 -------TADFSELSSS----------------GA----------GKLKVSSMKHKTYVD

ApSRP-12 -------SADFSYISTS----------------GA----------GKLKVSDIKHKAFVN

DmSRP-1 -------NSQVTKLMD-----------------------------QPVRVSKILQKAYIN

DmSRP-20 -------AADFSNIFQD----------------SP----------IGTRITKVQHKTFID

DmSRP-5 -------KAKLDGLFT-----------------SQ----------SGQKISAARHRGYID

DmSRP-7 -----G-QADLSDLFE-----------------MK----------TPQKISEARHKVFLN

SmSRP-2 -----G-KCDLSAIS------------------NT----------KDLFVSDVVHKSYLE

SmSRP-5 LVHIFNTRANLTGIV------------------CN----------NDVYVNDVIQKSFIE

SmSRP-8 -------NSDLTGLSE-----------------------------SSVQVSGVKHKAFVN

SmSRP-6 -----G-EADLGAMAASSSTGD-----------NS----------HGMHIGGLLLKTYVD

DpSRP-4 -------DADLSKIS------------------GS----------KELFVSDAFHRTIIE

DpSRP-2 -----N-AADFSGIS------------------GE----------KDLFVSNIFHKSFID

SmSRP-3 -----R-DADLSGIT------------------GQ----------KDLFVSAVLHKAFLE

SmSRP-4b -----G-IADLSGI-------------------ND----------QKLYVSAIFHSAVLE

DmSRP-4 -------QAELGGMLQ-----------------SE----------ESLFVSQIVHKAFIE

DmSRP-18 -------QAEFGKMLQ-----------------SP----------EPLKVSAIIHKAFIE

DmSRP-21 -------QAEFSNLLE-----------------SP----------EGVFVSKVLHKATIE

DmSRP-6 -------GANLSSLYQ-----------------GS----------EPLRISEVKHKAIIE

DmSRP-16 -------SADLNGLV------------------LE----------SGAKIDKIVQKAFLK

DmSRP-23 -------SADFNDLV------------------AN----------SGAHVGGVVHKAFLE

DmSRP-24 -------KSDLSGLFA-----------------DK----------SGGKVSQVSHKAFLE

DmSRP-13 -------SSDISVLL------------------NQ----------SGTRISQVVHKAFIE

DmSRP-15 -------TSDLSGLLT-----------------NG----------TGAKINHVVHKSFIE

.

DmSRP-14 ------------V--------------GDDFDDRV-------------------------

ApSRP-11 TCGEDTIGARHHVETYPASPQRMGRAGHESGPGSDHGDGHGSDDESVRDGGRRKRNAEDL

DmSRP-26 TCGEEKISEHHHVEMYPAPPLRK-RNKDVDATDDDAFDSSERVVDFGSLVQESALGRGFY

ApSRP-10 ------------V--------------CVDGTSSSALTSS--------------------

DmSRP-12 ------------L--------------QEDGGNADDSFSF--------------------

TuSRP-11 IWENPKTFTNTNI--------------NSINTNGNTINSN--------------------

DmSRP-8 ------------F--------------QEQGIGTPSTDVG--------------------

DmSRP-17 ------------V--------------DEQGTTAGAVTEA--------------------

DmSRP-9 ------------V--------------DEEGLPNAVPQKS--------------------

DmSRP-10 ------------V--------------DEEGLPNAVPQKS--------------------

DmSRP-3 ------------L--------------NESGSGSGPELPK--------------------

ApSRP-8 ------------V--------------NEIGTVASAATVV--------------------

DpSRP-5 ------------V--------------EEDGMAESTTSGL--------------------

ApSRP-3b ------------V--------------NEKGSKAGAVTVV--------------------

TuSRP-8 ------------I--------------NENGSIAGAASAT--------------------

DmSRP-19 ------------V--------------TEAGVDQPLETGL--------------------

SmSRP-7 ------------V--------------IEKGTEYSPSTPQ--------------------

TuSRP-1 ------------V--------------TEEGTLAAGATVA--------------------

ApSRP-3a ------------V--------------NEQGSTAHAATEV--------------------

DmSRP-25 ------------V--------------NEKGTEAYAATVV--------------------

DmSRP-2 ------------V--------------NEQGTEAAASSVT--------------------

TuSRP-2 ------------I--------------SEEGSQHQTSGSV--------------------

TuSRP-3 ------------L--------------DEEGSVAAAATLI--------------------

TuSRP-14 ------------V--------------FEKGTIASAATVV--------------------

TuSRP-12 ------------V--------------FEEGTIASVSTAL--------------------

TuSRP-5 ------------V--------------FEEGTIASAATVVEGL-----------------

TuSRP-6 ------------V--------------FEKGTIATAATVV--------------------

TuSRP-15 ------------V--------------FEKGTIATAATVV--------------------

TuSRP-13 ------------L--------------N--------------------------------

TuSRP-16 ------------V--------------TEAGTVATAATQV--------------------

TuSRP-10 ------------V--------------TETGTVAAAVTKS--------------------

TuSRP-7 ------------V--------------TETGTVATAVTQL--------------------

TuSRP-9 ------------V--------------TETGTVATAVTQL--------------------

DpSRP-3 ------------V--------------TESGTEAAAATVL--------------------

DmSRP-11 ------------V--------------DEVGSTAAAATIL--------------------

DmSRP-22 ------------V--------------DEEGSTAAAATVL--------------------

DpSRP-1 ------------V--------------NEEGSEAAAATAL--------------------

TuSRP-4 ------------V--------------NEEGTEAAASTAI--------------------

SmSRP-1 ------------V--------------NEEGSEAAAATGI--------------------

ApSRP-1 ------------V--------------NEKGTEAAAATVI--------------------

ApSRP-13 ------------V--------------NEKGTEGAAVNGL--------------------

ApSRP-5 ------------V--------------DEDGTEAAAVTSL--------------------

ApSRP-14 ------------V--------------NEDGTEAAAVTSI--------------------

ApSRP-15 ------------V--------------NQYGTEAAAITKM--------------------

ApSRP-2 ------------V--------------DEFGTEAAAVTTI--------------------

ApSRP-6 ------------V--------------NEKGTEAAAVTGA--------------------

ApSRP-12 ------------V--------------DETGTEATGVTGY--------------------

DmSRP-1 ------------V--------------GEAGTEASAASYA--------------------

DmSRP-20 ------------V--------------NEIGCEAAGASYA--------------------

DmSRP-5 ------------V--------------NEAGSEAAAVSFM--------------------

DmSRP-7 ------------V--------------TEFGCEVAPEAEV--------------------

SmSRP-2 ------------V--------------NEKGTKAAAVSAI--------------------

SmSRP-5 ------------V--------------EETGTTAAAATSI--------------------

SmSRP-8 ------------T--------------DEEGTEAAAATGV--------------------

SmSRP-6 ------------V--------------NEEGSEASSAAAL--------------------

DpSRP-4 ------------V--------------NEKGTEAAAATAF--------------------

DpSRP-2 ------------V--------------NEEGSEAAAATGS--------------------

SmSRP-3 ------------V--------------NEEGSEAAAATAV--------------------

SmSRP-4b ------------V--------------NEEGSEAAAATAI--------------------

DmSRP-4 ------------I--------------NEVGTEAAAATAA--------------------

DmSRP-18 ------------V--------------NEEGTEAAAATGM--------------------

DmSRP-21 ------------V--------------NEEGTEAAAATGM--------------------

DmSRP-6 ------------V--------------NEKGTTASGATFI--------------------

DmSRP-16 ------------I--------------DEKGGEASAATGV--------------------

DmSRP-23 ------------V--------------NEEGSEAAAATAV--------------------

DmSRP-24 ------------V--------------NEEGAEAAGATSV--------------------

DmSRP-13 ------------I--------------DEEGGSAGSASAS--------------------

DmSRP-15 ------------I--------------NERGASTGEASDH--------------------

DmSRP-14 ----------------------------VYVNRGFVFVVKDKST--IYMIGRMDAV----

ApSRP-11 PLHSPYSHLPLNL-R--PRQARLPDVPRLRFDRPFLYLVRHNPTGMIIYLGRFNP-----

DmSRP-26 DDLLDPKYLELPL-PLRPRQARVPDAPRLRFDKPFLYFVRHNPTGMILFMGRFNPRLL--

ApSRP-10 -AFTSQRVIKES----------------VVMDRPFAYALYNVANGIVYAAGKLEQPVWED

DmSRP-12 -GDLFRRALP------------------LVINHPFFYAIGNGKT--LLLSGHIVDI----

TuSRP-11 -NVNNNNGKSQYQ-S--SNNIE------VVFDKPFLFFIMDNISGLILAMGKHGR-----

DmSRP-8 -NGSLTHTFNGVK-Y-------------FLATHPFAFYIIDNTS--IYFAGHVTSF----

DmSRP-17 -ALANK----ATP-P--K----------FLLNRPFQYMIVEKATGLLLFAGQVRNPKAA-

DmSRP-9 -SGKNNIK--------------------FHVNRPFAYLVLQKKHKLLIHSGVFREGEIQ-

DmSRP-10 -SGTNNIK--------------------FHINRPFAYLVLQRTHKLLIHSGVFREGEIQ-

DmSRP-3 -NATEYKPIVISN-S--SRQKF------FRADHPFFFAIRSENV--TYLMGHVVEF----

ApSRP-8 -TI-------TRG-G--QTI--------FNINKPFIFFIHHVESDTVVFWSTVYKPMPYS

DpSRP-5 -NRL------GAF-G--EKY--------FEVDHPFIFFLWDYHSGILLFIGRITTPEP--

ApSRP-3b -SVIPLSNIPQPS-P--VT---------VLIDRPFIFYIFNRATKNILFSGQVYDV----

TuSRP-8 --LVERVGLFSGP-Y-------------FEADRPFLFFLTDKQSGLILFAGIFAQPN---

DmSRP-19 -LKGLF----SRS-K--K----------FEADHPFVFAIKYKDS--IAFIGHIANYAYV-

SmSRP-7 -DSD------ESE-D--EGNE-------IVVDRPFIFILRDTKSGLILFTGCIHEPSTE-

TuSRP-1 -EIGTR----KRS-P--R----------IIIDRPFIFLIRDLKTDAILFLGRVNSL----

ApSRP-3a -ELDHRFGDPIEV-Y-------------FEVNRPFLFMIEDITMDTIVFVGQVMNPLS--

DmSRP-25 -EIENKFGGSTAIEE-------------FNVNRPFVFFIEEESTGNILFAGKVHSPTTQN

DmSRP-2 -YL-------KKS-G--PDVL-------FRGDTPFMVLVRHDPTKLVLFYGLINEPPAAA

TuSRP-2 -GSYKLPILARSR-G--PR---------FIVNHPFGFIIRDNAYGINLFLGVINKL----

TuSRP-3 -VTVLRMSYFPAE-D-------------FVADRPFLFMIRDRLTGVNLFMGQMNKMAN--

TuSRP-14 ---GPMRTMASAP-Q--RPLIE------FIADRPFMFFIRDNKSGVNLFMGQLNNMTR--

TuSRP-12 -RRSTGGVSPKRI-T-------------FIADRPFMFFIRDNKSGVNLFMGQLSNIT---

TuSRP-5 -FMLACSPFSTPLIT-------------FIADRPFMFFIRDNKSGVNLFMGQLNNMS---

TuSRP-6 -NIKLKGKTCSSF-R--MPLIT------FIADRPFMFFIRDNKSGVNLFMGQLSKMTRQQ

TuSRP-15 -NIKLKGKTCSSF-R--MPLIT------FIADRPFMFFIRDNKSGVNLFMGQLSKM----

TuSRP-13 ----------------------------FIADPPFLFFISDNLPKVNLFMGQLNNIT---

TuSRP-16 -EFMLGCSLYSPQ-P--QKVFN------FIADRPFLFSIRDNLTKVNLFMGQLNSI----

TuSRP-10 -ACTSARAPSKVI-N-------------FIADRPFLFFIRDNLTNVNLFMGQLNNMS---

TuSRP-7 -EAVLACAPPKVI-N-------------FIADRPFLFFIRDNLTKVNLFMGQLNNMS---

TuSRP-9 -EAVPCSAPSKVI-N-------------FIADRPFLFFIRDNLTRVNLFMGQLNNMS---

DpSRP-3 -NL-------SRD-G--PSKT-------FAANQPFLFIIRDIPSKAVLFFGRVVRPGDA-

DmSRP-11 -LVSRSSRQPDPT-K-------------FNCNHPFVFLIYDEKVDTILFAGVYSDPRQMQ

DmSRP-22 -FTYRSARPVEPA-K-------------FECNHPFLFVIYDRTSRSILFTGIYRDPKTIK

DpSRP-1 -VAFRM----ARP-L--DGVR-------FICDHPFMFFIYDNLSESILFMGVYRNPK---

TuSRP-4 -LIARSGRPAFPT-R-------------FVVNRPFLFLIRDTATNVILFIGIVRRPYE--

SmSRP-1 -LAWRS----ARP-L--SPTS-------FIANKPFIFMLHDNLSNVVLFLGVVNRPEIT-

ApSRP-1 -MVMYSARYPIEP-L--KKVE-------FHADRPFVVAVASRNN-DVLFMGRLSNP----

ApSRP-13 -NAIIRT---MPD-R--LPKVY------FVADHPFLVSIVSRTK-TILFMGRLSNPLK--

ApSRP-5 ---IFKKGSGLSK-D-------------FIVDHPFIFFISTRCN-FILFVGRMTKID---

ApSRP-14 -HMNAGSGRSKD----------------FIVDHPFIFFISTMCN-FILFVGRMTKID---

ApSRP-15 -EFIPRRARSYTD---------------FIVDHPFMFFISTRYN-TIIFVGRMTAIIN--

ApSRP-2 -QFQLLCARSSID---------------VTVDHPFMFFISTKCN-SIIFVGRMTKID---

ApSRP-6 -GIENYNLEYVPK-D--IKNVK------FHACHPFLFIIKKDKD--ILFMGRLSNPTA--

ApSRP-12 -SSKYKKSEYSPS-D--VKAVK------FHACHSFMFIIKKNTN--IIFMGRLSNPNP--

DmSRP-1 -KFVPLSLPPKPT-E-------------FVANRPFVFAVRTPAS--VLFIGHVEYPTPMS

DmSRP-20 -AGVPMSLPLDPK-T-------------FVADHPFAFIIRDKHA--VYFTGHIVKF----

DmSRP-5 -KIVPMMLNMNKK-L-------------FKADHPFVFYIRNPQA--VFFAGRFSNPKSG-

DmSRP-7 -QPEVLKKNPDRK-F-------------FKADRPFVFAIRDRKN--VYFVGHFVKP----

SmSRP-2 -TIRGRMMQSETI---------------FRADHPFMFFIQDKVTKTVLFIGHVKNLVDSA

SmSRP-5 -ELIPLS---KPF-E-------------FTVDHPFAFLIMDRVSEIVLFIGSVFDLP---

SmSRP-8 -HLPGLPSLVPPT-P--Q----------FFVDHSFLFAIVNNVNVLSETATSVSEVVHKA

SmSRP-6 -CVTTATIRAEPE-F--TQY--------FVADHPFLFMIRDNLSGLILFVGRLVSPTK--

DpSRP-4 -RFIARSFRRTPF-I--PK---------FVADHPFIFLIRDNRKNTIRFVGRFCKPEKN-

DpSRP-2 -TCRKKRSLNFEE-D--QIVDP------FIADHPFMWLLRDNDSGMWIFLGRYVDPI---

SmSRP-3 -VMMLRCAMTFDP-E-------------FRADHPFLVFIRDNRSGSIMFWGRVVNPNPDA

SmSRP-4b -LFVEYCMPFTVE---------------FRANHPFLVCIRDNRSGCILFLGRMVNPDLNA

DmSRP-4 -VATFRSMPARQG-P--PKV--------FHANRPFFYAIKDNTHG-LLFAGHFITTKVEQ

DmSRP-18 -AVRRKRAIMSPE-E--PIE--------FFADHPFTYVLVHQKD-LPLFWGSVVRLEENT

DmSRP-21 -IMMTRMMTFPLQ---------------FQADRPFLYVIWNKKN--ILFAGAFVKAA---

DmSRP-6 -KVSVESLTIGEEVFE------------FIADHPFFFAIKDAQN--TLFLGHVSQL----

DmSRP-16 -LTRRKKSIDNLI-Q--PPME-------FIADHPFFYVIHDNKV--IYFQGHIVEPRW--

DmSRP-23 -VFRYKSIRSPPM-D-------------FNVNHPFAYVIRDAEN--IYFQGHFVNPEL--

DmSRP-24 -AVTNRAGFSTF----------------LMADHPFAFVIRDANT--IYFQGRVVSP----

DmSRP-13 -PIRGLSDYATSVVT-------------FTVNSPFVFMIRDDDN--IYFRGRVVDPLKKS

DmSRP-15 -AESIQKKTRAST-S-------------FKVNRPFVFLIRDKHT--VYFRGRVVRLPNEL

. * :

1. **I8 Ascaris.**

ApTLI-1e -------SCPKGLF-YKACSSG-CEETC--DNYKALRSGESLCKNL-PTEMCTCPTGQ--

DpTLI-1d ----CPIECPSGME-HQQCGSG-CQSIC--GQD---KDLTFTCPMS-VNDGCYCPTGH--

ApTLI-1c -------CSRRKHLTFTECEPS-EPITC--RNM---HDPPQSTPAI-CYSGCVCKKPY--

DmTLI-3c -------CAKQPYAEFTKCAPK-EPKTC--KN----MDKYVADSSD-CLPGCVCMEGY--

DpTLI-1c ----CAIDCGTDGSVYKECNKKKCERSC--QNNKN-PHPCPPMPDL-CYPGCVCPDGL--

ApTLI-1d ------VNCPTPLV-YKECFNKVCEPTC--DSLLQ-SDPCPKLPGF-CFPGCYCPEG---

DmTLI-3d --------CPSPLV-HTDCYKRRCEPSC--DNVH--GDDCPVLPDA-CFPGCYCPEGT--

DmTLI-1 -------RCPANET-FLACGPD-CQTEC--ATL---GKPCLVRHIR-CPDGCYCNKGF--

DmTLI-5 -------FCGENAT-MVRCAGV-CPETC--AF----KSL--KCPKY-CGVNCVCKPDY--

DmTLI-2 -------DCSVNGT-QTDCPTA-CPETC--DT----KGK-PNCTLI-CGGPCVCKPGY--

DmTLI-4 --------CTANGT-QTECPVA-CPETC--EY----SGN-GPCVKM-CGAPCVCKPGY--

DmTLI-6 ----------------VKCIPV-CPKIC--ADF---LYR-QKCVPKKCERGCACPKGWLR

SmTLI-1c -------CLDADNMEPTDCLVE-CPLTC--SNF---HKP-PICQTVRCKPGCKCKDGY--

DpTLI-1b -------CRSSHNEEYTPCEPE-HQLTC--KTM---GQQVSMKKPVECRPGCQCKKGY--

SmTLI-2 -------TCPRGAV-YTNCAPS-CAQTC--FDA---PQNTELCSKP-CAPGCLCPPKT--

SmTLI-1d -------QCGEGMY-YDQCGPA-CMKTC--KNIDEHEKT-ENCEAD-PVEGCFC-IGD--

DmTLI-3a -------SCGFGRV-YQACGPN-VEPTC--DSD---LAL-PASKGA-CNEGCFCPEGT--

ApTLI-1a -------QCSGGKV-YSQCLPV-VQETCTIAAE---VKS-KNINSY-CEEGCVCPFGT--

SmTLI-1a -------SCSENKV-YQDCGSD-CQASC--------SNPIRKCKSEWCNDGCFCTKGM--

DmTLI-3b -------KCPLGQV-FDECGDG-CALSC--DDL---PSK-GSCKRE-CVEGCRCPHGE--

SmTLI-1b -------PCPSGQV-FSQCSSS-CSRSC--VDI---ASN-SKCEEQ-CVEGCNCPLGM--

DpTLI-1a ----CGVHCRGGQT-YQVCANS-CARTC--YDI---ALY-PKCRRK-CVEGCNCPEGQ--

ApTLI-1b ------VQCPAGQN-YDTCGNS-CQRTC--QDI---STI-NNCKSN-CVEGCYCPKGL--

TuTLI-2 ----KLERCSSERQEFNECGTA-CPPTC--YL----PNP-EFCTRE-CVARCFCKEGY--

TuTLI-1 WTDDIDDPCPTDRV-YNPCGSA-CPLTC--AQ----PKK-GKCNRM-CVPGCFCKPGL--

TuTLI-5a -------FTNTSKV-FTDCGSL-CPPTC--AK----PKP-GICATV-CVEGCFCPKGT--

TuTLI-3c -------DGFIPKV-NNDCGSA-CPPTC--AN----PNP--VCTKQ-CVKGAFCPEGT--

TuTLI-3a -----TEQCEKPKV-FLECGSA-CPPTC--AK----PKP-TVCTLQ-CVKGCFCPKGT--

TuTLI-9a ------EQCEKPKV-FLECGSL-CPPTC--AK----PKP-EACAAV-CVKGCFCPKGT--

TuTLI-4b ----STPSCPSPKI-YNKGGSA-RTPTC--AN----PKP-EVCTAV-CVEGCFCPEGT--

TuTLI-4c -------SCPNPKI-YNKCGSA-CAPTC--AA----PKP-EACAQV-CVEGCFCPEGT--

TuTLI-5b --------CSKSKV-FNSCGSP-CPPTC--AN----PNP-GFCGTA-CVRGCFCPKGT--

TuTLI-3b ------PWCPNSKV-YTECGSN-CPPTC--AN----PKP-GPCGSA-CVKGCFCPIGT--

TuTLI-9b --------CPNSKV-YTDCGSA-CPPTC--AN----PNP-GPCAAV-CVKGCFCPKGT--

TuTLI-5d --------CSKSKV-YNECGSL-CPPTC--AN----PKP-GPCAAV-CVKGCFCPKGT--

TuTLI-4a ----CVEKCTKPKV-FSTCGSA-CPPTC--DN----PNP--ICTLQ-CVKGCFCPKGT--

TuTLI-5c --------DYIPKV-YSTCGSA-CPPTC--AN----PNP--ICSQQ-CVEGCFCPKGT--

TuTLI-7a -------PRKVQKR-IIGCGTA-CPRTC--SN----PKI-DSCIQV-CTGNPECPEGY--

TuTLI-7b ------KPRKVEKV-MIGCGSA-CPLTC--KY----PEP-RMCIQV-CTGLPECPRGY--

TuTLI-8 --------RTATKV-WNPCGTN-CPLTG--AN----PKP-RACIKS-CKADYFCPSGY--

TuTLI-6 -------PPTAAKV-YNSCGSA-CPPTC--AN----PEP-KFCILV-CKADWFCPKDY--

*

ApTLI-1e VFNNSI--CVNENRCEPCD----

DpTLI-1d AFNQELGRCVPQDHCEPCD----

ApTLI-1c VLDSFTKECVLPNECP-------

DmTLI-3c VYDTSRLACVLPANC--------

DpTLI-1c VRHSDGK-CVKPSECRDC-----

ApTLI-1d FIKELGS-CIKPSKCRN------

DmTLI-3d --VRKGPNCVPISECK-------

DmTLI-1 ARNAAGT-CIPLRRCNEGGYGN-

DmTLI-5 VFNENLQLCILKTDCP-------

DmTLI-2 VVNRMIPACVLRSDCPK------

DmTLI-4 VINERIPACVLRSDCP-------

DmTLI-6 LKHNQGR-CITRKQCK-------

SmTLI-1c IFDSKAKTCIKPSDCPC------

DpTLI-1b IFDPTSKTCIKPSECPCH-----

SmTLI-2 VLHR-GR-CIKPHRYK-------

SmTLI-1d RVLKDGV-CVDPMECKK------

DmTLI-3a VQYKEA--CITRELCP-------

ApTLI-1a VLN-DGV-CVVKEKCPCKLR---

SmTLI-1a LLGPKGK-CYQPSDCP-------

DmTLI-3b YVNEDGE-CVPKKMCH-------

SmTLI-1b TLDSDGE-CIPVSECS-------

DpTLI-1a TLDPFGL-CIPIHECPCI-----

ApTLI-1b TLSEFGE-CIPVAECP-------

TuTLI-2 FEALDGQ-CYTEQECVAIINAP-

TuTLI-1 YENAAGN-CVTLDECY-------

TuTLI-5a IENAAGK-CV--KQCSKSKV---

TuTLI-3c FENASGD-CV--KTC--------

TuTLI-3a FLNDSGK-CV--EKCSSDGN---

TuTLI-9a FLTDSGK-CV--EKCSSDG----

TuTLI-4b LLNGSGE-CV--KNCS-------

TuTLI-4c LLNSSGE-CV--KNCSSNGDNSS

TuTLI-5b LENAYGE-CV--EQCSSNG----

TuTLI-3b FENAAGE-CV--KQCSSNGS---

TuTLI-9b LENASGE-CV--KQCSSNGS---

TuTLI-5d FENAAGE-CV--EQCLTNGSS--

TuTLI-4a LLNRSGE-CV--KNCPGSG----

TuTLI-5c IEDASGK-CV--KQC--------

TuTLI-7a FENNIGK-CVLWKDF--------

TuTLI-7b YENHLGE-CVLREDC--------

TuTLI-8 LENSIGQ-CVIEEDCDLQ-----

TuTLI-6 YLNSAGN-CVLKEYCDNV-----

*

**E. I17 WAP.**

AgWAP-1 ----------GDECPL--ASKV------------GSCSPT----CLT-DRDCADIGGK--

BmWAP-4 -------AA-GT-CPL--PSKV------------YGCSPK----CKE-DYECTH-GKV--

TcWAP-5 --------S-SN-CPT--ASRI------------DSCSPK----CKD-NSNCHG-AQV--

SmWAP-3a ---CNSPKY-GS-CPS--EGLI------------NPSFYP----CVT-DRECSG-GRL--

AmWAP-4a --------V-GR-CPA--FEEQ------------NVCPARAPT-CEN-DFQCQSAGER--

SmWAP-2c CSDPFSTVT-AS-CPT--PGN-------------MQLTQP----CLI-DQDCSS-GSK--

DpWAP-1a ------KGT-GR-CPSKIRDPDRCPPVSSATDEANKRTDE----CSS-DDDCHG-TSK--

RpWAP-2a ----------LT-CPP--PTLI------------PVCRIT----CKT-DTECAD-LENEM

CfWAP-3 --------S-GN-CPL--RNTV------------TNCTPR----CMN-DEQCPF-NQK--

AmWAP-2 ------YKE-GH-CPL--RNSV------------SKCIPR----CVS-DYQCSF-NEK--

DmWAP-2 -----GPRA-GS-CPK--IGRQSRAR--------LSCLDN----CQY-DHECPE-VQK--

SmWAP-6 ------GKR-GF-CPY--PIPEI-----------QTTGIEAIL-CSS-DRNCPG-NLK--

SmWAP-1e ------GKR-GQ-CPY--WVLSTI----------EKCDSE----CKS-DYDCAG-ESK--

SmWAP-3b ------NKPDGL-CPRVELYT-------------NPTIY-----CSS-NDQCPG-NWV--

SmWAP-5 ------RKP-GD-CPKIFVMN-------------PWCPV-----CSE-DSQCPG-IQK--

SmWAP-2a ----------FD-CPD--QDK-------------IQNGTS----CLL-DSECPG-EEK--

RpWAP-1 ------ALE-GD-CPA--LNGEISK---------NACLAET---CTS-DLDCGT-KAM--

SmWAP-1d ------PKS-GA-CPVIADDEI------------GACAEE----CKN-DVDCPG-PQK--

AgWAP-3 -----YKKY-GD-CPPKPPSKLD-----------SLCLNT----CDGLDYKCPG-VEK--

NvWAP-1a ------KRA-GS-CPA--DEP-------------EHCGSP----CSH-DLDCPG-PQK--

AmWAP-4b ------KRP-GT-CPL--EEA-------------AICGNT----CQH-DLECPG-PEK--

DmWAP-1a -----FQKP-GI-CPA--PDHSQYTERTG-----YMCGSP----CSH-DLECRN-MEK--

TcWAP-2a -----IQKT-GT-CPE--KIDAG-----------DECGQM----CSH-DLECPS-VQK--

AgWAP-2a ------EKP-GS-CPR--ANEIESTDNAG-----FLCGTP----CSH-DLECPQ-MQK--

BmWAP-2a -----LQKA-GT-CPE--PATKD-----------LDCSTP----CSH-DLECPS-MQK--

PhWAP-1 ------DKF-GY-CPDSFDNIPVFV---------QACVFF----CTD-DRDCPS-TEK--

SmWAP-2b -----TNRE-GK-CPPSTSII-------------VDGGSR----CML-DTNCPG-SQR--

SmWAP-1a CPTLIDAHP-EL-CPP--TSS-------------MNKMKM----CVS-DSTCNG-TDK--

SmWAP-4 -----SSKP-GS-CPAMGLFG-------------PFCAS-----CTA-DTQCPG-MQK--

SmWAP-7 ------PKP-GT-CPAMGLYS-------------PFCAS-----CTV-DTDCLG-IQK--

BmWAP-1 -----EHKP-GK-CPV--SDTPKWE---------AACVQA----CNS-DSQCDG-TQR--

DpWAP-1d ------IKP-GF-CPP--VRSRTFLRVLAQFAGGAACADQ----CVS-DADCAG-PTR--

ApWAP-1 ------NKP-GE-CPVGDLDSVASLD--------RPCLEQ----CNS-DDFCHA-EYK--

TcWAP-4 -----FNKP-GR-CPQNEKTLSPFD---------AVCLKT----CTQ-DNQCSN-LKK--

CfWAP-2 -------KP-GY-CPERTSMTPFE----------AACLDA----CVD-DSRCPD-LAK--

AmWAP-3 ------TKP-GY-CPDKASMTPFE----------AVCLIA----CVD-DSRCPD-LTK--

DpWAP-1e -----LPKP-GA-CPA--AAHPFG----------CPADTKAVIECSS-DSDCSG-RAK--

SmWAP-1b ------EKP-GK-CPV--DAIS------------SECGDK----CSD-DSSCMG-LLK--

DpWAP-1b -----ITKP-GS-CPA--LDGQTNS---------TDCGVP----CSN-DMECQG-ADK--

BmWAP-2b ------KKS-GQ-CPY--LVPQS-----------GACEWS----CRS-DAECSG-GER--

NvWAP-1b ------IKP-GQ-CPY--LVPSS-----------SSCEVL----CST-DQECTA-GDK--

AmWAP-4c -----ATKP-GQ-CPY--LVPSS-----------SSCELQ----CSN-DQECSA-TEK--

DpWAP-1c -----VIKA-GQ-CPY--LVPISV----------DSCDSE----CSA-DEDCDG-QLK--

TcWAP-2b -----PKKQ-GQ-CPY--LVPATS----------TSCDFE----CNS-DMACNG-TMR--

AgWAP-2b -----PKKP-GQ-CPF--LVPPGSENSES-----DSCEYE----CRT-DAHCDG-SKR--

DmWAP-1b -----PRKP-GQ-CPY--LVPPGPDNLDA-----NTCAYE----CRT-DAHCDG-ARR--

SmWAP-1c ------HKP-GI-CPQ--SD---------------VCVNDSTT-CVR-DDDCRE-DEK--

TcWAP-3 -----SEKP-GD-CPP--SLPP------------PACIISSLKLCET-DEGCFG-PMK--

BmWAP-3 ------VKK-GN-CPEFPRGP-------------WICSHT----CTG-DSDCPR-ALK--

RpWAP-2b -----GLKP-GT-CPKSPSGP-------------WICSNM----CST-DSDCHR-KDK--

CfWAP-1b ------EKP-GS-CPSVPTGR-------------WVCSST----CNS-DNDCRG-SLK--

AmWAP-1b ------EKP-GS-CPAIPKGR-------------WICSST----CSV-DSDCRS-TMK--

TcWAP-1b -----GEKQ-GS-CPVAPSGP-------------WVCSSR----CAL-DSDCRG-AKK--

TcWAP-1a ------TKS-GD-CPP--YPNV------------GICEVA----CFE-DNHCAG-HFK--

CfWAP-1a ------AKA-GS-CPP--ALPV------------QFCGRS----CYV-DAHCAG-IGK--

AmWAP-1a ------EKP-GS-CPP--PLPV------------DICSQS----CFS-DSHCLG-IGK--

** * : *

AgWAP-1 CCSN---AC---NRKSCVER----

BmWAP-4 CCSN---SC---NAKSCSEPAAY-

TcWAP-5 CCTN---IC---GTKSCTDI----

SmWAP-3a CCGS---NY---ANT---------

AmWAP-4a CCKT---AC---GTK-CVNGEL--

SmWAP-2c CCAF--TNC---SYF-CAQA----

DpWAP-1a CCTD---GC---RRL-CVMPLLTS

RpWAP-2a CCPT---AC---GGSLCQRA----

CfWAP-3 CCPN---KC---SSTSCAQSS---

AmWAP-2 CCPN---KC---GSESCVQAS---

DmWAP-2 CCPS---SC---GPM-CVEPLG--

SmWAP-6 CCPIIQRGS---GKT---------

SmWAP-1e CCSN---GC---GLS-CVDRVNKT

SmWAP-3b CCYG---KA---GKT-CSRPQL--

SmWAP-5 CCPDSGKKC-------CFA-----

SmWAP-2a CCPK--SPC---SYT-CTAPV---

RpWAP-1 CCSN---GC---VLT-CIEVD---

SmWAP-1d CCLN---GC---GAHVCRDPA---

AgWAP-3 CCEH---SC---GHS-CQSPY---

NvWAP-1a CCAS--DKC---AASVCVAPK---

AmWAP-4b CCKS--EKC---GGSVCSVPQG--

DmWAP-1a CCFT--KGC---QFN-CQQPGNVT

TcWAP-2a CCQT--QQC---GAS-CTHPKNV-

AgWAP-2a CCQS--DGC---GRN-CQQPHN--

BmWAP-2a CCDG--GEC---GRH-CVLPHN--

PhWAP-1 CCLH---SC---GQT-CKPAL---

SmWAP-2b CCAT---PL---GCLICVQPNIT-

SmWAP-1a CCFD---GC---TNI-CTQPI---

SmWAP-4 CCPA---GN---SGRNCCS-----

SmWAP-7 CCPA---GF---SGRNCC------

BmWAP-1 CCHH---GC---GST-CSEPL---

DpWAP-1d CCPG---EC---GST-CTHPVLL-

ApWAP-1 CCRH---SC---GIT-CQQPV---

TcWAP-4 CCRH---SC---GVT-CQHPE---

CfWAP-2 CCPH---DC---GIT-CMHPVG--

AmWAP-3 CCRH---DC---GVT-CMHPIG--

DpWAP-1e CCSN---GC---SST-CTSPEEN-

SmWAP-1b CCSS--NEC---GSH-CVPS----

DpWAP-1b CCPS-LGGCPAGAGQHCVPPFNF-

BmWAP-2b CCAT---GC---GTA-CTQPV---

NvWAP-1b CCST---GC---GTQ-CVSPV---

AmWAP-4c CCST---GC---GTQ-CVAPVM--

DpWAP-1c CCSN---GC---GTQ-CVEPLIK-

TcWAP-2b CCSN---GC---GTQ-CVEPLL--

AgWAP-2b CCSN---GC---GTQ-CVEPQ---

DmWAP-1b CCSN---GC---GTQ-CVDPQL--

SmWAP-1c CCPH---TC---GSS-CAKPLR--

TcWAP-3 CCKN---DC---GGAIC-LPV---

BmWAP-3 CCHN---RC---GVLTCQKPEI--

RpWAP-2b CCPN---RC---GAMVCQKP----

CfWAP-1b CCKN---RC---GALACQKPM---

AmWAP-1b CCKN---RC---GAMACQKPD---

TcWAP-1b CCRN---RC---GAMACTKPE---

TcWAP-1a CCRT---AC---GGTFCTAPV---

CfWAP-1a CCPT---RC---GGSICSMP----

AmWAP-1a CCPT---NC---GGFVCTKPV---

**

### **F. I19 Pacifastin.**

TcPac-1a -TEQC-KVGDTKF--KD--CNFCKCTNGA----FECTEKKCPDRGKRGVP---

TcPac-1d -EAEC-NNGDTKK--VD--CNSCRCT-NG---LWSCTKKVCLERKTRNT----

NvPac-9a ---GC--PAKEFY--LD--CNMCNCGDSN---EPACTYKACPQPP--------

NvPac-7b ---QC--PSKSFY--ND--CNMCVCGPDD--ASAACTMMMCMPGETQQPS---

NvPac-5b --LQC-VPGSELI--HR--CNQCFCTDSG--TAMMCFKMGCGA----------

AgPac-1b -GQVC-SPNEIKM--KD--CNRCRCANNG--IGWFCTRRACPQRAKRSEPAPE

BmPac-1a --ITC-KPNQEFK--SD--CNLCKCSQSG--HSYTCTHNECLEGDTGTDADV-

RpPac-2a -TKSC-EVGTTWK--EE--CHSCFCTKEG---KVSCSKEACPPQLVPK-----

CfPac-1 --RKC-VQGKYYF--DG--CNKCFCGYNG---IGACTRRFCDPSVTIPP----

NvPac-11 --KQC-VPGKSYF--DG--CNTCFCSEAH---SVQCTRRLCPDPWKR------

RpPac-5c --QEC-IPGQITPANDG--CNFCICNKDG--QIGGCTKKLCLNGIEKAPE---

RpPac-3c -CPSC-EPGTSVTAPDG--CNSCFCQDDG--TIGGCTKMACPPKSC-------

RpPac-3b ---EC-IPGTKVPSGDG--CNDCTCTDEG--NIGPCTLKACPGI---------

AmPac-1 ---MC-VPGKSFF--DG--CNTCTCTDDG---NFICTMTACEDYDPETDTSV-

NvPac-5c --EKC-NPGMIFA--SD--CNVCICSKNG---KGVCTTFSCDTTYRF------

BmPac-3b --GYC-EPQHVYK--KD--CNVCKCQSNG--QIMTCTTRVCNSLSV-------

RpPac-3a --EQCGNVGEQFPSEDG--CNTCSCDEGG---AVVCTEKSCLLQ---------

BmPac-3a ---AC-LPNSYAI--ID--CNICYCNSNGEIDEERCTRNICDPREDSRRSS--

BmPac-2a -PKEC-KPNETFQ--IG--CNRCRCNSEG--TLYSCTRIGCLESEEKNHT---

RpPac-2c --AGC-VEGEVWK--ED--CHTCHCTM-G--RKA-CTRELCLSES--------

RpPac-2d ---NCPPNQQTWE--ED--CNQCYCER-G--KKV-CTKALCPDQG--------

RpPac-2b --KDC-KEGESWQ--ED--CNKCYCSHDG---KPVCTRMLCPSVL--------

NvPac-9b --KSC-PAGQHFY--WK--CNDCSCEENG--REASCTRNFCPDFG--------

NvPac-7c ----C-PAGEFFH--DK--CNVCHCSANG--FSAACTLMGCPSEDTTQPR---

NvPac-8b ---KC--PAQQFY--DD--CNRCVCSADG--HSAACTRLACPPHRV-------

NvPac-10 --NEC-PPNESFM--DK--CNYCRCGPEG--KDAACTKMNCP-----------

NvPac-12 -TNKC-PANQPFK--WN--CNYCTCGPEG--KDASCTRMACPQH---------

PhPac-2 ----C-IPGSIFK--KG--CEMCSCSPDG--QILSCVPISCKNLK--------

NvPac-3a ---FC-TPGSYFK--KD--CNMCSCSMDG--KTAACTDMLCPNEMKNY-----

NvPac-2a -IFSC-LPGSVFL--QD--CNACTCSNDG--LSAACTDMACPGDLN-------

NvPac-1b -EFHC-TPGSNFH--QD--CNSCICLKDG--QSAMCTGIACPTKVKRDLE---

NvPac-1a --FHC-TPGSTFQ--MD--CNSCTCSNDG--KTAMCTGIACIQENKSDVT---

NvPac-7a -TGKC-TPGQVFF--MS--CNLCKCSSDG--NYAACTFMQCFDFNF-------

BmPac-2d --FRC-NPGEQFT--RD--CNDCTCSADG--KSVFCTLRLCDQDITPH-----

RpPac-6 -CRIC-LPGKHFM--PDGDCNICKCSDDG-MSALGCTNEKCTEVRSG------

NvPac-4b ---YC-EPGRMFS--PD-NCNLCKCSNDG--TKAVCTMKLCEARKARSAN---

NvPac-4c ---YC-EAGRMFS--PD-NCNLCKCSNDG--TKAMCTQKLCQETEVT------

NvPac-3b ---YC-TPGKMFS--PD-NCNICKCSADG--LKAMCTLKLCSDD---------

BmPac-2b -VKTC-QPGQEFR--LD--CNKCLCDKEG--KDFSCTRMDCNALNS-------

NvPac-5a --ENC-FPGAVFQ--DD--CNGCICGSDG---KATCTNMDCNML---------

BmPac-4b -GREC-APGSSWS--NQ--CNSCRCNADG---YGICSDEACTEHII-------

BmPac-4d --REC-APGSTWS--NQ--CNSCRCNADG---YAICSDEACAEHINEPKKD--

BmPac-4f --RDC-APGSTWS--NQ--CNSCRCNADG---YAICSDEACAEHIDEP-----

BmPac-4g ---DC-APKTMWK--NE--CNTCWCTSDG---KPMCTKMGCISYNNFG-----

BmPac-4e ----C-VPNTTWK--NE--CYTCWCTSDG---KPMCTRVECITNNTPKKSEL-

BmPac-4c --KEC-APKTMWK--NE--CNTCWCTSDG---KPMCTRMECITNNTPEKSE--

RpPac-4 ---RC-APGVTLDAGDG--CNKCICSEKG--VVADCTRMACPGVVQSEV----

RpPac-1b -GKNC-EIGTTVK--LD--CNICHCTAMG----LACTRRLCHGQEL-------

BmPac-2c --ATC-VPGSVYN--QG--CNVCRCTDEG--RHATCTLMRCPQEKEETH----

NvPac-4a --PLC-EPGERFKL-DD--CSSCICNAAG--TTAECTLGFCDNFKTRMA----

TcPac-1b ---PC-APNDYFK--ID--CNTCYCNIEK--TGYLCTENLCPLTEP-------

NvPac-1d --HKC-KPRHLFK--KD--CNHCVCNAGG--ETAQCTVLDCSKLDL-------

RpPac-5b --NNC-PPGKKFLAEDG--CSWCICGPEG--TSPVCTLTLCPPEKVF------

AgPac-1d PGFSC-TPRSSFK--YQ--CNTCLCSDDG--KMAGCTFKFCVPGEW-------

BmPac-1b -HSVC-KPRNSFY--VS--CNICRCNDFG--TDYACTNKLCPLPA--------

TcPac-2 ---IC-KPLSKFK--ID--CNTCRCSGDG--RQYSCTEMKCPPLG--------

NvPac-6b --MQC-KPKTRFK--FY--CNTCWCSEEG--TTRICTKKYCPDNIFNKDGSL-

NvPac-1c --QVC-VPKSKFN--DY--CNTCGCSDDG--SSFICTRRLCDPEVWNKDGTM-

NvPac-3g -TRVC-EPNTHFN--EY--CNTCACSADG--MNKACTMMNCDLSIWNKDGSR-

NvPac-3h --RVC-QPNTQFK--EY--CNTCACSADG--TNKACTMMDCDLDMWNKDGS--

NvPac-3f -PRVC-EPNTHFM--DY--CNICACSEDG--TTYGCTMMNCDQNVWNKDGS--

NvPac-2e ---VC-EPRSHFK--DY--CNTCACSEDG--TTYGCTMMMCDESVWNKDGTR-

NvPac-2d ---IC-KPHSNFK--DY--CNTCFCNNDG--SEFACTRMSCPPEVWNKDGSL-

NvPac-2b --KVC-EPSTVFK--VY--CNTCGCSSDG--SSFSCTRMACNQDIWNVDGSL-

NvPac-3e --KVC-QAGTKFS--DY--CNTCFCNEDG--TSFACTRMMCDKNLWNKDGS--

NvPac-3d --KVC-QAGSRFN--DY--CNTCFCNNDG--TDFACTRMHCDENIWNKDGSM-

NvPac-2c -EKVC-EPRTQFK--EY--CNTCGCADDG--LSYICTRRMCDENIWNKDGS--

NvPac-3c -KQVC-EPLTQFK--DY--CNTCFCSNDG--LSFACTRMMCDHAIWNKDGSM-

NvPac-4d --QVC-QPLSQFK--DY--CNTCTCSEDG--SSYACTRMYCDKDIWNRDGS--

NvPac-3j ---AC-TPGKSFY--SE--CNRCVCLETG--NHAFCTLMDCAAL---------

NvPac-4f ---AC-IPGRAFY--SE--CNECVCTRSG--RSAFCTLMSCPTSP--------

PhPac-1 --ERC-EPGQSFA--KE--CNTCTCPDSGLKSLAGCTLKLCL-----------

BmPac-4a ---RC-QPGTSFQ--RD--CNTCVCLDNG---LGLCSLDACRRSSTPKKF---

RpPac-1a --KQC-TPGTTWK--ED--CNTCFCSSTG---QIGCTLMACHHYQLPTK----

AgPac-1a CEEKC-EPGTTFM--ED--CNKCRCGPDG---QKACTRKMCPPNELSDDSQVR

RpPac-5a ----C-TPGKRFLSEDG--CNWCVCNRDG--SNAACTLMLCPAKRDRLSK---

PhPac-3 --DSC-TPGETFK--KL--CNDCTCPPTGHKSAATCTLLTCGEE---------

NvPac-3i --KVC-QPGKAFSP-DG--CNTCVCNEYG--TQLACTSKLCMTTLKQAY----

NvPac-4e -ERVC-QPGKAYSP-DG--CNTCVCNRYG--TGQACTSKLCLSNLKA------

NvPac-6a --QVC-TPGTYFK--TE--CNTCVCAKDG--SASICTQKQCPPGLF-------

TcPac-1c -DFSC-TPGQTFK--KD--CNTCTCTPDG--KNAVCTLKKCAEAVANA-----

AgPac-1c --KKC-TPGTTFRSDDG--CNTCFCTETG---HAACTLKACLPPG--------

NvPac-8a ----C--PAPVFS--NG--CNTCVCSKVG--VNAACTLKACLDVD--------

TcPac-1e ---VC-QPGTTFK--KD--CNTCVCNKDG--TNAACTLKACL-----------

* * * * * *

1. **I21 7B2.**

Dm7B2-1 VLMTDLLNRMDKDMQVGY---YDVGNEAAAGSKDNVDLVSRSEYARLCDGGSDCILQSGS

Nv7B2-1 ---RDLIERMGSELADAAGDNYLDERESASSGMRGLPDKEIPLEM--------PIDYEAI

Cf7B2-1 ---YKVIDQMEKELVDTA-DTYLEYPEKVKELPIEL-----------------PADYDGM

Am7B2-1 --LRELINQMGNELIDTA-DSYLEYQDKPKEIPLEL-----------------PTDYDSM

Dp7B2-1 -------------YEEAP-ESLNEPRER--------------------------------

Tu7B2-1 --EGTLSKIPRAKFQTRM-DDIL-------------------------------------

Sm7B2-1 --LREVVAKMG----EAT-NDYLELP-SDNVVTDDESDRSRVPKEFDNDRQFQP-DYDTL

Tc7B2-1 ------------EMEEEP---------------------------------LFPLDYEAL

Ap7B2-1 --ERE---PTGADYDDAA-S----------------------------------------

Ph7B2-1 --FRDVVDRMGKDLAEAA-DSYLDMPDNERLQTNGF-LSNRVIKDLENEETSDPIDYDRL

Bm7B2-1 ---REVVERMGKDFNDAA-SSYLEFPASDR----HLALMAHASKDLENE----QLDYDSL

Ag7B2-1 --LRELVDRMGKDLAEAA-DSYIDPSAMDELPASRLALMARVTKDLESE----QLDYDAL

Dm7B2-1 ASGAASHPSLRDDEFLQHSSLWGHQFISGG---------MGEGPNRYPTIV--------K

Nv7B2-1 DA-INPKASIRDQEYLQHSTLWSHQQLNNY---------KTNDRHRIKPGAQAASKNSEK

Cf7B2-1 DT-LNPNPSIRDQEYLQHSTLWSHQRNNNNNNNNNNNKNKSNDRHRIQPTGLKGIKDE-K

Am7B2-1 DT-LNPNPSIRDQEYLQHSSLWSHQHITNN--------DKVNDRQRIKPGSVKNIKN--E

Dp7B2-1 ---LGLGLSLRDQEYLKHSSLFSRQQEDDQ---------PPSGTPTGSVKVSTPSSSSGK

Tu7B2-1 ---LGREPSLRDAEYLEHSSLFGHKYVQGG---------AGEGRQLLKPDGSVENYQVIK

Sm7B2-1 DT------MIRDQEYLQHSSLWGHQYMAG------------------------------K

Tc7B2-1 GE-PNIHPSIRDQEFLEHSSLYGSQFMSGG---------AGEGKQRLRPQGTIQNIQEIK

Ap7B2-1 -DGPNAVPSIRDNEYVQHGTLWGAQYMSGG---------AGEGVQTLNPDSPTRNKNV-K

Ph7B2-1 SN--NPNPSLRDREYLQHSSLWGHQFVAGG---------AGEGKQRLKPDGSVQNQQQVK

Bm7B2-1 ID-GNPSPSLRDQEYLQHSSLWGHQYVTGG---------AGEGEQRLRPSGVVPNRQMVK

Ag7B2-1 LDGSNPNPSPRDQEYLQHSSLWGHQYVSGG---------AGEGPNRPKPQV--------K

** *:::*.:*:. : . :

Dm7B2-1 ----NDAGLPAYCNPPNPCPEGYDMETQGGSCIVDFENTAIFSREFQAAQDCTCDNEHMF

Nv7B2-1 GEKSPENQLPAYCTPPNPCPVGY---TSENHCIENFENTAAFSRDYQSAQDCMCDSEHML

Cf7B2-1 ----AENPLPAYCTPPNPCPVGY---TSENNCLTNFENVATFSRDFQNAQDCMCDTEHML

Am7B2-1 ----KENALPAYCTPPNPCPVGY---TSKNNCIVNFENTAAFSRDYQSAQDCMCDTEHML

Dp7B2-1 ----TENVLPAYCNPPNPCPIGY---TAEDGCLEEFENTAAFSREYQAAQDCMCDTEHMF

Tu7B2-1 ----SDTILPAYCDPPNPCPIGY---TTDDGCLESFENSASFSRDYQSSQKCMCDREHMF

Sm7B2-1 ----PTFVLPAYCNPPNPCPKGY---THEDGCLENFVNSAAFSRNYQAAQECMCDTEHMF

Tc7B2-1 ----SDSTLPAYCNPPNPCPVGY---TAEQGCIEKFENTASYSRRYQAAQDCMCDTEHMF

Ap7B2-1 ----TDAALPAYCNPPNPCPVGY---TDEDGCIMDFENTAAFSRDYQESQECMCDSEHMF

Ph7B2-1 ----TDETLPAYCNPPNPCPIGY---TGEDGCLEEFENTAAFSRDYQTSQDCMCDSEHMF

Bm7B2-1 ----TDAVLPAYCNPPNPCPVGY---TEDQGCISEFENTAAFSREYQLSQRCMCDGEHMF

Ag7B2-1 ----TDASLPAYCNPPNPCPVGY---TEDQGCTMDFENTAAFSREYQAAQDCMCDAEHMF

***** ****** ** * * .* * * :** :* :* * ** ***:

Dm7B2-1 DCSEQDSADVGGDKGDL-------NSAVEQYIMQMGQEN-SLNNVNSLAKK--------A

Nv7B2-1 DCP-SSADTDNNVVPGM----PITNADFDQIVERF-----QEE-----------------

Cf7B2-1 ECS--GDTGNSNSLSNV----QISDSDFDEIVEQF-----QEE-----------------

Am7B2-1 DCS--VDSTNSNNLPNM----HISNSNFNQIVDQF-----QVE-----------------

Dp7B2-1 DCPASSLNHKNDKNRAT---ANMVDTAIRKIMSDF-----QGEHKSLVSKK---------

Tu7B2-1 DCSGNTEENQLDALARSIQNEVISDSDLDDLVDRM-----QEGHK-VVAKK---------

Sm7B2-1 DCPTSTRDSEISALAQSIQNEGVMSHALDKIMEEFDVA--HNEHKNMVAKKHYQPEPREN

Tc7B2-1 ECP-NPNPGDSDDDSFN-------DLEFNQFLQHTMQMNPGLQHKNLVAKK---------

Ap7B2-1 DCA------RSNSGTKH-------SVDVDELVRTFQV---DDEHKSLVAKK---------

Ph7B2-1 ECPVRDFQSHGNDLNEE-------DLDLDRIMEELTE---GEQHKNLVAKK---------

Bm7B2-1 SCP-------SDSTSDI-------DLRF------------PEHHKNLVAKK---------

Ag7B2-1 NCP---AAAQSESNPQM-------DSDLENFIARQFH---TQEHKNLVAKK---------

.*. . .

Dm7B2-1 GYPVMPDPRLDDAVINP-FLQGDRLPIAAKKGNLLFH

Nv7B2-1 ---------------NP-FFRGEKLPIAAKKGINVG-

Cf7B2-1 ---------------NP-FFRGEKLPIAAKKGIHVI-

Am7B2-1 --------------ENP-FFRGEKLPIAAKKGLNV--

Dp7B2-1 -FF----ADKS---ENP-YLQGDKLPIAAKKGNRVVA

Tu7B2-1 -FH----ASKK---RNPDYLLGEKLPIVAKKAPHLA-

Sm7B2-1 FFHEFLKFRNEPTKVNP-YLQGEKLPIVAKKAPKF--

Tc7B2-1 -FHQYARTSGR---LNP-FLSGERLPVAAKKGNNVVF

Ap7B2-1 -FH----VKKA---FNP-YLQGEFLPIAAKKGINI--

Ph7B2-1 -FH----TKKD---VNP-YLNGEKLPVAAKKGINLSN

Bm7B2-1 -YK----PDME----NP-YLMGERLPIAAKKGFDVS-

Ag7B2-1 -FH----VKKS---YNP-FLQGEKLPVAAKKGFNVN-

** :: *: **:.***. .

### **H. I25 Cystatin.**

DpCPI-2c ----GRKL--GYTLFAPTSMAFMMQMPQDAGD---PLVMDAD-------------FRRSV

DpCPI-5a ----GTKLSYTFFAPTSFAFTMQTPQ--DTVD---PLFVDAS-------------LRNKV

DpCPI-10a ----GTKLSYTFFAPTSFAFTMQTPQ--DTVD---PLFVDAS-------------LRNKV

BmCPI-1h ----E-----------------------TVSG---ISIRRLI----------------KS

DpCPI-1b ----------GI-----------TRV--PSEELTTSLIRHAV----------------LA

NvCPI-1b ----G------------------KAL--DSK----LTSRKLA----------------VK

TcCPI-1b ----R-----AI-----------RQL-EKISPDEKGLVKNLA----------------DF

SmCPI-1 MSAIG-----GT-----------GAM--KEAT---DEIQMIC----------------KS

BmCPI-1g -----------------------QTL--DDGDYIDEELRNYY----------------AE

DpCPI-1a ---IN-----AS-----------EDV--ELKD---PTLVAIS----------------TF

DpCPI-1d ----GVKTARSL-----------SPL--SPDD---EEVKDIA----------------AF

CfCPI-1a ----G-----CP-----------YEL--NPNL---PSLLVFA----------------EQ

AmCPI-1a -----------------------PNL---------PGFISFG----------------EQ

NvCPI-1a -----------------------PSL---------PGLSAFG----------------NQ

BmCPI-1i ----L-----NS-----------ANV--SAND---LEIRELV----------------KQ

BmCPI-1c ----N-----IR-----------KTF--EIDD---YKVSEML----------------QE

TuCPI-8 ----G-----PW-----------VTV--PVDD---PIIQKYT----------------EQ

TuCPI-12 ----G-----PW-----------VPV--PVDD---PIIQKYA----------------EE

TuCPI-9 ----G-----EW-----------GRV--SMDN---PTVLELA----------------EL

TuCPI-4 ----G-----GW-----------RDV--DVDN---QTVHLLS----------------QM

TuCPI-2 ----S-----GW-----------QTS--DANS---GTIKDLA----------------QV

TuCPI-1 ----G-----GW-----------QTS--DANS---ETIKDLA----------------QV

TuCPI-3 ----G-----GW-----------GSV--DANS---ETIKDLA----------------QV

TuCPI-14 ----T-----EW-----------TPL--PSDD---PTVVKFA----------------NL

TuCPI-18 ----T-----EW-----------KPL--PVDD---PTVIKLA----------------NQ

TuCPI-13 ----T-----EW-----------TPL--PVDD---PTVIKLA----------------KQ

TuCPI-17 ----T-----EW-----------TPL--PVDD---PTVIKLA----------------KQ

TuCPI-15 ----V-----KY-----------KPL--PVDD---STVIKLA----------------NQ

TuCPI-16 ----V-----KY-----------KPL--PVDD---STVIKLA----------------NQ

TuCPI-11 ----P-----EW-----------KPL--PVDD---PTVIKLA----------------DL

TuCPI-25 ----I-----EW-----------TPL--PVDD---PTVITLA----------------DQ

TuCPI-10 ----G-----PW-----------TPL--PVDD---PTVIKLA----------------DQ

TuCPI-24 ----G-----PW-----------TPL--PVDD---PSVIKLA----------------KQ

TuCPI-21 ----S-----VW-----------KSI--PVDD---PTVNLLT----------------EK

TuCPI-6 ----G-----GW-----------NSL--STDD---STVNQLA----------------IK

TuCPI-20 ----G-----AW-----------SSN--SVDD---PLVAELA----------------AK

TuCPI-23 ----G-----AW-----------SSN--SVDD---PLVVELA----------------AK

TuCPI-22 ----G-----GW-----------KIL--PVDD---PTVVQLA----------------AK

TuCPI-5 ----G-----GW-----------SSI--SVDH---PTVIQLA----------------AK

TuCPI-19 ----G-----GW-----------SSL--SVDH---PTVIQLA----------------AK

BmCPI-1b ----N-----TI-----------RTV--PPND---PILQMMV----------------RV

TcCPI-1a ----G------------------CPFDLNTNA---EGVDELI----------------DV

BmCPI-1a ----G-----CS-----------TRV--DVDA---AGVQELA----------------SL

SmCPI-2 ---VG-----GW-----------IDI--QEDS---EEILNVA----------------DR

BmCPI-1j ----------PL-----------EAHVEFESA---EMALQLA----------------NE

TuCPI-7 ----G-----GW-----------MPK--DIDY---EPAKDNA----------------KY

TcCPI-1f ----R-----GE-----------SPV--AKDN---EYVIKYL----------------EA

TcCPI-1h ----G-----GE-----------SPV--AKDN---EYVIKYL----------------EA

TcCPI-1d ----G-----GE-----------SPV--TKDN---EYVVKYL----------------EA

AgCPI-1 ----G-----GV-----------SDD-PELNK---EEHAERI----------------GA

AgCPI-2g ----G-----SS-----------NEL--TAEELKDKSHVERI----------------RA

AgCPI-2d ----G-----AA-----------QEL--TPEEYAKEEHQTRI----------------RT

AgCPI-2e ----G-----CA-----------TPL--AANEYGNSEHQERI----------------DK

AgCPI-2f ----G-----GS-----------RSL--SQDELAAAEHLERV----------------DK

NvCPI-1c ----G------------------APTSHSKDD---PEVQRYV----------------QL

AmCPI-1b ----------------------------DINN---PTVQELA----------------NK

CfCPI-1b ----G-----GT-----------ITT--SVND---PEVQLYA----------------NK

DmCPI-1b ----G------------------RHK--PYDE---EAAKAQL----------------QK

DmCPI-2 ----G-----AP-----------KPL--DGDD-L-SKAKELL----------------DT

DmCPI-3 ----G-----GV-----------SQL--EGNS--RKEALELL----------------DA

DmCPI-4 ----G-----GI-----------SQL--EGNE--RKEALELL----------------DA

AgCPI-2c ----G-----GA-----------TPV--DVKE---PTHIERV----------------RL

TcCPI-1c ----G-----AP-----------NRI--DKNS---EKIRQFV----------------KE

TcCPI-1i ----G-----GA-----------TEI--DKKS---DKVKQYV----------------RE

TcCPI-1e ----G-----GI-----------TEV--DKDD---DEVKTFV----------------RE

TcCPI-1g ----G-----GI-----------KEV--DKNN---DEVKTFV----------------HE

ApCPI-1a ----G-----GY-----------NDV--EADS---EKIRELA----------------LF

IsCPI-9 ----G-----GW-----------EKR--DPHE--NPHFQELA----------------HY

IsCPI-10 ----G-----GW-----------KTQ--DLTN---PKFENLA----------------HY

IsCPI-4 ----G-----GY-----------TRKTDHQTN---PKYLELA----------------HF

IsCPI-3 ----G-----GY-----------SLKTDHHTN---PKYLDLA----------------HF

IsCPI-1 ----G-----GY-----------SERANHQAN---PEFLNLA----------------HY

IsCPI-2 ----G-----GY-----------RER-SNQDD---PEYLELA----------------HY

IsCPI-5 ----G-----GW-----------RSR--DVYS--DPAYAELA----------------HY

IsCPI-11 ----G-----VW-----------RRH-HPDMD---PRYKEWA----------------HF

IsCPI-7 ----G-----VW-----------IKHQPDMD----PRYKEWA----------------HF

IsCPI-6 ----G-----VW-----------RKHHPDVD----PRYKEWA----------------HF

IsCPI-8 ----G-----VW-----------RRHHPDVDH----RYKEWA----------------HF

ApCPI-1b ----G------------------AKISLNSND---KKVQDIV----------------AY

DmCPI-1a ----G-----DA-----------EST--ESSE---TTTDQAVSEPPITLVHVLNPGEREY

PhCPI-2 ----G-----DR-----------NLL--NISD---PEIIRLS----------------KI

AgCPI-2a ----G-----GI-----------VAI--HVDAVGGKEQLRPT----------------LR

PhCPI-1 ----G-----GE-----------KET--DVNN---PTVLSAI----------------KS

BmCPI-1f ----G-----GK-----------HEE--DPSD---KEFKVLA----------------QE

BmCPI-1d ----G-----GL-----------QLQ--DAHD---QKYKLLA----------------EE

BmCPI-1e ----S-----GM-----------TES--DVTE---PHYKKLA----------------QQ

DpCPI-1f ----G-----GH-----------GQV--DTMS---TEIEAYS----------------DF

DpCPI-1l ----G-----GI-----------SPA--DPSS---EEIKAHA----------------AF

DpCPI-1e ----G-----AQ-----------QSA--DKSS---PYIQSVA----------------DF

RpCPI-1 ----G-----EP-----------KEA--SVES---EEIKAAA----------------NF

RpCPI-2 ----G-----GE-----------SSL--DTND---KRVTDLT----------------DY

DpCPI-1c ----G-----CP-----------ANM--DVDS---IQVKELA----------------NF

DpCPI-1k ----G-----GV-----------TSM--DLHS---QKIKELS----------------DF

DpCPI-1j ---LG-----TY-----------ISI--DVSD---EQVGELA----------------TF

DpCPI-8c ----N-----KF-----------SSI--DVDD---PDVKAVG----------------EF

DpCPI-10b ----G-----GF-----------TSV--SIND---ADVLEMA----------------RF

DpCPI-5b ----G-----GY-----------SAA--SASE---EDVQEIA----------------KF

DpCPI-1g ----G-----GY-----------SAA--DPTD---PAILEIA----------------NF

DpCPI-6b ----G-----GY-----------VIR--NVND---SDVKEMA----------------AF

DpCPI-9a ----G-----GF-----------TPA--KLND---AEIVKMV----------------DF

DpCPI-1i ----G-----GY-----------CPI--DPNE---KAVKEMA----------------NF

DpCPI-1h ----G-----GY-----------SQS--DIND---ASVKEMA----------------DF

DpCPI-6a ----G-----GY-----------KPA--MVDA---ADVKRMA----------------AF

DpCPI-8a ----V-----GF-----------YRI--DVDD---VEVKEIA----------------LF

DpCPI-8f ----D-----GY-----------SPV--DVDN---SYVKEIA----------------AY

DpCPI-8i ----N-----GF-----------SPV--DVNN---PEVKEMA----------------NF

DpCPI-8h ----D-----GF-----------ASL--DVED---VKVKEIA----------------AF

DpCPI-8g ----S-----GF-----------SPA--DVQA---DDVREMA----------------YF

DpCPI-4a ----G-----EF-----------SPI--DVND---SEVREIA----------------DF

DpCPI-3 ----G-----GY-----------SPA--NVHD---IDVNEMA----------------DF

DpCPI-4c ----G-----GF-----------MPM--NVNN---EQVREIA----------------EF

DpCPI-7b ----G-----GY-----------KTI--DVND---ATVKEMA----------------EF

DpCPI-2d ----G-----GF-----------STI--DVDD---AYIKEIA----------------DF

DpCPI-7a ----G-----GF-----------SPL--DVEN---VTVKEMA----------------AF

DpCPI-9b ----G-----GF-----------SSL--DVDD---AEVKEIA----------------AF

DpCPI-4b ----G-----GY-----------SPI--DVND---AKVKEIA----------------RF

DpCPI-8d ----G-----GF-----------SSI--DVND---PAVKEIA----------------SF

DpCPI-2a ----G-----GF-----------SPI--DVDD---PNVKEIA----------------DF

DpCPI-2b ----G-----GF-----------SPI--DTDD---ASVKEMA----------------DF

DpCPI-8b ----G-----GF-----------SQI--DVDN----DVKEIA----------------IF

DpCPI-8e ----G-----GF-----------YPT--DVEN---PEVKEMA----------------IF

DpCPI-2c LIRHFVREDV-----SSDDIGKLDKLVMADS--------NEVI--FTRKSANELCNLPPI

DpCPI-5a LIRHFARQSI-----SSDQLAKLDKLVMADS--QE-VVLSGKS---------GIEICDLP

DpCPI-10a LIRHFARQSI-----SSDQLAKLDKLVMADS--REAVITRTAD--GKISIDNAEIQPGAI

BmCPI-1h SIKELEK--------NPDQKYKLIHLGTPYLVPSL-D--SDVP--IKLSFLIGSTNCTKE

DpCPI-1b SLAIIDA-ES-----LSAFQFKVIGYSLVDS--RVSVDSGTRD--LDLKMGLVPTLCLKR

NvCPI-1b AVELLDD-LD-----EDDKRKHVIDVIDSRRERQD-KDNSNVV--IYMTVEVAATDC---

TcCPI-1b AANALDS-ID-----DDNNKRIILQILGAKK--MVGD--DGVY--YHIIMRMGVSRCLED

SmCPI-1 VKDAVES--------KLNKTFSEFQATSFKS--QV-V--AGVN--YFIKVHVGAG-----

BmCPI-1g RANQYLNQVS-----DTNNLYKLITVHAIKYGKQM----GRNI--VQMYIEVAPTFCLR-

DpCPI-1a AANSILKDAETFGIQNTSSLHKIMDAHVVPA-----SKPSPTQ--YMLTIELG-------

DpCPI-1d ALNRLDSFDD-----SNSKKRILVTVVEGTA--ST----EGRSKTFKMKIHVALADCP--

CfCPI-1a ALKSIDE-QT-----ANDYKHKLMSIVKVTR--SVPVSSNMIQ--YQLLLLIGESECL--

AmCPI-1a VAKSMDE-LI-----QNDFKHKVIDIVKVTR--AIPPSSNIIQ--YQILLHIGESDC---

NvCPI-1a VMQSMDE-AG-----VSDFKHKLISIVRVTR--AVPPGANVVQ--YQLLVEIGESNCL--

BmCPI-1i SLDKLEM-AS-----VHRYKQRVIQINSFST--KI-T--TGKV--TTIDFDVGYTSCLK-

BmCPI-1c SLMYLDV-KS-----NRNNKQKIVDVNSVST--QI-N--AGLL--TEIIFTVAYTSC-R-

TuCPI-8 ALERQNKEFG-------DRYKRLVSINQAKR--QT-L--SGYR--FEIEMIIRETDC---

TuCPI-12 AVETRNKKYD-------GHYKRLMSVDVAKK--QF-I--SGYR--FEIEMTIRETEC---

TuCPI-9 AVDDHNK-LS-----TNDYYFKLVKITSVSF--QA-L--NGIK--YSITFIIGQTKC---

TuCPI-4 AINHRNS-DE-----DTLYYRKLVNVESARM--QV-V--SGLK--YEVTLVIGETHC---

TuCPI-2 ATEHRNS-QI-----NSLYYRTLVEIKSAKQ--QV-V--NGMK--YELTLVLADTNC---

TuCPI-1 ATEHRNS-QI-----NSLYYRTLVEIKSAKQ--QV-V--NGMK--YELTLVLADTNC---

TuCPI-3 ATEHRNS-QI-----NSLYYRTLVEIKSAKQ--QV-V--NGMK--YELTLVLADTNC---

TuCPI-14 AVADINGK-------EKLFYNKLIQIKEAKS-----S--KAKF--YVFKVIVGKTDC---

TuCPI-18 AVAHINE-KW-----EKLFYNKLIEIKEAKS-----VADDGIT--YMLKMIIRLTYC---

TuCPI-13 AVADVNS-Q------EKFTYNKLIVITEAKS--VV--D-DGVT--YMLKMIIQKTYC---

TuCPI-17 AVANVNS-Q------EKFNYNKLIEIQEPKS--VV--D-DGVT--YMLKFIIRSTYC---

TuCPI-15 AVAKINA-EG-----NGKFYNKLIEIKEARS--KLRH--SKIT--YTIKVILRKTYS---

TuCPI-16 AVAKINA-EG-----NGKFYNKLIEIKEARS--KLRH--SKIT--YTIKVILRKTYS---

TuCPI-11 AVVDINA-KE-----NSSYYNKLVQIRAAKS--RL-M--SGIE--YELRLDIQKTDC---

TuCPI-25 AVAYINA-QD-----NSLYYNKLIQIKEAKS--RV-A--DKVE--YELKLVIRITDC---

TuCPI-10 AVVDINA-QD-----NSLYYNKLILIRDKRR--VG----DKIE--YELKLVIRQTDC---

TuCPI-24 AVVDINA-HD-----KSLYYNKLIEIREAKS--RV-A--DKIE--YELKLVIRITDC---

TuCPI-21 GIEHRNK-ND-----NSIYYEKLIAIKKAQT--QA-L--APAK--YKIEFLIGPTEC---

TuCPI-6 SVNHHNS-VN-----NSAYYKKLVKIQEARY--QV-V--AGFK--YEIKFLIGKTEC---

TuCPI-20 GLDYENR-YG-----NSFYYKKLITIKEARI--QA-AKPYGVN--HEVKLLIGQTDC---

TuCPI-23 GLDYENR-YG-----NSFNYKKLITIKEARA--QA-DKVSGIN--HEVKLLIGQTDC---

TuCPI-22 GVECYNK-NS-----NNIYYNKLIKIKKAAS--EI-V--AGML--YEIKFLIGATDC---

TuCPI-5 GVEHHNK-IA-----NNLYYKKLITIKEAKS--QV-V--AGMN--YEVKFLIGKTEC---

TuCPI-19 GVEHHNK-IA-----NNLYYKRLISIEEAKS--QV-V--AGIN--YEVKFLIGKTEC---

BmCPI-1b SLKKIEK-ES-----NEKNAMKVSKIIDANV--QK-T--SGIL--TKFLVVLDRLNCSQ-

TcCPI-1a ALEHIQT--------ERAKKHALVKVLRLQQ--QV-V--TGVK--YILTAEFAPTLCE--

BmCPI-1a AVHHLDRH-------DDTAKYSLISVVDVERQVQV-V--NGVR--YILTLLVNNNTC---

SmCPI-2 ALARIEA-QS-----NALFKQRISHVTNAKR--KV-V--AGLM--YEFKMKTEYTSCR--

BmCPI-1j ALKHIEA-RY-----PNPRKQKILRIFTLEK--QV-V--AGIH--YRMKVEVGLTNC---

TuCPI-7 AAKLIND-QS-----NDMYFQNLIHIHDVKS--QV-V--GGVK--YNITFDMSKTICRKN

TcCPI-1f ALNQLDA-ES-----EHENKFKVHEFISATS--QI-V--SGHI--YRINAKVILSDCK--

TcCPI-1h ALNQLDA-ES-----EHENKFKVHEFISATS--QI-V--SGHI--YRIKAKVILSDCK--

TcCPI-1d ALNQLDS-ES-----PHENKFKVHEFISATS--QT-V--SGHI--YRIKTKVVLSDCK--

AgCPI-1 ALATTDG--------HAGKAYKLHRVTK-----QV-V--SGVQYVYFISFENEESG----

AgCPI-2g GMVSYNS--------ERSKAYNEFEILAGST--QQ-V--AGSL--YKYTFRVTSESD---

AgCPI-2d GLQQQSA-LV-----DGSGNERKVKVVGATV--QL-V--AGKS--YTYRLSFPDDELK--

AgCPI-2e ILSF-----------HGLTRGNSLKVINATS--QV-V--AGMK--YVYFIQHNN------

AgCPI-2f ILVSS----------GGSKESSNARIVSGTV--QI-V--SGKL--YKYAVEF--------

NvCPI-1c GLEKYTE-NY-----QGTNQPMISNIKDVSV--QV-V--SGLL--YKIQTDIGVSTC---

AmCPI-1b GLKKFSE-NS-----EGSNEPMIVEIVDASR--QV-V--SGYL--YKIRVKLGTSNC---

CfCPI-1b ALRKVSE-ES-----DGPNEPFIVEIIEASV--QV-V--AGKL--YKIKAKLGTSDC---

DmCPI-1b SLDKLTA--------GEGPHYKIVKVYSASR--QV-D--SGIL--TRIDADL--------

DmCPI-2 TLAKLA---T-----GDGPNYQVVNVISASS--QL-V--AGSL--YKFEVKLSN------

DmCPI-3 TLAQLAT--------GDGPSYKAINVTSVTG--QV-V--AGSLNTYEVELDNGSDK----

DmCPI-4 TLAQLAN--------GDGPSYKALNVTSVTG--QV-V--AGRLNTYEVQLDNGSEI----

AgCPI-2c GLVGYES-----------GKHSNFEILFGTV--QV-V--AGTIHRYKIALK---------

TcCPI-1c GINGFNANYN-----SKNNKVKPVEVVSATT--QV-V--AGTL--YKITTKISESDCS--

TcCPI-1i SLTHLNT-QL-----TSSNKVKPVEVLSATS--QV-V--AGTI--HRIKVKISESDCS--

TcCPI-1e GLLNLNT-HL-----TTSNKVKPVEVVSASV--QV-V--AGSL--HRIKVKISESDCS--

TcCPI-1g GLLNLNT-HL-----TTSNKVKPVEVVSASV--QV-V--AGSL--HRIKVKISESDCS--

ApCPI-1a SLDSITQ-QT-----MSKRSLGLIRVVSAKS--QV-V--AGIN--YKIKLLVCEKDSTLG

IsCPI-9 AVSKGSK--------PKRYYDTVVTLIEVYT--QL-V--AGVN--YRLNYTYATTDCR--

IsCPI-10 AVSTQVE--------GREYYDTVLGILEVKT--QI-V--DGVM--FMLKFTTTQSTCKIE

IsCPI-4 ATSSWSAGQA-----NKAYYDTVEEVLEAQT--QV-V--AGIN--YKLTLKVAESVCE--

IsCPI-3 ATSSWSAQQP-----GKTHFDTVEEVERVET--QV-V--AGTN--YRMTLKVVESVCE--

IsCPI-1 ATSTWSAQQP-----GKTHFDTVAEVLKVET--QV-V--AGTN--YRLTLKVAESTCE--

IsCPI-2 ATSTWSAQQP-----GKTHFDTVVEVLKVET--QT-V--AGTN--YRLTLKVAESTCE--

IsCPI-5 AVSSQAG--------DSEFYDTVLELLEVET--QV-V--AGMN--YRLKFSTAETACKV-

IsCPI-11 AISSQVE--------DRTNFDTLMTLISVES--QV-TRPQDVT--SLETSSKEQHAAPKR

IsCPI-7 AISSQVE--------DRTNFDTLMNLMSVES--QV-I--VGVD--YKLKMKVAESDCVI-

IsCPI-6 AISSQVE--------DRTNFDTLMTLISVES--QV-I--AGVD--YKLKMKVAESNCVI-

IsCPI-8 AISSQVE--------DRTNFDTLMTLISVES--QV-I--AGVD--YKLKMKVAESDCVI-

ApCPI-1b ALLSIDR-QE-----GSNKPHVLSKIINVSK--QI-V--SGII--YNIELEICDN-----

DmCPI-1a LSPNLIG-VQNIAMTFLPLSMNFVNIIDAFR--EI-T--AGVR--YEILLNA--------

PhCPI-2 ALLKLNEEKN-----IFNQEKILVKILKASK--QI-I--SGSL--TELTLQILE------

AgCPI-2a GLSYLATSFL-------PRDYKFVEIVSATR--EV-V--AGVR--YELLASAEDET----

PhCPI-1 TMVKLNENLS-----SGENEKKFVETLKATV--QV-V--SGTL--TRVLLRINQGEET--

BmCPI-1f SLHEYAR-LE-----KNDFIHKVIDVNRVST--QT-V--SGKI--YNIHFSAVPTSC---

BmCPI-1d SLRQFLQ-KN-----GTTKPHTVVRLNKVTT--QV-V--SGTL--IRLDFVAAPTG----

BmCPI-1e SLNQFLK-ES-----GNTKPHIVVRLNKVTT--QV-V--SGTV--TQLDFVAAPTG----

DpCPI-1f ALAVIEE-QS-----NADEKLKVTKILASSV--QI-V--QGKN--IRLSLEVASTSC---

DpCPI-1l ALQAIQA-QS-----NSRNLLNIVRIKNAGT--QT-V--AGKK--IYLTIEIGQTKC---

DpCPI-1e ATKAISQR-------SNGNILNLIRVIRADT--QL-V--AGKK--VTLDVEVGFTNC---

RpCPI-1 AAERIDQ-MS-----NSIYKQILVRILEATS--QV-A--AGIK--MDLKLELGNTECM--

RpCPI-2 VEDELTN-RS-----NSQYTKTIVKVLNATV--QV-V--SGKL--TRLTVEVTDTNC---

DpCPI-1c ALSALED-AA-----NCTKVQSILRITKATS--QV-V--SGTL--YVLTIELVDTNCI--

DpCPI-1k AVSAISL-RS-----NEPNAPSKVRVLNASK--QV-V--SGMM--YTLQLELNFVDC---

DpCPI-1j ATTIL-S-HV-----RNAGDLTLVKISSASK--QV-L--DGPN--YRLGLQV--------

DpCPI-8c AMKVANT-AAASS--GHSAPVKLVKILKAEW--QV-VDAVGRN--FKLTLEL--------

DpCPI-10b ATNALSV--------NKASPLVLVAVVQAEK--QI-V--AGVN--YRLQLKF--------

DpCPI-5b ATHALSQ-NA-----NQASPFVLVQVVKAEK--QI-V--SGIN--YRLHVELKENAD---

DpCPI-1g ATQAASSSQSTDA--SNSAPFTLAKIHSAKK--QV-V--AGIN--FKLDLEFTRLNES--

DpCPI-6b AFSIL-T-AN-----SHPHHLALIKILKAES--QV-V--AGTN--YKMALLF--------

DpCPI-9a ATTAV-S-AS-----MNSGPVKLLKIVKAEI--QA-V--SGTK--YKLNLEL--------

DpCPI-1i AAISL-S-RS-----MNSVPLKLAKIRFAER--QV-V--AGFN--YRLDLEF--------

DpCPI-1h ATQAI-S-RS-----TNAGALSVAKIISAET--QV-V--SGRN--YKITLQV--------

DpCPI-6a ATSAI-S-AS-----DSGPAVQLIWIRRAWK--QV-V--SGTN--YKLILEL--------

DpCPI-8a AAKALSK--------SQNSILKLNKIILAEA--ED-V--AGKN--FKLVLRL--------

DpCPI-8f ATTAI-S-SS-----RNSITLSLQRILNAEA--RV-F--GGTN--YKLTLEL--------

DpCPI-8i ATSSI-S-AS-----DNPFILKLIKVVDAEA--KF-L--FGKN--FKLILRV--------

DpCPI-8h ASNSIS---------ANSGPVTLLRILKAEA--QT-V--AGKN--YKLIIEL--------

DpCPI-8g ATHVIS---------NTRNPVALAEIVKAES--QAHA--AGRN--YKLTLKL--------

DpCPI-4a ATTAI-S-AS-----SNAGPFRLIKILKAES--QVGI--GAVN--FKLTLEV--------

DpCPI-3 SRRAI-S-SR-----SNSGPSTLIRIVKAEK--QV-V--AGMN--YKLTLEM--------

DpCPI-4c ATSAI-S-SK-----INSGPVTLVNIVMAES--QT-V--AGKN--YKLTLEL--------

DpCPI-7b ATSAI-P-EK-----MNSGPVTLIKIIKAKS--QF-V--AGIN--FKLTLEL--------

DpCPI-2d ATTTI-S-AN-----NNSGPVRLIRIIKAES--QI-V--AGKN--FKLTLKL--------

DpCPI-7a ATTAI-S-AN-----TNSGPVTLVKVVKAQS--QV-V--AGLN--YKLTLEL--------

DpCPI-9b ASSAI-S-AN-----TNSGPLTLVKVLKAQS--QV-V--AGLN--FELRLEL--------

DpCPI-4b AAAAV-S-IN-----LNSGPIALVNIVKAES--EA-V--AGRN--YKLILEL--------

DpCPI-8d ATSTIAS-KK-----SKSGPTKLIKIVKAES--EN-V--AGIN--YKLTLEL--------

DpCPI-2a ATSTI-S-ES-----SNSGPLSLIKIVKAES--QV-V--AGRN--YKLILEL--------

DpCPI-2b ATTRI-S-ES-----SNSGPLALIKVVKAES--QV-V--AGMN--YKLTLEL--------

DpCPI-8b AGTAIASK-------RNSGPARVTKIAKAES--QI-V--AGTN--YKLTLELNQPLA---

DpCPI-8e ASKVL-E-SK-----RNSGPTKVTKIVTAES--QA-V--AGTN--YKLTLEI--------

DpCPI-2c DSDRSECEAYIPSWTFQPNSGRCENYVYG--------------------GC-----

DpCPI-5a PIENKGFECYALKHSWTFKSGKCVNYVYG--------------------GC-----

DpCPI-10a ALSQNLGNVYLVERVFMTGDEVSNA--ISAH-FQNNPN---------TALC-----

BmCPI-1h VDIENSPLQCFLDGSKSS--KPCTSFVWFVP-NT-KDI-----YQI-NVQC-----

DpCPI-1b PEEENEDESKSCTIDTERDPSMCTITLTQWP-WV-LDG-----YLFSDFDC-----

NvCPI-1b SEKDQGTECLDEVLPGPT--QICKLDISTNEKKPLQSP-----KLL-HWSC-----

TcCPI-1b SPINPYENCKDKLFENYT--KICKVQVYVND-DF-GSK-----KVV-KSQC-----

SmCPI-1 --------------------EHLHLRVYKPL----PGQGELSLHSVQEGKT-----

BmCPI-1g HADENELGGCEEIEALDH--KLCYGRLWPSP----DDE---LVVQSVSVIC-----

DpCPI-1a NNCTNTNDNC----TGGR--HICEVSVLDAP-WNEKRV-----LDEDKTKC-----

DpCPI-1d EGGSANNEACLASLSGNPQHYLCDIQVLVPL----RDSRFVQRRLV-NSRC-----

CfCPI-1a KNALEQEQECPLRATNSI--KLCSVTFEQRP-WLPTSL-----KII-RNNC-----

AmCPI-1a LKNAIEQSECSVQLNSSF--KICLVTFEEKP-WQQSSR-----KIV-KNNC-----

NvCPI-1a RTSLIEIAECPLQSNLPI--KLCLVTFEERP-WQSGSR-----KIT-RNNC-----

BmCPI-1i YEWVDDVMTCQFLEHLPR--RHCVSKVFERL-WA-ANG-----KNI-DVSC-----

BmCPI-1c NDVKVDINTCNVLEDEPL--RNCKAQIWDRT-WI-EDG-----TQI-KVSC-----

TuCPI-8 NKNDPKKHQCQFNSARAP--EPVVFDVWVKG----D--------------------

TuCPI-12 HQNDPKKHQCQFNSARAP--EPVVFDVWVNT----RNQ------------------

TuCPI-9 FKTDPNHKKCDLLHKNINIMNLCSYLFWIKP-GTPKTV-----EII-HHAC-----

TuCPI-4 AKEDEAAMLCQVEPNSLR--EKCVYTFWLEA-KT-QNT-----NIV-TSSC-----

TuCPI-2 AKQDAGAKLCPVGQGAAK--EECVYTIWIES-TK-EAP-----EVT-SSSC-----

TuCPI-1 AKQDAGAKLCPVGQGAAK--EECVYTIWVES-TK-EEP-----AVT-SSSC-----

TuCPI-3 AKQDAGAKLCPVGQGAAK--EECVYTIWIES-TK-ETP-----EVT-SSSC-----

TuCPI-14 PIRGPYTDACQIKDDAPR--KECSVYYYYNT-VEWKHG---------TYTC-----

TuCPI-18 PKSKPYHDDCEIRQASSA--QICTVEGHKPS-GS-EEI-----KIS-NLSC-----

TuCPI-13 PLSKPYHDDCEINQGLPG--STCIVDAHKPV-GS-EEI-----KIG-RLQC-----

TuCPI-17 PISKPYHDDCEIRE-LPG--RICSVDAHKPV-GS-EEI-----KIG-RLQC-----

TuCPI-15 HKSKPYTDACGINDTAPP--KLCTIDAYVRV-GS-DES-----KIV-ILRC-----

TuCPI-16 HKSKPYTDACGINDTAPP--KLCTIDAYVRV-GS-DES-----KIV-ILRC-----

TuCPI-11 PKSKPYTDACQINVTTLP--PICTVHLHEDP-GS-KEI-----KIT-AFQC-----

TuCPI-25 PKSKPYTDACQINQDEPP--KLCTIDAYVRA-GS-EEN-----KIY-KFLC-----

TuCPI-10 PKSKPYTDTCQINQDTLP--QICTYELFSRA-GS--KN-----KIT-TLQC-----

TuCPI-24 PKSKPYTDACQINQDLPP--KLCTYELFVRA-GS--KN-----KFT-TLQC-----

TuCPI-21 LKTDPNSASCQISTNKAS--ETCIFVFLIRR-GS-NDI-----HIT-RDFC-----

TuCPI-6 AKTGNYTDSCQVAVNSPT--ELCTYVFWMPP--V-DKD-----RIT-SFDC-----

TuCPI-20 AKWNVNATSCEVSPNAIP--ELCTYVTWVSP-DL-HWR-----ELT-QASC-----

TuCPI-23 AKWNANATSCEVSPNATP--ELCTYVIWVSP-DL-HWR-----SLM-QASC-----

TuCPI-22 VKSEPDASSCKVSPNAIP--KLCTYHFWIKS-WS-GFE-----QIT-QVSC-----

TuCPI-5 VKSDANAASCEVSANAIP--ELCTYVFWVRP-GS-DNA-----QIT-QASC-----

TuCPI-19 VKSDANAASCEVSANAIP--ELCTYVFWVQP-GS-DNA-----QIT-QASC-----

BmCPI-1b NTPISLRQNCTTVEDLGS--KVCDVVVFEKL-WL-KDK-----DV--SFTC-----

TcCPI-1a KSANLDASSCPRDTNAET--TICEITYLHKP-WISKAK-----HVI-KNNC-----

BmCPI-1a --TENQSEDCQVV-------TPCRISILEKP-WLRLPSGVKYRSIL-SNNC-----

SmCPI-2 KDLFVDYSQCSVNTEHEA--QICFVNTWEKK-WE----------------------

BmCPI-1j TALTNRSDCKHISDESLN--KFCRVNVWMRP-WT-NHP-----PNF-RVTC-----

TuCPI-7 EIDSDKPEQCVPDRNATI--KRCYAVVYERP-WE-SKR-----QLL-DHKC-----

TcCPI-1f KTVSTERGQCGTLKDAKP--KTCKFEVFEQL-WV-PNS-----RQI-KTDC-----

TcCPI-1h KSISTERGQCGTLKDAKP--KTCKFEVFEQL-WV-PNS-----RQI-KTEC-----

TcCPI-1d KTASTERGQCGTLKDAKP--KTCKFEVFEQT-WV-PNS-----RRI-KTDC-----

AgCPI-1 --------------------QQYKITVWERP-WL-KEKDPAEARKI-TFEV-----

AgCPI-2g --------------------IVCKISIWERV-WL-ESQDQR---KY-NVKC-----

AgCPI-2d --------------------RVCKLTVWEKP-WL-KEKAPQEAFKA-SFEC-----

AgCPI-2e --------------------AVCKLTSWERV-WL-AQSHPEDAYKY-TYDC-----

AgCPI-2f ------------DVDGSS--KLCKLSSWERP-WL-EKK-----DPTEAYKY-----

NvCPI-1c -SKGTVTGDCQLSKDHGV--EECVIEAWSQP-WL-DKG-----NPKITVKC-----

AmCPI-1b --PKGTKEKCQLKEGTEI--KECLFSIWSQP-WI-DKG-----SPKITINC-----

CfCPI-1b --PKGTKTNCQLQAGSEV--KECLITVWSRP-WI-DHG-----SPEITITC-----

DmCPI-1b -----------IDGSEEQ--HRCIVDIWTKV-WV-RKD-----EHEITFKC-----

DmCPI-2 --------------GAET--KECNVKIWDRP-WL-HEQ--GEATNV-KVQC-----

DmCPI-3 --------------------KQCTVKIWTQP-WL-KEN-----GTNIKIKC-----

DmCPI-4 --------------------KQGTVQIWSRA-WL-KEN-----GTNIKIKF-----

AgCPI-2c DDDQKVYSTCDVKVFTPL--PSAAN--GSKP-------------DY-DFDC-----

TcCPI-1c KNDNKDLDDCNILEGASP--KTCELEVWEKL-WE-NFR-----QF--TIKC-----

TcCPI-1i KDDEKDFDECNIREGASP--KICEVKVWDKP-WQ-NFR-----QY--NITC-----

TcCPI-1e RNDQKDFEQCNVLEGASP--KLCEMEVWDKP-WE-DFR-----RY--TIKC-----

TcCPI-1g RNDQKDFEQCNVLEGASP--KLCEMEVWDKP-WE-DFR-----RY--TIKC-----

ApCPI-1a ENIVMDPKNC----------RSCDITIWEQS-WL-NKK-----NVT-KVAC-----

IsCPI-9 TDQEYKPSKCRPKGKVR---GWCESIVYEMP-CE-HIV-----QIS-QHHC-----

IsCPI-10 AGVEYSKLNCHPRTSKVV--LALHGVVASRS-------------------------

IsCPI-4 ITSQYTKEACTPKPDAVR--KTCTTVIYEKV-WE-NMK-----SVS-SFSC-----

IsCPI-3 LTSTYSKEACTAKANAAH--RNCITVIYENL--Q-GEK-----SVS-SFDC-----

IsCPI-1 LTSTYNKDTCLPKADAAH--RTCTTVVFESL--Q-GDK-----SVS-SFEC-----

IsCPI-2 LTSTYNKDTCQANANAAQ--RTCTTVIYRNL--Q-GEK-----SIS-SFEC-----

IsCPI-5 GVDEYSRERCLPKVNLPK--ATCTAVVYERP-WQ-NHR-----EVT-SYEC-----

IsCPI-11 TSIVLVTFLVSFCFLQPY--MLCTAVVNYRP-WE-HKT-----SLK-SYNC-----

IsCPI-7 GVDSYSRERCYLKVDVPY--MLCTAVVNYRP-WE-HKA-----SLK-SYNC-----

IsCPI-6 GVDSYSRERCHLKVDAPY--MICTALVNYMP-WE-HKT-----SLK-SYNC-----

IsCPI-8 GVDLYSRERCHLKVDVPY--MICTAVVNYRP-WE-HKA-----SLK-SYNC-----

ApCPI-1b STSEVDEKKC----------RICNIKVWEQA-WE-NNK-----NTS-EFNC-----

DmCPI-1a LDTKAIQP------AEAD--IVCRLVILEKP-WLRTQWGDKHRELV-TSNC-----

PhCPI-2 --------------KNVP--KYYVAKIWERP-WL-NKT-----EVT-FFDY-----

AgCPI-2a --------------TGQR--HLCQLVILEKP-WITNEYGEKYRTLE-YTNC-----

PhCPI-1 --------------------HYCYSKVWEQL-WL-NKT-----EVL-AHHC-----

BmCPI-1f STAVQDPSFCEQKDGSSI--LQCHARIWSRP-WL-GKK-------TTTITC-----

BmCPI-1d --------------EESR--YQCHSEIWERP-WL-KKT-----DI--EVNC-----

BmCPI-1e --------------EESH--YQCHSKIWEQP-WL-KKT-----SI--EVDC-----

DpCPI-1f KKDQPIGDNCAIDESKGF--QVCNIQIWDRA-WL-QEK-----QVT-DLNC-----

DpCPI-1l -PANETSQSCSFDDQTDR--QLCKIEIWTRP-WL-NER-----TVT-SLKC-----

DpCPI-1e SKAEGAGTFCQLDSSQAN--VICHVAVWDRA-WL-NDR-----KVT-NVTC-----

RpCPI-1 KNMDKKANCEVVSENAEK--MICRVSVWSQP-WK-QSSGKSHLKLS-KFYC-----

RpCPI-2 ---------------------------LKSE----NKL---------KSLCSASNQ

DpCPI-1c RSENTDRSQCPANELTEGNHRQCTVGIWDQP-WL-NSK-----QIR-EPQC-----

DpCPI-1k ---QQDSEACIRR-------QICNVSIWEQP-WL-KKR-----EMT-KLTC-----

DpCPI-1j SSV-----------DGTN--LMCDVVVYSQG----NAR-----QLT-YSSC-----

DpCPI-8c DDG-----------AEES--LLCVVSVFEQSTWKMIQL-----SFV-TREV-----

DpCPI-10b NGQQLESEENHF--------IDCQVTVFDQV-WT-ATR-----QIT-SFQC-----

DpCPI-5b --------------SANV--ISCTVVVYDQS-WT-STR-----QIT-SSEC-----

DpCPI-1g --------------------LFCRVIVLEQS-WL-SVR-----EVT-NMTC-----

DpCPI-6b ANRPQHH-------SRYL--LLCDVIVFDQP-WT-HTR-----KLT-EYKC-----

DpCPI-9a AGA-----------YSKV--IPCEVVVFHQP-KT-NTQ-----KML-RSSC-----

DpCPI-1i TE------------PRGT--VHCKVVVFDQA-WT-STR-----ELS-QMQC-----

DpCPI-1h QG------------DAGV--QTCTVVVYDQS-WT-KTR-----KLT-SFKC-----

DpCPI-6a LNTN----------TGQV--LLCEVIVFDQP-WT-NTL-----ELR-SFRF-----

DpCPI-8a ----ENLDEEEVSFSKSF--INCEVVVFDQS-WT-STR-----ILR-ESDC-----

DpCPI-8f DHFIAGA-------KAEN--LLCKVIVFDQK--E-DYR-----KMT-DSLC-----

DpCPI-8i KNMVKGAD------GDRE--MLCEVVVFDQS-WT-STR-----KVT-ESTC-----

DpCPI-8h -----------IGTERDI--QICDVVVFDQE-RS-QTR-----ILI-DSKC-----

DpCPI-8g DSMVKETTAGIPTFQSGD--LLCEIIVFFQT-WS-NTR-----ILS-ESNC-----

DpCPI-4a DGA-----------DEKN--LRCEVVVFDQS-LV-KSW---------TSVC-----

DpCPI-3 ENA-----------NDGV--ILCDVIVFDQP-WT-NTR-----RLR-ESSC-----

DpCPI-4c EGS-----------QRDK--HLCKILAFDQP-WT-KTR-----ILS-EFNC-----

DpCPI-7b EG------------VQGA--IQCDVIVFYQR-WS-KTR-----KLT-QSKC-----

DpCPI-2d NSAID---------EADS--LLCDVVVFDQS-WS-QTR-----QLK-QSNC-----

DpCPI-7a NG------------AEGA--ILCEVTVFDQS-WT-NTR-----KLT-ESKC-----

DpCPI-9b KG------------AKGA--ILCEVLFLRQC-WS-QIS-----AAPFRSDC-----

DpCPI-4b EGS-----------DGEA--RICEIVVFDQP-WT-NTR-----ILS-NSNC-----

DpCPI-8d NQPLAIDRS-----------IICDVIVFDQT-WT-STR-----ILS-ESHC-----

DpCPI-2a --------SSVVDGASETEETLCEVIVFHQP-WT-QTR-----KLS-KSNC-----

DpCPI-2b --------GSAADGAVGS-NLICNVLVFHQS-WT-HTL-----ELK-ESNC-----

DpCPI-8b --------------TEKF--LICDALIFDQS-WT-KTR-----ILS-EHRC-----

DpCPI-8e SQPIAVVE------TERF--LLCKVVVFNQP-WT-KTR-----ILA-EWNC-----

**I. I31 Thyropin.**

TuTyr-5b ---------------CSVALSRYTSDPDNYLDYQSSRH------------------L---

TuTyr-5c ---------------CEQLLQPLANLTSFLGRKGIDAI------------------GLNS

BmTyr-2e ---------------EEKISFKTKCQMLQAEIDNGSEG------------------YRP-

TuTyr-1b ---------------CD---CILQRHQITSEKPKRVGH------------------FTP-

TuTyr-1a --------------SSDTTSCTYRRQMETNSNTRLPGR------------------LIP-

SmTyr-6b ---------------------------GQFDHV---------------------------

TuTyr-4a -------QCAYGNTKCLQHRTEALKTSLNESEP----N------------------YIP-

TuTyr-4c -------------SEIEQMRCNCIRDKDLLRQANDKTN------------------WSD-

TuTyr-2b -------------PNCSESRQIALKNSANFSHS---KT------------------LIP-

DmTyr-2a -----------------CNACLEAVKFARRQQERDPGY------------------FVP-

DpTyr-4 -------------------------KKGGGGGGGGSGE------------------LIP-

DpTyr-5 -------------TRCQKMRQTQLKKGGGGGSG----E------------------LIP-

DmTyr-3e ---------------VEQATLKPMELKTTRCRALSKTA------------------PFP-

NvTyr-2f ----------------SAEGKEINKVLGSRCQAMRERG------------------FVP-

CfTyr-1f ---------------LEMDERRAGKILGTRCQAMKNKG------------------HVP-

AmTyr-2f ---------------LETDEKQIGKVLGTRCQAMKDKG------------------HVP-

AgTyr-3e -----------------EPIAKRLSASATRCQALQMAA------------------SFP-

TcTyr-2f --------------------------SNTRCEALGG------------------------

DpTyr-3c ---------------CEMMKEIADGRKPAEPGY-NLIL------------------KNP-

SmTyr-1b ---------------CEQQRLLAELLSRNEKSD---RG------------------YVP-

DpTyr-3b ---------------CLQQQQIAQLLSLTEREG---KG------------------YVP-

BmTyr-2a -----------NVTMCTQQKMLAELLVVSEREG---KG------------------YVP-

DmTyr-3b ------CQQPGNVTSCHQAKALADILSINEREG---RG------------------YVP-

AgTyr-3b --------QPHNVTVCHQARMLSELLSVNEREG---RG------------------YVP-

TcTyr-2b ------CTHPKNVTECLHQRALSEILAVSERAG---RG------------------YVP-

CfTyr-1b ---------------CHRDRMLAEILSVSERQG---RG------------------YVP-

NvTyr-2b --------------ACHRNRMLAELLSISERQG---RG------------------YVP-

AmTyr-2b --------------ACHRDRVLVEMLSISERQG---RG------------------YVP-

BmTyr-2c ---------------CQQRRALALHTAAESGNP-PAWA------------------WVP-

SmTyr-1g -------------------KMLTRCQKEEIVAQ-TWSQ------------------RVP-

SmTyr-1d ------------KTGCEHARA-------------VARH------------------FAP-

SmTyr-1e -------------------------------------L------------------VIA-

DmTyr-3d --------------QCERLKLKNNLAAQRTGHS--SVW------------------FQP-

BmTyr-2d --------------PCERLREKNEAAALKYGKG----T------------------FIP-

DpTyr-3e -------------STCEELREKNLKMAEKFKKV----V------------------FTP-

AgTyr-3d --------------QCERLRLKNAMAAKRAGQP--NTW------------------FQP-

TcTyr-2e ------------LSQCERLREKNQRAAERYHKP----T------------------FMP-

CfTyr-1e ------------LSQCERLREKNLKRSQRLKQP----T------------------FLP-

AmTyr-2e ------------LSQCERLREKNLKTSQRLKQP----T------------------FLP-

NvTyr-2e ------------LSQCERLRERNLKTSQKYKQP----S------------------FLP-

TcTyr-2d ---------------CQHQNALSQHQAHESGVP-AGRV------------------YIP-

DpTyr-3d ---------------CQHMQMIMKYKARENGLP-ANRL------------------FIP-

NvTyr-2d ---------------CQHARAVAEHGARESGEP-ARRS------------------YIP-

CfTyr-1d ---------------CQHARAVAEHAARESGEP-ARRI------------------YIP-

AmTyr-2d ---------------CQHARAVAEHAARESGEP-ARRI------------------YIP-

DmTyr-3c --------------ACQHLQAIQLHQSSELGIP-ARQM------------------AVA-

AgTyr-3c --------------ACQHLQTIQLHQASELGVP-PKQK------------------YIA-

TuTyr-2a ---------------CLLERSANLAVKLESDHQSGKEI------------------LIP-

NvTyr-2c ---------------CEYLRDFGERMEGTREGM-ALAI------------------PAP-

CfTyr-1c ---------------CEYLRDFNDRMEGTREGM-SLAI------------------PAP-

AmTyr-2c ---------------CEYLREFNERMEGTREDM-SLAI------------------PPP-

BmTyr-2b ------------------LRDFDEKMEGTVDGM-KLAL------------------PAP-

TcTyr-2c ---------------CEYLHDFSESMEGTREGM-TLAL------------------PSP-

TuTyr-3 ---------------CFDRKSKPCLIEKSQRKK-VYDE------------------FVP-

BmTyr-1 ---------------CLRRAARPCTALARAHPQPHAGA------------------YVP-

IsTyr-1 ---------------CDVNRDSFLSTIEWCACFNKSRA------------------YIP-

DpTyr-1 ---------------CFSNADRPCVAMRRRSKPGLLGA------------------YIP-

ApTyr-1 --------------RCFDKTDRPCTAIKRRISPNALGV------------------YVP-

PhTyr-1 -------------CHCFDKTDRPCTAAKRRLSPDMLGV------------------FVP-

DmTyr-1 ---------------CFEKTDRPCAAVRRRIAGDFAGA------------------YAP-

AgTyr-1 ---------------CFEKTDRPCAAVRRRLGNDLSGS------------------YAP-

TcTyr-1 ---------------CFQRTERPCAAVKRKITPELLGV------------------YVP-

SmTyr-3 -------------------------------------S------------------YIP-

NvTyr-1 ----------------FSKAERPCAAVRKRSSP----E------------------VAP-

CfTyr-2 ---------------CFYKAERPCAAVRRRSSP----D------------------SAP-

AmTyr-1 ---------------CFSKAERPCAAVRKRSSP----D------------------VAP-

NvTyr-2a ---------------CEQLALAARRRSRALGPG-SPAQ------------------LVP-

CfTyr-1a ---------------CEQLALAAVRRSRALGAE-GLSQ------------------FVP-

AmTyr-2a ---------------CEQLAQAAVRRSRALGPR-GPAQ------------------FIP-

DpTyr-3a ---------------CERQRVETVKRARALQMT-DNDV------------------SLP-

SmTyr-1a -----------------------EKRARALETH----V------------------PIA-

SmTyr-1c ------------------------------PLSRNLAY------------------PAP-

TcTyr-2a ---------------CQTLRMAASRRAKALGVE-ARSV------------------RMP-

DmTyr-3a --------------ACQHLRRSESRRAKALEGS---SV------------------RVP-

AgTyr-3a ---------------CQHLRRAEARRAKSLGDSLLQTV------------------RIP-

TuTyr-4b -------------CWSELTRRNEELKFLKKKFDFVVGY------------------ELP-

TuTyr-5a IQQREKAINLNGKSFCSNRAGILVKTGFLFETS----E------------------YQP-

SmTyr-4a ---------------KTCREERQEALDRQENNPEMVGI------------------HVP-

SmTyr-4b ---------------CHEERQDALDRQQNNIGM--VGI------------------HVP-

SmTyr-6a --------------------------VSYEEADRNSEW------------------FLP-

DpTyr-3f -------------------SSTCTSPEENSSGPLMMSI------------------SPP-

SmTyr-1f -------------------------------------V------------------TLP-

SmTyr-2 ------------------------EREKAQQNQNILGL------------------FVP-

ApTyr-2a ---------------NDSLLRVKHRGRCKGDPTRTPDS------------------YMP-

PhTyr-2a ----------------CRTRQPCFRELKKAERNRENQE------------------FVP-

AgTyr-2a --------RGSCKDVCIASRTYALQQRASSPYT---VK------------------YVP-

DpTyr-2a -----------------------SAGGLGKKSAPTGET------------------FIP-

TcTyr-3a ---------------CHFEKARCVNKNLTLAKRGPCRQQKLCRDWEIYRHSNPDYKFHA-

RpTyr-1 -----------------CSDKQPCWADRNGRDPEGGLV------------------FIP-

NvTyr-3a ----------------------------------GARI----------------------

CfTyr-4 ----------------ETPACFSARLTARLGAR-------------------------P-

AmTyr-3a --------------------TPACFSARLTARP----S------------------ARP-

IsTyr-2b -----------EVQDCSSARRKALEAHRQAPKG---RI------------------YVP-

IsTyr-2a ---------CPDARRCWSQRSQALEQVRSGASG----V------------------FVP-

SmTyr-7 -------------------------QLAADQQHTEGKV------------------FVP-

DpTyr-2b ------------ANDCYSDREAAQEEVDHGAKG----M------------------YVP-

DmTyr-2b ------------DSNCWMDQSVTLEEQGHGGKS---VL------------------FVP-

ApTyr-2b ------QEEEAEVNDCLTDRQEALDDPSTSSHK-----------------------YIP-

NvTyr-3b ---------------CISDRRSVMEDQRQNSDR---KF------------------YIP-

AmTyr-3b ---------------CLTDRRSVLEDERQHSQE---KF------------------YVP-

CfTyr-3 ----------------MSDRRSVLEDQKQNSQE---KF------------------YIP-

TcTyr-3b -----------------DEDPSDCLSDRQTALNDGGQF------------------YVP-

PhTyr-2b -------DQEPEANDCFSDRQAVLAEQRSNN------L------------------YVP-

AgTyr-2b -------------SDCLSDRKYALDEQKYGTNA----L------------------YVP-

SmTyr-5a --------------------QKPCRVDRAKTTG-----------------------KYP-

TuTyr-5d -------------------------IQEYEITTLGKPY------------------DKL-

SmTyr-5b -----------------------------------VGK------------------TVS-

SmTyr-4c --------------------------------HGMIGR------------------TIA-

TuTyr-5b -HCSEN-GN-FASLQCH------------------------------DQL--CFCADPST

TuTyr-5c MQCDLD-GN-FVHRQCT------------------------------DSE--CSCVN-SV

BmTyr-2e -RCLSD-GS-FSPRQCV------------------------------RGR--CWCVD-AA

TuTyr-1b -QCEEN-GH-FKKYQCH----AS------------------------TGH--CWCVHPTN

TuTyr-1a -ECRPD-GK-FASLQCH----GE-----------------AV-----GGGRFCQCWD-PE

SmTyr-6b -ICQKN-GN-FHKLQCL------------------------------RDT--CFCVNETT

TuTyr-4a -GCNTF-GD-YSNYQCF------------------------------GKR--CFCVD-EN

TuTyr-4c YDCDHL-GN-YNPIQCF------------------------------GDS--CFCVD-QN

TuTyr-2b -SCNNQDSFLYNEVQCH----EL------------------------PGF--CFCVRPSS

DmTyr-2a -RCRKD-GN-FAAMQCY----GN------------------------NG---CWCSD-SQ

DpTyr-4 -ECDHL-GR-FQPIQCL----PA-----------------KSS----SGQVSCWCVD-EA

DpTyr-5 -ECDHL-GR-FQPIQCL----PA-----------------KYS----SGQVSCWCVD-EA

DmTyr-3e VSCDEA-GA-FRPLQC-------------------------------NGR-SCWCVD-AA

NvTyr-2f AICDKY-GR-FEPTQCA------------------------------GET--CWCVD-EA

CfTyr-1f TICDPQ-GR-FEPTQCA------------------------------GDT--CWCVD-EA

AmTyr-2f AICDRQ-GR-FEPMQC-------------------------------AGD-TCWCVD-EA

AgTyr-3e VACDTA-GS-FEPMQCN------------------------------GDT--CWCVD-AA

TcTyr-2f -QCDTT-GK-FLPTQCE------------------------------EET--CWCVD-EA

DpTyr-3c -RCTPQ-GE-FEEEQCD----K-------------------------DGQ--CWCVD-EF

SmTyr-1b -ECSVK-GH-FKSKQCS----R-------------------------NGL-VCWCVD-LR

DpTyr-3b -QCDED-GQKFAARQCS----R-------------------------NGL-VCWCVDPEL

BmTyr-2a -QCAAN-GS-FESRQCS----R-------------------------NGL-VCWCVD-TD

DmTyr-3b -ECNGPGGQ-FSPRQCS----R-------------------------NGL-VCWCVDPRT

AgTyr-3b -QCDGPGGS-FSTRQCS----R-------------------------NGL-VCWCVDPKT

TcTyr-2b -QCSED-GQ-FEPKQCS----R-------------------------NSL-VCWCVD-RM

CfTyr-1b -QCRED-GG-FETRQCS----R-------------------------NGL-VCWCVD-DE

NvTyr-2b -QCSED-GE-YERRQCS----R-------------------------NGL-VCWCVD-AL

AmTyr-2b -QCSED-GG-YESRQCS----R-------------------------NGL-VCWCVD-NN

BmTyr-2c -QCTED-GA-YQEVQCR----RS------------------------DKT--CWCVD-TA

SmTyr-1g -SCDNE-GG-FVPTQCD------------------------------DRV--CWCVD-QA

SmTyr-1d -SCRAEDGQ-FETVQCA------------------------------NGT--CWCVD-DS

SmTyr-1e -RCNAFSGA-WESIQCL----PS------------------------LGL--CWCVN-RD

DmTyr-3d -RCDPVTGH-WSPVQCLGKQPQPMDRHTEIVSRAFASEPAASAGEEAPGV--CWCAD-KK

BmTyr-2d -VCDAS-GA-WEPVQCM----SH------------------------IDV--CWCVS-AR

DpTyr-3e -KCNKANGD-WEPVQCL----EE------------------------VGI--CWCVD-KD

AgTyr-3d -RCDPETGF-WSPVQCL----GSMEDTTNASNGTNGTAPAIAEPPAPVGV--CWCAD-KK

TcTyr-2e -RCEANTGN-WETVQCL----EH------------------------VGV--CWCVT-PQ

CfTyr-1e -KCNSDSGT-WEPVQCL----EH------------------------VGV--CWCVN-RK

AmTyr-2e -RCDSENGM-WEPVQCL----EH------------------------VGV--CWCVN-GK

NvTyr-2e -RCNPDTGA-WEAVQCL----EH------------------------VGV--CWCVN-KK

TcTyr-2d -QCTPE-GA-YEPKQCN----PG------------------------TNE--CWCVD-WR

DpTyr-3d -RCRPEDGA-FEAVQCD----PV------------------------TRA--CWCVS-AD

NvTyr-2d -RCDAD-GQ-FEPVQCH------------------------------AGM--CWCVD-EE

CfTyr-1d -RCDTN-GV-FEPVQCH------------------------------NGV--CWCVD-EE

AmTyr-2d -RCDTN-GA-FEPVQCH------------------------------NGM--CWCVD-KK

DmTyr-3c -QCDPNNGK-WNQVQCS----P-------------------------DGH--CWCVD-DQ

AgTyr-3c -QCDID-GS-FRTIQCG----P-------------------------GNV--CWCVD-EF

TuTyr-2a -ECDLTKGL-YKAQQCH----KE------------------------TGY--CWCVDVNN

NvTyr-2c -QCEED-GS-YRALQCQ------------------------------EKN--CSCVD-EY

CfTyr-1c -QCEQD-GS-FKSLQCH------------------------------NST-DCNCVN-HR

AmTyr-2c -QCEKD-GS-YKPLQCH------------------------------NGT--CSCVN-DR

BmTyr-2b -TCQQD-GS-FTSQQCA------------------------------NGR--CWCVD-SF

TcTyr-2c -SCDSD-GN-YISTQCH------------------------------KGE--CWCVD-NF

TuTyr-3 -KCDKD-GF-YQPIQCH------------------------------LHK--CWCVD-RY

BmTyr-1 -SCDAR-GF-YRPRQCH----AA------------------------LGV--CWCVD-AH

IsTyr-1 -QCDEE-GY-FMPAQCH----SS------------------------AGM--CWCVD-RH

DpTyr-1 -TCDSD-GF-FLPTQCH----TA------------------------VGT--CWCVD-KH

ApTyr-1 -ACDAE-GY-YEHTQCH----SS------------------------VGM--CWCVD-KH

PhTyr-1 -DCDNQ-GY-FRSTQCH----TS------------------------TGM--CWCTD-KH

DmTyr-1 -DCDIQ-GF-YKPTQCH----NS------------------------VGV--CWCVD-KH

AgTyr-1 -DCDSQ-GF-YKPTQCH----QA------------------------VGV--CWCVD-EH

TcTyr-1 -DCDNQ-GY-YRPTQCH----SA------------------------IGM--CWCVD-KH

SmTyr-3 -SCDEH-GY-YQATQCS----HA-----------------T------VEL--CWCVD-KH

NvTyr-1 -NCDSR-GY-YQSTQCH----RG------------------------LGL--CWCVD-QH

CfTyr-2 -SCDSR-GY-YRSTQCH----RG------------------------LGL--CWCVD-PH

AmTyr-1 -ACDSR-GY-YRSTQCH----RG------------------------LGL--CWCVD-PH

NvTyr-2a -RCDNVTGE-FERVQCD----P-------------------------SGG--CYCVD-EY

CfTyr-1a -RCDNETGE-FERIQCN----P-------------------------QGR-GCWCVD-EI

AmTyr-2a -KCNNETGE-FERIQCD----PR------------------------EKQ--CWCVD-EI

DpTyr-3a -SCDPI-GD-YEPVQCD----PL------------------------TGN--CFCVD-ES

SmTyr-1a -KCRSD-GS-FESIQCD------------------------------EAE--CWCID-DG

SmTyr-1c -QCTRE-GL-FEPIQCA------------------------------DGV--CWCVD-EF

TcTyr-2a -RCNKS-GG-FEPIQCD----NE-----------------I------VSS--CWCVD-EA

DmTyr-3a -RCQKN-GD-FDAIQCQ----DE-----------------K------HGR-DCWCVD-DY

AgTyr-3a -RCTAL-GD-FEPVQCS----NE-----------------L------NGT-ECWCVD-EY

TuTyr-4b -ECNLD-GS-YKAKQCD------------------------------ETS--CYCVN-QK

TuTyr-5a -ECDYETGQ-YKPKQCK------------------------------TSK--CYCVDPDS

SmTyr-4a -KCDAN-GD-YQPKECK------------------------------QAY--CSCLD-YD

SmTyr-4b -KCDQD-GT-YSPKQCI------------------------------EAY--CHCVD-KD

SmTyr-6a IQCEKD-GT-YKPIQCK------------------------------QDI--CFCVD-KD

DpTyr-3f -VCTLK-GD-YAREQSQ------------------------------GEF--SWCVD-TT

SmTyr-1f -QCTDD-GA-YSPTQT-------------------------------TGI-LSWCVT-TD

SmTyr-2 -ECKAS-GT-YKAKQCH------------------------------EAY--CYCVD-PQ

ApTyr-2a -RCKAD-GT-YFRIQCH----KK------------------------EGY--CWCVT-PA

PhTyr-2a -TCLKD-GT-FSPMQCH----NE------------------------TGF--CWCVT-PK

AgTyr-2a -RCRED-GT-YAPVQCI----D-------------------------GGG--CWCVN-GQ

DpTyr-2a -ECNED-GR-FAEIQCH----QG------------------------TGY--CWCVT-PD

TcTyr-3a -TCRPD-GS-YAAAQCH----PD------------------------TGF--CWCVT-PQ

RpTyr-1 -KCLSD-GR-YAPVQCH----EA------------------------TGY--CWCVT-PQ

NvTyr-3a -VCRPD-GT-YAPVQCH----PQ------------------------TSY--CWCVT-PQ

CfTyr-4 -ICQHD-GT-YAPIQCH----IE------------------------TGY--CWCVT-PQ

AmTyr-3a -ICRSD-GT-YAPVQCH----EE------------------------TGY--CWCVT-PQ

IsTyr-2b -ECGSD-GT-YAEAQCH------------------------------TGY--CWCVNQRT

IsTyr-2a -DCSAD-GA-FVQVQCH----RL------------------------TGY--CWCVD-AQ

SmTyr-7 -ECTIE-GK-YSKLQCF----KP------------------------TGY--CWCVDPES

DpTyr-2b -ECTPD-NK-YQRVQCH----KS------------------------AGY--CWCANDET

DmTyr-2b -QCLPD-GR-YQRIQCY----SS--------------------TS--TSY--CWCVNEDT

ApTyr-2b -ECTVD-GR-YKHVQCY----KS------------------------VGY--CWCAQEDT

NvTyr-3b -ECTPD-GR-YHKVQCY------------------------------SGY--CWCVYQDT

AmTyr-3b -ACTPD-GR-YHRVQCY------------------------------SGY--CWCVYQDT

CfTyr-3 -QCTSD-GR-YHRVQCY------------------------------SGY--CWCVYQDT

TcTyr-3b -ECTPD-GR-YKKIQCY----KA------------------------AGY--CFCVHEDT

PhTyr-2b -ECTPD-GR-YNRIQCY----KS------------------------TGY--CWCVNEDD

AgTyr-2b -ECTPD-GR-YQRVQCY----RS------------------------TGY--CWCVNEDT

SmTyr-5a -MCDGN-GN-YEPKQCE----YS-----------------PRQTGNTLKK-RCYCAH-KD

TuTyr-5d -LCDLY-GN-FKANQCF----ES-------------------------GS-RCFCVD-SN

SmTyr-5b --CDEI-GN-YKSAQCL-------------------------------GS-GCYCVDRKS

SmTyr-4c --CDKA-GN-YERSQCT-------------------------------GS-KCYCVDSKT

* . : : . *

TuTyr-5b GQQVSPLVLID---A--VSSL-SC----------

TuTyr-5c GENVANYRIDRYGND--DRTM-KC----------

BmTyr-2e GERRHHAGP----------VP-------------

TuTyr-1b GTQI-GEKS-R---T--LTAE-GC----------

TuTyr-1a GNIIRAPSK-K------IKAC-DC----------

SmTyr-6b GNLTSSIVP-K-----------------------

TuTyr-4a GDRIFGDFL---------DSQ-------------

TuTyr-4c GLPIDYERY-N---V--TLKD-------------

TuTyr-2b GQLIPGISS-K---L--PAKP-DC----------

DmTyr-2a GRPIADDNK-Q---FRRKGKL-RC----------

DpTyr-4 GNQVANTTQ-F---L--RGEQ-TC----------

DpTyr-5 GNQVANTTQ-F---L--RGEQ-TC----------

DmTyr-3e GNQLQSTHV-F---G--AGDR-RC----------

NvTyr-2f GNQLVGSEP-F---V--KGTN-IC----------

CfTyr-1f GNQLIGSEP-F---L--KGTS-IC----------

AmTyr-2f GNQLIGSEP-----F--LKGT-NI----------

AgTyr-3e GNQLPLSST-F---K--RGQR-SC----------

TcTyr-2f GNQLLHTNT-F---K--KGEI-TC----------

DpTyr-3c GVELAGTRG-V---A--SVQQ-RS----------

SmTyr-1b GNKLPRTMG-P---A---ENV-TCA---------

DpTyr-3b GTKVKGSMG-S---A---QDV-VC----------

BmTyr-2a GNKLRGSMG-P---S---ATV-HC----------

DmTyr-3b GHKIKETMG-A---A---NNV-NC----------

AgTyr-3b GNKLKGTMG-A---A---ATV-SCEVVENMIGGR

TcTyr-2b GRKIRGSMG-P------AGNT-NC----------

CfTyr-1b GRKISGSMG-P---S---KKI-DC----------

NvTyr-2b GQKVSGSMG-P---A---EKV-DC----------

AmTyr-2b GRKISGTMG-P---A---DKV-DC----------

BmTyr-2c GNEIPGTRT-S------NSTP-TC----------

SmTyr-1g GIKVPDFEN-------------------------

SmTyr-1d GVEFPGTRT--------LGRP-NCSSP-------

SmTyr-1e GDQMAGSLV--------RGVP-ACSS--------

DmTyr-3d GAPLKGTLT-R------ESEP-IC----------

BmTyr-2d GEPLKGSLV-R------GSKP-SC----------

DpTyr-3e GEHIKGSLT--------RGSP-TC----------

AgTyr-3d GAPVKGSLT-K------GSEP-KC----------

TcTyr-2e GEPLKGTLT-R------GAQP-LC----------

CfTyr-1e GQPIKGSLI-R------GTEP-KC----------

AmTyr-2e GQPMKGSLT-R------APEP-KC----------

NvTyr-2e GEPIKGSLT-R------DAEP-KC----------

TcTyr-2d GFEISKTRT--------NSQL-SC----------

DpTyr-3d GRELAGTRV-P---P--GLQP-QC----------

NvTyr-2d GREAAGTRV-V---E--GLLP-KC----------

CfTyr-1d GKEAAGTRV-L---E--GIVP-RC----------

AmTyr-2d GREAAGTRV-L---E--GIVP-KC----------

DmTyr-3c GKILPGTRV-K---S--PATP-KC----------

AgTyr-3c GNEKSGTRT-N------NGQP-NC----------

TuTyr-2a GKPIWKTSAKG------DKASTDC----------

NvTyr-2c GASLKSPVE--------PAST-DC----------

CfTyr-1c GVILKTGVG--------PTASADC----------

AmTyr-2c GVVLKSSVN-R--------SS-DCK---------

BmTyr-2b GTEIPETST-H------NASAVDC----------

TcTyr-2c GTEIPRTRG---------TTQ-NC----------

TuTyr-3 GIEIENTRQ--------TEIP-DCD---------

BmTyr-1 GVELPGSRT--------KGAP-AC----------

IsTyr-1 GAEFANTRR-R---D----RP-DC----------

DpTyr-1 GVEQNGSRA--------RGKP-DC----------

ApTyr-1 GVEVPNSRV--------RGKP-NC----------

PhTyr-1 GVEYANTRI-R-------GMP-NC----------

DmTyr-1 GVEFANTRT--------RGKP-NC----------

AgTyr-1 GVEFANTRT-R-------GKP-NC----------

TcTyr-1 GVEFANTRT-H-------AKP-NC----------

SmTyr-3 GVEYANTRM--------KGKP-DCDSIVGK----

NvTyr-1 GIEFAGTRV-R------GTKP-DC----------

CfTyr-2 GVEFAGTRT-R------GTRP-DC----------

AmTyr-1 GVEFAGTRT-R------GSKP-D-----------

NvTyr-2a GGEVAGTRA-P------QRRLVDC----------

CfTyr-1a GAEIPGTRA-L------NKNDIDC----------

AmTyr-2a GVEIPGTRE-N---S--IDAI-DC----------

DpTyr-3a GFELAGTRA-R------SLQLVNC----------

SmTyr-1a GFELAGTR--T---N--ETNV-NCSKPRP-----

SmTyr-1c GVELAGTRD-S---N--RRIV-DCT---------

TcTyr-2a GFELPGTRA-P---A--AALV-NC----------

DmTyr-3a GVELPGSRNET------RTGV-VC----------

AgTyr-3a GVEITGSRR-S------HADDVNC----------

TuTyr-4b GERYGARVT-----IPRDSSE-------------

TuTyr-5a GEKTFGTVE-S--VN--GSNM-TC----------

SmTyr-4a GYPIRGYL--------------------------

SmTyr-4b GNVIV-----K-----------------------

SmTyr-6a GNRIFGDFP---------LLE-------------

DpTyr-3f GQPIDDSFT--------RGSV-RC----------

SmTyr-1f GQPIHESIG-R-------GDI-RCSPEG------

SmTyr-2 GTQIKGTIF-------------------------

ApTyr-2a GKVVANTIV-R------GQKP-KC----------

PhTyr-2a GKLIPNSAK-R------HDKP-NC----------

AgTyr-2a GKQLPNTMV-Q------HGKP-IC----------

DpTyr-2a GKPIPGSSI-R------HNKP-NC----------

TcTyr-3a GIPLPYTSV-R---WRPDAKP-HCGRKKKSTRRR

RpTyr-1 GKPLPNTSV-R------HARP-KC----------

NvTyr-3a GRPIPNSTV-R------EGRP-RC----------

CfTyr-4 GRPLPDTSV-K------HKKP-RC----------

AmTyr-3a GRPLPDTSV-R------NERP-RC----------

IsTyr-2b GRPIRGIAT-L------GVKP-DC----------

IsTyr-2a GKVLSGSSV-Q------NRRP-NC----------

SmTyr-7 GKTIPGTSI-Q------DDKP-DCD---------

DpTyr-2b GKPIPGTSV-Q------NSKPTNC----------

DmTyr-2b GKSIPGTSV-K------NKRP-QC----------

ApTyr-2b GKPIPGTSV-K------DSNP-KC----------

NvTyr-3b GKPIPGTSS-K------DLTP-NC----------

AmTyr-3b GKPIPGTSS-K------NHTP-NC----------

CfTyr-3 GKPIPGTSS-K------NRTP-NC----------

TcTyr-3b GKNIPGTSV-K------NGKP-KC----------

PhTyr-2b GKPIPGTSV-K------DQLP-KC----------

AgTyr-2b GKNIPGTST-K------DEKP-VC----------

SmTyr-5a GHLLGNYYF-D-----------------------

TuTyr-5d GNRISNVIS--------SSVI-------------

SmTyr-5b GNRIGDVVH-I---N--QAQT-LN----------

SmTyr-4c GEKIGDVVP--------ISQK-------------

*

**J. I32 IAP.**

TuBIR-6a -----ESVRLQT-FYSMLG---QSW-TA----CYRDFEELAAFGFYHTS---AP-RT--V

NvBIR-10 ----TYEDRLHT-Y--------KLW-PK---KSSHRSETLAETGFCYNC---KE-DE--V

NvBIR-11 --YIAYKDRLYT-F--------ILW----LEGSSQKPEILAEMGLCYNY---YE-NQ--V

NvBIR-8 PDYMIYEDRLHT-Y--------KLW-LK---ESSYRPEILAEMGFCYNC---KE-DE--V

NvBIR-9 ----TYEDRLHT-Y--------KIW----LKESSPSPETLAEMCFCYNC---KE-DK--V

TuBIR-7 --------ELST-F--------ISR-LL-D--SLIKNQNLAEAGFYYTG---FA-DG--T

TuBIR-6b ------ETRRFS-F--------ISW-PH---TEMIPHKELAKAGFYYTG---DS-DT--T

TuBIR-8 -----ERTDQTK-F--------TYQ-IN-SMSKDARIATFAKAGFFSYG---EH-DA--V

TuBIR-15 -------CRVAS-F--------AKH-DG-HVVKEITPDSYAEAGFFSYG---EH-DA--V

TuBIR-1 -----IEARIET-F--------EKH-DG-HIVAQITPSKYAEAGFFSYG---EH-DA--V

TuBIR-2 -----KDARIAT-F--------ASH-DG-HVIKEITPEKYAEAGFFSYG---EH-DA--V

TuBIR-4 -------ARIAT-F--------ASH-DG-HVIKEITPEKYAEAGFFSYG---QH-DA--V

TuBIR-3 -------SRFNT-F--------SRW-PE---QNNQSPSKLAEAGFVYQG---RD-DI--V

TuBIR-11 ------DQRKNT-F--------SDW-PL---KDIISPQKLAEAGFFYSG---LF-DI--V

TuBIR-14 ------ESRLAT-F--------KDW-PN----PAITPLELAEAGFFYSG---QS-DL--V

TuBIR-5 -----LTSRLST-F--------TNW-TS----ESISAPELAKAGFLFAG---QT-DT--V

TuBIR-12 ------ESRLST-Y--------ETW-TN----ENKSPSELAQAGFLFIG---ET-NT--V

TuBIR-9 ------QSRIDT-F--------SNW-NS----ENISVNNLAEAGFVYSG---KA-DI--A

TuBIR-16 ------ASRLST-Y--------GDW--I---NTNISSTALAEAGFYYTN---KL-DI--V

CfBIR-5a ---RFEIVRLHS-F--------MKS-VL----KRECAQKYAKNGLYYID---KG-NK--M

RpBIR-2 -------QAIDKFFFK------RDV-VCIPTRNCNNNQEMAKAGFFYL----KG-DC--V

BmBIR-2b ------ALRLLS-F--------SHW-----EDDSVSREALVSAGFYHIG----G-GR--L

TuBIR-13 ------MKRLES-F--------ESW-KR----DDICRTELADAGFVYYD---VD-NK--V

CfBIR-5b -----YESRLNS-Y--------QLW-PV----NGLKKEDMAAAGFVCAN---YE-DK--V

CfBIR-4b ----YYERRLES-Y--------VSW-PV-TIPQ--KKEDLAAAGLICAN---DG-DI--V

CfBIR-3a -----EIVRFNT-F--------KNW-TV----LYVKSEKLSAAGFYFTG---RN-ET--I

IsBIR-4a -----EEHRRRT-F--------DSW-PQ---VSPTMALKLARAGFYHVG---RG--R--T

ApBIR-1 ----WQMKRLKS-L--------QNW-TM----GKPSAKDMAEAGFYCPNPD-IP-DT--V

ApBIR-10 ------MNRLNS-L--------QNW-TM----NKPSAKEMAEAGFYCPNQD-TP-DT--V

ApBIR-4 -----YENCLKS-F--------KKW-PS----ECITPDKLARAGFYYTG---IQ-DK--V

ApBIR-2a ---TTYENRLRT-FY-------GVW-KL----NFITPDQMAKAGLYYLG---IQ-DR--V

ApBIR-3a ------KNRLKT-FA-------GVW-KL----QFITPTQMAKAGLYYVG---PQ-DR--V

TuBIR-10 ----FEKNRVKT-F--------KNW-PH--KTGKISKENMAKAGWFKCLPESAD-DA--V

TcBIR-1 ------ANRKST-F--------KKW-VF-SDKVMCNAAKLAEAGFIFVGNSLEP-DS--V

DpBIR-4 ------ENRLST-FFKNGQGLSGRW-PF-LEDCNCTPEKMATAGFFWCGSESQP-DL--V

IsBIR-1 ----RESNRLTS-F--------ARW-PF-QENCACTPAKMAQAGFYHCPIDNEP-DL--A

SmBIR-1 -------DRLKS-F--------NKW-PY-QRESKCTPVEMARAGFYCPDEK-FA-DL--A

NvBIR-1 -----KKGRLET-F--------KHW-PFKSENHQCNPDNMARAGFYAIGGKDEP-DL--A

BmBIR-1 -----VEERIKT-F--------KNG-PF-NDKNKCNVRNMAEAGFYSVATGVEDADA--A

AgBIR-8 -----QEDREKS-F--------KHW-PF-SDDKQCSIQKMAEAGFYWHGTETEI-DI--A

DmBIR-1 -------HRVES-Y--------KSW-PF-PETASCSISKMAEAGFYWTGTKREN-DT--A

DmBIR-3a -------VRLAT-F--------GEW-PL---NAPVSAEDLVANGFFATG---NW-LE--A

SmBIR-3a ------RHRLES-F--------SRW-PA---NAPIEAKKLAKAGFYYKG---KD-FS--V

NvBIR-5a ----EEVNRLRT-F--------LDW-PA---NCPVSTARIAKAGFYYTG---TA-QI--A

TcBIR-4a -----EQNRLDT-F--------EEW-PQ---DAAVSPPRIAKAGFFYTK---HD-VT--V

BmBIR-2a ------TNRLNT-F--------TNW-PA---LAPVDPIRIAKAGFFYTG---QG-ME--V

RpBIR-3a --------RLRT-F--------ANW-PE---DAAVDPRRIAKAGFYYMG---QG-LE--V

PhBIR-3a ------EQRLNT-F--------RDW-PG---NAAVEPSRIAQAGFYFTG---PG-LN--V

CfBIR-1a ------EKRLAT-F--------REW-PS---NAAIGASCLAKAGFYYTG---NY-LE--V

SmBIR-4 ------RGRALT-Y--------DGW-NC----KFLSPAVMARAGFVYLG---YS-DA--V

CfBIR-4a --YRFENRRLMS-F--------TNCVSTVNSRHYLQYYNFASAGFYYIQ---ND-DK--I

ApBIR-5 ------ESRKKT-F--------ETF-TK---KLTHDVKTFCKAGLFYIG---EN-DR--M

ApBIR-2b -----LEARLKS-F--------EKC-LI---PLKQNIQTLCEVGFFYIGNG-TN-DQ--M

ApBIR-3b --------RLQS-F--------EKC-LI---PLKQNIQTLCEAGFYYQGTG-TN-DS--M

DpBIR-5 ------ASRRET-F--------ASW-PH-MNYKWALPSQMAEAGFYHQPNTPES-DR--A

BmBIR-3 -----EAERKET-F--------KRW-PH-MDYKWALPARMAQAGFYHQPSPSGD-DR--A

NvBIR-3 ---------INN--------------PN------DSNHQMAQAGFYHQPYSTGE-DI--A

AgBIR-7 -----EAARRQT-F--------EAW-PH-MDYKWVLPDQMAQAGFYHQPGENGNKDR--A

TcBIR-2 -----EAKRRET-F--------THW-PH-MDYKWALPDQMAQAGFYHQPNASGD-DR--A

DmBIR-2 -------VRRQT-F--------EKW-PH-MDYKWALPDQMAQAGFYHQPSSSGE-DR--A

NvBIR-4 ------AARRDT-F--------SKW-PH-MNYKWALPDQMAQAGFYHQPNSTGE-DR--A

CfBIR-6 ---FSEAARRNT-F--------PKW-PH-MNYKWALPDQMAQAGFYHEPNATGD-DR--A

SmBIR-2 -------ARRET-F--------GKW-PH-MNYKWALPDQMAQAGFYHQPNSTCD-DR--A

IsBIR-3 -----LVSRVQS-FG-------DEY----VQKFKGDPETLAKAGLFYNGF--MECDR--A

AgBIR-6 ------SARVRS-F--------RNW-PY---SGIIHPLRLAYAGFCWRG---VD-DK--V

ApBIR-9 -------SRFKT-F--------KLF-PS---NTSQNKYTLSECGLKYSG---LD-DV--V

ApBIR-7 ------LSRLKS-Y--------NSF-PP---TLCQNKYSLSEAGFKYSG---TA-DI--V

ApBIR-8 -----FTSRLKT-Y--------NLF-PP---TIPQNKYVLSECGFIYTG---VQ-DI--V

PhBIR-1 -------DRLET-FN-------EHW-KDLKGFKFCTPKNFAEAGFYNSSSVKFP-DN--V

CfBIR-3b -----YQTRFNS-F--------ISW-PL---VKIQTGQQLAEAGFFYTG---QK-DK--V

SmBIR-7 -------QRKKT-F--------HNW-PA--YKCNVDSQAPARAGFIYLG---EE-DS--V

SmBIR-5 -------DRLGT-F--------KNW-PI----DFVSPRSLARAGFIYLD---ES-DT--V

SmBIR-6 --------RLCT-F--------QNW-AN----PFVQPRALARAGFVFLD---EK-DI--V

IsBIR-5 ------DARRAT-M--------TNF-PR---EKFQDVESLVAAGFFYDG---YM-DR--V

DmBIR-4a -------TRLKT-F--------TDW-PL----DWLDKRQLAQTGMYFTH---AG-DK--V

CfBIR-2a -----ELARLES-F--------KNW-PC----AWMKPEKLAAAGFYYTG---ES-DK--V

NvBIR-2a -------LRLQS-F--------ENW-PS----EHVRPADLAAAGFYFTK---QI-DR--V

PhBIR-2a -----ESKRLKT-F--------THW-PV----SFINPKDLAKNGFYFTN---VD-DV--V

RpBIR-1a -----ESERLKT-F--------DQW-PV----EFMPRHKMAEAGFYYLK---KD-DI--V

BmBIR-4a -----EEERLKT-F--------DQW-PV----TFLTPEQLARNGFYYLG---RG-DE--V

AgBIR-1a -------NRLRS-FT-------SRW-PV----TFISPNVLARYGFYYVG---TD-DT--V

AgBIR-2a -----EINRLRTYF--------PLW-TV----PYIYPEELARWGFFYTG---YR-DC--V

AgBIR-4a -------NRLRT-F--------PLW-TV----PYIYPEELARWGFFYTG---YR-DC--V

IsBIR-2 -----REKRRAT-F--------VNW-PR---HAFSNVEALVDAGLFYEG---ED-DM--A

TcBIR-3a ------ADRLST-F--------IDW-KS----SAVTPEALAKAGFYFLNNPSKP-DL--V

DpBIR-3 -------ARLST-F--------QRW-EQ-SAPNSPTPQALSSAGFIYRG---VG-DH--T

DpBIR-1a ------DIRLRT-F--------NEHFSA----TFLSPLLLAKAGFFYVG---VD-DQ--V

AgBIR-3 ------DARIRS-F--------ESW-RF---GHMQNPTRLAVAGFYYTG---TD-DE--V

TcBIR-3b -------ARLES-F--------ATW-PS---SAKQSPETLADAGFYYRG---VE-DH--T

AgBIR-2b ------DNRLAS-F--------QEW-PK---CMKQTPEQMADAGFFYTG---KS-DV--V

AgBIR-4b -----GGDRLAT-F--------KEW-PK---SIPQTPTQMADAGFFYTG---KS-DV--V

AgBIR-5 ----IEGDRLAT-F--------KEW-PK---SMPQTPERMADAGFFYTG---KS-DV--V

IsBIR-4c -------ARLRS-F--------AKW-PP---ASPLRPPDLVKAGFFYIG---IL-DY--T

BmBIR-2c ------ASRLAT-F--------DSW-PT---DKQQTPKDLSEAGFFHTG---TD-DQ--V

RpBIR-1b ------DSRLRS-Y--------ATW-PV---SLKLKPHILSDAGFFYTG---KG-DQ--T

BmBIR-4b ------AARLAT-F--------KDW-PR---CMRQKPEELAEAGFFYTG---QG-DK--T

NvBIR-7 ---STQTALLKL-T--------DDI-RM-AVDKKKSKEEIADAGFFYGG---SG-DQ--T

CfBIR-2b -----YEARLST-F--------ETW-PK---AMSQTKEELAEAGFFYTG---NG-DQ--T

NvBIR-2b -----YEARLLT-F--------NDW-PS--TRVSQTKEQLADAGFFYTG---TG-DQ--T

NvBIR-6 -----YESRLLT-F--------DEW-PS---RVTQTKEELADAGFFYGG---SG-DQ--K

AgBIR-1b ------ADRLKS-Y--------EDW-PT---SLKQKPQQLSDAGFFYTG---MS-DR--V

DmBIR-4b ------TARLRT-F--------EAW-PR---NLKQKPHQLAEAGFFYTG---VG-DR--V

ApBIR-6b -----FSARLKS-F--------RGW--N---NESQKPEDLATAGFFFTG---SN-DE--V

RpBIR-3c -----FESRVRT-F--------ANW-PS---TVSQKPEQLAEAGFYFTG---NR-DK--V

PhBIR-2b -----YASRIKT-F--------DKW-EA---HNIQKPEKLAEAGFYYIG---HE-DN--V

DpBIR-1b ----TLDARLKS-Y--------NNW-PS---HLKQTPRAMALAGFFHLG---TN-DH--V

SmBIR-3c ------ENRLKT-Y--------ETW-PT---AIPMLPTALAEAGFYYAG---VS-DH--V

DpBIR-2b -------ARLKT-F--------DDW-PP---GLEQRPPQLAEAGFYYMK---TG-DH--V

DmBIR-3c -------ARLRT-F--------TDW-PI---SNIQPASALAQAGLYYQK---IG-DQ--V

TcBIR-4c ------ESRLRS-F--------ATW-PP---DLIQTPDILSQAGFYYEG---MG-DQ--V

PhBIR-3c ------DSRLRT-F--------ENW-PS---NLTQQPNVLAQAGFFYVG---RQ-DPDMV

CfBIR-1c ---TTYEKRLQT-F--------HNW-PK---NLKQTPEMLATAGFYYQG---YD-DQ--V

NvBIR-5c -----YEGRLRT-F--------QGW-PS---NLRQTPEMLADAGFYYVG---AQ-DQ--V

SmBIR-3b -------ERLKT-F--------RGW-PS----KVVKPEDLARNGFFYLR---DE-DK--V

DmBIR-3b -------NRLVT-F--------KDW-PN----PNITPQALAKAGFYYLN---RL-DH--V

ApBIR-6a ------SNRLST-F--------AGW-PV---SFIISPKCLAAAGFYYTK---QT-DK--V

PhBIR-3b ------SARLDS-F--------SNW-PI---PFIVTPEALAETGFYFLH---KG-DA--V

DpBIR-2a ------ALRLAT-F--------QGW-PL----EYLSPRDLSRAGFFYRG---LA-DQ--T

RpBIR-3b ------ESRMTT-F--------ATW-PI---PNVINPVKLAQSGFYYTQ---VD-DK--V

IsBIR-4b -------HRLRT-F--------ARW-PL----DFLDPTDLAGAGFYYLQ---QD-DR--V

CfBIR-1b -----VSQRLQT-F--------DSW-PL---TSIIRPEQLALAGFYYLQ---YK-DL--V

NvBIR-5b -----YSHRLNT-F--------RNW-PI---PAIVSPERLARSGFYYLQ---QA-DM--V

TcBIR-4b ------AVRLKT-F--------AKW-PK---PHIVAPERLARAGFYYLN---TG-DN--T

TuBIR-6a ACIFCSY-----RNYISSVPRILLTLHNVQS----PSCPAIRGEA-----------

NvBIR-10 YCFCCDCEL---SPLQPGEDLWGRHH-P-PQ----SRSKMILDLEKVSEPW-----

NvBIR-11 YCFYCDCKM-----SQHGEDLWIRHA-I-LA----PDCNYLRTR------------

NvBIR-8 YCFCCNGEL---SQLQPGEDLWGRHA-I-FA----PDCTYLRNRKGEKFINEARTK

NvBIR-9 YRFCCNCEL---SQLQLGEDLWGRHA-I-LA----PDCTYLRNQKGDKFLNEARIK

TuBIR-7 VCYSCGVLW---DMWTANDDPWVRHA-H-LS----PKCYHIYLHRGS---------

TuBIR-6b RCFHCGGIL---SSWKKNTNVWSVHA-Y-YF----PHCHYLYLKR-----------

TuBIR-8 TCYYCAGTI---ANWLPNDDPWNIHA-H-LF----PDCMFVYLKRG----------

TuBIR-15 TCFFCAGTI---ANWLPKDDPWELHA-L-HF----PDCMYVYLKR-----------

TuBIR-1 TCYFCAGTI---ANWLPNDDPWQIHA-N-LF----PDCMYVYLKRGR---------

TuBIR-2 TCYYCAGTI---ANWLPNDDPWNIHA-H-LF----PDCMFVYLKRG----------

TuBIR-4 TCYYCAGTI---ANWLPNDDPWNIHA-H-LF----PDCMFVYLKRGKN--------

TuBIR-3 VCYHCGLTA---FNWTCDDDPWIEHV-N-AL----PLCGFIYIMRGN---------

TuBIR-11 HCYHCDGAL---QNWQIGDDPWAVHA-N-AF----QNCAFIYI-------------

TuBIR-14 TCFHCANSL---LEWIKNDNAWVNHA-L-FS----PCCTFIYIMC-----------

TuBIR-5 VCFHCGIHI---RNWIPEDDAWIAHA-I-CA----PWCTYLY--------------

TuBIR-12 VCFHCGIHI---QNWIPDDDVWVAHA-I-CS----HWCTFLYIKCG----------

TuBIR-9 VCFHCGVHV---GNWLPGEDAWVSHA-K-FA----PWCTFLYIRKGF---------

TuBIR-16 ACFHCGVHI---CDWLSAENPWVSHA-K-FS----PWCTFIYIR------------

CfBIR-5a KCFECGIII---FGME-NKDPQEEHK-R-NY-----KCRFIREIPF----------

RpBIR-2 QCYYCEKEL---DCWEEEDDAFVEHK-N-HA----AYCPFVKM-------------

BmBIR-2b RCAWCGGELAPFRRFGSLGRPLEVHR-M-YF----PRCAHAAA-------------

TuBIR-13 ICYQCGCIV---NDWFENADPWLIHA-Q-SS----PFCFHLY--------------

CfBIR-5b YCFHCSVQM---NDWKPHDDPIQIHN-I-RC----PDCKFIKRLVEKR--------

CfBIR-4b TCFYCGQAL---QKWEATDDPKNEHI-K-WY----PDCAFINRLLAE---------

CfBIR-3a KCFDCYLEI---SKWPNGVNVVENHI-L-FS----PKCRFARKISC----------

IsBIR-4a RCFSCGTEC---GDWRETQGAVERHR-T-LS----PDCAFLRSML-----------

ApBIR-1 RCFSCFIEL---DGWESTDKPWEEHK-K-RALSLNPPCRFIEI-------------

ApBIR-10 RCFSCFIEL---DGWEPTDQPWEEHR-N-RNLSSKPPCKFVEI-------------

ApBIR-4 RCLYCPIGF---ECWGKDDDPYIEHK-L-AS----PECPYFKEKLDH---------

ApBIR-2a RCLYCSTEF---DYWQQGDDPVVEHK-R-QS----PQCQFFN--------------

ApBIR-3a RCTFCSSEY---DYWQPGEDPSAEHK-R-QS----PHCAFFND-------------

TuBIR-10 ECFCCLKQM---EGWSSNDDPWKEHL-D-HS----PDCEFAKTGV-----------

TcBIR-1 KCFLCNKSL---DCWAEDDDPWTEHI-K-HS----PKCSFAKKNK-----------

DpBIR-4 RCFVCLKDF---EGWEPNDIPKDEHK-R-LS----PQCPYV---------------

IsBIR-1 RCYVCFKEL---TGWEPDDDPVKEHA-R--S----MDCAFVQLRK-----------

SmBIR-1 KCFVCDKEL---DGWEIGDVPWHEHE-S-HS----PNCP-----------------

NvBIR-1 ECFMCCKQL---DGWEPDDDPWLEHK-K-HQ----PNCQFIKLDK-----------

BmBIR-1 KCFLCGKEL---DGWESTDDPWIEHK-S-HA----AQCAFVQL-------------

AgBIR-8 ACFVCGKEL---DGWEESDDPWSEHR-K-HA----PQCPFVKF-------------

DmBIR-1 TCFVCGKTL---DGWEPEDDPWKEHV-K-HA----PQCEFAKLS------------

DmBIR-3a ECHFCHVRI---DRWEYGDQVAAGHR-R-SS----PICSMVLAPN-----------

SmBIR-3a KCFSCSRTI---EEWNFGDQAIQKHA-N-LN----PNCDFVCN-------------

NvBIR-5a QCFLCGTRV---SEWNFGDQAMALHR-I-AN----PECPFVLDPI-----------

TcBIR-4a ECFSCHLTI---SEWNYGDQVMAKHK-T-LN----PSCPFVLNPTT----------

BmBIR-2a QCFSCGGKI---SEWNYGDQVMWRHR-R-ME----PNCTFVVN-------------

RpBIR-3a QCFSCGGRI---AEWNYGDKVMAKHI-S-LD----PRCPFVLN-------------

PhBIR-3a TCFSCGCNI---SDWNYGDQVMTRHR-N-LS----PNCAFVRDP------------

CfBIR-1a QCFLCGTMI---SDWNYGDQAMARHR-R-KA----PNCPFVVDPA-----------

SmBIR-4 RCVYCGNCL---ETWVRNDKPLLEHL-R-HF----PDCEFMKAV------------

CfBIR-4a KCFDCNIII---SDWKDID-PMAKHQ-Q-QF----PRCRVVRRIPCGNV-------

ApBIR-5 LCFCCNQGL---MDWEVDDDPWVEHA-R-WS----PLCSYVLLS------------

ApBIR-2b LCYYCSQGL---KDWEENDEPWTEHA-K-WA----QSCSFVQL-------------

ApBIR-3b RCYYCDQGL---IDWDDYDEPWTEHA-R-WS----NTCIHVLL-------------

DpBIR-5 VCFLCNVCL---ICWEPSDEPWSEHE-R-HA----ATCPLVKGDY-----------

BmBIR-3 MCFACNVCL---VCWEKSDEPWVEHE-R-HS----PNCSFVKGEYT----------

NvBIR-3 MCFTCSVCL---VCWEPTDESWSEHE-RLHS----PACPSVKG-------------

AgBIR-7 MCFTCTVCL---VCWEKTDEPWSEHE-R-HS----PECPFVKGEF-----------

TcBIR-2 MCFTCTVCL---VCWERTDEPWSEHE-R-HS----PSCPFVMGE------------

DmBIR-2 MCFTCSVCL---VCWEKTDEPWSEHE-R-HS----PLCPFVKGEYT----------

NvBIR-4 MCFTCSVCL---VCWEPTDEPWSEHE-R-HS----PACPFVKGEYTQNVP------

CfBIR-6 MCFTCNVCL---VCWEPTDEPWSEHE-R-HS----PACPFVKGEYTQN--------

SmBIR-2 MCFTCNVCL---VCWEPTDEPWSEHE-R-HS----PSCPFV---------------

IsBIR-3 VCFQCGGGL---YQWDDGDSPFEEHA-R-WY----PDCPFVRLSLGDA--------

AgBIR-6 HCFDCGLTL---GGWLRTDDPWEKHA-R-SS----PNCPFIENE------------

ApBIR-9 ECFCCGLIL---HNWERLDDPWIEHC-R-FN----PRCLYVLLM------------

ApBIR-7 ECFCCGLVL---QKWTKDDIPFVEHA-K-WN----PKCIFVLL-------------

ApBIR-8 ECFSCGLVL---HNWKKDDIPWIEHS-R-HN----SKCIYVLLSKGNHFVEH----

PhBIR-1 KCFACFKEL---SDWEKNDDPWQEHV-K-RG----SKCPFVI--------------

CfBIR-3b VCFYCGLIL---KEWTDYEDPWEAHY-K-WA----AICFYILTIKG----------

SmBIR-7 ECVYCFGRL---KQWAYNERPILEHY-L-WF----PYRPL----------------

SmBIR-5 ECVFCEGRI---ARWVKGDKPMIEHY-R-FS----PYCPLMRIVF-----------

SmBIR-6 ECIFCHIRL---DEWKMKDIPLFEHY-R-FS----PFCPLTLGE------------

IsBIR-5 ICFSCGGAL---FHWDEHDDPLIEHV-R-WY----PDCAYVLLCLGPQE-------

DmBIR-4a KCFFCGVEI---GCWEQEDQPVPEHQ-R-WS----PNCPLLRRRTTNNVP------

CfBIR-2a KCFECHVEI---CQWQPDDSPMVDHQ-R-WS----GRCRFVRNIP-----------

NvBIR-2a RCFECSTEV---CRWEQGDDPMVEHQ-R-WG----GRCRFIRKLPCGN--------

PhBIR-2a KCAFCKTQI---GFWEEGDDPNKDHL-K-LS----PMCPFLR--------------

RpBIR-1a RCVFCGVEI---GKWVPGDDPMVDHM-K-WS----PQCRFVRKLPVG---------

BmBIR-4a CCAFCKVEI---MRWVEGDDPAADHR-R-WA----PQCPFVRKQMY----------

AgBIR-1a KCYFCRVEI---GLWEPQDDVIQEHL-R-WS----PYCPLLK--------------

AgBIR-2a RCYFCHIEL---GGWDEHDVVIEEHL-K-WS----PDCRLMT--------------

AgBIR-4a RCYFCRIEL---GGWDEHDVVIEEHL-K-WS----PHCRLMTKRPT----------

IsBIR-2 ICYYCGGAL---RSWQKDDIPFVEHA-R-WY----PECTFVKLSMEPALYN-----

TcBIR-3a KCAFCKAEI---CSWEQDDEPLSEHV-R-WS----PNCPFAKEKSQNL--------

DpBIR-3 QCFTCLVVL---SQWHIDHDPDLEHR-R-HS----PSCEFVLNR------------

DpBIR-1a QCAFCRGVV---RDWEINDDPRREHQ-R-LF----PSCAFILG-------------

AgBIR-3 RCFQCDAGL---RDWLVTDDPWQEHA-R-CF----AECTFLRLV------------

TcBIR-3b ICFSCGGAL---RDWKDEDEPWEEHA-K-WY----PRCEFLVASKGHD--------

AgBIR-2b ICFCCGGQL---RDWLPEYNPWVEHA-K-NF----SGCPYLKLV------------

AgBIR-4b ACYYCGGNL---RDWLAEDDPWVEHV-R-NF----SECPYVKLV------------

AgBIR-5 ACFYCGGNL---RDWLAEDDPWVEHV-R-NF----SECPYVKLV------------

IsBIR-4c KCFHCDGGL---CNWERGDDPWEEHA-R-WF----PKCQFVLL-------------

BmBIR-2c RCFYCDGGL---GKWEAGDAPWTEHA-R-WF----PHCGYVLLLKG----------

RpBIR-1b ICYHCGGGL---KDWEETDEPWVEHA-R-WF----CKCPYVLL-------------

BmBIR-4b KCFYCDGGL---KDWESDDVPWEQHA-R-WF----DRCAYVQLVK-----------

NvBIR-7 TCYQCGGNL---KNWEPNQDPWIQHGYK-WF------STYF---------------

CfBIR-2b LCYHCGGGL---RDWEPEDDPWEQHA-K-WF----DYCSYLLMTKG----------

NvBIR-2b TCYHCGGGL---KNWEPKDDPWVQHA-K-WF----STCFYVRLVK-----------

NvBIR-6 TCYQCGGGL---KNWEPNEDPWVQHA-K-WF----STYFFV---------------

AgBIR-1b KCFSCGGGL---KDWEQEDDPWQQHA-I-WY----SNCHYLQL-------------

DmBIR-4b RCFSCGGGL---MDWNDNDEPWEQHA-L-WL----SQCRFVKLMK-----------

ApBIR-6b RCYYCDGGL---QNWEVADNSWVEHA-K-WF----PNCGFLNLV------------

RpBIR-3c RCFHCDGGL---QLWEKDDVPWLEHA-K-WF----SDCGFVLLT------------

PhBIR-2b ICFHCGGGL---KDWEKDEDPWVEHA-R-WF----SKCRFVFL-------------

DpBIR-1b NCFHCGSGL---RNWEPEDDPWLEHA-R-WF----PQCRFVML-------------

SmBIR-3c RCFCCDGGL---RNWEVNDDPWVEHA-R-WF----GKCCFLT--------------

DpBIR-2b KCFCCDGAL---RNWEPKDDPWVEHA-R-WF----SRCNFLVSV------------

DmBIR-3c RCFHCNIGL---RSWQKEDEPWFEHA-K-WS----PKCQFVLL-------------

TcBIR-4c RCFHCDGGL---RHWDPQDDPWTEHA-R-WF----PRCSFIKLV------------

PhBIR-3c RCFHCDGGL---RHWAPEDEPWSEHA-R-WF----PNCPFLL--------------

CfBIR-1c RCFHCDGGL---HGWQPMDDVWIEHA-Y-WF----PKCGFVLLM------------

NvBIR-5c RCFHCDGGL---RNWEETDDAWIEHA-R-WF----PKCGYVALVR-----------

SmBIR-3b QCFFCRGVV---GQWEDGDNPAIEHR-K-HF----RNCPFMSGY------------

DmBIR-3b KCVWCNGVI---AKWEKNDNAFEEHK-R-FF----PQCPRVQMGP-----------

ApBIR-6a KCAFCNICI---CHWEFGDNAVDEHK-R-HN----PDCSFIL--------------

PhBIR-3b QCAFCNGIA---CRWEVGDIPEAEHR-R-HF----PDCPFLL--------------

DpBIR-2a QCAFCCITI---SQWEAHDDPMAEHR-R-HA----PNCPFVLQL------------

RpBIR-3b QCAFCDGLV---GNWEYGDEPDVEHQ-R-HF----PACTFVSN-------------

IsBIR-4b RCAFCRGTI---HNWERGDDPLVEHG-R-HF----PCCPFLLDP------------

CfBIR-1b ECAFCKGIL---MNWKVGDDPEHAHK-L-NF----PNCDFYMRE------------

NvBIR-5b ECAYCQGVI---LKWEPGDDPDREHR-I-HF----PNCDFYMRDGA----------

TcBIR-4b KCAFCKGVV---RAWEPGDDPDQEHK-R-HF----EDCPFVLTE------------

*

**K. I35 Timp.**

AgTIMP-1 ---AETCSCLPQHPQTAFCDSQYVIVAQVLRKTASKNEAMDAY---KIAIKKEYKMSDEA

DmTIMP-1 -RPADACSCMPSHPQTHFAQADYVVQLRVLRKSDTIEPGRTTY---KVHIKRTYKATPEA

TuTIMP-1 --TSDACSCVYSHPQEHFCSSDFVVTLSI--QGDPKQLHQYNYLRYPVKVYKIYKGMDKT

CfTIMP-1 ----AACSCMQAHPQTKFCESDFVAVIKV-KKVLPVNEYEIAY---KVKINRVFKSNSKA

AmTIMP-1 ----VACSCMNSHPQTLFCNSDFVILVRV-KKMTNVNEFETAY---NVKVNKFFKANKTT

BmTIMP-1 ---ASACTCALEHPQTHYCKSDFVIVGRV-QKTFRGREDYDIY---KVKIRNVFKATDKA

TcTIMP-1 -QYSNACSCMGYHPQTQYCRADFVILARV--KRSTVLNSLKVY---KVRIRKTYKGSDKA

IsTIMP-1 ------------APRSH-----------------------------GIELLRLFRGGPKA

SmTIMP-1 CAVGDTCTCFPVHPQAHYCNSDYVLLVKV-NNSTIGNQTQATHRMIDVKIKKSFKANEKV

*. : : . :. .

AgTIMP-1 RQLLNHGKLYTSTMDSACGIKLKPSTLYAIAAN-----SEQVGLCDFIRPYDELSLVEKR

DmTIMP-1 RRMLRDGRLSTPQDDAMCGINLDLGKVYIVAGR-----MPTLNICSYYKEYTRMTITERH

TuTIMP-1 --SVRKGFIYTGSALSSCSPTLAKNTTYLMTGRIVNG-KPFVSICNFISEWSSLTYRQKK

CfTIMP-1 DIALMQNLLRTPSSSSMCGVTLQVGETYVLNGRIVSG-QALISSCGLSIRWADTTSRQRK

AmTIMP-1 YPALRKNILWTASSDSMCGAQLKLGETYVVSGRVIYGDKAHISSCGIAMPWRFVTSRQRK

BmTIMP-1 VAALRSGRLFTPPHESLCGVSLQPRETYVITGQVLHL-EAHIYLCGYIAKWREVTPRQRK

TcTIMP-1 TVALKSGRLLTASDEAMCGANLEAGRVYAISGQVNSL-KAHINLCGMAIPWRNLTRRQRK

IsTIMP-1 MWALAEGLLWTPGNDGLCGVSLHENVRYLVTGSLHGA-KPWVSACGFVRPWNSLTRKQRK

SmTIMP-1 NFAIKNGQIWTPMNDGVCGINLKPNAKYLITGKVEGG-KAFISTCDYYQEWSNLTPKQRK

: . : * . *. * * : . : *. : : :..

AgTIMP-1 GLAGVYRKGCKCKINHC-WDDKCHQR----LGSCNWTP-FAPKGICETSYGSCVPAGVTK

DmTIMP-1 GFSGGYAKATNCTVTPC-FGERCFKGRNY-ADTCKWSP-F---GKCETNYSACMPHKVQT

TuTIMP-1 GFRRNFGRSCGCKVTD--------------------------------------------

CfTIMP-1 GLRQLYQPGCVCDILYTHWRRKGAVLESSGGKRCLWES-TPGPQDCQEKHGVCIA-----

AmTIMP-1 GFRHLYHSSCMCKVRYTPWWIKGITLENTDGTECLWES-RPGPEECQKDFGICMY-----

BmTIMP-1 GFRLLYKQGCTCKVHETRRRTKS-------PNTCVTNY-----NECYERHGICLHD----

TcTIMP-1 GLKSVYKKGCDCRIEYC-AGRKCHKT----PDTCLLTNRF-----CHPKQAICLRQ----

IsTIMP-1 GFQRLYQQGCRCSVRLQPGPN----------TQCEWETAFRGVEDCQEQYAMCVPQ----

SmTIMP-1 GFKLLYKLGCECKVAYCPLAKRGHKCNVN-ANTCSWTTAFDKDGDCQGHYSICMRQ----

*: : . * :

AgTIMP-1 KNGAPIKCHWRR--

DmTIMP-1 VNGVISRCRWRR--

TuTIMP-1 ---AGSYAFYSGY-

CfTIMP-1 ---ASGSCSWMPSV

AmTIMP-1 ---RESGCYWTPS-

BmTIMP-1 ---RERRCHWTRAP

TcTIMP-1 ---KSRKCMWGR--

IsTIMP-1 ---ANSGCTWLGG-

SmTIMP-1 ---ITGYCQWN---

**L. I39 Alpha 2M.**

ApA2M-2 ------------------------------------------LVEGVEHTLTANEEAE--

TuA2M-1 -------------------------------------DTGFGFIQTDKPIYTPKEKVRIR

SmA2M-3 --------------------------------------SGYAFLQTDKPLYTPKDIVKIR

DpA2M-2 --------------------------------------SMTIFIQTEKPVYHQSQIVRFR

AgA2M-4 --------------------------------------TTTALVQLSKPIYKPGDVLQFR

AgA2M-3 ------------------------------------SKSISGLIQVDKPVFKPGDTVNFR

AgA2M-5 ---------------------------------------ISGLIQIDKPVFRPGDLVKFR

DmA2M-4 --------------------------------------QHTVLVQTDKSIYKPGDLVHYR

DmA2M-3 -------------------------------------FKPYIKIQTDKGKYKPGDTINYR

DmA2M-2 ---------------------------------------NWLYIQSDKATYKPGDKIQFR

DmA2M-1 -------------------------------------KKPSVFVQTDKATYKPADLVQFR

SmA2M-2 ---------------------------------------FSAFIQTDKAVYKPGQLVHFR

IsA2M-1 --------------------------------------SYSVFVQTDKAVYKPGQKVLFR

TuA2M-2 RLARFGVGPWGIGNYKLTTIGTGGLTFTNETKLVYDGKSFSVFIQTDKAIYKPGQKVLFR

SmA2M-1 ---------------------------------------YMVFIQTDKAIYKPGQPVLIR

AgA2M-1 --------------------------------------FCSVLIQTDKSVYKPGDTVRYR

AgA2M-2 ---------------------------------------FSVFVQTDKSIYKPGDTVRFR

TcA2M-2 --------------------------------------SYSVFIQTDRAVYKPGSKVLFR

DpA2M-1 ---------------------------------------HSVFIQTDKAIYKPGHLVQFR

RpA2M-1 ---------------------------------------YSVFIQTDKAVYKPGHMVLFR

NvA2M-1 --------------------------------------SYSVFVQTDRSVYKPGSKIQFR

AmA2M-2 ------------------------------------LKSYSVFIQTDRAIYKPGNKVMFR

CfA2M-1 -------------------------------------------------IVGPTD-----

PhA2M-1 --------------------------------------SYSVFIQTDKAIYKPGHKVLFR

ApA2M-1 ------------------------------------HKSYSVFIQTDKAIYKPGHKVQFR

TcA2M-1 ------------------------------------PNKKITFIETDRMTYKSKDTVRLR

NvA2M-2 ---------------------------------------LVTFVETDKAIYKPGQDVNIR

AmA2M-1 ------------------------------------HDSLLTFVETDKPTYKPGQDVKIR

CfA2M-2 ----------------------------------------ITFVETDKPVYKPGQDVNIR

ApA2M-2 VVVLSSC----PC----NRYVNYVVTTEGHVAI--WQKY--------------------K

TuA2M-1 LMRLDDDLK--PM----ADKVKLSIKNPHKIIMDEVVLESSKDNYFINHEFNIPPHFLQD

SmA2M-3 ILNLNEKLI--PN----DKPVLLEIKNPNASRVL-YEKFNPDDKGIIEYVFKFPI----F

DpA2M-2 TIPINTELR--AF----DDAVDVYMIDPRGFVVRRWLSRQSNF-GAVSLDYALSD----Q

AgA2M-4 VIVLGGDLK--PPA--PSVTATVIVHDPQRNVIRRWTAVSLQL-GVFEEQLQIGT----V

AgA2M-3 VIVLDTELK--PPA--RVKSVYVTIRDPQRNVIRKWSTAKLYA-GVFESDLQIAP----T

AgA2M-5 AIVLDTELK--PPA--RIKSVNVTIQDPHQNKIRGWPAAKLYA-GVFENDLQLAP----A

DmA2M-4 VLILDANLK--PAR--GYGRVHVDIKDSGDNIIRSYKDIRLTN-SIYSNELRLSD----S

DmA2M-3 VIFLDENLR--PDT--AKDEVVVWFEDSKRNRIKQEKHIKTTG-GVYTGKFELSE----F

DmA2M-2 VLFLDKNTR--PAV--IDKPIKIEIRDGDQNLIKSWKDIKPAK-GVYSGELQLSD----R

DmA2M-1 ILFLDENTR--PAK--IEKPISVIIIDGAQNRIKQLSDVKLTK-GVFSGELQLSE----Q

SmA2M-2 IITTDIILK--PVD---LKESSIYIVDSQGNRVKQWSNVTFEK-GVFEESLQL-------

IsA2M-1 VIVMDPYLL--PTV---TGAMNVHVTDAKGNRIHQWDRVLTQK-GIYSSELQLSD----Q

TuA2M-2 AIIVRPNLA--LAD---TGANYVYIEDAKSNRIKQWDRVFTTH-GVLSFDFQLSE----N

SmA2M-1 VIVVSPSLR--PTG---TERLDIFVTDGDGNRVKQWNRVFTQR-GVFTTEMPLSD----E

AgA2M-1 VLVLDRSMKLLPAG---DSGMMVYIRDGKGNRIKQWSNASLGECGVFQAELTLST----E

AgA2M-2 VLVLDPNTK--PLQ--KADNISVHINDAKANRIKQWKEGKLVK-GVFESELTLST----A

TcA2M-2 AVVLNSQLK--PAAEVRNELLHIFVTDGQGNRIKEWKGIQALR-GVFTGEVKLSE----S

DpA2M-1 VIVVNPQLK--PSV---VGSLDVYMTDGKGNRIKQWNRVFTKQ-GVFASELQLSD----Q

RpA2M-1 VLVLSAHLK--PVE---TRTIDVHITDGKNNRIKQWINAEPNR-GVFSGELELSK----N

NvA2M-1 CIVLDSRLR--PTA---NRQLEIYITDGQGNRIKQWERPRLHQ-GIFNGELELSQ----S

AmA2M-2 CILLNSRLR--PTL---ERLVDIYITDGKGNRIKQWIRPPVTH-AIFNGEIELSE----F

CfA2M-1 ----------------------VYIIDGDGNRIKQWNRPPVTH-GIYSSELELSQ----S

PhA2M-1 AIILNAHMK--PAT---HDFLDVFITDGKGHRVKQWNRTSTTK-GVFSGELLLSE----Y

ApA2M-1 AIVLNYHLK--PTV---TGALDIYITDGQGNRVKHWSRALTTR-GVFSSELQLSE----S

TcA2M-1 VLTLGNNLL--PIL--THKIPFVRIRNPLGVGVIVWENVTTEL-GLAQLEYQLPQ----D

NvA2M-2 ILTLRHDLK--PWI---KAIPKVWIENPSEVRVAQWTNVTTEN-GMAQLTFPLST----E

AmA2M-1 ILMLMHDLK--PWQ---KSIPEVWIENPSFVKVKQWTNVSTEN-GMAQLTFPLSP----E

CfA2M-2 ILMLKHDLK--PWK---KTIPKVWIENPSEVRVAQWTNVSTEN-GMTQLKFALSP----E

. :

ApA2M-2 PVVHMTKIMDQVD--------------ICRFN------------------------LTFN

TuA2M-1 PVKNKWTIIMAYGPEF-----------QVTSN------------------------ATFE

SmA2M-3 PVHGLWSASIKYGYQL-----------TGIKT------------------------VHFE

DpA2M-2 PTFGEWTIRVTAQ--------------GQTEE------------------------EHFH

AgA2M-4 PLLGRYTITVTVTGA------------NEIVS------------------------KTFD

AgA2M-3 PMLGVWNISVEVEG-------------EELVS------------------------KTFE

AgA2M-5 PLLGVWNITVQVGE-------------EQLVF------------------------KTFE

DmA2M-4 PRFGTWSIVVDVS--------------DQEHT------------------------QTFE

DmA2M-3 ATLGSWSLHVQNG--------------DQHHDGGIYFGGRKQFGGFGHRWHRSDELVNFE

DmA2M-2 PVLGNWTVTATVQD-------------EGKVT------------------------NVLV

DmA2M-1 PVLGTWKISVSVDG-------------DNRET------------------------KSFE

SmA2M-2 ------------------------------------------------------------

IsA2M-1 PVLGDWAIHVDIL--------------GQKYS------------------------KNFT

TuA2M-2 PVLGDWTINVEID--------------RKIFK------------------------KTFT

SmA2M-1 PVLGDWNITVNILVINKLKFYAKIEEIDQKYQ------------------------KSFT

AgA2M-1 PVLGEWTINVEVV--------------GLKES------------------------KTFD

AgA2M-2 PVLGAWTINVEVL--------------GSKHN------------------------KVFE

TcA2M-2 PVLGNWNISVKIH--------------GQTFS------------------------KSIE

DpA2M-1 PVLGDWNITAVVS--------------GQSFS------------------------KHFQ

RpA2M-1 PILGDWKIVATVG--------------GQIYA------------------------KVFS

NvA2M-1 PVLGDWEIVAEIG--------------GQTFK------------------------KAIQ

AmA2M-2 PVLGTWKITANVG--------------DQTFE------------------------KDFE

CfA2M-1 PVLGNWKIIANVG--------------DQTFE------------------------KEFE

PhA2M-1 PVLGDWKIVVNVV--------------DQVFS------------------------KTFQ

ApA2M-1 PVLGDWNIVVTVL--------------DQVFH------------------------KSFL

TcA2M-1 PIEGKWKVEI-----------------GEDF-------------------------RAFE

NvA2M-2 PSSGIWRIKVEKNRP------------QLVHT------------------------STFE

AmA2M-1 PSLGSWHIKVMKKKPYP----------NLIHS------------------------TTFK

CfA2M-2 PSPGAWKIKVEKTRSQP----------QLIHT------------------------TVFE

ApA2M-2 VDPVMAPTSHLLVY---YTTEKGETINDVISFNVKQTDP---KVKI--SLKDNKNNW---

TuA2M-1 VREYILPLFQVTLKSLQFITPSTSIVNGSVKATYLTGKPVTGTVRF--KF----------

SmA2M-3 VKEYELPTFSVKIEPPKVILKNQKLIETRITANYVYGKPVVGLLNI--KFGIKTVEGIVV

DpA2M-2 VEEYYQTRFEVNVTMPAFYFDSDDFIHGTVMANYTSGAPVRGNLTLKATFKPIRATP---

AgA2M-4 VREYVLPAFEVAVKARAVPLEKHQRLNLTLSARYYTGQPVRGVATV--ELY---------

AgA2M-3 VKEYVLSTFDVQVMPSVIPLEEHQAVNLTIEANYHFGKPVQGVAKV--ELY---------

AgA2M-5 VKEYVLTSYDVQVMPSVMPLVEHQTLNLTIVANYHFGKPVQGVAKV--ELY---------

DmA2M-4 ILDHILPKFVVDIDTPKHAIYKDGKIAATVRAHYAFGQPIVGEATL--SIYPTFFG----

DmA2M-3 VEKYVLPKYSVKMDATQQVSVRDGEFNVVLKANYTYGKPVNGKVLV--NVHLDSTSSWEN

DmA2M-2 VDKYVVPKFEVVVLTAKNVAASAGYIRATIKARYTFKKPVKGHVVA--TIE---------

DmA2M-1 VDKYVLPKFEVIVDTPKAVVIADKVIKATIRAKYTYGKPVKGKATV--SMERSYGYF---

SmA2M-2 ---------------------------------YTYGKPVKGKVLL--NVTDYYCKW---

IsA2M-1 VAEYVLPTFEVRVKLPAYATYNKSEVVATVSATYTYGKPVKGTVTL--TVAPRTRYH---

TuA2M-2 VAEYVLPTFSVDVTLPNYVTYNKSDLIANVKATYTYGKPVTGEVTL--TVQPRIRYG---

SmA2M-1 VAEYVLPSFDVRIDLPNYATFTESDIVATITAKYTYGKPVKGKAVI--MVTPLVRSP---

AgA2M-1 VDEYVLPTYEVTVESPGYTFLDDELLKVVVNSKYTYGKPVAGELTV--SVKLASSMC---

AgA2M-2 VDEYVLPKFEVTVESPGITTFKDGKVKAIIRSKYTYGKPVKGEATV--SVSPEFQFH---

TcA2M-2 VAEYILPKFIVNIQAPKHMTFKENVLVANIQTQYNYGKKVKGEATV--TVYPTIFSG---

DpA2M-1 VAEYILPKFQVTIDLPTYLTFNESKMVATVKAKYTYGKPVKGNVTI--AAYPQYRVS---

RpA2M-1 VAEYVLPKFEVTVNIPKHITFKDSKFGATVRAKYTYGRPVKGEATI--SVAPSYVSD---

NvA2M-1 VAEYVLPKFEVTIDSPPHATFKEGKITVLVHAKYTYGKPVKGEATI--TAFPDIYSG---

AmA2M-2 VAEYVLPNFEVTIDASKHFTFKESKITATIYAKYTYGKPVKGEATI--TAYPDIFSG---

CfA2M-1 VAEYVLPNFEVTIDSPKHVQFKESKITVFVHARYTYGKPVKGEATI--TAYPDIFSG---

PhA2M-1 VAEYVLPKFEVIIDSPKHATFKDSKVVSKIKAKYTYGKSVKGEATV--TAYPLYYSG---

ApA2M-1 VAEYVLPKFEVTIEVPEHTTFKQSVVSATIHAKYTYGKPVKGEATV--SVYPEYYSD---

TcA2M-1 VSKYVLPRFKVQILHPK-IIYIGSRVAIKVCGRYSYGEMVKGSAFI--RLSSIFPNF---

NvA2M-2 VRKYVLPRFQVTIGAPSYILADAPNATWKVCVRYSYGEPVKGKLLL--SLRPQTPIW---

AmA2M-1 VEKYVLPKFQMTINSPQYILANVENVTWNICVKYSYGKPVKGNLLL--KLTPQTPSW---

CfA2M-2 VKKYVLPRFQVTVTSPGYILADAENVTWNICAKYSYGKPVKGRLLL--KSTPQIPIW---

ApA2M-2 -----YPEELMELNLMAEENSLVCLIGGRGTENIS---PLRNMDD---------DTDLLE

TuA2M-1 -----KIRDSTNRSIGIGQTDELELVKGETSYKFST--NEFSESGVEVSM---IIGSVLV

SmA2M-3 EIGSLYLQPLK---------------EGERWISINVE-RDIKQKN--IVWFPEVDGSNLL

DpA2M-2 -----LIPDRPGSIRDSVIERNLTFKEFRGFYTFKYPMAELQRMVPK------LDGIELR

AgA2M-4 -----LEDDKL----DQ---RRVVGVYGAIQLDLPFN-EHLSVYD---------DSQDVR

AgA2M-3 -----LDDDKL----KL---KKELTVYGKGQVELRFD-N-FAMDA---------DQQDVP

AgA2M-5 -----LVDDTL----DQ---KKELTMYGMGQVELRFN-ELLELYE---------DQQDVR

DmA2M-4 -----SLQPFV----NDLITRKVVPIDGNAYFEFDIE-NELHLKQ--------DYERQYL

DmA2M-3 VDGKTVQTDYP----GHSVVGTADMVGGKAKLTMDLK-DFASYLPHK------TSSSYAQ

DmA2M-2 ---------------GSST-EQSLPIDGEVNVEFPIS-ATAKR--------------LLK

DmA2M-1 -----GDLNAN----GNKQ-EKTIDVDGKGHVEFDII-HWAQRGQ---------YLPPIK

SmA2M-2 -----PCSPYN----MKPF-STTTSIDGTADIQVNLV-RDLALPDWM------RHDRRFD

IsA2M-1 -----QLRPRP----YEQY-QTKAEIDGSVDIPVAVV-RDLSLKT-D------FFRRDIE

TuA2M-2 -----SLTTRP----LEQY-QVKAKLDGSVDIPVDVV-RDVKLRK-D------FYDREIE

SmA2M-1 -----QIRTYY----EDPL-RKTVEIDGKVDVPFNLQ-QELNIKD--------DYHRMVR

AgA2M-1 -----FRREPT----ETSICQKVLPIDGKTDVEFNLK-EILSSKT---------YIRELT

AgA2M-2 -----YVQPFA----KDVITRKVIPIDGKGSVEFDLR-EDIHLEG--------DYSRNIV

TcA2M-2 -----VIQPIF----QNPI-RKVIPIEGSATVTFDIA-KELKLTD--------EYERVVM

DpA2M-1 -----YIQPFF----TEPV-RKTVQIDGTVDVDFNLF-KELKLVD--------DFERDIR

RpA2M-1 -----ILQPIF----MQPV-IKTIPIDGKAVVEFDIV-KEIKLNQ--------DFERLIV

NvA2M-1 -----VLQPIY----SPPI-RKTVNIDGKTTVDFDIA-NDLKYDD--------DYKRPVV

AmA2M-2 -----VIQPIY----QQPV-RKVIPINGKVIVDFDIY-NELKLTD--------EYERPVM

CfA2M-1 -----VIQPIF----QQPV-RKVVPIDGKAIVDFDIL-SELRLTD--------EYERPVM

PhA2M-1 -----FIQPIF----ENPI-RKVVPIDGKAIVEFDIV-KELKITD--------DYERTIQ

ApA2M-1 -----LIQPIY----QNPL-RKVVPINGKTVVQFDVV-KDLSLND--------EFRRIIT

TcA2M-1 -----KTFQSL----------KKMD---EGCAEFVLTPTDFSYFSIKKLFPLSDPKISVL

NvA2M-2 -----KRKQTV----ADINYEETLDAKSDGCMNYTVSAQVLGLPHWK------VAPNNVV

AmA2M-1 -----TRLPNL-----PAIRYETKLDKGDGCTDFVLSGSVLGLAHWK------MDPNNIV

CfA2M-2 -----RRKPNL-----PEIHYETELDSSDGCTEFVLSGAVLGLAQWK------VAPNNIV

ApA2M-2 SGLLFLEKRLDGKVTTSRQSDLYPR---------------------HHSFLDTFSMD---

TuA2M-1 VEATVIEAATRQKVKHIDSSTYFVSSPYKVSFESSFR--VFRPEHPIRLIAEIYDVH-NQ

SmA2M-3 IEAEVIEKATGNRETAIDYSTIFSYSPFVVSFPRSSL--DFKPGVPYQVQADVKFVN-GK

DpA2M-2 ITAIVGDRYREDYVEGFSTARIYNS-SLKLNFLGGSP-QVIKPAMPFTCFLAVSFHD-GS

AgA2M-4 VHVAFTQDETNRTIYKEQRITVYKL-PYRVELVKEQP--EFRAKVPFDCQLRVRYQD-GT

AgA2M-3 VKVSFVEQYTNRTVVKQSQITVYRY-AYRVELIKESP--QFRPGLPFKCALQFTHHD-GT

AgA2M-5 VKLTFTEQHTNRTVVKEQAITVYKH-PYRAQLTKESP--QFRPGTPFKCTLTLIYHD-GR

DmA2M-4 LDALVEEKSTGSVQNYSTVLTLHLN-HYRVEAVKVPS--YYIPGVPFEATARIARND-GG

DmA2M-3 ITATVEEDFTGVKLNETGGVQLYPY-RYEMSCTDYSSCFSFKPDKEHELNFKITYVD-GS

DmA2M-2 ITAIVTEELTDIKHNGTAYVTVHQH-RHKLEDLFWPT--HYRPGVSSEFKTVVRNLD-GS

DmA2M-1 LFAVVTEELTGNKQNATATVVLHQQ-RYSIEPYERPE--HFEANKSFIYQVVVKNVD-GS

SmA2M-2 VFAVVTEELTGRKQNGSGDITMYDS-QYKLSFSESGN---FKPGLKYTITLQVSLQD-GS

IsA2M-1 FFALVEERLTGRKYNSTSYLTLHDK-EVKVELVKTSE--TFKPGLKYTCFLKVAYQD-DT

TuA2M-2 FFALVEESLTGRKYNKTAILKVYDK-DINIELIKTAP--YYKPGLKVICYLKVAYRD-DT

SmA2M-1 FEVIVTESVTERRENATGVIAMFKY-KEKIELIKHSE--TFKPGLKFSAFVKVADND-DI

AgA2M-1 IEAEVCETLTGRTQKGSTTVQLHDE-RYQVRMIEESS--YF-PGLPYNAWIQVTNLD-GS

AgA2M-2 IEAVVEEELTGRKQNASAKVMIYDR-RYKMELVKSDD--NFKPGLPYTAWLKVSYQD-GA

TcA2M-2 VDVTVEEASTGRRQNNSVEVHLHKY-NYKMDLIKTAD--YFKPGLKYTAYVKVSSHE-GT

DpA2M-1 FDVTVIEGLTERKQNMSSLLTLYKY-KYKMELIKTSD--SFKPGLKYTAFLKLAYQD-NT

RpA2M-1 FDVTVKEELTGRKQNTTAEMWVHKH-KYKMDLIKTSQ--YFKPGLKFTAYIKLAHYD-GT

NvA2M-1 IEVAVEEAVTGRRQNNSMQITLHKH-KYTMELLRTAE--YYKPGLKYTAFLKVTYHD-GS

AmA2M-2 LDVTVEESLTGRRQNTSTHITLHKN-KYTMDLIKTSE--YYKPGLKYTAFIKITYHD-GT

CfA2M-1 IDVVVEEALTTRRQNTSIQITLHKH-KYTMELIKTSE--YFKPGLKYTAFLKLTNHD-GS

PhA2M-1 VDVT----------------------------------------------IKLVYHD-GK

ApA2M-1 FDVTVEEALTGRSQNTSANVMFHNH-KYKMDLIRTSE--YFKPGLKYTAFIKMSHHD-GT

TcA2M-1 ITATVTEHGTDKIELDATKSVISLK-PYGLKFAKKAM---FMPGLPYQGFLQLNNVNMDL

NvA2M-2 LLANFTEAKSGVVETATSRSPVMHQ-PLKLEFSPHTL-KYFKPGLPYHGKLRVLRADASS

AmA2M-1 LIAEFTEAGTGIVETTISRTVVLHE-ALKLEYEHYTP-KYFKFGLPYHGKLRVLRYD-DT

CfA2M-2 LIANFTEAGTGIVETTISRTVVVHQ-ALKLEFLPYTP-KYFKLGLPYHGKLRVSRHD-DT

ApA2M-2 -------Q----LW------TWKC-----VNFTSDVI--------KKGMNIHAPSKPGHW

TuA2M-1 PVVGIPVR----LS------VFSS---------------------RMVTKDVISDDLGRV

SmA2M-3 EAPKVSVSIISAVS------KKGG---QVFRH-------------LPRPLASLTNELGRV

DpA2M-2 ILPAERFRYDR-LE------IRPT-----VQFRSSASRTL-----EVREAKALPEQPGIW

AgA2M-4 PAKGAAFE----VK------VEGA-----YTTD-------------RRVAYTSD-AAGVI

AgA2M-3 PAKGIS------GK------VEVS-----------DV--------RFETTTTSD-NDGLI

AgA2M-5 PAGHVPFF----VN------VEGE-----------DV--------DHQQTYTTG-RDGTI

DmA2M-4 QLRDFNPQ----IT------AYLT-----NVYGSSEM--------YNRTAYSLD-ASGEI

DmA2M-3 LITDTKSV----VKAKFTEGIRRNYAFYAFGTDHQEPELPTIEKKTFVFESHLN-ASGVA

DmA2M-2 PVMDSSKM----VN---------------FNVLCCQV--------SKNFSASL--QNSIA

DmA2M-1 PVTNSAKN----VK------IGFDKSYSYFHEPSPKT--------RINFEAPVN-ENGIA

SmA2M-2 PILDD-GE----IS------INYF-----SSWN--SP--------GKTLNYTIP-KNGEI

IsA2M-1 PVHDAVNQ----LT------LYQG-----FNFN-EDL--------WKTSRHWVP-ANGVV

TuA2M-2 PVEDNGPP----VK------IMYG-----YGYD-EDA--------YNSIIEEVP-NKGII

SmA2M-1 PVNDSTNP----LI------IRYG-----YGHD-DSL--------YQTKQFKIP-TNGSI

AgA2M-1 PVQDGAKE----VE------IVLR----NYNID------------LHKQSSTLD-DKGMA

AgA2M-2 PVQDQTNP----VE------VKQS------SFE--ST--------TSVQNYTLD-QNGMA

TcA2M-2 PLRSENRE----VT------VRYG-----YSRA-DEV--------YVTEKHRLD-KNGVA

DpA2M-1 PIQDANGV----VI------VKHG-----FSHN-QDE--------YNRTEYPVP-RNGIL

RpA2M-1 PVSDQTNP----LK------VKSG-----YSYE-SSE--------YIESHYKIP-PTGVV

NvA2M-1 PVVDNTNP----VH------ISYG-----YTYN-SED--------LHNITRMLD-KNGMV

AmA2M-2 PVRDNKNP----VI------IKYG-----YSYDNQSI--------YTNITGMLD-ENGMV

CfA2M-1 PVQDTKND----VL------IAYG-----YSYD-RSD--------YFNITKMLD-QNGMI

PhA2M-1 PVIDDVNP----II------VKHG-----FSYD-TEK--------YTAKEYKLP-LNGLL

ApA2M-1 PVYDDRNP----VK------VWHG-----FSHE-TEK--------LDESKHMLP-RNGLI

TcA2M-1 RG--QVIEICYNIA------IK-------KSWNYLNN--------EQCSNFTLQGNEKLI

NvA2M-2 PAPNEKIQVC--LR------IRRK-----DEWQ-RSV--------VECRNFTSSNADGFL

AmA2M-1 PAPNEKIQIC--LK------VRGK-----IEWE-KDV--------VDCRDFRSS-TDGFV

CfA2M-2 PAPHEKIQLC--VR------VRGE-----DDWL-RVV--------VECRNFTSS-SDGFV

ApA2M-2 KLRMLTVGSTGL-----KITDTLDIKVT--------------------------------

TuA2M-1 TVDYDTTTQDKK----------IIFEVR-----------------TRDAKLKEDEQSFSR

SmA2M-3 MFELGTGIEDQE-----LE---ITVTTE-----------------EEGLSVENQATNVLN

DpA2M-2 EFTIDLRSELAQ-----TTGSNVGTGDKRVKDLVNEISALKLIANYKDGYGERATAEMLA

AgA2M-4 KLTLQPEASSES----------IDITVRVFAREV-----------FLEKSTLKRTGGMNF

AgA2M-3 KLELQPSEGTEQ----------LSIHFN-----------------AVDGF-FFY-EDVNK

AgA2M-5 KLLMRPTELTET----------IDITVS-----------------EDNSE-FTYTERIEK

DmA2M-4 KMKFTVPIGDRD-----EF---HSIIVD-----------------YQGVI-SEV-GKIPS

DmA2M-3 PFKVVLPDLPDIANFTRYYSIELEFVDE-----------------KRDLY-TTY-PYREP

DmA2M-2 TEHIMLP-ET-C-----QS---CLVTST-----------------FDTAE-NIE-RYIYK

DmA2M-1 TFNVRLP-DSDS-----RY---YRIFAS-----------------FDGSE-NTI-GSISK

SmA2M-2 DVEIIPP--EST-----EN---LRFETS-----------------YKGAR-T-T-SYVSK

IsA2M-1 RLELFPPNDNAT-----VV---LGLRAE-----------------FRGQT-HYL-EGIYP

TuA2M-2 KFE-FTAVNNSH-----SV---IGMKAE-----------------YKGQV-YNL-NSFEM

SmA2M-1 ELEIYPPLADNI-----QR---LIIIAK-----------------YKSIE-QYF-PPIRR

AgA2M-1 QLNVKLD-ELDF-----DY---VSVEVK-----------------YRGKD-YYV-QGITK

AgA2M-2 KLEIN-T-EVNS-----SY---INVVGV-----------------YLGQE-FYL-HGISK

TcA2M-2 KLEYYTPVNVTN-----TTA--LRIEAQ-----------------YQDLK-ERI-SPIPA

DpA2M-1 ELNFYPPVDENV-----YT---LGIETQ-----------------YQDLV-EWF-STINR

RpA2M-1 ELTFYPP--KNI-----SV---LGIEAE-----------------YLDIK-EWF-STVNA

NvA2M-1 ELDFYPPLSMPD-----KIFRPLRIEAQ-----------------YLNLH-EWF-PSTNP

AmA2M-2 KLDFYPP-KTNH-----NISYPLNIEAQ-----------------YLNLY-EWF-PSTNQ

CfA2M-1 QLDLYPP-KQKD-----NISFPLNIEAQ-----------------YLNLH-EWF-PTTNS

PhA2M-1 ELNFHIN-DPNV-----TT---LGIEAS-----------------YLDLT-EWF-STVSK

ApA2M-1 PLVYY-P-QINA-----SV---IVIEAE-----------------YLNQR-EAL-STILP

TcA2M-1 PFHILPL-KNNV-----IH---LQLNARSLN--------------FTNIV-DNF-LVVR-

NvA2M-2 DFIVPPQ-NKNI-----VL---LSFVATAVNYPTKYYSPDKRWRVFVDQP-SAY-IDVEP

AmA2M-1 DFVVPPQ-HKNI-----VL---LSFVATAVDYPTTYYSPEQRWRVFMNQP-STS-ITVNP

CfA2M-2 DFVVPPP-HRNI-----VL---LNFIATGVDYPTKYYSPDTRWRVFMDQP-SAH-IVVNP

ApA2M-2 --------STLEV-DVRTPVEINV-------GETVQTDIYVA-----------------N

TuA2M-1 LLVEPSNQTSAALTLVEKNDRFKV-------GDKYTNSIIFE-----------------G

SmA2M-3 VIKFKSPDTTSYV-WIAAPPEEMIFTV----GRTFQTQVTVFPAA--------------G

DpA2M-2 ITHYSIGDKHIKV-LTSTR-SPKV-------GEYLILHVRTN-----------------F

AgA2M-4 VS--RTGKRWQRGAALRAHKQAVKI------GKPFKLKVTCN-----------------E

AgA2M-3 VE--TVTDAYIKL-ELKSPIKR---------NKLMRFMVTCT-----------------E

AgA2M-5 VH--ADTNVFLKL-ELKSPIKL---------GKLIRLMVTCN-----------------E

DmA2M-4 KH--LHSKNYITA-KVLND-RPTV-------NQEISVVVRSF-----------------A

DmA2M-3 KQIENPSSEEEKE-WFRAEVQRPKDVWNLKIGQEYQVILNSS-----------------R

DmA2M-2 LN------KPLMI-AINTK-KPQL-------RKLLKINIISD-----------------T

DmA2M-1 FEPTPMSREPLKI-QVNTK-KPRL-------GEQVSFDVVSI-----------------E

SmA2M-2 AQ--SLSERYLQI-TLLSQ-KPKI-------GDEVEVLVDST-----------------K

IsA2M-1 AR--SPTRSFLQA-WVTTE-DPMI-------GDLVEVEVNST-----------------Q

TuA2M-2 SL--SPSSNYIQV-AIPDGYQAKI-------DETLRLSVTTT-----------------E

SmA2M-1 AE--SPSNTYIQA-VLMTE-KAKVVFKWFAGGEEVLIHVNST-----------------A

AgA2M-1 PR--DYEEALMRV-RLSEK-EPTA-------GKDLTFDVACT-----------------K

AgA2M-2 AE--SDVDSYIRA-QVLTE-MPLV-------GKDVLVEVTST-----------------S

TcA2M-2 AV--SYSNTFLQV-SLETE-RPIV-------NLDVEILVNCT-----------------E

DpA2M-1 AQ--SPSNSFIQV-ILKTE-NPKV-------NEEIAIEVNST-----------------A

RpA2M-1 AV--SPSDTFIQA-IIKTK-KPKV-------NNDVVLVVNST-----------------E

NvA2M-1 AT--SRSESYIQA-MLRTD-KPKV-------NEYVEIEVNST-----------------H

AmA2M-2 AM--SQNNEYIQA-ILKTE-KPMV-------NQDIEIEVNST-----------------I

CfA2M-1 AT--SLSNEYIQA-VLKTE-KPMV-------NNYVEIEVNST-----------------A

PhA2M-1 SM--SPSETYLQT-TLMTE-NPKT-------NQDIVIFVNST-----------------S

ApA2M-1 AH--STSNTFMQA-TVLTE-RPTV-------NKDVEIQVNST-----------------E

TcA2M-1 LF--SPSLTYI---TIDQIHHSNNC------KSIQQFAVQYTTEKLK---------EH-E

NvA2M-2 WY--SPSSSYL---SVTRGSQPLVC------GEKYSFNVMYTSGTSSTVNATNLSPDDTE

AmA2M-1 WY--SPSDSYL---TVARGNQPIVC------GEKYSFNVMYTTSSNMN-----------E

CfA2M-2 WY--SPSDSYL---AVARGYQPIIC------GEKYSFNVMYTVPAKSKTN---------E

:

ApA2M-2 NVNSCMDVN---------------------ALLSLSEGAVFSSS----------------

TuA2M-1 ST-FIFSQA---------------------YYITVVRGRIDKLERIP-------------

SmA2M-3 DI-KLH------------------------YMV-VSRGMLLLQGES--------------

DpA2M-2 YA-ETFN-----------------------YIV-VSKGIVLVTG----------------

AgA2M-4 EL-SFFM-----------------------YYV-VSRGVIVDSAFLQ-------------

AgA2M-3 RM-TFFV-----------------------YYV-MSKGNIIDAGFMR-------------

AgA2M-5 RM-TFFI-----------------------YYV-ISKGNIVDAGFVR-------------

DmA2M-4 PI-KYFM-----------------------YQV-VGRGDIILSRNVD-------------

DmA2M-3 PL-KYFV-----------------------YNI-VGRGNILETKRVD-------------

DmA2M-2 YL-PYFI-----------------------LTV-VARGNIVLSLFQE-------------

DmA2M-1 DL-PYFV-----------------------YTI-VARGNVILSDYVD-------------

SmA2M-2 ELNDNLI-----------------------VQV-IGRGKILHHESIP-------------

IsA2M-1 PL-DHLV-----------------------YEV-MGRGDIVFAQTLP-------------

TuA2M-2 PI-ESFV-----------------------AIV-VSKGKIVSTETFY-------------

SmA2M-1 PM-PSFNVVAGLGALLKKIAKCCDSVALQIYQIVLGRGDIVFADKVE-------------

AgA2M-1 PL-QCVS-----------------------YSL-LARGELLAGGAVK-------------

AgA2M-2 PM-KYFT-----------------------YQL-LGRGDVLLSNTIA-------------

TcA2M-2 RL-RYIS-----------------------YVL-MGRGDVLNANTFQ-------------

DpA2M-1 PL-DSYI-----------------------YEV-MGRGNLVVARTVQ-------------

RpA2M-1 PL-RYVT-----------------------YLV-LGRGDVVTANSVR-------------

NvA2M-1 PL-KYLS-----------------------YQI-LGRGDVLNAASIQ-------------

AmA2M-2 PL-KYIN-----------------------YEI-FGRGDILDAGSIY-------------

CfA2M-1 PL-KYIS-----------------------YEV-LGRGDILDAGSIY-------------

PhA2M-1 PL-KYYN-----------------------YLV-LGRGDVLISNTVQ-------------

ApA2M-1 SL-QYIT-----------------------YQV-LGRGDVIVASTVQ-------------

TcA2M-1 NV-TFF------------------------YMI-KSRGQIFKLRKITHNVRKSSPNYSTE

NvA2M-2 PI-SFH------------------------YSI-NSKGDLLVFGHVKYKPRKDTLLDYSE

AmA2M-1 TI-SFH------------------------YSI-NSKGSILIYGHVKHKPNRDTILNYFE

CfA2M-2 SI-SFH------------------------YSI-NSKGDLLIYGHVKHKPTRDMVLNYSE

: * :

ApA2M-2 ------------------------------------------------NQPFVAEKMRLG

TuA2M-1 -----------DND------NFGFT----IEPHMAPSFRLIAISYR--YDRVVSDSLLIN

SmA2M-3 -----------ASERNAVVHPIQFS----VTADMSPSARVIVYIIK--NNQIIADSMKID

DpA2M-2 -----------SQSSSPSVTTLSIP----LSAEMAPVATVVIYHVAK-YGEVVADSLTIP

AgA2M-4 -----------PKK--VTEHFIEIE----ASDQMVPRSKVIVVTVA--KNVVLCDFVDID

AgA2M-3 -----------PNK--QPKYLLQLN----ATEKMIPRAKILIATVA--GRTVVYDFADLD

AgA2M-5 -----------PNR--QTKFMFQLT----ASEKMIPKAYIFVATVS--QDVVVWDSLEID

DmA2M-4 -----------VAP--GTFHTIKFL----ASFAMMPRANLLVYTVI--DGEFVYDEQVIQ

DmA2M-3 -----------LAE-PQTTVNVTIK----PTFLTTPYGRVYFYYVDE-TGEFRYTEETFS

DmA2M-2 -----------MKE-KKKSQEIEFE----PTFALVPQATIFVHYII--DGVLMSDEKTVD

DmA2M-1 -----------VPD-GQKTYTVKFT----PTFSMVPKATIYVYYVV--NNDLQFEEKTID

SmA2M-2 -----------PSK--AKSQTLKFK----ITSEMAPKIRVIVYYATS-CGEVVADALDFS

IsA2M-1 -----------ASG--VRTYRFSFS----TSFRMAPRARVLVYYVRK-DGELVADAVNFD

TuA2M-2 -----------FKS--GKQHVASLS----ITHSMAPKARIIVYYVRTANSEIVADSVAID

SmA2M-1 -----------ANG--QKTIAVKFV----ATNNMSPRTRFIIYYTTS-SGEVVADGLSFE

AgA2M-1 -----------GSE-ASTTISITIP----STFAMVPRAKLLVHYISS-AGYIVSSYDTVE

AgA2M-2 -----------VPE--SKTQSFKFP----ATFAMVPRAKLVVYYIAP-NGDMVSDSKVIT

TcA2M-2 -----------VDN--MHEYRFHFT----ATHAMVPVAHLIVSYVRD-DGELVGDALDIE

DpA2M-1 -----------AGN--QRSHTFRFQ----ATAAMAPVARVVVYYVRA-DGEVVADALNFD

RpA2M-1 -----------VPE-GAKTVEWRFL----ATYNMAPIAHIIIQYVKA-DGEVIADSLDIQ

NvA2M-1 -----------ISD--RYTATFKFL----ATYVMAPIAHVVVYYVRE-DSELVADSLDVE

AmA2M-2 -----------VQN--KHTTNFKFL----ATYVMAPTAHVIVYYVGN-DGEVIADALDVE

CfA2M-1 -----------VQD--KYTTSFKFL----ATYVMAPTAHIIVYYVKT-DGEVIADSLDVE

PhA2M-1 -----------VPE-MSKTHKFLFV----ATPAMAPTAHVLVYFMTE-SGEVVADGLNVD

ApA2M-1 -----------IPNAGQHTAVIRFL----ATYAMAPTAHVIVQFVKD-DGEVVADAIDVE

TcA2M-1 FKDILGASHKHTKASSIAKFTLKFK----LDEKIFSNYQLLIYYVTP-EGELASANKEVE

NvA2M-2 FRHVLGAGAGSKPNPSVHRFPLSVK----ITASMAPVSELLLYYVRP-DGEVVTASHSIE

AmA2M-1 FHNLLGTIESSANKTNKEAIVHRFPLSVKVTPSMAPVSELLLYYVRS-DGEIVATTYTIE

CfA2M-2 FRNLFGAVETSGNKTNQDTIVHRFPLSVKITPSMAPVSELLLYYVRP-DGEIVATTYSIK

. .

ApA2M-2 AYGA--TSLI--------VRITALKEGL------KNLTVDISGYVSEK---CHLINDNKK

TuA2M-1 VDPPECSLNLTY------TNSKGATNDI---EPGENGTLIVSANQSENRRQISIVGVDEA

SmA2M-3 VHQTCKYNNG--------DDLKLSATATGFYKPGQNVKIEITGERDSF---VGLLAVDEA

DpA2M-2 VNGI--SRNN--------FTLTLNPMKD---KTGDTVEVVVLGDPGSY---VGISAIDKG

AgA2M-4 FED---LRNN--------FDLQIDRTEI---RPGDQLQLNMRGPPGAF---VALAAYDKS

AgA2M-3 FQE---LRNN--------FDLSIDEQEI---KPGRQIELSMSGRPGAY---VGLAAYDKA

AgA2M-5 LKQ---FSNH--------LDIIIDEKEL---KPGQEIELLLKGRPSAY---VGLAAYDKG

DmA2M-4 LEEN--LLNA--------VQVDA-PIRA---PPGQDIDIGISTKPYSY---VGLMLVDQN

DmA2M-3 VEVE--LQNQ--------IEIKA-PAEV---KPGADVALEIKTSPKSF---VGLLAVDQS

DmA2M-2 IERD--FENT--------IEI-LTTNEA---LPRDEVSLKVKTNPHSF---VGLLGVDQS

DmA2M-1 FEKE--FSNS--------IDV-SAPTNA---KPSEEVKLRIKTDADSF---VGLLGVDQS

SmA2M-2 VEGI--FKTK--------VNIHANPNST---KPGSPVDISVQTEPQSY---VALLAVDQS

IsA2M-1 LGGI--LRTP--------VQVQSNLAET---KPGGQVDILVSTRPNAY---VGLLGVDQS

TuA2M-2 VGGI--FRTN--------VEVSASTEKA---KPGEKVDIKVKTNSNAY---VAVLGLDES

SmA2M-1 VEGV--FRNFVIKLFQKIVDLKANKHGS---QPGDTINFQVTTQPNSF---VGLLAVDQS

AgA2M-1 FKRV--FENQ--------IQLTLSKDEL---KPVETLDIDIRTEKDSF---VGLLAVDQS

AgA2M-2 FDSE--LQNF--------MKVSLSKEQS---KPGQDVEISISTNPDSY---VGLLGVDQS

TcA2M-2 VDGL--LQNY--------MEIQVNPVET---EPELDIDIAIRAQPNSY---VAIMAVDQN

DpA2M-1 VDGT--FQNF--------VDIQVTPDSV---EPGKAVDIVVKAKPNSY---VGVLGVDQS

RpA2M-1 LEGT--LQNY--------VRLDSTLEES---EPGGNIQLNIETKPNSY---VGVLGIDQS

NvA2M-1 LEGT--LQNF--------VDIKPVSDEV---GPGDNIDLTITAKPNSY---VGLLGVDQR

AmA2M-2 LEGV--LQNF--------VDIKMAPKEV---APGENVNLIITSKPNSY---IGLLGVDQR

CfA2M-1 LAGV--LQNF--------IDLKVTPGEV---MPGESVNLVISAKPNSF---IGLLGVDQR

PhA2M-1 FDNI--LQNF--------VKIDAKPLTS---EPGKSVELSIQAKPNSY---VGVLGIDQS

ApA2M-1 LDGV--LQNY--------INVDVSRDEV---EPDTSVDINFEAKPNSY---IGVAGIDQS

TcA2M-1 VEPC--LNNK--------VEANWSHKQI---APGATASLLIESKSESL---CSVVTTDKA

NvA2M-2 VGHC--FENK--------VKTAWQQEKQ---SPGSLAKFHVEATPLSL---CGISAVDKS

AmA2M-1 VGHC--FENK--------VKSTWHTDAQ---IPGSPTQYHVEAAPRSL---CAISAVDKS

CfA2M-2 VGHC--FENK--------VKSAWHTDAQ---TPGTVTQYHVEAAPWSL---CGISAVDKS

. : ::

ApA2M-2 SEDLE-------------L---SNSTASIVVKSVPIYVHPEGI-----------------

TuA2M-1 VYLLR-------------S--ANTLTRHGLRKMFNSKDKGCGPGGGIDPTDVLLNSGLVI

SmA2M-3 VYLLN-------------K--EGLLTRKTMFDEMERHDLGCGP-----------------

DpA2M-2 FFNMQ-------------A--GNELSYAEVITKMTSFDESLNG-----------------

AgA2M-4 LLQYS-------------N--NHDIFWEDVWGVFDKFYSVERN-----------------

AgA2M-3 LLLFN-------------K--NHDLFWEDIGQVFDGFHAINEN-----------------

AgA2M-5 LLAYS-------------K--QHDLFWEDVMQVFDTFHATDQN-----------------

DmA2M-4 ANDLR-------------S--GHDLTHKRLMDALRSYELSDVN-----------------

DmA2M-3 VLLLG-------------S--NNDLNKESFNWRLNGYDTS--------------------

DmA2M-2 VLLLR-------------S--GNDLNRDLILNNLATYSTDLVI-----------------

DmA2M-1 VLLLK-------------S--GNDLSQDDIFNSLNIYQTSTP------------------

SmA2M-2 VLLLK-------------S--GNDITQREILDDLQSYEIGRQS-----------------

IsA2M-1 VLLLK-------------K--GNDLSQEQVIEELESFDSGKQA-----------------

TuA2M-2 VLLLK-------------S--GNDITRDDVTNELATYDSNYYN-----------------

SmA2M-1 VLLLK-------------S--GNDVTQEEIIHQLELFDTGKQP-----------------

AgA2M-1 VLLLK-------------S--GNDISRDEVVQQLEMYESAQNY-----------------

AgA2M-2 VLLLK-------------S--GNDITKQQVFSELEKYEERSYG-----------------

TcA2M-2 AVKMR-------------P--GFDLTHSEVAEELKKYDPAQQS-----------------

DpA2M-1 VLLLK-------------T--GNDISRQDVLDEVKSYDSTRRP-----------------

RpA2M-1 VQLLK-------------T--GNDIDEEEVVRELSSYETSDFG-----------------

NvA2M-1 SLLLK-------------S--GNDITYDQVRKELMSYDVNDAA-----------------

AmA2M-2 SLLLK-------------S--GNDISYEQVYKELKSYDNAHES-----------------

CfA2M-1 SLLLK-------------S--GNDISYEQVYKELQSYDRVKAS-----------------

PhA2M-1 VLLLK-------------S--GNDISHDDVLNELRSYDSGNYS-----------------

ApA2M-1 VLLLK-------------T--GNDISHDNVLDELRTYDNGEHS-----------------

TcA2M-1 VTFMD------------------DLRFLNVKSLLKPFLQQKEA-----------------

NvA2M-2 TRFLTQSQPVSSEGGSAASTGSNLLEPEATFARLKPFHLPPETMPMQSTWAHCDKSQGAE

AmA2M-1 TLFLS-------------KSESNLMSSTQTFDALKRFHPTPKF--------YFPWENSRC

CfA2M-2 TRFLG-------------S-KANLIDADQTFEQLKRFYIEPEL-------RPIWTQCKVT

.

ApA2M-2 ----------------------------------QHQTTDNAYFCANEQLIVSTTSDFRY

TuA2M-1 AGIPSNSIGSSCVHMNDKSSKRRPIRASPRIYIT--SSSSSMRKFASSPTYSSHSASPVS

SmA2M-3 -----------------------------------GGGQDTEAVFRKTGFLILSNAAINE

DpA2M-2 ----------TLRHYWTSREGDAETVVN---YPASTFGIDANRTFEYASLVVFTDAFVVR

AgA2M-4 ----------------------------------------EFDLFHSLGLFARTMEHITF

AgA2M-3 ----------------------------------------EFDIFHSLGLFARTLDDILF

AgA2M-5 ----------------------------------------EFDVFNSMGLFARLSGGNR-

DmA2M-4 --------------------------------------TPMGSPGKESGVITMSNTDYFI

DmA2M-3 --------------------------TP---WQG----GYSYYPGERTGVVTMTNAYFFY

DmA2M-2 ------------------------------------LTNANINIYRSSGGCYTNPGYTNC

DmA2M-1 -------------------------------WMN----GYGRYPGQTSGLVTLTNANYPY

SmA2M-2 ------------------YYSYRDGNYMIRPWRP--PSPSTMQLFSNVGLVFFTNGLFAH

IsA2M-1 -------------RVWPPWYRRRRRSL----WWP--GSTTAHDLFKDSGMVVLTNGLVYE

TuA2M-2 -------------PNSDDSFSYFNRNYF---NSL----SAAANIFHNAGLIILSNGIVFD

SmA2M-1 --------------------KSHLDLIYSSIWFP--GSATASEVFKDAGVMVMSNALVYE

AgA2M-1 -------------------------------HWD----AYSTSDCQSVGAVLLSNRFIPR

AgA2M-2 -------------------FYRRKKRFA---WNP----HAEHRDFSTVGAFVMSNANDPP

TcA2M-2 ----------PYSMIMHDSKYH----FF---WKP--GAANPHSAIYNSGADLLTNSHVDR

DpA2M-1 ------------------------DFES---WLPEVGGPKMCEELR--------------

RpA2M-1 -------IFRPFKKVLDDLGQRRSVY-----WSP--GSFTADEVFSGSGAVILTNGYVHK

NvA2M-1 -------------------FYDQEDYEH---SWIRPGSANADEVFRKTGTVVLTNGYVHK

AmA2M-2 -------------PYTNSIFDR----YL---WSP--GSATAKDVFRESGAIIITNGYVHE

CfA2M-1 -------------PYTDSFFGRPL-------WSP--GSGTADDVFRKTGVVILTNGLVHE

PhA2M-1 ----------PYSNFHNNFFRRFKRSFF---YWP--GSATAQKVFDNSGAVILTNGWVYD

ApA2M-1 ----------NYMPYLRESLDRRSMF-----WWP--GSYTAHQAFDKSGATILTNAFVND

TcA2M-1 -------PESGRKSCLPPVKKNRRRRFV----YSFSEDFDAYDIFEKFGIVTITNFKVVT

NvA2M-2 DSNSDEGPMEEIDHLPKPAARNKRHSVT---YNVAANYVDAIQAFDDFGTIVMSDLILES

AmA2M-1 KSAIGPEEMKEEINHLPQFLRSKRQTIT---YSKRVNYVDAVQAFVDFGVIVMSDLVLET

CfA2M-2 TQQETNTEEIDHLPIPLWGRRKKRTVISPGPYSGLTNYVDAIQAFDDFGAVVMSDLILET

ApA2M-2 QH-------------------------------------INAPKNRDGIVF-------EI

TuA2M-1 SS--KQYSTNAFINQCCRLGKIKPNDKAKAMSCEERRDILLRSVKNVNCASAFLDCCLNS

SmA2M-3 HK-----RIDGECDANHKRNKRSLSTLPKPVKVEHFYTGLAAVETEVTYKASYMAVARSA

DpA2M-2 RP-----DACNITLGFLACMSGSCYRSERRCDGQ-----YDCDDRSDEAGC-------PS

AgA2M-4 DK-------------------------------------ANDQTARDGSSS--------S

AgA2M-3 DS-------------------------------------ANEKTGRNALQS---------

AgA2M-5 ---------------------------------------IGASPTTTERFG-------SA

DmA2M-4 EK-----EAESNPALDREVSTGPEEDKLTTVRKTD----IGPAHKIEVNTL-------PP

DmA2M-3 NR-----TAPDYNIQGFGG---------SSFA-------MRKTTVAHDSHVFHSGAGGPT

DmA2M-2 TG-----SL------------------------------IGRTMFKNEPTKNSGPVPIVG

DmA2M-1 NT-------EFPDYVEDDPEI------------------YAFENNLDALPPMPAIANFPP

SmA2M-2 IP-------SYGGYGGYGGYGGGGMYDGELYEMDA----MPRPAVHFAPQAFASGNRGPP

IsA2M-1 SD-----DGLFARKQVIR---------------------LDTDVLTNPVLP---PSDLPE

TuA2M-2 HSIQLHYRSSFPVSLSESADSMSAFDE------------APNPVGSRFYTKSLSSSTAPV

SmA2M-1 EY-----NFIMPRGGGELRPGVE----------------KGKPVGDYYIPKEAEVGFRDP

AgA2M-1 DI--------FPQARLFACSTSAGG--------------FGAAPMMAACKMKGVIMESEM

AgA2M-2 QI-----HPVFFSLPALA---------------------APPGVIITSARPFVAATALSA

TcA2M-2 HQ-----PTLEDIYLRPVFYGT------STVKPD-----RGFGLPLHTVTR-------PP

DpA2M-1 ---------------------------------------LAAPTLSVDVSF---------

RpA2M-1 HT-----PWLY----------------------------FRGGMPQDDLMFSANVSPEMA

NvA2M-1 NP-----QLTYPEEEGDRLKNPAQGISISTLRPD-----IGPPVKHRFATR-------PA

AmA2M-2 NL-----PIKQPGILEGRITGSPHG--ASTLRPD-----LGPPVMHKLATR-------PP

CfA2M-1 SF-----PMLY----------------------------YRANLDEALYAS------AAG

PhA2M-1 FVPLIHFRSKLPSVEVEEFPMMPEISM-KTVDVSSSVINSGPRVRHRFPEAWLWDSIYSG

ApA2M-1 YN-----PWVY----------------------------YRSNVMDDQEMM-----PIPS

TcA2M-1 KP-----C-------------------------------YTGPIPPTEDPVSSLTDQYDT

NvA2M-2 RP-----CPPWRSSFGRVPLGPSSDEIDE----------PDRMLKAVKAMPLAFQFGAPG

AmA2M-1 RP----CPWLFMEYTAL----------------------SRQYISTNEYMSMKDNSEFAV

CfA2M-2 RP-----CPQLHRMDRFRSSTIQTFFLRSTSV-------LESEAGMFKMAAKTLPMAYPL

ApA2M-2 QAKKSAHIL---------------------------------------------------

TuA2M-1 IIPPLLRTS------------------------------VFDVSSGESKGQDNTEAGVTL

SmA2M-3 MSNKANDAN------------------------LEDSEL---------------------

DpA2M-2 NIRRELAQY---------------------------------------------------

AgA2M-4 KNGPSNSQA---------------------------------------------------

AgA2M-3 -GKPIGKLV---------------------------------------------------

AgA2M-5 ASRPISRLV---------------------------------------------------

DmA2M-4 GKGRYAFSY------------------------TPKPFW---------------------

DmA2M-3 QAVGFSAES--------------------------------------------------A

DmA2M-2 STRAQASLP---------------------------------------------------

DmA2M-1 DTGNTVQPV---------------------------------------------------

SmA2M-2 AAPPPRPPP----------------------------------------------PPQSR

IsA2M-1 APPPVPGRI---------------------------------------------------

TuA2M-2 KVRKYFPETWLWANQTSGEQILRYGFSIPGLAPSPAGAVSGKSIVLDDKLLDYRPRLAKI

SmA2M-1 LDGSIIP-----------------------------------------------------

AgA2M-1 ATAPVNEPT---------------------------------------------------

AgA2M-2 SSPVASDPI---------------------------------------------------

TcA2M-2 LAGPYAFSR------------------------IPKPVW---------------------

DpA2M-1 -SKKVEEPI---------------------------------------------------

RpA2M-1 FDGGASSGL---------------------------------------------------

NvA2M-1 WAGRYAFSY------------------------VPLLPW---------------------

AmA2M-2 LAGPYAFSR------------------------IPPPVW---------------------

CfA2M-1 FSGPAALPP---------------------------------------------------

PhA2M-1 AMPATAFGG---------------------GAIIPEAAF-----------DAPLSSVGAP

ApA2M-1 TVSETSSSI---------------------------------------------------

TcA2M-1 QNEDNITPI---------------------------------------------------

NvA2M-2 IPGPEASPM-------------------------------------------GQDVNYVN

AmA2M-1 AAAAMDSGI--------------------------------------------------G

CfA2M-2 LNEGPEQAY---------------------------------------------------

ApA2M-2 ----------LSQERKPTTLMYQIVL--GDL------------------DNTISWIGRGK

TuA2M-1 GEEDAIEESTLIRQDFRETWLFDLVTLDESQ--------TSVKYPVTVPHTITSWR----

SmA2M-3 IDEFEVYVLQNLRHLFPETWIFHTEKITNKECSKNNKNSCKLTYSTNFPHSITTWR----

DpA2M-2 --RLKRIN--RLLRMYENSWLWKDINI-GPH--------GHSIFSVQIPEIPTHWV----

AgA2M-4 ----------SFRTNFLESWLWKTDKI-GSS--------GSATTKESVPDTITAWH----

AgA2M-3 ----------SYRTNFQESWLWKNVSI-GRS--------GSRKLIEVVPDTTTSWY----

AgA2M-5 ----------AYRTNFLESWLWQNVSI-GRT--------GSRTVHEVLPDTTTSWY----

DmA2M-4 --HNPRV---HVMRDPADTWLFLNISA-SSD--------GRNSIHRRIPSEMTSWV----

DmA2M-3 SASAAPV----VRKNFAETWIFADIES-TEE--------EVFKWVKTIPDTITNWV----

DmA2M-2 ----------PVRKLFPETWLFSNITDVGAN--------GEYIIKETVPDTLTSWV----

DmA2M-1 ----------EIRKNFADVWIWQSIGRSVGE--------EEFTLTKKIPDTITSWV----

SmA2M-2 PISDTLMEPTRVRRHFPETFLWTNATA-GAD--------GMISITANAPDTITSFF----

IsA2M-1 ----------RLRQQYPETWLWSNVTA-SHD--------GRVVISSTVPDTITSWV----

TuA2M-2 VLPDATESRIVLRKNFPETWLWDHTYS-RSD--------GLATFSSNIPDTITSWS----

SmA2M-1 ----------IMRQHFPETWIWSNATA-GQD--------GRAVFTREAPDTITSWV----

AgA2M-1 -----------VRSKFPETWIWESISK-CKE---------MESIRKIVPDTITSWI----

AgA2M-2 ----------VVRRTFPESWIWESDE--GFS--------GEKTLQKKVPDTITSWI----

TcA2M-2 --NKPKV---YLTEEIADTWLFTNFSS-GYE--------GKTSIRRKIPSSLNTWV----

DpA2M-1 ----------ALRQHFPETFLWLDITNLGTD--------GTARFVKEAPDTITSWV----

RpA2M-1 -------AAVKVRSDFPETWLWEALDT-GVD--------GKARLNKQVPDSITSWI----

NvA2M-1 ---RPRV---FLMHDISDTWLFSNMSS-GYE--------GKTVIRRTVPDTITSWV----

AmA2M-2 --NKPRV---FLMHDILNTWLFTNFSA-GHE--------GKNELKRNVPDSITSWV----

CfA2M-1 -------EKVKVRKNFPETWLWQTLDA-GYQ--------GKAELRRNVPDSITSWV----

PhA2M-1 ASEGAPV---KIRKNFPETWLWESLDS-GYD--------GRAILKPTVPDTITSWV----

ApA2M-1 ----------KVRKNFPETWLWESTES-GQD--------GRASMKSTVPDTITSWV----

TcA2M-1 ------------RSFFPETWLWEIVPV---R--------SVAVIHRTLPHTITTWM----

NvA2M-2 PTSMESQTSTIVRSYFPETWIWELVPT-GKD--------GRATIERQLPDSITDWI----

AmA2M-1 YVDQNQAQMATLRSYFPETWLWELVPI-GEE--------GKITIERTLPHTITDWV----

CfA2M-2 -VDQVPDQPATLRSYFPETWLWELVPT-GKE--------GKVAIERTLPHTITDWV----

:: :

ApA2M-2 HGNGVHLTSRNTPGILSEEEP----RTFWISWE------KG------VLAFGYGQE--IH

TuA2M-1 -LNAMSLSAKDGLCLMDKPLRLISNKELHIRVDLPYSIVVNEQVEMLVTIFNNGPT--RK

SmA2M-3 -VQALAVSQTNGSLCIAEPIRLTVFKSVFIQMQLPYSAVRMEQILVPVTIFNYGDE--EL

DpA2M-2 -VSAFSVSPKNGFGLVRSSREFAGYRPFYMNVEMPTNCRQGEQVGIRITLFNYATI--EA

AgA2M-4 -LTGFSIDPVYGLGIIKQPLQLTTVQPFYIVPNMPYSIKRGELVELQFIVFNNFPK--KY

AgA2M-3 -LTGFSIDPVYGLGIIKKPIQFTTVQPFYIVENLPYSIKRGEAVVLQFTLFNNLGA--EY

AgA2M-5 -LTGFSIDPVYGLGIIKKPIEFITVKPFYIVDSLPYSIKRGEAAVLQFTLFNNLEA--EY

DmA2M-4 -VSAFALDPVNGLGLSPPNGKLEAYKEFYISTELPYSIKRDELIAIPFVVHNNRDS--DL

DmA2M-3 -VTGFSLHPQKGLGVTNDQTNIKTFQPFFVSVRLPYSVKRGEVINVPALVFNYLPK--TL

DmA2M-2 -ITGFSLSPQSGLAVTRNPSRIRVFQPFFITTNLPYSVKRGEVIAIPVIVFNYLGM--DV

DmA2M-1 -VTGFSLNPTSGIALTKNPSKIRVFQPFFVSTNLPYSVKRGEVIAIPVVIFNYLDK--TL

SmA2M-2 -ITAFAMNENTGLGLSKSPEKLQVFRPFFVALNLPYSIVRGEAVALQALVFNYLKE--DV

IsA2M-1 -ISAFALDSLTGLGIAPSQAKVTVFRPFFVTASLPYSILRGESVAIQCVVFNYNNK--PV

TuA2M-2 -VTAFAVDKRTGLGVVEKPTKVTVFRPFFVKLNLPYSIIRGETVAIQVLVFNYFTK--PR

SmA2M-1 -ISAFSLDMFTGLAVSPSPLRVTIFRPFFISLNLPYAVIRNEAIAIQAVIFNYMKETIEV

AgA2M-1 -ITGFSLSKSHGLGLVDNPSKVNVFMPFFLSIDLPYSVKLGETIRIPVVVFNYMDE--DQ

AgA2M-2 -ITGFSVNPIYGLGLTQQPRKLNVFLPFFVSTNLPYSVKRGEVVAIPIVVFNYMED--DQ

TcA2M-2 -VTGFSLDPIHGLGLTTTSKKVKVSKSFVVTLDLPFSVQRREILAVPVVVYNYMDK--DV

DpA2M-1 -ITAFSLDTFHGLGVIEQPAKMQVFRPFFIQLNLPYSVIRGEVVAIQAVVFNYMNK--EI

RpA2M-1 -ISAFSLDPVYGLGLMDVPKKVKVFRQFFISLDLPYSVIRGETMTIPVVVFNYMDK--SV

NvA2M-1 -LTGFSVDPAFGLGLIEAPRKLRVFKPFFLSMNLPYSVIRGEIVAIPIVVFNYMSK--DL

AmA2M-2 -LTAFSVNDVHGLGLIKEPQKLKVFRPFFIAMDLPYSVIRGEIVAIQIVVFNYMNK--NV

CfA2M-1 -LTAFSVSDAHGLGLIEEPRKLKVFRPFFISMDLPYSVIRGEIVGIQIVVFNYMNK--DL

PhA2M-1 -LTGFSVDPVYGLGLIEAPTKVKVFRPFFVSLDLPYSIIRGEIVSIPVVVFNYFSE--EV

ApA2M-1 -ITAFSVDSLYGLGLLDSPKKLKVFRPFFISVDLPYSVRRGEYVSIPVVVFNYLSK--DV

TcA2M-1 -TNVMCVSATEGVGFSK-TGEITTFRPFFVDILTPYSIKRGETLYLHAIIFNYLTY--NI

NvA2M-2 -GNTVCISSKSGLGIGN-PVQITSFQPFFLDYSLPYSVKRGEQLRLKVSLFNYMQH--SL

AmA2M-1 -GYTTCISPTHGLGIAP-PTTITAFQSFFLDYNLPYSIKRGEIMRFKVSLFNYMHH--SL

CfA2M-2 -GYTTCISSTHGLGIAP-PTTITGFQPFFLDYSLPYSVKRGEMLHMKVSLFNYMQH--SL

: . : ..

ApA2M-2 QYPLLKWNMDK-K-----------------------------------------IKINHI

TuA2M-1 KVNLFMYGVDG-VCSEADAGQKTER----------RL---VTVEPGMLHTEGFALSPIRT

SmA2M-3 PVTVYMYGVEG-ICMGAGAGERSES----------QK---ITIPKNSATTVTFPIMPLEV

DpA2M-2 DVVVTLAD-SP-DYKFVHVEEFGEVKSYEARTSRGEKQHLTWIPAQGSQVVYIPIVPTKL

AgA2M-4 KASVTLFSVDN-QTEFVG--------RPATETSYTKS---IEASPDTGVPVAFLIKARKL

AgA2M-3 IADVTLYNVAN-QTEFVG--------RPNTDLSYTKS---VSVPPKVGVPISFLIKARKL

AgA2M-5 IADVTLYNVAN-QTEFIE--------RPDKDLSYTKS---VSVPPKVGVPISFGVKARKL

DmA2M-4 NVEVTFYNSAL-DFDFPQLDPKATN-QPKVELYRRRS---LQVPGRSARSVSFIVTPKRV

DmA2M-3 DVELTLDNEDQ-EYDFVDASNEVIG-----DQKRTQN---IRVGANEAAGASFLIRPKVI

DmA2M-2 KAKVLMDNSDG-QYEFIETTNKNVS-QYLRGVRRKKT---LWIPANTGRGISFMIRPKKV

DmA2M-1 DADVVMDNSDQ-EYEFTEATNEVLE-KAIDEVRRVKR---VTIPANSGKSVSFMIRPKNV

SmA2M-2 DAEITLENGQN-ALDFVELENTVDE--DKSQKKLIKT---IRAKAGEGTSVSFLVAAKKL

IsA2M-1 QARVTLENAKS-EFVFTSLSNDVGG--EQSKDRRSKE---VTVPAQDGVAVSFLITPTKL

TuA2M-2 EAQVVLHNDNK-EFNFTVASNEVDT--YLNDATRSQF---VQVAAESAASVTFLISTNRV

SmA2M-1 CATVTLEN-TN-QFDFVTVEDVVNE----VEVVNSKT---KKVESGTPATVYFMIVPKEL

AgA2M-1 LADVIFYNNDD-EFEFVSDTKD------QKEKHRQEQ---ITVPRGTGKTLTFVLKPTKV

AgA2M-2 TAEVVLHNDEQ-EFEFADVENEVVE-SNKVELFRQKR---LDIASNTGKSVSFMVKPKKL

TcA2M-2 NAEVTLHNPEQ-KFEFAEVSNNVNS-TRKVELYRRKK---INIKRNSGTSVSFMIRPLKQ

DpA2M-1 TAELTFEN-IG-DFQFIDNGLEDNE-ISSEAIFRKKS---VRIPAQDGTPVSFLIRPTTL

RpA2M-1 YADVTLEN-TG-QFEFADYSNDVNE-APKLELYRRKK---LTIQPNSGSSTSFMITPKEL

NvA2M-1 NVEVVLEN-NG-DFEFAEVSNEVHDNTKRLELYRTKK---IFVKANSAESVAFMIVPTKL

AmA2M-2 VAEVLLTN-EG-QFDFAEISNEIQD-VPKLELYRKKK---VEVKANSGSSISFMIIPREL

CfA2M-1 TAEVLLTN-EG-QFDFAEVSNEVHD-VPKLELYRRKK---VDVKANSGSSVSFMIIPREL

PhA2M-1 TAEVTLKN-EG-EFEFSDAANEVED---TPSTHRTKT---VKIKPNSGEALSFMITPKTL

ApA2M-1 TADVTLEN-IG-QFDFADTSNDVRD--SKLELYKRKS---LTIKSNSGSPTSFLIQTKDL

TcA2M-1 PIRITLGTSEG--LKLVDTKNRKSF-----------S---YCISSNNTATHIFELKGTDV

NvA2M-2 PVLIKLLDHEGLDLGFNSSSEAS-----------------YCLGPRDSIVHEFPLLPREL

AmA2M-1 PVKIKLEEMEKIDLHLSEPTAS------------------FCVKPRDNIVHEYILKPRVI

CfA2M-2 PVKIKLEDATG------------------LDLHLSHAVASFCVKPRDSVVHEYILRPRVL

: :

ApA2M-2 GFATILGTTGQF--------------------------------------RVWNY-----

TuA2M-1 GEFKIQVDALAH------------GTS--DVVIKTLHVVPQGITIIDSYAVQLDPRNLQH

SmA2M-3 ASYPLRVVALSW------------SES--DAVEKILRVVPEGIPVQKPLSIMLDPSGTIR

DpA2M-2 GEIEVTIQAKSL------------IRK--DQVVRRIRVEADGVPQFRHTSVMLDL-----

AgA2M-4 GEMTVRVDASIE------------PAK--DSIESVIRVIPESLVKREMISRFFCH-----

AgA2M-3 GEMAVRVKASIM------------LGHETDALEKVIRVMPESLVQPRMDTRFFCF-----

AgA2M-5 GEMVVRIKASIM------------TGKETDAMEKVIRVIPENIMFEKTETRFFSM-----

DmA2M-4 GPLLVKAMAASS------------QAG--DTVEQNLLVEHPGAMERINRGFLFEL-----

DmA2M-3 GNILLKFKAISP------------LAG--DAIHKPLKVVPEGITQYQNRAFFINL-----

DmA2M-2 GLTTLKITAISK------------YAG--DRLHQILKVEADGVQKYVNKAVLINVQRLNR

DmA2M-1 GFTTLKITATSA------------LAG--DAIHQKLKVEPEGVTLFENRAVFINL-----

SmA2M-2 EYIDINVVAKSK------------VAA--DAIVRKLLVKAEGKKMYSNKAFLVDL-----

IsA2M-1 GYIDIHVSATSS------------LAG--DSILKKLLVKPEGSKQHFNRAVLVDR-----

TuA2M-2 GSINLKVTALTD------------NAG--DTVIKQLLVKPEGKTQYLNRAVLLDF-----

SmA2M-1 GYIDVKVTARSST--------RSQVVS--DSLKRKLLVKPEGVPQFVNKAYLIDL-----

AgA2M-1 GHVTLKITAKCA------------LAG--DGIERQLLVEPEGLPQYINKALLVDL-----

AgA2M-2 GHITIKVTAKTK------------IAG--DAVERQLLVEPEGLPQFINKAAFIDL-----

TcA2M-2 DTIEIKVTANSP------------KNQ--DVAIKHLQVTTEGETEYYTKTVLIDL-----

DpA2M-1 GNIDLRLTAKAA------------TAG--DAIVKKLLVKAEGETIYRNKAYLLDL-----

RpA2M-1 GFIDIKVVAKST------------LAG--DIVERKLLVKAEGKTVYKNAALFIDL-----

NvA2M-1 NHITIKAKATSV------------MAG--DSVEYPLLVKAEGETQYRNKVVFVDL-----

AmA2M-2 GYITIKATANSI------------LAG--NSVNRKLLVKAEGETQYVNRATFLDL-----

CfA2M-1 GYITIKATANSV------------LAG--DSVEHKLLVNAEGETQYKNEAVFLDL-----

PhA2M-1 GYISISVTATSK------------LAG--DGVDRKLLVKPEGETQYRNKAIFVDL-----

ApA2M-1 GYISIKLTATSK------------LAG--DAIEKKLLVKPEGETIYKNKAIFVDL-----

TcA2M-1 GNVNITVVAELDPNFPGHCG-PEIIINKRDVVFKTLIVEPEGHPITVTKSALLCA-----

NvA2M-2 GEINVTVAAEVDKERAEACGAPTPLPDNRDEIIKPVLVKTEGFPVEISRSSFLCP-----

AmA2M-1 GEVNITVTAFVDIDYPEPCG-SETVIFTQDVIVKPILILPEGFPVEETKSALICP-----

CfA2M-2 GDVNITVSASVDSEYGEPCG-PEVLLYTRDVIVKSILVLPEGFPVEATKSAFICP-----

: :

ApA2M-2 N--------------D--EAGFSQVLHLE------TP----NTMISGSESGTLVVTGGLN

TuA2M-1 RQKRSIKRENLFDSIDPDKGEQKTRIDL-------IPRTQTSAIVPDSEECVVSAIADSL

SmA2M-3 QRKKRNAIDGIEETFDEEHNRQSLVIDMP------LP----KEYIPGTEKCLVSTMGDFL

DpA2M-2 S--------------N--RAWFLQYVYVNVTETPIIPYEKDRYYVFGSNRARVSVVGDVV

AgA2M-4 N--------------T--YQNQSFVLGLD------FD----RKADAGTRKIDFILTPNIL

AgA2M-3 D--------------D--HKNQTFPINLD------IN----KKADSGSTKIEFRLNPNLL

AgA2M-5 D--------------E--YGKQEFNMQLD------IP----KNI--STVQIKCRISSNLL

DmA2M-4 N--------------S--NAQNRRNVTIA------VP----RNAIPESTRIEVSAVGDLI

DmA2M-3 K--------------D--TGEFKNTFELE------VP----EDVVPDSERVEFGLVGDLL

DmA2M-2 R--------------S--LAPPEKTIIIE------KA----DNVIEGSETVEFEVCGTSQ

DmA2M-1 K--------------D--QPEMSQSLDAD------IP----NEVVPQSEFIEFSVVGDLL

SmA2M-2 R--------------N--QSSFEAKVSID------IP----AGAVSESEKIEVSAISDVM

IsA2M-1 R--------------NPSAPPTSTNISIP------IP----KNAVPGSERISVSAVGDLL

TuA2M-2 S--------------KAGGSSSNHLVPLW------IP----NNAVPGSKGLSVSVIGDVL

SmA2M-1 R--------------S--SSLFNASVNVS------IP----KTAVSGSERVEISTIADIM

AgA2M-1 R--------------L--VKEIKQPFEVE------IP----VDAVPDSTNVEVSVIGDVL

AgA2M-2 R--------------A--APELTKTFEVE------IP----KNAVPDSTRIEVAVIGDVM

TcA2M-2 R--------------N--NPNYKKSINFT------IP----QNMVTGSEKIEVSAVGDLL

DpA2M-1 R--------------S--IRNYNKNVSVT------IP----FNAVPGSAAVELSAIVDIM

RpA2M-1 R--------------N--SNHFKTNFTLD------IP----KYIVAGSEQIEIATVGDIL

NvA2M-1 R--------------D--TDSMKTNVTVD------IP----KHFVSDSEYVEVSAVGDIL

AmA2M-2 R--------------N--TKSTSINVTID------IP----KNAVPGSEHIEISAVGDIL

CfA2M-1 R--------------N--VENTGANITIN------IP----NNAVLGSESIQISAVGDIL

PhA2M-1 R--------------S--GGEFKTNVTLD------FP----KNIVPDSEFVEISAVGDIL

ApA2M-1 R--------------K--ESLFEKNITLE------IP----SNIVPDSEFIEIGAVGDIL

TcA2M-1 T--------------D------NITWELP------VP----NDVVAKTANSKLILNGDIL

NvA2M-2 R--------------D-FSDDTSLVWELE------LPRPEDEQVVEGSVSAYVSLIGDVL

AmA2M-1 K--------------D-SSDDSSFMWELT------LP----KDAVPDSGRAYLNLIGDIL

CfA2M-2 K--------------D-FSDDSTITWHLD------LP----DDLVPESARGYVSMIGDIL

:

ApA2M-2 FPFFVQNEPKFSTSLVSF--------LSTFTPLLMSEHNNNGTEEKSLVDLLSKSIPVL-

TuA2M-1 GPSVITTL-SNINHLIIKPTGCGEQNVIRMAPTLFTLDYLNAT-GRLTVTQRETGLKYLK

SmA2M-3 GQAVVTSIQGLEARFFHLPTGCGEQTMIKLAPLVYSVLYLKRT-GRLTAEGEKNGYSLMQ

DpA2M-2 GPA-FPNMPVNATSLLTLPMDCAEQNMFSFAANLYTVKYMRLT-TQRKREIDRQAFYHLN

AgA2M-4 TSV-MDNL----ESLLSVPTGCGEQNMMRLVPIVLVLDYLTSI-GSADKQLTAKAIGLLR

AgA2M-3 TTV-IKNL----DHLLGVPTGCGEQNMVKFVPNILVLDYLHAI-GSKEQHLIDKATNLLR

AgA2M-5 SPV-IHNL----DSLLDVPSASGAPSMINFIPPLVVLDYLKAV-SSTTTHLIEKATGLLR

DmA2M-4 GSL-VGNL----DSLILLPTGCGEQTMVNFVPNLIVLRYLGRL-RQLTPEVELRATNNLA

DmA2M-3 GPV-VKNL----ENLLRLPSGCGEQTMSKLVPNYLVRDYLKSI-KKLTPALDTRIKRNLQ

DmA2M-2 APQ-LEHL----DDLVHLPCGCGEQNMFNFVPSILALSYLKAK-NRQDQEIENKAKRYVE

DmA2M-1 GPT-LQNL----DNLVRMPYGCGEQNMVNFVPNILVLKYLEVT-GRKLPSVESKARKFLE

SmA2M-2 GST-INNI----DQLLRMPYGCGEQNMLNFVPNIVITEYLTKT-KQLNDEIKNKALKFME

IsA2M-1 GPH-VNNL----DQLLVMPHGCGEQNMLDFVPNVVVLDYLRRA-NRLSPAVRGKALRNLE

TuA2M-2 GTS-LNNL----DDLLKIPYGCGEQNMLNLVPNIVVLQYLKTA-GRLTLDIQSKALHHME

SmA2M-1 GPT-VDNF----DNLLQLPFGCGEQNMIRFVPNIVVIDYLSSI-QHLTPIVKSVALTNME

AgA2M-1 GSS-IENL----DSLIRMPFGCGEQNMLNFVPCIVVLDYLKAC-KRLTVEIESKAKRCME

AgA2M-2 GST-IQNL----DSLIRMPYGCGEQNMLNFVPNIVVLDYLKAT-NKLTANIEAKAKKFME

TcA2M-2 GPT-MVHL----ENLIRLPTGCGEQNLIHLMPNLIILQYLRYT-RQVTPTIQNEALDLLE

DpA2M-1 GPS-INNL----NTLLRMPFGCGEQNMLLFVPNIVVTEYLKNI-GQLTDAISSKALGFME

RpA2M-1 GPS-ISNL----AHLIKMPFGCGEQNMLNFVPNIVILDYLKNS-YKLTKAVEERCLNYLE

NvA2M-1 GPS-IPNL----SKLIKMPFGCGEQNMLNFVPNIVILDYLKNT-NQLSPAIESKSIRYLE

AmA2M-2 GPS-ILNL----ANLIKMPSGCGEQNMLNFVPNIMILNYLKNT-NQLTQAVQNKALRYME

CfA2M-1 GPS-IPNL----ANLIKMPFGCGEQNMLNFVPNIVILNYLKNT-NQLTQAVQSKALKYLD

PhA2M-1 GPS-IPNL----QNLIRMPFGCGEQNMLNFVPNIVVMNYLQNT-RQLTPAIQSKAMKFLE

ApA2M-1 GPS-TMNL----ASLIQMPFGCGEQNMLNFVPNIVILDYLKNT-KQLTTAVETKSLKYME

TcA2M-1 GQT-IQNL----DDLIAMPTGCGEQIMANLAPNIYILKYLNET-KQLTSSVRHKIARNLK

NvA2M-2 GPA-LENL----EQLVRLPMGCGEQNMILFVPNIHAIAYLDAINRQTGSEMRARAIKNMQ

AmA2M-1 GPA-LENL----DKLIKLPKGCGEQNMILFVPNNHVIKYLDAM-RINKPDLRAKAIRNME

CfA2M-2 GPA-LENL----DNLVRLPMGCGEQNMILFVPNIHVIGYLDTT-GVENPELRAKAVRNME

:. : : . :

ApA2M-2 ------LSYQNSDGSFGDHP-------------NVPCYWCDIRVLEILWRS-----QSHV

TuA2M-1 AGYENQMMFRKLDGSFSTFE---KRP---------SSLWLTAFVTRILCKA-----APFL

SmA2M-3 QGYSFMLNYRKADGSFAVYQ---HVA---------SSTWLTALVAKVLCQA-----SLFI

DpA2M-2 IGYQRQLSFQHRDGAFSYF----RADWDY----SSKSVWLTAFCARILAEANFNEWENYL

AgA2M-4 AGYQNQMRYRQPDGSFGLWE---KSG---------GAVFLTAFVGKTLATA-----AKYI

AgA2M-3 QGYQNQMRYRQTDGSFGLWE---TTN---------GSVFLTAFVGTSMQTA-----VKYI

AgA2M-5 NGYQLELKYRQRDGSFGNWR-----D-------SKGSVFVTALVGTSLEAA-----SKHI

DmA2M-4 IGYQRILYYRHENGAFSAFG---LDI-------KRSSTWLTAYVARSLRQA-----APFT

DmA2M-3 DGYQHMLHYRHDDGSFSSFG---PTKWRQEDPVRNGSTWLTAYVLRSFSKI-----KDII

DmA2M-2 TGYQIELNYKRNDGSFSAWG---QHD-------ALGSTWLTAYVIRSFHQA-----AKYI

DmA2M-1 IGYQRELTYKHDDGSYSAFG---KSD-------ASGSTWLTAYVMRSFHQA-----GTYT

SmA2M-2 SGYQRELTYKRTDNSFSAFG---NSD-------KNGSVWLTAFVVKSFVQA-----KNYI

IsA2M-1 DGYQRQLTYKRDDNSFSAFG---NTD-------RSGSTWLTAFVLKSFVQA-----VPYT

TuA2M-2 LGYQRELTYKRSDGSFSAFG---ESD-------KNGSTWLTAFVLKSFYQA-----KEYI

SmA2M-1 TGYQRQLTYKREDGSFSAFG---NSD-------QSGSTWLTAFVMRAFSQA-----KYFI

AgA2M-1 IGYQRELTYKHQDGSFSAFG---ESD-------KSGSTWLTAFVAKSFQQA-----AKHM

AgA2M-2 AGYQRELGYKHRDGSFSAFG---END-------KSGSTWLTAFVARSFKQA-----ANHI

TcA2M-2 KGYQQQLSYKRKDGSFSAFG---MRD-------EKSSVWVTAYVALTLRQA-----KGHI

DpA2M-1 TGYQKELTYKRDDGSFSAFG---KSD-------AAGSTWLTAFVARSFRQA-----QPYI

RpA2M-1 KGYQQELTYKHDNGSFSAFG---NSD-------TSGSTWLTAFVAKSFYYA-----GRHS

NvA2M-1 TGYQQELTYRHTDGSFSAFG---KTD-------PSGSTWLTAFVAKSFKQA-----EKYI

AmA2M-2 IGYQRELTYRHNDGSFSAFG---MSD-------SSGSTWLTAFVAKAFKQA-----AAYI

CfA2M-1 TGYQQELTYRHTDGSFSAFG---MSD-------PSGSTWLTAFVVKSFKQA-----AEYI

PhA2M-1 TGYQQELTYRRDDGSFSAFG---KSD-------PSGSTWLTAFVAKSFQQA-----GAYI

ApA2M-1 TGYQQELTYRRSDGSFSAFG---SAD-------ASGSTWLTAYVVKSFRQA-----MPYI

TcA2M-1 IGYQRILNYIHKDGSFSAFG---YHD-------SSGSMFLTAFVVRTLQEM-----KKLV

NvA2M-2 KGYQRELNYRHPDGSYSAFGAAADEA-------GSGSMWLTAFVVKSFAQA-----RSII

AmA2M-1 KGYQRELKYRFMDGSYSAFE---EGE---------SSIWLTAFVLKSFAQA-----ASLI

CfA2M-2 KGYQRELIYRHPDGSYSAFGPNVTED--------GSSIWLTAFVIKSFAQA-----KNII

: : :.::. . : :

ApA2M-2 --GV-DSDLIKGLKTWIQKQV-------FEDFSTVVGQSDM-------EINK--------

TuA2M-1 GDSL-DPEVILTAVDYLVDHQESSPSGSWKEYHPVI-HKSALGG----LTGV--------

SmA2M-3 --DI-PKEVTCNALDWTLRKQ--RDTGAFFADFGVY-HTEMIGG----VQSE--------

DpA2M-2 --YI-DPGVIAKAVDWMIQFQ--SPEGAFYEVAPRFADRKMNST----TSWGFNDPIRYR

AgA2M-4 -SEI-EPSMVEQAFDWLAARQ--HSTGRFDEVGPVF-HRDMQGG----LRQG--------

AgA2M-3 -SDI-DAAMVEKALDWLASKQ--HFSGRFDKAGAEY-HKEMQGG----LRNG--------

AgA2M-5 -TEV-DLTLVDRLFEWLAAKQ--HSSGRFDEEQPIT-YYSLQGG----SRNG--------

DmA2M-4 --QV-DSNVLQKALTYLGSVQ--SANGGFEERGDVF-ERF--------GDDG--------

DmA2M-3 --DL-DEQILAKGYEFLLTRQ--AENGSFTEHGEYF-YSS--------QRSL--------

DmA2M-2 --DI-DKNVLVAGLDFLVSRQ--STDGKFKELGMVI-HNS--------HGSP--------

DmA2M-1 --DI-DPKVITAGLDFLVSKQ--KESGEFPEVGKLF-DNA--------NQNP--------

SmA2M-2 --TI-DDTVVSSSLTWLAQQQ--AKNGSFSEVGEVF-HKAMQGG----SGKG--------

IsA2M-1 --SV-DPAVLENATRWLVERQ--KPDGSFEEPGEVI-YKPMQSG----AGSG--------

TuA2M-2 --TI-DDKVLAEATDFLLKQQ--KSDGSFPEHGEIH-HKPMQGGA---AGSQ--------

SmA2M-1 --AI-DEQVVNGSLYWLIAQQ--LENGSFPEVGVVS-NKAIQGG----SGKG--------

AgA2M-1 --TI-EEDVIDSALGWLSKVQ--TADGAFPEVGTIC-HKDMQGG----AGSG--------

AgA2M-2 --TI-DEGVIDKSLEWLSDHQ--APNGSFPEVGVVS-HKDMQGG----SGSG--------

TcA2M-2 --YV-DEKIIEGCLEWLANIQ--GRNGSFVEVGSVI-YKEIQSR----EGNS--------

DpA2M-1 --TI-EDHVIEDSLKWLSANQ--APNGSFPEVGKVS-HTDMQGG----SGKG--------

RpA2M-1 --DIVDPQVISDALSWLASKQ--ANNGSWSEVGQVS-HKEMQGG----AAEG--------

NvA2M-1 --TV-EEKIIADALKWLAEKQ--APNGSFPEVGTVS-HRDMQGG----AAKG--------

AmA2M-2 --PI-EDRIIDEALQWLSNNQ--APNGSFPEVGKVS-HRDMQGG----AAKG--------

CfA2M-1 --AV-EDRIINEALEWLSNNQ--ASNGSFPEVGKVS-HRDMQGG----AAKG--------

PhA2M-1 --TV-EERIIEEALDWLQKNQ--GSNGSFPEVGHVS-HQDMQGG----AAKG--------

ApA2M-1 --PI-EEKIIIEGLQWLSNNQ--ANNGSFPEVGYVS-HSDIQGG----SSKG--------

TcA2M-1 --YV-DQKIIERAVLWILSHQ--LENGCFSTMSHVF--QDMGGTNS--ENST--------

NvA2M-2 --QI-DERDLKLSVKWIVRRQ--LENGCFPVVGQVF-HKDMKGGLREEDGSS--------

AmA2M-1 --HI-DKYVLESSVSWITMNQ--LEDGCFPVIGTVF-HKSMKGGLQ-EHGSS--------

CfA2M-2 --HI-DERDLKISVKWMLKKQ--LENGCFPMIGRVF-HKDMKGGLQDDDSSS--------

: : :

ApA2M-2 -IICAADTLATLMELGI-ES-----EIDSKIANHTKSYLEQHLDN---VVKPYPLAITSY

TuA2M-1 -IPITAFTYTTLRSCENFTYPRILAKRRDKSLKLAESYLCGKLTSELAKGDPYHLALLAY

SmA2M-3 -ATLTAYVLISIMECSKCDS-----ADKRIAALRAISYLEHHVGF---LNHPYSLAVVTY

DpA2M-2 NISLTAHVVIALTTIRDLPGELG--PRVAVSRSRAISWLDRNLNLLEKFGDPYEVAIVAY

AgA2M-4 -IALTSFVLIALLEQPK-VA-----TKHRAAIEKGIDYVTQTLGS---IEDSYDLAIATY

AgA2M-3 -VALTSYVLMALLENDI-AK-----AKHAEVIQKGMTYLSNQFGS---INNAYDLSIATY

AgA2M-5 -IALTSFVLIAFLQNTK-AS-----AQHRSIIEKGIQYVANQLES---IADVYDLSLATY

DmA2M-4 -ISLTAFVTLALMENVD-LY-----PEYRNNINKALDFITRGLDG---SSNLHAMAIGTY

DmA2M-3 -LTLTANSLLALLEEEK---------PNQAAIDKAVAYLSANTAE---SIELLPKSIAIY

DmA2M-2 -LALTSFVLLTFFENEE-YM-----PKYKHVIDRAVEFVVTEVHQ---SNEPYDLAIAAL

DmA2M-1 -LALTSFVLLAFFENHE-LI-----PKYQSAIKKAVRYVAEEADK---TDDQYSLAIAAV

SmA2M-2 -LSLTAYVLSAFLESKG-IELLPGTSSVEEVVKSSLEYLEKELHN---LKSDYDLVITTY

IsA2M-1 -AALTAYVLIALLENKVGFQ-----HALRFAASAAEEFLLKELRT---QSDPYVVAVVTY

TuA2M-2 -GALSAYVTIALLQNNY-FK-----QQYPKHFDRSEKYLYEQLRE---AKTSYETNIIAY

SmA2M-1 -LALTAYVLLAFVEN-K-AE-----RIFSSQMTKALRLLEDQIES---IEDSYSLAIVSY

AgA2M-1 -MALTAYTVIAFLENPK-LG-----EKYKASVDKALTYVKEHISE---LDDVYAHALAAY

AgA2M-2 -VALTAYTLIAFLENIN-LV-----DKYKNTINKAIDYVYRNTES---LDDTYALALAAY

TcA2M-2 -LALTAFTLLAFIENQK-YA-----STYSNTINKGLDYIARYISE---QESIHTIALCSY

DpA2M-1 -VPLTAYVLLAFLENK--AG-----LRYGPSMQKAAEFLVKELPS---ITDPYALSLVTY

RpA2M-1 -LALTAYTLTAFLETRT-LI-----GRYNNVINKAVDYLDRNVRL---INDTYPLAVTAY

NvA2M-1 -LALTAYVLSAFLEVEN-IE-----GRYRNVIYKGVDYVVRNMQG---IDDNYALSICTY

AmA2M-2 -LTLTAFTLIAFLENAN-TN-----GRYRNTINKGIDYIVRNIND---LDDAYALSICTY

CfA2M-1 -LALTAYTLIAFLENED-SV-----GKHRNTINKAVDYIVRNMEE---LNDTYALSLCAY

PhA2M-1 -LALTAFTLITFLETQK-VN-----PKFKNTIDKAVDYIVKNLDG---LEDPYAIAISSY

ApA2M-1 -LALTAYTLIAFLENQK-AT-----PVYRNTINRAVEYLVRNLPG---VEDPYAIAICSY

TcA2M-1 -AALTAYVIISLLDGNI--------DVPEAVKTNAKYCIRGYYDL-----DRYTLAISSY

NvA2M-2 -SALTAYVLIALLESGV--------PLSAALVNNALYCLEKASVSDHFADNPYTGALTTY

AmA2M-1 -SALTAYILISLLESGV--------PLSPSVVNDAQKCLEKGMNN----DDLYTTVLTTY

CfA2M-2 -SALTAYILISLLESGV--------PLTATLINNALHCLEKGMEN--GGGTTYTAAISTY

:: :: : :

ApA2M-2 ALM----VSNSYFTKKALTKLQSLST--NQESEFGWPKVHPTSDW-----YDDVVPQKKG

TuA2M-1 SLSTTTCLQSAERRKSIISRLREIGVYSSGE-NKLF--------W---------------

SmA2M-3 ALT----LAKSPKASSANAKLKALAIFNEAG-DTRF--------W------DVNHAEFQG

DpA2M-2 AMM----LAKSTSAEAAFGLLQQKAR--EDG-GYKY--------WGREPVPLPAQRLENQ

AgA2M-4 ALL----LQKHSSGERFLEKLIGLSTVQQNG-TERF--------W----------ARDAH

AgA2M-3 AMM----LNGHTMKEEALNKLIDMSFIDADK-NERF--------W---------------

AgA2M-5 ALM----LADHRQKSSALNKLIELGI--ATN-ETRY--------W---------------

DmA2M-4 VLS----RANHNAKAAFLQRLDSMAT--NKD-GLKW--------W-----NKTAPAGEQQ

DmA2M-3 ALQ----KAKAPEAAKQVASLKSLAK--HED-DRTW--------WTED--LDKLRASKNC

DmA2M-2 ALS----LARNRNAYKVLDKLDKLAT--RRG-DHKW--------W-----------TGSD

DmA2M-1 ALQ----LAKHPQSEKVIAKLESVAR--KEN-DRMW--------W-----SKATESTGED

SmA2M-2 VLH----LADSKKKDEAFEKMNKVAK--TEK-DVKF--------W-----SVPLPVENSS

IsA2M-1 ALH----LSGHRARDGAFQKLLSLAT--RED-DMVF--------W-KDPGVAPVNTTDKQ

TuA2M-2 ALH----LMKSATIEPALAKSLIKMK--QDG-DVSY--------WSDEDETEKTNFTNKQ

SmA2M-1 TLH----VINSGKKDAAFRQLQSKSI--SAG-EFRY--------W-----KKNASAETAE

AgA2M-1 ALQ----IADHPLKNEVYASLLSKSN--KQG-DIQW--------W-----SKEIPEKNDS

AgA2M-2 ALQ----LADHSSKGLILSKLDTKAT--TDS-DSKW--------W-----HKPIPETEQK

TcA2M-2 TLQ----LARHPSKQSAFNLLDLRSK--SRG-NLKW--------W-----SKDVPSNEIK

DpA2M-1 ALH----LAEVEERDAAFDMLQAKAN--TTDEEFRF--------W-----SKPKSEKDKS

RpA2M-1 ALT----LARHPTSSIAFDKLESLAN--TSS-DMKW--------W-----TRSLSVSERK

NvA2M-1 VLS----LARNAYEDEAFRLLDSKAT--TKD-EQKW--------W-----SKPIPEDDKK

AmA2M-2 ALN----LAKHPYENTAFNLLESKAM--TKE-DIKW--------W-----NKPIPVNDKN

CfA2M-1 ALN----LAKHPYETSAFNFLESMAM--KKQ-GIKW--------W-----SKPIPKDDKN

PhA2M-1 ALH----LAEHPSKDQAFHLLEEKAK--TED-DMKF--------W-----KKPIPAGDEK

ApA2M-1 ALH----LADHPEKNVAFNLLELKAN--TVD-GKKW--------W-----KRMDRANDKK

TcA2M-1 ALLK---INWFSEAERMLKKLFQVSS--HKD-NMMW--------W---------TNREIN

NvA2M-2 ALA----LLEHPRANESLRSLMGRAS--RQK-DLLW--------W------------EDK

AmA2M-1 VLA----LLEHPKANSSMKSLMNRAT--RYK-NLIW--------W------------EDK

CfA2M-2 ALS----LLEHPKANNSMKLLMERAT--RNN-DLLW--------W------------EDK

: : *

ApA2M-2 E--------NISID--EFKASLYCLMIYSA--RRELKSSE--PIVRYLYYRTKILDTYPE

TuA2M-1 ---------NTTTP---IETTGYVLLALLN--LRETKPDEIKAIINYLESQRSYTGAFDA

SmA2M-3 NKPWIYVNRPNALA---VETTSYALLTQLL--F-DDIAYS-HPIINWLNQQRNDQGAFAS

DpA2M-2 R-PFLLPRLPNAFDAANVETTAYALLTYVG--RQELFVE---PIVKWINTQRLTDGGWAS

AgA2M-4 G----------------IETTAYGLLSFVL--A-EKYVDG-TSIMRWLVKQRYTPGSFPR

AgA2M-3 ---------NTTNP---IETTAYALLSFVM--A-EKYTDG-IPVMNWLVNQRYVTGSFPS

AgA2M-5 --P------RDTAS---IETTAYALLSLVH--A-KRYADG-LMVMHWLVNQQSATGSFPR

DmA2M-4 S-PWYNA--TRSVN---IEISAYAALALLE--N-NLVGDA-LPVLNWLMDQRNPKGGFVA

DmA2M-3 GRWWCWI---WSQD---VEITSYALLSLLD--SDQETADSVLNTVRWLIAQRNGFGGFAS

DmA2M-2 ------K--CKSSE---VETTSYVLLALLE--H-NISDEP-KPIVDWLISKRNSNGGFVS

DmA2M-1 GRVFHWK--PRSND---VEITSYVLLALLE--K-DPAEKA-LPIIKWLISQRNSNGGFSS

SmA2M-2 V-PYYNR--PASVD---VEMTAYAMLTYVQ--R-GLIPEA-IPIMRWLISKRNSNGGFES

IsA2M-1 S-DFFFK--AHFKD---VEMTAYALLTLME--R-GDVSAA-IPVMRWLVSKQNSNGGYSS

TuA2M-2 S-SHFFL--PKSTD---IEATAYGLLSLVN--H-NQTDQA-VTVMKWLISRQNAQGGFSS

SmA2M-1 T---IKL--ATPID---IEMTAYALMSYVL--R-NDLSGS-ILIMKWLITQRNVNGGFQS

AgA2M-1 NCCWWYR--PCSVN---VEMSAYGLLATLE--ASSAGLEG-LPIMKWLVSQRNDKGGFES

AgA2M-2 N-PWYSR--PNSVN---VEMSAYGMLAFLE--A-GLDTDA-LPIMKWLIGQRNDKGGFQS

TcA2M-2 N-PWNKL--PRSID---IETSAYGLLTFLE--A-NYFEDA-IPVLNWLLDQQNSLGGFTS

DpA2M-1 N-PWSSL--TTSVD---VEMTAYALLTFLQ--R-GLVIEA-LPIMKWMVANRNSNGGFSS

RpA2M-1 N-PHMFS--PNSID---VEMTAYAMLGYLE--R-SLVNEC-LPIVRWLISQQNEDGGFAS

NvA2M-1 N-PWFSL--SRTVD---VEMTSYALLAYLR--R-NQLSDA-AAIMKWLVKQRNAEGGFAS

AmA2M-2 --PWYYSL-PRSID---VEMTSYSLLTYLE--R-NLIADS-IPVMKWLVKQRNAEGGFAS

CfA2M-1 --PHYSL--PRSVD---VEMTSYALLSYLR--R-NLVADA-IPVMKWLVKQRNTEGGFAS

PhA2M-1 N-PWHERL-PNGVS---VEMTAYAMLTYLE--R-NLIEDA-FPIMKWLVSQRNDEGGFAS

ApA2M-1 N-PWVHE--PNSVD---VEMTAYALLTYLQ--R-ELVEDG-LPILHWLVSQQNDQGGFAS

TcA2M-1 G--------SEASD---IEVTSYVLLALIQQKNEENLAKA-HSIVQWLSTKLGHRGSFKT

NvA2M-2 SRPG-----SLALS---IEMTSYGLLSLIKLGGENNTLEA-LRVVRWLSKKRNAEGGFSS

AmA2M-1 SKP------SIGLS---IEMTAYVILTLLKLGE-ENLSEA-LKAVRWISKQRNSEGGFTS

CfA2M-2 P--------SLGLS---IEMTAYAVLSLVKLGGEANMVEA-LKAVRWMSKQRNAEGGFTS

.: : * : : :: . . :

ApA2M-2 L--AYLAVKAFAMYDKIGSDPH-----RKLTISLATS---GMELTDTLELDPSTKS--QY

TuA2M-1 TQDTIVALEALSTYAKSAYNLT----DINLICNISSGR--FRKS---IEFHEDNAQVMRT

SmA2M-3 TQDTIMALQAMAEYSYRAKLPA-----LNMVCNVSSQ---TSRRQRSLIMTNENSLVLQK

DpA2M-2 TQDTIIATQALIEYTVRHRIRE----VTSLTLHVEATS--NPQLQRTMYITENNLATPQF

AgA2M-4 TQDTFVGLKALTKLAEKISPSR-----NDYSVQLRHA---GRKKE--FRVTSQDIGTLQN

AgA2M-3 TQDTFVGLKALTKMAEKISPSR-----NDYTVQLKYKK--SAKY---FKINSEQIDVENF

AgA2M-5 TQDTFVGIRALAALSEAIAPQK-----NDYTAIVLHGK--ARKV---YKVAASEADQEYH

DmA2M-4 SQDTVVGLQALLMFAERFSSQG-----NNLQIGFHYGE--GAETI--INVNAENSLALQT

DmA2M-3 SQDTVVGLTALIKFAEKSGYEA-----AKWEVTVSNKG--KREKTEKLNTSEENDLLLQT

DmA2M-2 SQDTVVGIMALTKYELQSHAST-----EAIDIEFWHLN--EDKKH--VRVTKENEFKVQT

DmA2M-1 TQDTVIGLQALTKFAYKTGSGS-----GTMDIEFSSAG--ESKNT--IKVNPENSLVLQT

SmA2M-2 TQDTVMGIQALAKFAASLTPPA----GSKLDISVSYDS--NKTD---FAITKETALILHR

IsA2M-1 TQDTVIGIQALARLAASVVSQT-----IAVDASVKYGD--GRKRT--LKIHSGNALVLQR

TuA2M-2 TQDTVIALQALSSIASHLTSST-----QNIDVTFKFGNVTEKQTSRTFQIRDNNAQVLQK

SmA2M-1 TQDTVVGIQALTMLAKRIVDSQ-----IYIDVMFQYDN--EQKN---VHLDKDNSMILMK

AgA2M-1 TQDTVVGLQALSKMAAQLSSSE-----ADMSLKVIITG--EQEKC--LQVNGGNILVLQK

AgA2M-2 TQDTVVGLQALAKLAAKITSPN-----NDVTLTAKINE--NQEKR--MTVNAENGMILQK

TcA2M-2 SQDTFVGLWAIYKLVLKLATN------VNMQVEFTYGK--DQRHN--FNVNKNNAMIVQK

DpA2M-1 TQDTVIGLYALAKLAEKITVPN-----TNINVKIKHDT--GAET---FSLNRENAMVLQK

RpA2M-1 TQDTVVALGALAKFAAKIIVPN-----TDIAVSFTYGK--DVTKE--FKINSANSIILQK

NvA2M-1 TQDTVVGLYALAKLGEKLRTNV-----YDVQVRITTDV--GESKE--ININSRNFMIVQK

AmA2M-2 TQDTVIGIQALAKLGEKLITKNNDIQNNNISVTFAYEE--GQNQ---MNINSDNSMILQK

CfA2M-1 TQDTVIGLQALAKLAEKLSKDT-----SSVRIAFKYGR--DGQGY--MNINSGNSMILQK

PhA2M-1 TQDTVIGIFALAKLAEKITSPN-----FNVQAVFHYKA--GSQAQTTINVNTQKAMILQK

ApA2M-1 SQDTVITLYALSQMAERITPGT-----LKLSATFSYMK--NGQSE--LKVTKDNAMVLQL

TcA2M-1 TQDTVVALDALTKYSKFLSHKT------DININVVALE--SAHN---FVMTDKDRLKSKK

NvA2M-2 TQDTVLGLEALTKYALKMANAS----ATELSVLLTAN---DMEKL--FKINDENRMLLNR

AmA2M-1 TQDTILGLEALTKYAMIVHHNN----ITDLSVLVTASK--EVDDV--YKLQDENRVILKQ

CfA2M-2 TQDTVLGLEALTKYAAAMSNDN-----TDLSVLVTGN---EVDQL--YRMHNDNRMVLTQ

: : *:

ApA2M-2 LHLPSL-----PTKVFVYATGAGCSTIQGRVLYSTYTTA---------------------

TuA2M-1 FEINNE-----CDYVDMITRGTGLGSVRVKYKYNVLEAP---------------------

SmA2M-3 LELPAG------GKIHVDVEGKGIATMSLSLHYNIESSKKDQCKFDLKIQTTEIEDIIMP

DpA2M-2 MDIPNA-----WGTVKIQARGAGYAIAQLSLQYNVDVNR---------------------

AgA2M-4 AQQGVDE----TAQLELHVAGIGFGLLQVVYEYGVDLRN---------------------

AgA2M-3 VDIPED-----TKKLEINVGGIGFGLLEVVYQFNLNLVN---------------------

AgA2M-5 DVLPGD-----SKLVRFSANGRGFGMFTVAFQYGIDVRN---------------------

DmA2M-4 VELPNN-----LKNLSVSATGRGMALAQVSYTYNTNVTS---------------------

DmA2M-3 VEFPQG-----TKSLEFEAKGTGAAMVQISYQYNLVEKE---------------------

DmA2M-2 HQLPEN-----TNEVKLLAKGQGRAQVQLTYRYNVATKE---------------------

DmA2M-1 HDLPKS-----TRKVDFTAKGTGSAMVQLSYRYNLAEKE---------------------

SmA2M-2 EQLPRT-----TRDLTISASGNGVGVVQVSWSYNVLTTE---------------------

IsA2M-1 IELPSD-----LKYVEIESSGFGVAIIQVSWSFNLAVSS---------------------

TuA2M-2 FEMTDARGETIPDHVEIVTQGTGVAVVQVSWRYNLAVSA---------------------

SmA2M-1 EEIPST-----VKMVNITATGRGFAIVQVSYSYNIMVSK---------------------

AgA2M-1 HELAAN-----TRKLEMIATGTGCALFQLSYKYNIKDVD---------------------

AgA2M-2 FELPSA-----ARNIEIQATGSGFAVVQLSYKYNMNVTG---------------------

TcA2M-2 LQLPKD-----IREVNVTAQGKGLAVFRVSYEYNMNVTG---------------------

DpA2M-1 FKLPPK-----TSHVEISAVGSGFSIIQVSTSYNLNVTG---------------------

RpA2M-1 QEIPKQ-----IREVNITARGSGFAVAMVAYSYNVNVTG---------------------

NvA2M-1 HLLLSR-----TRAINITATGTGFALVQVASRYNLNVTG---------------------

AmA2M-2 QMLSRK-----TRLVNITATGNGFVLVQVTYQYNLNVTG---------------------

CfA2M-1 QILPSK-----TRFVNITASGKGFVLVQVSYQYNLNVTG---------------------

PhA2M-1 HELPKK-----VREINITATGSGFAIVQVSYRFNLNVTG---------------------

ApA2M-1 IELPKR-----TRILNVTATGTGLAIIKVSYRYNVNVTG---------------------

TcA2M-1 IVLKNP-----ANKVRVEVQGQGCVLIQAITSYNVKQLR---------------------

NvA2M-2 VELPTL-----PTTLEIFAEGEGCLLVQSSLRYHKAKAS---------------------

AmA2M-1 IRLPIL-----PTIVEIFAQGEGCVLIQSNLKYNVASST---------------------

CfA2M-2 IRLPVI-----PTIIEIFAEGEGCVLVQSNIKYNVAHAT---------------------

: . * * :

ApA2M-2 -----ENDKKP-FDLWSGVTDVI------------------------------QPSK-GF

TuA2M-1 ------EKLCG-FEL----DVNV------------------------------TQAI-DS

SmA2M-3 QMPKPEFDG---FDL----LPEMVVRTITSSEDQKRKFGYKVVVQKEQEDDEYEYEYDNE

DpA2M-2 ---FVTPPAVRAFSV----VPRL------------------------------SFSGRNN

AgA2M-4 -----FTAQ---FVL----ELQK------------------------------SVTN-AN

AgA2M-3 -----FENR---FQL----DLEK------------------------------QNTG-SD

AgA2M-5 -----IEHG---FSL----RLVD------------------------------QFSN-EA

DmA2M-4 -----AWPR---FVL----DPTV------------------------------NRNS-HA

DmA2M-3 -----PKPS---FKI----QTTV------------------------------LPES-SP

DmA2M-2 -----ARPS---FKL----TTTV-------------------------------KKS-HK

DmA2M-1 -----KKPS---FKV----TPTV-------------------------------KDT-PN

SmA2M-2 -----DRPA---FSI------HV------------------------------NATG-EN

IsA2M-1 -----EAPA---FFL----NPLL------------------------------DKTS-TE

TuA2M-2 -----EEPA---FFL----NPIL------------------------------GKAS-TD

SmA2M-1 -----ENPS---FQV----NPFV------------------------------DRSS-TK

AgA2M-1 -----NSPR---FTL----KPEA------------------------------KQGS-IK

AgA2M-2 -----EWPR---FVL----DPQV------------------------------NANT-NP

TcA2M-2 -----PWPM---FTL----DPQV------------------------------DKNS-NK

DpA2M-1 -----EWPL---FTL----DPQL------------------------------FKNA-NQ

RpA2M-1 -----AWPL---FTL----DPQV------------------------------DKNS-DH

NvA2M-1 -----AFPL---FTL----DPQV------------------------------DKIS-TN

AmA2M-2 -----AWPL---FTL----DPQV------------------------------DKNS-NA

CfA2M-1 -----AWPL---FTL----DPQV------------------------------DKNS-NP

PhA2M-1 -----AWPL---FTL----DPQV------------------------------DKNS-DN

ApA2M-1 -----AWPL---FSL----DPQV------------------------------DKNS-NA

TcA2M-1 -----NGDA---FKL----DMEV------------------------------LPVS-NI

NvA2M-2 -----GSEA---FDL----STST------------------------------ASVS-TA

AmA2M-1 -----GSDA---FDL----SAEV------------------------------RSVG-YG

CfA2M-2 -----GSEA---FDL----SVNA------------------------------ASST-WV

* :

ApA2M-2 TDEIYGHAITLRLK----------------------------------------------

TuA2M-1 PEQIILSDSDIQLDDLFNITMLAEIGVLNDRLVQVESVVDSTLTDNNDSSSSKTGPSYTA

SmA2M-3 NDLAHESRHIVKLE----------------------------------------------

DpA2M-2 SH--------INYD----------------------------------------------

AgA2M-4 HQ--------LQLE----------------------------------------------

AgA2M-3 YE--------LRLK----------------------------------------------

AgA2M-5 YT--------LQLQ----------------------------------------------

DmA2M-4 DY--------LHLS----------------------------------------------

DmA2M-3 AN--------LELS----------------------------------------------

DmA2M-2 GR--------LILG----------------------------------------------

DmA2M-1 QL--------LIVD----------------------------------------------

SmA2M-2 EE--------LIIN----------------------------------------------

IsA2M-1 SY--------LQLS----------------------------------------------

TuA2M-2 NF--------LQLN----------------------------------------------

SmA2M-1 DR--------LQLN----------------------------------------------

AgA2M-1 SC--------IDLS----------------------------------------------

AgA2M-2 DY--------LHLS----------------------------------------------

TcA2M-2 DH--------LQVS----------------------------------------------

DpA2M-1 NR--------MQLT----------------------------------------------

RpA2M-1 NH--------LQLS----------------------------------------------

NvA2M-1 DH--------LQLS----------------------------------------------

AmA2M-2 NH--------LQLS----------------------------------------------

CfA2M-1 NH--------LQLS----------------------------------------------

PhA2M-1 NH--------LQVS----------------------------------------------

ApA2M-1 NH--------LQLS----------------------------------------------

TcA2M-1 DK-----CSITTLS----------------------------------------------

NvA2M-2 DQTPNDGCSIQRLT----------------------------------------------

AmA2M-1 NE-----CSLQEIT----------------------------------------------

CfA2M-2 DE-----CSMQKIT----------------------------------------------

ApA2M-2 ---------------------TCFRM-------NNETEDAIRLEVKLFSGYYFDK-----

TuA2M-1 NEKAESNLKLSGASKVTNKLTVCAKRFDSV------DSGMVILEVGILSGFVPDE--NDL

SmA2M-3 ---------------------ICVRHLG------DKPAGMSILDIGIFTGYKPVK--DDL

DpA2M-2 ---------------------ICSSWTNQR---ESNQSGMAVLDVAVPTGYYMQQ--QVL

AgA2M-4 ---------------------VCSSFTPQL---SDGRSNMVLVEVNFPSGYTVEQRGQPI

AgA2M-3 ---------------------VCASYIPQL---TDRRSNMALIEVTLPSGYVVDR--NPI

AgA2M-5 ---------------------VCTSFSPQL---MHTRSNLALVEVNFPSGYVVSR--KSL

DmA2M-4 ---------------------ACASFVSVVGE-NEQRSNMAVMEVHLPSGFVVDR--DTL

DmA2M-3 ---------------------VCVDYVEEG---ESKESNMAILEVSLPSGYTADE--DSF

DmA2M-2 ---------------------ICGTYTPIAASERNKTTNMALMQVQLPSGYVCDI--EPF

DmA2M-1 ---------------------VCAEYVPLEDADKDKDSNMAVMEIALPSGFVGDS--TSL

SmA2M-2 ---------------------SCAKYIYKV----NGESNMAVMEIEFPSGYVADL--DHL

IsA2M-1 ---------------------VCTHYRG-----EGEASNMAVMEVGLPSGYLFDF--DTL

TuA2M-2 ---------------------LCTYYKA------GLATNMAVMEVELPSGYSADV--DAL

SmA2M-1 ---------------------VCAAYAE-----NGATSNMAVMEVTLPSGFVIDR--DSL

AgA2M-1 ---------------------ITTSFIPKE---DQAVSNMAVMEVDMPSGFIVES--DTL

AgA2M-2 ---------------------VCASFVPSA---GQNVSNMAVMEVGFPSGFTADS--DTL

TcA2M-2 ---------------------ICTGFVSRNLS-ETPESNMAVMEVNLPSGFTADI--DSL

DpA2M-1 ---------------------ICSSFVG-------EESNMAVMEISLPSGYVMDE--DSL

RpA2M-1 ---------------------ICSAYVG------GNESNMAVMEVSLPSGYVVDQ--DSL

NvA2M-1 ---------------------ICSGFIPTK---EANESNMAVMEVSFPSGFTVDQ--DAL

AmA2M-2 ---------------------ICSGFVPTK---EANESNMVVMEVNLPSGFTVDK--ESL

CfA2M-1 ---------------------ICSGFVPTK---EANESNMAVMEVSLPSGFTVDR--DSL

PhA2M-1 ---------------------ICSKFVGTK---ETNESNMAVMEVSLPSGFVVDS--DSL

ApA2M-1 ---------------------VCSGFRG------GNDSNMAVMEVTLPSGFTVDN--DAL

TcA2M-1 ---------------------PCFKYNG-----PDHIANMAILEVGLPSGYQADR--ASL

NvA2M-2 ---------------------VCTRYKL-----PDEESNMAVLEIAMVSGFRPDR--ASL

AmA2M-1 ---------------------ICSRYKM-----ADEESNMALLEVGIISGYVPDR--ASL

CfA2M-2 ---------------------ICTRYKM-----ADGESNMAVLEVGMISGYIPDR--TSL

. ::: . :*:

ApA2M-2 ---I-SSASVSDV-HHDSHSNHIWFVFAKVKSSCIVCVSYTAKSIQKVTGL-RPAVAKVY

TuA2M-1 VALK--KNNPFIS-SYEKTARSVIFYLEDISSKQQYCLNFKIYQENKVANL-QSAMVKIY

SmA2M-3 VKLL-KDATIS---QYEPSDTSVVLYIDTVPHDEDLCIKLRTMQEISVGKV-QPTTVKIY

DpA2M-2 DEYI-LSRKVRNLRRAKFLDHKVVFYFDALDGDD-TCVKFTFERWHPVANMTRYLPVRVY

AgA2M-4 TGAT-KHNPIQKT-EVRFGATSVVVYYNSMGPER-NCFTITAYRRQKVTLK-RPAYVLV-

AgA2M-3 SEQT-KVNPIQKT-EIRYGGTSVVLYYDNMGSER-NCFTLTAYRRFKVALK-RPAYVVVY

AgA2M-5 VDET-RRNPFKDV-EVRYGQTSLVIYYETLGPEE-NCFSVTANRLFRVAFH-RQAYVMVH

DmA2M-4 PTLE-SSERIKKV-ETQNRNTKVVIYFDYLDRRE-VCPTLHAYKTVKVTKH-RPVAVVMY

DmA2M-3 ADIR-NIERVRLV-ETKNGDSVVVIYFENLAKNEEKCIRIEAYRTHAVANQ-KPSSVVLY

DmA2M-2 ADIE-AISDVKRV-ETKNEDTEVHIYFEKLSPGDRKCLTLEAIYTHAVANL-KPSWVRLY

DmA2M-1 GKIQ-AVDRVKRV-ETKNSDSTVVVYFDSLTPGDVRCLPLEASKAHAVAKQ-KPASVSLY

SmA2M-2 PSIN-EEKKIKRV-ETKNGDTSIVVYFDKIGKE--VCTSARGHRAFKVAKI-KPALVSVY

IsA2M-1 SSIH-RTKEVRRV-ESQDSDTNVVIYFDRIGREE-LCVTVPAHREHKVANQ-KPVPVKVY

TuA2M-2 QSIKSQGNGIKRI-ESHNGDTNVVIYFDRLTREE-LCLTVPAHRTQKVANN-KPVPVTLY

SmA2M-1 PALH-RVDEVKRV-DIKDRDTTVVVYFDKLDNKL-VCPTIKAYRTYRVAKQ-KATAVYVY

AgA2M-1 KQLK-QHEMVKKV-ETKRSDTTVVLYFDNIGEEA-VHLQMSAFQKHEVENA-KPANVIIY

AgA2M-2 PSLE-NMPFIKKV-ETKDGDTTVVLYFDSLDQRE-LCPTISAFRTHKVAKQ-KPAPVVIY

TcA2M-2 PSLE-VSQNVQKV-ETSNGLTRVTLYFNNVSSVNEYCPTVSAFRTHKVANQ-KPVSVIIF

DpA2M-1 PSLR-AIKDVKKV-ETKEGGTGISLYFDKMTRNT-VCPTVQAYRVFKVAEQ-RKVPVVMY

RpA2M-1 PSLE-ISQDVKRV-ETKDRDTVVVLYFDKMTAKE-YCPTISAFRTHKVAMQ-RPVPVTVY

NvA2M-1 PSLE-LSQNVKRV-ETKNGDTMVVLYFDKMVHDKSYCPTVSAYRTHKVAKQ-KPVPVSIY

AmA2M-2 PSLE--------------GDTIVILYFDEMSRQE-YCPTVSAFRTHKVAKQ-KPVPVTIY

CfA2M-1 ------------------------------------------------------------

PhA2M-1 PSLR-VSQNVKRV-ETKDSDSVVILYFDKLIKQE-YCPTISAYRTHMVANQ-KPVPVIVY

ApA2M-1 PSLR-LSNNIKRV-ETKDGDTVVMLYFDKMMAEE-YCPTISAFQTHKVANQ-KPVPVTVY

TcA2M-1 YKLI-DESSVKM---FEELEEKIVLYLTKLGNRQ-MCVNFNINENAIVKSR-SNSTVKLY

NvA2M-2 HDLL-DEHATGVK-RFEENDDTVAIYFDKLTAQK-TCISFQAIRENVVDHA-EPANIKLY

AmA2M-1 HSLL--DPSSKVK-LFEEDQDIVTIYFNKLTGQK-TCISFRIIQEYFIDHL-KPANIKLY

CfA2M-2 HSLL-EDPATKVK-RFEEDRDVVTIYFDKLINQK-TCISFMVTRENVVDRL-EPANVKLY

ApA2M-2 VVSRPDQSSYKLFH----------------------------------------------

TuA2M-1 DYYKKGQGCSQLYHSTRRT-----------------------------------------

SmA2M-3 DYYEPDKSCQKFYTPDG-------------------------------------------

DpA2M-2 DYYAPERFNETVVQTYEL------------------------------------------

AgA2M-4 ------------------------------------------------------------

AgA2M-3 DYYNTNLNAIKVYEVDKQNLCEICDEEDCPAECKK-------------------------

AgA2M-5 DTYDEKFRAIKFYQVPH-------------------------------------------

DmA2M-4 DYYDSARRARQFYRAPKSNICDICEHANCGDLCEKAEKRESKRPDDYTAIAGHSSGSRHT

DmA2M-3 DYYDTNKKATEYYS-IKSKLCDICEGDDCKSKC---------------------------

DmA2M-2 DYYATERSATEFYH-VDTSLCDICHGNECGNMC---------------------------

DmA2M-1 DYYDTERKATEYYQ-VKSSLCDICEGADCGEGCKKD------------------------

SmA2M-2 DYYDPIKRGEQFYNAP--------------------------------------------

IsA2M-1 DYYDLARSARMFYSPYKT------------------------------------------

TuA2M-2 DYYNRHESARIMYEPKM-------------------------------------------

SmA2M-1 DYYDQAKAARYFFQ----------------------------------------------

AgA2M-1 DYYDNTRCARSFY-----------------------------------------------

AgA2M-2 DYYDNSRIARQFYDGPKASLCDICENEDCGEACSIRSQKQRSSDSPSRQPTVEGTMQSGS

TcA2M-2 DYYDSSRRARQFYRSRTSTLC---------------------------------------

DpA2M-1 DYYDSSRRARVFYEPV--------------------------------------------

RpA2M-1 DYYDQSRQARVFYTPSVTS-----------------------------------------

NvA2M-1 DYYDSSRRARVFYEPKMTTLCD--------------------------------------

AmA2M-2 DYYDSSRRARVFYEPRKATLC---------------------------------------

CfA2M-1 ------------------------------------------------------------

PhA2M-1 DYYDSSRRARTFYQPLKATIC---------------------------------------

ApA2M-1 DYYDQSRRARVFYAPRKAT-----------------------------------------

TcA2M-1 DYYKPEYQVSQFYKIKENC-----------------------------------------

NvA2M-2 DYYQQELTVSTSYAFMDVC-----------------------------------------

AmA2M-1 DYYQQELTVSTNYKIPS-------------------------------------------

CfA2M-2 DYYQQELTISSSYSFAPTCSS---------------------------------------

1. **I51 IC.**

TuSCPYI-6 ----------------TKVKPDVVIDTSLLNGKTLSFEDFQKVLPNGINLL-G-------

BmSCPYI-6 -------------------TSVGGTIVNDHNCDVLLPAQVFLDEPLF-QYFMA-------

SmSCPYI-2 --------------------DVA---TPVFNGNHLKPSEMKSM-PEI-EFP-S-------

IsSCPYI-6 -------------------EHDAEHVMPVYHGNHILPSEASQA-PSV-SFD-S-------

RpSCPYI-3 ---------------NIDY-INGNSVVPAYWGNVLKPEEAQSV-PNV-TFD-S-------

DmSCPYI-9 EHLFGSAYFVPRVPLNISYQLDGDSLAPVYNGNVIKPTEAAKA-PQI-DFD-GLVDPITG

AgSCPYI-7 --------FVPRVALDIQY-QAGELLHPVKYGNVLKPSETQAA-PQV-QFD-GNFNFTGQ

ApSCPYI-5 --------------ISYDYNSKK---VPVYRGNIIKPNEALYS-PKV-NFE-A-------

BmSCPYI-5 -------------NIDVLYNLKDGTFLPVCAGNVIKPTEALEA-PII-TYE-S-------

AmSCPYI-3 ------------------YKIDDDTSVKVYTGNVIKPAEASEM-PYV-EYK-V-------

NvSCPYI-8 ------------------F-GSEDKLARVHRGNLIKSYEAKNA-PSV-TYK-A-------

CfSCPYI-3 ------------VPLDISYELENDQLTRVYNGNVIKPSEASNV-PNV-KYN-A-------

PhSCPYI-3 -------------LVAPVY-----------WGNIVKPDEAFIK-PQV-KFK-S-------

TcSCPYI-7 -----------KDNVQ----------HPVYYGNVIKPADASNK-PEV-HYE-S-------

TuSCPYI-1 ---------------------SG---ISI-CGTELDSLQTFDE-PLV-KFP-A-------

TuSCPYI-3 -----------LNLIEVKY--SS---ASVSCGTELKSLQTAEK-PMI-KYP-T-------

TuSCPYI-2 -----------LDLIEVTY-PNAICRESVSCGNELKSFQTLEE-PII-KYP-S-------

IsSCPYI-2 ------------EGVKVHYPSSN---AVVKMGNVIRKEDAAQA-PTI-EFK---------

IsSCPYI-4 -----------KQNVVVRY--QN---CDVSLGNTLRPEEAASS-PDSVVFQ-T-------

IsSCPYI-1 ---------VPQGVANVNY-GDG---TAVCMGNTISPQIASNK-PTV-SFEAQ-------

IsSCPYI-3 ----------PQGVVNVNY-GNG---TAVCMGNTISPQDTSNK-PTV-SFEAQ-------

TuSCPYI-5 -------SEHYLALVDIEY-AGD---LQVSCGNELTPNQTAKE-PVVFNYH-S-------

PhSCPYI-2 -------------HLKV---------DKENCGSEYVKSEWQAE-PKV-NFVDA-------

DpSCPYI-4 ------------YIVNVDY-ADH---ACVHMGNQLVPRQTQLQ-PQQVNFP-T-------

DmSCPYI-8 ------------KVISVLY-PCD---IDIKPGIMVVINETLKQ-PII-RFK-A-------

DmSCPYI-7 ------------ELLRIKY-DNT---IDIEEGKTYTPTELKFQ-PRL-DWN-A-------

AgSCPYI-5 ----------PDCWARVSF-KSG---RQAEGGNRLTPTQIRNP-PVV-SWN-A-------

DmSCPYI-6 ---------GPQEFLNVTY-HGH---LAAHCGKVLEPMQVRDE-PSV-KWP-S-------

AgSCPYI-6 ----------PDAFAKVVY-RGK---KLVDAGKELSPAEVREE-PKV-EWY-A-------

TcSCPYI-6 ------------SQITIIY-PK----KTVDLGQEFAPQDVREQ-PQV-HWE-A-------

TcSCPYI-5 -----------SAKITITY-PGG---RTVEFGKELKPEEVKDE-PQV-CWD-A-------

TcSCPYI-4 ------------AHLFVTY-PNG---KKVHLGEELTPSEVKDE-PQV-KWD-A-------

NvSCPYI-6 -----------TKPFSIAY--EG---KSVQLGEEWTPTGTIPI-PTV-KWD-F-------

IsSCPYI-5 ------------SLSCRRF-LRP---SCVRFQSCAAKSKMEAH-QVV-------------

NvSCPYI-7 --------NAPNETIEIKY--GD---KEVKLGNEFTPSETKEI-PEV-HYK-H-------

AmSCPYI-2 ---------APTEKIEVKY--GN---KSVDLGNELTPTETQQI-PEI-HYK-H-------

CfSCPYI-2 ---------APIEKIEVKY--GG---KVVDLGTELTPTETHEI-PEI-HYK-H-------

DmSCPYI-2 -----------AGTIKVIY-GDD---LEVKQGNELTPTQVKDQ-PIV-SWSGL-------

NvSCPYI-5 -------------YYKVSY--NN---KEFGFASELTPTEVKDA-PTHIGWG-L-------

DmSCPYI-3 -------------KATITY-PSG---VQVELGKELTPTQVKDQ-PTV-VFD-A-------

DpSCPYI-3 ---------APPETLIVEY-EGG---LIVNGGNQLTPTQVQNK-PVKIQWT-F-------

NvSCPYI-4 -----------NELLTVTFKDSNDKDKDVQFGDELTPTLVKDP-PAM-SWF-S-------

AgSCPYI-3 -----------KEFAKITY-PSG---VTVSGGNELRPTQVKDQ-PRV-EWT-A-------

AgSCPYI-2 --------VAPEQTIKITYPQSD---VEVSLGNQLTPTQVKAR-PKL-CWE-V-------

SmSCPYI-1 -----------QGILEIKF--GN---HELKMGNILTPTQVQNK-PTHIFYV-A-------

DmSCPYI-1 ----------PNQLLKVTY-SNN---LVAKDGVELTPTQVKDQ-PVV-EWD-A-------

AgSCPYI-1 ---------PPESLLHVTY-PGG---LRVNLGNILTPTEVKHV-PEV-AWPEA-------

TcSCPYI-3 ----------PSKLLKVEYKKTN---KEVHLGNELAPKDVRDA-PSV-TYS-G-------

BmSCPYI-3 -----------SELLNIQY-SNG---INVDLGKELTPTQVKDA-PTV-KWA-S-------

NvSCPYI-3 ------------DLLSVSF-EDK---VLRFIGEELTPTQVKDV-PSV-AWK-S-------

DmSCPYI-4 -----------ATLLTVTY-GGG---QVVDVGGELTPTQVQSQ-PKV-KWD-A-------

BmSCPYI-2 ----------PTKNIELKY-PSG---AIASQGNELTPTQVKDQ-PSV-TFE-A-------

NvSCPYI-2 ------------ELLSVTY--ND---RPVEFSMELTPTQVKDA-PAV-TWS-P-------

DpSCPYI-2 ------------EAVHVSY-DSG---VHVDQGKELTPTQVKNE-PTKVNWL-A-------

TuSCPYI-4 ------------HTVEVKY-PSG---AEVKFGNELTPTVVKDV-PTHISWP-S-------

RpSCPYI-1 ----------PAQHLKVTF--EN---NAVNDGNVLTPTQVKNQ-PSV-EWD-A-------

RpSCPYI-2 --------RAPAQKLKVTF--GG---KEVDDGNILTPTQVKNQ-PSV-EWN-A-------

DpSCPYI-1 -----------AATITIKY-DSG---VAVDGGNELTPTQVQNQ-PIHIEWP-V-------

PhSCPYI-1 -----------AEVAEIKY--GN---LALSLGNELTPTQVKNP-PSVLKWK-A-------

ApSCPYI-4 ---------------QVYY-PSG---LKAELGYELTPTQVKDQ-PSV-RWN-A-------

NvSCPYI-1 --------------VKVSY-PSG---VSVDIGKELTPTQVKDQ-PSV-EWD-A-------

AmSCPYI-1 ----------PENVLKVTY-PNQ---ISVDIGKVLTPTQVKDK-PNV-TWN-G-------

CfSCPYI-1 ---------VPANVLNVTY-PNN---LSIEIGKVLTPTQVKDQ-PTV-QWD-G-------

ApSCPYI-2 -----------KEIIQVNY-SNG---AKALLGNELTPTKVKDQ-PLV-SWN-A-------

ApSCPYI-3 ------------EVVQVNY-MSG---AKALLGNELTPTKVKDQ-PSV-SWN-A-------

TcSCPYI-2 -------------VAEVHY-PKG---VKVQLGNTLTPTQVKDP-PTV-KWE-A-------

DmSCPYI-5 -------------TAVVEY-PGD---IVVKPGQVLTPTQVKDE-PCV-KWE-A-------

AgSCPYI-4 -----------AEVAKVTY-PSG---AVVSEGNVLTPTQVKDV-PKV-EWN-A-------

TcSCPYI-1 -----------ETTAEVTY-PSG---VKVEMGNELTPTQVKDV-PTV-KWN-A-------

ApSCPYI-1 ----------PSDKIQVSY-PSG---VIVDMGNELTPTQVKDE-PSV-TWP-A-------

BmSCPYI-1 ------------ALLQVKY-PSG---VEVKEGNELTPTQVKDE-PSV-KWD-A-------

BmSCPYI-4 -----------AALLQVKY-PSG---VEVEEGNELTPTQVKDE-PSV-KWD-A-------

TuSCPYI-6 ----D-SNNFYT----LALLNLDSQFGKSD---PVCHWMV-----------SN-IHNQSD

BmSCPYI-6 ----D-SKKFYT----IILVDPDSPPQVDGEF--YLHMLK-----------SN-I--PGL

SmSCPYI-2 ----D-PQSLWT----LVATNLDCHFEQDS--FEYLLWFVYVLQLYNLHVRGN-I--RGN

IsSCPYI-6 ----E-PDALWS----LVLTSLDGHLLDND--KEYLHWFV-----------VKYI--PGA

RpSCPYI-3 ----K-KDDLWT----LVMTTPDDYFSPN---MEYCHWFV-----------GN-I--RDG

DmSCPYI-9 QAA-G-QDTYWT----LVASNPDAHYTNGT--AECLHWFI-----------AN-I--PNG

AgSCPYI-7 PASEE-QQSWWS----LLLTNPDGHFEDSE--KEYCHWFV-----------GN-I--PNG

ApSCPYI-5 ----P-EKTLWT----LMLTNPDGHLHKEN--SEYIHWLV-----------GN-I--PGG

BmSCPYI-5 ----D-DNALWT----LAFTSLDGHLYENE--KEYVHWLV-----------AN-I--PGN

AmSCPYI-3 ----E-DDTLWT----LVMCTPDGNLENSN--NEYCHWFL-----------GN-I--PGN

NvSCPYI-8 ----E-PDSLYT----LLLTTPDGNF-SDPSY-EYCHWFI-----------GN-I--PGN

CfSCPYI-3 ----K-AGSLWT----LIMTTPDGNLNTC---NEYCHWFI-----------GN-I--PGN

PhSCPYI-3 ----D-PNELWT----LSLVCPDGHLTIQN--GEYIHWLV-----------GN-I--PGG

TcSCPYI-7 ----D-DKTLWT----LIMTNPDGHFTQQD--KEYVHWFV-----------GN-I--PGN

TuSCPYI-1 ----E-TNTNYT----FMMLDIDAPSPSAPIMRSFIHWMV-----------VN-A--LGG

TuSCPYI-3 ----K-SNTLYT----FMMLDLDEPSPSLPTFRSVIHWLV-----------IN-V--KRD

TuSCPYI-2 ----E-SNTLYT----FMMLDPDALSPAAPTLRSYIHWMV-----------IN-V--ERD

IsSCPYI-2 ----ERRNNLYT----IMMLDPDAPSRRNPKHRSWVHWLI-----------VN-AEGPGT

IsSCPYI-4 ----H-SNSLYT----LVMVDPDAPSRQNPKMRFWRHWLL-----------VN-V--PSN

IsSCPYI-1 ----D-ALPPYT----LVMVDPDAPSASEPIYRSYLHWVM-----------VN-A--PSS

IsSCPYI-3 ----D-ASPPYT----LVMVDPDAPSASKPIYRSWLHWVV-----------VN-V--PSS

TuSCPYI-5 ----G-WYEHYS----VVMIDPDAPKDKG----TVLHWMV-----------VN-I--PGS

PhSCPYI-2 ----K-HDKSYT----VMCVDPDPPGYEKGQY--WLHWLV-----------SN-V--KGD

DpSCPYI-4 ----NGSGGLFT----LMAIDPDVPSRNNSIYSEFLQWLV-----------VN-I--PDE

DmSCPYI-8 ----D-PEHYHT----LMMVDLDVPPDNN---TEWLIWMV-----------GN-I--PGC

DmSCPYI-7 ----D-PESFYT----VLMICPDAPNRENPMYRSWLHWLV-----------VN-V--PGL

AgSCPYI-5 ----N-ERALYT----LILTDPDVPSRDDPRYREFIHWAV-----------GN-I--PGN

DmSCPYI-6 ----A-PENYYA----LLMVDPDVPNAITPTHREFLHWMV-----------LN-I--PGN

AgSCPYI-6 ----D-PTALYT----LIMTDPDSPSRMEPWNREFAHWLV-----------GN-V--PGR

TcSCPYI-6 ----D-PEKYYT----LVMTDPDAPSRRCPFVAEVIHWLV-----------GN-I--KGC

TcSCPYI-5 ----A-PDKYYT----LLMFDPDAPSRMEPKIADVKHWLV-----------VN-I--QGC

TcSCPYI-4 ----A-STKYYT----LVMFDPDAPSRSDPSFADVKHWLV-----------GN-I--QGG

NvSCPYI-6 ----E-SSTFYT----IIMIDIDPPSRAKANFREFVHWFV-----------VN-I--PGN

IsSCPYI-5 ------PDVIDTVPPGVVQVNPDAPSRQSPKYREWHHWLV-----------VN-I--PGV

NvSCPYI-7 ----E-GGVLYT----LVMTDPDVPVRGYN--REWQHWVV-----------GN-I--PED

AmSCPYI-2 ----E-GGVLYT----LVMTDPDVPTRKGYN-REFRHWLV-----------GN-I--PEE

CfSCPYI-2 ----E-GGVLYT----LVMTDPDAPRRGGYN-REFRHWLV-----------GN-I--PEE

DmSCPYI-2 ----EGKSNLLT----LLMVDPDAPTRQDPKYREILHWSV-----------VN-I--PGS

NvSCPYI-5 ----D-SSSFYT----LIMNDPDAPSRQDPKMREFLHWAV-----------VN-I--PGD

DmSCPYI-3 ----E-PNSLYT----ILLVDPDAPSREDPKFRELLHWLV-----------IN-I--PGN

DpSCPYI-3 ----Q-DGDLFT----LCLIDPDAPSRDLPLLREFQHWIV-----------VN-V--PGN

NvSCPYI-4 ----E-DSAYYT----VAMVDPDAPSRDDPNLREMLHWLV-----------CN-I--PGG

AgSCPYI-3 ----K-PDAYYT----LFMVDPDAPNRQEPKFREIGHWLV-----------GN-I--PGT

AgSCPYI-2 ----E-PSALYT----LLMADPDAPSRSNPEMRSWKHWLV-----------GN-I--PGA

SmSCPYI-1 ----E-SGAFYT----LYMTDLDVPSRKEPKCRECNHWLI-----------IN-I--PGN

DmSCPYI-1 ----Q-PGEFYT----LIMTDPDAPSRAEPKFREFKHWIL-----------AN-I--AGN

AgSCPYI-1 ----E-PDAYYA----LVLTDPDAPSRTAPKFREWHHWLV-----------VN-I--PGM

TcSCPYI-3 ----D-PHAFYT----LVMTDPDAPSRKNPKAKEWNHWLV-----------GN-I--PGS

BmSCPYI-3 ----K-ENEYYT----LAMVDPDAPSRENPKFREWHHWLV-----------GN-I--SGG

NvSCPYI-3 ----E-ASGFYT----ICMTDPDAPSRSEPKFREFLHWLV-----------VN-V--PGE

DmSCPYI-4 ----D-PNAFYT----LLLTDPDAPSRKEPKFREWHHWLV-----------VN-I--PGN

BmSCPYI-2 ----E-ADAFYT----LVFTDPDNYDGPELVYREWHHWLV-----------GN-I--PGG

NvSCPYI-2 ----E-ASTFYT----LCMTDPDATSRKNPILREVLHWLV-----------TN-I--PGN

DpSCPYI-2 ----E-EGSNYT----LCMTDPDAPSRAEPSKREVLHWLV-----------VN-I--PGN

TuSCPYI-4 ----E-EGALYT----LCMTDPDAPSRQNPKYREWHHWLV-----------VN-I--PDN

RpSCPYI-1 ----D-PSALYT----LCMTDPDAPSRKDPKFREWHHWLV-----------VN-I--PGS

RpSCPYI-2 ----D-PNSYYT----LCMTDPDAPSRENPKFREWHHWLV-----------VN-I--PGN

DpSCPYI-1 ----E-EGAHYT----LCMTDPDAPSRNTPTFREWHHWLV-----------VN-I--PGN

PhSCPYI-1 ----E-EDSFYT----LCMTDPDAPSRKDPKFREWHHWLV-----------VN-I--PGT

ApSCPYI-4 ----E-LYSFYT----LCLTDPDA-----GQLKEFNHWLV-----------GN-I--PGA

NvSCPYI-1 ----D-SSSYYT----LCMTDPDAPSRKDPKFREWHHWLV-----------TN-I--PGK

AmSCPYI-1 ----D-ANTYYT----LCMTDPDAPSRKNPKFREWHHWLI-----------GN-I--PGS

CfSCPYI-1 ----E-TNAFYT----LCMTDPDAPSRQNPKFREWHHWLV-----------GN-I--PGS

ApSCPYI-2 ----D-ANSFYT----LCLIDPDAPSRAEPTNREWHHWLV-----------GN-I--PGG

ApSCPYI-3 ----D-PNSFYT----LCLTEPDAPSRAEPIQREWHHWLV-----------GN-I--PGG

TcSCPYI-2 ----E-SDAFYT----LCMTDPDAPSRKDPKFREWHHWLV-----------VN-I--PGD

DmSCPYI-5 ----D-ANKLYT----LCMTDPDAPSRKDPKFREWHHWLV-----------GN-I--PGG

AgSCPYI-4 ----D-SGALYT----LCMTDPDAPSRKEPTYREWHHWLV-----------GN-I--PGA

TcSCPYI-1 ----D-NNALYT----LCMTDPDAPSRKEPKFREWHHWLV-----------GN-I--PGG

ApSCPYI-1 ----D-PNALYT----LCMTDPDAPSRKEHTYREWHHWLV-----------GN-I--PGN

BmSCPYI-1 ----E-PGQYYT----LAMTDPDAPSRKEPTFREWHHWLV-----------GN-I--QGN

BmSCPYI-4 ----E-PGQYYT----LAMVDPDVPSRKLPINRELQHWLV-----------GN-I--QGN

: . * :

TuSCPYI-6 --GSTVHKEVIS-----FIPPYAFNGFGYHRYVFMLLQSK-EPKELPE------INDVEL

BmSCPYI-6 ALKTKESSKTIGIDYRGYKPPTPSRGIDTHRYITLLYEQA-DGNNFL----PTV----PS

SmSCPYI-2 --DISTGETICD-----YLQPFPAKGSGYQRVVFVLYKQD-EKINYNSL--KRQAPCLNL

IsSCPYI-6 C-NVANGEVVCD-----YMQPFLPRGTGYHRYVFVLYKQE-GLIDYSKF--KLPPKCTSL

RpSCPYI-3 --DLKTGEVIFD-----YLQPLPAQGLGYLRYIFVLYKQE-EILNYNG---LKLKNKNMQ

DmSCPYI-9 --KVSEGQVLAE-----YLPPFPPRGVGYQRMVFVLYKQQ-ARLDLGSYQ-LAAADYGNL

AgSCPYI-7 --DVTSGEELVP-----YLQPFPAKGTGYQRHIFVLYKQT-SRLDFSQ---YRITDAFDL

ApSCPYI-5 --DVNRGETVFN-----YLQPFPAKGTGYQRMIFVLYKQS-SEIDFSSI--KSVSEKIDL

BmSCPYI-5 --AIEKGETLVD-----YLQPFPLKGTGYHRYVFVLYKQD-KTIDYAL---PKVTSSSAL

AmSCPYI-3 --KLEMGEQIID-----YMKPFPARGVGYYRYIFILYKQN-QRLDYVEY--KKDQPCLTL

NvSCPYI-8 --DVAKGEQLVD-----YLRPIPPKGIGFCRYIFVLYKQD-KKIDFSEY--KKEIPCLKL

CfSCPYI-3 --RVEEGEELID-----YLRPIAPYGIGYCRYIFVLYKQD-CHIDFSGY--KKTKPCLNL

PhSCPYI-3 --EVGKGEEIWD-----YLPPFPPRGVGYLRYIFVLYKQE-KKIDFSSL--KNKLPRLEL

TcSCPYI-7 --KIEKGETIVD-----YLQPIPPKGTGYHRHIFILYKQE-KKLDFSDF--KKPGKCLNL

TuSCPYI-1 --QLEPQSTVHP-----YISPMPTPGLGAHRYVFMVFEQP-KGFTIDP-------NATVL

TuSCPYI-3 --DLQSGFNIYS-----YLIPVPTPNMGAHRYVFMVFEQP-KEFAIGS-------NAMIA

TuSCPYI-2 --DLKSGSTIHS-----YIAPTPTPLLGAHRYVFMVFEQP-EKFAVGS-------DAIVL

IsSCPYI-2 G-RVDPDNVIQS-----YKGPGPPAGSGAHRYVFLIFCQGKRRIN------AKAVKQWVP

IsSCPYI-4 C-DLSGADCVTE-----YAGPSPPKGSGPHRYAFLVYTQGSTRISERD---VHVP----E

IsSCPYI-1 D-GFGEGEEAVQ-----YIGPAPPQGSGPHRYVFLVVAQN-GRNISKS-------DVSYS

IsSCPYI-3 D-RFGEGEEAVQ-----YNGPAPPKGSGPHRYVFLVVAQD-GKNISKS---E----VSYS

TuSCPYI-5 --NMTQGEVACP-----YFGPKPPMDHGLHRYISLIYLQS------DEG--PVAVKDWSQ

PhSCPYI-2 --DLAKGDLTKAKHSLPYYGPAPPEGSGLHRYIFLAFEQENDNVELDV---PKV------

DpSCPYI-4 --DIERGDVLAE-----YLGPLPSHKGGQHRFIFLAHKQP-DGSIINTRGLPHAEPCDWA

DmSCPYI-8 --DVAMGQTLVA-----YDNRRTIHGSNIHRIVFLAFKQY-LELDFDE---TFVPEGEEK

DmSCPYI-7 --DIMKGQPISE-----YFGPLPPKDSGIQRYLILVYQQS-DKLDFDE---KKMELSNAD

AgSCPYI-5 --DIDRGETLVE-----YLGAVTPRGTGLHRFVLLVFEHL-QKLDFSAE--PRITAQCGT

DmSCPYI-6 --LLALGDVRVG-----YMGATPLKGTGTHRFVFLLYKQR-DYTKFDF---PKLPKHSVK

AgSCPYI-6 --HVQNGDTLFE-----YIPVFPRSGVGFHRYIFLVFRQQ-SWNDYSQA--PRASSKNRT

TcSCPYI-6 --DMSTGEVIAE-----YRGAGPPRGTGLHRYLFMVFEHE-QAVTFDEV--RMPKEGSRR

TcSCPYI-5 --EVKTGEVIAE-----YMGSGAPQGTGLHRYIFLVFEQK-GKMQFKE---PKSGKLDKE

TcSCPYI-4 --DVSTGDVIAE-----YFGSGPPKDTGLHRYIFLVYEQK-ERLTFDE---PRSLKLSRA

NvSCPYI-6 --DISQGQTIAE-----YTPTAPPIDGGMHRVVFLVYKQP-EKLTFDE---PYAGNRSLD

IsSCPYI-5 --NVPQGEVLSE-----YVGSGPPKGTGLHRYVFVVYKQP-GRLTCDE---KRLTNRSGD

NvSCPYI-7 --KVAKGEVLTE-----YVAPAPSKTTGLHRFVFLLYKQNQGSITFDE---RRIGNRD-K

AmSCPYI-2 --NIAKGEILAE-----YVGPAPPKNSGKHRYVFLVYKQNQGSITFDE---RRLSNRDGP

CfSCPYI-2 --NIAKGEILAE-----YVGPAPPKNTGKHRYVFLIYKQNQGAITFDE---RRLSTWDGS

DmSCPYI-2 NENPSGGHSLAD-----YVGSGPPKDTGLHRYIFLLYRQE-NKIEET----PTISNTTRT

NvSCPYI-5 --DFSKGETLAE-----YMGAGPPQGTGLHRYIITLYRQP-SKLTFDE---KPMNNLSIE

DmSCPYI-3 --KVSEGQTIAE-----YIGAGPREGTGLHRYVFLVFKQN-DKITTE----KFVSKTSRT

DpSCPYI-3 --DFMKGEALAV-----YLGSQPPPLSGFHRYTFLVYKQP-NYLTCDE---NRLLEQNIK

NvSCPYI-4 --DLSKGDVIVE-----YVGSAPGKDTDLHRYVLLAYKQP-EKLTIEE---AHISNHEHT

AgSCPYI-3 --KVEDGDHMYA-----FVGSGPPNGSGLHRYVFLVYEQPGGLIDFSKA--PRVSNRSRN

AgSCPYI-2 --DVDAGDVLAD-----YVGSGPPQGTGLHRYVFLVYKQP-SRIVFNE---TVLSSRN-P

SmSCPYI-1 --EVSKGDVLSD-----YIGSGPLQGTGLHRYVYLVYKQQ-NKITSDE---PKLENNSLE

DmSCPYI-1 --DLASGEPIAE-----YIGSGPPQGTGLHRYVFLLYKQS-GKLEFDE---ERVSKRSRK

AgSCPYI-1 --DLAKGDTLSD-----YIGAAPPRKTGLHRYVFLLYRQN-ERIYYKE---SRLSNRSTQ

TcSCPYI-3 --DLSKAQVLTE-----YVGAGPPKDTGLHRYVFLLYKQP-GKITFQE---EHKSNTNG-

BmSCPYI-3 --NIGKSEILSE-----YIGSGPPKGTGLHRYIFLIYKQP-EKCDFSKV--PKLPNNSGE

NvSCPYI-3 --DIAKGDTLAA-----YVGSGPPKDTGLHRYVLLAYKQPAGKIDVSEE--KRIPNNSRD

DmSCPYI-4 --QVENGVVLTE-----YVGAGPPQGTGLHRYVFLVFKQP-QKLTCNE---PKIPKTSGD

BmSCPYI-2 --DVSAGETLSG-----YIGSGPPQGTGIHRYVYILYKQP-GKLDFDE---KRLTNTSID

NvSCPYI-2 --DVSQGENLAE-----YRGSGPPEGSGLHRYVFLLYKQP-GKLSFDGE--KRISNRSRD

DpSCPYI-2 --EINKGEVLAE-----YIGSGAPKGTGLHRYVFLVYKQP-GVLSCDE---PRISNRSRE

TuSCPYI-4 --DVGKGKTISE-----YVGSGPPKGTGLHRYVFLVYKQP-GQLSPDE---KFRSNRCGE

RpSCPYI-1 --DVSKGEVLSE-----FISSAPPKGTGLHRYVLLVYKQK-EKLTPDE---PRLKNNSGS

RpSCPYI-2 --DITKGEVLSE-----FISSAPPKGAGLHRYVLLVYKQN-DKLSCNE---PRLKNNSGK

DpSCPYI-1 --DIKNGEVLSQ-----YVGSGPPEGTGLHRYVFLAYKQP-GPLTCDE---PRLTNRSGK

PhSCPYI-1 --DVNKGETLSE-----YVGSGPPKGTGLHRYVYLIYKQN-GKIETSKL--RKLTNKSGD

ApSCPYI-4 --DVSVGETLTA-----YVGSATPPKTGLHRYVFLVYKQP-SKLVFDE---QHISNRTAE

NvSCPYI-1 --DVSKGDVLSD-----YIGSGPPPDTGLHRYVFLVYKQP-SKITFDE---KRLTNRSGD

AmSCPYI-1 --EIAKGDVLSD-----YIGSGPPKDTGLHRYVFLLYKQP-GKLTFDE---RRLTNRSGQ

CfSCPYI-1 --DVSKGDVLSE-----YIGSGPPQGTGLHRYVFLLYKQP-GKLTFNE---KRLTNRSGD

ApSCPYI-2 --NVSLGETLSG-----YVGSGPPPKTGLHRYVFLVFKQP-SKLSFDE---PRISNKSAE

ApSCPYI-3 --NVSLGETLSG-----YIGSGPPPNIGLNRYVFLVYQQP-SKLSFDE---PRLSNRSVE

TcSCPYI-2 --SIEKGEVLSG-----YIGSGPPKGSGLHRYVFVNYKQK-GKISCNE---KRLPSNSGD

DmSCPYI-5 --DVAKGEVLSA-----YVGSGPPPDTGLHRYVFLIYEQR-CKLTFDE---KRLPNNSGD

AgSCPYI-4 --DVAQGETLSA-----YVGSGPPQGTGLHRYVFLVYKQN-GKLTFDE---PRLTNTSAD

TcSCPYI-1 --NVGQGETLSA-----YVGSGPPEGTGLHRYVFLIYKQS-GKINFDE---KRLPNTSGD

ApSCPYI-1 --DIAKGETLSE-----YVGSGPPPETGLHRYVFLAYKQP-SKLNFDE---PRLTNRSAE

BmSCPYI-1 --EVNSGETLSQ-----YVGSGPPEKTGLHRYVFLLYKQP-SKLTFDE---PRLTNTSSD

BmSCPYI-4 --EVCSGETLSQ-----YLGAAPPIATGLHRYVFLLYKQP-SKLTFDE---PRLPNISTV

: . *

TuSCPYI-6 KKRVINLNSLLDK----GLTPVGLSWFQSSWDE-------

BmSCPYI-6 SRNRFSLAKWLLGKN--LCGPVAGTQFRLQF---------

SmSCPYI-2 KARTFLTNDFYK----------------------------

IsSCPYI-6 EQRTFKTHNFYEEHEK-VLTPAGLAFFQ------------

RpSCPYI-3 EERIFSTYEFYKERQK-SLTPAGLSFFTAD----------

DmSCPYI-9 EKRTFSTLDFYRQHQE-QLTPAGLAFYQTNWDESLTQFYH

AgSCPYI-7 PARTFRTLDFYRQHQD-SITPAGLAFFQS-----------

ApSCPYI-5 ANRTFSTFDFYCSHED-IMTPAGLAFYQ------------

BmSCPYI-5 QDRTFVTREWYKKHQD-NITPIGLSFYQ------------

AmSCPYI-3 KERNWNTLEFYRKYQD-YITPAGLAFFQSDWDPT------

NvSCPYI-8 TDRDWKTYDFIRKHQD-YMTPAGLAFFQCDY---------

CfSCPYI-3 KERDWKTLEFYRKHQD-QMTPAGLAFFQSDWDSS------

PhSCPYI-3 SDRNFNTLNFYKERQD-ELTPAGLSFFQSD----------

TcSCPYI-7 EDRTFSTLDFYRERQD-DLTPGGLAFFQADWDRS------

TuSCPYI-1 DRNKFNVAEWVKQNT--LFGPVAGNYFLEGN---------

TuSCPYI-3 VGSKFNVSEWASQNK--VFGPVAGNYFLGK----------

TuSCPYI-2 ERNNFNVAEWAERNR--VFGPIAGNYFLE-----------

IsSCPYI-2 QRPGFDLAKFRRRAN--LHLPFAGNYFFA-----------

IsSCPYI-4 ARGKFNLAKFLSSLG--LADALAANFFYS-----------

IsSCPYI-1 DRKSFNFEMFLQNNS--LPQPLAANFFFSE----------

IsSCPYI-3 DRRSFDFERFLKNNS--LPQPLAANFFFS-----------

TuSCPYI-5 NRYNFNLTSWVQDNN--LHGPLFGNFFKAQN---------

PhSCPYI-2 -RSKFHLNEWLAKHTKLCGAHARTQVHTEKFFT-------

DpSCPYI-4 SRARFSARKFAQLHR--LGQPTAINYFTTEF---------

DmSCPYI-8 GRGTFNCHNFARKYA--LGNPMAANFYLVEWL--------

DmSCPYI-7 GHSNFDVMKFTQKYE--MGSPVAGNIFQSRWD--------

AgSCPYI-5 VRRYFSTRNFTRKYD--LSGVYAGNFFQTQYD--------

DmSCPYI-6 GRSGFETKRFAKKYR--FGHPVAGNFFTSQWS--------

AgSCPYI-6 PRIRFCTRDFARHYS--LGSPVAGNFFIAQYD--------

TcSCPYI-6 HRLRFSTENFRKKYN--FERIFAWNF--------------

TcSCPYI-5 HRISWSMRKFRRENE--LGEAYAGNYFVA-----------

TcSCPYI-4 HRLKWSLKEFVKKYN--LGAAVAGDYFKAKWE--------

NvSCPYI-6 GRFYFSQRKFSAKYN--MGAPIAGNVFFS-----------

IsSCPYI-5 HRGEFKIREFAKKYQ--LGEPVAANFYQA-----------

NvSCPYI-7 RRNRFSTKKFAEKYN--LEGPIAGNYMKAK----------

AmSCPYI-2 QRKRFNVKKFAEKYN--LEGPLAGNFMRVEY---------

CfSCPYI-2 QRKRFSIKKFAEKYN--LEGPIAGNFMLAEYD--------

DmSCPYI-2 GRLNFNARDFAAKHG--LGEPIAANYYQAQYDDY------

NvSCPYI-5 GRVNFNLRKFIEKYK--LDEHVAGNMFKAQY---------

DmSCPYI-3 GRINVKARDYIQKYS--FGGPVAGNFFQAQYDDY------

DpSCPYI-3 GRGKFSIRKFAAKYN--LGQPVAGNVFLSKA---------

NvSCPYI-4 GRPAFSIKNFADKYK--MGDPLAGNMYRA-----------

AgSCPYI-3 HRVNYRHREFVKQYG--LGELVAGNFYQAQY---------

AgSCPYI-2 NRGKWNPAEFVKEYE--LGVPVAGNFYQAQYDDY------

SmSCPYI-1 KRSNFKIKNFAKKYN--LGEPIAGNFYQ------------

DmSCPYI-1 DRPKFSAAKFAINHE--LGNPIAGTFYQAQYD--------

AgSCPYI-1 GRGKFSTHKFSEKYE--LGLPVAGNFFQAQF---------

TcSCPYI-3 NRAKFSTENFAKKYG--LGNPVAGNFYQAK----------

BmSCPYI-3 KRGKFSISQFAQQYK--LGIPVAGNFFVAQ----------

NvSCPYI-3 GRPKFSIQKFADKYK--LGAPIAGNMYQAEY---------

DmSCPYI-4 KRANFSTSKFMSKYK--LGDPIAGNFFQAQWD--------

BmSCPYI-2 GRASFSTKKFAEKYN--LGAPVAGNFYRA-----------

NvSCPYI-2 GRLKFSIRKFADKYG--LGEPIAGNMYQAQ----------

DpSCPYI-2 GRINFSIRKFAVKYN--LGQPIAGNLFQAQY---------

TuSCPYI-4 GRECFKIREFAKKYK--LGVPIAGNFYVAQWDDYVP----

RpSCPYI-1 NRAKFSVSKFAKKYN--LGEPVFGNFYQAEWDD-------

RpSCPYI-2 NRGKFSISKFAKKYK--IGDPMFGNFYQAEWD--------

DpSCPYI-1 HRGKFSIRKFAEKYN--LGQPIAGNVYQAKWD--------

PhSCPYI-1 HRGKFSIQKFSEEHN--LGNPIAGNFYQAQWDD-------

ApSCPYI-4 NRFKFSIHNFSKKYK--LGTPVAGNFYLAQY---------

NvSCPYI-1 GRNNFSIKKFAQKYN--LGNPIAGSMYQAAF---------

AmSCPYI-1 NRGNFSIRKFATKYK--LGDPIAANMYQAEFDD-------

CfSCPYI-1 NRGKFSIKNFAAKYK--LGDPIAGNMYQAEFDDY------

ApSCPYI-2 HRDKFSINKFALKYN--LGTPVAGNFYQAQY---------

ApSCPYI-3 HRNKFSVNEFALKYN--LGTPVAGNFYLAQ----------

TcSCPYI-2 GRGKFSIKKFAEKYQ--LGEPLAGNFFQAEWD--------

DmSCPYI-5 GRGGFKIAEFAKKYA--LGNPIAGNLYQAE----------

AgSCPYI-4 NRGGFAIRKFAEKYQ--LGNPVAGNFYQAEWDD-------

TcSCPYI-1 NRGCFSIRKFAEKYK--LGQPVAGNFYQAQWD--------

ApSCPYI-1 KREKFSIAKFALKYN--LGNPVAGNFYQAQY---------

BmSCPYI-1 KRANFKIAEFAKKYN--LGDPIAGNFYEAQ----------

BmSCPYI-4 KRINFKIAEFAMKYN--LGVPIAGNFFVAQ----------

1. **I63.**

DmLec-31 --NIKHSWDKSAELCRRYG--AELVAIDSYAENNETLAIARA-----SDPNQR-------

DpLec-22 ---KKATWYKAEAFCRQFR--MTLADVPT-DKVEWIKHKVNN-----PSAVDG-------

SmLec-12b -------YAFSLKECQSDG--GRLAQVSSIKQLEEIQDLLGK-----DAHYSE-------

SmLec-4a -----MTWEGARNQCLSLG--GDLVTFKSRQDETRVLNLVTN-----SNDDSS-------

ApLec-2b ---PEVSWHTARSICNGIK--AELTSVHNAEEEQFVESFIRE-----STDSRS-------

SmLec-9 ---PEVTWYTARKICREMQ--GQLSSVGSQAEQNFIIDRIKK-----METYTS-------

SmLec-8 -DNKKKSWYDVRDKCRSMG--KSLLSLDSNDKNDFFNRIFHT-----RNMSNC-------

SmLec-20 -----VTWKEADQRCNSNS--QKLVCIENNEEFDFIKTQVKK------------------

SmLec-15b ----SATWYEAFDVCSQHN--KKLLTLDNKKLSDHFKKEMAK-----RSTDLV-------

SmLec-15a ----GIPFFNAYRICASNN--QHLATIDNEELHIQMNQQIES-----RDVGTN-------

SmLec-18 -------HIAAYRICNDNN--QQLANAEIEDLATALINETNN------------------

SmLec-16 ---QGVNFFEAYRFCSDNK--QNLANIDNEKLSTDMMNEVKN------------------

SmLec-5a ---YLTSWNNAQDFCNYME--SDLVIISNEKIQKDINMYRYR-----QLNSMN-------

DpLec-29 --NVRRNWQDANASCKEIQ--AKLAEPKSTDELRKLTDYLRY-----NRTDQI-------

ApLec-6 ---DRVTWFEADAVCQFHH--AQLATVESNSQFEAIRSYLKE-----LDVIEN-------

DmLec-10 ----EVNWLEANHVCNRVG--AVLATVRNEEQHQLMLHYVNR-----KERIFG-------

DmLec-26 ---QEANWHVADRSCRKLG--AELMVLDNQEDKLLTTTFLKSMGLSFTQSWHH-------

SmLec-12a -----LNYTDSIKYCDQMG--GEIVSIGNDAEQDAIRNLLVD-----EKAFDS---TCIH

DmLec-9 ---AKTNWFEASNHCRQNG--GFLLNLESREELELLSPHLHP------------------

DmLec-30 ----MQTWFEAYVTCRKMN--GHLANIQDEKELDGILALAPN------------------

DmLec-29 --QKKMPWDSAYDTCRQMG--GHLANILDEKELNEIFSEETK------------------

DmLec-27 --ENKQNWFGASNTCRQLG--GHIATIRDEQEFNEIFSRAPA------------------

DmLec-13 --EEKLNWHDALDKCHKMG--GHLASLQSQEELDRFNNQLNG------------------

DmLec-21 ---VELNWLDAQAKCRRMG--GHLASIKTKQEFDAIVEKLDD------------------

DmLec-24 -HNLQVNWRTAEQRCIEMG--GHLAAFQNAEEYNAIVGQLNK------------------

DmLec-25 -DIVQQNWTSALSACQKMG--GNLASIINEADFNAIVSQLSK------------------

DmLec-17 ---VRQNWFDAADKCRRMG--GHLATPQDEDELYLIRKQLEA------------------

DmLec-18 ---VKQNWFDAMTKCREMG--GHLASPQNEEELHLISQKLDT------------------

DmLec-6 --KDAYDWQSAVDFCRDMG--GYIAAIKDQEELDAISARLDD------------------

DmLec-11 --KHKVDWFKATSMCHKMG--AHLLTIQSEDELDAIRTELKD-----INDGSH-------

DmLec-12 -DETRRNWTSAGSACRQMG--TQLATIRSAEELAALRAKLNK------------------

DmLec-8 --VSEKNWSTASKTCRNMG--GHLADIKDEADLAAIKANLKE----------D-------

SmLec-19 --------------CRYNS-----------------------------------------

SmLec-11a ----SGNFDDSELACEMGN--GRLAMVKS-PEIHLFLHKLRI-----RRFLLN-------

SmLec-11b ----IATYDQAAQQCEFKG--GYLANIKTPSTYEFIKGLVYN-----NRKWLEMDEKKMA

SmLec-10 ---LALSWTAADAKCKELT-KGGLVADVDITVQRVLSNILND-----ASIKQP-------

DmLec-16 --IKKINWFGAQNNCLRKG--LNLADVSTMEDFKAVVHYVTS-----QVGFDD-------

DmLec-19 ---KKMNWFGALNNCLRKG--LTLADLSNQRDFDGAIGFLSG-----LGNTED-------

DmLec-3 ---AKVNWFQAQATCAAYG--YTLVSITSEQDQRSLRNFLFNYARNQQDLLTD-------

DmLec-32 --RQPLNFLDALSFCRSRG--GTLISESNPALQGFISWELWR-----RHRSDV-------

SmLec-2 --NQPANFMGASRTCESRG--GSLVDETSPTLQGFLSWELWQ-----RHRNEP-------

TuLec-2 -NDKPMNFENARRFCDLRG--GSLVDETSPALQGFLSWELYR-----RHRSDQ-------

DpLec-1 --NEGNTFAKARDTCIKQR--GDLWNNVARSTLELITRELER-----KKHAMK-------

SmLec-17 ---KGGNFNTARQYCKNRG--GDLTHHITNVTQNFLATEMER-----LRSRMS-------

TuLec-1 ----GGSFNDAENYCKARG--GLVVNSVGDVTQNFLQYELQR-----LKAKLK-------

SmLec-12c ----LVNNAEARAICRNNF--GSLASLVSPFERNFIVDFLMN------------------

SmLec-5b ---GENNWQTAENWCETEN--GNLTTISSSLENIFVYSFVRQ-----KVG----------

TuLec-3 ----SGSYDENLKYCNGLN--ASMITIESSEENDEIFKQFAS-----LNIFR--------

TuLec-4 ---------DNLKYCNGLN--ASMITIESSEENDEIFKQFAS-----LHIFR--------

DmLec-22 --GSWKNFYESDRHCRSLN--AGLLSISNPTEFNVINEWLPI-----IAPYQP-------

DmLec-28 ------NF--SDRHCQSLN--AGLLSFSNKMEFTAINEWLTT-----VVPQSP-------

ApLec-5 ----ELNYFLAYEYCRSIG--LQLASFETLEKTNSISDFLRN-----AGYNKF-------

DmLec-7 ---PELNYFLAYQYCRSLG--LQLASFETKEKAESMTTYLKN-----AGYGNY-------

DpLec-10 -----ECWSNANYMCTQGG--MSLVAIETGEEDSVISRHLMN-----IPELKG-------

ApLec-2a IVNELLNWEDAEEYCSQNGGGGHLASIDSHRTQMLIDTILIN-----SPSYSD-------

SmLec-4b ---EYANWTTASFSCRSKGDDYDLVSIHSPLEHEFLLNELKS-----LGMERA-------

SmLec-7 --------IEASRNCTAMH--SFLLSIQTEREGVIVFNQAVK-----ENVKKE-------

DpLec-18 ---TQKPWVDAFDFCKANG--RSLISFKTAEKQAEFETYLPA-----IIAKSD-------

DpLec-23 --STQKPWIDAYNYCAANG--RKLISIPNVARQLEISTYLPS-----VIGKDK-------

DpLec-24 -VPQQLPWLKAYEFCNSYR--KNLISIQSSTDQQVVTQFLMP-----LILNDP-------

DpLec-13 ---AQQSWAQANDFCKANS--MKLLSIETQNQQKDVSDILMP------------------

DpLec-20 -VKTQLSWAKANDYCNTYD--MKLLSIKTQSVQNDVSDILMP-----LVLGYP-------

DpLec-3 ----KMIREDARQHCRDNN--MHLLSIETQEENEFVINLVKK-----NDDLIP-------

ApLec-3 -KTSKVDWLEARNICREYC--MDLVSIETQEENNLIFRLIQQ-----NDAPYI-------

ApLec-4 ----EVDWLDARNICRRHC--MDAVSLETPQENEFVKQRISR-----GN---I-------

DmLec-23 --SLEVDWLDARNICRRHC--MDAVSLETPQENDFVKQRIAR-----GNVRYI-------

SmLec-13 ------TWLDAVRFCGDLG--ADSISLETQAETEAIYGEIKR-----IHGVGG-------

SmLec-3 --ENPVTWIDALTHCGKLN--SQLLSIETVEESFAINLHIYR-----KHGHNK-------

SmLec-1a ---NLVTWLDAYTHCSRLN--SQLLSIETIEESFAINLHMSR-----KHGRDV-------

SmLec-1b ----AVIWVDAFKHCATLD--SQLVSIETVEESFAINLHLYY-----KHGQTQ-------

SmLec-6 ----NGTFLQGIAYCRNLM--SDLVSIETKIESHCINYHLTG-----TNDLGL-------

SmLec-14 ----TGTYLEGYEYCKARD--SELISIETKVESDIINRFIVD-----SHNPSA-------

DpLec-15 -----GNWEYAYERCLEQN--MTLSTIETQQEDKMIDDFFQL-----NHEFND-------

DpLec-16 -----GNWEYAYERCLEQN--MTLSTIETQQEDKMIDDFFQL-----NHEFND-------

DpLec-17 ---KVDSWYIGDSYCRSNG--ATLLSLETQTEISLIDNYIAS-----ASSGLD-------

DpLec-2 --NKSYSWYEANQFCKEGN--MALLSLENENEDRLVYKHIKS-----ILALNG-------

DpLec-7 --QLKRDWAGANSFCRGGS--MALLKIESSQENDLIYNHYLA-----TPGITQ-------

DpLec-11 --PTALDWAGADSFCRGGN--MALIKIESSQENDLIYNHYLA-----TPGITK-------

DpLec-14 ------NWTAAQEFCRKND--MFLLSLETQNETELINNHIKN-----SGLPKD-------

DpLec-4 ---VGKNWISADTFCKDEG--MGLLSLETVEEDKLIYDHIKI-----TPELNS-------

DpLec-8 ----LPTWMRCDEYCKGGN--MTLLSLETEAEDMLINSHVQA-----NPELNF-------

DpLec-9 ---SQLDWTSADQICRDGS--MTLLTIETLEEDQSIFDYVIS-----SSKLVP-------

DpLec-5 ---NRVTWLDADKFCRIGG--MTLLSLESKEEDQMINNHIKS-----TSEFSD-------

DpLec-6 ----RLEWVSADTFCRDEN--MRLLSIETYEEDQLIYNQVKS-----ISQLHS-------

DmLec-14 --QTKVNWYVAYENCRRLQ--SELVTFETAEEFDAIAAFLNA-----RGDRSE-------

DmLec-15 -GTESLNWYEAYEKCRELN--SELVTFETDQEFDAVTAFLTA-----NGSRLT-------

DmLec-20 ---ESLNWYEAYEKCRELN--SELVTFETDQEFDAVTVSNGS------------------

DmLec-4 ---LQKNWFGAYEICRQQQ--AELISLETFDELRLVSEYLLA-----NNIFER-------

DmLec-5 --KLDRNWYDAFEACRQMN--ADLVAFEDRKEQKLIYHYLVD-----NEMDTT-------

DmLec-2 -PMNKVNWFQAAGACRMMN--AHLASIEDKPEMEALIKYMKA-----KGFKNN-------

DpLec-12 ---FLMAWNDAKYYCRLNN--YTLISLETKAEDDLIHQHIET-----TELLSE-------

DpLec-19 ---FKANWYKSAKFCNYHG--MQLASIESQIENDQLEKHIKE-----FGFGNE-------

ApLec-1 -IFFKANWYKAAQYCRYHG--MHLASISNQEENDKLEKYIKD-----NGLGHE-------

DmLec-1 --FFKANWFKATQYCRYHG--MHLASISSQEENDRLEKHIRD-----FGLGHE-------

DpLec-28 ----------HTTYCSDNN--MKLVSIEDNNEENTIYLAWVV------------------

DpLec-26 ---ANKNHADHSMLCIDNN--MTLVSFENRTEEEFVQRTWST------------------

DpLec-27 -VFNTKSFPDMTMYCLDYK--MKLIIFENRTEEEIVQKTWGT------------------

DpLec-25 -----RNQADQTMYCVDNK--MKLISLENATEEERVQAAWGT-----LSP----------

DpLec-21 -----------TMFCVDHN--MKLVSFENRTEEELVQAAWGT------------------

*

DmLec-31 --------ASDKYWLGL---ASLDD----------LRTN----TLE----------SASG

DpLec-22 --------KGEWYWTSA---TDRFA----------RGQ-----MIW----------MNTG

SmLec-12b -------KCNSGFYVDL---TSEKR------------------YYK----------TPAN

SmLec-4a ---------GDDFWLGL---RLLND--------SGINE-----WRW-----------NDQ

ApLec-2b ----------AIYWLGG---AWN------------DKS-----WYW----------VDNS

SmLec-9 ---------GTLFWLGG---FRKLN----------HDRG----WQWVDSSPFNYSAYKLE

SmLec-8 -LGRIRNLRSNNTWQH----RYIY-------------------WRW----------AATE

SmLec-20 --------TGINYWIGL---TATED----------TTTKLIE-WSW----------IPKR

SmLec-15b -------------WIGLHSYPEIID----------IETYLY--WQW----------NGTG

SmLec-15a ------------YWIGL---KSFKG----------NYGLPISNWHW----------NGTG

SmLec-18 --------RKASYWIGL---KNFDI----------YGIPIKK-WHW----------NSTR

SmLec-16 --------RKINYWIGL---KSFKQ----------YGIQVNK-WHW----------NSTS

SmLec-5a ---------VKDFWIGL---NDFKS----------PGD-----FEW----------VDET

DpLec-29 ---------GGGYWTGG---LN-------------PGLM----WLW----------PSLG

ApLec-6 ------------VWIGL---KRNSE----------ASE-----FTW-----------TNY

DmLec-10 ---------NRTFWLGA---TNLVD----------RSYF----WTW----------MSTG

DmLec-26 -----------SVWAGI---NCLGN----------RRT-----FLL----------ARNG

SmLec-12a KAYLGIHEDISKQWRSG------------------DGE-----FVS-------------G

DmLec-9 ---------AYSYWLSI---NDLGE----------RGV-----YVS----------EATG

DmLec-30 ----------NSYWIDI---SKLVE----------NGGT----FVS----------TLTG

DmLec-29 ----------KKYWVDI---NSRAN----------DGAS----WIS----------TLSG

DmLec-27 ----------GVFWIDM---NAMFK----------NGL-----FAS----------SLTG

DmLec-13 ---------LNRYWIDV---TNQFN----------ESE-----FVS----------VTKG

DmLec-21 ---------SKSYFLGV---NENTK----------TGE-----FVS----------AASG

DmLec-24 ----------ANYWLGV---NDLAK----------QGE-----FIS----------LASG

DmLec-25 ---------DNTYMIGI---SDLAE----------KGV-----FIS----------VSSG

DmLec-17 ----------RWFWLDI---SNLVD----------KDQ-----YIS----------LATG

DmLec-18 ----------ESYWLDL---SDLTD----------HGQ-----YIS----------LVSG

DmLec-6 ----------KSYWLGI---NDLQS----------SNT-----YVS----------VASG

DmLec-11 -----------DFWLDI---NDIAK----------WGE-----FIS----------LATG

DmLec-12 ---------ERHYWLDI---TDLEK----------EGD-----FRI----------SASG

DmLec-8 ----------THYWLGI---NDLDH----------EGK-----FLS----------MPTG

SmLec-19 ------------YWAGI---RQVE-----------DSK-----WVF----------MDYS

SmLec-11a ------------YWIGL---NDKVA----------EGV-----WVY----------SDGS

SmLec-11b DILRVNNNRIPAIWIGV---RYY------------KGK-----WVF-----------SDD

SmLec-10 --------IQSHFWIGG---KTTVT----------KGNKNVH-WIW-------------K

DmLec-16 ------------FWFGG---NDLQS----------EGR-----FKY----------ISSG

DmLec-19 ------------FWFGG---NDLYH----------EGR-----FQY----------ISNG

DmLec-3 -----------PLWTSG---TDLAS----------DNN-----WVW----------FSKG

DmLec-32 ---------SSQYWMGA---VRDGS----------DRSS----WKW-----------VNG

SmLec-2 ---------SGQYWMGA---TRDSN----------NLNN----WKW-----------ISG

TuLec-2 ---------YGQYWLGAV--RDTAS----------PNN-----WKW-----------ING

DpLec-1 ---------SPMVWVGA---QKEAS--------FTSRT-----WRW-----------VTG

SmLec-17 ---------QVLLWLGM---TKVPG----------SKVRT---WRW-----------VTG

TuLec-1 ---------SRLVWLGA---KREIPANQPVTHRSRSNV-----WRW----------VNGG

SmLec-12c --------------------RSNVT----------ESTST---WFV---------ESTGG

SmLec-5b -----------SYWIGL---YVD------------DSG-----LKW----------EAEN

TuLec-3 ------------FWIAG---KTEG-------------------ILL---NEIHQTKIDLM

TuLec-4 ------------FWISG---RTEG-------------------FKL---NKKHQTKVDLM

DmLec-22 -----------EFWTSG---NKLGG----------TSD-----YYW----------QSTG

DmLec-28 -----------ELWTSG---NKLGG----------SED-----YYW----------QSTG

ApLec-5 -----------DYWTSG---NRLG-----------TDM-----LIW----------MSTG

DmLec-7 -----------DFWTSG---NRLG-----------TGM-----FLW----------MSTG

DpLec-10 ----------YAYWTAG---RYN------------DTE-----WQW----------FSNK

ApLec-2a ---------NAPYWIGA---TDMNN----------EGF-----FKW-----------TDS

SmLec-4b -------------WIGL---NDKAI----------EGQ-----FVW-----------SDG

SmLec-7 ---------PFGYWTSG---TRVTS----------KRGFE---FVW----------SSTW

DpLec-18 -NLFTT----IGFWTSG---TFCLETLAANVYCPSKNT-----WAW----------AATR

DpLec-23 VDIFSY----IGFWTSG---IYAL------------TA-----YTW----------ASIP

DpLec-24 ------VAKNIGVWTSG---ASVPG----------LKT-----YFW----------VSKL

DpLec-13 ---------LIGIWTSG---SYNSG----------QKS-----FTW----------LSTQ

DpLec-20 ------ISKQIGVWTSG---AYNTG----------QKG-----FAW-------------L

DpLec-3 ----------NQFWTLG---YNANL----------DPNN----WGW------QQPNLNVS

ApLec-3 -------------WTSGRLCDFKGCENRPDLEPKTVNG-----WFWSATRGKISATNQTS

ApLec-4 ----------RYIWTSGRKCNFNGCD-RPDLVPANVNG-----WFW----------SGSG

DmLec-23 -------------WTSGRKCNFAGCDRPDLQP-PNENG-----WFW----------SGSG

SmLec-13 --------EGAAYWTSG---NNFEQ----------TGGE----FNW----------WHGL

SmLec-3 ----------VAYWTSG---SDSYQ----------QHR-----YIW----------MNIG

SmLec-1a ----------TYYWTSG---SDAYQ----------QGR-----YIW----------MSNG

SmLec-1b ----------VSYWTSG---SDAYQ----------EDD-----YIW----------MSKG

SmLec-6 ------------YWTSGVADFNRGN----------TRS-----FHW----------LSTG

SmLec-14 ---------PGNFWTSG---FDFNR----------NRQ-----FYW----------MGTG

DpLec-15 ----------RILWTSG---KYSEL----------GSQ-----WGW---NHYGNETVAPG

DpLec-16 ----------RILWTSG---KYSEL----------GSQ-----WGW---NHYGNETVAPG

DpLec-17 ---------KYAYWTSG---QW-------------GGTG----YVW----------TGSN

DpLec-2 ----------ELYWTSG---QHSLR------------------WEW-----------ADG

DpLec-7 ----------DIYWTSG---RYSRD----------GNKE----WEW--------ATAPPY

DpLec-11 ----------DIYWTSG---RYSRD----------GNKE----WEW--------ATTPPY

DpLec-14 -----------FYWTSG---SDEAN----------EGQ-----WIW----------TSTQ

DpLec-4 ----------VSYWTSG---MYSLD----------GDKI----WEW--------ASTEPF

DpLec-8 ----------SEYWTSG---RYSQE----------GNNR----WEW--------ASTQPF

DpLec-9 ---------NGLYWTAG---KYSQD----------NDE-----WEW--------ASNEPF

DpLec-5 ----------DYYWISG---KFS------------NNR-----WEW-----------ANN

DpLec-6 ---------TDYYWTSG---KYS------------NNR-----WEW--------ASTEPF

DmLec-14 ------------HWTSG---NDLGK----------TGT-----HYW----------FSNA

DmLec-15 ------------YWTSG---NDLAK----------TGS-----HRW----------FTNA

DmLec-20 ---------RLTYWTSG---NDLAK----------TGS-----HRW----------FTNG

DmLec-4 ------------YWTSG---TDLGT----------KGK-----HVW----------FSNG

DmLec-5 ------------YWTAG---TDLAE----------QDS-----FVW----------FSNG

DmLec-2 ----------DYFWISG---NDLGT----------EGA-----FYW----------MSNG

DpLec-12 ---------LPWFWTSG---TYS------------EDQ-----WKW-------STSPLPG

DpLec-19 -----------HFWTAG---TDQGE----------EGS-----FFW----------MSTG

ApLec-1 -----------HFWTSG---TDQAE----------EGS-----FFW----------LANG

DmLec-1 -----------HFWISG---TDLAD----------EGN-----FFW----------MATG

DpLec-28 -----------NFWTSG---TYNKT----------TGI-----WTW----------SSTM

DpLec-26 ---------QIPFWTSL---TDTRR----------DGT-----WVW----------EGTM

DpLec-27 ---------QNPYWTSL---TDSRR----------DGT-----WVW----------ESTM

DpLec-25 ------------FWTSL---TDTRR----------EGT-----WLW----------ESSM

DpLec-21 ---------EVSYWTSL---TDTRR----------DGT-----WLW---------ESSMT

DmLec-31 ALISQ--YSGY--WS---L--H------QPN---------------------------AE

DpLec-22 QL-----YQN--------P---------NSN----------------------------S

SmLec-12b ETVN---YTK---FV-------------DDS----------------------RRS---M

SmLec-4a SRLS---YTN---WNILLV--H------ENV----------------------TAN---V

ApLec-2b TET----FSA---WLATDAANA------LPY----------------------------K

SmLec-9 ARLLFDWYPD---WM---KEKN------EPI-------------------PGLKHQNKFA

SmLec-8 RDIS---YKY---WC---P---------HSE----------------------------V

SmLec-20 HRNY---FSN---WC-------------APD------------------------GLQLG

SmLec-15b QDLD---YKD---WC---V--N------EPL---------------------------IA

SmLec-15a KSLK---FKA---WC-------------PPE---------------------TATREHHK

SmLec-18 TAVT---YQN---WC-------------APQ--------------------THKLNPQYK

SmLec-16 NEVT---YKN---WC---P--P------QTN----------------------KDNEYYK

SmLec-5a KPM----YSN---WL---N--D------TVN----------------------------A

DpLec-29 TSLT---NIDPSMWF------N------TPD--------------------ISTEN---N

ApLec-6 QPLA---RSG---YF---R---------EEV----------------------PRS---S

DmLec-10 IPVT---YAQ---WSRREP---------KSD----------------------------R

DmLec-26 ETVP---YLN---WV---P--L------EPK-----------------------------

SmLec-12a ISEM---FFM---VD---E--G------S-------------------------------

DmLec-9 LEAP---FLN---WS---A--G------EPD----------------------------N

DmLec-30 REPF---FVK---WK---S---------NQD----------------------------T

DmLec-29 RDVP---FLK---WK---P---------DLA----------------------------T

DmLec-27 RSPP---FFK---WK---K--E------ERG----------------------------N

DmLec-13 SKAN---FLS---WA---D--G------EPT----------------------------K

DmLec-21 KSGL---YHE---WG---P--G------EPH---------------------------HN

DmLec-24 KRAT---YFK---WR---K--N------EPK----------------------YNN---P

DmLec-25 KRAP---FLK---WN---P--G------EPL----------------------YEH---V

DmLec-17 KEVS---YLK---WR---H--G------EPK----------------------------K

DmLec-18 SKAP---FLK---WN---K--G------QPN---------------------------RE

DmLec-6 REVE---FLN---WN---A--G------EPN---------------------------HG

DmLec-11 MNPP---FLK---WH---K--H------RPQ----------------------------V

DmLec-12 KRPN---FLK---WR---A--G------QPN----------------------NFS---G

DmLec-8 KQTT---FLK---WA---S--G------RPS----------------------------Q

SmLec-19 VLGN---FSY---WA---P--K------EPD----------------------------G

SmLec-11a RVQG---FKR---WA---P--G------EPN----------------------------N

SmLec-11b TVTN---FTI---WA---A--G------EPN----------------------NAK---T

SmLec-10 NGAE---LEK---FY------GKL----QPD----------------------MSA---T

DmLec-16 KLVR---YMG---DS------NIV----EPT----------------------QRS---N

DmLec-19 RLVR---YYS---NY------SNV----LPL----------------------EHS---E

DmLec-3 RAVN---YRN---FQ---N--G------LPG---------------------------YS

DmLec-32 DELT---VSF---WS-------------HPG----------------------------G

SmLec-2 NDVS---VSF---WN-------------LPG----------------------------G

TuLec-2 KDVS---ISF---WS-------------QPR----------------------------Q

DpLec-1 NVVE---RPP---WG---R--E------QPN----------------------NYN---G

SmLec-17 QQVV---SPL---WG---D--D------QPN----------------------NYN---G

TuLec-1 LITQ---FL----WA---D--D------QPN----------------------NYN---G

SmLec-12c LPSG---SDD---DI------G------YPT----------------------NLGG--S

SmLec-5b QDT----FFN---WD------G------QPE---------------------------IS

TuLec-3 KSTY---FSN---WG---Q--G------SPN----------------------CPI---G

TuLec-4 KSTD---FSN---WG---L--N------SPH----------------------CTP---G

DmLec-22 QKAV---YLP---WS---A--G------QPT----------------------------T

DmLec-28 KKAF---YLP---WQ---A--G------QPT----------------------------P

ApLec-5 LPFN---TTF---NQMKRP--N------SPDNNGLDNEDLKMNARPELPIARKKRGESGS

DmLec-7 LPFNAT-FDF---FENSAD--AIQAGLLDPV-----------DHNSNTSPQRTARDSSGA

DpLec-10 KPIT---FDD---LK-------------NTDKVK-----------------DKEA----V

ApLec-2a SPFT---YSN---WY---QG-HIHQPYSGPNSKQ-----------------PNDDGL--S

SmLec-4b SPFD---YND---WAWHEKLIG------KPN-------------------KFSTTTKGFG

SmLec-7 EPLT---YTA---WC---P--S------EPN----------------------NWM---N

DpLec-18 ENFG---YVN---WE---T--G------QPD------------------------VTMLP

DpLec-23 QYFT---YTN---WI---V--G------NPD----------------------ITNL--P

DpLec-24 SLFF---YSN---WF---Y--G------EPR----------------------------P

DpLec-13 KPFT---YVN---WF---Y--G------EPN----------------------------T

DpLec-20 EPFT---FVN---WF---Y--G------EPN----------------------------T

DpLec-3 KPLT---YKN---WC---R--GS-----EPD----------------------------N

ApLec-3 AGWT---YIP---WS---K--SGHKKTPQPD----------------------NAEFDIN

ApLec-4 AKIGPTTQRNSGDWS---H--TGGFGQAQPD---------------------NREAPQGN

DmLec-23 AKIGPTSQRNTGDWS---S--TGGYQQPQPD---------------------NREAAQGN

SmLec-13 KPFN---YTN---WC---H--L------QPD----------------------NMG---L

SmLec-3 IPFS---YTN---WC---H--P------GPD----------------------NYD---G

SmLec-1a KPLS---YTN---WC---A--N------EPN----------------------------D

SmLec-1b KPFT---YSN---WC---A--N------EPN----------------------------N

SmLec-6 EAFT---FTN---WC---P--G------QPD----------------------RWK---E

SmLec-14 TNFS---DTN---WC---S--G------EPN----------------------------G

DpLec-15 TPMG---YTN---WY---P--G------RPK----------------------------N

DpLec-16 TPMG---YTN---WY---P--G------RPK----------------------------N

DpLec-17 QPFT---ATN---WY---P--G------QPD----------------------------D

DpLec-2 ESIN---YTN---WK---TGKS------EPN-------------------------EEIK

DpLec-7 AKFN---YTN---WS---P--T------STS----------------------GP----Q

DpLec-11 AKFN---YTN---WS---P--N------STS----------------------GP----Q

DpLec-14 ENIT---VTN---WR---N--N------QPD----------------------------G

DpLec-4 QPLT---YVN---WS---P--G------QPD----------------------------N

DpLec-8 QPFN---YTN---WH---PIYN------QPD----------------------------D

DpLec-9 EPFS---YEN---WG---V--G------EPS----------------------------N

DpLec-5 EPLT---YTH---WY---T--G------EPS----------------------------Y

DpLec-6 QAMN---YTN---WF---T--G------EPS-----------------------HS---Q

DmLec-14 QLVT---IKR---WA---P--K------QPD----------------------NAG---G

DmLec-15 QRIS---SLR---WA---R--N------QPD----------------------NAG---Q

DmLec-20 QRIS---SLR---WA---R--N------QPD----------------------NAG---Q

DmLec-4 QPLS---TDL---WY---G--G------EPN----------------------NKN---N

DmLec-5 QPVA---SDL---WC---N--N------EPN----------------------NAK---N

DmLec-2 RPMT---YAP---WNGPKQ---------MPD----------------------NYG---G

DpLec-12 EPFS---YSN---WA---S--T------RPI----------------------------Y

DpLec-19 RPVT---FTN---WN---A--G------EPN------------------NFRYENG---E

ApLec-1 RPIG---YTN---WN---A--G------EPN------------------NFKYENG---E

DmLec-1 RPIT---FTN---WN---A--G------EPN------------------------NFRYE

DpLec-28 ANVSPG-YTN---WA---P--F------EPN----------------------NTL---V

DpLec-26 TILDTGDYTH---WF---P--G------RPS------------------------SASDN

DpLec-27 TVLKAGNYTN---WF---P--G------RPS---------------------YVAN---N

DpLec-25 TVPE---YTN---WY---P--G------RPN------------------------SVASN

DpLec-21 VPAD---YTN---WY---P--K------RPN--------------------------TAV

DmLec-31 S-G--ECV----------------AAAFA-------------GKSQSWDLGTC-------

DpLec-22 D-ATSLCV----------------IVGPITPVSA--------ESTSFLQEIES-------

SmLec-12b D-R--HCA----------------VVHE--------------LQRYMWSDQDC-------

SmLec-4a E-M--FCG----------------IFE---------------IITEQWTIEPC-------

ApLec-2b N----ICL----------------AISWLSSPPVNL------PRGLYWTANDC-------

SmLec-9 Q-E--SCL----------------AFQWKSVKDSRGRLK---YSGLYWESQTC-------

SmLec-8 V-KEAMCG----------------YIA---------------LDPMCWMAERC-------

SmLec-20 AFH--HCA----------------FIR---------------ADEQCWQAWEC-------

SmLec-15b V-S--MCG----------------YMATN-------------GKVQCWKSVKC-------

SmLec-15a Y-E--WCG----------------YLD---------------SEKKCWRTKPC------D

SmLec-18 N-E--WCG----------------YIH---------------KDEQCWKTEAC-------

SmLec-16 N-E--WCG----------------YIN---------------VNEQCWKTDVC-------

SmLec-5a S-G--NCV----------------MLD---------------VETQYWKIQNC-------

DpLec-29 N-G--KCL----------------RLSYDRN-----------LQRYALQGSDC-------

ApLec-6 D-P--VCV----------------VTDP--------------TANFKWHSLHC-------

DmLec-10 T-GQDACL----------------VL----------------GTDNLWHSEPC-------

DmLec-26 ------------------------------------------------------------

SmLec-12a --E--KCA----------------AIQ-R-------------PGYFKLNNVQC-------

DmLec-9 SSGYDRCV----------------ELWLS-------------TTSFQMNDLPC-------

DmLec-30 K-KKNQCV----------------Y-----------------IYAKEMSYDEC-------

DmLec-29 N-IHRHCV----------------Y-----------------INSNEMYFENC-------

DmLec-27 K-F--DCV----------------NV----------------YNKEMYNEN-C-------

DmLec-13 D-G--ECV----------------DIRTF-------------NGKTTMNDNSC-------

DmLec-21 N-DQERCV----------------S-----------------ILRKLMYVGNC-------

DmLec-24 T-Q--HCA----------------YVF---------------GHENIMIVLSC-------

DmLec-25 D-Q--RCV----------------SI----------------HNGGMWVAS-C-------

DmLec-17 S-STANCA----------------Y-----------------LYAGDYYTYQC-------

DmLec-18 N-A--QCV----------------R-----------------VKGGLYQTFQC-------

DmLec-6 N-EDENCV----------------E-----------------LIRSKMNDDPC-------

DmLec-11 Q-IHQRCV----------------H-----------------LRGGEMMDGKC-------

DmLec-12 N-Q--HCV----------------D-----------------LLDGLMYDNKC-------

DmLec-8 L-DTLNCV----------------F-----------------LYNGEMYDYPC-------

SmLec-19 N-ENEDCV----------------LLAA--------------HVQTKWVTTQC-------

SmLec-11a A-KGQHCG----------------KIIM--------------SKNLLWDDDQC-------

SmLec-11b E-E--DCV----------------------------------LMSQQWHDVKC-------

SmLec-10 E-L--ACL----------------SLSR--------------ELDWQWDDKTC-------

DmLec-16 L-D--DCL----------------EIRIR-------------PNVTVVLDVNC-------

DmLec-19 C-D--DCL----------------EVRIR-------------SEINMVSADNC-------

DmLec-3 S-DNRHCL----------------GIN---------------GINGLWVNENC-------

DmLec-32 D-E--DCA----------------RFDG--------------SKGWLWSDTNC-------

SmLec-2 N-E--NCS----------------RFDG--------------TKGWLWSDTNC-------

TuLec-2 N-F--NCS----------------RFDG--------------TRGWLWADTNC-------

DpLec-1 E-Q--NCV----------------VLDG--------------GRDWLWNDVGC-------

SmLec-17 E-Q--NCA----------------VYDG--------------GRNWLWNDVGC-------

TuLec-1 Q-Q--NCI----------------VLDG--------------GRKWQWNDVTC-------

SmLec-12c A-T--QCV----------------SVHV--------------SPRVGWSVIDC-------

SmLec-5b S-G--LCA----------------VM----------------ERNGKWLMQLC-------

TuLec-3 E-E--CCI----------------MV----------------DSQKQWHNYVC-------

TuLec-4 E-Q--CCI----------------MV----------------NSFKQWQDYDC-------

DmLec-22 TAG--DCL----------------TLMANVTMTPEEAI----LSVHRLTVKPC-------

DmLec-28 ITG--DCL----------------TLLANVTMTAEGTT----MSEHRLSVRGC-------

ApLec-5 R-D--GCV----------------AIK---------------APNMDWVTADC-------

DmLec-7 E-K--GCV----------------ILK---------------QPTLKWMPEDC-------

DpLec-10 S-N--RCI----------------LLNYVEED----------YPSGGYASRIC-------

ApLec-2a R-Q--DCV----------------ELRQVYRPQNRLLKYFNRNSSYTWNDRDC-------

SmLec-4b K-ITPDCV----------------KIEMT-------------SIAGKWTSYDC-------

SmLec-7 D-E--YCI----------------HLTNLQS-H---------NTKACWNDRGC----MES

DpLec-18 R-T--ACA----------------RAVP--------------TNKYKWDDIDC-------

DpLec-23 R-A--TCI----------------RAAP--------------AENYQWDDIDC-------

DpLec-24 T-SLSECV----------------RIAP--------------VPEGPWASTPC-------

DpLec-13 G-LSNQCV----------------RIRIVPES----------LTDGKWATLDC-------

DpLec-20 G-ISNPCV----------------RIVPES------------MSDAKWATLPC-------

DpLec-3 GFENEHFI----------------A-----------------VINRCWHDVPG-------

ApLec-3 G-TVESCL----------------SVLNNVY-----------GDGIAWHDIGC-------

ApLec-4 D-E--SCL----------------AVLNNFY-----------QDGVKWHDVAC-------

DmLec-23 D-E--SCL----------------SILNNFY-----------NDGIKWHDVAC-------

SmLec-13 I-E--NCV----------------LIWYWP------------RGYSCWNDRFC-------

SmLec-3 N-E--DCM----------------HLIHGS------------NGNTCWNDAKC-------

SmLec-1a ENGFERCI----------------HLNYLPNGMF--------AICWLWNVTNA-------

SmLec-1b D-GNEDCI----------------HLNYYP------------SGTTCWNDAKC-------

SmLec-6 N-D--FCI----------------L-----------------LITKCWNDVPC-------

SmLec-14 P-TYEYCV----------------HIINNNVLLGTGTK----FSHINWCSGEP-------

DpLec-15 N-KDGRCL----------------GLVFNDYGVT--------YENGHWDEFSC-------

DpLec-16 N-KDGRCL----------------GLVFNDYGVT--------YENGHWDEFSC-------

DpLec-17 M-ATGFCV----------------RLFYGT------------DYAGRWSDIIC-------

DpLec-2 N-E--AYL----------------YLDTSYD-----------FESGFWFASNN---SRLL

DpLec-7 P-D--FCLPNGSDCNPAYAEQFSVDIFFLFD--------------GRWYDVVNT------

DpLec-11 P-D--FCL-NGSDCNPAYAEQFSVDISFFFD--------------GRWYDVVNT------

DpLec-14 G-KKENCL----------------YLHS--------------RDEFKWGDWMC-------

DpLec-4 N-GPGYCL----------------HLDLIKT-----------FSAGYWADIFC-------

DpLec-8 N-EPGSCA----------------LLYFL-------------DYSGYWADNVC-------

DpLec-9 A-TDEYCA----------------YANFSPPQN---------FSAGYWYDDRC-------

DpLec-5 N-QSGSFV----------------YINYL-------------FPNGIWFDQIG-------

DpLec-6 T-G--SFA----------------YINFNF------------NNTGLWFDEIG-PTPTTT

DmLec-14 R-E--HCI----------------HLGYIYGY----------STEFQLNDRPC-------

DmLec-15 K-E--HCI----------------HLGYIYKD----------SRKFELNDRPC-------

DmLec-20 K-E--HCI----------------HLGYIYK-D---------SRKFELNDRPC-------

DmLec-4 E-E--HCD----------------ELGSDFRP----------TKSPGMNDRNC-------

DmLec-5 E-E--HCV----------------EYKPLHP-----------EAKMGLNDRVC-------

DmLec-2 N-E--NCV----------------HMF---------------ATREMINDANC-------

DpLec-12 N-MNGYCT----------------EINYS-------------TFQGAWVDFPC-------

DpLec-19 E-E--HCL----------------ELWNRD------------GKGLKWNDSPC-------

ApLec-1 E-E--HCL----------------ELWNRD------------GKGLKWNDSPC-------

DmLec-1 NGEEENCL----------------ELWNRD------------GKGLKWNDSPC-------

DpLec-28 D-G--NCL----------------------------------TIKNGWYDDPC-------

DpLec-26 E-E--DCM----------------LYGGN-------------TFLAYWGDVNC-------

DpLec-27 A-D--DCM----------------LYGGT-------------IYLNFWGDISC-------

DpLec-25 T-D--DCM----------------AYGGA-------------TYLTFWGDVNC-------

DpLec-21 N-NVDDCM----------------AYGGA-------------TYLNFWGDIAC-------

DmLec-31 ----ESLLP----FM-----------------CRAQA------------

DpLec-22 --LEDEYFA----LIETISKEDDINDILIHGTCPKHFT-----------

SmLec-12b ----SLASC----FL-----------------CRMTG------------

SmLec-4a ----SLTAI----SV-----------------CEADVG-----------

ApLec-2b ----DTVGG----YI-----------------CKKNQTKL---------

SmLec-9 ----EQSGG----YI-----------------CKKNQIAL---------

SmLec-8 --TNKDVNS----FV-----------------CEAV-------------

SmLec-20 ----DTNYGAANGFI-----------------CKNM-------------

SmLec-15b --SNAGITS----FI-----------------CEDL-------------

SmLec-15a RDTEQNIHG----FI-----------------CE---------------

SmLec-18 DRDSRNVNG----FI-----------------CESSI------------

SmLec-16 DRDSKIVSG----FI-----------------C----------------

SmLec-5a ----TSEFG----FI-----------------CSKDWSFNQ--------

DpLec-29 ----QRYLY----FV-----------------CEYDVNSTAP-------

ApLec-6 --GGPEVAS----FV-----------------CELPVPY----------

DmLec-10 ----QRKHN----FI-----------------CENVCQ-----------

DmLec-26 --------------L-----------------CRT--------------

SmLec-12a ---TGTSYC----QI-----------------CEYDN------------

DmLec-9 ----YSSVA----FI-----------------CQLN-------------

DmLec-30 ----FEKKS----FV-----------------CQADQWA----------

DmLec-29 ----ANDNY----FA-----------------CQAEQWA----------

DmLec-27 ----FNTHL----FI-----------------CQAEQWD----------

DmLec-13 ----FANLY----FI-----------------CEKSIE-----------

DmLec-21 ----TYEKS----FI-----------------CQYGI------------

DmLec-24 ---TTDVMH----FI-----------------CQSD-------------

DmLec-25 ----TSDFK----YI-----------------CEAN-------------

DmLec-17 ----SDRNF----FI-----------------CQAV-------------

DmLec-18 ----DHRVL----FI-----------------CQANQ------------

DmLec-6 ----HRKKH----VI-----------------CQTDKEV----------

DmLec-11 ----SEQFL----FI-----------------CQLAVN-----------

DmLec-12 ----ESLSY----FI-----------------CQSDD------------

DmLec-8 ----HYTFR----FI-----------------CQTEE------------

SmLec-19 ----HWGYR----FI-----------------CQLVDAC----------

SmLec-11a ----NNQWL----YI-----------------CQFQG------------

SmLec-11b ----NTIRN----FA-----------------CQYNV------------

SmLec-10 ----ESSFV----FV-----------------CQHNIL-----------

DmLec-16 ----QEKKY----FI-----------------CEQNQM-----------

DmLec-19 ----HERQY----FI-----------------CSERYC-----------

DmLec-3 ----SELRY----FV-----------------CEKRCQ-----------

DmLec-32 ----NTLLN----FI-----------------CQHQPKT----------

SmLec-2 ----NLNLN----FI-----------------CQHRPLT----------

TuLec-2 ----NLPIH----FV-----------------CQHRPL-----------

DpLec-1 ---NLDQIH----WI-----------------CKFPPLTCGHPD-----

SmLec-17 ---NLDYLL----WI-----------------CQYGPP-----------

TuLec-1 ---DLDYLP----WI-----------------CQYTP------------

SmLec-12c ----EAKAG----FI-----------------CHD--------------

SmLec-5b --DGNQIKT----AI-----------------CEKPVS-----------

TuLec-3 ----SNDLY----FA-----------------CEKRRIFESSE------

TuLec-4 ----LEDFN----FA-----------------CEKRRI-----------

DmLec-22 ----TQWAP----HI-----------------CQAPLE-----------

DmLec-28 ----TKWAP----HV-----------------CQAP-------------

ApLec-5 ----TDLKD----FI-----------------CEQTRC-----------

DmLec-7 ----SAVKD----FI-----------------CEQTRCYY---------

DpLec-10 ---ASFGYR----FV-----------------CEATA------------

ApLec-2a ----SVKNR----FL-----------------CQTQ-------------

SmLec-4b ----NVELG----YI-----------------CQKTFR-----------

SmLec-7 TTHSRHPMH----YI-----------------CETEA------------

DpLec-18 ----GQFLP----FI-----------------CE---------------

DpLec-23 ----GQFLP----FI-----------------CE---------------

DpLec-24 ----EQWLP----FV-----------------CQD--------------

DpLec-13 ----NQWLP----SI-----------------CEDN-------------

DpLec-20 ----DQWLP----FI-----------------CEKN-------------

DpLec-3 ----HFDWA----SV-----------------CEREANIFDPNLDEKFF

ApLec-3 ----YHEKP----FV-----------------CEDSD------------

ApLec-4 ----HHLKP----FV-----------------CEDSDELL---------

DmLec-23 ----HHIKP----FV-----------------CEDSD------------

SmLec-13 ----KTNLI----CV-----------------SCNK-------------

SmLec-3 ---WATHVF----DD-----------------NLTK-------------

SmLec-1a --------------------------------CP---------------

SmLec-1b ---WSYNRL----FI-----------------CEKEQ------------

SmLec-6 QGNYQFLCEYNLLFA-----------------N----------------

SmLec-14 ---NGPTYEYCVHII-----------------N----------------

DpLec-15 ---QNGYYG----VF-----------------CEETQQ-----------

DpLec-16 ---QNGYYG----VF-----------------CEETQQ-----------

DpLec-17 ----SGEGYTPFYFI-----------------CEL--------------

DpLec-2 QSSDNSSFSRPMSYI-----------------CES--------------

DpLec-7 ---QA--MN----FI-----------------CESIA------------

DpLec-11 ---QA--MN----FI-----------------CESIA------------

DpLec-14 ----NLSQY----FI-----------------CEW--------------

DpLec-4 ----TNSFR----FI-----------------CESIN------------

DpLec-8 ----IYSTR----FV-----------------CESIE------------

DpLec-9 ---ATPGFK----FI-----------------CEQND------------

DpLec-5 ----PAYIL----FI-----------------CESIDE-----------

DpLec-6 DATTASTTS----VT-----------------TKATTETTK--------

DmLec-14 HNHASSLFK----YI-----------------CEAPKQ-----------

DmLec-15 SQDPNSLFK----YI-----------------CEAPE------------

DmLec-20 SQDPNSLFK----YI-----------------CEAPEME----------

DmLec-4 ----NFESS----FI-----------------CEEVQPK----------

DmLec-5 ----TFKTG----YI-----------------CRAPQP-----------

DmLec-2 ----KIQML----YV-----------------CEATEPKTF--------

DpLec-12 ----DFTNY----FI-----------------CEGNS------------

DpLec-19 ----SFETY----FV-----------------CEAPL------------

ApLec-1 ----SFETF----FV-----------------CEM--------------

DmLec-1 ----SFETY----FV-----------------CEVQPN-----------

DpLec-28 ----ANLYD----AV-----------------CESHPED----------

DpLec-26 ---TTTLAN----AI-----------------CEAHP------------

DpLec-27 ----ATLAR----GI-----------------CEVQP------------

DpLec-25 ---ATTMAH----AV-----------------CEAQP------------

DpLec-21 ----TTMAH----AI-----------------CEAQP------------

**O. I87.**

IsBand7-8 ------KGFVDGGHRAVIFDRFTGV-KNYVVGEGTH-FLIPWVQRPIIYD-VRSRPRNVP

SmBand7-4 ------LYNVDGGHRAVIFDRFTGV-KNFVVGEGTH-FLIPWVQKPIIYD-IRSRPRNVP

TuBand7-12 ------LYNVDGGHRAVIFDRFSGV-KPNVVGEGTH-FLIPWVQKPIIFD-VRSRPRNVP

DmBand7-8 ------LYNVEGGHRAVIFDRFTGI-KENVVGEGTH-FFIPWVQRPIIFD-IRSQPRNVP

DpBand7-4 -------YNVEGGHRAVIFDRFSGV-KNEVVGEGTH-FFVPWVQKPIIYD-IRSRPRNVP

AmBand7-4 ------LYNVDGGHRAVIFDRFTGI-KNQVVGEGTH-FIIPWVQRPIIFD-VRSRPRNIP

PhBand7-5 ------LYNVDGGHRAVIFDRFAGV-KNQVIGEGTH-FFIPWVQRPIIFD-TRSRPRNVP

TcBand7-5 -------YNVDGGHRAVIFDRFSGI-KKQVIGEGTH-FFIPWVQRPIIFD-VRSRPRNVP

ApBand7-4 SVANTALYNVDGGHRAVIFDRFTGI-KNTVVGEGTH-FLIPWVQKPIIFD-VRSRPRNVP

AgBand7-4 ------LYNVDGGHRAVIFDRFSGV-KQQVTGEGTH-FFVPWVQRPIIFD-IRSQPRNVP

BmBand7-3 ------LYNVDGGHRAVIFDRFAGV-KQLVVGEGTH-FFIPWVQRPIIFD-IRSRPRNVP

RpBand7-5 ------LYNVDGGHRAVIFDRFTGV-KNQVVGEGTH-FFIPWIQRPIIFD-IRSRPRNIP

CfBand7-3 ---NSALYNVDGGHRAVIFDRFAGI-KNAVIGEGTH-FFIPWVQKPIIFD-IRSRPRNVP

NvBand7-4 -------YNVDGGHRAVIFDRFVGV-KNNVTGEGTH-FFIPWIQKPIIFD-IRSRPRNVP

ApBand7-6 ----NSMFTVEGGHRAIMFNRIGGI-QREVYPEGLH-FRLPWFQYPVIFD-IRSRPRKIS

AmBand7-6 -----SMYTVEAGHRAIIFSRLGGI-QQDILTEGLH-FRIPWFHWPIIYD-IRSRPRKLS

IsBand7-9 -----SVFTVDGGHRAIIFNRIGGI-QKDVFAEGLH-FRIPWIQYPIIYD-IRSRPRKIS

DmBand7-10 ----QSLYTVEGGHRAIIFSRLGGI-QSDIYSEGLH-VRIPWFQYPIIYD-IRSRPRKIS

TcBand7-6 -----AMYTVEGGHRAIMFNRIGGV-QKDIYTEGLH-FRVPWFQYPIIYD-IRSRPRKIS

PhBand7-4 -----SLYTVEGGHRAIIFSRIGGI-QKEVYSEGLH-FKIPWLEYPIIYD-IRSRPRKIS

RpBand7-7 -----SMYTVEGGHRAIIFSRLNGI-QKDVFSEGLH-FRVPWFQYPIIYD-IRSRPRKIS

AmBand7-7 -----NVHTCGPNEALVVSGGCCGSMKKRTIVGGYA-FTWWFVTDVQRLS-LEVMTLNPV

ApBand7-5 ----WGFVTCGPNEALVIS---GFCYGKPNLVPGGRAFVWPVIQYCQRIC-LNTMTIQVD

SmBand7-5 ------FVTCGPNEALVVS---GCCHSKPLLVPGGRAFVWPFVQQVQRIS-LNTITLVVD

BmBand7-4 -------VTCGPNEALVIS---GCCYSKPLLVPGGRAFVWPAIQSVQRIS-LNTMTLQVE

AmBand7-5 -----GFVTCGPNEALVVS---GCCYSKPLLVPGGRVFVWPIVQQVQKIS-LNTMTLQVE

DmBand7-9 -----GFVTCGPNEALVVS---GCCYMKPLLVPGGRAFVWPVGQQVQRIS-LNTMTLQVE

PhBand7-3 -----GFVTCGPNEALVVS---GCCYNKPLLVPGGRAFVWPGIQEVQRIS-LNTMTLQVE

AgBand7-5 -----GFVTCGPNEALVVS---GCCHMKPLLVPGGRAFVWPSIQQVQRIS-LNTMTLQVE

RpBand7-6 ------FVTCGPNEALVVS---GCCHTKPLLVPGGRAFVWPTLQCVQRIS-LNTMTLQVE

TuBand7-14 ------VKKIKPLERCIIY-RLGK--RLPIKGPGLV-IVIPFVDVIDFID-LNPHRLCVV

ApBand7-3 --FC--LHRVDEGHVAVYY-RGGALLS-QISYPGYH-IMMPFLTTFRSVQ-VTLQTDEVK

IsBand7-7 ------LFSVDQRQSAVVF-QFGEA-VRTIENPGLN-IKIPFIQNVEFFD-KRLLDVEVE

IsBand7-5 ------VKVVPQQQAWVVE-KLGKF--DKVLQPGLN-LLIPVIQRVAYKHTLKEEAIDVT

IsBand7-6 --------------------AHGQV--SRILEPGLN-LLLPIVDRVRYVQSLKELAIDVP

TuBand7-10 ------VIFVPQQEAWVVE-RMGKF--HQICEPGLN-FLIPIVDRVKYVQSLKEIAIDIP

CfBand7-2 --------FVPQQQAWIVE-RMGKF--HKILEPGLN-ILFPVVDKVKYVQILKEMAIDVP

ApBand7-2 ---NTGILFVPQQEAWIVE-RMGKF--NRILEPGLN-FLIPFLDRIGYVQSLKELAIDIP

AmBand7-3 -------LFVPQQEAWIVE-RMGKF--HRILNPGLN-ILTPIIDKIKYVQCLKEIAIEIP

DpBand7-3 ----TIMLFVPQQEAWVVE-RMGKF--HKILKPGLN-FLIPVLDNIKYVQSLKEIAIDVP

RpBand7-4 --------FIPQQEAWIVE-RMGKF--HRILEPGLN-ILFPILDSVKYVQSLKEMAIDIP

NvBand7-3 ------VMFVPQQEAWIVE-RMGKF--HRILEPGLN-LLIPVIDSVRYVQSLKEIAIDVP

DmBand7-7 -----CVMFVPQQEAWVVE-RMGRF--HRILDPGLN-ILVPVADKIKYVQSLKEIAIDVP

TcBand7-4 ------IMFVPQQEAWVVE-RMGKF--HRILEPGLN-VLIPVVDRVKYVQSLKEIAVDIP

AgBand7-3 ------IMFVPQQEAWIVE-RMGKF--HRILEPGLN-VLLPVVDRVKYVQSLKEIAIDVP

DmBand7-4 -------TIVPEYSRMIIL-RLGRL-RKGLRGPGLV-FILPCIDETHRVD-MRTDVTNVR

DmBand7-5 ----------YEFHRLVIF-RLGRI--RSCLGPGLV-FLLPCIDSFNTVD-IRTDVVNVD

RpBand7-3 ---C---KIAYQYERVVIL-RLGRVREGGPRGPGIY-LYLPCVDERMKLD-LRTLVFQLD

DmBand7-6 ----------SEYERAVIL-RLGRLRPKPPRGPGVI-FLVPCIDDLAVVD-IRTRSFDLH

TuBand7-1 ---K--SYVVQDYERSVTL-RLGNLVGDGAKGSGII-FIIPCIDTYCKVD-LRTVSFNVP

RpBand7-2 ---------VNQFERAVIL-RFGKLRKGRARGPGLI-FVLVCIDNVMKVD-LRTATYAIP

SmBand7-3 ---C--WVVIKEYERAITF-RFGRLRKIEPRGPGLI-FILPCVDTYNVVD-LRTLYFDVP

TuBand7-3 ------VEIIKEYERAVVF-RLGRLKKGGVEGPGVH-VLIPCIDTLSKVD-LRTITFDVP

IsBand7-2 ---C--IVIVKEYERAVIF-RMGRLLPGGAKGPGLF-FIVPCTDNYSVVE-LRTWAFDVP

IsBand7-3 ---C--LVVVQEFERAVIF-RLGRLQPGGAAGPGLF-FIIPCIDEYRVVD-LRTVVFNVC

IsBand7-4 ---C--IVIANEYQRVVIF-RLGRLVSGGARGPGLF-FIIPCVDRYCEID-LRTISIDVP

TcBand7-2 ---C--LKIVQEYERAVIF-RLGRLRSGGPRGPGIF-FILPCIDDYIKID-LRTVTFDIP

DpBand7-2 --IC--FKVVQEYERAVIF-RLGRLLSGGAKGPGIF-FILPCIETYTKVD-LRTGVFDIP

SmBand7-2 ---C--LKVVQEYERAVIF-RLGRLLSGGAKGPGLL-FILPCIEDFVNID-MRTLTFDVP

TuBand7-2 ---C--VKVVQEYERAVIF-RLGRLLDGGAKGPGIF-IILPCIENYTKVD-LRTLTFDVP

TuBand7-8 ---C--VKVVQEYERAVIF-RLGRLLSGGAKGPGIF-IILPCIENYTKVD-LRTLTFDVP

PhBand7-1 ---C--FKVVQEYERAVIF-RLGRLLSGGAKGPGIF-FILPCVDNYAKVD-LRSSVFDIR

ApBand7-1 -------KVVQEYERAVIF-RLGRLVSGGAKGPGIF-FILPCIDNYARVD-LRTRTYDVP

TcBand7-3 ---C--FKVVQEYERAVIF-RLGRLLSGGAKGPGIF-FILPCIDAYARVD-LRTRTYDIP

AgBand7-1 ---C--FKVVQEYERAVIF-RLGRLMQGGAKGPGIF-FILPCIDAYARVD-LRTRTYDVP

DmBand7-1 ---C--FKVVQEYERAVIF-RLGRLMQGGAKGPGIF-FILPCIDSYARVD-LRTRTYDVP

BmBand7-2 ---C--FKVVQEYERAVIF-RLGRLLSGGAKGPGIF-FILPCIDTYARVD-LRTRTYDVP

AmBand7-1 -------MVVQEYERAVIF-RLGRLLSGGAKGPGIF-FILPCVDNYARVD-LRTRTYDVP

CfBand7-1 ---C--FKVVQEYERAVIF-RLGRLLSGGAKGPGIF-FILPCVDNYARVD-LRTRTYDVP

NvBand7-2 ---C--FKVVQEYERAVIF-RLGRLLSGGAKGPGIF-FILPCVDSYARVD-LRTRTYDVP

DpBand7-1 ---CFSVKVVQEYERAVIF-RLGRLLKGGARGPGIF-FIVPCIDTYRKID-LRTVSFDVP

IsBand7-1 ---C--VKIVQEYERAVIF-RLGRLVKGGARGPGLF-FIIPCIDNYTKVD-LRTVSFDVP

SmBand7-1 ---C--VKVVQEYERAVIF-RLGRLVKGGARGPGIF-FIIPCIDTYRKVD-LRTVSFDVA

TuBand7-7 ---C--IKVVQEYERAVIF-RLGRLLKGGARGPGIF-FVIPCIDSYTKVD-LRTVTFDVP

TuBand7-6 ------VKVVQEYERAVIF-RLGRLVKGGARGPGIF-FIIPCIDSYCKID-LRTVTFDVP

TuBand7-9 --FC--IKVVQEYERAVIF-RLGRLLKGGARGPGIF-FIIPCIDSYCKID-LRTVSFDVP

TuBand7-5 ------NIVVQEYERAVIF-RLGRLVKGGAKGPGIF-FIIPCIDTYSKVD-LRTVSFDVP

TuBand7-11 ---C--IKVVQEYERAVIF-RLGRLVSGGARGPGIF-FIIPCIDTYSKVD-LRTVSFDVP

TuBand7-13 ---C--IKVVQEYERAVIF-RLGRLVTGGARGPGIF-FIIPCIDTYSKVD-LRTVSFDVP

TuBand7-4 ---C--IKVVQEYERAVIF-RLGRLVTGGARGPGIF-FIIPCIDTYSKVD-LRTVSFDVP

DmBand7-3 ---C--FKVVAEYERAIIF-RLGRLS-GGARGPGMF-FILPCIDEYRKVD-LRTVTFNVP

BmBand7-1 ---C--FKVVQEFERAVIF-RLGRLRKGGARGPGLF-FVLPCIDTYRKVD-LRTVSFDVP

AmBand7-2 ---CFTFKVVQEYERAVVF-RMGRL-KGAAYGPGTF-FVMPCVDNCVRVD-LRTVSFDVP

NvBand7-1 ---CVIFKVVQEYERAVVF-RMGRL-KAGPQGPGTF-FVIPCIDNCVRVD-LRTVSFDVP

TcBand7-1 ---C--FKVVQEYERAVIF-RLGRLRTGGARGPGIF-FILPCVDSYCKVD-LRTVSFDVP

AgBand7-2 ---C--FKVVQEYERAVIF-RLGRLRSGGARGPGVF-FVLPCIDNYCKVD-LRTVSFDVP

DmBand7-2 ---C--FKVVSEYERAVIF-RMGRLRSGGARGPGVF-FVLPCVDDYYPVD-LRTVSFDVP

RpBand7-1 ---V--FKVVQEYERAVIF-RLGRLRAGGARGPGIF-FVLPCIDVYAKVD-LRTVSFDVP

PhBand7-2 ------FRVVQEYERAVIF-RLGRLRKGGPRGPGIF-FVLPCIDSYSKVD-LRTVSFDVP

* .

IsBand7-8 VVT------------------------------GSKDLQNVNITLRILFRPVQ--EQLPR

SmBand7-4 VVT------------------------------GSKDLQNVNITLRILFRPIP--DTLPK

TuBand7-12 VVT------------------------------GSKDLQNVNITLRILFRPVP--ENLPK

DmBand7-8 VIT------------------------------GSKDLQNVNITLRILYRPIP--DQLPK

DpBand7-4 VIT------------------------------GSKDLQNVNITLRVLFRPVP--TSLPN

AmBand7-4 VIT------------------------------GSKDLQNVNITLRILFRPIP--DSLPK

PhBand7-5 VIT------------------------------GSKGNIVII--------PLP--EQLPR

TcBand7-5 VIT------------------------------GSKDLQNVNITLRILFRPVP--DQLPR

ApBand7-4 VIT------------------------------GSKDLQNVNITLRILFRPLP--EQLPK

AgBand7-4 VIT------------------------------GSKDLQNVNITLRILFRPVP--DQLPK

BmBand7-3 TIT------------------------------GSKDLQNVNITLRILFRPVP--DQLPR

RpBand7-5 TVT------------------------------GSKDLQNVNITLRILFRPVP--DQLPK

CfBand7-3 VIT------------------------------GSKDLQNVNITLRILFRPVP--DSLPK

NvBand7-4 VIT------------------------------GSKDLQNVNITLRILFRPVP--ESLPK

ApBand7-6 SPT------------------------------GSKDLQMVNISLRVLSRPDA--IKLPD

AmBand7-6 SPT------------------------------GSKDLQMVNISLRVLSRPDA--QSLPT

IsBand7-9 SPT------------------------------GSKDLQMVNISLRVLARPDA--IMLPT

DmBand7-10 SPT------------------------------GSKDLQMINISLRVLSRPDS--LNLPY

TcBand7-6 SPT------------------------------GSKDLQMVNISLRVLSRPNA--SQLPI

PhBand7-4 SPT------------------------------GSKDLQMVMISLRVLSRPDA--INLPT

RpBand7-7 SPT------------------------------GSKDLQMVNISLRVLSRPDS--LQLPF

AmBand7-7 CES-----------------------------VETAQGVPLT-VTGVAQCKIM--KADEL

ApBand7-5 SPK-----------------------------VYTIQGVPLS-VTGIAQVKIQGQNEEML

SmBand7-5 SPK-----------------------------VYTNQGVAIS-VTGIAQVKIQGQNEDML

BmBand7-4 SPT-----------------------------VYTSQGVPIS-VTGIAQVKIQGQNSEML

AmBand7-5 SPT-----------------------------VYTCQGVPIS-VTGIAQVKIQGQNEEML

DmBand7-9 SPC-----------------------------VYTSQGVPIS-VTGIAQVKVQGQNEDML

PhBand7-3 SPT-----------------------------VYTSQGVPIS-VTGIAQVKIQGQNEEML

AgBand7-5 SPT-----------------------------VYTSQGVPIS-VTGIAQVKIQGQNEDML

RpBand7-6 SPT-----------------------------VYTSQGVPIS-VTGIAQVKIQGQNEEML

TuBand7-14 SKE----------------------------QMLTSDGSLIEFIDFTVEMTVF--NAIRT

ApBand7-3 NVP-----------------------------CGTSGGVMIY-FDRIEVVNIL--NASSV

IsBand7-7 AKE-----------------------------LTAADGKRVI-VDAYAKFQIN--NPVMF

IsBand7-5 AQT-----------------------------AISNDNVTLS-IDGVLYVKII--DPMAA

IsBand7-6 QQS-----------------------------AITLDNVTLN-IDGVLYLKVV--DPYRA

TuBand7-10 KQS-----------------------------AVTSDNVTLA-IDGVLYLRVV--DPYKA

CfBand7-2 QQS-----------------------------AVTSDNVTLS-IDAVLYLKVT--DPYLT

ApBand7-2 KQT-----------------------------AVTLDNVTLN-IDGVLYLRVN--DPYLA

AmBand7-3 QQS-----------------------------AVTSDNVTLN-IDGILYLRVV--NPFLA

DpBand7-3 QQS-----------------------------AITLDNVTLS-IDGVLYLRIV--DPYKA

RpBand7-4 KQS-----------------------------AITSDNVTLN-IDGVLYLRVL--DPYLA

NvBand7-3 KQS-----------------------------AITSDNVTLS-IDGVLYLKIN--NPYLA

DmBand7-7 KQS-----------------------------AITSDNVTLS-IDGVLYLRII--DPYKA

TcBand7-4 KQS-----------------------------AITSDNVTLN-IDGVLYLRIV--DAYLA

AgBand7-3 KQS-----------------------------AITSDNVTLS-IDGVLYLRIL--DPYLA

DmBand7-4 PQD-----------------------------VLTKDSVTIT-VNAVVYYCIY--SPIDS

DmBand7-5 PQE-----------------------------MLTKDSVSIT-VNAVVFYCIY--DPINS

RpBand7-3 PQQ-----------------------------VLTNDSCTLQ-IDVIIFCRVE--DPVKT

DmBand7-6 RQE-----------------------------ILTRDMVTIS-IDGVVYYSIK--SPFDA

TuBand7-1 PQE-----------------------------VLTKDLVCIK-VDAVVYYRIV--DSTAS

RpBand7-2 PQE-----------------------------VLTKDSCTVS-VDAVVYYSVS--DPIRA

SmBand7-3 PQE-----------------------------ILTKDSVTIA-VDAVVYYRTF--DATMA

TuBand7-3 PQE-----------------------------ILTSDSVTVS-VDAVVYFRIC--NPIFA

IsBand7-2 PQE-----------------------------VLSKDSVTLA-VDAVVYYRVF--NPVIA

IsBand7-3 PQE-----------------------------ILSKDSVTVA-VDAVVYYRVF--NPVAA

IsBand7-4 AQE-----------------------------ILSRDSVTVT-VDAVIYYRIV--NPIAS

TcBand7-2 PQE-----------------------------VLSKDSVTIW-VDAVVYFRVE--DPLAA

DpBand7-2 PQE-----------------------------VLTKDSVTVS-VDAVVYFRVS--NATVS

SmBand7-2 PQE-----------------------------VLTKDSVTVS-VDAVVYYRVS--NATIS

TuBand7-2 PQE-----------------------------VLTKDSVTVS-VDAVVYYRVF--NATVS

TuBand7-8 PQE-----------------------------ILTKDSVTVS-VDAVVYYRVN--NATIS

PhBand7-1 PQE-----------------------------VLTKDSVTVS-VDAVVYYRVC--NATIS

ApBand7-1 PQE-----------------------------VLTKDSVTVS-VDAVVYYRVC--NATIS

TcBand7-3 PQE-----------------------------VLTKDSVTVS-VDAVVYYRVS--NATVS

AgBand7-1 PQE-----------------------------VLTKDSVTVS-VDAVVYYRVS--NATVS

DmBand7-1 PQE-----------------------------VLTKDSVTVS-VDAVVYYRVS--NATVS

BmBand7-2 PQE-----------------------------VLTKDSVTVS-VDAVVYYRVH--NATIS

AmBand7-1 PQE-----------------------------VLTKDSVTVS-VDAVVYYRVN--NATIS

CfBand7-1 PQE-----------------------------VLTKDSVTVS-VDAVVYYRVN--NATIS

NvBand7-2 PQE-----------------------------VLTKDSVTVS-VDAVVYYRVN--NATIS

DpBand7-1 PQE-----------------------------ILSRDSVTVA-VDAVVYYRVH--NPTIA

IsBand7-1 PQE-----------------------------ILTKDSVTVA-VDAVVYYRIQ--NATVA

SmBand7-1 PQE-----------------------------ILSKDSVTVA-VDAVVYYRIS--NATIA

TuBand7-7 PQE-----------------------------VLSKDSVTTT-VDAVVYYRIS--NATVA

TuBand7-6 PQE-----------------------------ILSKDSVTVA-VDAVVYYRIS--NATVA

TuBand7-9 PQE-----------------------------ILSKDSVTVA-VDAVVYYRIS--NATVA

TuBand7-5 PQE-----------------------------ILSKDSVTVA-VDAVVYYRIS--NATIA

TuBand7-11 PQE-----------------------------ILSKDSVTVA-VDAVVYFRIS--NAIAS

TuBand7-13 PQESKVVNYKMDAQKAPAMIEAELSLRHGAMKILSKDSVTVA-VDAVVYFRIN--NAIAS

TuBand7-4 PQE-----------------------------ILSKDSVTVA-VDAVVYFRIS--NAIAS

DmBand7-3 QQE-----------------------------MLTKDSVTVT-VDAVVYYRIS--DPLYA

BmBand7-1 PQE-----------------------------VLTRDSVTVA-VDAVVYYRIK--EPLNA

AmBand7-2 PQE-----------------------------VLTKDSVTVS-VDAVVYYRIK--EPLNA

NvBand7-1 PQE-----------------------------VLTKDSVTVS-VDAVVYYRIK--EPLNA

TcBand7-1 PQE-----------------------------ALTKDSVTVT-VDAVVYYRIQ--DPLNA

AgBand7-2 PQE-----------------------------VLTRDSVTVS-VDAVVYYRIR--DPLNA

DmBand7-2 PQE-----------------------------VLSKDSVTVT-VDAVVYYRIS--DPLKA

RpBand7-1 PQE-----------------------------VLTKDSCTVC-VDAVVYYRIE--DPLRS

PhBand7-2 PQE-----------------------------VLTKDSVTVT-VDAVVYYNIK--DPLSA

:

IsBand7-8 MYTTLGV--DYDERVLPSITNEVL----KAVVAQFDASEMIT-QREVVSQKVCDELTERA

SmBand7-4 LYMSLGS--DYDERVLPSITNEVL----KAVVAQFDASELIT-QREIVSQRVSEALIERA

TuBand7-12 MYSSLGV--DYDERVLPSITNEVL----KAVVAQFDAGELIT-QREIVSQRVSEDLTERA

DmBand7-8 IYTILGQ--DYDERVLPSIAPEVL----KAVVAQFDAGELIT-QREMVSQRVSQELTVRA

DpBand7-4 IYSTLGI--DYDERVLPSITNEIL----KAVVAQFDAGELIT-QREVVSQKVSEALTERA

AmBand7-4 IYTVLGI--DYAERVLPSITNEVL----KAVVAQFDAGELIT-QREIVSQKVREDLTERA

PhBand7-5 IYTILGV--DYDERVLPSITTEVL----KAVVAQFDAGELIT-QREVVSQKVSEELTDRA

TcBand7-5 IYTVLGQ--DYEERVLPSITTEVL----KAVVAQFDAGELIT-QRDLVSQKVSEDLTERA

ApBand7-4 IYTILGV--DYDERVLPSITTEVL----KAVVAQFDAGELIT-QRENVSRKVSETLIERA

AgBand7-4 IYTILGQ--DYDERVLPSITTEVL----KAVVAQFDAGELIT-QREMVSQKVSDDLTERA

BmBand7-3 IYTILGI--DYDERVLPSITSEVL----KAVVAQFDAGELIT-QREIVSQKVNDSLTERA

RpBand7-5 IYTILGV--DYDERVLPSITTEVL----KAVVAQFDAGELIT-QRELVSQKVNEELTERA

CfBand7-3 IYTILGV--DYDERVLPSITTEVL----KAVVAQFDAGELIT-QRELVSQKVSDDLTDRA

NvBand7-4 IYTILGV--DYDERVLPSITTEVL----KAVVAQFDAGELIT-QRELVSQKVSEDLTERA

ApBand7-6 MYQHLGI--DYDEKVLPSICNEVL----KSVVAKYNASQLIT-QRQQVSLLIRKQLVDRA

AmBand7-6 MYRQLGL--DYDEKVLPSICNEVL----KSVVAKFNASQLIT-QRQQVSNLVRKELTERA

IsBand7-9 VYRMLGT--DYDERVLPSICNEVL----KSVVAKFNASQLIT-QRQQVSLLVRRELTERA

DmBand7-10 LHKQLGV--DYDEKVLPSICNEVL----KSVIAKFNASQLIT-QRQQVSLLIRKELVERA

TcBand7-6 VYRQLGL--DYDEKVLPSICNEVL----KSVVAKFNAAQLIT-QRQQVSLLVRRELTERA

PhBand7-4 MYRTLGL--DYDEKVLPSICNEVL----KSVVAKFNASQLIT-QRQQVSLLVRRELTERA

RpBand7-7 MYRQLGL--DYDEKVLPSICNEVL----KSVVAKFNASQLIT-QRQQVSLLVRRELIERA

AmBand7-7 LHTASEQFLGKSVYEIKSTILSTLEGHLRAILGTLSVEEVYK-DRDQFATLVREVAAPDV

ApBand7-5 LTACEQF-LGKPKQEIHEIALHTLEGHQRAIMGSMTVEEIYK-DRKKFSKQVFEVASSDL

SmBand7-5 MTACQQF-LGKTEDQVKGIALVTLEGHQRAIMGTMTVEEIYK-DRKKFSKQVFEVASSDL

BmBand7-4 LSACEQF-LGKTEQEIQHIALVTLEGHQRAIMGSMTVEEIYK-DRKIFSKKVFEVASSDL

AmBand7-5 STACEQF-LGKTEEEIHNIALVTLEGHQRAIMGSMTVEEIYK-DRKKFSKEVFEVASSDL

DmBand7-9 LTACEQF-LGKSEAEINHIALVTLEGHQRAIMGSMTVEEIYK-DRKKFSKQVFEVASSDL

PhBand7-3 TAACEQF-LGKSENEIQNIALVTLEGHQRAIMGSMTVEEIYK-DRKKFSKHVFEVASSDL

AgBand7-5 LTACEQF-LGKSEAEIQHIALVTLEGHQRAIMGSMTVEEIYK-DRKKFSKQVFEVASSDL

RpBand7-6 LAACEQF-LGKSDAEIQHIALVTLEGHQRAIMGSMTVEEIYK-DRKKFSKHVFEVASSDL

TuBand7-14 STQLKDS--RQNVDQFV---KLSF----LNTMGGIHVEDLER-KMEFIIKQYAETCNQYI

ApBand7-3 FDIVKNYTADYDKTLIFNKVHHEL----NQFCSVHNLHEVYIDLFDQIDENLKVALQKDL

IsBand7-7 YKTVHDY--QGVKIRLTRNLESSM----RKVIGKISLSSLLSQERINVMLNILNQVDGEA

IsBand7-5 SYGVNNP--YYAITQLA---QTTM----RSEIGKLPLDRTFE-ERETLNVAIVAAINQAA

IsBand7-6 SYGVEDP--EFAITQLA---QTTM----RSELGKIALDSVFK-ERESLNIAIVDAINKAS

TuBand7-10 SYGVEDP--EFAITQLA---QTTM----RSEIGKITLDTVFK-ERETLNIAIVEAINKAG

CfBand7-2 SYGVEDA--EFAIIQVA---QTTM----RSELGKIPLDKVFR-EREELNVSIVESINKAS

ApBand7-2 SYGVEDP--EFAITQLA---QTTM----RSELGKISLDKVFR-ERENLNFAIVESLNKAS

AmBand7-3 SYGVDDP--EFAVVQLA---QTTM----RSELGKISLDKVFR-EREGLNVCIVDSINKAS

DpBand7-3 SYGVEDA--EFAITQLA---QTTM----RSELGKIHLDSVFR-ERENLNLGIVEAINKAS

RpBand7-4 SYGVEDP--EFAITQLA---QTTM----RSEIGKISLDNVFR-ERESLNVGIVASINKAS

NvBand7-3 SYGVQDP--EFAIIQLA---QTTM----RSELGKIALDKVFQ-EREGLNISIVESINKAS

DmBand7-7 SYGVEDP--EFAITQLA---QTTM----RSELGKMSMDKVFR-ERESLNVSIVDSINKAS

TcBand7-4 SYGVEDP--EFAITQLA---QTTM----RSELGKISLDKVFR-ERENLNVSIVDSINKAS

AgBand7-3 SYGVEDP--EFAITQLA---QTTM----RSELGKMSLDKVFR-ERESLNISIVESINKAS

DmBand7-4 IIQVDDA--KQATQLIS---QVTL----RNIVGSKTLNVLLT-SRQQLSREIQQAVAGIT

DmBand7-5 IIKVDDA--RDATERIS---QVTL----RNIVGSKGLHELLA-SRQQLSLEIQQAVAKIT

RpBand7-3 VVAVSDL--RVATLNLA---STIL----RNIIGQRDLTEILS-QKDAITLALKKILDVGT

DmBand7-6 MLQVYDP--EEATEKLA---MTTL----RNVAGTHKLMDLLS-SKEYLSNQIEGILYNST

TuBand7-1 ILNVSDV--GRSTRRLA---AITL----RNVVATKTLTEIIS-ERVIISSKIENNLQEAT

RpBand7-2 VVQVVSF--RYSTCTLA---ATIL----RNIMGQKNLTEILS-ERESIAFVIKEALDSAT

SmBand7-3 ITNVQDY--KKASHLLA---ASIL----RNTLGTKNMVDILT-QRESLSYAMQKQLDEAT

TuBand7-3 VTNVQDY--RRSTQLLA---ATTL----RNVLGGKSLSQLLS-ELDTISQILKSNLDTTT

IsBand7-2 ITNVQDF--ARSTKLLA---SSIL----RNVLGTKSLSEMLS-ERDSISQLMQSTLDAAT

IsBand7-3 TVNIKDH--ARSTILLA---ATIL----RNVLGTKMLSDVLS-QRKSISRTMQTLLDVAT

IsBand7-4 VMNVEDY--FVATNLLA---AAML----RNVLGTKNLSDILS-DRESISQMMQSALDVAT

TcBand7-2 ILKVENF--RTSTHLLA---MTTL----RNILGTKTLMEILS-DRENIVHLMQTQLDVAT

DpBand7-2 VANVENA--HHSTRLLA---QTTL----RNILGTKDLHEILG-DRETISGSMQAALDEAT

SmBand7-2 VANVENA--HHSTRLLA---QTTL----RNMLGTKNLHEILS-DRENISATMQSNLDDGT

TuBand7-2 ISNVENA--HHSTRLLA---QTTL----RNILGTFNLQEVLI-SRESISTSMQTVLDEAT

TuBand7-8 IANVENA--HHSTRLLA---QTTL----RNMLGTHNLHEILS-DRELISTSMQTVLDEAT

PhBand7-1 VANVENA--HHSTRLLA---QTTL----RNTMGTRLLSEILS-ERENISQVMQSALDDAT

ApBand7-1 VANVANA--HQSTRLLA---QTTL----RNVLGTRPLHEILS-DRDAISKTMQVSLDEAT

TcBand7-3 IANVENA--HHSTRLLA---QTTL----RNIMGQRPLHEILS-ERESISQHMKALLDEAT

AgBand7-1 IANVENA--HHSTRLLA---QTTL----RNTMGTRHLHEILS-ERMTISGSMQLSLDEAT

DmBand7-1 IANVENA--HHSTRLLA---QTTL----RNTMGTRHLHEILS-ERMTISGTMQVQLDEAT

BmBand7-2 IANVENA--HHSTRLLA---QTTL----RNTMGTRPLHEILS-ERETISGNMQLSLDEAT

AmBand7-1 ITNVENA--HHSTKLLA---QTTL----RNTMGTRPLHEILS-ERETISGNMQVSLDEAT

CfBand7-1 IANVENA--HHSTRLLA---QTTL----RNTMGTRPLHEILS-ERETISGNMQVALDDAT

NvBand7-2 IANVENA--HHSTRLLA---QTTL----RNTMGTRPLHEILS-ERETISGNMQISLDEAT

DpBand7-1 VSNVENF--SHSTRLLA---ATTL----RNVLGTKNLAEVLS-ERETISHTMQSSLDEAT

IsBand7-1 VTNVEDY--GRSTRLLA---ATTL----RNVLGTKNLSEILS-EREPISHTMQTNLDEAT

SmBand7-1 VANVEDY--GHATRLLA---ATTL----RNVLGTKNLSEILA-ERETISHTMQAVLDEAT

TuBand7-7 VTNVEDY--GRSTRLLA---ATTL----RNVLGTKNLSELLS-ERESISHMIQSSLDEAT

TuBand7-6 VTNVADY--GMSTRLLA---ATTL----RNVLGTKNLSELLS-ERESISHMIQSSLDVAT

TuBand7-9 VTNVEDY--GRSTRLLA---ATTL----RNVLGTKNLSELLS-ERESISHMIQSSLDEAT

TuBand7-5 VSNVEDY--GRSTRLLA---ATTL----RNVLGTKNLSEILS-ERESISHIMQSSLDEAT

TuBand7-11 VSNVEDY--ARSTRLLA---ATTL----RNVLGTKNLSEILS-ERESISHMIQSSLDEAT

TuBand7-13 VSNVEDY--ARSTRLLA---ATTL----RNVLGTKNLSEILS-ERESISHMIQSSLDEAT

TuBand7-4 VSNVEDY--ARSTRLLA---ATTL----RNVLGTKNLSEILS-ERESISHMIQSSLDEAT

DmBand7-3 VIQVEDY--SMSTRLLA---ATTL----RNIVGTRNLSELLT-ERETLAHNMQATLDEAT

BmBand7-1 VVRVADY--SASTRLLA---ATTL----RNVLGMRDLAQLLS-DREAISHMMQASLDEAT

AmBand7-2 VIKIANY--SHSTRLLA---ASTL----RTVLGTRNLAEILS-ERETISHTMQTSLDEAT

NvBand7-1 VVKIANY--SHSTRLLA---ASTL----RTVLGTRSLAEILA-ERETISHTMQAALDEAT

TcBand7-1 VTKVTNY--SNSTRLLA---MTTL----RNILGTRNLAEILS-DREAISHAMQTNLDVAT

AgBand7-2 VVQVANY--SHSTRLLA---ATTL----RNVLGTRNLSELLT-EREAISHSMQVTLDEAT

DmBand7-2 VIQVYNY--SHSTSLLA---ATTL----RNVLGTRNLSELLT-ERETISHTMQMSLDEAT

RpBand7-1 VVAISNY--SHSTRLLA---ATTL----RNVLGTRNLAEILA-EREVISHTMQTALDLAT

PhBand7-2 VVQVSNY--SHSTQLLA---ATTL----RNVLGTKNLSEILS-ERETIAHTMQTSLDEAT

. : . .

IsBand7-8 SQ--FGVILDDISITHLTFGK------EFTQAVEMKQVAQQE----AERARFLVEKAEQQ

SmBand7-4 GQ--FGLFLDDISITHLTFGK------EFTHAVELKQVAQQD----AERARYLVEKAEQV

TuBand7-12 AQ--FGLLLDDISLTHLTFGR------EFTAAVEMKQVAQQD----AEKARFLVEKAEQL

DmBand7-8 KQ--FGFILDDISLTHLTFGR------EFTLAVEMKQVAQQE----AEKARFVVEKAEQQ

DpBand7-4 GQ--FGLILDDISITHLTFGK------EFTQAVELKQVAQQE----AERARFLVEKAEQL

AmBand7-4 TQ--FGLILDDISITHLTFGK------EFTQAVEMKQVAQQE----AEKARFLVEKAEQH

PhBand7-5 SQ--FGVILDDISITHLTFGK------EFTQAVELKQVAQQE----AEKARFLVEKAEQN

TcBand7-5 SQ--FGVILDDISITHLTFGR------EFTLAVELKQVAQQE----AEKARFLVEKAEQN

ApBand7-4 GQ--FGVVLDDISITHLTFGK------EFTQAVELKQVAQQD----AERARFLVEKAEQQ

AgBand7-4 AQ--FGVILDDISITHLTFGK------EFTQAVEMKQVAQQE----AEKARFMVEKAEQM

BmBand7-3 AQ--FGLILDDISITHLTFGK------EFTQAVELKQVAQQE----AEKARFLVEKAEQQ

RpBand7-5 AQ--FGVILDDIALTHLTFGK------EFTQAVELKQVAQQE----AERARFLVEKAEQQ

CfBand7-3 SQ--FGLILDDISITHLTFGK------EFTQAVELKQVAQQD----AEKARFLVEKAEQQ

NvBand7-4 SQ--FGVILDDISITHLTFGK------EFTQAVELKQVAQQE----AEKARFLVEKAEQQ

ApBand7-6 RD--FNIILDDVSITELSFGK------EYTAAVEAKQVAHQE----AQRAVFFVERAKQE

AmBand7-6 RD--FNIVLDDVSITELSFGK------EYTAAVESKQVAQQE----AQRAAFFVEKAKQE

IsBand7-9 RD--FNIILDDVSITELSFGK------EYAAAVEAKQVAQQE----AQRAMFTVEQAVQE

DmBand7-10 RD--FNIILDDVSLTELSFGK------EYTAAIEAKQVAQQE----AQRAVFFVERAKQE

TcBand7-6 RD--FNIILDDVSITELSFGK------EYTAAVEAKQVAQQE----AQRAAFIVEKAKQE

PhBand7-4 RD--FNIILDDVSITELSFGK------EYTAAVEAKQVAQQE----AQRAAFVVERAKQE

RpBand7-7 RD--FNIILDDVSITELSFGK------EYTAAVEAKQVAQQE----AQRAVFVVERAKQE

AmBand7-7 GR--MGIEILSFTIKDVYDDV------QYLASLG-KAQTAAV----KRDADVGVAEANRD

ApBand7-5 VN--MGITVVSYTIKDIRDEE------GYLRALG-LARTAEV----KRDARIGEAEAKRE

SmBand7-5 VN--MGITVVSYTLKDIRDDE------GYLKALG-MARTAEV----KRDARIGEADARRD

BmBand7-4 IN--MGITVVSYTLKDIRDEE------GYLKALG-MARTAEV----KRDARIGEAEAQAE

AmBand7-5 VN--MGITVVSYTLKDIRDEE---GAKGYLKALG-MARTAEV----KRDARIGEAEARRD

DmBand7-9 AN--MGITVVSYTIKDLRDEE------GYLRSLG-MARTAEV----KRDARIGEAEARAE

PhBand7-3 VN--MGITVVSYTLKDIRDEE------GYLKSLG-KARTAEV----KRDARIGEAEARRD

AgBand7-5 VN--MGITVVSYTLKDIRDEEFNGSNRGYLKSLG-MARTAEV----KRDARIGEAEARCD

RpBand7-6 VN--MGITVVSYTLKDIRDEE------GYLKSLG-MARTAEV----KRDARIGEAEARKD

TuBand7-14 NK--WGWSMVVIEIPRIKVL----------------------------------------

ApBand7-3 TEMAPGLKVHAVRVTKPKIPE------TIRKNYEIMEAEKTKLLIAEQRQKVVEKEAETE

IsBand7-7 KS--FGIDVVDVRILRADLPK------ENSAAIY-RRMQTAR----EKEATQIRAEGQEE

IsBand7-5 IN--WGIQCMRYEIKDIQPPQ------TILKAME-LQVAAER----QKRAQILESEGNRQ

IsBand7-6 GA--WGIVCLRYEIRDIRLPQ------RVHEAMQ-MQVEAER----KKRAAVLESEGI--

TuBand7-10 LA--WGITCLRYEIRDIKLPE------RVQEALS-MQVEAER----RKRAVVLESEGRRE

CfBand7-2 NA--WGITCLRYEIRDIRFPP------RVQEAMQ-MQVEAER----KKRAAILESEGV--

ApBand7-2 AS--WGLVCFRYEIRDIKLPN------RVQEAMQ-MQVEAER----KKRAAILDSEGIRE

AmBand7-3 EA--WGITCLRYEIRDIRLPQ------RVQEAMQ-MQVEAER----KKRAAVLESEGARE

DpBand7-3 EA--WGIACLRYEIRDIKLPA------RVQEAMQ-MQVEAER----KKRAAILESEGIRE

RpBand7-4 EA--WGITCLRYEIRDIKLPV------RVQEAMQ-MQVEAER----KKRAAILESEGVR-

NvBand7-3 EA--WGISCLRYEIRDIKLPE------RVHVAMQ-MQVEAER----KKRAAILESEGI--

DmBand7-7 EA--WGIACLRYEIRDIRLPT------RVHEAMQ-MQVEAER----RKRAAILESEG---

TcBand7-4 EA--WGMTCLRYEIRDIKLPP------RVQEAMQ-MQVEAER----KKRAAILESE----

AgBand7-3 EA--WGISCLRYEIRDIKLPS------RVHEAMQ-MQVEAER----RKRAAILESEGVR-

DmBand7-4 YR--WGVRVERVDVMDITLPT------SLERSLA-SEAEAVR----EARAKIILAEGELK

DmBand7-5 ER--WGVRVERVDLMEISLPS------SLERSLA-SEAEATR----EARAKIILAEGEAK

RpBand7-3 FP--WGVKVLRVELMGIRLPL------NMQRAMA-SEAEATR----EAKAKMIFAEGELL

DmBand7-6 EP--WGIRVERVEIKEIFMPD------QLKRALA-VEQEAMR----EAKAKVAAAQGERD

TuBand7-1 RP--WGVDVERVEMKEVRLPA------QLQKLMA-TEAETER----EASAKIIIASGEQK

RpBand7-2 HP--WGITVERVEIKDVRLPV------QMQRAMA-AEAEATR----DARAKVIAAEGEL-

SmBand7-3 DP--WGVKIERVEMKDVRLPH------NMQRAMA-AEAEATR----EAKA----------

TuBand7-3 ES--WGVKVERVEIKDIRLPT------QLQRAMA-AEAEAMR----EARAKVIASEGEQK

IsBand7-2 DP--WGVKVERVEMKDFRIPV------QMQRAMA-AEAEAMR----EGRAKVIAAEGEQR

IsBand7-3 DP--WGVKVERVELTDVQLPA------QMQRAMA-AEAEAVR----EGRAKVVAAEGEQR

IsBand7-4 DP--WGVKVERVEIKDVRLPH------QMQRAMA-AEAEAVR----EGRAKVVAAEGEER

TcBand7-2 DP--WGIKVERVEITDIRLPQ------SLQRAMA-TEAEASR----EARAKIIAAEGEMN

DpBand7-2 ES--WGIKVERVEIKDVRLPV------QLQRAMA-AEAEASR----EARAKVIAAEGEFK

SmBand7-2 EG--WGIKVERVEIKDVRLPV------QLQRAMA-AEAEAAR----EARAKVIAAEGEQK

TuBand7-2 ER--WGIKVERTEITDVRLPV------QLQRAMA-AEAEAAR----EARAKVIAAEGEQK

TuBand7-8 ER--WGIKVERTEIKDVRLPV------QLQRAMA-AEAEAAR----EARAKVIAAEGEQK

PhBand7-1 VA--WGIKVERVEIKDVRLPI------QLQRAMA-AEAEASR----EARAKVIAAEGEQK

ApBand7-1 ES--WGIKVERVEIKDVRLPV------QLQRAMA-AEAEAAR----EARAKVIAAEGEQ-

TcBand7-3 DS--WGINVERVEIKDVRLPI------QLQRAMA-AEAEAAR----EARAKVIAAEGEQK

AgBand7-1 EA--WGIKVERVEIKDVRLPV------QLQRAMA-AEAEAAR----EARAKVIAAEGEQK

DmBand7-1 DA--WGIKVERVEIKDVRLPV------QLQRAMA-AEAEAAR----EARAKVIAAEGEQ-

BmBand7-2 EA--WGIKVERVEIKDVRLPV------QLQRAMA-AEAEAAR----EARAKVIAAEGEQK

AmBand7-1 DT--WGIKVERVEIKDVRLPV------QLQRAMA-AEAEAAR----EARAKVIAAEGEQK

CfBand7-1 DT--WGIKVERVEIKDVRLPV------QLQRAMA-AEAEAAR----EARAKVIAAEGEQK

NvBand7-2 DS--WGIKVERVEIKDVRLPV------QLQRAMA-AEAEAAR----EARAKVIAAEGEQK

DpBand7-1 DP--WGVKVERVEIKDVRLPV------QLQRAMA-AEAEAAR----EARAKVIAAEGEQK

IsBand7-1 DA--WGVKVERVEIKDVRLPV------QMQRAMA-AEAEASR----EARAKVIAAEGEQR

SmBand7-1 DP--WGVKVERVEMKDVRLPV------QLQRAMA-AEAEATR----EARAKVIAAEGEQR

TuBand7-7 DP--WGVKVERVEVKDVRLPQ------QLQRAMA-AEAEASR----EARAKVIAAEGEQK

TuBand7-6 DP--WGVKVERVEVKDVRLPQ------QLQRAMA-AEAEASR----EARAKVIAAEGEQK

TuBand7-9 DP--WGVKVERVEVKDVRLPV------QLQRAMA-AEAEAAR----EARAKVIAAEGEQK

TuBand7-5 DP--WGVKVERVEIKDARLPV------QLQRAMA-TEAEAAR----EARAKVIAAEGEQR

TuBand7-11 DS--WGVKVERVEVKDARLPF------QLQRAMA-AEAEATR----EARAKVIAAEGEQR

TuBand7-13 ES--WGVKVERVEIKDVRLPV------LLQRAMA-AEAEATR----EARAKVIAAEGEQR

TuBand7-4 DS--WGVKVERVEIKDVRLPV------QLQRAMA-AEAEAAR----EARAKVIAAEGEQR

DmBand7-3 EP--WGVMVERVEIKDVSLPV------SMQRAMA-AEAEAAR----DARAKVIAAEGEKK

BmBand7-1 EP--WGVEVERVEIKDVRLPV------QLQKAMA-AEAEADR----EARAKIIAAEGEIK

AmBand7-2 EP--WGVKVERVEIKDVRLPV------QLQRAMA-TEAEAAR----EARAKVIAAEGEML

NvBand7-1 EP--WGVKVERVEIKDVRLPV------QLQRAMA-AEAEAAR----EARAKVIAAEGEM-

TcBand7-1 DP--WGVKVERVEIKDVSLPQ------QLQRAMA-AEAEASR----EARAKVIAAEGEMK

AgBand7-2 DP--WGVQVERVEIKDVSLPD------SLQRSMA-AEAEAAR----EARAKVIAAEGEMK

DmBand7-2 DP--WGVKVERVEIKDVSLPT------ALQRAMA-AEAEAAR----EARAKVIAAEGEMK

RpBand7-1 EP--WGVKVERVEIKDVRLPV------QMQRAMA-AEAEATR----EARAKVIAAEGEMK

PhBand7-2 DP--WGVKVERVEIKDVRLPV------LLQKAMA-AEAEAAR----EACAKVIAAEGEMK

. :

1. **I93.**

SmFz-9 --QAQCE--NLHSNLCFGIALP-YKQTSLSLLGSSMN-QSEVQRQM-QLWAGLSSVPRCW

IsFz-5 -----CE--PIINATCFGVSLP-YGHTTVELVNDSTS-QLEIQERL-DLWQGLQQIPRCW

ApFz-4 ---NHSL-------SCLGVKLP-YTSTSTDLVPDAET-PEEAQERL-HYWQGLKKVPKCW

RpFz-6 ---AKCE--PLNYTTCLGVKLP-YTWTSLDLVDGLVS-QEQGQEQL-KEWRRLVHIPKCW

NvFz-6 ----KCV--KMINTTCLGATLP-YDTTSLDLMPKYTT-QEMIMEKL-HILRGLEHIPKCW

AmFz-4 ---AKCV--EIQKNTCMGTRLP-YTTTTLELIPEHIT-QDIIEEKL-HVLQTLRHVPKCW

CfFz-7 --SAKCV--PLHKGTCMGTKLP-YSFTSLDLIPERVT-QDIIEEKL-YSLQALKHVPKCW

AgFz-6 ---GKCE--PIKHRQCLHSTLP-YSSISL-DLTDSYS-QEEMHTKL-HQYNALKSVPKCW

TcFz-5 --PAKCQ--LLNYTTCMGMKLP-YFSTTL-ELTDLTT-QEKVQEKL-HHYKYLRFIPKCW

DpFz-4 ---AKCQ--PIKNNICLTTKLP-YSQTSLELVTDSHT-QDDVQDKL-MFWQNLKSVPKCW

DpFz-6 ---SQCV--PRNLAFCNNT-LN-YTETVYPNLSGDLS-EDDFVRSW-AFLQTVIDS-YCH

SmFz-8 --GSQCR--HRTLSFCDD--VSSYNSTYLPNLAGATT-EFEKWQLV-SYYNSIVDW-ECN

DmFz-7 --NSGCQ--STMLPMCQGV-LD-YDLT----FNREGA-APRDAVSM-AAYDSLIRA-NCS

RpFz-5 ----NCH--SPQLPMCRGV-IP-WDLTSIPSLPGIST-MESLREAM-PYFELILDS-GCS

TuFz-4 ---AECI--RRSLPMCNGQ-VP-YNSTVYPNYIGDAN-EIEASRSL-PYYNYIAKS-KCN

DpFz-5 -VEPECI--RRQLPFCRGV-LP-YSETILPNWVGDNT-EAERNFSV-PYFEIIAES-ECH

SmFz-6 --CTKCV--YNTVPMCQN--MG-YNLTLFPNTFKHGT-QEDVSEAL-LFYSALMKS-NCS

TuFz-7 --RHDCV--PMKVAYCIKHNVR-YSHITLPNKNDDDTSQESINIQL-AEYDPILSV-KCY

DmFz-4 --GLQCQ--PIAVSACQG--LG-YNMIALPNLAGHTN-QLEAELQI-AKLVPLIES-GCS

AgFz-4 ---GVCL--PVIVKFCQQHRVP-YNYTVFPNYIGHFG-QPEAQIEI-DLFEALVDV-QCY

DmFz-6 --PGTCL--PIIVRFCQGPQIP-YNYTVFPNYIGHFG-QLETQTDL-DSYEALVDV-RCY

PhFz-6 ---GFCF--KLIVEFCANHKVP-YNFTTFPNHVVNMN-QDQAEQEL-DLYDALVDV-RCY

BmFz-4 ---GVCL--PVIVSFCHQHRIS-YNFTVFPNYIGHFG-QRDAQQDL-EIYDAVVDV-RCY

NvFz-4 ----ICL--PVIVNFCQYHKIP-YNYTVFPNYMMHFS-QREAQHDL-ELYDAVIDV-RCY

RpFz-3 ----LCL--PVIVPFCIQHKVP-YNFTMFPNYIGHFN-QREASQEL-EVYDAVVDV-RCY

TcFz-4 --PGICL--PVIVNFCLQHKVP-YNYTVFPNYMGQFG-QRDAQQEL-ELYDAVVDV-RCY

CfFz-5 ---GVCL--PIIVNFCQQHNVP-YNYTVFPNYMGNFG-QREAQHEL-ELYDAVVDV-RCY

AmFz-3 --PGVCL--PIIVAFCKYHKIP-YNFTIFPNYMGNFG-QRDAQHEL-ELYSAVIDV-KCY

BmFz-5 ---GKCE--RITLPLCQE--LG-YNWTSMPNLMGHKD-QKEAEQAM-SPFSDILGS-GCS

IsFz-1 ---PTCLEIPANLTLCRD--IG-YSKMRLPNLLEHDS-MAEVQQQA-RSWVQLANR-RCH

DpFz-1 --QPTCLDIPRNLSLCHG--IR-YSKMRLPNLLDHDS-MAEVIQQA-ASWVPLLNV-RCH

SmFz-1 -VQPTCVDIPRNMTLCHD--IG-YTKMRLPNLLDHDT-MAEVSQQA-GSWVPLLNI-ECH

PhFz-4 --------------MCQN--LG-YNLTTLPNFMDHKD-QSQAERAL-GTFMPLVHY-NCS

CfFz-4 --GPKCE--KLSVSFCRG--LR-YNLTAMPNFMGHED-QRQAERGL-TTFMPLVHY-NCS

NvFz-3 ---AKCE--RLNVSFCRG--LR-YNLTAMPNFMGHED-QLQAEREL-AKLMPLVHY-NCS

TuFz-3 --VRTCE--QIKFESCKK--IG-YNVTGFPNSAGHET-QDEAGQTF-QTFDPLIKF-RCS

DmFz-2 --FRQCE--TIRIEMCRK--IG-YNETSMPNLVGNEM-QTDVEYTL-QTFAPLIEY-DCS

AgFz-1 --QRTCE--PIRIELCRG--IG-YNETSLPNIVGHEL-QSDANFTL-QTFFPLIQF-GCS

TcFz-2 --VRTCE--PIRVDMCTN--LG-YNMTEMPNLGGNDI-QQEADYTL-KSFSPLIQY-GCS

BmFz-2 ASVRTCE--PIKVAMCKN--IG-YNQTGMPNLARHTL-QADADITL-QTFSPLVQY-GCS

IsFz-2 ---RMCE--PIRIEMCKD--IG-YNVTGMPNLVGHEL-QQDAQLQL-QTFKPLVQY-GCA

SmFz-5 ----SCD--PIRIEMCRG--LG-YNVTSMPNLVGHEL-QQDAEMQL-QTFSPLVQY-GCS

CfFz-3 ---GACE--PIRIEMCRG--LG-YNVTVMPNLVGHEI-QGDADFTL-QTFSPLIQY-GCS

PhFz-1 ---RTCE--PIRVELCRG--LG-YNMTGMPNLVGHDL-QGDADFTL-QTFSPLIQY-GCS

SmFz-4 ----RCE--RITIPMCQD--MP-YNLTRMPNYMGHAE-QSEAAIEV-HEFIPLVEI-GCS

TuFz-6 --KKRCE--TVSVAFCRE--IG-YNETIFPNLLNHNS-QQNAGLAL-HRITPLIKV-NCS

BmFz-3 -QHGRCE--PITIQFCQK--LR-YNQTIFPNILNQAR-QEDAAANM-LLFTTLIKL-NCS

TuFz-2 --RSKCE--EITVPMCRG--IG-YNMTSMPNQFHHEK-QDEAGMEA-HQFWPLVEI-SCS

TuFz-1 ---PKCE--EITVPMCRG--IG-YNMTSMPNQFHHEK-QDEAGMEA-HQF-------RLW

TuFz-5 --RPKCE--EITVPMCRG--IG-YNMTSMPNQFHHEK-QDEAGMEA-HQFWPLVEI-SCS

DpFz-3 --DARCE--EITIPMCRG--IG-YNWTSMPNSLHHET-QEEAGLEV-HQFWPLVEI-QCS

TcFz-6 ---ERCE--DITIPMCMG--IG-YNQTRMPNELNHET-QEEAGLEV-HQFWPLVEI-KCS

IsFz-4 ----RCE--DITIPMCKG--IG-YNQTSMPNQFNQDT-QDEAGMEV-HQFWPLVEI-QCS

SmFz-2 -DPSRCE--EITIPMCRG--IG-YNATFMPNQFNHDT-QDEAGLEV-HQFWPLVEI-QCS

ApFz-2 --XGRCE--EITIPMCRG--IG-YNMTSMPNQLNHET-QEEAGMEV-HQFWPLVEI-NCS

BmFz-1 --QPRCQ--DITIPMCRG--IG-YNLTSFPNALDHDT-QEEAGLEV-HQYWPLVEI-KCS

AgFz-2 ---SRCE--EITIPMCRG--IG-YNLTSFPNEMNHET-QEEAGLEV-HQFWPLVEI-KCS

DmFz-3 --NLRCE--EITIPMCRG--IG-YNMTSFPNEMNHET-QDEAGLEV-HQFWPLVEI-KCS

NvFz-2 ---SRCE--EITIPMCKG--IG-YNLTTMPNELNHDT-QDEAGLEV-HQFWPLVEI-KCS

AmFz-1 --NGRCE--EITIPMCRG--IG-YNLTAMPNELNHDN-QEEAGLEV-HQFWPLVEI-KCS

CfFz-2 ---GRCE--EITIPMCRN--IG-YNLTAMPNELNHDT-QEEAGLEV-HQFWPLVEI-KCS

PhFz-2 --------------MCRG--IG-YNLTSMPNELNHES-QEEAGLEV-HQFWPLVEI-GCS

RpFz-2 --GPRCE--EITIPMCRG--IG-YNLTSMPNELNHDS-QEEAGLEV-HQFWPLVEI-RCS

TcFz-1 --HDKCK--PITVPFCID--VP-YNSTIFPNLVGHNT-QEDAGYEV-HQYFPLIKI-NCS

ApFz-1 GHHGRCE--PITIPFCMG--IA-YNETIMPNILGQMR-QDEAGFEV-QQYYPLVKI-QCS

AgFz-3 ---NRCE--PITIPFCIG--IP-YNRTIMPNRFGHTK-QDEAALEV-HQYVPLVKI-DCS

DmFz-1 ---NRCE--PITISICKN--IP-YNMTIMPNLIGHTK-QEEAGLEV-HQFAPLVKI-GCS

PhFz-3 ---GKCE--QIKIPFCMG--LQ-YNETIMPNLLGHTK-QEDAGLDV-HQYFPLLKV-KCS

RpFz-1 ---GKCE--PITVPICKK--IQ-YNVTIMPNLLNHNT-QEDAALEV-HQFYPLVKI-DCN

AmFz-2 -HHGRCE--PITINLCMN--IP-YNETIMPNLMNHQK-QEDAGQEV-HQFAPLVKM-KCS

CfFz-1 ---GRCE--PITINLCMN--IA-YNETIMPNLMNHQK-QEDAGQEV-HQYVPLVKM-KCS

NvFz-1 --HGKCE--TITISLCKD--MP-YNETIMPNLLNHQK-QEDAGPEV-HQFSPLVKL-KCS

DpFz-2 --HGRCE--AITIPLCKD--IL-YNETIFPNLLNHQK-QEDAGLEV-HQFYPLVKV-KCS

IsFz-3 ---GRCE--PISIPLCKD--IQ-YNETIMPNLLNHQK-QEDAGMEV-HQFFPLVKV-KCS

SmFz-3 ---GRCE--PITIPLCKD--IQ-YNETIMPNLLNHQK-QEDAGLEV-HQFFPLVKV-QCS

SmFz-7 -ERKQCV--PVGARFCND--VF-YNHTSFPNIFGHKT-RYEVDDFVDQHFSQLITS-GCY

ApFz-3 --PRRCT--PVRLNLCKSV-LE-YNLTSYPNHFGHKN-LDEIHDDL-IAFRDLVDA-ECY

DmFz-5 ---RRCS--PLELSYCRQ--VG-YNITTYPNLLGHAS-YEQLAEDV-IVFRELVDG-ECH

AgFz-5 ----KCE--PLRLGYCRS--VG-YNVTTYPNFFGHGS-LEEVEADL-ISFRELVDA-ECF

PhFz-5 ----QCS--PLSLEYCSK--MS-YNTTSYPNIVGHSN-YFQVLDDV-ISFREIVDA-ECY

RpFz-4 ---RTCA--PMELNYCNR--YV-ANYTSYPNIVGHYN-MEQVMDNV-IIFRELVDS-ECY

NvFz-5 ---RRCS--PIEFSYCKH--LP-YNVTSYPNFFGHND-AREVNDDI-IAFRELVDA-ECY

TcFz-3 --PRKCF--PLQLKYCTK--LP-YNVTTYPNLMGHRN-VIEVKDNV-ITFRELVDA-ECY

AmFz-5 ---RRCT--SLNLSYCKH--LP-YNVTSYPNILGHRS-LADVQEDV-IAFRELVDA-ECY

CfFz-6 ----KCN--KLDLPYCKH--LS-YNISSYPNVLGHRS-LADVEEDV-IAFRELVDA-ECY

*

SmFz-9 SVIRPLLCAVYMPRC-------E--NGS---ID-LP-----SYDLCRITRKRCRIVEVIE

IsFz-5 AVVQPLLCAVYRPKC-------A--DGR---VT-LP-----SQEMCRLVRGFCKIVALGS

ApFz-4 AAIQPLLCALYMPNC-------E--NSS---LH-LP-----PQEMCKLIENHCQILKIEN

RpFz-6 AVIQPFLCALYMPKC-------D--DHG---VY-LP-----SQEMCKIIMGPCRILVSYE

NvFz-6 AVVQPFLCSLFLPKC-------V--NDT---VD-LP-----SQEMCKVVSGPCKILLNHT

AmFz-4 AVVQPLLCSIFMPKC-------I--NDT---VD-LP-----SQEMCKMVSGPCRIVFNHT

CfFz-7 AVVQPFLCSIFMPKC-------V--NNT---VE-LP-----SQEMCRMVSGPCRMLINHT

AgFz-6 AVIQPFLCAVFTPKC-------EKINGQDM-VY-LP-----TLEMCKLTLEPCRILHNTS

TcFz-5 AVIQPFLCALYMPKC-----------ENGK-VD-LP-----SREMCQLTLKPCKMFYNSS

DpFz-4 AVIQPFLCALYMPRC-----------ENGT-VE-LP-----SQEMCKVIRNPCRIVELEH

DpFz-6 PLIEQFVCQAAQPEC-------R--PNDKM-PI-GP-----CRQLCLEVAKACDPHIWSI

SmFz-8 LWLKEYLCYILEPMC-------V--DEV---AV-PP-----CNSLCKAAKKGCEKFITGS

DmFz-7 VRAAEFICGALEPEC-------R--PLHIG-QL-PP-----CRRICKAILEACSIPIYNS

RpFz-5 QRARQFLCTLLEPEC-------Q--PLGSS-VT-PP-----CRNTCKVVAEECSDFIINI

TuFz-4 RRIKQLLCTFLEPPC-------V----EGR-PI-PP-----CKKFCRIALEGCAEYVPAT

DpFz-5 PRVQQYACAVLEPPC----------RGSGI-SL-PP-----CRQFCRAIAEDCSSYVLSA

SmFz-6 QSLAFFLCSLYVPVC-------R--PDYSY-SI-LP-----CNSLCRKILNHCDAAMKEL

TuFz-7 SLLPLFLCSLSTSFC----------NSTSQ-PI-KP-----CRSFCKEALRRCDFFLSVF

DmFz-4 RRARFLLCSSLFPLC-------T--PDVPR-PV-AA-----CKLLCETVRGECMENAPPE

AgFz-4 ELVPLFLCSLFVPKC----------GNSGA-TV-PP-----CKSLCTETMRRCSFFFDVF

DmFz-6 ELVSLFLCTLFVPKC----------GQSGA-TV-PP-----CKTLCTETMRRCGFFFDVF

PhFz-6 ELSALFLCSVFVPKC----------GRQGE-LL-YP-----CKSLCEETKRRCGFFLEVF

BmFz-4 ELTALFLCSLFVPKC----------GPLGH-MV-RP-----CRSLCQETMRRCGFFLEVF

NvFz-4 ELAGLFLCSVFVPKC---------GHKMGR-AV-GP-----CRSLCFETVRRCGFFLDVF

RpFz-3 ELAALFLCSVFVPKC----------GPEGQ-LV-RP-----CKSLCTETKRRCGFFLEVF

TcFz-4 ELSALFLCSLFVPKC----------GPHGE-VV-RP-----CRNLCNETKRRCGFFLDVF

CfFz-5 ELAALFLCSVFVPKC----------GYGGR-VV-FP-----CRSLCHQTKRRCGFFLKVF

AmFz-3 ELAALFLCSVFVPKC----------GSRGH-VV-RP-----CRSLCYHTKRRCGFFLDVF

BmFz-5 LQARFLLCSAFAPLC-------S--EEVSG-SV-SA-----CRALCETVADDCKEQIKI-

IsFz-1 PDTQLFLCSLFSPVC------------LER-PI-FP-----CRSLCEAVRSGCEGTMLRY

DpFz-1 ADTQLFLCSLFSPVC------------LDR-PI-YP-----CRGLCERVRQGCEGRMKTY

SmFz-1 PDTQLFLCSLFSPVC------------LDR-PI-YP-----CRSLCDKVRAGCEGRMRVY

PhFz-4 KHLRQFLCAVLTPMC-------S--EQIKG-AI-PP-----CRGFCERIQGDCQPVIDQF

CfFz-4 RHLRLFLCAVFAPVC-------S--EHVAM-QI-PA-----CKSLCLSVRRDCEPALTSL

NvFz-3 RHLRFFLCSVFAPVC-------S--EHVAM-QI-PA-----CKPLCLSVRRDCENTLKDL

TuFz-3 SQLKFFLCSVYFPMC-------T--EKIVQ-TI-GP-----CRPFCETVRDRCLPILNDF

DmFz-2 SQLKLFLCAAYVPMC-------T--PKAPVHAI-GP-----CRSLCESVRIRCHPVLQGF

AgFz-1 KQLKFFLCATYVPMC-------T--PKVSM-PI-GP-----CRSLCNTVKNRCHPILQGF

TcFz-2 SQLKLFLCSVYVPMC-------T--EKVAN-PI-GP-----CRGLCESVRAKCYPVLKGF

BmFz-2 SQLHLFLCSVYVPMC-------T--DKVAL-PI-GP-----CRGLCDSVHARCFPVLHGF

IsFz-2 SQLKFFLCSVYVPMC-------T--EKVAQ-PI-GP-----CRPLCESVRTRCQPVLQEF

SmFz-5 SQLRLFLCSVYVPMC-------T--DKVPM-PI-GP-----CRSLCEVVRSRCQPVLQEF

CfFz-3 AQLHLFLCSVYAPMC-------T--EKVPA-PI-GP-----CRGLCEQVRARCFPVLQGF

PhFz-1 GQLHFFLCSVYVPMC-------T--EKVAT-PI-GP-----CRGLCESVRSRCFPVLQGF

SmFz-4 KHLKFFLCSLYAPMC-------S--EQVDL-AI-PS-----CQSICEEVKTHCLPILQQF

TuFz-6 PDLKLLLCAVFFPPC----------TILEA-PI-PP-----CRSICLSSKNGCEDVIHRF

BmFz-3 PDLRFFLCSVYAPVC----------TILDS-AI-PP-----CRHLCEAAKQSCDVVIRKF

TuFz-2 DDLRFFLCSMYTPIC-------M--EDYTE-RL-PV-----CRSVCERAQAGCAAIMLQY

TuFz-1 K----FLTT--------------------------------C-SVCERAHAGCAVIMLTY

TuFz-5 DDLKLFLCSMYTPIC-------M--EDYPG-RL-PA-----CRSVCERAHAGCAAIMLTY

DpFz-3 PDMRFFLCSIYAPIC-------I--QDYPT-SI-PA-----CKSVCLRAESGCAPLMRKY

TcFz-6 PDLKFFLCSMYAPIC-------L--PGYKK-PL-PP-----CRGLCKRAREGCEPIMTQY

IsFz-4 DDLKFFLCSLYTPIC-------M--EEYAG-SV-PA-----CRSVCERARAGCAPIMRQY

SmFz-2 PDLKFFLCSMYAPIC-------I--EDYLQ-PL-PA-----CRSVCDRAKAGCAPLMRLY

ApFz-2 ADLKFFLCSVYTPIC-------I--EEYQR-PL-QA-----CRSVCERARDGCLPVMQRY

BmFz-1 SDLKFFLCSVYTPIC-------I--EDYAK-PL-PA-----CRSVCERARAGCAPLMQQY

AgFz-2 PDLKFFLCSMYTPIC-------I--EDYHK-PL-PV-----CRSVCERARAGCAPIMESY

DmFz-3 PDLKFFLCSMYTPIC-------L--EDYHK-PL-PV-----CRSVCERARSGCAPIMQQY

NvFz-2 LDLKFFLCSMYTPIC-------L--PEYSK-PL-PA-----CRSVCERARMGCAPLMHQY

AmFz-1 PDLKFFLCSMYTPIC-------L--PEYTK-PL-PA-----CRSVCERARAGCAPLMQQY

CfFz-2 PDLKFFLCSMYTPIC-------L--PEYTK-PL-PA-----CRSVCERARAGCAPLMQQY

PhFz-2 PDLKFFLCSMYTPIC-------I--EDYHK-PL-PA-----CRSVCERAREGCTPLMLQY

RpFz-2 PDLKFFLCSMYAPIC-------I--EDYHR-PL-PA-----CRSVCERARSGCAPLMQQY

TcFz-1 ADLHLFLCSVFVPVC----------TILEK-PV-PP-----CRSLCLSAKSGCEGIMRKF

ApFz-1 HDLQFFLCSVFAPVC----------TIIEK-PI-PP-----CRSLCVSARNGCEAIMNRF

AgFz-3 PDLKFFLCLLYAPVC----------TILPF-PI-PP-----CRSLCESARA-CESIMKTF

DmFz-1 DDLQLFLCSLYVPVC----------TILER-PI-PP-----CRSLCESARV-CEKLMKTY

PhFz-3 PTVQLFLCSLYFPVC----------TILPN-PL-PP-----CRSLCISARKGCEDLMNQF

RpFz-1 SVLLFFLCSVYVPVC----------TILDR-PI-PP-----CRSLCISARSGCEEVLHKF

AmFz-2 PDLRFFLCTVYAPVC----------TIIDR-AI-PP-----CRSLCESARAGCERLMNSF

CfFz-1 PDLRFFLCTVYAPVC----------TIIEK-AI-PP-----CRSLCESARSGCEGLMNSF

NvFz-1 PDLKFFLCAMYAPVC----------TILEK-AL-PP-----CRSLCESARNGCERLMNNF

DpFz-2 PDLQIFLCSVYAPVC----------TVLDK-PI-PP-----CRSLCLSARSGCEGLMNKF

IsFz-3 PDLQFFLCSMYAPVC----------TILEY-PI-PP-----CRSLCMSARSGCESLMNKF

SmFz-3 PDLQFFLCSMYAPVC----------TILDQ-AI-PP-----CRSLCMSARVGCEGLMNKF

SmFz-7 NHLKHFLCSLLQPSCS------------QHQVI-FP-----CQEFCHAFLTQCHSVFPF-

ApFz-3 QRTLDFVCQLLQPAC-------KPDVKLDRDEITLP-----CKHSCRLFTTGCGHRIPE-

DmFz-5 REAYDFVCRLLQPPC----------DTHGS-DL-QPTPGQICREYCESFMAGCGGRLPQ-

AgFz-5 RQAFDFICRLLQPPC-------EYRSIEEPTAG-TV-----CRQYCQSFWAGCGERLPE-

PhFz-5 RLAYEFVCLILQPPCEKRGGAKE--EEEEDFLV-MP-----CRSYCNEFIKNCGSRISS-

RpFz-4 RLAQEFVCQLLQPPC-------------SQNLI-LP-----CRSFCNEFWEGCGSRLPA-

NvFz-5 KQAYDFICQVLQPAC-------LEGDEEDV-LS-PP-----CRGFCKEFWSGCGSRLPE-

TcFz-3 RHAYDFICQILQPSC-------VSGEGQDE-MI-LP-----CRSFCREFMAGCGARLSE-

AmFz-5 RLAYDFVCQILQPTC-------ISSQPEDL-LQ-LP-----CRSFCREFWNGCGNRLPD-

CfFz-6 PLAYDFICQVLQPAC-------QLSHPEDL-LQ-LP-----CRSFCREFWNGCGNRLSE-

* *

SmFz-9 ---NWPEFL-KCDK---PHFK----HQCKNE--------

IsFz-5 ---EWPSFL-RCQHDIFA-------SGCKN---------

ApFz-4 ---SWPAEF-NCEN--DTIYP---AKLCKND--------

RpFz-6 ---PWPAVF-RCDN--QTRYP----PMCKNDVRELK---

NvFz-6 ---IWPNFI-KCDN--EELFS----RSC-----------

AmFz-4 ---IWPSFV-KCEN--TDLFP------------------

CfFz-7 ---IWPSFA-KCDN--TKLFP----RLC-----------

AgFz-6 ---FFPEFL-KCNETLYPSKCNNDVREEMKFNASGQCQK

TcFz-5 ---IFPKFL-NCDD--ERIFS----SNCKND--------

DpFz-4 RS-GWPDFM-RCED--ADKFP----SGCKN---------

DpFz-6 L--EKEKMMFNCEQ--YAMVD--DLNLCFSS--------

SmFz-8 P--TLEAVF-RCDV--FPSAT--AQILCAGLG-------

DmFz-7 D--VLGELF-DCNL--YPDAH--ESHKCEDPTRRRDYCY

RpFz-5 L--DLSQIF-KCDN--YPDSE----DECIN---------

TuFz-4 L--ELSAAF-DCRR--YPDST--DPSVCVN---------

DpFz-5 L--TLASVL-DCDQ--FPQSN--DPDVCLNL--------

SmFz-6 QL-DWPSRI-DCYY--FPVVD--EYNICSEE--------

TuFz-7 SL-EWPSEI-NCDQ--YPDDP--DPDVCVGYR-------

DmFz-4 LMELWPSFL-NCDG--LPQPE--KHELCMQIPQEV----

AgFz-4 GL-ELPEYL-RCSI--FNDAV-SDQEECVGMA-------

DmFz-6 GL-SLPEYL-NCKL--FKDFP--SSEDCVGL--------

PhFz-6 GLPALPEYLMQCNL--LPDSD--DPDACVGH--------

BmFz-4 GL-SMPDYL-QCEI--FPEST--DTDVCLGNREV-----

NvFz-4 GL-TFPDYL-QCDI--FTEST--DPNVCIGHQE------

RpFz-3 GL-TLPNYL-DCDL--FPESE--DQNVCIGH--------

TcFz-4 GL-TLPEYL-DCSL--FPEKP--DRGHCIGY--------

CfFz-5 GL-SLPDYL-ECEL--FPESS--NPDECIGY--------

AmFz-3 GL-TLPEYL-ECDL--FPENS--NSDECVG---------

BmFz-5 ---LSPTVMLDCSA--FPLRA--NRKLCMRAPN------

IsFz-1 GY-PWPDMV-RCDK--FPVDN----DMCISVQ-------

DpFz-1 GY-PWPDML-RCDK--FPLDN----DMCIGPL-------

SmFz-1 GY-PWPDFL-RCEK--FPLDN----DMCITA--------

PhFz-4 EF-PWPSLL-NCSR--FPVYN----GYCISG--------

CfFz-4 TL-PWPHML-DCDR--FLDRG--RNTLCVQP--------

NvFz-3 TL-PWPHML-DCDR--FPDSG---NTLCVQP--------

TuFz-3 GF-AWPSYM-NCSL--FPAAN-DNQTMCMVG--------

DmFz-2 GF-PWPPAL-DCDK--FPREN-NHETMCMEGFRED----

AgFz-1 GF-PWPSAL-DCNR--FPEEN-NHEHMCMQG--------

TcFz-2 GF-SWPDAL-NCSR--FPVEN-NHEHLCMEGPKD-----

BmFz-2 GF-SWPPEL-DCTL--FPAEN-NHEHMCMEGP-------

IsFz-2 GF-PWPAAL-NCSK--FPPQN-NHRHMCMDGPA------

SmFz-5 GF-PWPSAL-NCSQ--FPPEN-NQHHMCMVGPG------

CfFz-3 GF-PWPAAL-NCSK--FPPEN-NHQHMCMEGP-------

PhFz-1 GF-PWPAAL-DCSK--FPQEN-NHEHMCMEGP-------

SmFz-4 NF-NWPRML-NCSR--LPVPE--MNELCMEFP-------

TuFz-6 GQ-EWPSYL-DCDK--FPDV-----EPCVS---------

BmFz-3 DF-PWPPEL-ECSE--FPEVS--DNNICV----------

TuFz-2 GF-PWPESM-DCNN--FPVFG-SQEQLCMDH--------

TuFz-1 GF-PWPESM-DCNN--FPVYPGAPEQLCMDQ--------

TuFz-5 GF-PWPESM-DCNN--FPVYPGTPEQLCMDQK-------

DpFz-3 GF-AWPDRM-QCDK--FPNFG-DPQNLCMDARN------

TcFz-6 GF-KWPERM-DCEQ--FPVYGASPDQLCMD---------

IsFz-4 GF-AWPERM-NCDA--LPQYG-DQEQLCMDAKE------

SmFz-2 GF-AWPERM-NCDR--LPEYG-DPSQLCMDSK-------

ApFz-2 GF-VWPEKM-QCDK--LPVHG--GPELCMAQD-------

BmFz-1 GF-PWPERM-ACDA---------AECECACRPPLLAA--

AgFz-2 SF-NWPERM-ACEN--LPVSG-DSDNLCMEMP-------

DmFz-3 SF-EWPERM-ACEH--LPLHG-DPDNLCMEQPSYTEA--

NvFz-2 GF-SWPERM-ACER--LPNQG-DPENLCMEQD-------

AmFz-1 GF-SWPERM-ACER--LPAHG-DPENLCMEQDNH-----

CfFz-2 GF-SWPERM-ACER--LPNHGEDPENLCMEQD-------

PhFz-2 GF-PWPERM-ACER--FPIHDSDPENLCMEQ--------

RpFz-2 GF-QWPDRM-ACEK--LPVHG-DPDNLCMEM--------

TcFz-1 GY-NWPENL-DCNQ--YPENS----NLCVEKHNI-----

ApFz-1 QI-EWPDNL-ECNQ--FPENG----QLCVGEN-------

AgFz-3 NF-PWPENL-ECSQ--FPEYG--GEELCVSKH-------

DmFz-1 NF-NWPENL-ECSK--FPVHG--GEDLCVAE--------

PhFz-3 GF-FWPDKF-ECDN--FPEVN-QSQS-------------

RpFz-1 GF-LWPENL-ECDR--FPDPP----DICV----------

AmFz-2 GF-AWPDNL-DCSK--LPENG--GPELCVGHNET-----

CfFz-1 GF-AWPEAL-DCSR--MPEND--GTELCVAT--------

NvFz-1 GF-YWPESM-ECSK--FPENR--DGVLCVGKN-------

DpFz-2 GF-QWPESL-ECSR--YPEGG-AAGELCVGENN------

IsFz-3 GF-KWPESL-ECDK--FPEVG--SEKLCVGEN-------

SmFz-3 GF-QWPESL-DCSK--FPEAG--SHDICVGE--------

SmFz-7 ---ILSRFL-HCNN--YPSIK-NTAEKCLAEP-------

ApFz-3 ---KLLAGF-DCAK--LPDYS-NIGSTCTSK--------

DmFz-5 ---RFRQFF-DCER--FPEST--GTQSCHQKPHCVSD--

AgFz-5 ---RLRRYL-DCER--FPEST--GVQSCHS---------

PhFz-5 ---RFMNFL-DCTK--FSEFS--DTGICV----------

RpFz-4 ---NLQDYF-NCSQ--FPEYS-AEGPPCLP---------

NvFz-5 ---RLKAAL-DCAK--FPEYA--DEGSCRS---------

TcFz-3 ---KMKESL-DCSQ--FPEYSCAAKPGCVE---------

AmFz-5 ---KFKPLL-DCSN--FPEYV--DQGGCRAKPGCVQ---

CfFz-6 ---KIKRAL-DCSN--FPEYV--GPGSCR----------

*
