# Supplementary material for: Insights into the molecular evolution of peptidase inhibitors in arthropods
Source: PLoS One. 2017 Nov 6;12(11):e0187643. doi: 10.1371/journal.pone.0187643 (PMC5673224; doi:10.1371/journal.pone.0187643)
Supplement: S3 Fig — (DOCX) [file pone.0187643.s003.docx]

**S3 Fig.** Information about accession numbers/gene models corresponding to the peptidase inhibitor families used in this study. Ag, *Anopheles gambiae*; Am, *Apis mellifera*; Ap, *Acyrtosiphon pisum*; Bm, *Bombyx mori*; Cf, *Camponotus floridanus*; Dm, *Drosophila melanogaster*; Dp, *Daphnia pulex*; Is, *Ixodes scapularis*; Nv, *Nasonia vitripennis*; Ph, *Pediculus humanus*; Rp, *Rhodnius prolixus*; Sm, *Strigamia maritima*; Tc, *Tribolium castaneum*; Tu, *Tetranychus urticae*.

**A. I1 Kazal sequences.**

| **Protein** | **Organism** | **Accession number/Gene model** |
| --- | --- | --- |
| ApKaz-1 | *Acyrthosiphon pisum* | XP_001945453.1 |
| ApKaz-2 | *Acyrthosiphon pisum* | XP_008185835.1 |
| ApKaz-3 | *Acyrthosiphon pisum* | XP_008181786.1 |
| ApKaz-4 | *Acyrthosiphon pisum* | XP_001942581.2 |
| ApKaz-5 | *Acyrthosiphon pisum* | XP_003242765.1 |
| ApKaz-6 | *Acyrthosiphon pisum* | XP_003242292.2 |
| ApKaz-7 | *Acyrthosiphon pisum* | XP_001943921.2 |
| ApKaz-8 | *Acyrthosiphon pisum* | XP_001945396.1 |
| ApKaz-9 | *Acyrthosiphon pisum* | XP_008180872.1 |
| DmKaz-1 | *Drosophila melanogaster* | ACL85205.1 |
| DmKaz-2 | *Drosophila melanogaster* | ACL85205.1 |
| DmKaz-3 | *Drosophila melanogaster* | AAM71054.2 |
| DmKaz-4 | *Drosophila melanogaster* | AGB92932.1 |
| DmKaz-5 | *Drosophila melanogaster* | ACT79358.1 |
| DmKaz-6 | *Drosophila melanogaster* | AHN54364.1 |
| DmKaz-7 | *Drosophila melanogaster* | AAV59053.1 |
| DmKaz-8 | *Drosophila melanogaster* | AHN54365.1 |
| DmKaz-9 | *Drosophila melanogaster* | AAY55418.1 |
| DmKaz-10 | *Drosophila melanogaster* | AHN54363.1 |
| DmKaz-11 | *Drosophila melanogaster* | AGB94527.1 |
| DmKaz-12 | *Drosophila melanogaster* | AAF48217.2 |
| DpKaz-1 | *Daphnia pulex* | EFX77037.1 |
| DpKaz-2 | *Daphnia pulex* | EFX89193.1 |
| DpKaz-3 | *Daphnia pulex* | EFX89486.1 |
| DpKaz-4 | *Daphnia pulex* | EFX72774.1 |
| DpKaz-5 | *Daphnia pulex* | EFX72773.1 |
| DpKaz-6 | *Daphnia pulex* | EFX89192.1 |
| DpKaz-7 | *Daphnia pulex* | EFX89772.1 |
| SmKaz-1 | *Strigamia maritima* | SMAR012874 |
| SmKaz-2 | *Strigamia maritima* | SMAR005837 |
| SmKaz-3 | *Strigamia maritima* | SMAR000933 |
| SmKaz-4 | *Strigamia maritima* | SMAR013380 |
| SmKaz-5 | *Strigamia maritima* | SMAR002860 |
| SmKaz-6 | *Strigamia maritima* | [SMAR0](http://metazoa.ensembl.org/Strigamia_maritima/Gene/Summary?db=core;g=SMAR012874;r=JH432198:12730-30686;t=SMAR012874-RA;tl=uWkw0HOny1pkMwqY-274747-1115163)02968 |
| SmKaz-7 | *Strigamia maritima* | SMAR013711 |
| SmKaz-8 | *Strigamia maritima* | SMAR014322 |
| SmKaz-9 | *Strigamia maritima* | SMAR004004 |
| SmKaz-10 | *Strigamia maritima* | SMAR003269 |
| TuKaz-1 | *Tetranychus urticae* | tetur22g02190 |
| TuKaz-2 | *Tetranychus urticae* | tetur36g00270 |
| TuKaz-3 | *Tetranychus urticae* | tetur08g07590 |
| TuKaz-4 | *Tetranychus urticae* | tetur11g06220 |
| TuKaz-5 | *Tetranychus urticae* | tetur10g01770 |
| TuKaz-6 | *Tetranychus urticae* | tetur03g05500 |
| TuKaz-7 | *Tetranychus urticae* | tetur43g00220 |
| TuKaz-8 | *Tetranychus urticae* | tetur05g04370 |

**B. I2 Kunitz-A sequences.**

| **Protein** | **Organism** | **Accession number/Gene model** |
| --- | --- | --- |
| ApKuz-1 | *Acyrthosiphon pisum* | XP_008188452.1 |
| ApKuz-2 | *Acyrthosiphon pisum* | XP_008189853.1 |
| ApKuz-3 | *Acyrthosiphon pisum* | XP_003247303.1 |
| ApKuz-4 | *Acyrthosiphon pisum* | XP_001945053.2 |
| DmKuz-1 | *Drosophila melanogaster* | FBpp0087737 |
| DmKuz-2 | *Drosophila melanogaster* | FBpp0306588 |
| DmKuz-3 | *Drosophila melanogaster* | FBpp0289145 |
| DmKuz-4 | *Drosophila melanogaster* | FBpp0087006 |
| DmKuz-5 | *Drosophila melanogaster* | FBpp0291051 |
| DmKuz-6 | *Drosophila melanogaster* | FBpp0293107 |
| DmKuz-7 | *Drosophila melanogaster* | FBpp0289151 |
| DmKuz-8 | *Drosophila melanogaster* | FBpp0110063 |
| DmKuz-9 | *Drosophila melanogaster* | FBpp0111385 |
| DmKuz-10 | *Drosophila melanogaster* | FBpp0289528 |
| DmKuz-11 | *Drosophila melanogaster* | FBpp0292841 |
| DmKuz-12 | *Drosophila melanogaster* | FBpp0292842 |
| DmKuz-13 | *Drosophila melanogaster* | FBpp0305768 |
| DmKuz-14 | *Drosophila melanogaster* | FBpp0086129 |
| DmKuz-15 | *Drosophila melanogaster* | FBpp0077245 |
| DmKuz-16 | *Drosophila melanogaster* | FBpp0077238 |
| DmKuz-17 | *Drosophila melanogaster* | FBpp0077236 |
| DmKuz-18 | *Drosophila melanogaster* | FBpp0077235 |
| DmKuz-19 | *Drosophila melanogaster* | FBpp0077234 |
| DmKuz-20 | *Drosophila melanogaster* | FBpp0077233 |
| DmKuz-21 | *Drosophila melanogaster* | FBpp0289527 |
| DpKuz-1 | *Daphnia pulex* | EFX84732.1 |
| DpKuz-2 | *Daphnia pulex* | EFX79782.1 |
| DpKuz-3 | *Daphnia pulex* | EFX81066.1 |
| DpKuz-4 | *Daphnia pulex* | EFX80872.1 |
| DpKuz-5 | *Daphnia pulex* | EFX81054.1 |
| DpKuz-6 | *Daphnia pulex* | EFX79924.1 |
| DpKuz-7 | *Daphnia pulex* | EFX66312.1 |
| DpKuz-8 | *Daphnia pulex* | EFX81245.1 |
| DpKuz-9 | *Daphnia pulex* | EFX90099.1 |
| DpKuz-10 | *Daphnia pulex* | EFX67760.1 |
| DpKuz-11 | *Daphnia pulex* | EFX65648.1 |
| DpKuz-12 | *Daphnia pulex* | EFX81065.1 |
| DpKuz-13 | *Daphnia pulex* | EFX87486.1 |
| DpKuz-14 | *Daphnia pulex* | EFX77761.1 |
| DpKuz-15 | *Daphnia pulex* | EFX81063.1 |
| DpKuz-16 | *Daphnia pulex* | EFX79925.1 |
| DpKuz-17 | *Daphnia pulex* | EFX82505.1 |
| DpKuz-18 | *Daphnia pulex* | EFX81062.1 |
| DpKuz-19 | *Daphnia pulex* | EFX61998.1 |
| DpKuz-20 | *Daphnia pulex* | EFX63852.1 |
| DpKuz-21 | *Daphnia pulex* | EFX65647.1 |
| DpKuz-22 | *Daphnia pulex* | EFX81064.1 |
| SmKuz-1 | *Strigamia maritima* | SMAR012896 |
| SmKuz-2 | *Strigamia maritima* | SMAR012402 |
| SmKuz-3 | *Strigamia maritima* | SMAR012895 |
| SmKuz-4 | *Strigamia maritima* | SMAR008271 |
| SmKuz-5 | *Strigamia maritima* | SMAR11117 |
| SmKuz-6 | *Strigamia maritima* | SMAR08155 |
| TuKuz-1 | *Tetranychus urticae* | tetur05g06950 |
| TuKuz-2 | *Tetranychus urticae* | tetur01g03690 |
| TuKuz-3 | *Tetranychus urticae* | tetur04g02080 |
| TuKuz-4 | *Tetranychus urticae* | tetur13g03820 |
| TuKuz-5 | *Tetranychus urticae* | tetur01g00790 |

**C. I4 Serpin sequences.**

| **Protein** | **Organism** | **Accession number/Gene model** |
| --- | --- | --- |
| ApSRP-1 | *Acyrthosiphon pisum* | XP_008178942.1 |
| ApSRP-2 | *Acyrthosiphon pisum* | XP_008178957.1 |
| ApSRP-3 | *Acyrthosiphon pisum* | XP_001952220.2 |
| ApSRP-4 | *Acyrthosiphon pisum* | XP_001951580.2 |
| ApSRP-5 | *Acyrthosiphon pisum* | NP_001156567.1 |
| ApSRP-6 | *Acyrthosiphon pisum* | XP_001950205.1 |
| ApSRP-7 | *Acyrthosiphon pisum* | NP_001153315.1 |
| ApSRP-8 | *Acyrthosiphon pisum* | XP_001946416.1 |
| ApSRP-9 | *Acyrthosiphon pisum* | XP_008179044.1 |
| ApSRP-10 | *Acyrthosiphon pisum* | XP_001944367.1 |
| ApSRP-11 | *Acyrthosiphon pisum* | XP_001943519.1 |
| ApSRP-12 | *Acyrthosiphon pisum* | XP_001950393.1 |
| ApSRP-13 | *Acyrthosiphon pisum* | XP_008190234.1 |
| ApSRP-14 | *Acyrthosiphon pisum* | XP_001950277.2 |
| ApSRP-15 | *Acyrthosiphon pisum* | NP_001156102.2 |
| DmSRP-1 | *Drosophila melanogaster* | FBpp0088123 |
| DmSRP-2 | *Drosophila melanogaster* | FBpp0079171 |
| DmSRP-3 | *Drosophila melanogaster* | FBpp0079528 |
| DmSRP-4 | *Drosophila melanogaster* | FBpp0289587 |
| DmSRP-5 | *Drosophila melanogaster* | FBpp0085495 |
| DmSRP-6 | *Drosophila melanogaster* | FBpp0085497 |
| DmSRP-7 | *Drosophila melanogaster* | FBpp00873076 |
| DmSRP-8 | *Drosophila melanogaster* | FBpp0110074 |
| DmSRP-9 | *Drosophila melanogaster* | FBpp0077830 |
| DmSRP-10 | *Drosophila melanogaster* | FBpp0077830 |
| DmSRP-11 | *Drosophila melanogaster* | FBpp0082594 |
| DmSRP-12 | *Drosophila melanogaster* | FBpp0088124 |
| DmSRP-13 | *Drosophila melanogaster* | FBpp0110138 |
| DmSRP-14 | *Drosophila melanogaster* | FBpp0110068 |
| DmSRP-15 | *Drosophila melanogaster* | FBpp0079179 |
| DmSRP-16 | *Drosophila melanogaster* | FBpp0079094 |
| DmSRP-17 | *Drosophila melanogaster* | FBpp0074610 |
| DmSRP-18 | *Drosophila melanogaster* | FBpp0089190 |
| DmSRP-19 | *Drosophila melanogaster* | FBpp0099762 |
| DmSRP-20 | *Drosophila melanogaster* | FBpp0088112 |
| DmSRP-21 | *Drosophila melanogaster* | FBpp0085912 |
| DmSRP-22 | *Drosophila melanogaster* | FBpp0082595 |
| DmSRP-23 | *Drosophila melanogaster* | FBpp0080979 |
| DmSRP-24 | *Drosophila melanogaster* | FBpp0289586 |
| DmSRP-25 | *Drosophila melanogaster* | FBpp0078950 |
| DmSRP-26 | *Drosophila melanogaster* | FBpp0298308 |
| DpSRP-1 | *Daphnia pulex* | EFX75020.1 |
| DpSRP-2 | *Daphnia pulex* | EFX68536.1 |
| DpSRP-3 | *Daphnia pulex* | EFX66670.1 |
| DpSRP-4 | *Daphnia pulex* | EFX68557.1 |
| DpSRP-5 | *Daphnia pulex* | EFX80056.1 |
| SmSRP-1 | *Strigamia maritima* | SMAR013388 |
| SmSRP-2 | *Strigamia maritima* | SMAR003087 |
| SmSRP-3 | *Strigamia maritima* | SMAR003048 |
| SmSRP-4 | *Strigamia maritima* | SMAR003045 |
| SmSRP-5 | *Strigamia maritima* | SMAR005784 |
| SmSRP-6 | *Strigamia maritima* | SMAR001099 |
| SmSRP-7 | *Strigamia maritima* | SMAR000663 |
| SmSRP-8 | *Strigamia maritima* | SMAR004057 |
| TuSRP-1 | *Tetranychus urticae* | tetur01g02260 |
| TuSRP-2 | *Tetranychus urticae* | tetur18g03300 |
| TuSRP-3 | *Tetranychus urticae* | tetur20g01980 |
| TuSRP-4 | *Tetranychus urticae* | tetur05g01350 |
| TuSRP-5 | *Tetranychus urticae* | tetur128g00050 |
| TuSRP-6 | *Tetranychus urticae* | tetur10g04760 |
| TuSRP-7 | *Tetranychus urticae* | tetur08g03770 |
| TuSRP-8 | *Tetranychus urticae* | tetur02g03240 |
| TuSRP-9 | *Tetranychus urticae* | tetur08g04280 |
| TuSRP-10 | *Tetranychus urticae* | tetur08g04290 |
| TuSRP-11 | *Tetranychus urticae* | tetur30g02180 |
| TuSRP-12 | *Tetranychus urticae* | tetur10g04810 |
| TuSRP-13 | *Tetranychus urticae* | tetur128g00060 |
| TuSRP-14 | *Tetranychus urticae* | tetur10g04760 |
| TuSRP-15 | *Tetranychus urticae* | tetur10g04780 |
| TuSRP-16 | *Tetranychus urticae* | tetur10g04790 |

**D. I8 Ascaris sequences.**

| **Protein** | **Organism** | **Accession number/Gene model** |
| --- | --- | --- |
| ApTLI-1 | *Acyrthosiphon pisum* | XP_001952865.2 |
| DmTLI-1 | *Drosophila melanogaster* | NP_001245459.1 |
| DmTLI-2 | *Drosophila melanogaster* | NP_996090.1 |
| DmTLI-3 | *Drosophila melanogaster* | NP_524060.2 |
| DmTLI-4 | *Drosophila melanogaster* | NP_523892.1 |
| DmTLI-5 | *Drosophila melanogaster* | NP_001097347.1 |
| DpTLI-1 | *Daphnia pulex* | EFX89583.1 |
| SmTLI-1 | *Strigamia maritima* | SMAR010682 |
| SmTLI-2 | *Strigamia maritima* | SMAR002832 |
| TuTLI-1 | *Tetranychus urticae* | tetur22g00250 |
| TuTLI-2 | *Tetranychus urticae* | tetur32g00620 |
| TuTLI-3 | *Tetranychus urticae* | tetur40g00391 |
| TuTLI-4 | *Tetranychus urticae* | tetur03g09900 |
| TuTLI-5 | *Tetranychus urticae* | tetur40g00200 |
| TuTLI-6 | *Tetranychus urticae* | tetur22g00290 |
| TuTLI-7 | *Tetranychus urticae* | tetur22g00260 |
| TuTLI-8 | *Tetranychus urticae* | tetur40g00392 |
| TuTLI-9 | *Tetranychus urticae* | tetur95g00090 |

**E. I17 WAP (Whey Acid Protein) sequences.**

| **Protein** | **Organism** | **Accession number/Gene model** |
| --- | --- | --- |
| AgWAP-1 | *Anopheles gambiae* | AGAP011460 |
| AgWAP-2 | *Anopheles gambiae* | AGAP004333 |
| AgWAP-3 | *Anopheles gambiae* | AGAP003441 |
| AmWAP-1 | *Apis mellifera* | XP_006570011.1 |
| AmWAP-2 | *Apis mellifera* | XP_006570013.1 |
| AmWAP-3 | *Apis mellifera* | XP_006564337.1 |
| AmWAP-4 | *Apis mellifera* | XP_001120452.2 |
| ApWAP-1 | *Acyrthosiphon pisum* | XP_008182111.1 |
| BmWAP-1 | *Bombyx mori* | NP_001037043.1 |
| BmWAP-2 | *Bombyx mori* | XP_004921896.1 |
| BmWAP-3 | *Bombyx mori* | XP_004922907.1 |
| BmWAP-4 | *Bombyx mori* | NP_001040277.1 |
| CfWAP-1 | *Camponotus floridanus* | CFLO11805 |
| CfWAP-2 | *Camponotus floridanus* | CFLO20294 |
| CfWAP-3 | *Camponotus floridanus* | CFLO11796 |
| DmWAP-1 | *Drosophila melanogaster* | NP_651570.1 |
| DmWAP-2 | *Drosophila melanogaster* | NP_651220.3 |
| DpWAP-1 | *Daphnia pulex* | EFX61998.1 |
| NvWAP-1 | *Nasonia vitripennis* | sgp2_SCAFFOLD4_379 |
| PhWAP-1 | *Pediculus humanus* | PHUM580230 |
| RpWAP-1 | *Rhodnius prolixus* | RPRC004631 |
| RpWAP-2 | *Rhodnius prolixus* | RPRC007846 |
| SmWAP-1 | *Strigamia maritima* | SMAR013036 |
| SmWAP-2 | *Strigamia maritima* | SMAR012562 |
| SmWAP-3 | *Strigamia maritima* | SMAR007726 |
| SmWAP-4 | *Strigamia maritima* | SMAR007963 |
| SmWAP-5 | *Strigamia maritima* | SMAR007726 |
| SmWAP-6 | *Strigamia maritima* | SMAR001439 |
| SmWAP-7 | *Strigamia maritima* | SMAR007961 |
| TcWAP-1 | *Tribolium castaneum* | XP_969824.1 |
| TcWAP-2 | *Tribolium castaneum* | XP_008196249.1 |
| TcWAP-3 | *Tribolium castaneum* | XP_008198656.1 |
| TcWAP-4 | *Tribolium castaneum* | XP_008201369.1 |
| TcWAP-5 | *Tribolium castaneum* | XP_969756.1 |

**F. I19 Pacifastin sequences.**

| **Protein** | **Organism** | **Accession number/Gene model** |
| --- | --- | --- |
| AgPac-1 | *Anopheles gambiae* | AGAP011319 |
| AmPac-1 | *Apis mellifera* | XP_003250953.1 |
| BmPac-1 | *Bombyx mori* | XP_004927292.1 |
| BmPac-2 | *Bombyx mori* | XP_004927278.1 |
| BmPac-3 | *Bombyx mori* | XP_004927294.1 |
| BmPac-4 | *Bombyx mori* | XP_004927552.1 |
| CfPac-1 | *Camponotus floridanus* | CFLO15516 |
| NvPac-1 | *Nasonia vitripennis* | NV14516 |
| NvPac-2 | *Nasonia vitripennis* | NV14517 |
| NvPac-3 | *Nasonia vitripennis* | NV30509 |
| NvPac-4 | *Nasonia vitripennis* | NV20863 |
| NvPac-5 | *Nasonia vitripennis* | NV16044 |
| NvPac-6 | *Nasonia vitripennis* | NV16045 |
| NvPac-7 | *Nasonia vitripennis* | NV16007 |
| NvPac-8 | *Nasonia vitripennis* | NV21832 |
| NvPac-9 | *Nasonia vitripennis* | NV21693 |
| NvPac-10 | *Nasonia vitripennis* | NV10307 |
| NvPac-11 | *Nasonia vitripennis* | NV21693 |
| NvPac-12 | *Nasonia vitripennis* | NV10308 |
| PhPac-1 | *Pediculus humanus* | PHUM595790 |
| PhPac-2 | *Pediculus humanus* | PHUM595810 |
| PhPac-3 | *Pediculus humanus* | PHUM595800 |
| RpPac-1 | *Rhodnius prolixus* | RPRC009857 |
| RpPac-2 | *Rhodnius prolixus* | RPRC010392 |
| RpPac-3 | *Rhodnius prolixus* | RPRC010179 |
| RpPac-4 | *Rhodnius prolixus* | RPRC015341 |
| RpPac-5 | *Rhodnius prolixus* | RPRC015340 |
| RpPac-6 | *Rhodnius prolixus* | RPRC012510 |
| TcPac-1 | *Tribolium castaneum* | XP_001812139.2 |
| TcPac-2 | *Tribolium castaneum* | XP_008200141.1 |

**G. I21 7B2 family sequences.**

| **Protein** | **Organism** | **Accession number/Gene model** |
| --- | --- | --- |
| Ag7B2-1 | *Anopheles gambiae* | AGAP008082 |
| Am7B2-1 | *Apis mellifera* | XP_006562605.1 |
| Ap7B2-1 | *Acyrthosiphon pisum* | XP_008190028.1 |
| Bm7B2-1 | *Bombyx mori* | XP_004926576.1 |
| Cf7B2-1 | *Camponotus floridanus* | CFLO13825 |
| Dm7B2-1 | *Drosophila melanogaster* | NP_620471.3 |
| Dp7B2-1 | *Daphnia pulex* | EFX89982.1 |
| Is7B2-1 | *Ixodes scapularis* | ISCW008663 |
| Nv7B2-1 | *Nasonia vitripennis* | NV15958 |
| Ph7B2-1 | *Pediculus humanus* | PHUM611520 |
| Rp7B2-1 | *Rhodnius prolixus* | RPRC015183 |
| Sm7B2-1 | *Strigamia maritima* | SMAR003050 |
| Tc7B2-1 | *Tribolium castaneum* | XP_973692.1 |
| Tu7B2-1 | *Tetranychus urticae* | tetur25g00440 |

**H. I25 Cystatin sequences. Accession number/Gene model**s are in Santamaria et al. 2012

| **Protein** | **Organism** | **Accession number/Gene model** |
| --- | --- | --- |
| SmCPI-1 | *Strigamia maritima* | SMAR014607 |
| SmCPI-2 | *Strigamia maritima* | SMAR009040 |

**H. I31 Thyropin sequences. Accession number/Gene model**s are in Santamaria et al. 2012

| **Protein** | **Organism** | **Accession number/Gene model** |
| --- | --- | --- |
| SmTyr-1 | *Strigamia maritima* | SMAR012402 |
| SmTyr-2 | *Strigamia maritima* | SMAR006623 |
| SmTyr-3 | *Strigamia maritima* | SMAR014997 |
| SmTyr-4 | *Strigamia maritima* | SMAR006622 |
| SmTyr-5 | *Strigamia maritima* | SMAR006621 |
| SmTyr-6 | *Strigamia maritima* | SMAR005274 |
| SmTyr-7 | *Strigamia maritima* | SMAR014572 |

**H. I32 IAP family sequences.**

| **Protein** | **Organism** | **Accession number/Gene model** |
| --- | --- | --- |
| AgBIR-1 | *Anopheles gambiae* | AGAP007294 |
| AgBIR-2 | *Anopheles gambiae* | AGAP007292 |
| AgBIR-3 | *Anopheles gambiae* | AGAP011326 |
| AgBIR-4 | *Anopheles gambiae* | AGAP007293 |
| AgBIR-5 | *Anopheles gambiae* | AGAP007291 |
| AgBIR-6 | *Anopheles gambiae* | AGAP013034 |
| AgBIR-7 | *Anopheles gambiae* | AGAP002651 |
| AgBIR-8 | *Anopheles gambiae* | AGAP008420 |
| ApBIR-1 | *Acyrthosiphon pisum* | NP_001156583.1 |
| ApBIR-2 | *Acyrthosiphon pisum* | BAH72482.1 |
| ApBIR-3 | *Acyrthosiphon pisum* | XP_008184238.1 |
| ApBIR-4 | *Acyrthosiphon pisum* | XP_001947156.2 |
| ApBIR-5 | *Acyrthosiphon pisum* | XP_001947156.2 |
| ApBIR-6 | *Acyrthosiphon pisum* | XP_003246156.1 |
| ApBIR-7 | *Acyrthosiphon pisum* | XP_008183320.1 |
| ApBIR-8 | *Acyrthosiphon pisum* | XP_008189690.1 |
| ApBIR-9 | *Acyrthosiphon pisum* | XP_008187480.1 |
| ApBIR-10 | *Acyrthosiphon pisum* | XP_003246599.2 |
| BmBIR-1 | *Bombyx mori* | ADM32525.1 |
| BmBIR-2 | *Bombyx mori* | ADM32523.1 |
| BmBIR-3 | *Bombyx mori* | ADM32524.1 |
| BmBIR-4 | *Bombyx mori* | AAN46650.1 |
| CfBIR-1 | *Camponotus floridanus* | CFLO17518 |
| CfBIR-2 | *Camponotus floridanus* | CFLO17515 |
| CfBIR-3 | *Camponotus floridanus* | CFLO11336 |
| CfBIR-4 | *Camponotus floridanus* | CFLO17517 |
| CfBIR-5 | *Camponotus floridanus* | CFLO17516 |
| CfBIR-6 | *Camponotus floridanus* | CFLO15321 |
| DmBIR-1 | *Drosophila melanogaster* | ACL91414.1 |
| DmBIR-2 | *Drosophila melanogaster* | AGB95844.1 |
| DmBIR-3 | *Drosophila melanogaster* | AAC47155.1 |
| DmBIR-4 | *Drosophila melanogaster* | 2205254A |
| DpBIR-1 | *Daphnia pulex* | EFX66487.1 |
| DpBIR-2 | *Daphnia pulex* | EFX73046.1 |
| DpBIR-3 | *Daphnia pulex* | EFX88533.1 |
| DpBIR-4 | *Daphnia pulex* | EFX87271.1 |
| DpBIR-5 | *Daphnia pulex* | EFX81544.1 |
| IsBIR-1 | *Ixodes scapularis* | ISCW012508 |
| IsBIR-2 | *Ixodes scapularis* | ISCW003136 |
| IsBIR-3 | *Ixodes scapularis* | ISCW004187 |
| IsBIR-4 | *Ixodes scapularis* | ISCW010694 |
| IsBIR-5 | *Ixodes scapularis* | ISCW015499 |
| NvBIR-1 | *Nasonia vitripennis* | NV10358 |
| NvBIR-2 | *Nasonia vitripennis* | NV14467 |
| NvBIR-3 | *Nasonia vitripennis* | NV50742 |
| NvBIR-4 | *Nasonia vitripennis* | NV18397 |
| NvBIR-5 | *Nasonia vitripennis* | NV14469 |
| NvBIR-6 | *Nasonia vitripennis* | NV26183 |
| NvBIR-7 | *Nasonia vitripennis* | NV18809 |
| NvBIR-8 | *Nasonia vitripennis* | NV22471 |
| NvBIR-9 | *Nasonia vitripennis* | NV30942 |
| NvBIR-10 | *Nasonia vitripennis* | NV31603 |
| NvBIR-11 | *Nasonia vitripennis* | NV23587 |
| PhBIR-1 | *Pediculus humanus* | PHUM418030 |
| PhBIR-2 | *Pediculus humanus* | PHUM253740 |
| PhBIR-3 | *Pediculus humanus* | PHUM080100 |
| RpBIR-1 | *Rhodnius prolixus* | RPRC001809 |
| RpBIR-2 | *Rhodnius prolixus* | RPRC011720 |
| RpBIR-3 | *Rhodnius prolixus* | RPRC007068 |
| SmBIR-1 | *Strigamia maritima* | SMAR007518 |
| SmBIR-2 | *Strigamia maritima* | SMAR003040 |
| SmBIR-3 | *Strigamia maritima* | SMAR002085 |
| SmBIR-4 | *Strigamia maritima* | SMAR004880 |
| SmBIR-5 | *Strigamia maritima* | SMAR014324 |
| SmBIR-6 | *Strigamia maritima* | SMAR015763 |
| SmBIR-7 | *Strigamia maritima* | SMAR000969 |
| TcBIR-1 | *Tribolium castaneum* | EFA01269.1 |
| TcBIR-2 | *Tribolium castaneum* | XP_008195667.1 |
| TcBIR-3 | *Tribolium castaneum* | XP_008201643.1 |
| TcBIR-4 | *Tribolium castaneum* | AEQ93553.1 |
| TuBIR-1 | *Tetranychus urticae* | tetur07g05870 |
| TuBIR-2 | *Tetranychus urticae* | tetur03g04890 |
| TuBIR-3 | *Tetranychus urticae* | tetur06g01110 |
| TuBIR-4 | *Tetranychus urticae* | tetur03g07570 |
| TuBIR-5 | *Tetranychus urticae* | tetur58g00080 |
| TuBIR-6 | *Tetranychus urticae* | tetur45g00130 |
| TuBIR-7 | *Tetranychus urticae* | tetur03g09840 |
| TuBIR-8 | *Tetranychus urticae* | tetur03g03840 |
| TuBIR-9 | *Tetranychus urticae* | tetur06g03410 |
| TuBIR-10 | *Tetranychus urticae* | tetur08g02350 |
| TuBIR-11 | *Tetranychus urticae* | tetur39g00770 |
| TuBIR-12 | *Tetranychus urticae* | tetur01g12100 |
| TuBIR-13 | *Tetranychus urticae* | tetur03g09850 |
| TuBIR-14 | *Tetranychus urticae* | tetur12g01630 |
| TuBIR-15 | *Tetranychus urticae* | tetur08g00890 |
| TuBIR-16 | *Tetranychus urticae* | tetur07g02900 |

**G. I35 Timp family sequences.**

| **Protein** | **Organism** | **Accession number/Gene model** |
| --- | --- | --- |
| AgTIMP-1 | *Anopheles gambiae* | AGAP003319 |
| AmTIMP-1 | *Apis mellifera* | XP_006562313.1 |
| BmTIMP-1 | *Bombyx mori* | XP_004931914.1 |
| CfTIMP-1 | *Camponotus floridanus* | CFLO13407 |
| DmTIMP-1 | *Drosophila melanogaster* | CAA08989.1 |
| IsTIMP-1 | *Ixodes scapularis* | ISCW005740 |
| SmTIMP-1 | *Strigamia maritima* | [SMAR0](http://metazoa.ensembl.org/Strigamia_maritima/Gene/Summary?db=core;g=SMAR012874;r=JH432198:12730-30686;t=SMAR012874-RA;tl=uWkw0HOny1pkMwqY-274747-1115163)15495 |
| TcTIMP-1 | *Tribolium castaneum* | EFA12156.1 |
| TuTIMP-1 | *Tetranychus urticae* | tetur28g00190 |

**I. I51 Serine Carboxypeptidase Y inhibitor sequences.**

| **Protein** | **Organism** | **Accession number/Gene model** |
| --- | --- | --- |
| AgSCPYI-1 | *Anopheles gambiae* | AGAP012962 |
| AgSCPYI-2 | *Anopheles gambiae* | AGAP002049 |
| AgSCPYI-3 | *Anopheles gambiae* | AGAP013036 |
| AgSCPYI-4 | *Anopheles gambiae* | AGAP008909 |
| AgSCPYI-5 | *Anopheles gambiae* | AGAP013427 |
| AgSCPYI-6 | *Anopheles gambiae* | AGAP003580 |
| AgSCPYI-7 | *Anopheles gambiae* | AGAP000652 |
| AmSCPYI-1 | *Apis mellifera* | XP_623194.2 |
| AmSCPYI-2 | *Apis mellifera* | ADD51170.1 |
| AmSCPYI-3 | *Apis mellifera* | XP_006568702.1 |
| ApSCPYI-1 | *Acyrthosiphon pisum* | BAH71163.1 |
| ApSCPYI-2 | *Acyrthosiphon pisum* | BAH71516.1 |
| ApSCPYI-3 | *Acyrthosiphon pisum* | XP_001949401.3 |
| ApSCPYI-4 | *Acyrthosiphon pisum* | XP_008185285.1 |
| ApSCPYI-5 | *Acyrthosiphon pisum* | XP_008186104.1 |
| BmSCPYI-1 | *Bombyx mori* | ABF51354.1 |
| BmSCPYI-2 | *Bombyx mori* | XP_004922756.1 |
| BmSCPYI-3 | *Bombyx mori* | XP_004926491.1 |
| BmSCPYI-4 | *Bombyx mori* | XP_004922749.1 |
| BmSCPYI-5 | *Bombyx mori* | XP_004927224.1 |
| BmSCPYI-6 | *Bombyx mori* | XP_004932727.1 |
| CfSCPYI-1 | *Camponotus floridanus* | CFLO20344 |
| CfSCPYI-2 | *Camponotus floridanus* | CFLO20345 |
| CfSCPYI-3 | *Camponotus floridanus* | CFLO20733 |
| DmSCPYI-1 | *Drosophila melanogaster* | ACL89749.1 |
| DmSCPYI-2 | *Drosophila melanogaster* | ACL89539.1 |
| DmSCPYI-3 | *Drosophila melanogaster* | ACL92153.1 |
| DmSCPYI-4 | *Drosophila melanogaster* | ACL92074.1 |
| DmSCPYI-5 | *Drosophila melanogaster* | ACL89821.1 |
| DmSCPYI-6 | *Drosophila melanogaster* | AAG22206.1 |
| DmSCPYI-7 | *Drosophila melanogaster* | AAF51385.1 |
| DmSCPYI-8 | *Drosophila melanogaster* | ACL91964.1 |
| DmSCPYI-9 | *Drosophila melanogaster* | ACL86902.1 |
| DpSCPYI-1 | *Daphnia pulex* | EFX88098.1 |
| DpSCPYI-2 | *Daphnia pulex* | EFX88254.1 |
| DpSCPYI-3 | *Daphnia pulex* | EFX88099.1 |
| DpSCPYI-4 | *Daphnia pulex* | EFX71296.1 |
| IsSCPYI-1 | *Ixodes scapularis* | ISCW002921 |
| IsSCPYI-2 | *Ixodes scapularis* | ISCW017967 |
| IsSCPYI-3 | *Ixodes scapularis* | ISCW024097 |
| IsSCPYI-4 | *Ixodes scapularis* | ISCW017970 |
| IsSCPYI-5 | *Ixodes scapularis* | ISCW014710 |
| IsSCPYI-6 | *Ixodes scapularis* | ISCW021329 |
| NvSCPYI-1 | *Nasonia vitripennis* | NV13131 |
| NvSCPYI-2 | *Nasonia vitripennis* | NV10952 |
| NvSCPYI-3 | *Nasonia vitripennis* | NV10976 |
| NvSCPYI-4 | *Nasonia vitripennis* | NV13130 |
| NvSCPYI-5 | *Nasonia vitripennis* | NV10975 |
| NvSCPYI-6 | *Nasonia vitripennis* | NV10974 |
| NvSCPYI-7 | *Nasonia vitripennis* | NV13129 |
| NvSCPYI-8 | *Nasonia vitripennis* | NV13295 |
| PhSCPYI-1 | *Pediculus humanus* | PHUM415200 |
| PhSCPYI-2 | *Pediculus humanus* | PHUM239560 |
| PhSCPYI-3 | *Pediculus humanus* | PHUM580880 |
| RpSCPYI-1 | *Rhodnius prolixus* | RPRC009156 |
| RpSCPYI-2 | *Rhodnius prolixus* | RPRC007652 |
| RpSCPYI-3 | *Rhodnius prolixus* | RPRC012355 |
| SmSCPYI-1 | *Strigamia maritima* | SMAR000748 |
| SmSCPYI-2 | *Strigamia maritima* | SMAR009822 |
| TcSCPYI-1 | *Tribolium castaneum* | XP_008200360.1 |
| TcSCPYI-2 | *Tribolium castaneum* | EFA03302.1 |
| TcSCPYI-3 | *Tribolium castaneum* | XP_972374.2 |
| TcSCPYI-4 | *Tribolium castaneum* | XP_972426.2 |
| TcSCPYI-5 | *Tribolium castaneum* | EFA03423.1 |
| TcSCPYI-6 | *Tribolium castaneum* | EFA03424.1 |
| TcSCPYI-7 | *Tribolium castaneum* | EEZ99758.1 |
| TuSCPYI-1 | *Tetranychus urticae* | tetur26g01220 |
| TuSCPYI-2 | *Tetranychus urticae* | tetur26g01230 |
| TuSCPYI-3 | *Tetranychus urticae* | tetur26g00930 |
| TuSCPYI-4 | *Tetranychus urticae* | tetur01g03240 |
| TuSCPYI-5 | *Tetranychus urticae* | tetur12g04350 |
| TuSCPYI-6 | *Tetranychus urticae* | tetur10g04940 |

**I. I63 sequences.**

| **Protein** | **Organism** | **Accession number/Gene model** |
| --- | --- | --- |
| ApLec-1 | *Acyrthosiphon pisum* | XP_001950803.2 |
| ApLec-2 | *Acyrthosiphon pisum* | XP_001943623.2 |
| ApLec-3 | *Acyrthosiphon pisum* | XP_001950366.1 |
| ApLec-4 | *Acyrthosiphon pisum* | BAH70797.1 |
| ApLec-5 | *Acyrthosiphon pisum* | XP_001945032.2 |
| ApLec-6 | *Acyrthosiphon pisum* | XP_001943610.3 |
| DmLec-1 | *Drosophila melanogaster* | AAS93748.1 |
| DmLec-2 | *Drosophila melanogaster* | AGB95184.1 |
| DmLec-3 | *Drosophila melanogaster* | ACL92332.1 |
| DmLec-4 | *Drosophila melanogaster* | AFH03545.1 |
| DmLec-5 | *Drosophila melanogaster* | AFH03543.1 |
| DmLec-6 | *Drosophila melanogaster* | ACL89234.1 |
| DmLec-7 | *Drosophila melanogaster* | ADV37209.1 |
| DmLec-8 | *Drosophila melanogaster* | ACX61618.3 |
| DmLec-9 | *Drosophila melanogaster* | BAD51434.1 |
| DmLec-10 | *Drosophila melanogaster* | ACL91806.1 |
| DmLec-11 | *Drosophila melanogaster* | ACD99527.1 |
| DmLec-12 | *Drosophila melanogaster* | AAF51328.1 |
| DmLec-13 | *Drosophila melanogaster* | AGB93427.1 |
| DmLec-14 | *Drosophila melanogaster* | AAY55758.1 |
| DmLec-15 | *Drosophila melanogaster* | ADV37094.1 |
| DmLec-16 | *Drosophila melanogaster* | AAM52638.1 |
| DmLec-17 | *Drosophila melanogaster* | AAF51471.2 |
| DmLec-18 | *Drosophila melanogaster* | AAF51469.2 |
| DmLec-19 | *Drosophila melanogaster* | ACZ94379.1 |
| DmLec-20 | *Drosophila melanogaster* | BAA12071.1 |
| DmLec-21 | *Drosophila melanogaster* | AEN94442.1 |
| DmLec-22 | *Drosophila melanogaster* | AAY54873.1 |
| DmLec-23 | *Drosophila melanogaster* | AGB92735.1 |
| DmLec-24 | *Drosophila melanogaster* | ACL90840.1 |
| DmLec-25 | *Drosophila melanogaster* | AHN54242.1 |
| DmLec-26 | *Drosophila melanogaster* | AAF53047.2 |
| DmLec-27 | *Drosophila melanogaster* | AAT49107.1 |
| DmLec-28 | *Drosophila melanogaster* | AGB93604.1 |
| DmLec-29 | *Drosophila melanogaster* | AAT49133.1 |
| DmLec-30 | *Drosophila melanogaster* | CAB53208.1 |
| DmLec-31 | *Drosophila melanogaster* | ABV82230.1 |
| DmLec-32 | *Drosophila melanogaster* | ACL86923.1 |
| DpLec-1 | *Daphnia pulex* | EFX85039.1 |
| DpLec-2 | *Daphnia pulex* | EFX83034.1 |
| DpLec-3 | *Daphnia pulex* | EFX80424.1 |
| DpLec-4 | *Daphnia pulex* | EFX82991.1 |
| DpLec-5 | *Daphnia pulex* | EFX66724.1 |
| DpLec-6 | *Daphnia pulex* | EFX64507.1 |
| DpLec-7 | *Daphnia pulex* | EFX63059.1 |
| DpLec-8 | *Daphnia pulex* | EFX80123.1 |
| DpLec-9 | *Daphnia pulex* | EFX83128.1 |
| DpLec-10 | *Daphnia pulex* | EFX89810.1 |
| DpLec-11 | *Daphnia pulex* | EFX73079.1 |
| DpLec-12 | *Daphnia pulex* | EFX70787.1 |
| DpLec-13 | *Daphnia pulex* | EFX71125.1 |
| DpLec-14 | *Daphnia pulex* | EFX79085.1 |
| DpLec-15 | *Daphnia pulex* | EFX82923.1 |
| DpLec-16 | *Daphnia pulex* | EFX65557.1 |
| DpLec-17 | *Daphnia pulex* | EFX69792.1 |
| DpLec-18 | *Daphnia pulex* | EFX76485.1 |
| DpLec-19 | *Daphnia pulex* | EFX87034.1 |
| DpLec-20 | *Daphnia pulex* | EFX71126.1 |
| DpLec-21 | *Daphnia pulex* | EFX88108.1 |
| DpLec-22 | *Daphnia pulex* | EFX67863.1 |
| DpLec-23 | *Daphnia pulex* | EFX74454.1 |
| DpLec-24 | *Daphnia pulex* | EFX76445.1 |
| DpLec-25 | *Daphnia pulex* | EFX88107.1 |
| DpLec-26 | *Daphnia pulex* | EFX88109.1 |
| DpLec-27 | *Daphnia pulex* | EFX88110.1 |
| DpLec-28 | *Daphnia pulex* | EFX79503.1 |
| DpLec-29 | *Daphnia pulex* | EFX86648.1 |
| SmLec-1 | *Strigamia maritima* | [SMAR004728](http://metazoa.ensembl.org/Strigamia_maritima/Gene/Summary?db=core;g=SMAR004728;r=AFFK01019414:964-5378;t=SMAR004728-RA;tl=AYSNhIEf8tyYwEAg-414966-2024492) |
| SmLec-2 | *Strigamia maritima* | SMAR010033 |
| SmLec-3 | *Strigamia maritima* | [SMAR012094](http://metazoa.ensembl.org/Strigamia_maritima/Gene/Summary?db=core;g=SMAR004728;r=AFFK01019414:964-5378;t=SMAR004728-RA;tl=AYSNhIEf8tyYwEAg-414966-2024492) |
| SmLec-4 | *Strigamia maritima* | [SMAR005267](http://metazoa.ensembl.org/Strigamia_maritima/Gene/Summary?db=core;g=SMAR004728;r=AFFK01019414:964-5378;t=SMAR004728-RA;tl=AYSNhIEf8tyYwEAg-414966-2024492) |
| SmLec-5 | *Strigamia maritima* | SMAR013259 |
| SmLec-6 | *Strigamia maritima* | [SMAR007751](http://metazoa.ensembl.org/Strigamia_maritima/Gene/Summary?db=core;g=SMAR004728;r=AFFK01019414:964-5378;t=SMAR004728-RA;tl=AYSNhIEf8tyYwEAg-414966-2024492) |
| SmLec-7 | *Strigamia maritima* | [SMAR005914](http://metazoa.ensembl.org/Strigamia_maritima/Gene/Summary?db=core;g=SMAR004728;r=AFFK01019414:964-5378;t=SMAR004728-RA;tl=AYSNhIEf8tyYwEAg-414966-2024492) |
| SmLec-8 | *Strigamia maritima* | [SMAR009658](http://metazoa.ensembl.org/Strigamia_maritima/Gene/Summary?db=core;g=SMAR004728;r=AFFK01019414:964-5378;t=SMAR004728-RA;tl=AYSNhIEf8tyYwEAg-414966-2024492) |
| SmLec-9 | *Strigamia maritima* | [SMAR008714](http://metazoa.ensembl.org/Strigamia_maritima/Gene/Summary?db=core;g=SMAR004728;r=AFFK01019414:964-5378;t=SMAR004728-RA;tl=AYSNhIEf8tyYwEAg-414966-2024492) |
| SmLec-10 | *Strigamia maritima* | SMAR010073 |
| SmLec-11 | *Strigamia maritima* | SMAR006202 |
| SmLec-12 | *Strigamia maritima* | SMAR002047 |
| SmLec-13 | *Strigamia maritima* | [SMAR004729](http://metazoa.ensembl.org/Strigamia_maritima/Gene/Summary?db=core;g=SMAR004728;r=AFFK01019414:964-5378;t=SMAR004728-RA;tl=AYSNhIEf8tyYwEAg-414966-2024492) |
| SmLec-14 | *Strigamia maritima* | [SMAR007754](http://metazoa.ensembl.org/Strigamia_maritima/Gene/Summary?db=core;g=SMAR004728;r=AFFK01019414:964-5378;t=SMAR004728-RA;tl=AYSNhIEf8tyYwEAg-414966-2024492) |
| SmLec-15 | *Strigamia maritima* | [SMAR004884](http://metazoa.ensembl.org/Strigamia_maritima/Gene/Summary?db=core;g=SMAR004728;r=AFFK01019414:964-5378;t=SMAR004728-RA;tl=AYSNhIEf8tyYwEAg-414966-2024492) |
| SmLec-16 | *Strigamia maritima* | SMAR013086 |
| SmLec-17 | *Strigamia maritima* | SMAR011363 |
| SmLec-18 | *Strigamia maritima* | [SMAR013084](http://metazoa.ensembl.org/Strigamia_maritima/Gene/Summary?db=core;g=SMAR004728;r=AFFK01019414:964-5378;t=SMAR004728-RA;tl=AYSNhIEf8tyYwEAg-414966-2024492) |
| SmLec-19 | *Strigamia maritima* | [SMAR014727](http://metazoa.ensembl.org/Strigamia_maritima/Gene/Summary?db=core;g=SMAR004728;r=AFFK01019414:964-5378;t=SMAR004728-RA;tl=AYSNhIEf8tyYwEAg-414966-2024492) |
| SmLec-20 | *Strigamia maritima* | [SMAR002311](http://metazoa.ensembl.org/Strigamia_maritima/Gene/Summary?db=core;g=SMAR004728;r=AFFK01019414:964-5378;t=SMAR004728-RA;tl=AYSNhIEf8tyYwEAg-414966-2024492) |
| TuLec-1 | *Tetranychus urticae* | tetur13g01750 |
| TuLec-2 | *Tetranychus urticae* | tetur02g12240 |
| TuLec-3 | *Tetranychus urticae* | tetur17g03220 |
| TuLec-4 | *Tetranychus urticae* | tetur17g03290 |

**G. I87 sequences.**

| **Protein** | **Organism** | **Accession number/Gene model** |
| --- | --- | --- |
| AgBand7-1 | *Anopheles gambiae* | AGAP004871 |
| AgBand7-2 | *Anopheles gambiae* | AGAP003352 |
| AgBand7-3 | *Anopheles gambiae* | AGAP009439 |
| AgBand7-4 | *Anopheles gambiae* | AGAP009323 |
| AgBand7-5 | *Anopheles gambiae* | AGAP007494 |
| AmBand7-1 | *Apis mellifera* | XP_006565717.1 |
| AmBand7-2 | *Apis mellifera* | XP_006568845.1 |
| AmBand7-3 | *Apis mellifera* | XP_395784.2 |
| AmBand7-4 | *Apis mellifera* | XP_006559391.1 |
| AmBand7-5 | *Apis mellifera* | XP_006611149.1 |
| AmBand7-6 | *Apis mellifera* | XP_624330.3 |
| AmBand7-7 | *Apis mellifera* | XP_006610674.1 |
| ApBand7-1 | *Acyrthosiphon pisum* | XP_001943813.2 |
| ApBand7-2 | *Acyrthosiphon pisum* | XP_001944404.1 |
| ApBand7-3 | *Acyrthosiphon pisum* | XP_001943498.1 |
| ApBand7-4 | *Acyrthosiphon pisum* | NP_001119688.1 |
| ApBand7-5 | *Acyrthosiphon pisum* | XP_001951716.2 |
| ApBand7-6 | *Acyrthosiphon pisum* | NP_001155675.1 |
| BmBand7-1 | *Bombyx mori* | XP_004932048.1 |
| BmBand7-2 | *Bombyx mori* | XP_004932882.1 |
| BmBand7-3 | *Bombyx mori* | NP_001040289.1 |
| BmBand7-4 | *Bombyx mori* | XP_004929936.1 |
| CfBand7-1 | *Camponotus floridanus* | CFLO20707 |
| CfBand7-2 | *Camponotus floridanus* | CFLO20707 |
| CfBand7-3 | *Camponotus floridanus* | CFLO23214 |
| DmBand7-1 | *Drosophila melanogaster* | NP_001261420.1 |
| DmBand7-2 | *Drosophila melanogaster* | NP_996512.1 |
| DmBand7-3 | *Drosophila melanogaster* | NP_573357.1 |
| DmBand7-4 | *Drosophila melanogaster* | NP_731667.2 |
| DmBand7-5 | *Drosophila melanogaster* | NP_001287292.1 |
| DmBand7-6 | *Drosophila melanogaster* | NP_001262246.1 |
| DmBand7-7 | *Drosophila melanogaster* | NP_001261161.1 |
| DmBand7-8 | *Drosophila melanogaster* | NP_001163012.2 |
| DmBand7-9 | *Drosophila melanogaster* | NP_477358.1 |
| DmBand7-10 | *Drosophila melanogaster* | NP_001097371.1 |
| DpBand7-1 | *Daphnia pulex* | EFX85897.1 |
| DpBand7-2 | *Daphnia pulex* | EFX85707.1 |
| DpBand7-3 | *Daphnia pulex* | EFX89890.1 |
| DpBand7-4 | *Daphnia pulex* | EFX77060.1 |
| IsBand7-1 | *Ixodes scapularis* | ISCW002156 |
| IsBand7-2 | *Ixodes scapularis* | ISCW013233 |
| IsBand7-3 | *Ixodes scapularis* | ISCW012132 |
| IsBand7-4 | *Ixodes scapularis* | ISCW019104 |
| IsBand7-5 | *Ixodes scapularis* | ISCW016005 |
| IsBand7-6 | *Ixodes scapularis* | ISCW007970 |
| IsBand7-7 | *Ixodes scapularis* | ISCW016037 |
| IsBand7-8 | *Ixodes scapularis* | ISCW001788 |
| IsBand7-9 | *Ixodes scapularis* | ISCW021457 |
| NvBand7-1 | *Nasonia vitripennis* | NV14377 |
| NvBand7-2 | *Nasonia vitripennis* | NV10014 |
| NvBand7-3 | *Nasonia vitripennis* | NV13699 |
| NvBand7-4 | *Nasonia vitripennis* | NV10743 |
| PhBand7-1 | *Pediculus humanus* | PHUM582220 |
| PhBand7-2 | *Pediculus humanus* | PHUM491220 |
| PhBand7-3 | *Pediculus humanus* | PHUM475220 |
| PhBand7-4 | *Pediculus humanus* | PHUM115890 |
| PhBand7-5 | *Pediculus humanus* | PHUM507380 |
| RpBand7-1 | *Rhodnius prolixus* | RPRC013343 |
| RpBand7-2 | *Rhodnius prolixus* | RPRC013548 |
| RpBand7-3 | *Rhodnius prolixus* | RPRC013340 |
| RpBand7-4 | *Rhodnius prolixus* | RPRC008160 |
| RpBand7-5 | *Rhodnius prolixus* | RPRC011460 |
| RpBand7-6 | *Rhodnius prolixus* | RPRC012129 |
| RpBand7-7 | *Rhodnius prolixus* | RPRC009384 |
| SmBand7-1 | *Strigamia maritima* | SMAR004199 |
| SmBand7-2 | *Strigamia maritima* | SMAR004891 |
| SmBand7-3 | *Strigamia maritima* | SMAR004602 |
| SmBand7-4 | *Strigamia maritima* | SMAR008880 |
| SmBand7-5 | *Strigamia maritima* | SMAR001126 |
| TcBand7-1 | *Tribolium castaneum* | XP_971694.2 |
| TcBand7-2 | *Tribolium castaneum* | XP_971747.1 |
| TcBand7-3 | *Tribolium castaneum* | XP_008191473.1 |
| TcBand7-4 | *Tribolium castaneum* | XP_008195784.1 |
| TcBand7-5 | *Tribolium castaneum* | XP_974606.1 |
| TcBand7-6 | *Tribolium castaneum* | XP_008194845.1 |
| TuBand7-1 | *Tetranychus urticae* | tetur12g00490 |
| TuBand7-2 | *Tetranychus urticae* | tetur10g02430 |
| TuBand7-3 | *Tetranychus urticae* | tetur12g04360 |
| TuBand7-4 | *Tetranychus urticae* | tetur14g01720 |
| TuBand7-5 | *Tetranychus urticae* | tetur02g02060 |
| TuBand7-6 | *Tetranychus urticae* | tetur17g01120 |
| TuBand7-7 | *Tetranychus urticae* | tetur17g01130 |
| TuBand7-8 | *Tetranychus urticae* | tetur01g13950 |
| TuBand7-9 | *Tetranychus urticae* | tetur17g01140 |
| TuBand7-10 | *Tetranychus urticae* | tetur06g06260 |
| TuBand7-11 | *Tetranychus urticae* | tetur18g03240 |
| TuBand7-12 | *Tetranychus urticae* | tetur19g00750 |
| TuBand7-13 | *Tetranychus urticae* | tetur02g04520 |
| TuBand7-14 | *Tetranychus urticae* | tetur07g07030 |

**G. I93 sequences.**

| **Protein** | **Organism** | **Accession number/Gene model** |
| --- | --- | --- |
| AgFz-1 | *Anopheles gambiae* | AGAP008871 |
| AgFz-2 | *Anopheles gambiae* | AGAP010442 |
| AgFz-3 | *Anopheles gambiae* | AGAP011379 |
| AgFz-4 | *Anopheles gambiae* | AGAP006631 |
| AgFz-5 | *Anopheles gambiae* | AGAP000303 |
| AgFz-6 | *Anopheles gambiae* | AGAP008625 |
| AmFz-1 | *Apis mellifera* | XP_006559293.1 |
| AmFz-2 | *Apis mellifera* | XP_006613539.1 |
| AmFz-3 | *Apis mellifera* | XP_006564530.1 |
| AmFz-4 | *Apis mellifera* | XP_006563975.1 |
| AmFz-5 | *Apis mellifera* | XP_006557894.1 |
| ApFz-1 | *Acyrthosiphon pisum* | XP_001951740.2 |
| ApFz-2 | *Acyrthosiphon pisum* | XP_008188372.1 |
| ApFz-3 | *Acyrthosiphon pisum* | XP_001943859.1 |
| ApFz-4 | *Acyrthosiphon pisum* | XP_008186524.1 |
| BmFz-1 | *Bombyx mori* | XP_004927411.1 |
| BmFz-2 | *Bombyx mori* | XP_004926317.1 |
| BmFz-3 | *Bombyx mori* | XP_004929280.1 |
| BmFz-4 | *Bombyx mori* | XP_004923924.1 |
| BmFz-5 | *Bombyx mori* | XP_004923982.1 |
| CfFz-1 | *Camponotus floridanus* | CFLO17421 |
| CfFz-2 | *Camponotus floridanus* | CFLO12283 |
| CfFz-3 | *Camponotus floridanus* | CFLO21130 |
| CfFz-4 | *Camponotus floridanus* | CFLO15364 |
| CfFz-5 | *Camponotus floridanus* | CFLO15916 |
| CfFz-6 | *Camponotus floridanus* | CFLO22144 |
| CfFz-7 | *Camponotus floridanus* | CFLO17965 |
| DmFz-1 | *Drosophila melanogaster* | XP_002030476.1 |
| DmFz-2 | *Drosophila melanogaster* | NP_727170.2 |
| DmFz-3 | *Drosophila melanogaster* | NP_001262037.1 |
| DmFz-4 | *Drosophila melanogaster* | NP_001284760.1 |
| DmFz-5 | *Drosophila melanogaster* | NP_001245577.1 |
| DmFz-6 | *Drosophila melanogaster* | NP_610297.2 |
| DmFz-7 | *Drosophila melanogaster* | NP_609130.1 |
| DpFz-1 | *Daphnia pulex* | EFX90356.1 |
| DpFz-2 | *Daphnia pulex* | EFX86266.1 |
| DpFz-3 | *Daphnia pulex* | EFX87638.1 |
| DpFz-4 | *Daphnia pulex* | EFX80809.1 |
| DpFz-5 | *Daphnia pulex* | EFX70349.1 |
| DpFz-6 | *Daphnia pulex* | EFX79362.1 |
| IsFz-1 | *Ixodes scapularis* | ISCW003981 |
| IsFz-2 | *Ixodes scapularis* | ISCW016122 |
| IsFz-3 | *Ixodes scapularis* | ISCW004077 |
| IsFz-4 | *Ixodes scapularis* | ISCW003177 |
| IsFz-5 | *Ixodes scapularis* | ISCW021763 |
| NvFz-1 | *Nasonia vitripennis* | NV13851 |
| NvFz-2 | *Nasonia vitripennis* | NV18922 |
| NvFz-3 | *Nasonia vitripennis* | NV16169 |
| NvFz-4 | *Nasonia vitripennis* | NV11450 |
| NvFz-5 | *Nasonia vitripennis* | NV12697 |
| NvFz-6 | *Nasonia vitripennis* | NV11077 |
| PhFz-1 | *Pediculus humanus* | PHUM440660 |
| PhFz-2 | *Pediculus humanus* | PHUM188130 |
| PhFz-3 | *Pediculus humanus* | PHUM074510 |
| PhFz-4 | *Pediculus humanus* | PHUM024690 |
| PhFz-5 | *Pediculus humanus* | PHUM000430 |
| PhFz-6 | *Pediculus humanus* | PHUM454890 |
| RpFz-1 | *Rhodnius prolixus* | RPRC000842 |
| RpFz-2 | *Rhodnius prolixus* | RPRC004621 |
| RpFz-3 | *Rhodnius prolixus* | RPRC010306 |
| RpFz-4 | *Rhodnius prolixus* | RPRC000610 |
| RpFz-5 | *Rhodnius prolixus* | RPRC012094 |
| RpFz-6 | *Rhodnius prolixus* | RPRC004966 |
| SmFz-1 | *Strigamia maritima* | SMAR013121 |
| SmFz-2 | *Strigamia maritima* | SMAR012389 |
| SmFz-3 | *Strigamia maritima* | SMAR014833 |
| SmFz-4 | *Strigamia maritima* | SMAR007293 |
| SmFz-5 | *Strigamia maritima* | SMAR009650 |
| SmFz-6 | *Strigamia maritima* | SMAR015318 |
| SmFz-7 | *Strigamia maritima* | SMAR008785 |
| SmFz-8 | *Strigamia maritima* | SMAR003941 |
| SmFz-9 | *Strigamia maritima* | SMAR004227 |
| TcFz-1 | *Tribolium castaneum* | NP_001164247.1 |
| TcFz-2 | *Tribolium castaneum* | XP_967603.1 |
| TcFz-3 | *Tribolium castaneum* | XP_008197743.1 |
| TcFz-4 | *Tribolium castaneum* | XP_008199427.1 |
| TcFz-5 | *Tribolium castaneum* | XP_008196961.1 |
| TcFz-6 | *Tribolium castaneum* | XP_008190828.1 |
| TuFz-1 | *Tetranychus urticae* | tetur11g01850 |
| TuFz-2 | *Tetranychus urticae* | tetur20g00140 |
| TuFz-3 | *Tetranychus urticae* | tetur09g03780 |
| TuFz-4 | *Tetranychus urticae* | tetur20g01060 |
| TuFz-5 | *Tetranychus urticae* | tetur03g02450 |
| TuFz-6 | *Tetranychus urticae* | tetur11g01770 |
| TuFz-7 | *Tetranychus urticae* | tetur10g05360 |
