# Supplementary material for: Insights into the molecular evolution of peptidase inhibitors in arthropods
Source: PLoS One. 2017 Nov 6;12(11):e0187643. doi: 10.1371/journal.pone.0187643 (PMC5673224; doi:10.1371/journal.pone.0187643)
Supplement: S4 Fig — (PPT) [file pone.0187643.s004.ppt]

## Slide 1
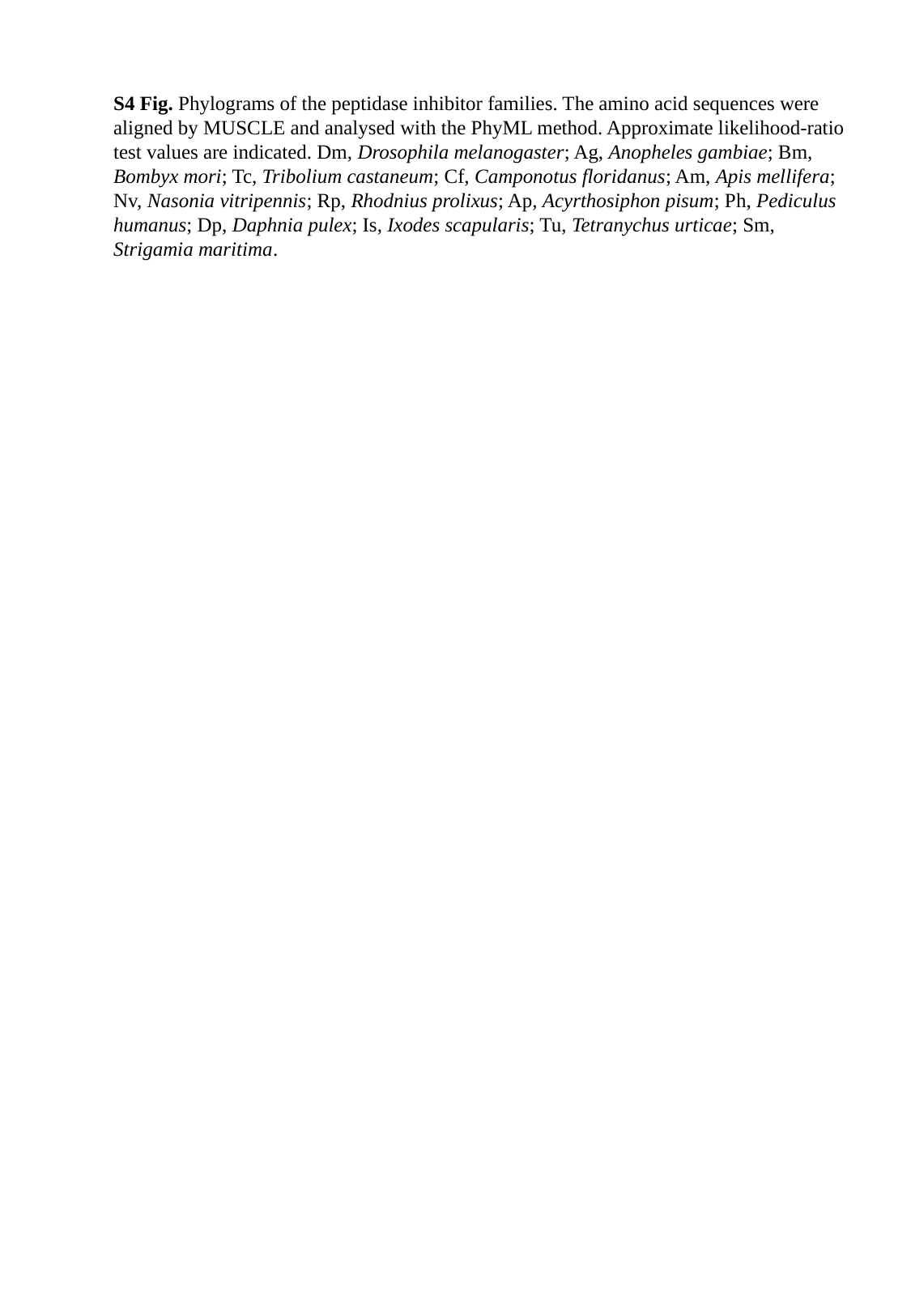

S4 Fig. Phylograms of the peptidase inhibitor families. The amino acid sequences were aligned by MUSCLE and analysed with the PhyML method. Approximate likelihood-ratio test values are indicated. Dm, Drosophila melanogaster; Ag, Anopheles gambiae; Bm, Bombyx mori; Tc, Tribolium castaneum; Cf, Camponotus floridanus; Am, Apis mellifera; Nv, Nasonia vitripennis; Rp, Rhodnius prolixus; Ap, Acyrthosiphon pisum; Ph, Pediculus humanus; Dp, Daphnia pulex; Is, Ixodes scapularis; Tu, Tetranychus urticae; Sm, Strigamia maritima.

## Slide 2
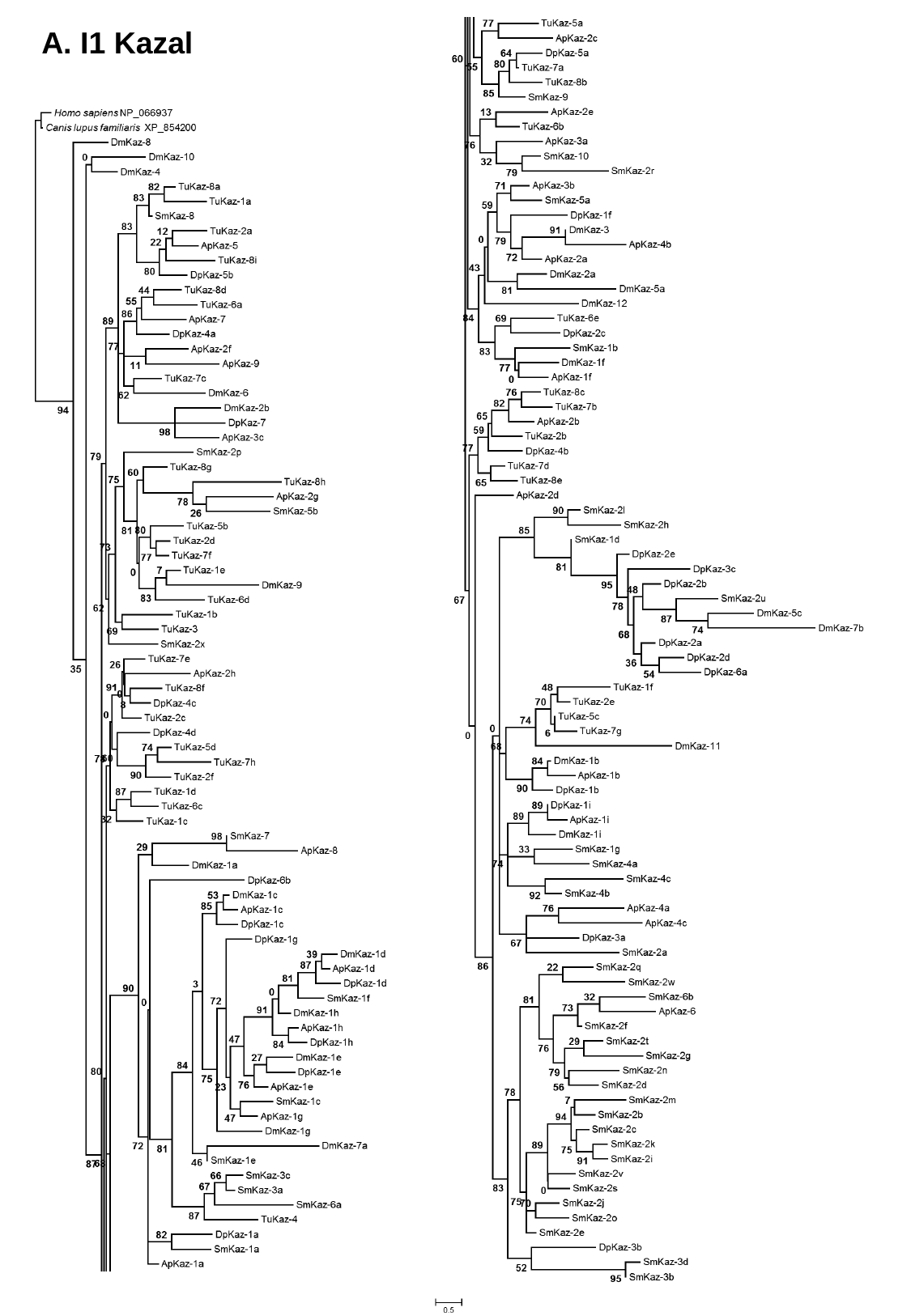

A. I1 Kazal

## Slide 3
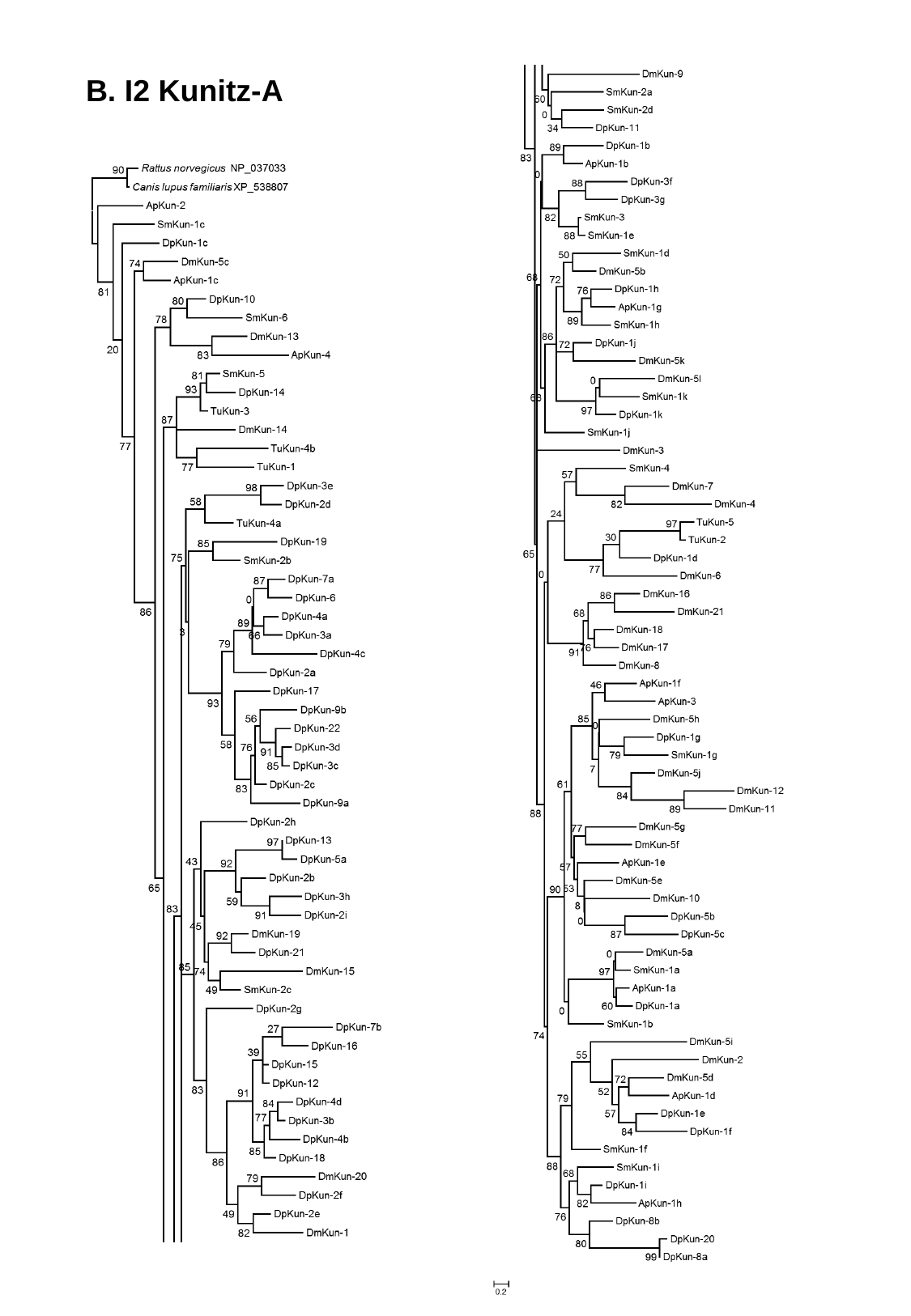

B. I2 Kunitz-A

## Slide 4
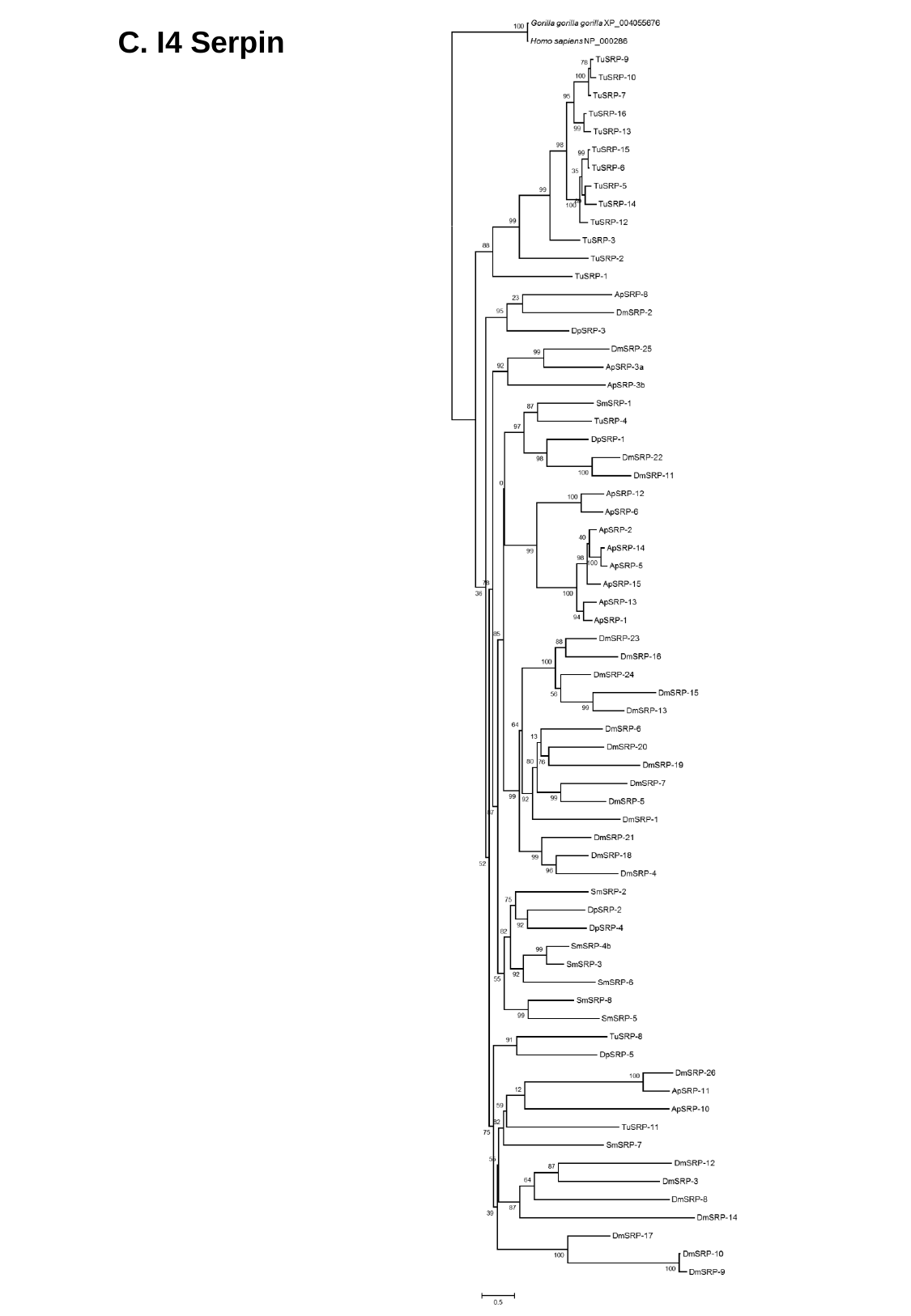

C. I4 Serpin

## Slide 5
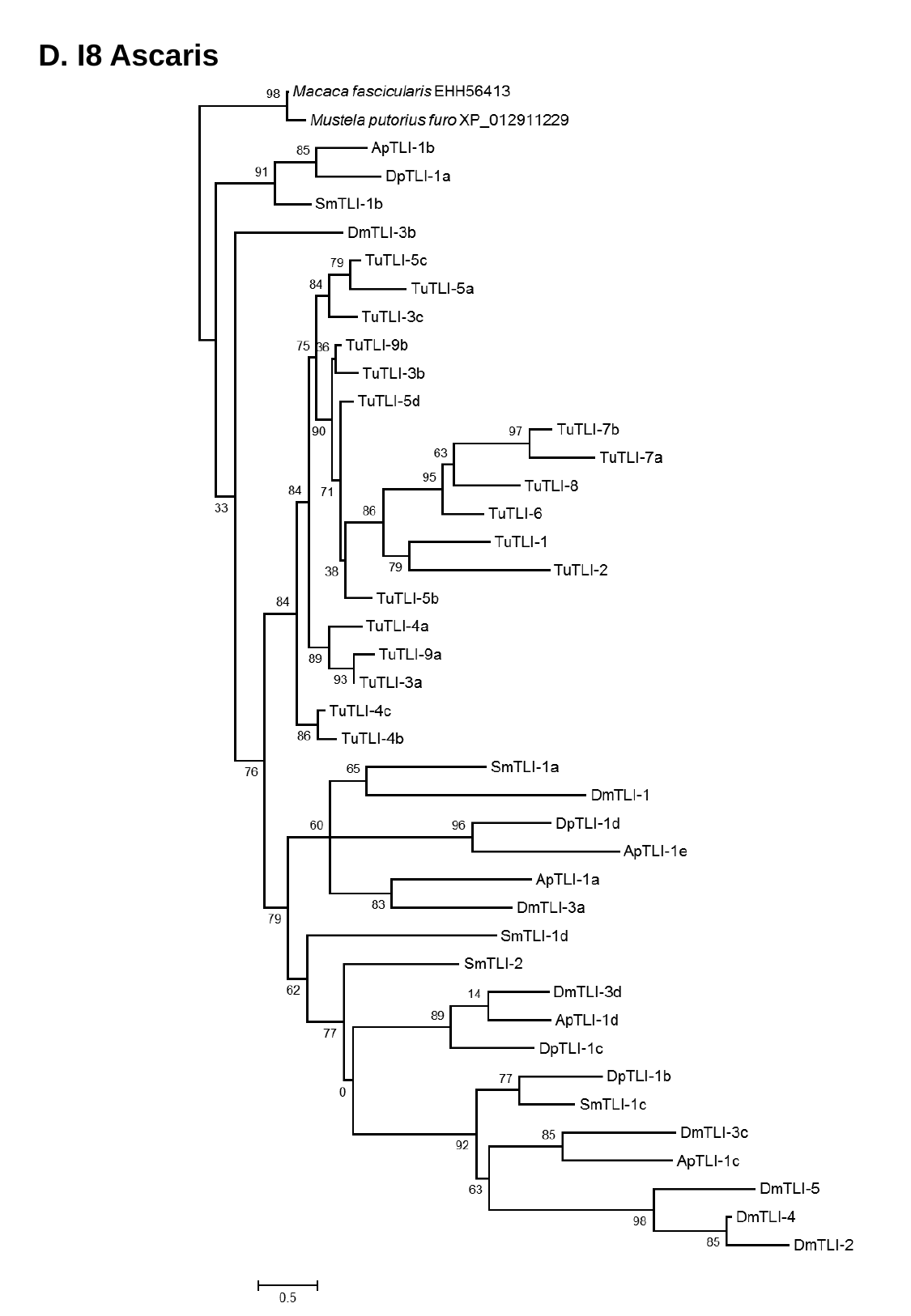

D. I8 Ascaris

## Slide 6
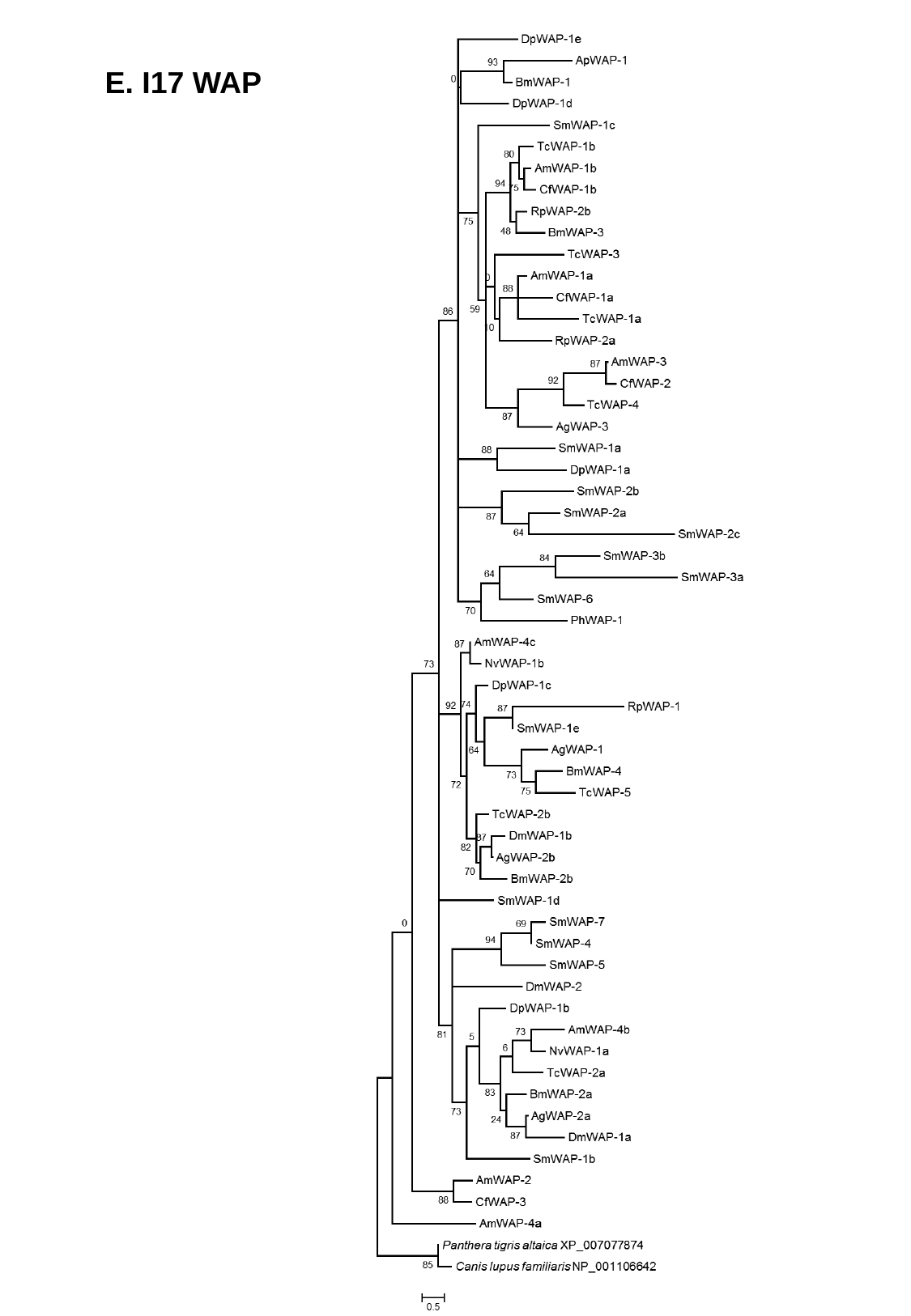

E. I17 WAP

## Slide 7
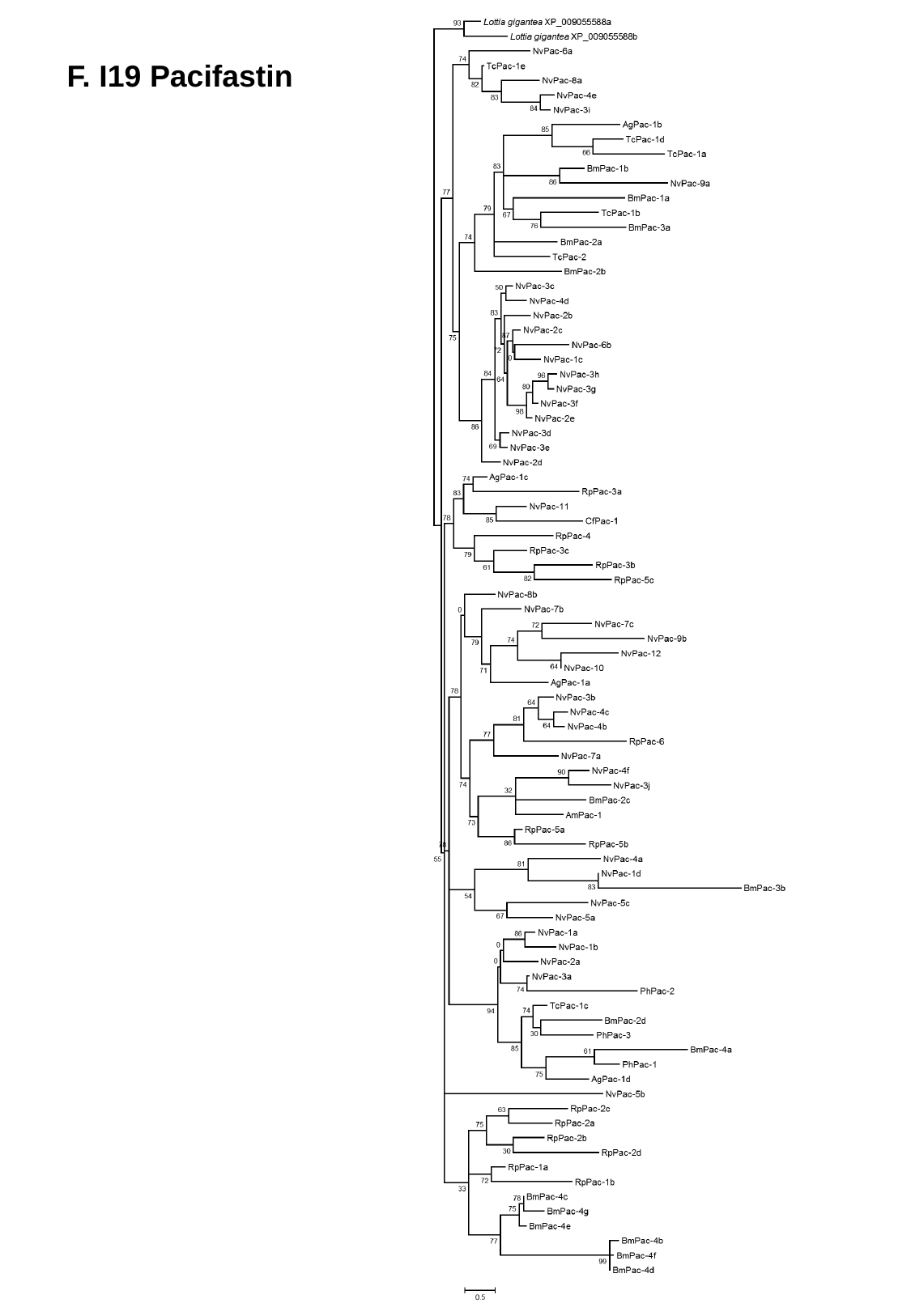

F. I19 Pacifastin

## Slide 8
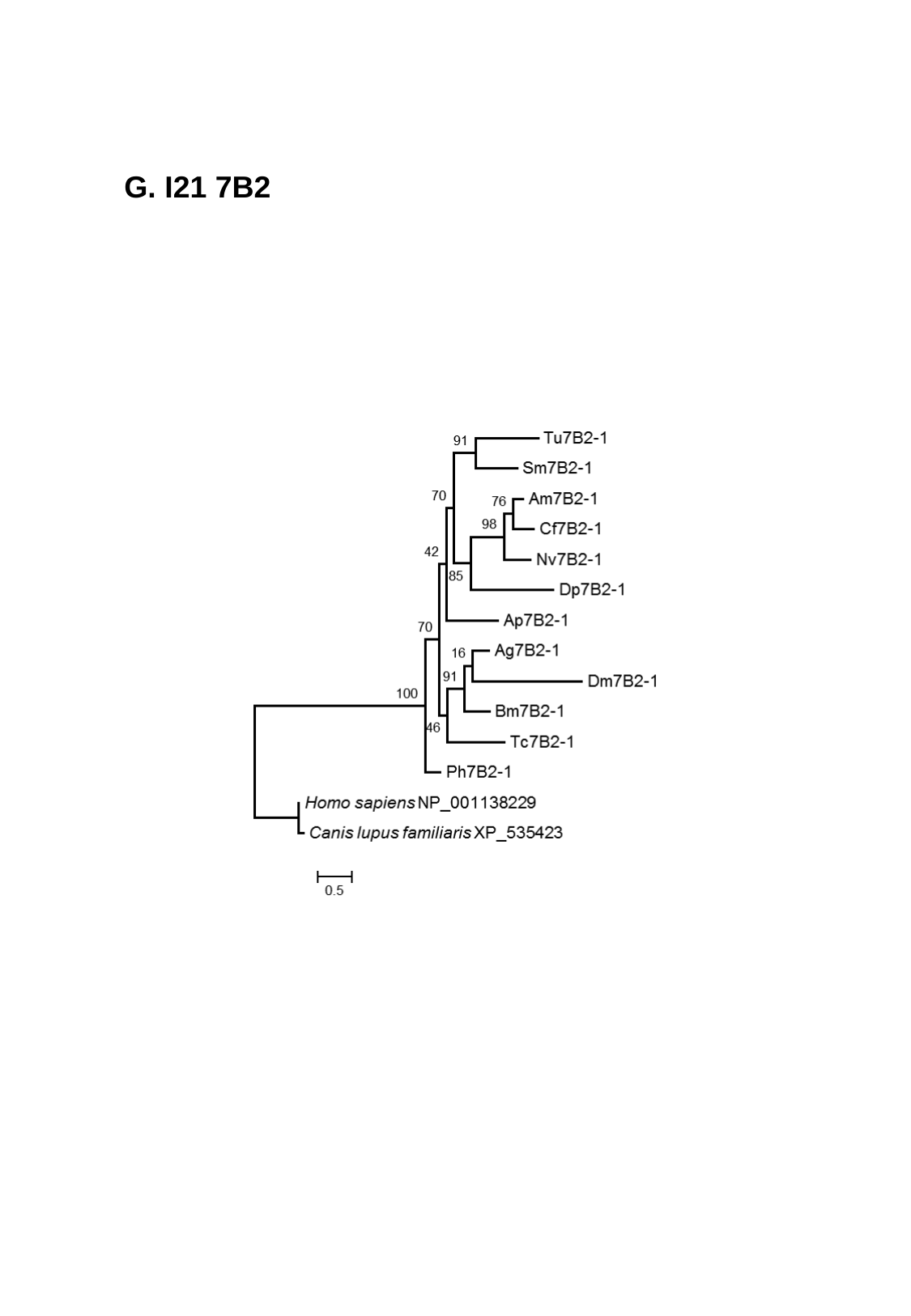

G. I21 7B2

## Slide 9
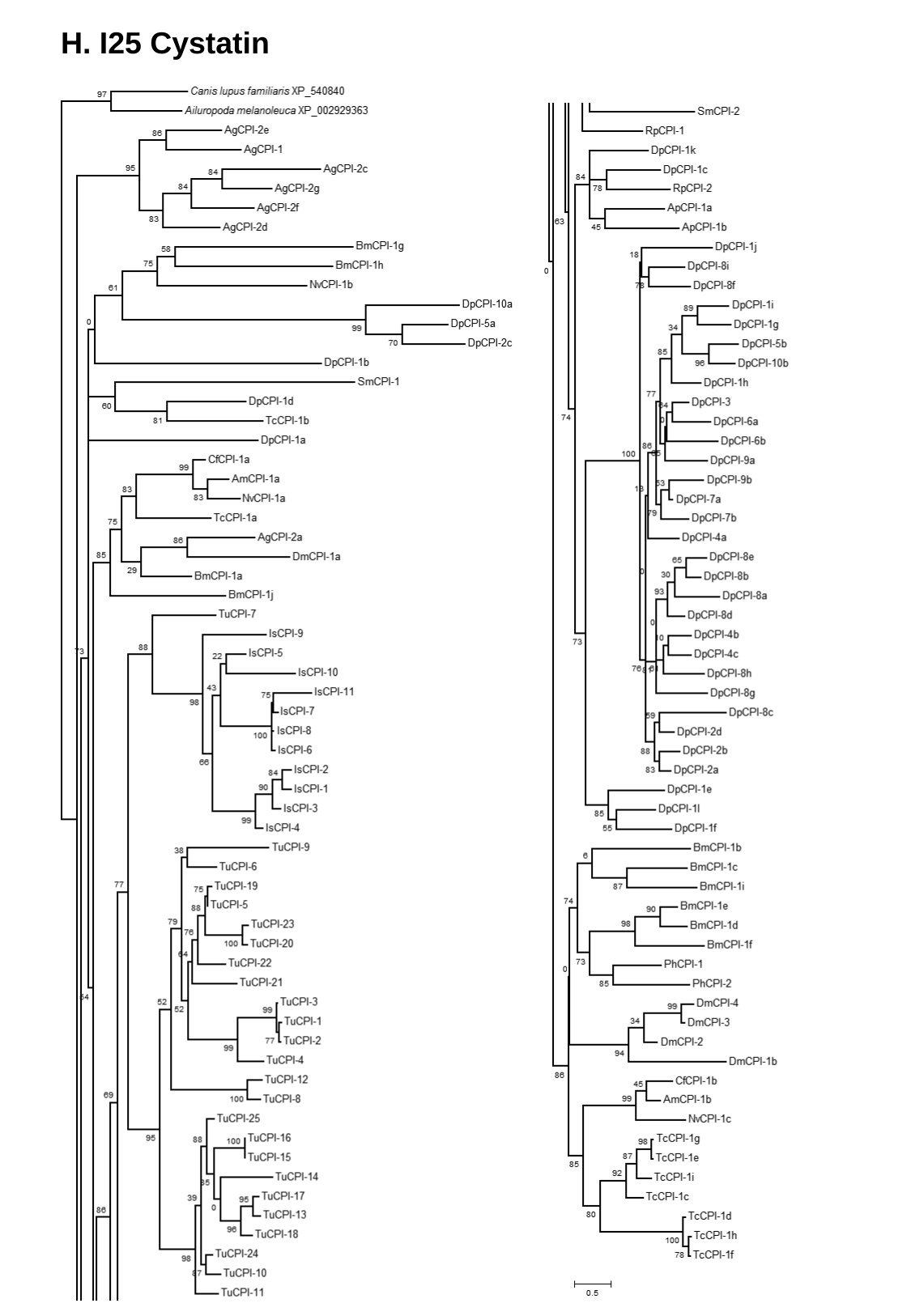

H. I25 Cystatin

## Slide 10
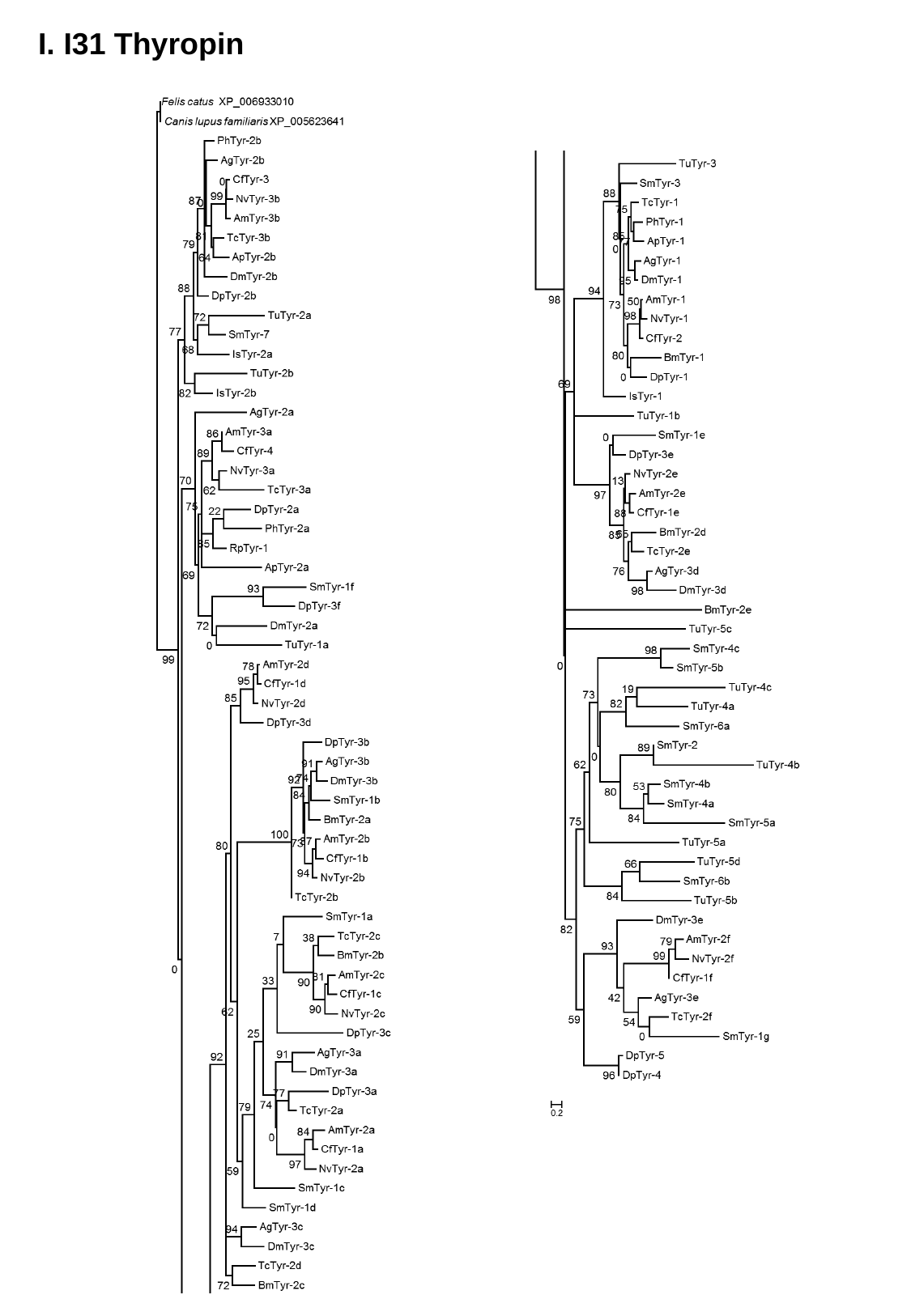

I. I31 Thyropin

## Slide 11
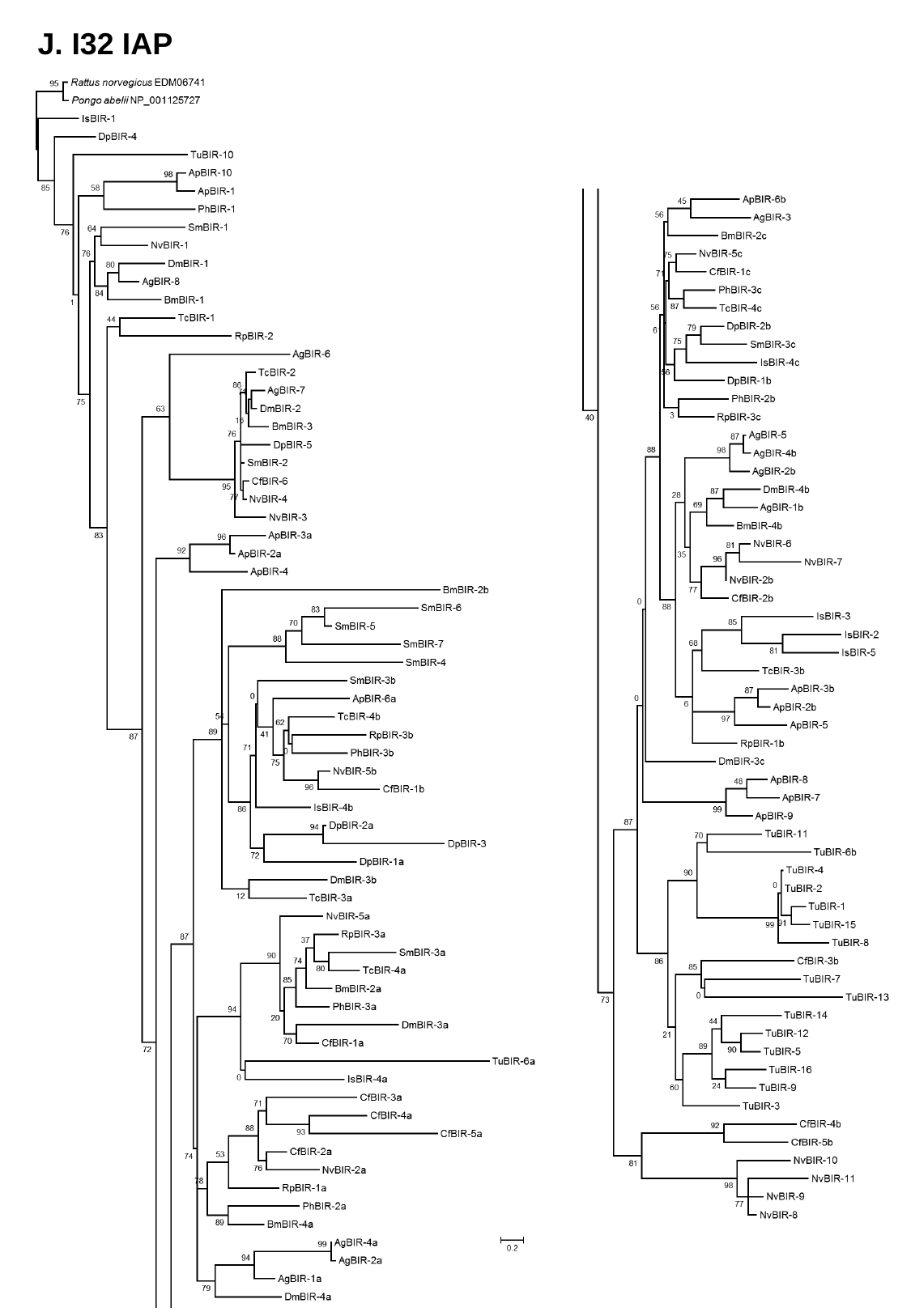

J. I32 IAP

## Slide 12
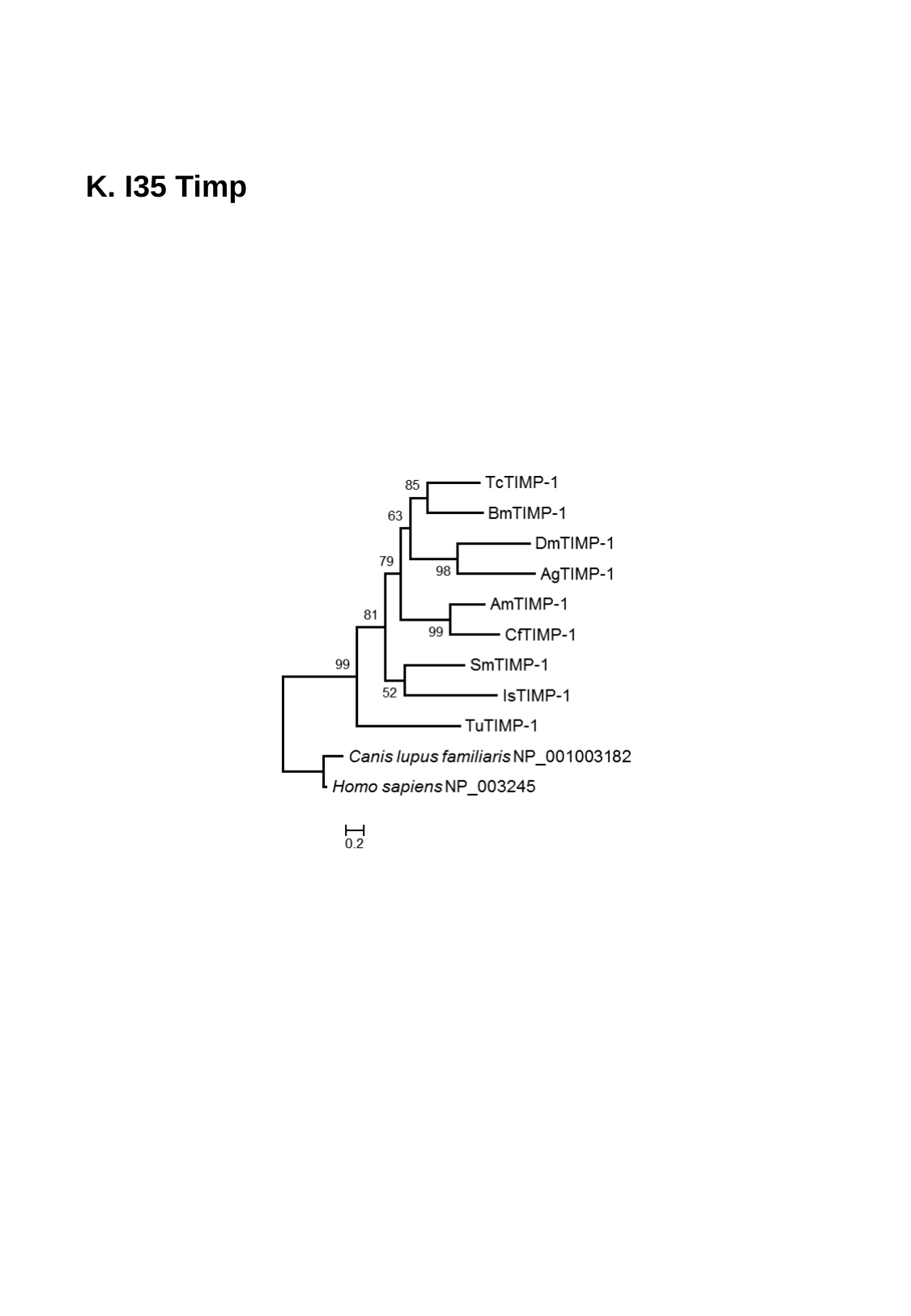

K. I35 Timp

## Slide 13
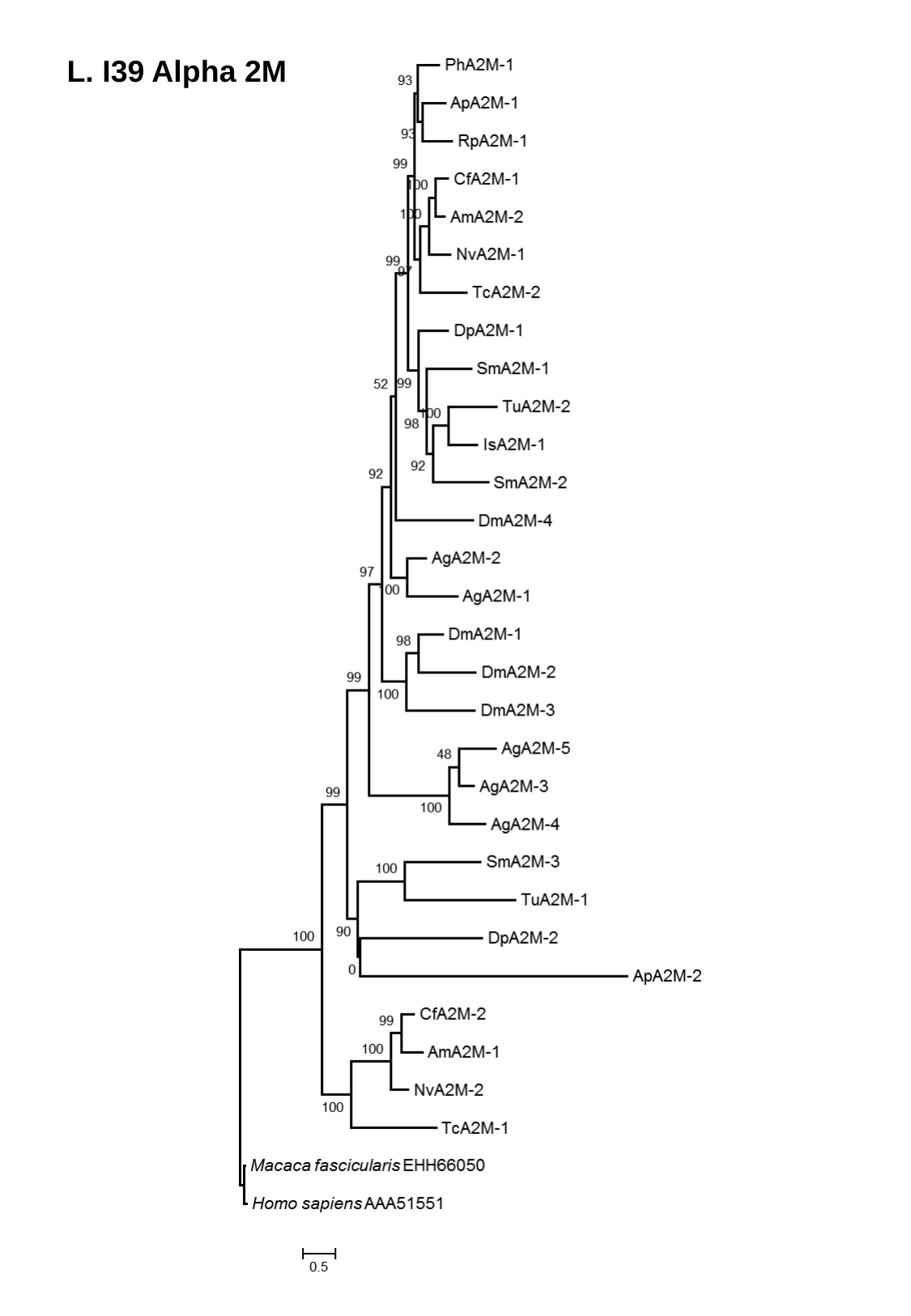

L. I39 Alpha 2M

## Slide 14
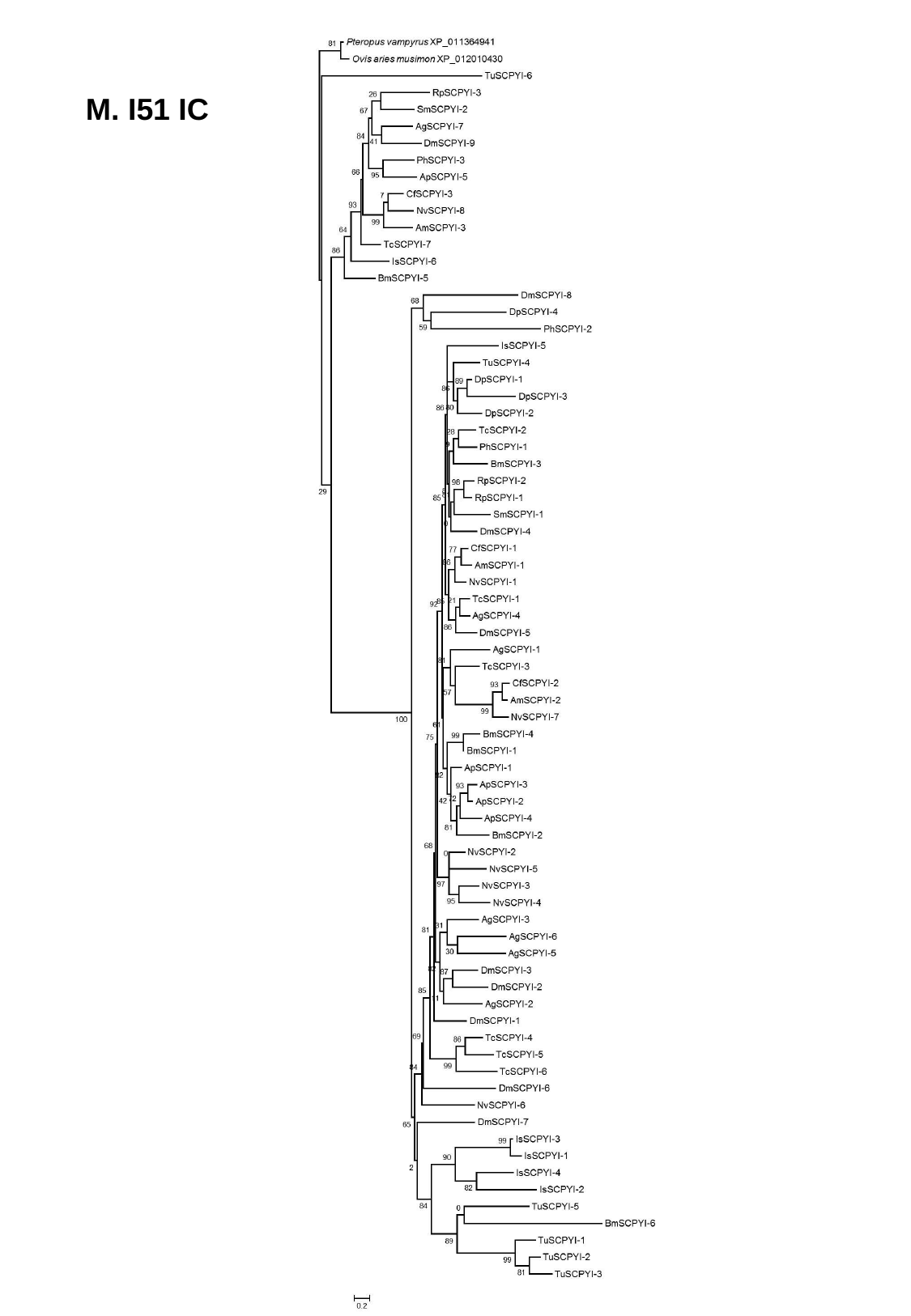

M. I51 IC

## Slide 15
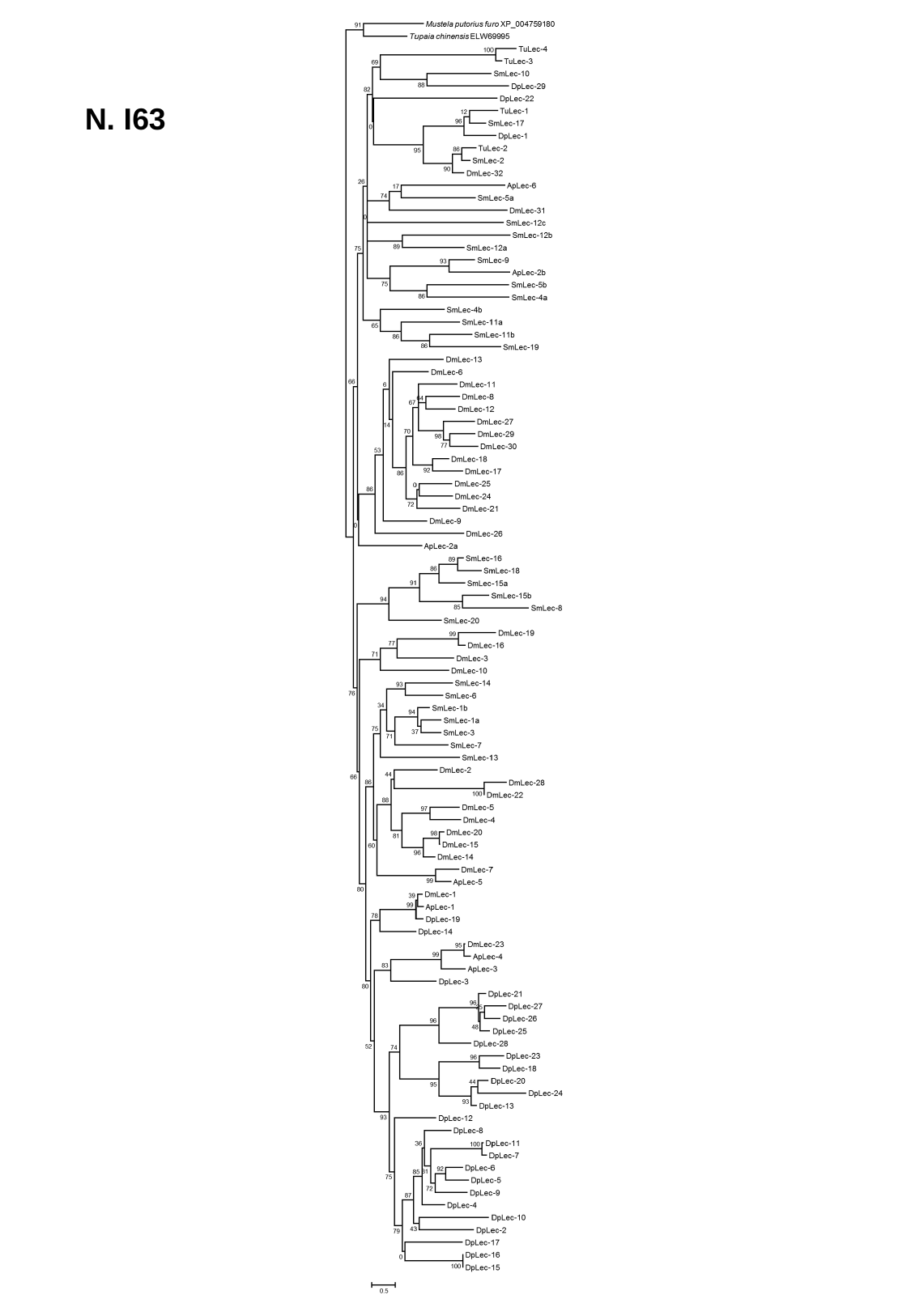

N. I63

## Slide 16
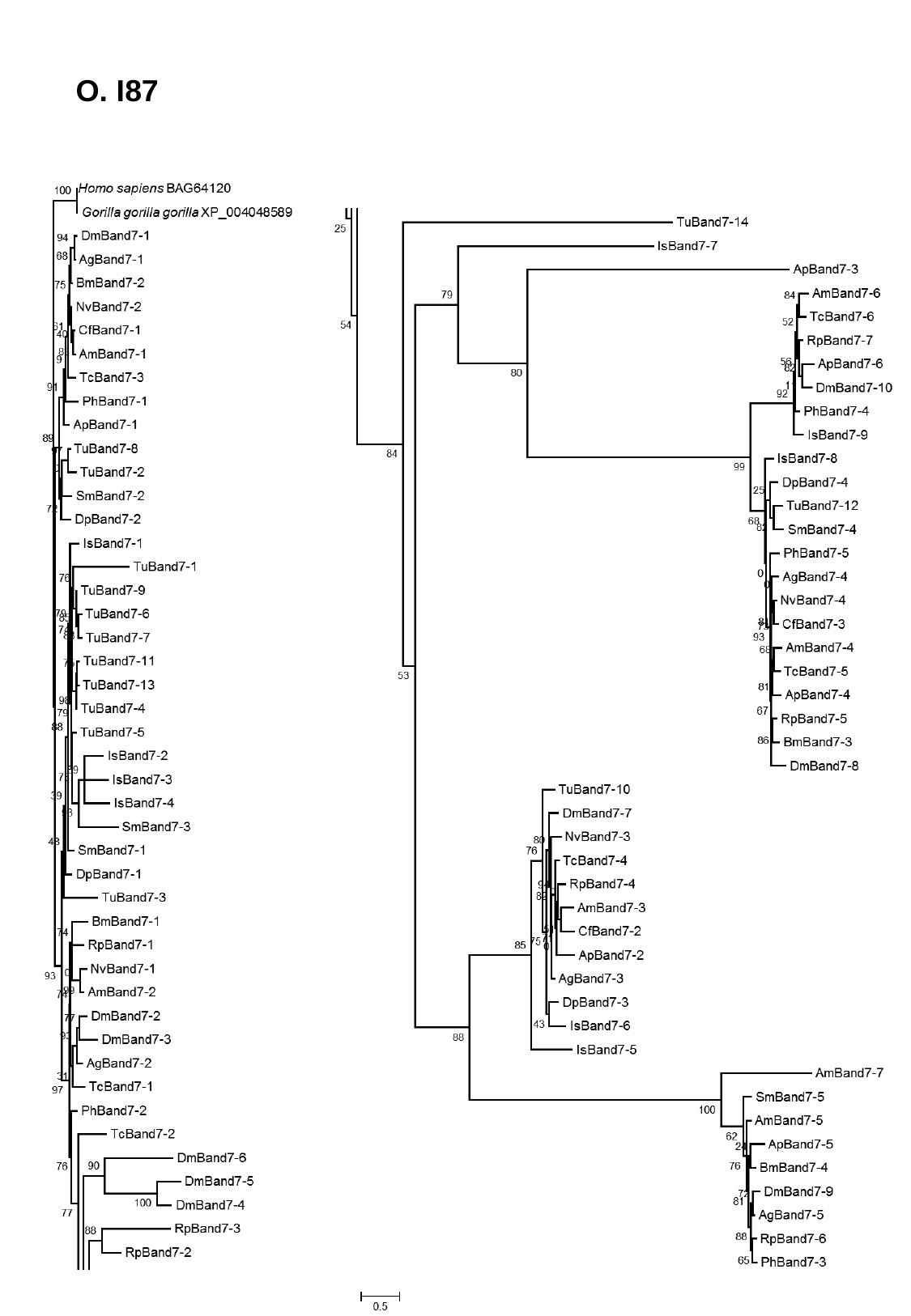

O. I87

## Slide 17
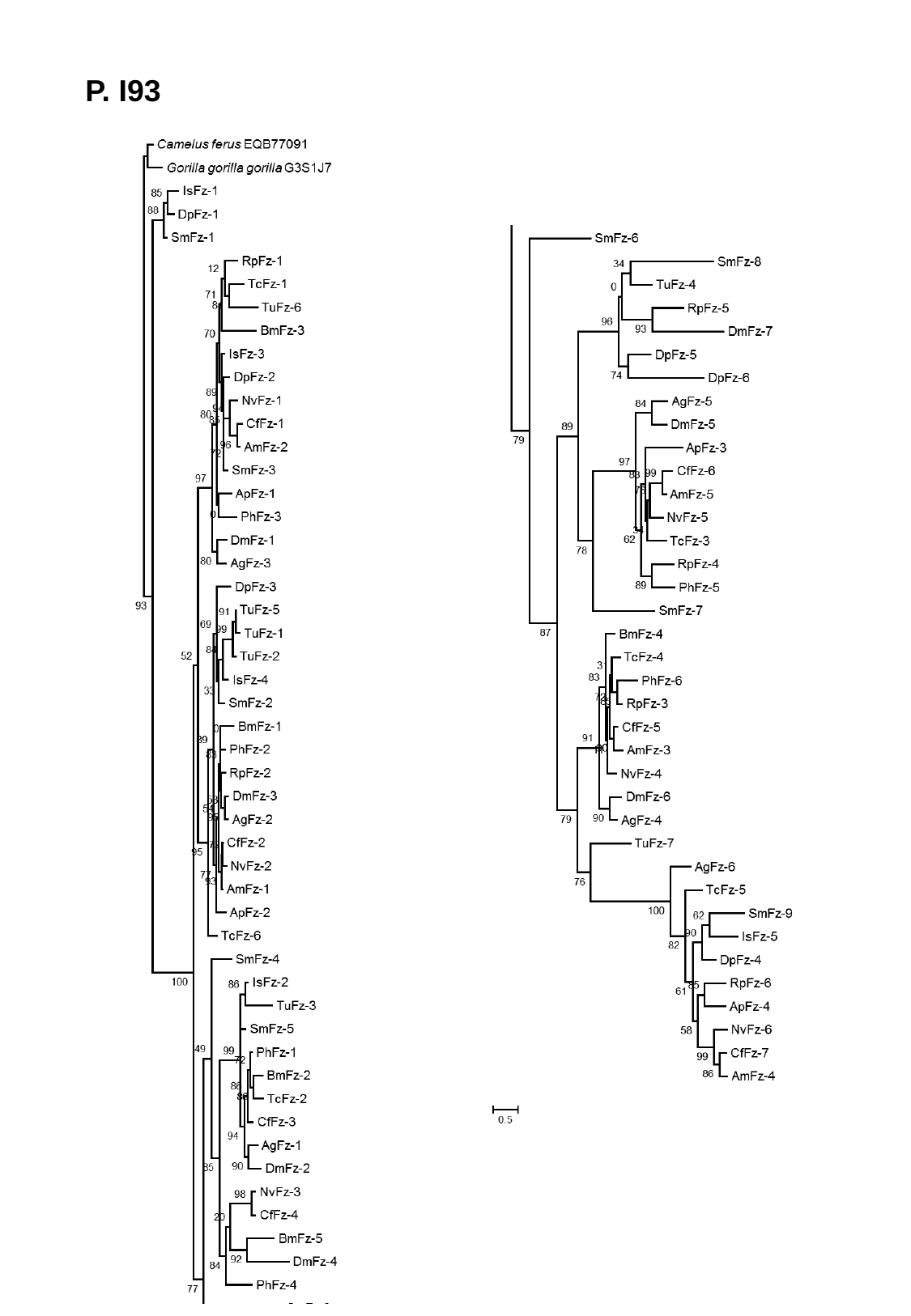

P. I93
