# Supplementary material for: Insights into the molecular evolution of peptidase inhibitors in arthropods
Source: PLoS One. 2017 Nov 6;12(11):e0187643. doi: 10.1371/journal.pone.0187643 (PMC5673224; doi:10.1371/journal.pone.0187643)
Supplement: S1 Table — (DOCX) [file pone.0187643.s005.docx]

| Species | Genome version | Blast page |
| --- | --- | --- |
| *Acyrthosiphon pisum* | Acyr 2.0 (reference annotation release 102) | <http://blast.ncbi.nlm.nih.gov/Blast.cgi> |
| *Anopheles gambiae* | AgamP 4.4 gene set | <https://www.vectorbase.org/blast> |
| *Apis mellifera* | Amel 4.5 (reference annotation release 103) | <http://blast.ncbi.nlm.nih.gov/Blast.cgi> |
| *Bombyx mori* | ASM15162v1 (reference annotation release 101) | <http://blast.ncbi.nlm.nih.gov/Blast.cgi> |
| *Camponotus floridanus* | Cflo 3.3 OGSv3.3 | <http://hymenopteragenome.org/ant_genomes/> |
| *Daphnia pulex* | v 1.0 | <http://blast.ncbi.nlm.nih.gov/Blast.cgi> |
| *Drosophila melanogaster* | Release 6 (reference annotation release 106) | <http://blast.ncbi.nlm.nih.gov/Blast.cgi> |
| *Ixodes scapularis* | IscaW 1.4 gene set | <https://www.vectorbase.org/blast> |
| *Nasonia vitripennis* | Nvit 2.0 OGSv1.2 | <http://hymenopteragenome.org/nasonia/> |
| *Pediculus humanus* | PhumU 2.1 gene set | <https://www.vectorbase.org/blast> |
| *Rhodnius prolixus* | RproC 3.1 gene set | <https://www.vectorbase.org/blast> |
| *Tetranychus urticae* | ASM23943v1 | http://bioinformatics.psb.ugent.be/orcae/overview/Tetur |
| *Tribolium castaneum* | Tcas 5.2 (reference annotation release 103) | <http://blast.ncbi.nlm.nih.gov/Blast.cgi> |
| *Strigamia marítima* | Smar1 | <http://metazoa.ensembl.org/Strigamia_maritima/Info/Index> |

**S1 Table.** Genome versions and web pages where genome Blast searches can be performed for the selected arthropod species.
